# Global Trends of Early, Middle, and Late‐Onset Lung Cancer From 1990 to 2021: Results From the Global Burden of Disease Study 2021

**DOI:** 10.1002/cam4.70639

**Published:** 2025-02-07

**Authors:** Zongyuan Li, Cheng Yu, Jianqi Hao, Nanzhi Luo, Haoning Peng, Jian Zhang, Qiang Pu, Lunxu Liu

**Affiliations:** ^1^ Department of Thoracic Surgery and Institute of Thoracic Oncology, West China Hospital Sichuan University Chengdu Sichuan China; ^2^ West China School of Medicine Sichuan University Chengdu Sichuan China; ^3^ Western China Collaborative Innovation Center for Early Diagnosis and Multidisciplinary Therapy of Lung Cancer Sichuan University Chengdu Sichuan China

**Keywords:** disability‐adjusted life years (DALYs), Global Burden of Disease (GBD), incidence, lung cancer, mortality

## Abstract

**Background:**

Although the global burden of lung cancer has generally declined in recent decades, the variation in onset age‐related trends remains insufficiently explored. In the current study, we aimed to systematically evaluate the most update temporal trends in incidence, mortality and DALYs of early, middle, and late‐onset lung cancer (EOLC, MOLC, and LOLC) from 1990 to 2021, with stratifications of gender, location, and socio‐demographic development.

**Methods:**

We retrieved cross‐sectional data from the Global Burden of Diseases, Injuries, and Risk Factors Study (GBD) 2021. The global, regional, and national burden of lung cancer from 1990 to 2021 was evaluated primarily by age‐standardized rates of incidence (ASIR), mortality (ASMR), and DALYs (ASDR). Joinpoint regression analysis was employed to assess temporal trends and turning point years. Frontier analysis was applied to examine the lowest achievable DALYs, and cross‐country inequalities were evaluated sing the slope index of inequality (SII) and concentration index. We also forecasted the burden from 2022 to 2035.

**Results:**

The global ASIR of EOLC decreased from 4.81 per 100,000 in 1990 to 3.13 in 2021 (AAPC: −1.38, 95% confidence interval [CI]: −1.53 to −1.22, *p* < 0.001), with a steeper decline in males (AAPC: −1.79) compared to females (AAPC: ‐0.63). MOLC incidence also declined from 92.77 to 72.47 per 100,000 (AAPC: ‐0.81, 95% CI: −0.9 to −0.73, *p* < 0.01), while LOLC demonstrated a slight increase from 195.39 to 225.8 per (AAPC: 0.43, 95% CI: 0.37 to 0.5, *p* < 0.01). Notably, LOLC in females showed a consistent rise in incidence rate (AAPC: 1.13, 95% CI: 1.05 to 1.21, *p* < 0.01). In contrast to EOLC and MOLC, 11, 10, and 9 out of 21 GBD regions showed a rising trend for ASIR, ASMR, and ASDR of LOLC, respectively. East Asia showed the steepest increase in ASIR (from 229.26 in 1990 to 375.90 in 2021, AAPC = 1.6, 95% CI: 1.31 to 1.89, *p* < 0.001) of LOLC. Moreover, according to socio‐demographic index (SDI) quintiles, the middle SDI region demonstrated the largest rise in ASIR of LOLC. Frontier analysis revealed that countries with higher SDIs had a greater capacity for reducing lung cancer burdens. Cross‐country inequalities of lung cancer burden in females were found to improve much slower than in males. The projections implied that, although lung cancer would generally decline in the next decade, the incidence, mortality, and DALY rates of LOLC in females might remarkably increase.

**Conclusions:**

The global incidence, mortality, and DALY rates of lung cancer showed a general decline from 1990 to 2021. However, concerning trends of LOLC burden, especially among females and in specific regions or countries, were observed in this study. This study could help to guide more targeted prevention and intervention strategies for lung cancer control.

## 1 Introduction

In 2022, lung cancer ranked as the most commonly diagnosed cancer and the leading cause of cancer‐related mortality around the world, with approximately 2.5 million new cases (12.4%) and 1.8 million deaths (18.7%), contributing to a significant burden on healthcare systems [1]. Although substantial progress has been achieved in the early detection and treatment of lung cancer, the 5‐year overall survival (OS) rate continues to be dismally low at just 25% [2, 3]. Lung cancer, like other malignant tumors, is typically considered as a disease of the elderly, with its incidence and burden projected to increase as life expectancy continues to improve [4]. Meanwhile, with the onset occurring at various stages of life, lung cancer of different age groups could show heterogeneity in epidemiological, clinicopathological and prognostic characteristics [4, 5, 6, 7, 8]. For instance, although nearly 47% of the lung cancer patients are over 70 years old (defined as late‐onset lung cancer, LOLC) [4, 9], the number of individuals diagnosed with lung cancer earlier than 50 years old (defined as early‐onset lung cancer, EOLC) and between 50 and 70 years old (defined as middle‐onset lung cancer, MOLC) also shows an increasing trend [5, 10]. Compared to MOLC and LOLC, patients with earlier‐onset lung cancer are more likely to have a significant genetic predisposition [7, 11], and tend to present with advanced‐stage disease, which contributes to poor survival outcomes [9]. Moreover, lung cancer screening is recommended for high‐risk individuals starting at age 50 [12], with randomized trials indicating its efficacy up to age 77 [12, 13], making it essential to understand lung cancer trends across different age groups for effective detection, prevention, and risk reduction measures. On the whole, the burden of lung cancer is not evenly distributed across populations but influenced by factors such as age at onset, gender, socio‐demographic conditions, and geographic regions. However, most previous studies focused on regional disparities in the burden of all‐age lung cancer [14, 15], and limited research has systematically investigated the characters of lung cancer across different age groups on a global scale, particularly in terms of epidemiology and disease burden. Therefore, it is crucial to understand the distinct trends associated with varying onset periods of lung cancer to shape future global health interventions strategies.

The Global Burden of Diseases, Injuries, and Risk Factors Study (GBD), initiated in 1991, has consistently offered a rigorous empirical evaluation of health trends worldwide over the past three decades [16]. The most recent study of the series, GBD 2021, systematically assessed incidence, mortality, disability‐adjusted life years (DALYs) and risk factors of diseases, including various cancers, providing estimates across a wide range of demographic and geographic parameters (e.g., age, gender, year, socio‐demographic index (SDI), region, country, and territory) [17, 18]. The GBD 2021 provided valuable insights into the epidemiological trend and global burden of lung cancer, which was defined as tracheal, bronchus, and lung cancer in the dataset.

In the current study, we aimed to comprehensively examine the temporal trends in incidence, mortality, and DALYs of EOLC, MOLC, and LOLC at gender and regional levels, from 1990 to 2021, utilizing data from the GBD 2021. We also explored the effect of socio‐demographic development, cross‐country inequalities, risk factors, and conducted decomposition and future forecasting analyses, with the aim to shed light on lung cancer prevention and control across various age ranges.

## 2 Material and Methods

### 2.1 Data Source and Definitions

We retrieved all cross‐sectional data from the Global Health Data Exchange GBD Results Tool (http://ghdx.healthdata.org/gbd‐results‐tool), administered by the Institute for Health Metrics and Evaluation (IHME), which estimated the burden of 288 causes of death, 371 diseases and injuries, and 88 risk factors across 204 countries and territories from 1990 to 2021 [19]. Lung cancer datasets were obtained from administrative data using codes from the International Classification of Diseases, 9th (ICD‐9) and 10th (ICD‐10) revisions, including 162–162.9, 209.21, V10.1‐V10.20, V16.1‐V16.2, V16.4‐V16.40 for ICD‐9, and C33, C34‐C34.92, Z12.2, Z80.1‐Z80.2, Z85.1‐Z85.20 for ICD‐10. In this study, EOLC, MOLC, and LOLC were defined as lung cancer cases diagnosed in individuals aged 15–49 years, 50–69 years, and 70 years or older, respectively. These age ranges were determined based on prior published investigations, taking into account their clinical significance and implications [9, 10].

The incidence, mortality, DALYs, risk factor proportions and 95% uncertainty intervals (UIs) were extracted directly from GBD 2021, with stratification based on age, gender, year, metric (number and rates), 5 SDI levels, 21 GBD categorized regions and 204 countries or territories. All rates were reported per 100,000 population. List of the 21 GBD regions and corresponding countries was presented in Table S1. The 204 countries or territories were classified into five SDI levels (low, low‐middle, middle, high‐middle and high) based on their SDI. The SDI, ranging from 0 to 1, was computed by the GBD to reflect the development status of different regions by incorporating lag‐distributed income per capita, average years of schooling for those aged 15 and above, and the total fertility rate for individuals under 25 [17]. The GBD 2021 examined three groups of risk factors, including behavioral, environmental and occupational, and metabolic factors. In this study, the proportions of DALYs attributed to risk factors of lung cancer were obtained. The detailed methodology used in GBD 2021 has been introduced in previous studies [16, 17, 18].

### 2.2 Statistical Analysis

The incidence, mortality, DALYs rates and their average annual percentage changes (AAPCs) from 1990 to 2021 were used to quantify the epidemic trends of EOLC, MOLC and LOLC. The age‐standardized rate (ASR), comprising age‐standardized incidence rate (ASIR), mortality rate (ASMR), and disability‐adjusted life year rate (ASDR), was calculated using the direct standardization method [10]. The global population, chosen as the standard reference, was detailed in Table S2. Joinpoint regression models with a maximum of 5 joinpoints were employed to examine the temporal trends in ASIR, ASMR and ASDR for EOLC, MOLC and LOLC from 1990 to 2021. The best‐fit models were finally selected, and significance tests for trend changes were conducted using a Monte Carlo Permutation approach [20]. The AAPCs were computed as weighted averages of the annual percent changes (APCs) across different time segments from the joinpoint models, providing a single measure to describe overall trends, even when the trend shifts within the period. To further evaluate the robustness of our findings, we conducted a sensitivity analysis using estimated annual percentage changes (EAPCs), which were visually displayed on global maps. To further evaluate the relationships between EOLC, MOLC, and LOLC burdens and socio‐demographic development, frontier analysis was applied to examine the lowest achievable DALYs, as determined by the SDI [21]. Cross‐country inequalities in lung cancer burden were quantified using the slope index of inequality (SII) and concentration index, two established metrics for measuring absolute and relative gradient inequality, respectively [22]. Decomposition analysis was employed to demonstrate the contributions of aging, population growth, and epidemiological changes in driving changes of lung cancer DALYs between 1990 and 2021 [23]. We conducted a Bayesian age‐period‐cohort (BAPC) model to project the ASIR, ASMR and ASDR of EOLC, MOL and LOLC from 2022 to 2035 [24]. The autoregressive integrated moving average (ARIMA) model was used as sensitivity analysis [25], which could vlidate the findings from the BAPC results.

All data analysis and graphics were conducted in statistical software R (version 4.2.1, R Development Core Team, Austria). Joinpoint software (Windows command‐line version 5.2.0, IMS Inc., America) was called in R for temporal trends assessment. Statistical significance was assessed at the level of *p* < 0.05 (two‐sided).

### 2.3 Ethics and GATHER Statement

Since this study utilized de‐identified, population‐level, publicly available data from the GBD 2021 study, which complies with rigorous ethical standards for data confidentiality, ethical approval and informed consent were not required. The current study adhered to the Guidelines for Accurate and Transparent Health Estimates Reporting (GATHER) [26]. Data were acquired under the provisions of the IHME's Free‐of‐Charge Non‐commercial User Agreement.

## 3 Results

### 3.1 Global Trends of EOLC, MOLC, and LOLC

**FIGURE 1:**
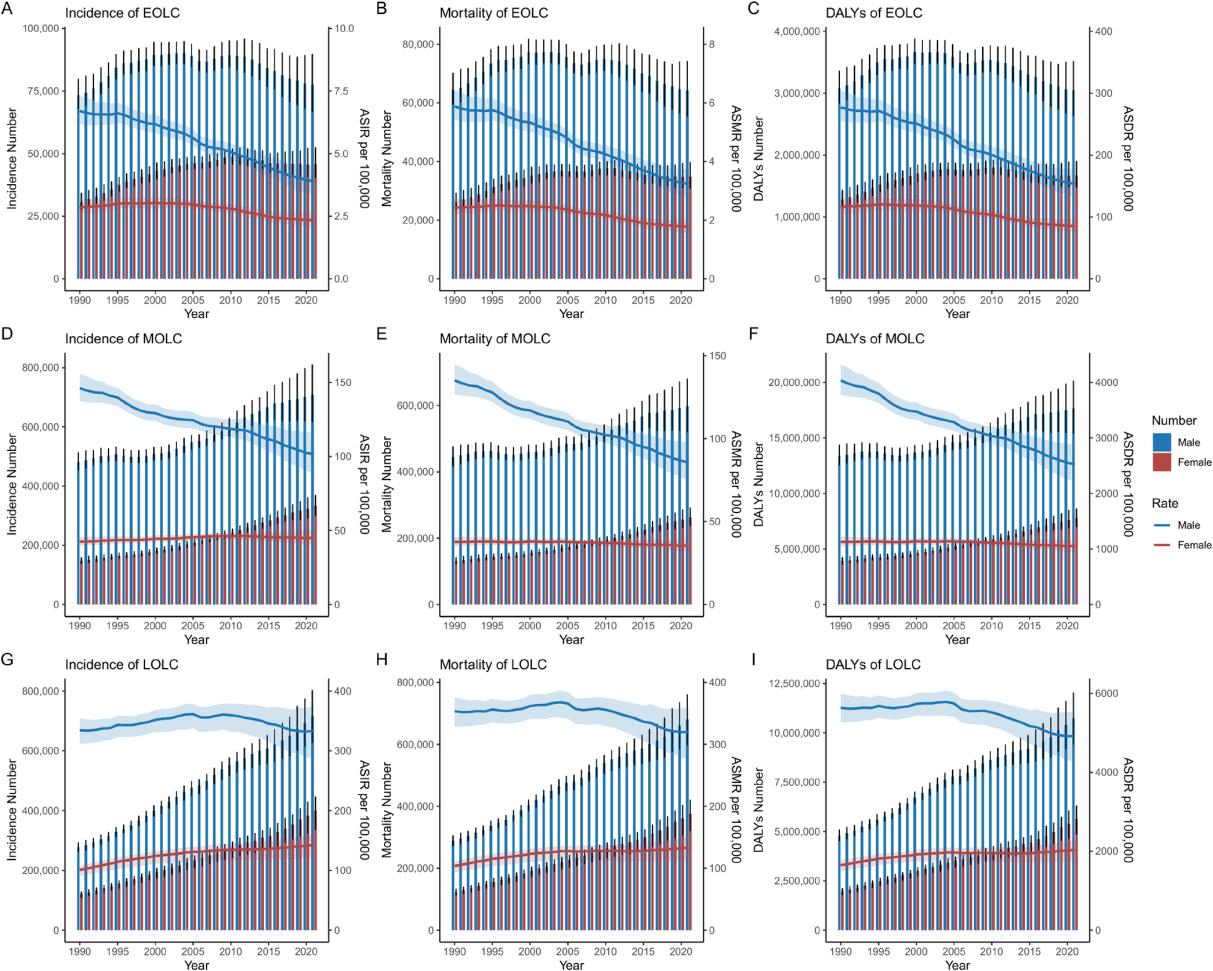
Global trends in incidence, mortality and DALYs of EOLC (A, B, C), MOLC (D, E, F) and LOLC (G, H, I) from 1990 to 2021. DALYs, disability‐adjusted life years; EOLC, early‐onset lung cancer; MOLC, middle‐onset lung cancer; LOLC, late‐onset lung cancer; ASIR, age‐standardized incidence rate; ASMR, age‐standardized mortality rate; ASDR, age‐standardized disability‐adjusted life year rate.

The global ASIR, ASMR and ASDR of all‐age lung cancer declined from 28.54, 27.58 and 690.86 per 100,000 population in 1990 to 26.43, 23.50 and 533.00 in 2021. For EOLC, the global ASIR decreased from 4.81 (95%UI: 4.51 to 5.13) in 1990 to 3.13 (95%UI: 2.78 to 3.48) in 2021, with AAPC of −1.38 (95% confidence interval [CI]: −1.53 to −1.22, *p* < 0.001) (Table 1, Table S3). Rates declined more in males (AAPC = −1.79, 95% CI: −2.01 to −1.57, *p* < 0.001) than in females (AAPC = −0.63, 95% CI: −0.73 to −0.53, *p* < 0.001) (Table 1 and Figure 1A). The Global ASMR of EOLC decreased from 4.18 (95% UI: 3.92 to 4.48) in 1990 to 2.51 (95% UI: 2.23 to 2.79) in 2021 (AAPC = −1.62, 95% CI: −1.75 to −1.49) (Table 1 and Table S3). Both sexes exhibited a significant decline in ASMR over the past 30 years, with a more pronounced reduction observed in males compared to females (AAPC: −1.95 vs. −1.00) (Table 1 and Figure 1B). Similarly, the ASDR also declined from 198.3 (95% UI, 185.28 to 212.38) in 1990 to 119.57 (95% UI, 106.44 to 132.92) in 2021 (AAPC = −1.62, 95% CI: −1.75 to −1.49, *p* < 0.001) (Table 1 and Figure 1C). Globally, the ASIR, ASMR and ASDR of MOLC also decreased from 92.77 (95% UI: 88.1 to 97.48), 85 (95% UI: 80.47 to 89.61) and 2545.79 (95% UI: 2408.07 to 2685.3) in 1990 to 72.47 (95% UI: 65.19 to 80.22), 59.89 (95% UI: 53.88 to 66.2) and 1770.81 (95% UI: 1590.97 to 1958.72) in 2021, with AAPC of −0.81 (95% CI: −0.9 to −0.73, *p* < 0.01), −1.14 (95% CI: −1.22 to −1.06, *p* < 0.01) and − 1.18 (95% CI: −1.27 to −1.1, *p* < 0.01) (Table 1, Table S3). While decreasing trends were observed in incidence, mortality, and DALY rates of MOLC in the overall and male population, the ASIR in females showed a slight but significant increase (AAPC = 0.18, 95% CI: 0.13 to 0.22, *p* < 0.01) (Table 1 and Figure 1D–F). In contrast to EOLC and MOLC, the ASIR, ASMR and ASDR of LOLC demonstrated clear upward trends, increasing from 195.39 (95% UI: 181.97 to 205.24), 204.11 (95% UI: 189.79 to 214.79) and 3290.52 (95% UI: 3077.82 to 3458.64) in 1990 to 225.8 (95% UI: 197.32 to 249.36), 213.83 (95% UI: 186.98 to 236.1) and 3312.58 (95% UI: 2919.75 to 3656.63) in 2021, with AAPC of 0.43 (95% CI: 0.37 to 0.5, *p* < 0.01), 0.12 (95% CI: −0.02 to 0.26, *p* = 0.094) and 0.02 (95% CI: −0.14 to 0.17, *p* = 0.805) (Table 1, Table S3). With sex‐specific classification, the burden of LOLC showed a decreasing trend in males, consistent with the overall pattern, while it increased in females in ASIR (AAPC = 1.13, 95% CI: 1.05 to 1.21, *p* < 0.01), ASMR (AAPC = 0.8, 95% CI: 0.7 to 0.91, *p* < 0.01) and ASDR (AAPC = 0.69, 95% CI 0.63 to 0.74, *p* < 0.01) (Table 1, Figure 1G–I). Detailed global ASIR, ASMR and ASDR of EOLC, MOLC and LOLC in 1990 and 2021 and AAPC from 1990 to 2021 were shown in Table 1 and Table S3.

**TABLE 1:** The incidence, mortality and DALYs of EOLC, MOLC, and LOLC in 2021 and AAPC from 1990 to 2021, by gender, SDI levels, and GBD regions.

Group	Incidence				Mortality				DALYs			
	Cases (95% UI), 2021	ASIR (95% UI), 2021	AAPC (95% CI), 1990–2021	*p*	Cases (95% UI), 2021	ASMR (95% UI), 2021	AAPC (95% CI), 1990–2021	*p*	Cases (95% UI), 2021	ASDR (95% UI), 2021	AAPC (95% CI), 1990–2021	*p*
EOLC												
Global	123,409 (109,786, 137,017)	3.13 (2.78, 3.48)	−1.38 (−1.53, −1.22)	< 0.001	99,134 (88,235, 109,946)	2.51 (2.23, 2.79)	−1.62 (−1.75, −1.49)	< 0.001	4,721,455 (4,203,757, 5,230,900)	119.57 (106.44,132.92)	−1.62 (−1.75, −1.49)	< 0.001
Gender												
Male	77,550 (66,876, 89,638)	3.9 (3.36, 4.51)	−1.79 (−2.01, −1.57)	< 0.001	64,278 (55,476, 74,185)	3.23 (2.78, 3.73)	−1.95 (−2.11, −1.78)	< 0.001	3,050,046 (2,634,184, 3,515,522)	153.38 (131.86,176.61)	−1.87 (−2.04, −1.71)	< 0.001
Female	45,859 (40,442, 52,381)	2.34 (2.06, 2.68)	−0.63 (−0.73, −0.53)	< 0.001	34,856 (30,910, 39,654)	1.78 (1.57, 2.02)	−1 (−1.09, −0.91)	< 0.001	1,671,409 (1,483,165, 1,898,095)	85.3 (75.14,97.21)	−1.01 (−1.1, −0.92)	< 0.001
SDI levels												
Low SDI	4051 (3348, 4926)	1.03 (0.84, 1.26)	−0.06 (−0.17, 0.05)	0.257	3743 (3088, 4554)	0.95 (0.78, 1.17)	−0.09 (−0.2, 0.02)	0.107	182,282 (150,280, 222,326)	45.16 (36.94,55.58)	−0.06 (−0.18, 0.07)	0.367
Low‐middle SDI	15,341 (13,826, 17,181)	1.73 (1.54, 1.96)	0.24 (0.14, 0.34)	< 0.001	13,999 (12,597, 15,692)	1.58 (1.41, 1.79)	0.2 (0.14, 0.26)	< 0.001	680,284 (613,299, 761,260)	75.97 (67.54,86.34)	0.19 (0.09, 0.28)	< 0.001
Middle SDI	46,390 (39,076, 53,448)	3.5 (2.94, 4.06)	−0.64 (−0.8, −0.48)	< 0.001	38,430 (32,389, 44,207)	2.9 (2.43, 3.36)	−0.94 (−1.1, −0.79)	< 0.001	1,836,379 (1,550,959, 2,108,792)	139.25 (116.57,160.94)	−0.96 (−1.11, −0.82)	< 0.001
High‐middle SDI	38,839 (33,225, 45,215)	5.13 (4.38, 5.99)	−1.35 (−1.62, −1.08)	< 0.001	30,419 (25,947, 35,332)	4.02 (3.43, 4.7)	−1.78 (−2.1, −1.46)	< 0.001	1,435,320 (1,223,873, 1,665,825)	191.71 (163.26,224.31)	−1.76 (−2.07, −1.45)	< 0.001
High SDI	18,681 (17,939, 19,412)	3.16 (3.01, 3.31)	−2.4 (−2.52, −2.27)	< 0.001	12,455 (11,933, 12,997)	2.11 (2.01, 2.21)	−2.88 (−3.1, −2.65)	< 0.001	582,990 (558,640, 609,008)	99.36 (94.56,104.38)	−2.85 (−3.07, −2.64)	< 0.001
GBD regions												
High‐income Asia Pacific	2646 (2439, 2877)	2.49 (2.23, 2.79)	−1.62 (−1.99, −1.25)	< 0.001	1468 (1370, 1595)	1.37 (1.26, 1.52)	−2.57 (−2.87, −2.28)	< 0.001	68,626 (64,023, 74,461)	65.65 (60.04, 72.48)	−2.59 (−2.88, −2.3)	< 0.001
Central Asia	1098 (964, 1232)	2.33 (2.04, 2.64)	−3.59 (−3.97, −3.21)	< 0.001	983 (863, 1105)	2.09 (1.83, 2.37)	−3.65 (−4.04, −3.26)	< 0.001	47,692 (42,031, 53,620)	100.59 (88,1, 14.4)	−3.56 (−3.96, −3.16)	< 0.001
Southeast Asia	12,779 (10,416, 15,369)	3.42 (2.74, 4.13)	0.09 (−0.03, 0.21)	0.155	11,412 (9288, 13,723)	3.05 (2.44, 3.7)	−0.04 (−0.16, 0.09)	0.58	547,738 (446,351, 658,192)	146.55 (117.08, 177.7)	−0.02 (−0.15, 0.11)	0.741
East Asia	54,415 (42,955, 66,477)	6.46 (5.12, 7.94)	−0.21 (−0.37, −0.05)	0.009	42,069 (32,981, 51,447)	5 (3.95, 6.16)	−0.73 (−0.89, −0.57)	< 0.001	1,998,988 (1,570,706, 2,440,256)	241.08 (190.72, 96.57)	−0.73 (−0.9, −0.56)	< 0.001
Central Europe	2932 (2638, 3199)	4.25 (3.81, 4.65)	−2.6 (−2.79, −2.4)	< 0.001	2436 (2192, 2653)	3.52 (3.17, 3.85)	−2.83 (−3.01, −2.66)	< 0.001	112,213 (101,015, 122,190)	164.16 (147.52, 179.39)	−2.82 (−2.99, −2.64)	< 0.001
Eastern Europe	4468 (4009, 4985)	3.73 (3.33, 4.19)	−2.88 (−3.5, −2.26)	< 0.001	3584 (3222, 3995)	3 (2.68, 3.36)	−3.05 (−3.72, −2.39)	< 0.001	167,002 (150,230, 185,665)	140.38 (125.78, 156.99)	−3.08 (−3.73, −2.44)	< 0.001
North Africa and Middle East	7118 (6175, 8274)	4.41 (3.73, 5.23)	−1.41 (−1.53, −1.28)	< 0.001	6559 (5680, 7645)	4.07 (3.44, 4.83)	−1.45 (−1.58, −1.32)	< 0.001	316,958 (276,200, 368,208)	195.59 (165.62, 31.55)	−1.42 (−1.54, −1.29)	< 0.001
Australasia	499 (440, 571)	3.1 (2.64, 3.64)	−1.27 (−1.66, −0.89)	< 0.001	301 (268, 337)	1.87 (1.64, 2.12)	−1.89 (−2.22, −1.56)	< 0.001	13,961 (12,431, 15,672)	86.97 (76.11, 99.35)	−1.87 (−2.18, −1.55)	< 0.001
Western Europe	8801 (8251, 9347)	3.78 (3.5, 4.06)	−1.85 (−2.03, −1.67)	< 0.001	5640 (5290, 5979)	2.41 (2.25, 2.58)	−2.49 (−2.55, −2.42)	< 0.001	260,855 (245,005, 276,376)	112.85 (105.38, 120.69)	−2.48 (−2.55, −2.42)	< 0.001
Andean Latin America	594 (452, 750)	1.83 (1.36, 2.37)	−0.98 (−2.06, 0.11)	0.078	518 (392, 655)	1.59 (1.18, 2.06)	−1.17 (−2.23, −0.1)	0.033	25,681 (19,475, 32,349)	78.21 (57.99, 101.2)	−1.17 (−2.22, −0.1)	0.032
Caribbean	654 (553, 764)	2.78 (2.32, 3.31)	−1.18 (−1.49, −0.88)	< 0.001	538 (452, 634)	2.29 (1.9, 2.73)	−1.32 (−1.66, −0.99)	< 0.001	25,534 (21,472, 30,069)	108.54 (90.14, 129.62)	−1.41 (−1.8, −1.01)	< 0.001
High‐income North America	4975 (4817, 5141)	2.74 (2.64, 2.85)	−3.46 (−3.68, −3.24)	< 0.001	3409 (3297, 3518)	1.88 (1.81, 1.95)	−3.79 (−3.97, −3.61)	< 0.001	159,286 (154,166, 164,385)	88.02 (84.86, 91.37)	−3.74 (−3.91, −3.58)	< 0.001
Western Sub‐Saharan Africa	779 (585, 1017)	0.48 (0.36, 0.62)	0.16 (0.06, 0.27)	0.002	718 (541, 945)	0.44 (0.33, 0.57)	0.14 (0.03, 0.24)	0.01	35,277 (26,579, 46,371)	21.07 (15.76, 27.53)	0.16 (0.06, 0.27)	0.002
South Asia	12,786 (10,978, 14,815)	2.87 (2.44, 3.35)	0.51 (0.25, 0.78)	< 0.001	11,603 (9954, 13,512)	2.61 (2.21, 3.05)	0.44 (0.18, 0.71)	0.001	561,271 (483,291, 651,588)	124.82 (106.05, 146.16)	0.39 (0.16, 0.62)	0.001
Oceania	171 (124, 251)	2.83 (1.86, 4.38)	0.15 (0.07, 0.23)	< 0.001	158 (113, 235)	2.61 (1.71, 4.07)	0.14 (0.06, 0.21)	0.001	7801 (5617, 11,553)	126.85 (82.99, 197.2)	0.16 (0.08, 0.23)	< 0.001
Central Sub‐Saharan Africa	682 (464, 1041)	1.47 (0.98, 2.31)	−0.29 (−0.42, −0.17)	< 0.001	631 (427, 970)	1.37 (0.9, 2.14)	−0.31 (−0.44, −0.18)	< 0.001	30,154 (20,415, 46,113)	64.04 (42.03, 100.38)	−0.3 (−0.42, −0.18)	< 0.001
Central Latin America	1862 (1614, 2142)	1.44 (1.25, 1.67)	−1.52 (−1.81, −1.24)	< 0.001	1634 (1413, 1881)	1.27 (1.09, 1.47)	−1.67 (−1.95, −1.38)	< 0.001	80,154 (69,479, 92,246)	61.89 (53.39, 71.86)	−1.64 (−1.93, −1.35)	< 0.001
Southern Latin America	867 (779, 965)	2.44 (2.1, 2.83)	−2.94 (−3.13, −2.76)	< 0.001	730 (658, 809)	2.06 (1.76, 2.39)	−3.17 (−3.36, −2.98)	< 0.001	34,942 (31,485, 38,764)	98.49 (84.37, 114.5)	−3.1 (−3.29, −2.91)	< 0.001
Tropical Latin America	2545 (2418, 2665)	2.04 (1.92, 2.17)	−1.15 (−1.47, −0.84)	< 0.001	2239 (2128, 2344)	1.8 (1.69, 1.91)	−1.28 (−1.58, −0.97)	< 0.001	107,486 (102,187, 112,452)	86.39 (81.25, 91.9)	−1.24 (−1.54, −0.93)	< 0.001
Eastern Sub‐Saharan Africa	1497 (1239, 1913)	1.04 (0.85, 1.33)	−0.63 (−0.72, −0.54)	< 0.001	1381 (1141, 1764)	0.96 (0.78, 1.23)	−0.67 (−0.76, −0.58)	< 0.001	66,766 (55,106, 85,256)	45.36 (36.95, 57.97)	−0.63 (−0.72, −0.54)	< 0.001
Southern Sub‐Saharan Africa	1239 (1077, 1412)	3.32 (2.82, 3.89)	−0.99 (−1.4, −0.58)	< 0.001	1124 (975, 1277)	3.01 (2.55, 3.55)	−1.03 (−1.43, −0.62)	< 0.001	53,071 (46,122, 60,376)	140.75 (119.21, 65.77)	−1.03 (−1.46, −0.59)	< 0.001

MOLC												
Global	1,041,009 (938,152, 1,149,875)	72.47 (65.19, 80.22)	−0.81 (−0.9, −0.73)	< 0.001	860,322 (775,590, 948,271)	59.89 (53.88, 66.2)	−1.14 (−1.22, −1.06)	< 0.001	25,438,474 (22,905,890, 28,055,144)	1770.81 (1590.97, 1958.72)	−1.18 (−1.27, −1.1)	< 0.001
Gender												
Male	708,015 (617,366, 809,766)	101.45 (88.37, 115.93)	−1.19 (−1.31, −1.07)	< 0.001	597,268 (521,019, 680,419)	85.61 (74.62, 97.7)	−1.47 (−1.56, −1.38)	< 0.001	17,652,457 (15,417,161, 20,125,560)	2523.93 (2199.88, 2878.15)	−1.52 (−1.61, −1.43)	< 0.001
Female	332,994 (299,681, 368,594)	45.11 (40.21, 50.49)	0.18 (0.13, 0.22)	< 0.001	263,053 (237,849, 291,235)	35.62 (31.92, 39.78)	−0.19 (−0.3, −0.07)	0.001	7,786,017 (7,020,549, 8,625,757)	1056.89 (946.12, 1181.64)	−0.23 (−0.32, −0.14)	< 0.001
SDI levels												
Low SDI	15,722 (13,164, 18,643)	17.75 (14.84, 21.22)	−0.28 (−0.34, −0.22)	< 0.001	15,721 (13,185, 18,731)	17.81 (14.87, 21.37)	−0.3 (−0.36, −0.24)	< 0.001	479,154 (401,042, 572,641)	533.91 (445.2, 642.02)	−0.29 (−0.35, −0.23)	< 0.001
Low‐middle SDI	73,702 (66,997, 81,566)	29.56 (26.61, 32.85)	0.29 (0.19, 0.38)	< 0.001	72,924 (66,142, 80,795)	29.3 (26.34, 32.61)	0.23 (0.1, 0.35)	< 0.001	2,206,406 (2,000,209, 2,446,837)	879.17 (788.93, 979.52)	0.23 (0.13, 0.32)	< 0.001
Middle SDI	318,220 (266,259, 370,600)	66.82 (55.61, 78.19)	−0.01 (−0.08, 0.06)	0.682	278,618 (233,024, 322,824)	58.57 (48.8, 68.06)	−0.41 (−0.51, −0.32)	< 0.001	8,320,279 (6,955,198, 9,628,079)	1738.67 (1447.32, 020.05)	−0.45 (−0.54, −0.36)	< 0.001
High‐middle SDI	356,525 (311,385, 411,913)	107.43 (93.45, 124.08)	−0.57 (−0.78, −0.36)	< 0.001	292,675 (255,969, 335,452)	88.12 (76.9, 101.73)	−1.02 (−1.24, −0.8)	< 0.001	8,633,069 (7,528,914, 9,895,535)	2613.07 (2276.26, 3019.61)	−1.07 (−1.29, −0.84)	< 0.001
High SDI	275,728 (263,887, 284,984)	93.94 (89.63, 97.73)	−1.39 (−1.49, −1.29)	< 0.001	199,384 (191,729, 205,918)	67.75 (64.91, 70.27)	−1.87 (−1.99, −1.75)	< 0.001	5,770,320 (5,554,937, 5,972,478)	1988.96 (1906.31, 2063.34)	−1.93 (−2.06, −1.79)	< 0.001
GBD regions												
High‐income Asia Pacific	34,403 (31,888, 36,766)	64.51 (58.16, 70.65)	−0.7 (−0.92, −0.49)	< 0.001	21,438 (19,988, 22,751)	40.09 (36.66, 43.48)	−1.41 (−1.54, −1.28)	< 0.001	611,728 (572,226, 649,311)	1161.62 (1064.15, 1261.82)	−1.46 (−1.72, −1.2)	< 0.001
Central Asia	7066 (6220, 7900)	44.81 (39.4, 50.15)	−2.88 (−3.07, −2.7)	< 0.001	6858 (6036, 7655)	43.58 (38.35, 48.74)	−2.92 (−3.11, −2.74)	< 0.001	206,374 (181,204, 231,007)	1299.49 (1141.02, 1454.79)	−3.02 (−3.2, −2.84)	< 0.001
Southeast Asia	74,776 (61,215, 87,300)	61.46 (49.87, 72.64)	−0.05 (−0.13, 0.03)	0.182	72,710 (59,425, 84,987)	59.9 (48.47, 70.81)	−0.16 (−0.24, −0.08)	< 0.001	2,184,416 (1,782,109, 2,564,341)	1782.13 (1441.18, 2109.44)	−0.17 (−0.24, −0.09)	< 0.001
East Asia	440,931 (351,600, 540,297)	112.94 (89.62, 138.7)	0.39 (0.22, 0.57)	< 0.001	354,542 (282,071, 435,278)	90.77 (71.89, 112.1)	−0.25 (−0.46, −0.04)	0.02	10,499,613 (8,332,924, 12,910,358)	2688.24 (2125.49, 3323.66)	−0.3 (−0.51, −0.09)	0.006
Central Europe	43,886 (40,648, 46,874)	133.68 (123.03, 143.64)	−0.34 (−0.47, −0.22)	< 0.001	40,292 (37,364, 42,952)	122.02 (112.45, 130.69)	−0.53 (−0.66, −0.4)	< 0.001	1,159,504 (1,073,841, 1,237,188)	3605.69 (3320.11, 3867.92)	−0.65 (−0.77, −0.53)	< 0.001
Eastern Europe	50,686 (45,474, 55,766)	87.31 (78.22, 96.76)	−1.57 (−2.21, −0.93)	< 0.001	43,169 (38,795, 47,305)	74.16 (66.42, 82)	−1.85 (−2.54, −1.16)	< 0.001	1,266,874 (1,138,890, 1,390,227)	2223.11 (1991.15, 2463.57)	−2.01 (−2.78, −1.24)	< 0.001
North Africa and Middle East	39,415 (33,730, 45,788)	97.24 (82.41, 114.55)	−0.65 (−0.78, −0.51)	< 0.001	39,261 (33,681, 45,740)	97.16 (82.39, 114.44)	−0.7 (−0.82, −0.59)	< 0.001	1,181,332 (1,011,473, 1,374,601)	2880.51 (2440.83, 3390.4)	−0.74 (−0.88, −0.61)	< 0.001
Australasia	6050 (5553, 6524)	80.14 (71.26, 89.09)	−1.4 (−1.62, −1.19)	< 0.001	4085 (3807, 4380)	54.03 (49.13, 59.07)	−2.03 (−2.22, −1.83)	< 0.001	119,188 (111,137, 127,744)	1598.18 (1452.05, 1749.63)	−2.02 (−2.21, −1.81)	< 0.001
Western Europe	121,983 (116,005, 127,017)	101.69 (95.91, 107.08)	−0.82 (−1, −0.64)	< 0.001	88,525 (84,743, 91,887)	73.59 (70.08, 76.91)	−1.41 (−1.49, −1.32)	< 0.001	2,587,885 (2,481,080, 2,687,893)	2173.27 (2069.03, 2272.88)	−1.47 (−1.62, −1.31)	< 0.001
Andean Latin America	2555 (1932, 3239)	26.64 (19.85, 34.86)	−0.86 (−1.55, −0.16)	0.015	2433 (1838, 3077)	25.4 (18.89, 32.94)	−1.04 (−1.72, −0.35)	0.003	72,704 (54,822, 92,424)	754.18 (559.57, 982.9)	−1.03 (−1.72, −0.35)	0.003
Caribbean	5498 (4796, 6255)	63.53 (54.55, 73.25)	0.09 (−0.06, 0.24)	0.267	4756 (4172, 5394)	55.04 (47.44, 62.81)	−0.16 (−0.26, −0.06)	0.001	141,954 (124,395, 161,291)	1631.31 (1405.09, 1864.81)	−0.2 (−0.35, −0.05)	0.009
High‐income North America	99,417 (95,242, 102,718)	98.97 (94.26, 102.74)	−2.31 (−2.47, −2.15)	< 0.001	70,820 (68,010, 73,138)	70.38 (67.28, 72.8)	−2.68 (−2.87, −2.49)	< 0.001	2,049,738 (1,974,275, 2,117,227)	2072.54 (1982.71, 2143.97)	−2.72 (−2.95, −2.5)	< 0.001
Western Sub‐Saharan Africa	4051 (3273, 4913)	11.71 (9.43, 14.34)	0.3 (0.23, 0.38)	< 0.001	4053 (3278, 4939)	11.78 (9.48, 14.51)	0.29 (0.22, 0.36)	< 0.001	122,375 (98,763, 149,717)	346.85 (278.47, 428.17)	0.26 (0.18, 0.33)	< 0.001
South Asia	56,090 (46,810, 64,290)	43.87 (36.33, 50.69)	0.29 (−0.06, 0.63)	0.101	55,186 (46,132, 63,152)	43.21 (35.82, 49.87)	0.22 (−0.13, 0.57)	0.22	1,673,825 (1,394,349, 1,918,014)	1303.21 (1078.96, 1505.85)	0.23 (−0.09, 0.55)	0.163
Oceania	694 (517, 967)	50.38 (36.29, 73.22)	0.06 (−0.07, 0.2)	0.342	692 (513, 978)	50.52 (36.35, 74.1)	0.06 (−0.04, 0.16)	0.265	21,033 (15,510, 29,791)	1495.86 (1070.21, 2200)	0.07 (−0.03, 0.16)	0.15
Central Sub‐Saharan Africa	3179 (2206, 4872)	30.45 (20.78, 46.88)	−0.25 (−0.38, −0.13)	< 0.001	3178 (2204, 4883)	30.61 (20.81, 47.51)	−0.27 (−0.39, −0.14)	< 0.001	97,994 (67,901, 150,590)	920.22 (623.49, 1431.58)	−0.27 (−0.4, −0.14)	< 0.001
Central Latin America	10,915 (9536, 12,587)	26 (22.55, 30.07)	−1.5 (−1.81, −1.2)	< 0.001	10,412 (9035, 11,991)	24.83 (21.48, 28.69)	−1.65 (−1.97, −1.33)	< 0.001	309,881 (268,546, 357,402)	734.53 (633.67, 850.26)	−1.64 (−1.92, −1.36)	< 0.001
Southern Latin America	8309 (7546, 9166)	62.23 (55.85, 69.13)	−1.31 (−1.43, −1.19)	< 0.001	7729 (7021, 8498)	57.82 (52.08, 64.12)	−1.48 (−1.6, −1.35)	< 0.001	225,879 (204,391, 249,307)	1699.56 (1526.14, 1888.61)	−1.6 (−1.71, −1.48)	< 0.001
Tropical Latin America	19,224 (18,253, 20,153)	44.68 (41.83, 47.42)	−0.44 (−0.62, −0.27)	< 0.001	18,422 (17,462, 19,287)	42.85 (40.15, 45.48)	−0.55 (−0.73, −0.38)	< 0.001	547,194 (518,914, 573,395)	1269.41 (1190.41, 1347.46)	−0.58 (−0.81, −0.35)	< 0.001
Eastern Sub‐Saharan Africa	5585 (4797, 6794)	18.87 (15.97, 23.04)	−0.77 (−0.85, −0.7)	< 0.001	5586 (4783, 6803)	18.95 (16.05, 23.19)	−0.8 (−0.87, −0.72)	< 0.001	169,733 (144,937, 207,711)	564.9 (478.28, 694.56)	−0.8 (−0.87, −0.73)	< 0.001
Southern Sub‐Saharan Africa	6297 (5683, 7080)	61.33 (54.13, 69.79)	0.43 (−0.4, 1.27)	0.306	6176 (5572, 6958)	60.25 (53.33, 68.47)	0.39 (−0.44, 1.23)	0.355	189,250 (170,442, 213,400)	1834.28 (1618.86, 2090.72)	0.36 (−0.45, 1.18)	0.388

LOLC												
Global	1,116,271 (992,421, 1,221,803)	225.8 (197.32, 249.36)	0.43 (0.37, 0.5)	< 0.001	1,057,092 (941,474, 1,156,023)	213.83 (186.98, 236.1)	0.12 (−0.02, 0.26)	0.094	16,376,344 (14,665,347, 17,876,317)	3312.58 (2919.75, 3656.63)	0.02 (−0.14, 0.17)	0.805
Gender												
Male	716,416 (629,127, 801,895)	333.52 (288.71, 374.99)	−0.03 (−0.11, 0.05)	0.467	680,995 (596,852, 760,398)	319.91 (276.95, 359.65)	−0.36 (−0.49, −0.23)	< 0.001	10,737,127 (9,422,374, 12,027,868)	4921.27 (4271.89, 5540.33)	−0.49 (−0.63, −0.35)	< 0.001
Female	399,855 (338,328, 445,601)	142.93 (120.27, 160.73)	1.13 (1.05, 1.21)	< 0.001	376,097 (319,862, 420,227)	133.35 (112.45, 149.87)	0.8 (0.7, 0.91)	< 0.001	5,639,217 (4,869,130, 6,294,339)	2039.45 (1747.56, 2282.67)	0.69 (0.63, 0.74)	< 0.001
SDI levels												
Low SDI	8129 (7101, 9338)	37.2 (31.95, 42.98)	0.19 (0.07, 0.31)	0.002	9494 (8280, 10,908)	44.23 (37.94, 51.12)	0.21 (0.09, 0.33)	0.001	159,255 (138,817, 183,305)	706.46 (605.93, 816.7)	0.1 (−0.01, 0.21)	0.069
Low‐middle SDI	41,650 (37,862, 45,662)	59.3 (53.18, 65.64)	0.59 (0.43, 0.75)	< 0.001	48,341 (43,972, 52,950)	69.55 (62.29, 77.04)	0.56 (0.41, 0.71)	< 0.001	799,441 (730,108, 877,294)	1117.46 (1006.14, 1237.21)	0.54 (0.36, 0.72)	< 0.001
Middle SDI	284,117 (235,026, 329,915)	202.49 (166.95, 236.84)	1.23 (1.07, 1.38)	< 0.001	288,053 (239,958, 332,777)	206.9 (171.4, 240.85)	0.77 (0.61, 0.92)	< 0.001	4,581,142 (3,828,644, 5,319,345)	3226.1 (2680.51, 3752.5)	0.68 (0.56, 0.81)	< 0.001
High‐middle SDI	335,820 (290,943, 380,285)	285.97 (243.92, 328.05)	1.14 (1.06, 1.21)	< 0.001	325,523 (283,172, 367,323)	276.8 (237.14, 317.25)	0.66 (0.5, 0.83)	< 0.001	5,124,023 (4,463,389, 5,792,273)	4375.42 (3767.08, 5015.28)	0.54 (0.45, 0.63)	< 0.001
High SDI	445,612 (393,434, 473,335)	306.03 (269.58, 326.3)	0.02 (−0.14, 0.18)	0.843	384,689 (339,216, 409,333)	259.4 (229.16, 275.78)	−0.4 (−0.49, −0.32)	< 0.001	5,696,677 (5,110,732, 6,023,870)	4002.54 (3594.21, 4228.46)	−0.6 (−0.69, −0.52)	< 0.001
GBD regions												
High‐income Asia Pacific	117,576 (98,938, 128,056)	317.13 (267.94, 350.79)	0.43 (0.15, 0.72)	0.003	93,282 (77,730, 102,070)	242.17 (206.22, 64.06)	−0.07 (−0.13, −0.02)	0.005	1,279,010 (1,094,057, 1,381,446)	3576.2 (3102.55, 3860.86)	−0.24 (−0.29, −0.19)	< 0.001
Central Asia	2788 (2527, 3048)	81.7 (73.4, 90.18)	−1.08 (−1.44, −0.73)	< 0.001	3143 (2847, 3436)	92.08 (82.72, 101.29)	−1.13 (−1.48, −0.77)	< 0.001	53,205 (48,384, 58,093)	1559.93 (1405.12, 1713.16)	−1.12 (−1.54, −0.7)	< 0.001
Southeast Asia	44,184 (36,175, 50,634)	147.58 (119.07, 173.74)	0.61 (0.52, 0.69)	< 0.001	50,827 (41,621, 58,307)	171.27 (138.27, 201.64)	0.54 (0.46, 0.63)	< 0.001	824,208 (674,216, 950,454)	2706.94 (2195.18, 3176.79)	0.46 (0.38, 0.54)	< 0.001
East Asia	459,568 (375,795, 547,079)	375.9 (304.78, 450.61)	1.6 (1.31, 1.89)	< 0.001	437,495 (357,811, 519,849)	362.08 (293.93, 434.62)	1.03 (0.76, 1.3)	< 0.001	6,894,183 (5,628,962, 8,225,716)	5556.08 (4504.94, 6691.09)	0.96 (0.71, 1.21)	< 0.001
Central Europe	35,296 (32,531, 37,607)	239.37 (218.76, 256.59)	0.92 (0.86, 0.98)	< 0.001	37,934 (35,046, 40,424)	256.24 (234.91, 274.13)	0.74 (0.68, 0.8)	< 0.001	618,669 (572,364, 658,396)	4228.16 (3894.38, 4515.29)	0.7 (0.64, 0.76)	< 0.001
Eastern Europe	28,847 (26,554, 31,065)	136.74 (124.54, 148.13)	−0.4 (−0.92, 0.13)	0.136	28,214 (26,039, 30,367)	133.12 (121.26, 144.09)	−0.68 (−1.21, −0.15)	0.012	469,430 (433,950, 505,524)	2234.56 (2038.72, 2420.35)	−0.75 (−1.23, −0.26)	0.002
North Africa and Middle East	24,642 (21,388, 28,683)	243.28 (204.61, 288.56)	0.3 (0.16, 0.43)	< 0.001	28,744 (24,980, 33,497)	286.17 (240.95, 339.51)	0.26 (0.13, 0.39)	< 0.001	464,705 (405,528, 543,275)	4516.13 (3825.54, 5345.97)	0.2 (−0.01, 0.42)	0.059
Australasia	9829 (8401, 11,014)	267.3 (224.7, 309.72)	−0.05 (−0.39, 0.29)	0.776	7874 (6792, 8837)	211.35 (179.28, 242.38)	−0.65 (−0.8, −0.5)	< 0.001	118,148 (103,132, 130,977)	3261.05 (2799.25, 3693.67)	−0.84 (−0.99, −0.7)	< 0.001
Western Europe	165,032 (145,759, 176,719)	250.73 (221.13, 268.79)	−0.01 (−0.1, 0.09)	0.862	153,576 (134,653, 164,332)	227.02 (200.82, 42.81)	−0.43 (−0.55, −0.3)	< 0.001	2,289,419 (2,044,857, 2,430,131)	3581.91 (3206.74, 3804.97)	−0.56 (−0.69, −0.43)	< 0.001
Andean Latin America	2898 (2293, 3525)	87.76 (67.53, 110.37)	−0.28 (−1.12, 0.56)	0.513	3362 (2657, 4087)	101.41 (77.61, 127.62)	−0.37 (−1.12, 0.38)	0.327	50,920 (39,866, 62,146)	1552.8 (1186.35, 1958.14)	−0.45 (−1.3, 0.41)	0.304
Caribbean	5267 (4669, 5932)	163.91 (139.92, 187.67)	−0.15 (−0.32, 0.02)	0.08	5398 (4794, 6053)	166.44 (142.94, 190.84)	−0.43 (−0.59, −0.27)	< 0.001	82,547 (73,314, 92,392)	2599.88 (2244.14, 2968.21)	−0.37 (−0.52, −0.21)	< 0.001
High‐income North America	147,133 (130,109, 156,621)	339.08 (298.37, 362.25)	−0.47 (−0.63, −0.31)	< 0.001	123,087 (108,931, 131,213)	280.94 (246.66, 299.67)	−0.76 (−0.94, −0.57)	< 0.001	1,868,265 (1,673,802, 1,979,130)	4337.95 (3871.17, 4601.14)	−0.97 (−1.15, −0.78)	< 0.001
Western Sub‐Saharan Africa	2495 (2157, 2888)	30.46 (25.63, 35.76)	0.82 (0.72, 0.92)	< 0.001	2919 (2531, 3373)	36.06 (30.44, 42.36)	0.81 (0.72, 0.91)	< 0.001	48,445 (41,433, 56,371)	578.87 (485.16, 681.87)	0.77 (0.63, 0.92)	< 0.001
South Asia	28,982 (24,504, 32,869)	78.74 (66.1, 89.99)	0.63 (0.33, 0.94)	< 0.001	33,392 (28,374, 37,815)	91.82 (77.39, 105)	0.6 (0.37, 0.82)	< 0.001	559,200 (473,719, 633,818)	1488.48 (1255.99, 1702.09)	0.54 (0.25, 0.84)	< 0.001
Oceania	365 (271, 520)	130.91 (94.33, 191.28)	0.26 (0.18, 0.35)	< 0.001	423 (313, 611)	154.55 (111.08, 226.92)	0.25 (0.17, 0.33)	< 0.001	7182 (5281, 10,345)	2494.16 (1792.49, 3665.12)	0.23 (0.14, 0.33)	< 0.001
Central Sub‐Saharan Africa	1093 (810, 1593)	56.93 (40.42, 84.71)	−0.17 (−0.29, −0.06)	0.004	1269 (932, 1848)	67.26 (47.41, 100.89)	−0.18 (−0.3, −0.06)	0.004	21,820 (16,078, 31,693)	1102.07 (783.53, 1638.47)	−0.21 (−0.31, −0.1)	< 0.001
Central Latin America	10,704 (9432, 11,941)	78.37 (68.34, 88.28)	−1.13 (−1.36, −0.9)	< 0.001	12,304 (10,845, 13,749)	89.84 (78.36, 101.18)	−1.24 (−1.44, −1.04)	< 0.001	190,642 (169,351, 213,583)	1400.88 (1227.49, 1578.1)	−1.26 (−1.51, −1.01)	< 0.001
Southern Latin America	7929 (7092, 8728)	144.36 (126.67, 160.96)	−0.38 (−0.6, −0.15)	0.001	8855 (7973, 9750)	160.08 (141.16, 178.34)	−0.52 (−0.74, −0.3)	< 0.001	138,868 (125,373, 152,559)	2560.22 (2279.01, 2835.21)	−0.56 (−0.76, −0.36)	< 0.001
Tropical Latin America	15,699 (14,008, 16,791)	109.37 (97.03, 118.1)	0.24 (0.05, 0.43)	0.013	18,066 (16,054, 19,327)	125.56 (111.22, 135.79)	0.16 (−0.03, 0.35)	0.101	283,241 (255,772, 301,488)	1978.73 (1778.86, 2130.49)	0.1 (−0.08, 0.28)	0.276
Eastern Sub‐Saharan Africa	2868 (2488, 3298)	42.15 (35.74, 49.41)	0.12 (0.05, 0.2)	0.001	3361 (2919, 3882)	50.26 (42.63, 59.09)	0.17 (0.1, 0.24)	< 0.001	55,881 (48,660, 64,832)	796.11 (680.31, 933.34)	−0.03 (−0.09, 0.04)	0.423
Southern Sub‐Saharan Africa	3076 (2778, 3401)	115.73 (101.3, 130.77)	0.64 (0.21, 1.07)	0.003	3565 (3230, 3933)	135.77 (118.99, 153.44)	0.6 (0.17, 1.03)	0.006	58,357 (52,700, 64,252)	2149.3 (1891.26, 2422.98)	0.61 (0.16, 1.06)	0.007

*Note:* ASIR, ASMR and ASDR are reported per 100,000 population.

Abbreviations: AAPC, average annual percentage change; ASDR, age‐standardized disability‐adjusted life year rate; ASIR, age‐standardized incidence rate; ASMR, age‐standardized mortality rate; CI, confidence interval; DALYs, disability‐adjusted life‐years; EOLC, early‐onset lung cancer; GBD, global burden of disease; LOLC, late‐onset lung cancer; MOLC, middle‐onset lung cancer; SDI, socio‐demographic index; UI, uncertainty interval.

**FIGURE 2:**
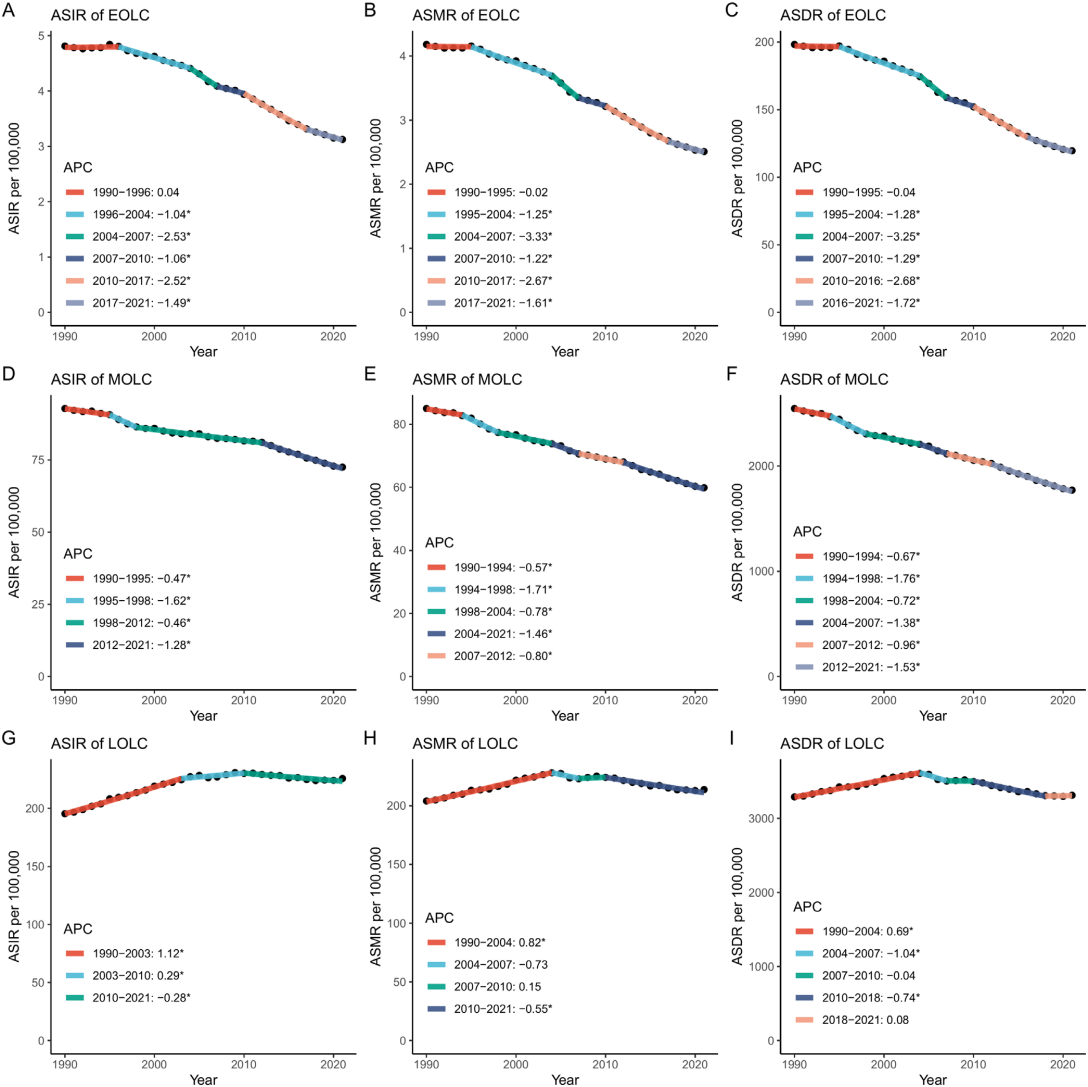
Joinpoint regression analysis of global incidence, mortality and burden for EOLC (A, B, C), MOLC (D, E, F) and LOLC (G, H, I) from 1990 to 2021. EOLC, early‐onset lung cancer; MOLC, middle‐onset lung cancer; LOLC, late‐onset lung cancer; ASIR, age‐standardized incidence rate; ASMR, age‐standardized mortality rate; ASDR, age‐standardized disability‐adjusted life year rate; APC annual percentage change; * with significance, *p* < 0.05.

Joinpoint regression detected notable changes in the incidence, mortality, and DALY trends for EOLC, MOLC, and LOLC from 1990 to 2021, by gender (Figure 2, Figures S1 and S2). In general, the global burden of EOLC and MOLC has declined at different rates over the time periods identified by joinpoint analysis (Figure 2A–F), whereas LOLC has exhibited an upward trend. Notably, significant increases in ASIR of LOLC were observed during two periods: from 1990 to 2003 (APC = 1.12, 95% CI: 1.07 to 1.18, *p* < 0.001) and from 2003 to 2010 (APC = 0.29, 95% CI: 0.08 to 0.49, *p* = 0.008), while a slight decline was observed from 2010 to 2021 (APC = −0.28; 95% CI −0.39 to −0.17, *p* < 0.001) (Figure 2G). Substantial changes in ASMR of LOLC were noted in 2004, 2007, and 2010 (1990–2004 [APC = 0.82, 95% CI: 0.78 to 0.87, *p* < 0.001], 2004‐2007[APC = −0.73, 95% CI: −1.74 to 0.29, *p* = 0.15], 2007–2010[APC = 0.15, 95% CI: −0.92 to 1.22, *p* = 0.777], 2010–2021[APC = −0.55, 95% CI: −0.64 to −0.45, *p* < 0.001]) (Figure 2H). For ASDR of LOLC, significant increase was observed from 1990 to 2004 (APC = 0.69, 95% CI: 0.64 to 0.73), and there was a consistent decrease from 2004 to 2018. A minor increase has been noted since 2018; however, it did not achieve statistical significance (Figure 2I). Importantly, a more detailed joinpoint analysis by gender (Figures S1 and S2) revealed a consistent rise in ASIR of LOLC among females since 1990 (1990–1995 [APC = 2.60, 95% CI: 2.46 to 2.75, *p* < 0.001], 1995–2000 [APC =1.64, 95% CI: 1.43 to 1.84, *p* < 0.001], 2000–2004 [APC = 1.11, 95% CI: 0.77 to 1.44, *p* < 0.001], 2004–2009 [APC = 0.68, 95% CI: 0.46 to 0.90, *p* < 0.001], 2009–2015 [APC = 0.21, 95% CI: 0.04 to 0.39, *p* = 0.02], 2015–2021 [APC = 0.80, 95% CI: 0.64 to 0.97, *p* < 0.001]) (Figure S2G).

### 3.2 Trends of EOLC, MOLC, and LOLC by Regions and Nations

**FIGURE 3:**
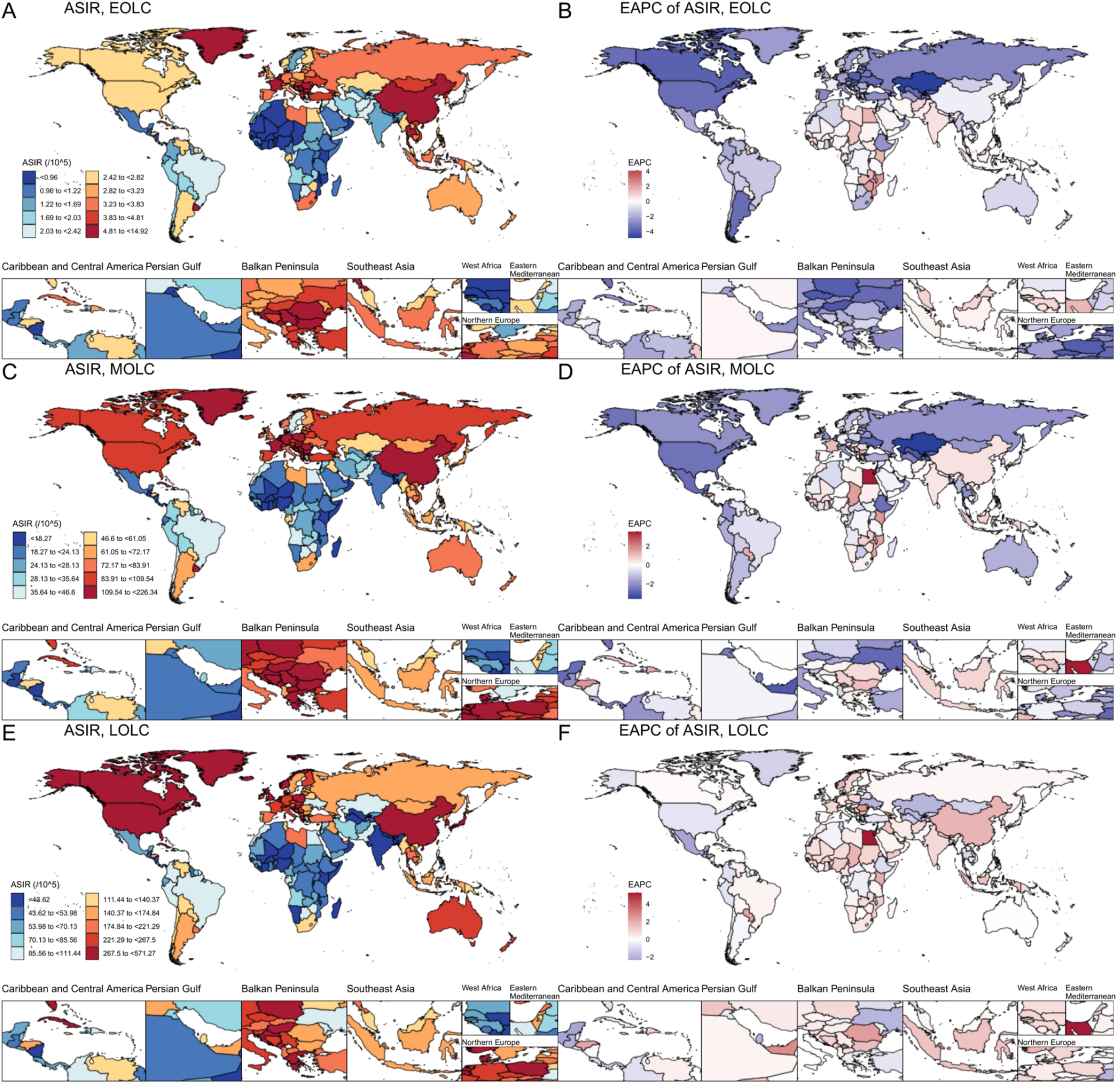
Among 204 countries and territories, the ASIR of EOLC (A), MOLC (C) and LOLC (E) in 2021, and the EAPC of ASIR for EOLC (B), MOLC (D) and LOLC (F) from 1990 to 2021. ASIR, age‐standardized incidence rate; EOLC, early‐onset lung cancer; MOLC, middle‐onset lung cancer; LOLC, late‐onset lung cancer; EAPC, estimated annual percent changes.

The global distribution of ASIR for EOLC, MOLC and LOLC in 2021, along with the EAPC for ASIR from 1990 to 2021 were shown in Figure 3 and Table S4. The incidence, mortality and DALYs of EOLC, MOLC and LOLC in 2021 and AAPC from 1990 to 2021 among 204 countries and territories were presented in Tables S5–S7.

According to the GBD region classification (Table S1), Central Asia experienced the most pronounced reduction of ASIR for EOLC, from 7.11 (95% UI: 6.53 to 7.65) per 100,000 population in 1990 to 2.33 (95% UI: 2.04 to 2.64) in 2021, with AAPC of −3.59 (95% CI: −3.97 to −3.21). However, the Southeast Asia (AAPC = 0.09, 95% CI: −0.03 to 0.21, *p* = 0.155), Western Sub‐Saharan Africa (AAPC = 0.16, CI: 0.06 to 0.27, *p* = 0.002), South Asia (AAPC = 0.51, CI: 0.25 to 0.78, *p* < 0.001) and Oceania (AAPC = 0.15, CI: 0.07 to 0.23, *p* < 0.001) regions showed increasing trends in incidence. Mortality and DALY rates of EOLC increased in the Western Sub‐Saharan Africa, South Asia and Oceania, and decreased in the other 18 GBD regions (Table 1 and Table S3). Similar to EOLC, the ASIR, ASMR, and ASDR for MOLC exhibited increasing trends in Western Sub‐Saharan Africa, South Asia, Oceania and Southern Sub‐Saharan Africa, while the ASIR also exhibited a rising trend in the East Asia and Caribbean (Table 1 and Table S3). In contrast to EOLC and MOLC, 11, 10 and 9 out of 21 GBD regions showed a rising trend for ASIR, ASMR and ASDR of LOLC, respectively. East Asia showed the steepest increase in ASIR (from 229.26 in 1990 to 375.90 in 2021, AAPC = 1.6, 95% CI: 1.31 to 1.89, *p* < 0.001), ASMR (from 266.21 in 1990 to 362.08 in 2021, AAPC = 1.03, 95% CI: 0.76 to 1.3, *p* < 0.001), and ASDR (from 4180.79 in 1990 to 5556.08 in 2021, AAPC = 0.96, 95% CI: 0.71 to 1.21, *p* < 0.001) for LOLC (Table 1 and Table S3).

When different individual nations were examined, Lesotho (AAPC = 3.58, 95% CI: 3.30 to 3.85, *p* < 0.001) and Zimbabwe (AAPC = 1.84, 95% CI: 1.30 to 2.38, *p* < 0.001) demonstrated the most substantial increases in the ASIR of EOLC, while Kazakhstan experienced the steepest decline (AAPC = −4.45, 95% CI: −4.88 to −4.02, *p* < 0.001) (Table S5). At the same time, Lesotho also exhibited the largest increase in ASMR (AAPC = 3.57, 95% CI: 3.29 to 3.86, *p* < 0.001) and ASDR (AAPC = 3.57, 95% CI: 3.29 to 3.85, *p* < 0.001) of EOLC (Tables S6 and S7). As for LOLC, Egypt and Lesotho were found to have the most increases in the ASIR (AAPC = 2.60 [95% CI: 1.98 to 3.21, *p* < 0.001] and AAPC = 2.50 [95% CI: 2.28 to 2.71, *p* < 0.001], respectively), ASMR (AAPC = 2.58 [95% CI: 1.97 to 3.19, *p* < 0.001] and AAPC = 2.48 [95% CI: 2.26 to 2.70, *p* < 0.001], respectively) and ASDR (AAPC = 2.55 [95% CI: 2.34 to 2.77, *p* < 0.001] and AAPC = 2.52 [95% CI: 1.86 to 3.18, *p* < 0.001], respectively) from 1990 to 2021 (Tables S5–S7). In terms of LOLC, Egypt observed the largest increase in the ASIR (AAPC = 4.01, 95% CI: 3.54 to 4.49, *p* < 0.001), ASMR (AAPC = 3.98, 95% CI: 3.51 to 4.46, *p* < 0.001) and ASDR (AAPC = 3.94, 95% CI: 3.41 to 4.48, *p* < 0.001) (Tables S5–S7). Among the three most populous countries (China, India and the United States of America), the incidence, mortality and DALY rates of LOLC showed a decline in the United States of America, contrasting with increases observed in China and India.

In the sensitivity analysis of EAPC, the top five countries or territories exhibiting significant increasing trends in ASIR, ASMR and ASDR of EOLC, MOLC and LOLC demonstrated results that were largely in agreement with the AAPC findings (Tables S4–S7). More detailed geo‐visualization for ASIR, ASMR, and ASDR of lung cancer in 2021 and EAPC from 1990 to 2021, by gender, were presented in Figures S3–S10.

### 3.3 Trends of EOLC, MOLC, and LOLC According to SDI

According to the SDI quintiles, the low‐middle SDI quintile was the only region that demonstrated a significant upward trend in the ASIR, both for EOLC (from 1.62 in 1990 to 1.73 in 2021, AAPC = 0.24 [95% CI: 0.14 to 0.34, *p* < 0.001]) and MOLC (from 27.26 in 1990 to 29.56 in 2021, AAPC = 0.29 [95% CI: 0.19 to 0.38, *p* < 0.001]). This trend was also evident for the ASMR and ASDR of EOLC and MOLC (Table 1 and Table S3). In comparison with EOLC and MOLC, except for the high SDI region, significant increases in ASIR, ASMR and ASDR were observed in the other four SDI regions for LOLC. The middle SDI region demonstrated the largest rise in ASIR (AAPC = 1.23, 95% CI: 1.07 to1.38, *p* < 0.001), ASMR (AAPC = 0.77, 95% CI: 0.61 to 0.92, *p* < 0.001) and ASDR (AAPC = 0.68, 95% CI: 0.56 to 0.81, *p* < 0.001) for LOLC (Table 1 and Table S3).

When the association between SDI and burden of lung cancer was examined at the global and GBD regional level, the overall trend indicated that the disease burden gradually increased with the SDI, but tended to plateau or decrease at higher levels (Figures S11–S13). Regarding EOLC and MOLC, the ASIR, ASMR and ASDR gradually increased with SDI, reaching peak values between 0.7 and 0.8, followed by a marked decrease (Figure S11). However, the downward trend observed in EOLC and MOLC was only marginally noticeable in LOLC (Figure S11). With stratification of gender, steeper rise trends were observed in the ASIR, ASMR and ASDR of LOLC in females, compared to males (Figures S12 and S13). At the national level, the socio‐demographic trends were similar to the changes observed at the GBD regional level (Figures S14 and S16).

**FIGURE 4:**
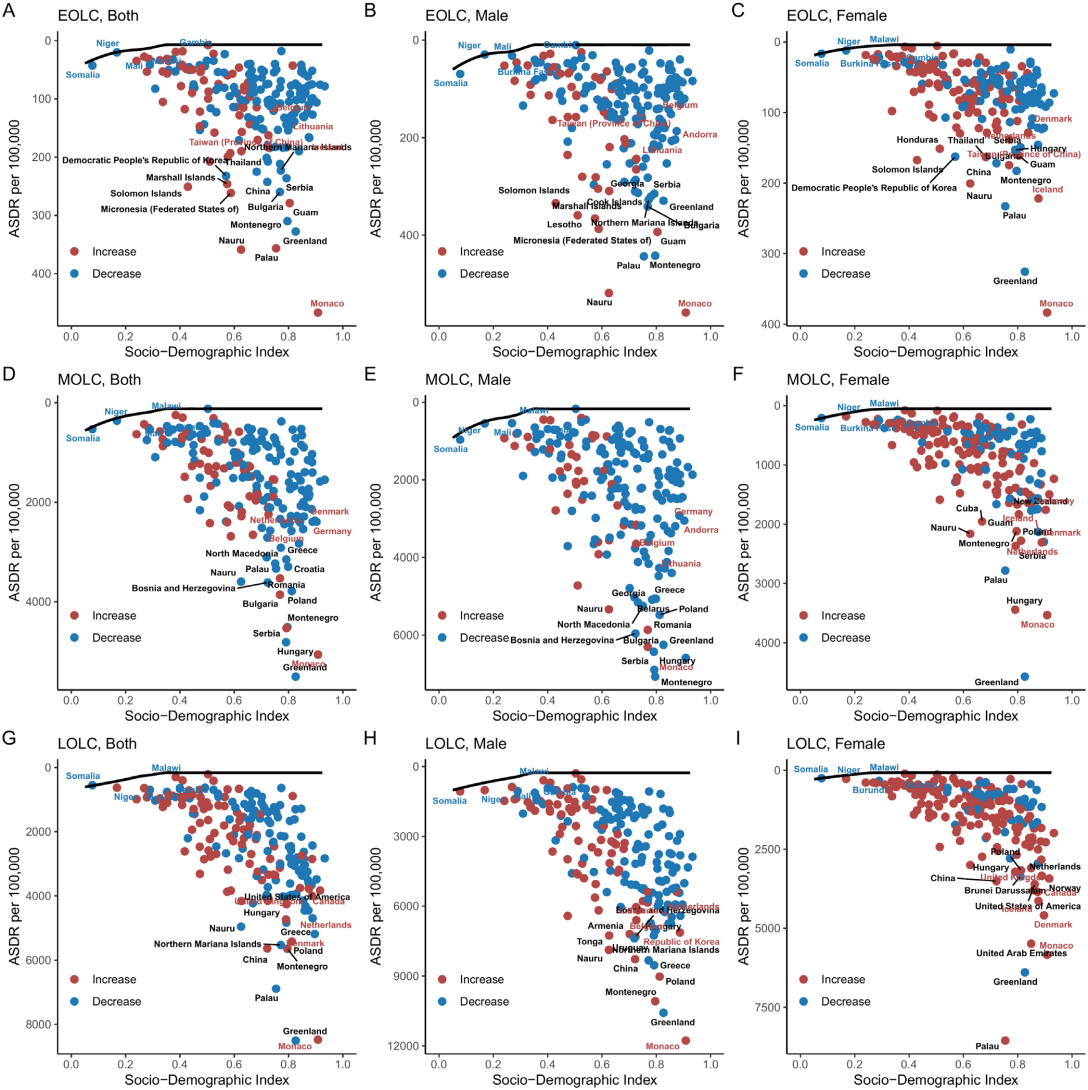
Frontier analysis based on SDI and ASDR in 2021. The frontier is depicted as a solid black line, with countries and territories represented by dots. The top 15 countries showing the largest effective difference (i.e., the greatest gap in ASDR from the frontier) are labeled in black. Examples of frontier countries with low SDI (< 0.5) and small effective differences are labeled in blue, while examples of countries and territories with high SDI (> 0.85) but relatively large effective differences for their development level are labeled in red. Red dots indicate an increase in ASDR from 1990 to 2021, while blue dots indicate a decrease in ASDR during the same period. SDI: Socio‐demographic index; ASDR, age‐standardized disability‐adjusted life year rate; EOLC, early‐onset lung cancer; MOLC, middle‐onset lung cancer; LOLC, late‐onset lung cancer.

The age‐standardized DALYs burden and the effective difference in countries or territories with different SDI levels in 2021 were shown in Figure 4 and Tables S8–S10. The lowest achievable DALYs and unrealized health gains of countries or territories across different stages of socio‐demographic development during from 1990 to 2021 were shown in Figure S17. As shown in Figure 4, the effective difference generally increased in response to advancements in socio‐demographic development, suggesting that countries or territories with a higher SDI had a greater capacity for improving lung cancer burdens.

### 3.4 Cross‐Country Inequalities

**FIGURE 5:**
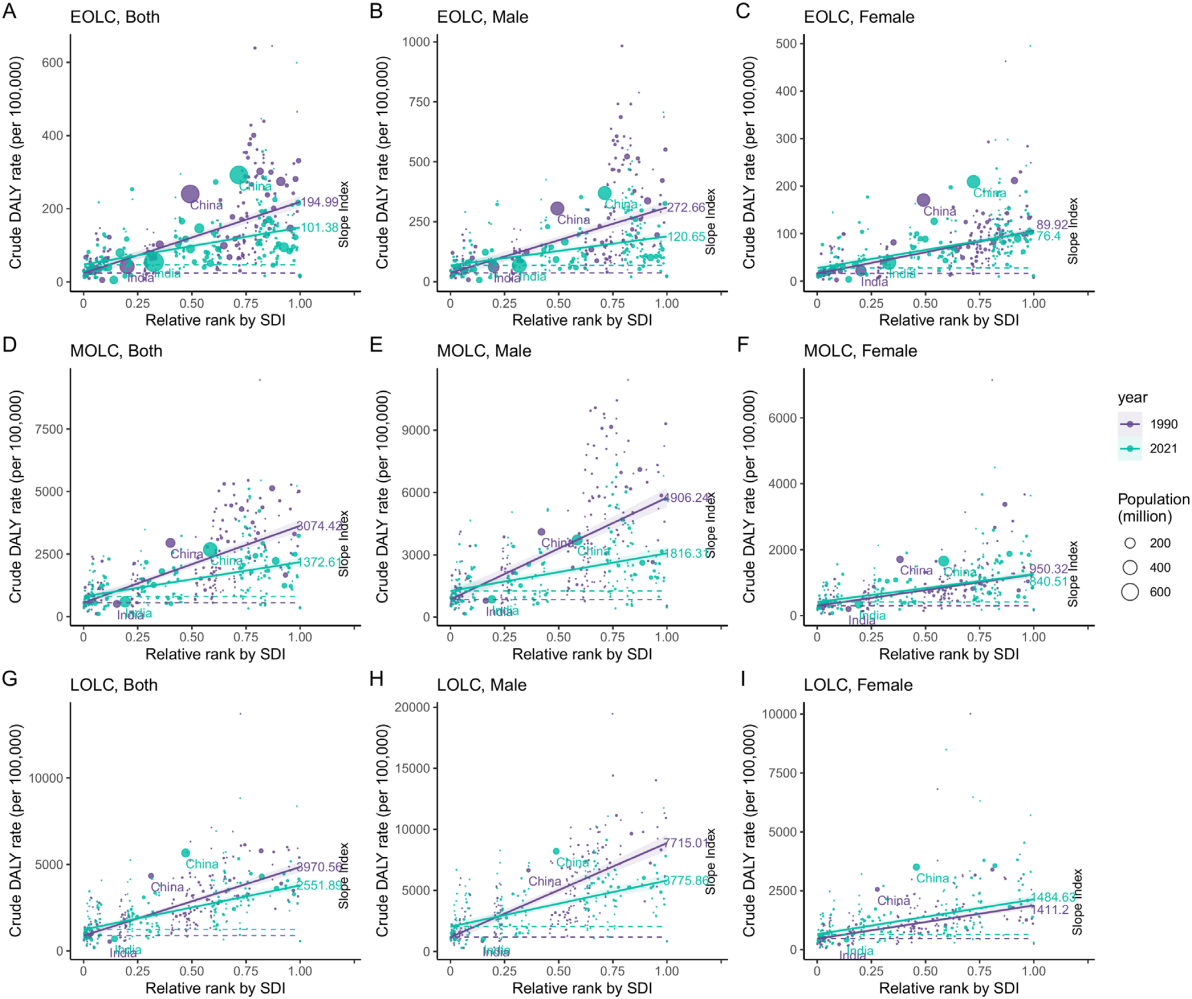
Health inequality regression curves for the DALYs of EOLC (A–C), MOLC (D–F) and LOLC (G–I) worldwide, in 1990 and 2021. The SII values, i.e., the slopes of the regression curves, are labeled next to the corresponding curves. DALYs, disability‐adjusted life‐years; EOLC, early‐onset lung cancer; MOLC, middle‐onset lung cancer; LOLC, late‐onset lung cancer; SII, slope index of inequality; SDI: Socio‐demographic index.

The global absolute and relative inequalities in lung cancer among different age groups, as measures by SII and concentration index, demonstrated a general decreasing trend from 1990 to 2021 (Figure 5, Figures S18 and S19, Tables S11 and S12). The SII, which indicated the gap in crude DALY rates between countries of the highest and lowest SDI levels, declined from 194.99 (95% CI: 160.37 to 229.61) in 1990 to 101.38 (95% CI: 78.78 to 123.98) in 2021 for EOLC, from 3074.42 (95% CI: 2608.76 to 3540.08) in 1990 to 1372.61 (95% CI: 1072.71 to 1672.51) in 2021 for MOLC, and from 3970.56 (95% CI: 3402.13 to 4539) in 1990 to 2551.89 (95% CI: 2109.86 to 2993.91) in 2021 for LOLC (Figure 5 and Table S11). Moreover, the relative inequalities consistent trends for EOLC, MOLC and LOLC, with absolute values of concentration index declined from 1990 to 2021 (Figure S19 and Table S12). However, when compared to males, almost no improvement of cross‐country inequalities has been achieved in females, regardless of age groups. Especially for LOLC, a slight increase was observed in SII from 1411.25 (95% CI: 1169.29 to 1653.2) in 1990 to 1484.63 (95% CI: 1194.78 to 1774.47) in 2021 in females (Figure 5 and Table S11).

### 3.5 Decomposition Analysis

**FIGURE 6:**
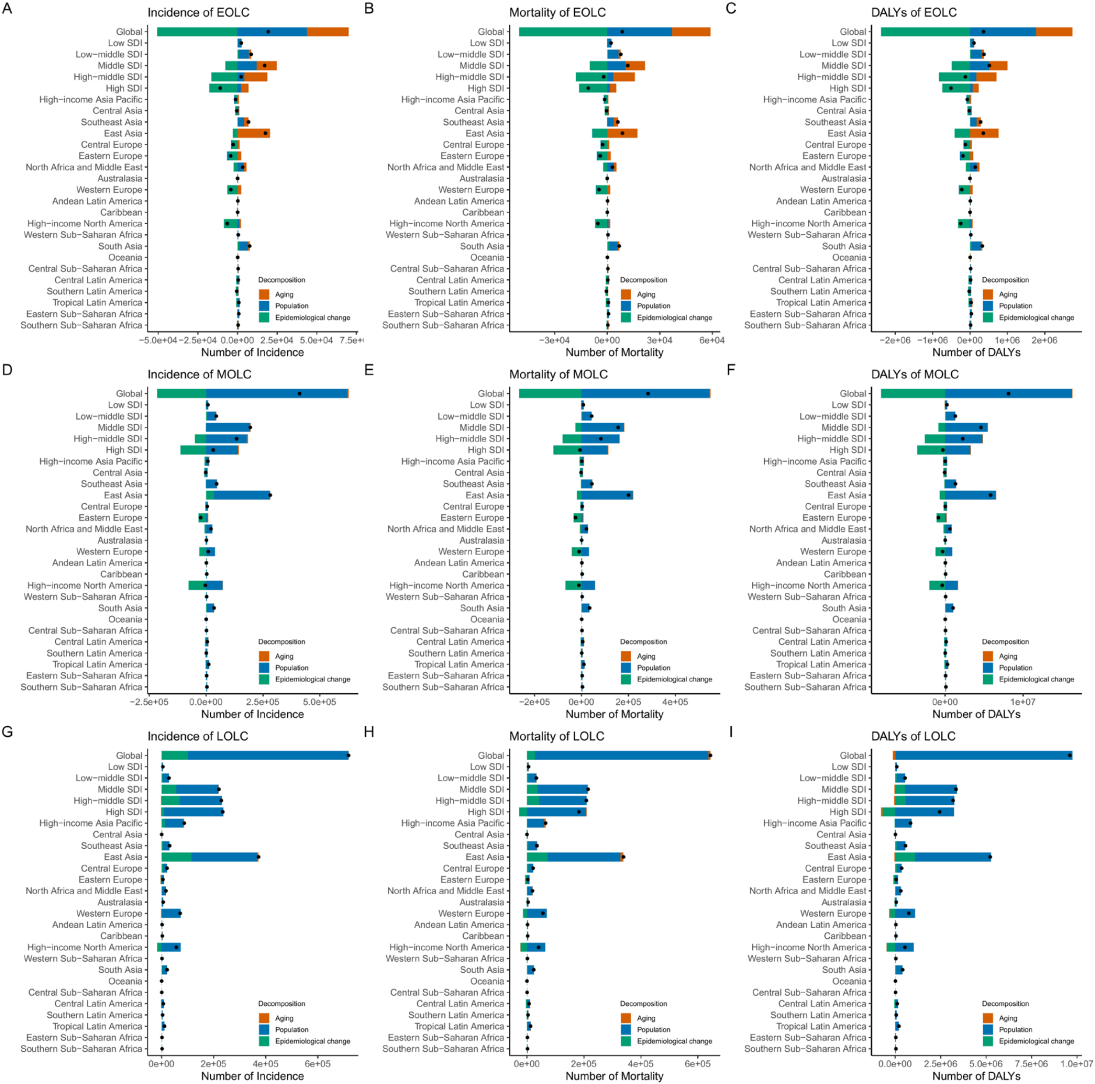
Changes in incidence, mortality, and DALYs for EOLC (A–C), MOLC (D–F), and LOLC (G–I) attributed to aging, population growth, and epidemiological changes at the global, SDI quintile, and regional levels from 1990 to 2021. Black dots represent the cumulative contribution of all three factors to the observed changes. A positive value for any component reflects its contribution to an increase in lung cancer incidence, mortality, and DALYs, while a negative value indicates a reduction in these measures. DALYs, disability‐adjusted life‐years; EOLC, early‐onset lung cancer; MOLC, middle‐onset lung cancer; LOLC, late‐onset lung cancer; SDI: Socio‐demographic index.

Over the past three decades, considerable global increases have been observed in the number of incidence, mortality and DALYs, especially for MOLC and LOLC (Figure 6). In terms of global DALYs, aging, population growth and epidemiological changes accounted for 270.97%, 491.42%, and −662.4% of the effective difference for EOLC, with the largest increase occurring in the middle SDI region among SDI quintiles and East Asia among GBD regions (Figure 6C, Table S13). Population growth and epidemiological changes contributed to 200.42% and −101.30% of the DALYs burden changes for MOLC, while population growth was observed to play an essential role in driving changes for LOLC (Figure 6F,I and Table S13). The attribution characters among the incidence (Figure 6A,D,G) and mortality (Figure 6B,E,H) were similar to those observed in global DALYs. Notably, the increased magnitude of epidemiological changes was greater among females compared to males (Figures S20 and S21). The impact of demographics and epidemiology on changes in incidence, mortality, and DALYs differed across SDI levels and regions (Figure 6, Figures S20 and S21, Tables S14 and S15).

### 3.6 Risk Factors for EOLC, MOLC, and LOLC

**FIGURE 7:**
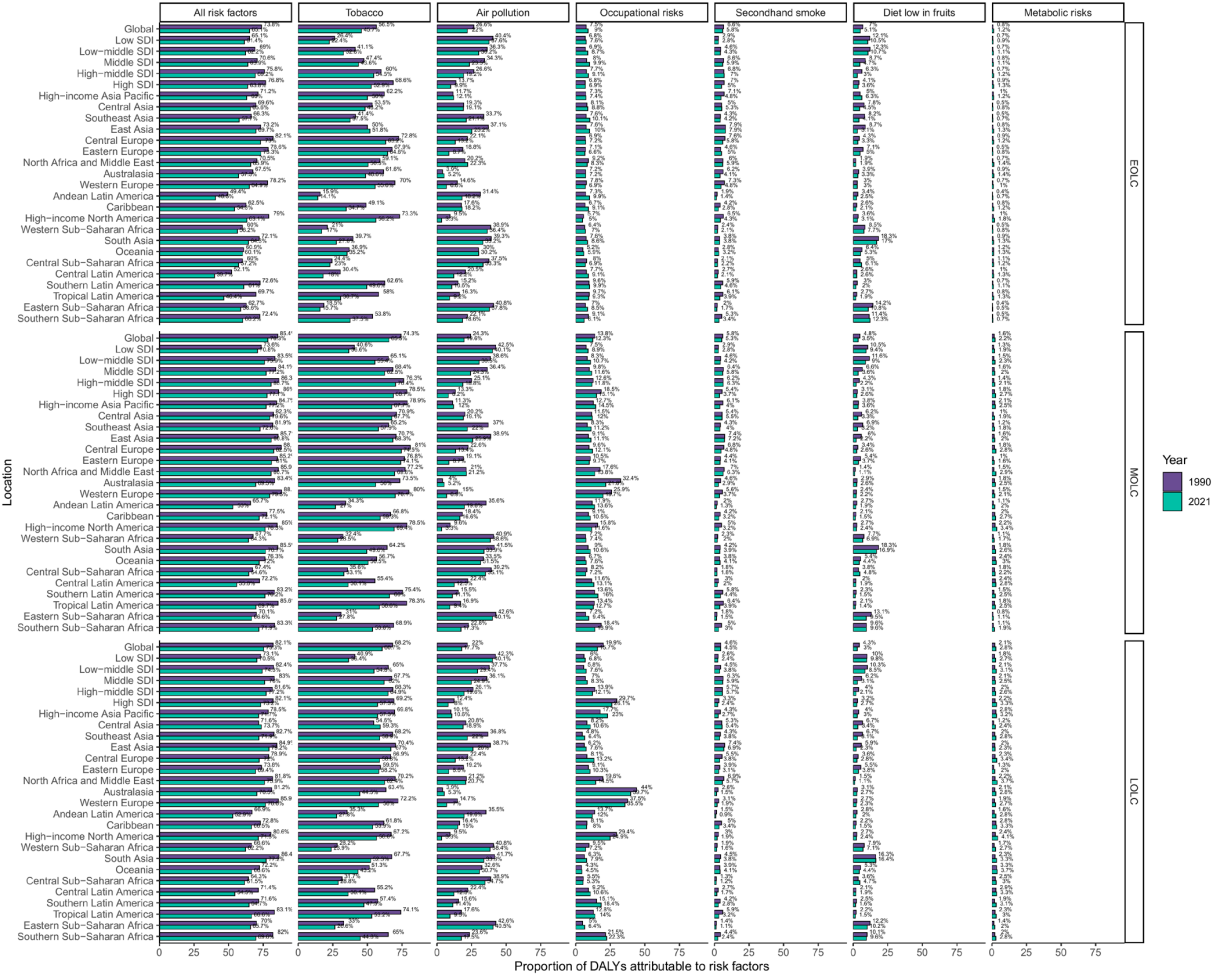
Percentage contributions of major risk factors to DALYs of EOLC, MOLC and LOLC at the global, SDI quintile, and regional levels in 1990 and 2021. DALYs, disability‐adjusted life‐years; EOLC, early‐onset lung cancer; MOLC, middle‐onset lung cancer; LOLC, late‐onset lung cancer; SDI: Socio‐demographic index.

We explored the contribution of overall and 6 main risk factors for DALYs of EOLC, MOLC and LOLC in 1990 and 2021 (Figure 7). Globally, tobacco (including smoking, chewing tobacco and secondhand smoke) was the leading risk factor for all of EOLC (45.7%, 95% CI: 39.1% to 51.1%), MOLC (65.5%, 95% CI: 59.5% to 70.3%) and LOLC (60.7%, 95% CI: 54.4% to 66.1%) in 2021, followed by air pollution (EOLC: 22.0%, MOLC: 19.6%, LOLC: 17.7%), occupational risks (EOLC: 9.0%, MOLC: 12.3%, LOLC: 15.7%), secondhand smoke (subcategory of tobacco) (EOLC: 5.8%, MOLC: 5.3%, LOLC: 4.5%), diet low in fruits (EOLC: 5.1%, MOLC: 3.5%, LOLC: 3.0%) and metabolic risks (EOLC: 1.2%, MOLC: 2.2%, LOLC: 2.8%). When stratified by SDI quintiles and regions, air pollution is identified as the predominant factor affecting health in the low SDI region, outpacing the impact of tobacco. Compared with 1990, the proportions of all risk factors showed a general decline trend. Notably, in contrast to the male population, a much lower proportion of DALYs attributable to all risk factors and tobacco were identified in females, leaving almost 40% to 50% of the total DALYs unattributable (Figures S22 and S23).

### 3.7 Prediction of EOLC, MOLC, and LOLC Burden to 2035

**FIGURE 8:**
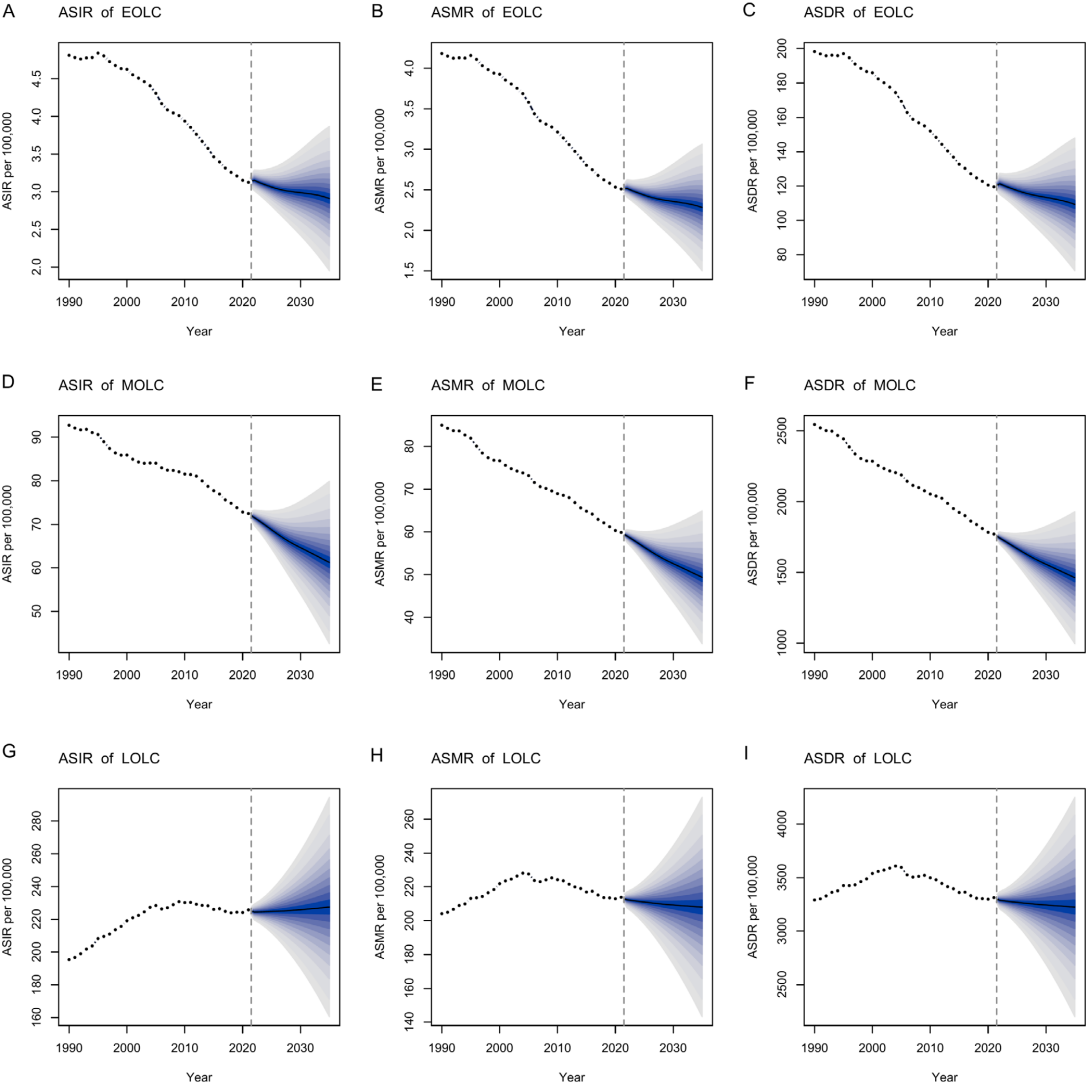
BAPC model predicted trends of ASIR, ASMR and ASDR for EOLC (A–C), MOLC (D–F), and LOLC (G–I): Observed (1990–2021) and predicted rates (2022–2035). The blue region in the figures shows the upper and lower limits of the 95% uncertainty intervals (95% UI). BAPC, Bayesian age‐period‐cohort; ASIR, age‐standardized incidence rate; ASMR, age‐standardized mortality rate; ASDR, age‐standardized disability‐adjusted life year rate; EOLC, early‐onset lung cancer; MOLC, middle‐onset lung cancer; LOLC, late‐onset lung cancer.

Except for a slight increase in ASIR of LOLC (Figure 8G), the global incidence, mortality and DALY rates of lung cancer across different age groups would continue to decrease from 2022 to 2035 (Figure 8 and Table S16). The decreasing trends in ASIR (apart from LOLC), ASMR and ASDR for males were similar to the overall population (Figure S24). From 2022 to 2035, the ASIR of LOLC in males were higher than in females, however, the ASIR, ASMR and ASDR of LOLC in females demonstrated remarkable increases (Figures S24 and S25). In sensitivity analysis of ARIMA models, the forecasting trends of EOLC, MOLC and LOLC were consistent with the BAPC results (Figures S26–S28 and Table S17).

## 4 Discussion

In the current study, we systematically evaluated the most update global trends of incidence, mortality and DALYs of EOLC, MOLC and LOLC from 1990 to 2021, with stratification of gender, SDI level, GBD region and country or territory. Although a general decline in global incidence, mortality and DALY rates was observed for EOLC and MOLC, the burden of LOLC presented a fluctuating and complex trend of change, indicating the necessity for specific public health policies addressing LOLC. The analysis by gender, geographic location and SDI level revealed that the burden spectrum of EOLC, MOLC and LOLC varied significantly among different demographic, geographic and socio‐economic patterns. Notably, 11 out of 21 GBD regions displayed an increase in the incidences of LOLC, with East Asia, Central Europe and Western Sub‐Saharan Africa exhibiting the most pronounced rise. Moreover, among the female population, the peculiar steadily increasing ASIR for LOLC, coupled with the negligible progress in cross‐country inequalities, pointed to a concerning trend that needed to be taken seriously.

Notably, we observed a pronounced difference in the global trends of EOLC, MOLC and LOLC, especially in females. Although both EOLC and MOLC showed a general decreasing trend in incidence, mortality and DALY rates from 1990 to 2021, LOLC showed a relatively stable trend, with potential slight increases. The underlying reasons for this remained obscure. Typically, the observed decline in incidence rate of all‐age lung cancer was considered mainly attributed to tobacco control in recent decades [27]. Among the well‐established risk factors for lung cancer, smoking is a major modifiable factor that causally increased the carcinogenesis of lung neoplasms, accounting for nearly 30% of all cancer‐related deaths [28, 29, 30]. In 2018, it was estimated that the prevalence of cigarette smoking among US adults was 13.7%, representing a two‐thirds decline since 1965, which contributed largely to the prevention and control of lung cancer [27]. However, according to survey findings, older smokers (≥ 65 years) were less likely to be motivated to quit smoking, to make quit attempts, and to achieve cessation, compared to younger adults [31, 32]. Accordingly, the effectiveness of smoking cessation in controlling LOLC might be compromised in the elderly. When demographic characteristics were considered, the global trends of population aging and increased life expectancy have resulted in the elderly experiencing prolonged exposure to environmental risk factors and genetic mutations, thereby elevating the potential for lung oncogenesis. Besides, given that the recommended age to begin lung cancer screening is 50, individuals aged 65 and older were reported to have a higher likelihood of undergoing annual screening compared to those aged 55 to 59 years [12, 33]. The growing trend in lung cancer screening for the elderly was associated with higher detection rates. Moreover, despite recent advancements in lung cancer treatment, elderly patients diagnosed with lung cancer were less likely to be subjected to surgery or radiation, and tended to experience more comorbidities and poorer outcomes, compared to younger patients [4]. The promotion of early screening and interventions for lung cancer could possibly further reduce the mortality and DALY rates among elderly patients.

Our results also revealed concerning trends of lung cancer burden in females, with a stability for MOLC and a significant rise for LOLC from 1990 to 2021. These findings provided further valuable insights into the disparities of gender and age groups, building upon earlier understandings [34]. While our understanding is not yet comprehensive, the observed trends in females might be partially attributed to the cigarette epidemic [35, 36], environmental risks [37, 38] and genetic factors [39]. According to the four‐stage model, the impact of cigarette epidemic presented a 20‐year to 30‐year lag on smoking rates and smoking‐related mortality in females, compared to males [35, 36]. This model provided an effective description for what has been experienced in many counyries, especially in economically developed nations. Therefore, the cumulative effects of smoking on incidence, mortality and DALYs of lung cancer could be lagged in females. Besides, secondhand smoke was also a nonegligible risk factor for lung cancer, particular in never‐smoked women [40]. Apart from smoking, in comparison with males, females were thought to be subject to a unique set of exogenous and endogenous exposures. For instance, occupational risks contributed to a rising attributable DALY rate of lung cancer in females in countries at the higher end of the SDI range. Higher degree of exposure to indoor cooking fumes was also considered to correlate with carcinogenesis of lung in the female population [37]. In addition, distinct genetic polymorphisms and estrogen might play potential roles in the incidence and deaths of lung cancer in females, but further exploration on more specific mechanisms is required [39].

At regional and national levels, a distinct disparity was observed in the trends of lung cancer incidence, mortality and DALY rates. Western Sub‐Saharan Africa, South Asia and Oceania were three GBD regions that had a significant increasing trend in the incidence of EOLC, while East Asia and Western Sub‐Saharan Africa were the two for MOLC. These regional variations could be attributed to a multifaceted interplay of factors, including local environment, cultural practices, economic development, lifestyle choices and genetic predispositions. In our study, the East Asia region was identified as experiencing a marked rise in incidence rates of LOLC. Recent epidemiological evidence indicated that adenocarcinoma has become the predominant subtype of lung cancer worldwide in 2020, with the highest ASIR per 100,000 population occurring in East Asia in both males (23.5) and females (16.0) [41]. The concerning trends in ASIR of lung adenocarcinoma might partially contributed to the overall rise in incidence of LOLC in East Asia, particularly among never‐smokers and women [41]. Besides, apart from secondhand smoke and indoor fuels, a high proportion of mutation in the epidermal growth factor receptor (EGFR) in East Asia (estimated 51.4%) could also account for, at least in part, the regional disparities observed in our study [42, 43]. In East Asia, China and Taiwan (Province of China) were the two with significant increases in incidence rate of LOLC. Therefore, especially among the Chinese population, control of environmental risk factors, early screening, examining the genetic characteristics and precision medicine may serve as effective strategies for managing LOLC.

Compared with lower SDI regions, countries or territories with more advanced socio‐demographic developments tended to accompany with more extensive lung cancer screenings, higher levels of cigarette pandemic, increased air pollution and more occupational exposures [36, 44, 45], which jointly contributed to the increasing trends for lung cancer burden. However, in more developed countries or territories, the general burden of lung cancer might be reduced by better prevention strategies, healthier lifestyles and stronger health care systems [14, 31, 33]. Consequently, the association between SDI and incidence, mortality and DALY rates of lung cancer demonstrated a general trend of gradual rises followed by sharp declines. Moreover, the practices and strategies of frontier countries or territories with relative low SDI were noteworthy, providing valuable reference points for the other nations, especially those with limited resources and heavy disease burden. In comparison, our findings revealed that there was greater potential for reducing lung cancer burdens in countries or territories with a higher SDI, such as Monaco, Greenland, Palau and Montenegro. In these nations or territories, the health benefits associated with socio‐demographic development appear to be overshadowed by other factors. Future investigations are required to clarify the drivers in frontier countries and obstacles faced by those lagging behind. Another notable point was the stagnation of improvement for cross‐country inequalities observed in females, despite remark progress has been achieved in the overall and male populations. More attention should be paid to the female group to further reduce the burden of lung cancer.

The present study is subject to several limitations. First, although the population‐level data used in this study were acquired from GBD 2021, a reputable database for global disease burden, the accuracy of the estimates was constrained by the quality of data from cancer registries across different countries. For instance, in many lower SDI countries or territories, the cases of lung cancer diagnosis and reporting might be underestimated due to limited cancer screening programs and health policies that prioritize non‐tumor diseases [34, 44]. Additionally, statistical estimation methods were employed when data for specific countries or years were unavailable [46]. Second, the implementation of a trichotomy at 50 and 70 years of ages inevitably presented limitations, since demographic, behavioral and molecular characteristics were unlikely to undergo significant changes at these specific ages. Nonetheless, the current selection of age ranges was based on clinical considerations, offering valuable insights for lung cancer prevention and management. Third, the concerning trends of LOLC burden, especially among females and in certain regions or countries, were still not well understood, suggesting the need for further investigation. Fourth, due to data unavailability within the GBD database, the temporal trends of other socio‐demographic factors, clinical features and pathological subtypes were not presented in this study. Therefore, further epidemiological investigations into the global, regional and national burden of lung cancer are essential to address these gaps.

## 5 Conclusions

In conclusion, the global ASIR, ASMR, and ASDR of EOLC and MOLC showed a general decrease from 1990 to 2021, especially in higher SDI regions. However, the burden of LOLC demonstrated a potential slight increase worldwide, with concerning steep rises observed among females and in certain regions and countries. Notably, in the female population, our findings revealed a stable trend for MOLC and a significant increase for LOLC in lung cancer incidence, mortality, and DALY rates since 1990, despite a slowdown in the rate of increase. These variations across genders and locations could partially explained by cigarette pandemic patterns, environmental exposures, lifestyle choices, molecular patterns and hormonal levels. Regarding socio‐demographic development, the low‐middle SDI region showed relatively modest increases in EOLC and MOLC incidence, while the middle and high‐middle SDI regions experienced a significant rise in LOLC incidence. Countries with higher SDIs were found to have a greater capacity for reducing lung cancer burdens. Moreover, our analysis suggested that more efforts were needed to improve cross‐country inequalities of lung cancer burden in females in the future. Our findings could help to guide more targeted prevention and intervention strategies for controlling lung cancer incidence, mortality and DALYs across genders and regions worldwide.

## Author Contributions

**Zongyuan Li:** conceptualization (lead), data curation (equal), investigation (equal), methodology (equal), resources (equal), software (equal), validation (equal), visualization (equal), writing – original draft (equal), writing – review and editing (equal). **Cheng Yu:** conceptualization (equal), formal analysis (equal), funding acquisition (equal), methodology (equal), supervision (equal), visualization (equal), writing – original draft (equal), writing – review and editing (equal). **Jianqi Hao:** methodology (equal), resources (equal), supervision (equal), validation (equal), writing – original draft (equal), writing – review and editing (equal). **Nanzhi Luo:** investigation (equal), methodology (equal), resources (equal), supervision (equal), validation (equal), writing – original draft (equal), writing – review and editing (equal). **Haoning Peng:** formal analysis (equal), methodology (equal), software (equal), visualization (equal), writing – original draft (equal), writing – review and editing (equal). **Jian Zhang:** formal analysis (equal), funding acquisition (equal), software (equal), validation (equal), writing – original draft (equal), writing – review and editing (equal). **Qiang Pu:** data curation (equal), methodology (equal), resources (equal), software (equal), validation (equal), writing – original draft (equal), writing – review and editing (equal). **Lunxu Liu:** conceptualization (equal), formal analysis (equal), funding acquisition (equal), methodology (equal), resources (equal), supervision (equal), writing – original draft (equal), writing – review and editing (equal).

## Conflicts of Interest

The authors declare no conflicts of interest.

## Supporting information


Figure S1.

Table S1.

## Data Availability

Data are available in a public, open access repository. To obtain the data used in this study, please visit the Global Health Data Exchange GBD Results Tool (http://ghdx.healthdata.org/gbd‐results‐tool).
