# Supplementary material for: Global Trends of Early, Middle, and Late‐Onset Lung Cancer From 1990 to 2021: Results From the Global Burden of Disease Study 2021
Source: Cancer Med. 2025 Feb 7;14(3):e70639. doi: 10.1002/cam4.70639 (PMC11803626; doi:10.1002/cam4.70639)

Supplementary Figures

Global Trends of Early, Middle and Late-onset Lung Cancer from 1990 to 2021: Results from the Global Burden of Disease Study 2021

Contents

Supplementary Figure S1. Joinpoint regression analysis of global incidence, mortality and burden for EOLC (A, B, C), MOLC (D, E, F) and LOLC (G, H, I) **in male** from 1990 to 2021. EOLC, early-onset lung cancer; MOLC, middle-onset lung cancer; LOLC, late-onset lung cancer; ASIR, age-standardized incidence rate; ASMR, age-standardized mortality rate; ASDR, age-standardized disability-adjusted life year rate; APC annual percentage change; * with significance, P value < 0.05. 6

Supplementary Figure S2. Joinpoint regression analysis of global incidence, mortality and burden for EOLC (A, B, C), MOLC (D, E, F) and LOLC (G, H, I) **in female** from 1990 to 2021. EOLC, early-onset lung cancer; MOLC, middle-onset lung cancer; LOLC, late-onset lung cancer; ASIR, age-standardized incidence rate; ASMR, age-standardized mortality rate; ASDR, age-standardized disability-adjusted life year rate; APC annual percentage change; * with significance, P value < 0.05. 7

Supplementary Figure S3. Among 204 countries and territories, the **ASMR** of EOLC (A), MOLC (C) and LOLC (E) in 2021, and the EAPC of ASMR for EOLC (B), MOLC (D) and LOLC (F) from 1990 to 2021. ASMR, age-standardized mortality rate; EOLC, early-onset lung cancer; MOLC, middle-onset lung cancer; LOLC, late-onset lung cancer; EAPC, estimated annual percent changes. 8

Supplementary Figure S4. Among 204 countries and territories, the **ASDR** of EOLC (A), MOLC (C) and LOLC (E) in 2021, and the EAPC of ASDR for EOLC (B), MOLC (D) and LOLC (F) from 1990 to 2021. ASDR, age-standardized disability-adjusted life year rate; EOLC, early-onset lung cancer; MOLC, middle-onset lung cancer; LOLC, late-onset lung cancer; EAPC, estimated annual percent changes... 9

Supplementary Figure S5. Among 204 countries and territories, the **ASIR** of EOLC (A), MOLC (C) and LOLC (E) **in male** in 2021, and the EAPC of ASIR for EOLC (B), MOLC (D) and LOLC (F) in male from 1990 to 2021. ASIR, age-standardized incidence rate; EOLC, early-onset lung cancer; MOLC, middle-onset lung cancer; LOLC, late-onset lung cancer; EAPC, estimated annual percent changes. 10

Supplementary Figure S6. Among 204 countries and territories, the **ASMR** of EOLC (A), MOLC (C) and LOLC (E) **in male** in 2021, and the EAPC of ASMR for EOLC (B), MOLC (D) and LOLC (F) in male from 1990 to 2021. ASMR, age-standardized mortality rate; EOLC, early-onset lung cancer;

MOLC, middle-onset lung cancer; LOLC, late-onset lung cancer; EAPC, estimated annual percent changes.. 11

Supplementary Figure S7. Among 204 countries and territories, the **ASDR** of EOLC (A), MOLC (C) and LOLC (E) **in male** in 2021, and the EAPC of ASDR for EOLC (B), MOLC (D) and LOLC (F) in male from 1990 to 2021. ASDR, age-standardized disability-adjusted life year rate; EOLC, early-onset lung cancer; MOLC, middle-onset lung cancer; LOLC, late-onset lung cancer; EAPC, estimated annual percent changes. 12

Supplementary Figure S8. Among 204 countries and territories, the **ASIR** of EOLC (A), MOLC (C) and LOLC (E) **in female** in 2021, and the EAPC of ASIR for EOLC (B), MOLC (D) and LOLC (F) in female from 1990 to 2021. ASIR, age-standardized incidence rate; EOLC, early-onset lung cancer; MOLC, middle-onset lung cancer; LOLC, late-onset lung cancer; EAPC, estimated annual percent changes.. 13

Supplementary Figure S9. Among 204 countries and territories, the **ASMR** of EOLC (A), MOLC (C) and LOLC (E) **in female** in 2021, and the EAPC of ASMR for EOLC (B), MOLC (D) and LOLC (F) in female from 1990 to 2021. ASMR, age-standardized mortality rate; EOLC, early-onset lung cancer; MOLC, middle-onset lung cancer; LOLC, late-onset lung cancer; EAPC, estimated annual percent changes.. 14

Supplementary Figure S10. Among 204 countries and territories, the **ASDR** of EOLC (A), MOLC (C) and LOLC (E) in female in 2021, and the EAPC of ASDR for EOLC (B), MOLC (D) and LOLC (F) **in female** from 1990 to 2021. ASDR, age-standardized disability-adjusted life year rate; EOLC, early-onset lung cancer; MOLC, middle-onset lung cancer; LOLC, late-onset lung cancer; EAPC, estimated annual percent changes. 15

Supplementary Figure S11. The association between SDI and incidence, mortality and burden of EOLC (A, B, C), MOLC (D, E, F) and LOLC (G, H, I) at the region level from 1990 to 2021. EOLC, early-onset lung cancer; MOLC, middle-onset lung cancer; LOLC, late-onset lung cancer; ASIR, age-standardized incidence rate; ASMR, age-standardized mortality rate; ASDR, age-standardized disability-adjusted life year rate; SDI, Socio-demographic index.. 16

Supplementary Figure S12. The association between SDI and incidence, mortality and burden of EOLC (A, B, C), MOLC (D, E, F) and LOLC (G, H, I) **in male** at the region level from 1990 to 2021. EOLC, early-onset lung cancer; MOLC, middle-onset lung cancer; LOLC, late-onset lung cancer; ASIR, age-standardized incidence rate; ASMR, age-standardized mortality rate; ASDR, age-standardized disability-adjusted life year rate; SDI, Socio-demographic index.. 17

Supplementary Figure S13. The association between SDI and incidence, mortality and burden of

EOLC (A, B, C), MOLC (D, E, F) and LOLC (G, H, I) **in female** at the region level from 1990 to 2021. EOLC, early-onset lung cancer; MOLC, middle-onset lung cancer; LOLC, late-onset lung cancer; ASIR, age-standardized incidence rate; ASMR, age-standardized mortality rate; ASDR, age-standardized disability-adjusted life year rate; SDI, Socio-demographic index.. 18

Supplementary Figure S14. The association between SDI and incidence, mortality and burden of EOLC (A, B, C), MOLC (D, E, F) and LOLC (G, H, I) at 204 countries and territories in 2021. EOLC, early-onset lung cancer; MOLC, middle-onset lung cancer; LOLC, late-onset lung cancer; ASIR, age-standardized incidence rate; ASMR, age-standardized mortality rate; ASDR, age-standardized disability-adjusted life year rate; SDI, Socio-demographic index.. 19

Supplementary Figure S15. The association between SDI and incidence, mortality and burden of EOLC (A, B, C), MOLC (D, E, F) and LOLC (G, H, I) **in male** at 204 countries and territories in 2021. EOLC, early-onset lung cancer; MOLC, middle-onset lung cancer; LOLC, late-onset lung cancer; ASIR, age-standardized incidence rate; ASMR, age-standardized mortality rate; ASDR, age-standardized disability-adjusted life year rate; SDI, Socio-demographic index.. 20

Supplementary Figure S16. The association between SDI and incidence, mortality and burden of EOLC (A, B, C), MOLC (D, E, F) and LOLC (G, H, I) **in female** at 204 countries and territories in 2021. EOLC, early-onset lung cancer; MOLC, middle-onset lung cancer; LOLC, late-onset lung cancer; ASIR, age-standardized incidence rate; ASMR, age-standardized mortality rate; ASDR, age-standardized disability-adjusted life year rate; SDI, Socio-demographic index.................................... 21

Supplementary Figure S17. Frontier analysis based on SDI and ASDR from 1990 to 2021. The frontier is depicted as a solid black line, with countries and territories represented by dots. SDI: Socio-demographic index; ASDR, age-standardized disability-adjusted life year rate; EOLC, early-onset lung cancer; MOLC, middle-onset lung cancer; LOLC, late-onset lung cancer. 22

Supplementary Figure S18. Global trends of SII for the DALYs of EOLC (A, B, C), MOLC (D, E, F) and LOLC (G, H, I), from 1990 to 2021. SII, slope index of inequality; DALYs, disability-adjusted life-years; EOLC, early-onset lung cancer; MOLC, middle-onset lung cancer; LOLC, late-onset lung cancer. 23

Supplementary Figure S19. Health inequality concentration curves for the DALYs of EOLC (A, B, C), MOLC (D, E, F) and LOLC (G, H, I) worldwide, in 1990 and 2021. The CI values are labeled in the bottom right corner of the figures. DALYs, disability-adjusted life-years; EOLC, early-onset lung cancer; MOLC, middle-onset lung cancer; LOLC, late-onset lung cancer; CI, concentration index. 24

Supplementary Figure S20. Changes in incidence, mortality, and DALYs for EOLC (A, B, C), MOLC (D, E, F), and LOLC (G, H, I) attributed to aging, population growth, and epidemiological changes **in**

male at the global, SDI quintile, and regional levels from 1990 to 2021. Black dots represent the cumulative contribution of all three factors to the observed changes. A positive value for any component reflects its contribution to an increase in lung cancer incidence, mortality, and DALYs, while a negative value indicates a reduction in these measures. DALYs, disability-adjusted life-years; EOLC, early-onset lung cancer; MOLC, middle-onset lung cancer; LOLC, late-onset lung cancer; SDI: Socio-demographic index... 25

Supplementary Figure S21. Changes in incidence, mortality, and DALYs for EOLC (A, B, C), MOLC (D, E, F), and LOLC (G, H, I) attributed to aging, population growth, and epidemiological changes **in female** at the global, SDI quintile, and regional levels from 1990 to 2021. Black dots represent the cumulative contribution of all three factors to the observed changes. A positive value for any component reflects its contribution to an increase in lung cancer incidence, mortality, and DALYs, while a negative value indicates a reduction in these measures. DALYs, disability-adjusted life-years; EOLC, early-onset lung cancer; MOLC, middle-onset lung cancer; LOLC, late-onset lung cancer; SDI: Socio-demographic index... 26

Supplementary Figure S22. Percentage contributions of major risk factors to DALYs of EOLC, MOLC and LOLC **in male** at the global, SDI quintile, and regional levels in 1990 and 2021. DALYs, disability-adjusted life-years; EOLC, early-onset lung cancer; MOLC, middle-onset lung cancer; LOLC, late-onset lung cancer; SDI: Socio-demographic index. 27

Supplementary Figure S23. Percentage contributions of major risk factors to DALYs of EOLC, MOLC and LOLC **in female** at the global, SDI quintile, and regional levels in 1990 and 2021. DALYs, disability-adjusted life-years; EOLC, early-onset lung cancer; MOLC, middle-onset lung cancer; LOLC, late-onset lung cancer; SDI: Socio-demographic index. 28

Supplementary Figure S24. BAPC model predicted trends of ASIR, ASMR and ASDR for EOLC (A, B, C), MOLC (D, E, F), and LOLC (G, H, I) **in male**: observed (1990 – 2021) and predicted rates (2022 – 2035). The blue region in the figures shows the upper and lower limits of the 95% uncertainty intervals (95% UI). BAPC, Bayesian age-period-cohort; ASIR, age-standardized incidence rate; ASMR, age-standardized mortality rate; ASDR, age-standardized disability-adjusted life year rate; EOLC, early-onset lung cancer; MOLC, middle-onset lung cancer; LOLC, late-onset lung cancer......................... 29

Supplementary Figure S25. BAPC model predicted trends of ASIR, ASMR and ASDR for EOLC (A, B, C), MOLC (D, E, F), and LOLC (G, H, I) **in female**: observed (1990 – 2021) and predicted rates (2022 – 2035). The blue region in the figures shows the upper and lower limits of the 95% uncertainty intervals (95% UI). BAPC, Bayesian age-period-cohort; ASIR, age-standardized incidence rate; ASMR, age-standardized mortality rate; ASDR, age-standardized disability-adjusted life year rate; EOLC, early-

onset lung cancer; MOLC, middle-onset lung cancer; LOLC, late-onset lung cancer. 30

Supplementary Figure S26. ARIMA model predicted trends of ASIR, ASMR and ASDR for EOLC (A, B, C), MOLC (D, E, F), and LOLC (G, H, I): observed (1990 – 2021) and predicted rates (2022 – 2035). The blue region in the figures shows the upper and lower limits of the 95% uncertainty intervals (95% UI). ARIMA, autoregressive integrated moving average; ASIR, age-standardized incidence rate; ASMR, age-standardized mortality rate; ASDR, age-standardized disability-adjusted life year rate; EOLC, early-onset lung cancer; MOLC, middle-onset lung cancer; LOLC, late-onset lung cancer. 31

Supplementary Figure S27. ARIMA model predicted trends of ASIR, ASMR and ASDR for EOLC (A, B, C), MOLC (D, E, F), and LOLC (G, H, I) **in male**: observed (1990 – 2021) and predicted rates (2022 – 2035). The blue region in the figures shows the upper and lower limits of the 95% uncertainty intervals (95% UI). ARIMA, autoregressive integrated moving average; ASIR, age-standardized incidence rate; ASMR, age-standardized mortality rate; ASDR, age-standardized disability-adjusted life year rate; EOLC, early-onset lung cancer; MOLC, middle-onset lung cancer; LOLC, late-onset lung cancer. 32

Supplementary Figure S28. ARIMA model predicted trends of ASIR, ASMR and ASDR for EOLC (A, B, C), MOLC (D, E, F), and LOLC (G, H, I) **in female**: observed (1990 – 2021) and predicted rates (2022 – 2035). The blue region in the figures shows the upper and lower limits of the 95% uncertainty intervals (95% UI). ARIMA, autoregressive integrated moving average; ASIR, age-standardized incidence rate; ASMR, age-standardized mortality rate; ASDR, age-standardized disability-adjusted life year rate; EOLC, early-onset lung cancer; MOLC, middle-onset lung cancer; LOLC, late-onset lung cancer. 33

Supplementary Figure S1. Joinpoint regression analysis of global incidence, mortality and burden for EOLC (A, B, C), MOLC (D, E, F) and LOLC (G, H, I) **in male** from 1990 to 2021. EOLC, early-onset lung cancer; MOLC, middle-onset lung cancer; LOLC, late-onset lung cancer; ASIR, age-standardized incidence rate; ASMR, age-standardized mortality rate; ASDR, age-standardized disability-adjusted life year rate; APC annual percentage change; * with significance, P value < 0.05.

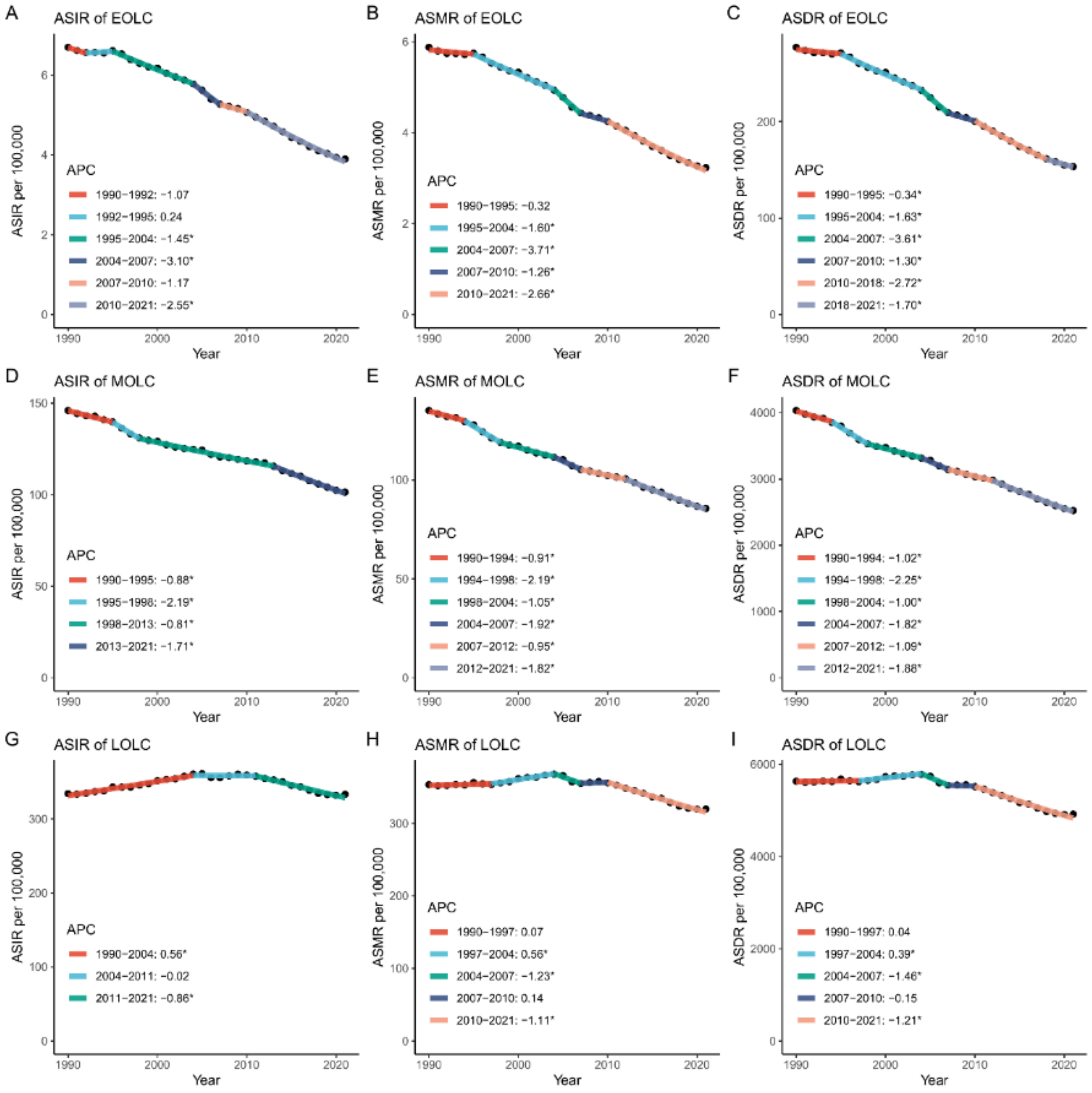

Supplementary Figure S2. Joinpoint regression analysis of global incidence, mortality and burden for EOLC (A, B, C), MOLC (D, E, F) and LOLC (G, H, I) **in female** from 1990 to 2021. EOLC, early-onset lung cancer; MOLC, middle-onset lung cancer; LOLC, late-onset lung cancer; ASIR, age-standardized incidence rate; ASMR, age-standardized mortality rate; ASDR, age-standardized disability-adjusted life year rate; APC annual percentage change; * with significance, P value < 0.05.

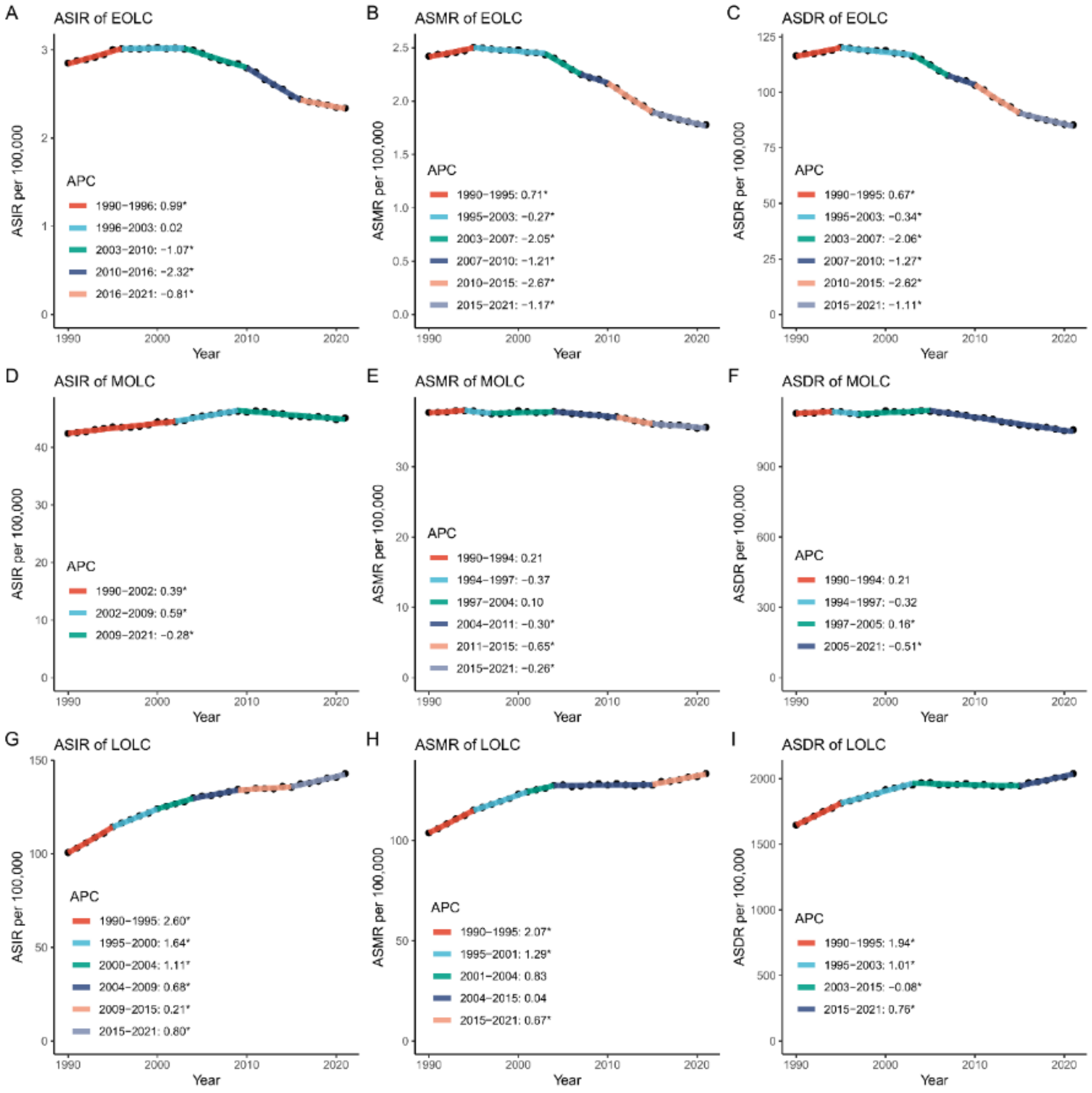

Supplementary Figure S3. Among 204 countries and territories, the **ASMR** of EOLC (A), MOLC (C) and LOLC (E) in 2021, and the EAPC of ASMR for EOLC (B), MOLC (D) and LOLC (F) from 1990 to 2021. ASMR, age-standardized mortality rate; EOLC, early-onset lung cancer; MOLC, middle-onset lung cancer; LOLC, late-onset lung cancer; EAPC, estimated annual percent changes.

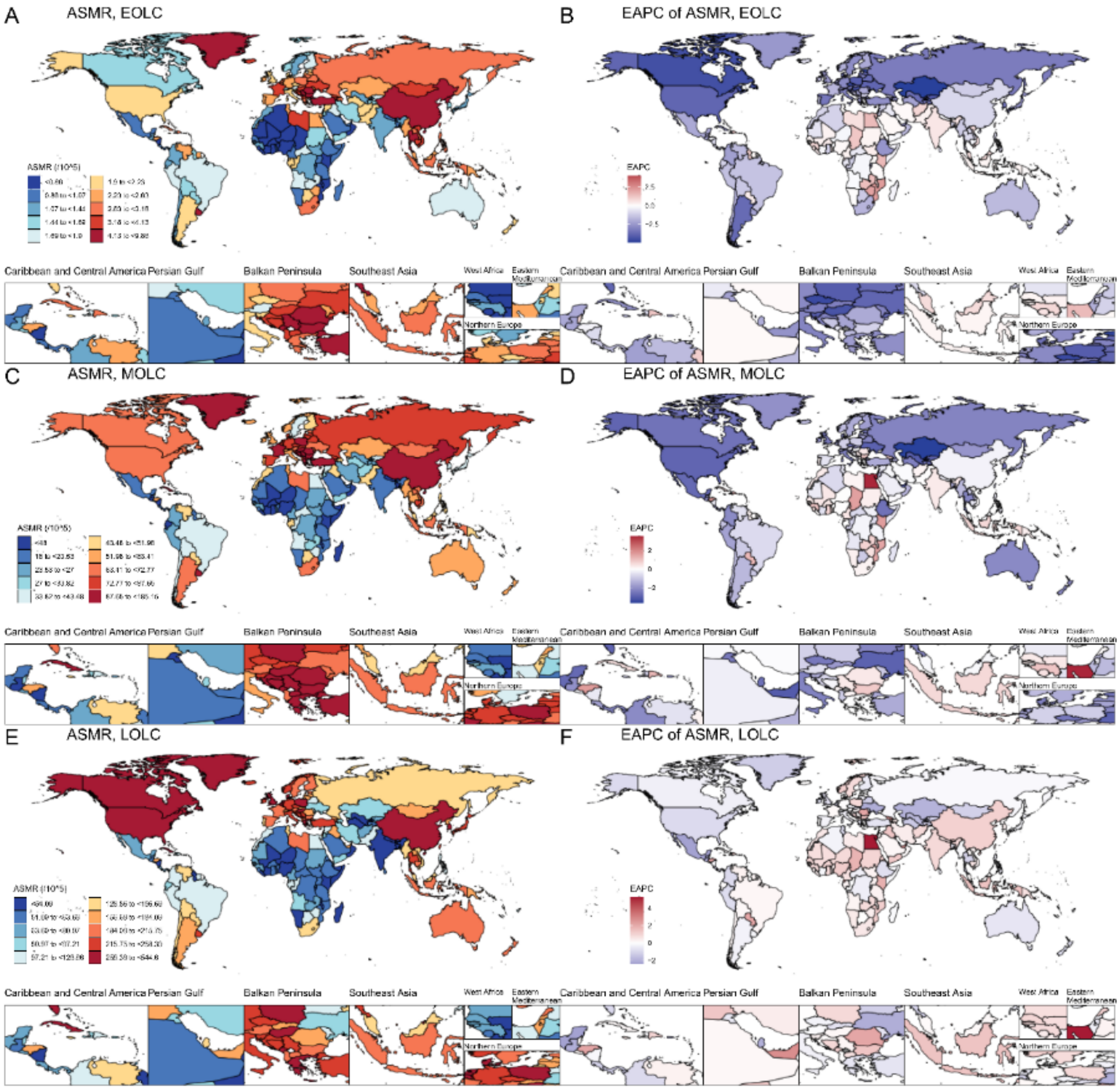

Supplementary Figure S4. Among 204 countries and territories, the **ASDR** of EOLC (A), MOLC (C) and LOLC (E) in 2021, and the EAPC of ASDR for EOLC (B), MOLC (D) and LOLC (F) from 1990 to 2021. ASDR, age-standardized disability-adjusted life year rate; EOLC, early-onset lung cancer; MOLC, middle-onset lung cancer; LOLC, late-onset lung cancer; EAPC, estimated annual percent changes.

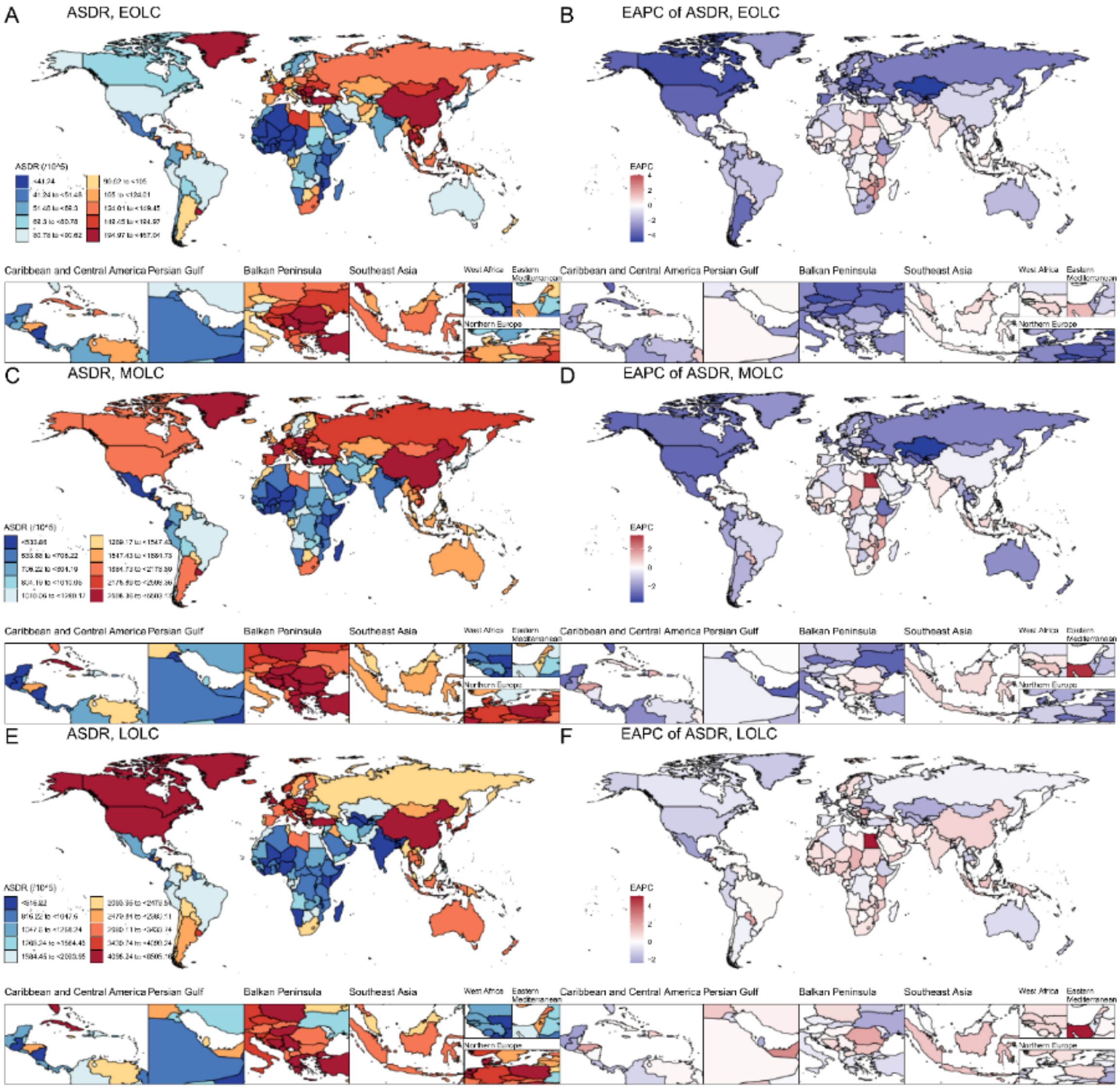

Supplementary Figure S5. Among 204 countries and territories, the **ASIR** of EOLC (A), MOLC (C) and LOLC (E) **in male** in 2021, and the EAPC of ASIR for EOLC (B), MOLC (D) and LOLC (F) in male from 1990 to 2021. ASIR, age-standardized incidence rate; EOLC, early-onset lung cancer; MOLC, middle-onset lung cancer; LOLC, late-onset lung cancer; EAPC, estimated annual percent changes.

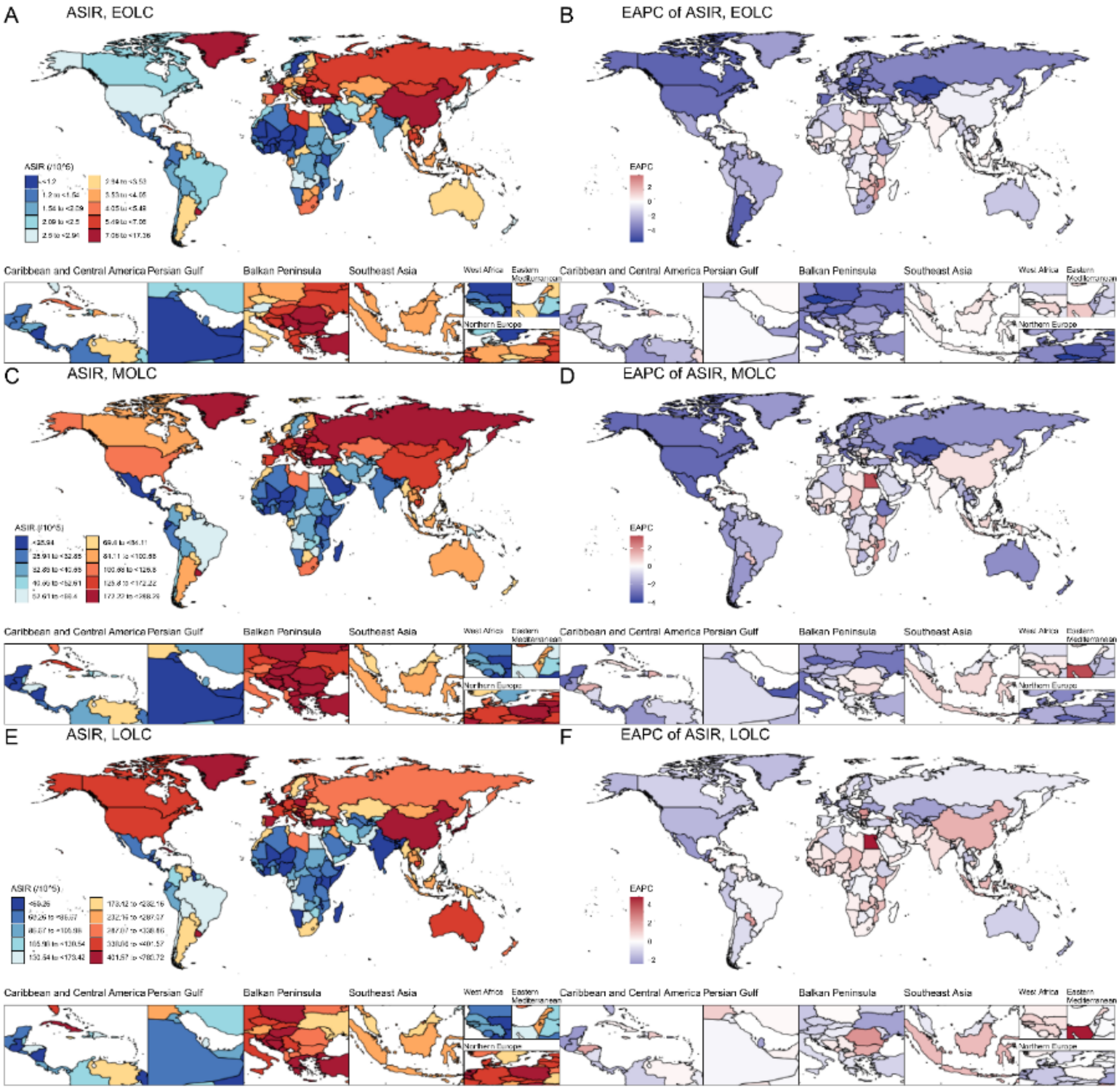

Supplementary Figure S6. Among 204 countries and territories, the **ASMR** of EOLC (A), MOLC (C) and LOLC (E) **in male** in 2021, and the EAPC of ASMR for EOLC (B), MOLC (D) and LOLC (F) in male from 1990 to 2021. ASMR, age-standardized mortality rate; EOLC, early-onset lung cancer; MOLC, middle-onset lung cancer; LOLC, late-onset lung cancer; EAPC, estimated annual percent changes.

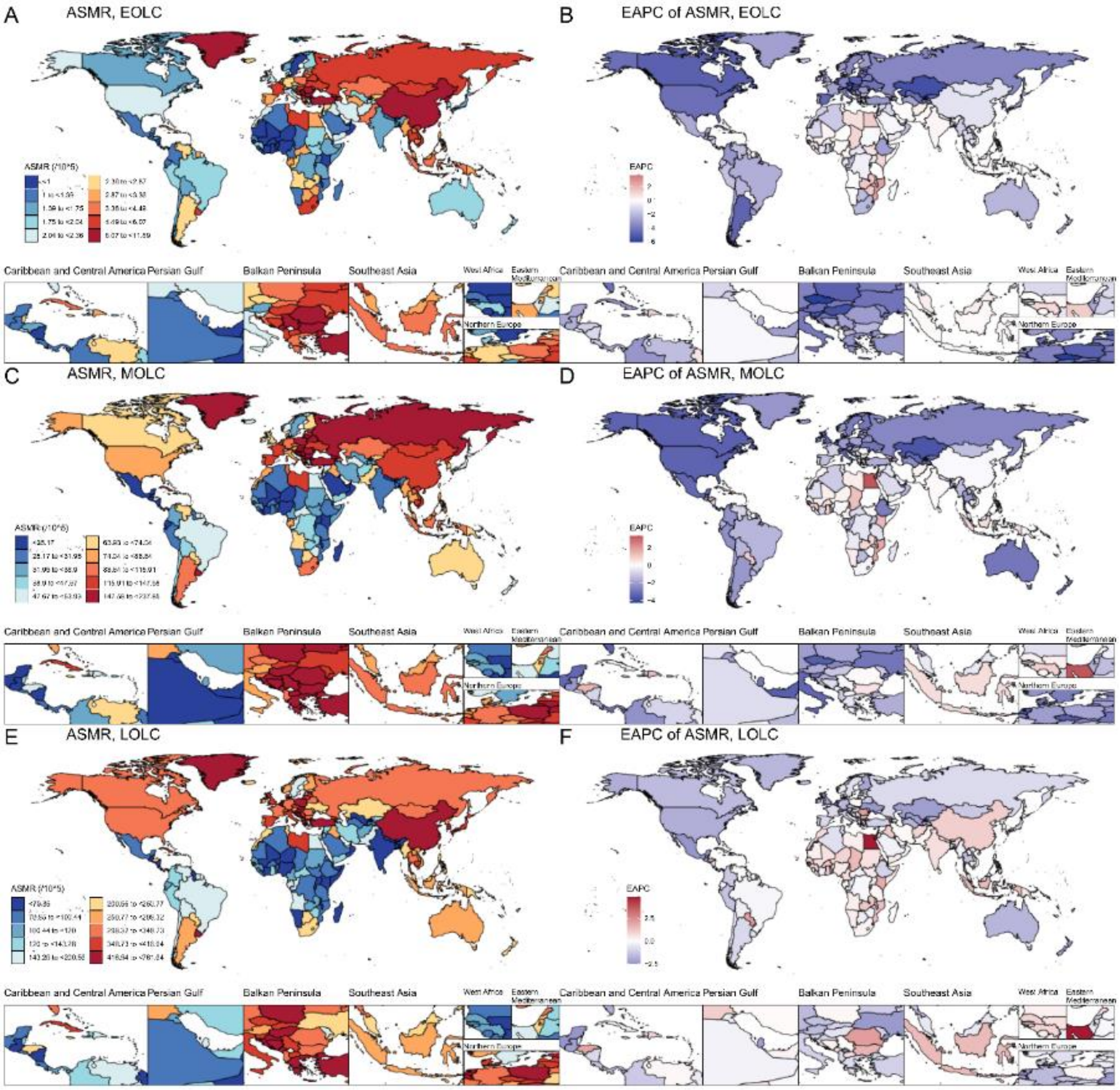

Supplementary Figure S7. Among 204 countries and territories, the **ASDR** of EOLC (A), MOLC (C) and LOLC (E) **in male** in 2021, and the EAPC of ASDR for EOLC (B), MOLC (D) and LOLC (F) in male from 1990 to 2021. ASDR, age-standardized disability-adjusted life year rate; EOLC, early-onset lung cancer; MOLC, middle-onset lung cancer; LOLC, late-onset lung cancer; EAPC, estimated annual percent changes.

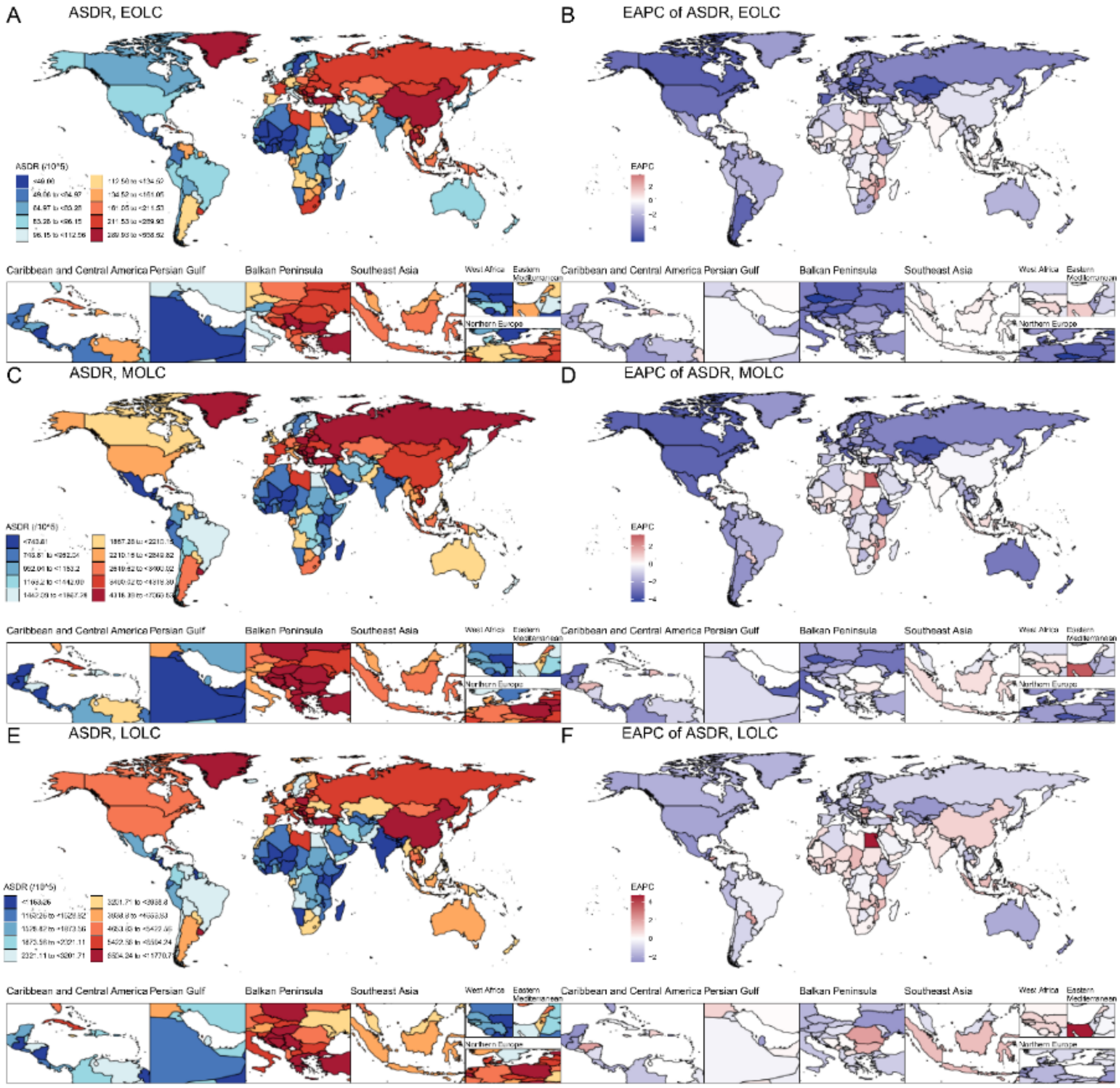

Supplementary Figure S8. Among 204 countries and territories, the **ASIR** of EOLC (A), MOLC (C) and LOLC (E) **in female** in 2021, and the EAPC of ASIR for EOLC (B), MOLC (D) and LOLC (F) in female from 1990 to 2021. ASIR, age-standardized incidence rate; EOLC, early-onset lung cancer; MOLC, middle-onset lung cancer; LOLC, late-onset lung cancer; EAPC, estimated annual percent changes.

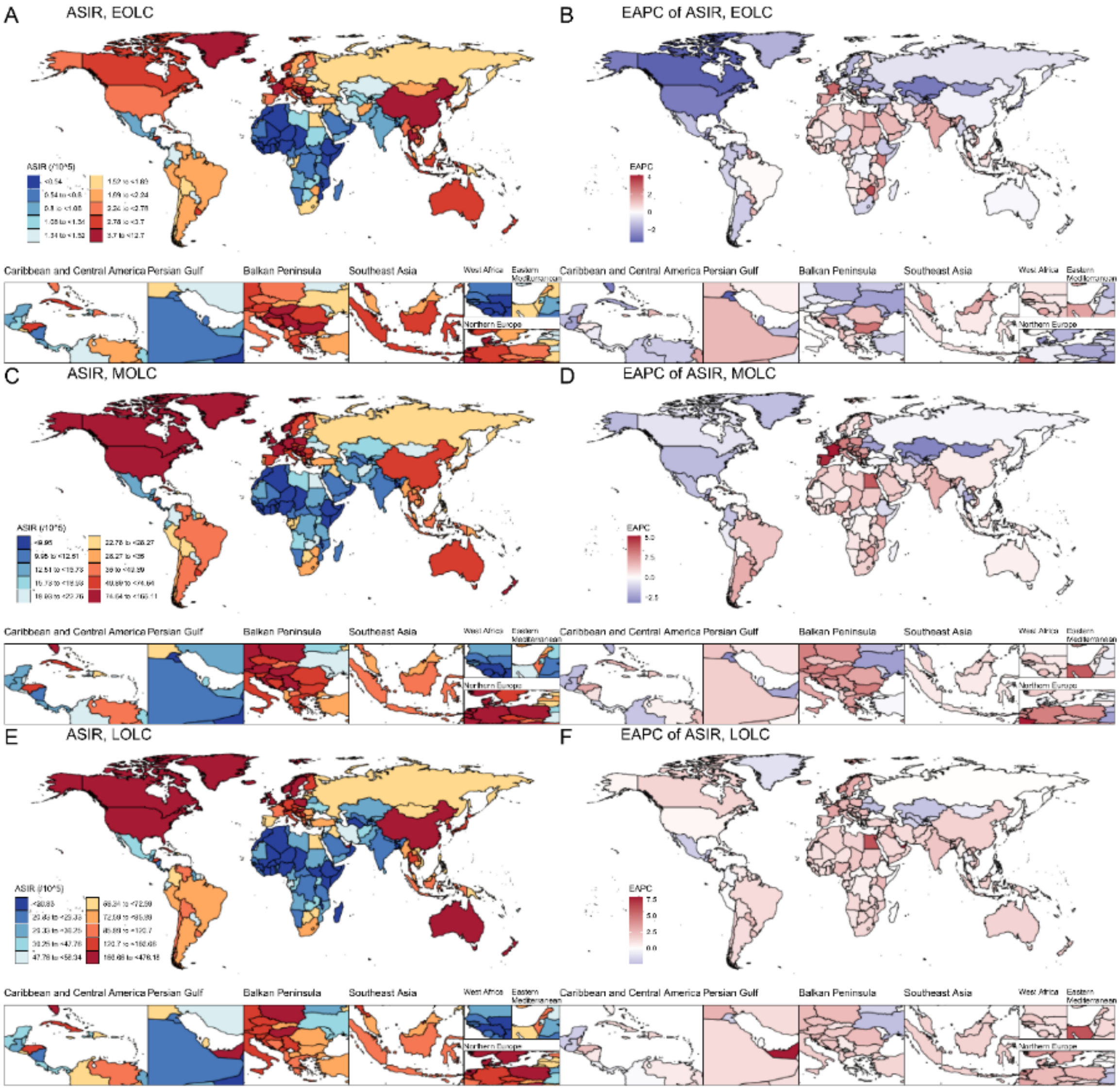

Supplementary Figure S9. Among 204 countries and territories, the **ASMR** of EOLC (A), MOLC (C) and LOLC (E) **in female** in 2021, and the EAPC of ASMR for EOLC (B), MOLC (D) and LOLC (F) in female from 1990 to 2021. ASMR, age-standardized mortality rate; EOLC, early-onset lung cancer; MOLC, middle-onset lung cancer; LOLC, late-onset lung cancer; EAPC, estimated annual percent changes.

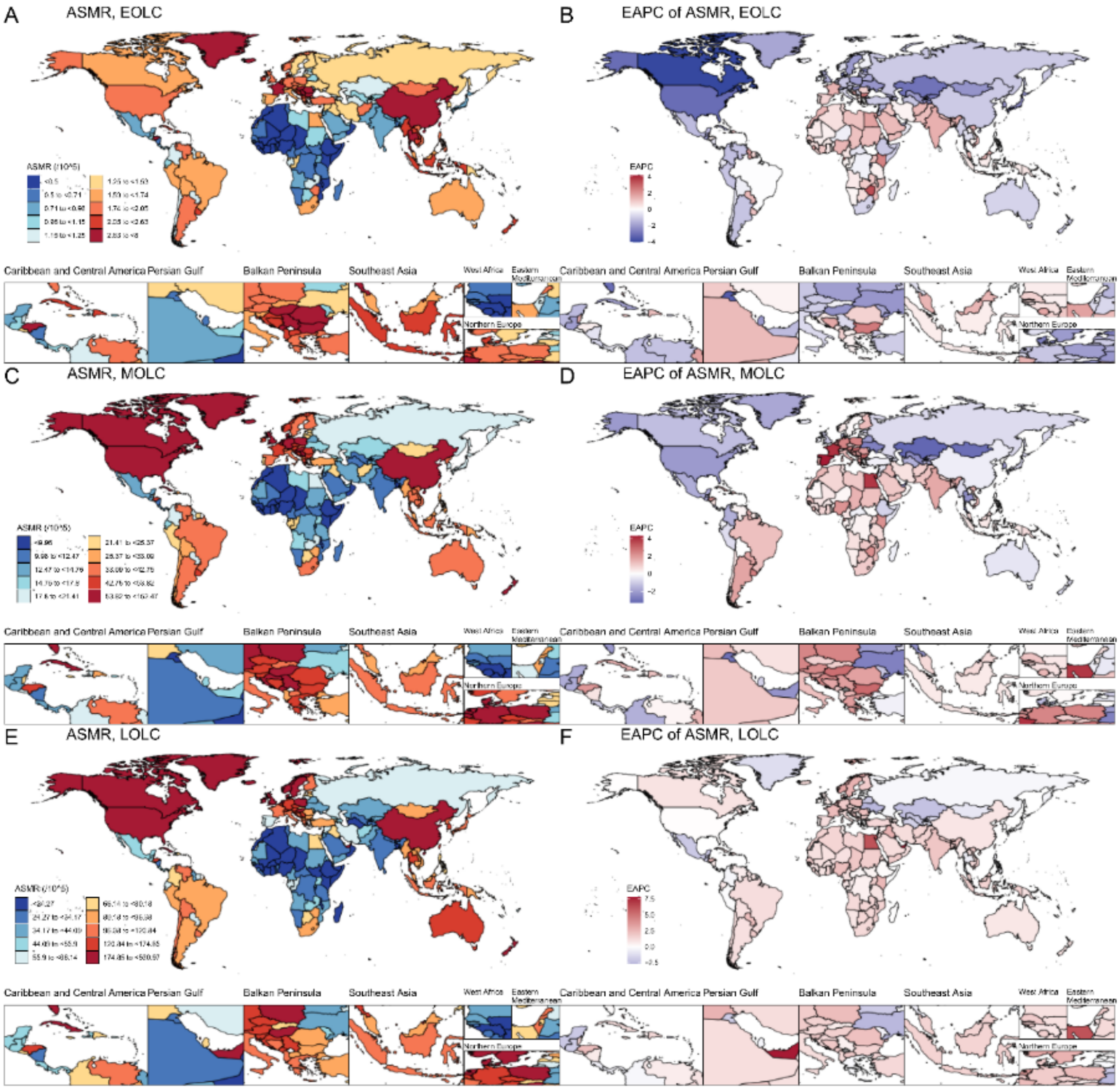

Supplementary Figure S10. Among 204 countries and territories, the **ASDR** of EOLC (A), MOLC (C) and LOLC (E) in female in 2021, and the EAPC of ASDR for EOLC (B), MOLC (D) and LOLC (F) **in female** from 1990 to 2021. ASDR, age-standardized disability-adjusted life year rate; EOLC, early-onset lung cancer; MOLC, middle-onset lung cancer; LOLC, late-onset lung cancer; EAPC, estimated annual percent changes.

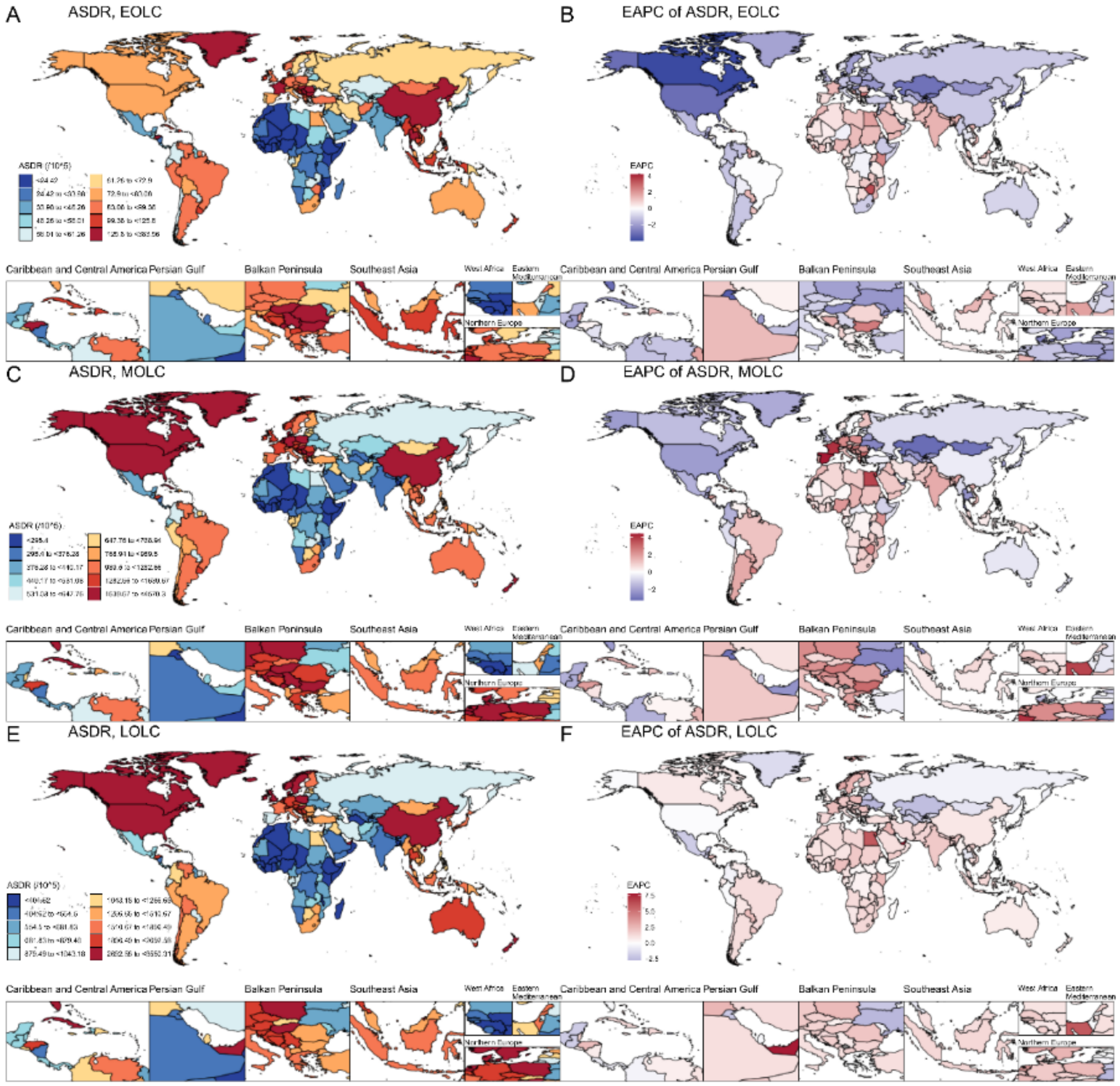

Supplementary Figure S11. The association between SDI and incidence, mortality and burden of EOLC (A, B, C), MOLC (D, E, F) and LOLC (G, H, I) at the region level from 1990 to 2021. EOLC, early-onset lung cancer; MOLC, middle-onset lung cancer; LOLC, late-onset lung cancer; ASIR, age-standardized incidence rate; ASMR, age-standardized mortality rate; ASDR, age-standardized disability-adjusted life year rate; SDI, Socio-demographic index.

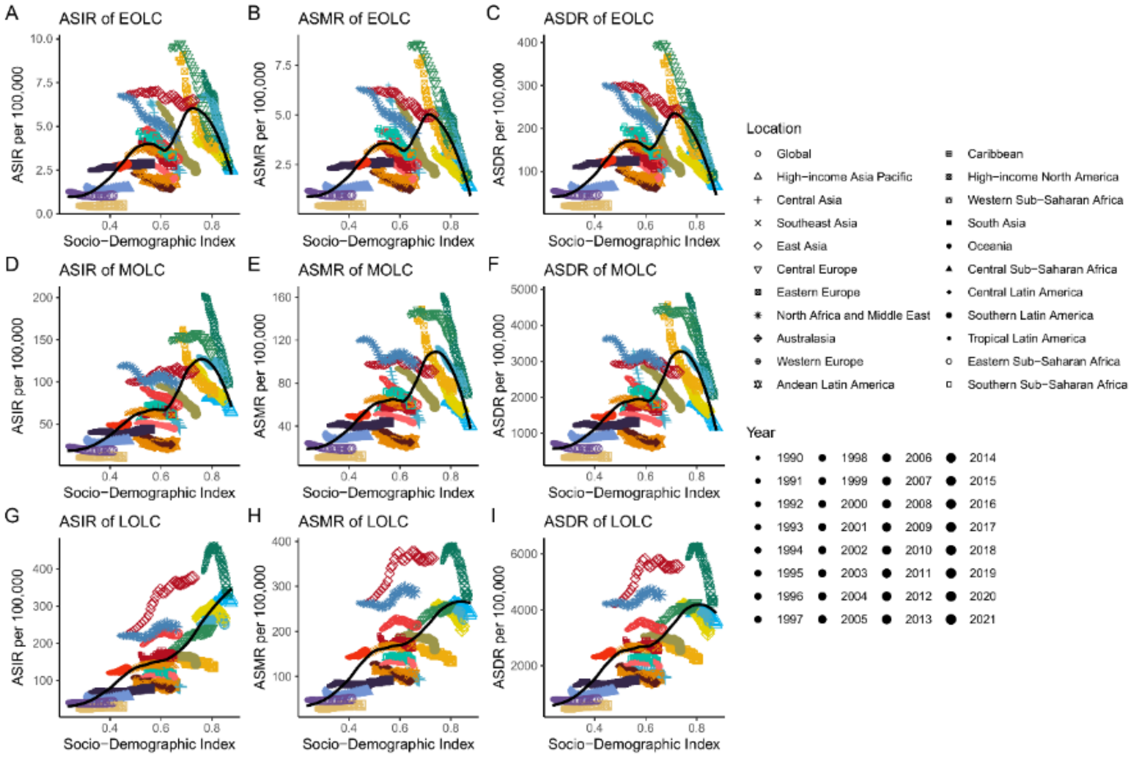

Supplementary Figure S12. The association between SDI and incidence, mortality and burden of EOLC (A, B, C), MOLC (D, E, F) and LOLC (G, H, I) **in male** at the region level from 1990 to 2021. EOLC, early-onset lung cancer; MOLC, middle-onset lung cancer; LOLC, late-onset lung cancer; ASIR, age-standardized incidence rate; ASMR, age-standardized mortality rate; ASDR, age-standardized disability-adjusted life year rate; SDI, Socio-demographic index.

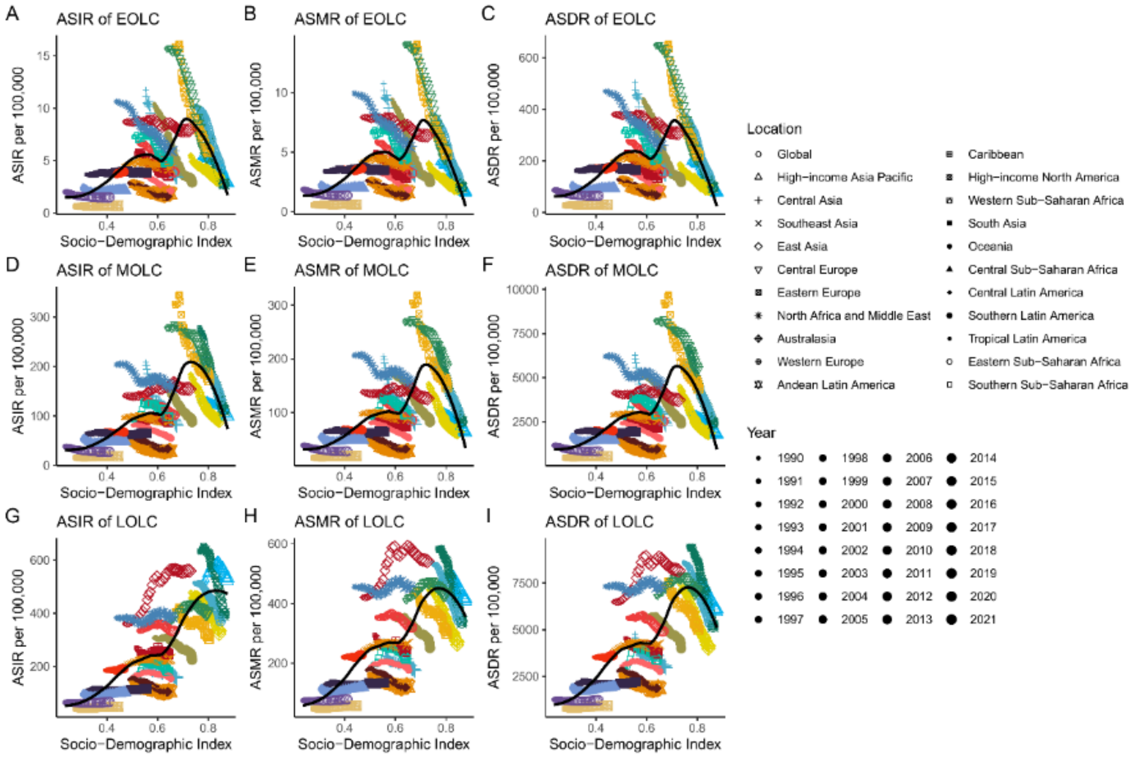

Supplementary Figure S13. The association between SDI and incidence, mortality and burden of EOLC (A, B, C), MOLC (D, E, F) and LOLC (G, H, I) **in female** at the region level from 1990 to 2021. EOLC, early-onset lung cancer; MOLC, middle-onset lung cancer; LOLC, late-onset lung cancer; ASIR, age-standardized incidence rate; ASMR, age-standardized mortality rate; ASDR, age-standardized disability-adjusted life year rate; SDI, Socio-demographic index.

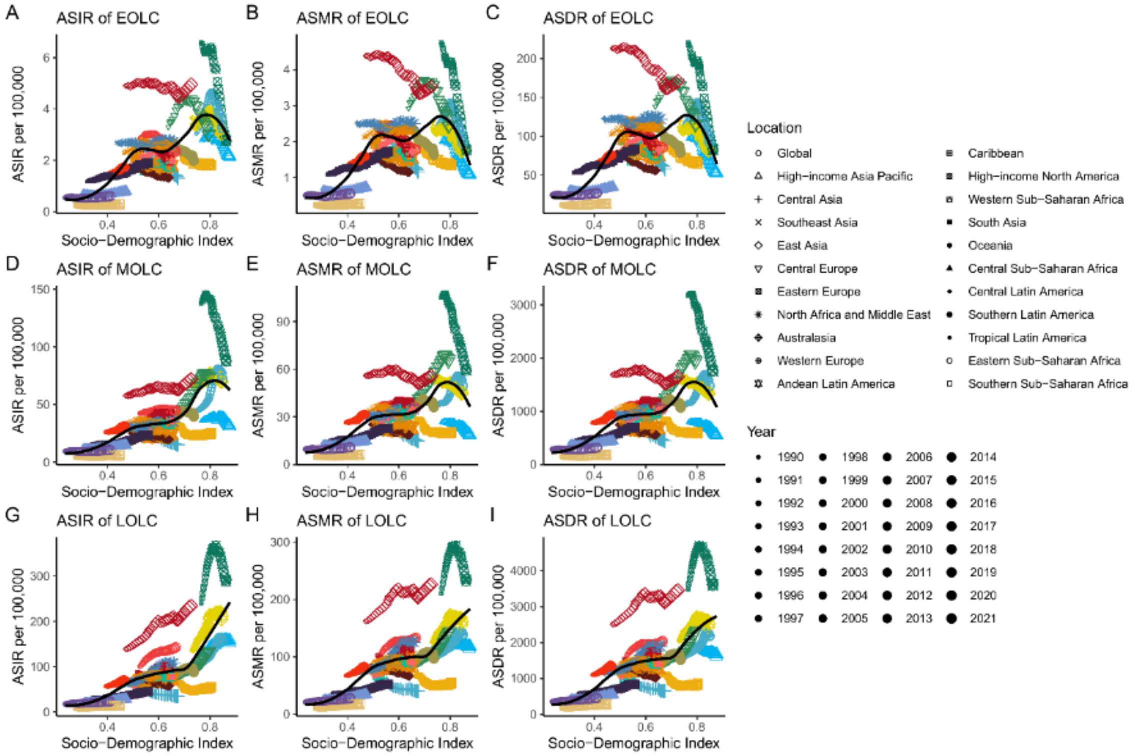

Supplementary Figure S14. The association between SDI and incidence, mortality and burden of EOLC (A, B, C), MOLC (D, E, F) and LOLC (G, H, I) at 204 countries and territories in 2021. EOLC, early-onset lung cancer; MOLC, middle-onset lung cancer; LOLC, late-onset lung cancer; ASIR, age-standardized incidence rate; ASMR, age-standardized mortality rate; ASDR, age-standardized disability-adjusted life year rate; SDI, Socio-demographic index.

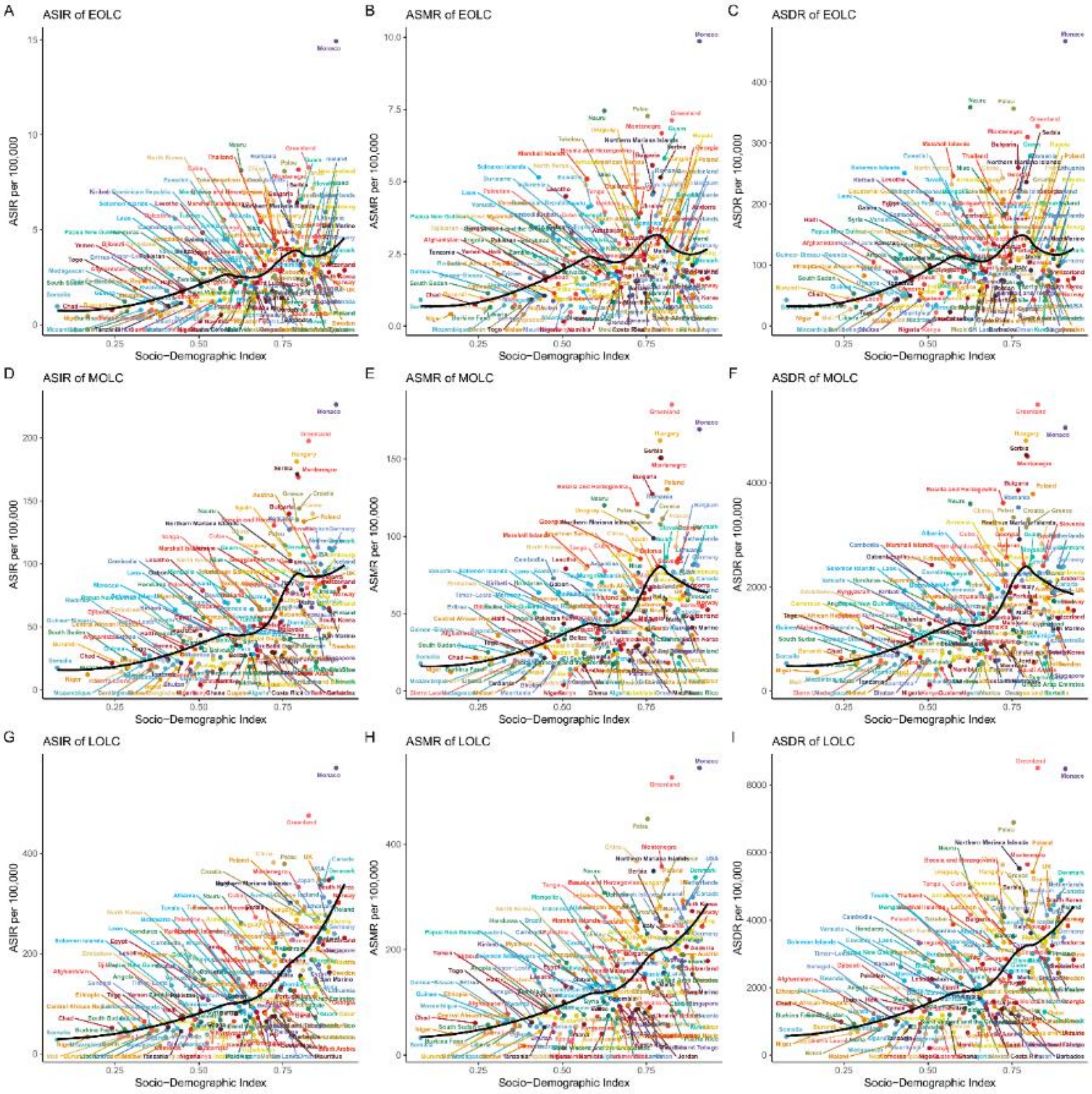

Supplementary Figure S15. The association between SDI and incidence, mortality and burden of EOLC (A, B, C), MOLC (D, E, F) and LOLC (G, H, I) **in male** at 204 countries and territories in 2021. EOLC, early-onset lung cancer; MOLC, middle-onset lung cancer; LOLC, late-onset lung cancer; ASIR, age-standardized incidence rate; ASMR, age-standardized mortality rate; ASDR, age-standardized disability-adjusted life year rate; SDI, Socio-demographic index.

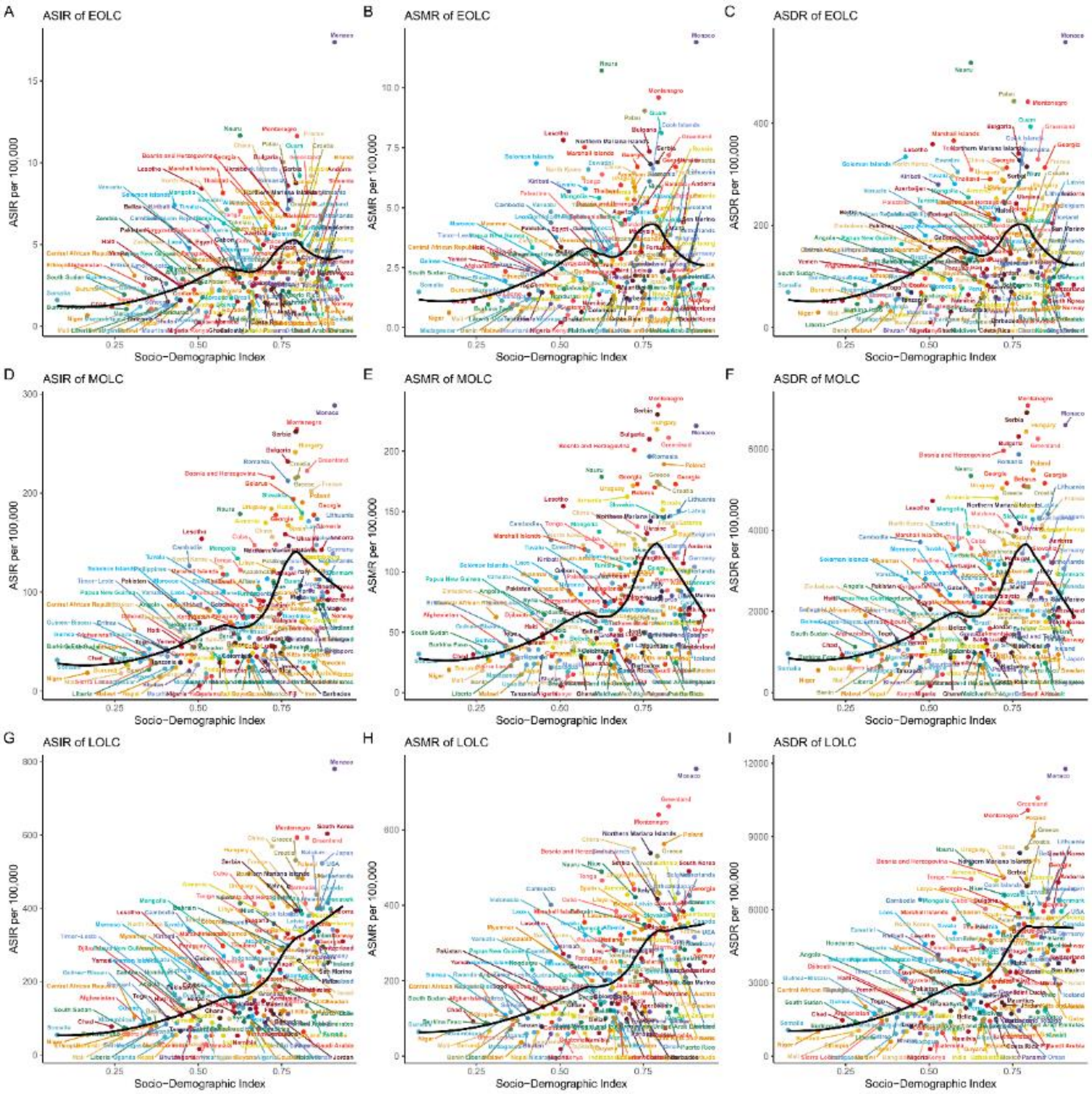

Supplementary Figure S16. The association between SDI and incidence, mortality and burden of EOLC (A, B, C), MOLC (D, E, F) and LOLC (G, H, I) **in female** at 204 countries and territories in 2021. EOLC, early-onset lung cancer; MOLC, middle-onset lung cancer; LOLC, late-onset lung cancer; ASIR, age-standardized incidence rate; ASMR, age-standardized mortality rate; ASDR, age-standardized disability-adjusted life year rate; SDI, Socio-demographic index.

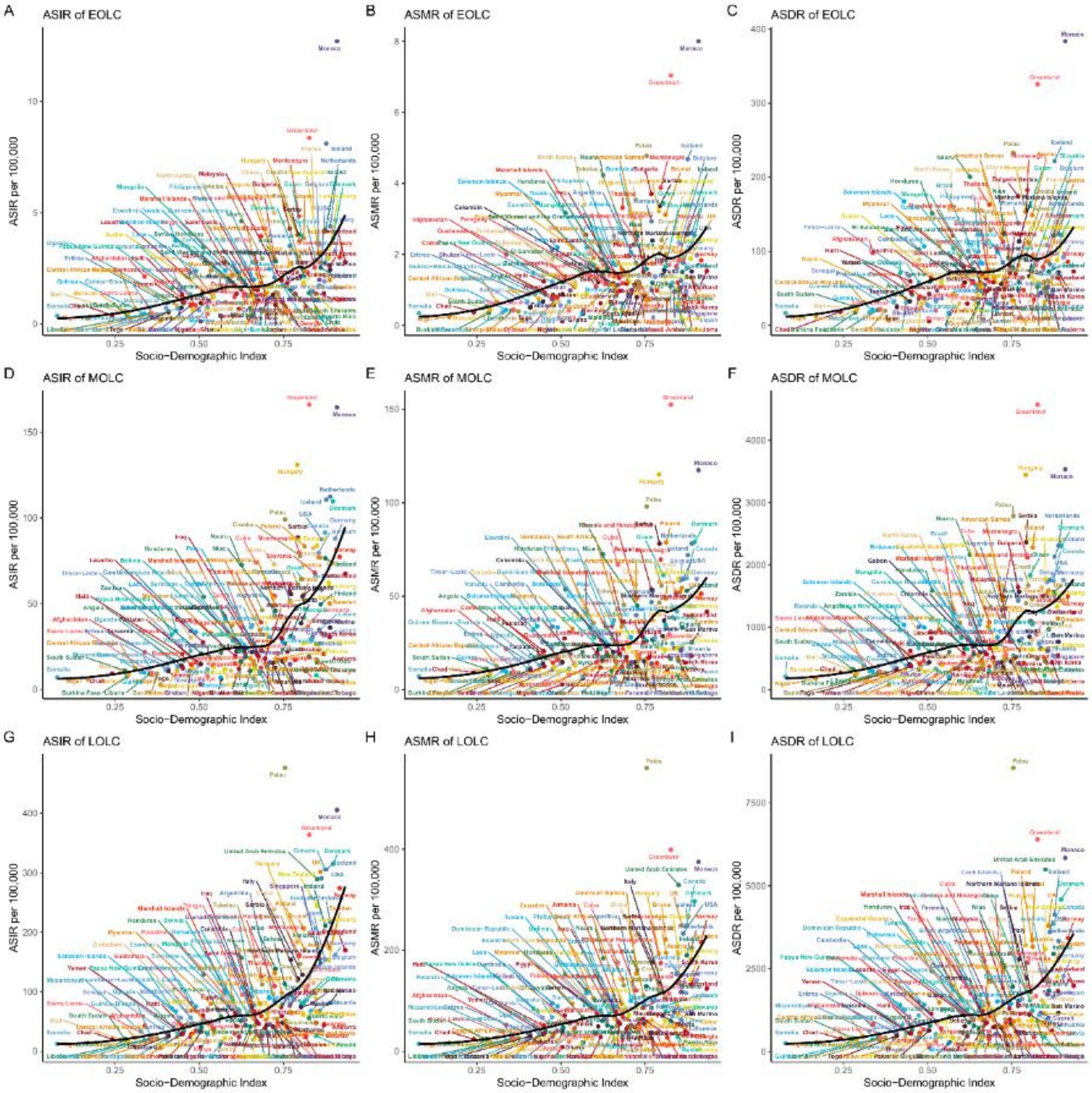

Supplementary Figure S17. Frontier analysis based on SDI and ASDR from 1990 to 2021. The frontier is depicted as a solid black line, with countries and territories represented by dots. SDI: Socio-demographic index; ASDR, age-standardized disability-adjusted life year rate; EOLC, early-onset lung cancer; MOLC, middle-onset lung cancer; LOLC, late-onset lung cancer.

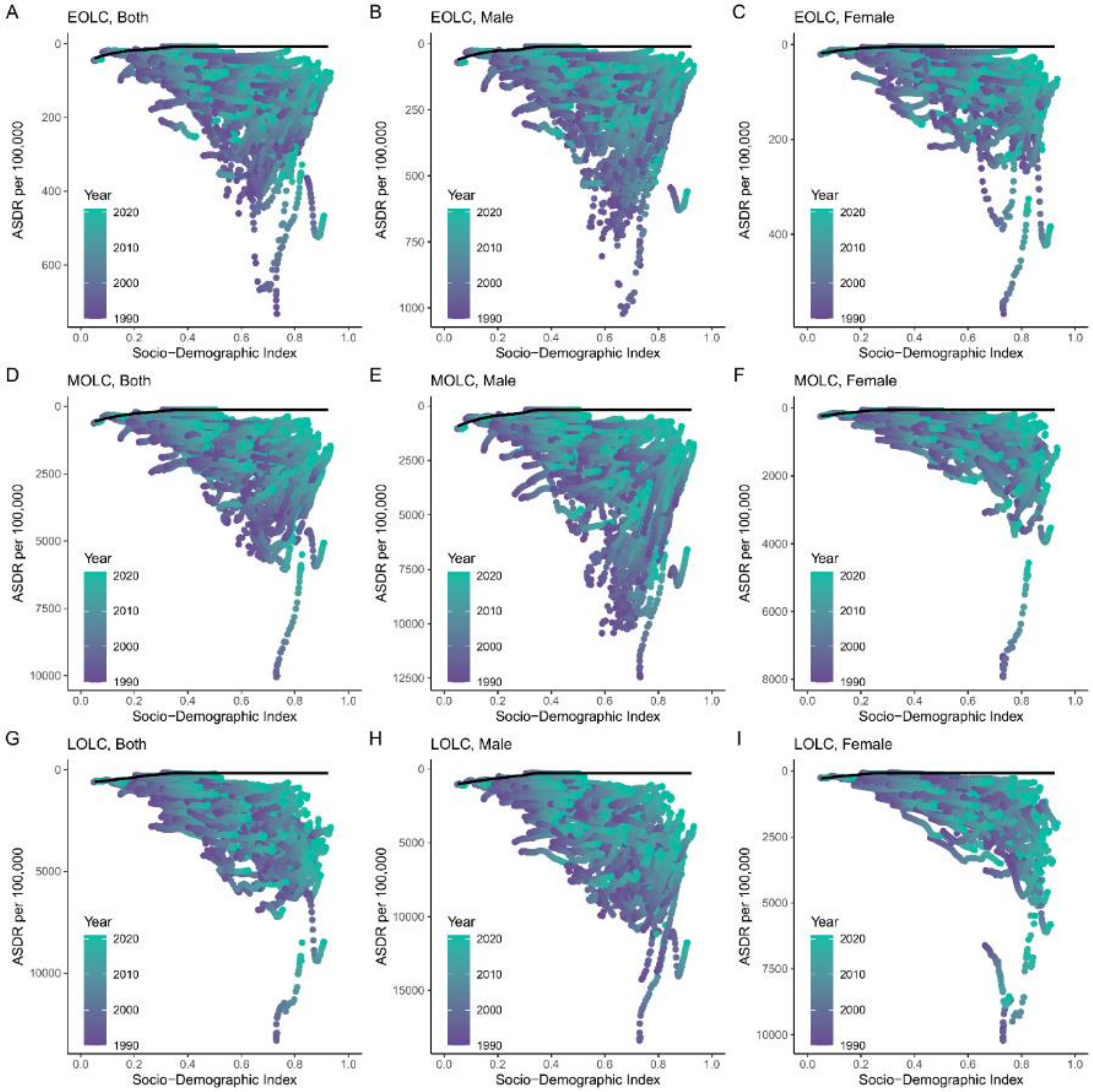

Supplementary Figure S18. Global trends of SII for the DALYs of EOLC (A, B, C), MOLC (D, E, F) and LOLC (G, H, I), from 1990 to 2021. SII, slope index of inequality; DALYs, disability-adjusted life-years; EOLC, early-onset lung cancer; MOLC, middle-onset lung cancer; LOLC, late-onset lung cancer.

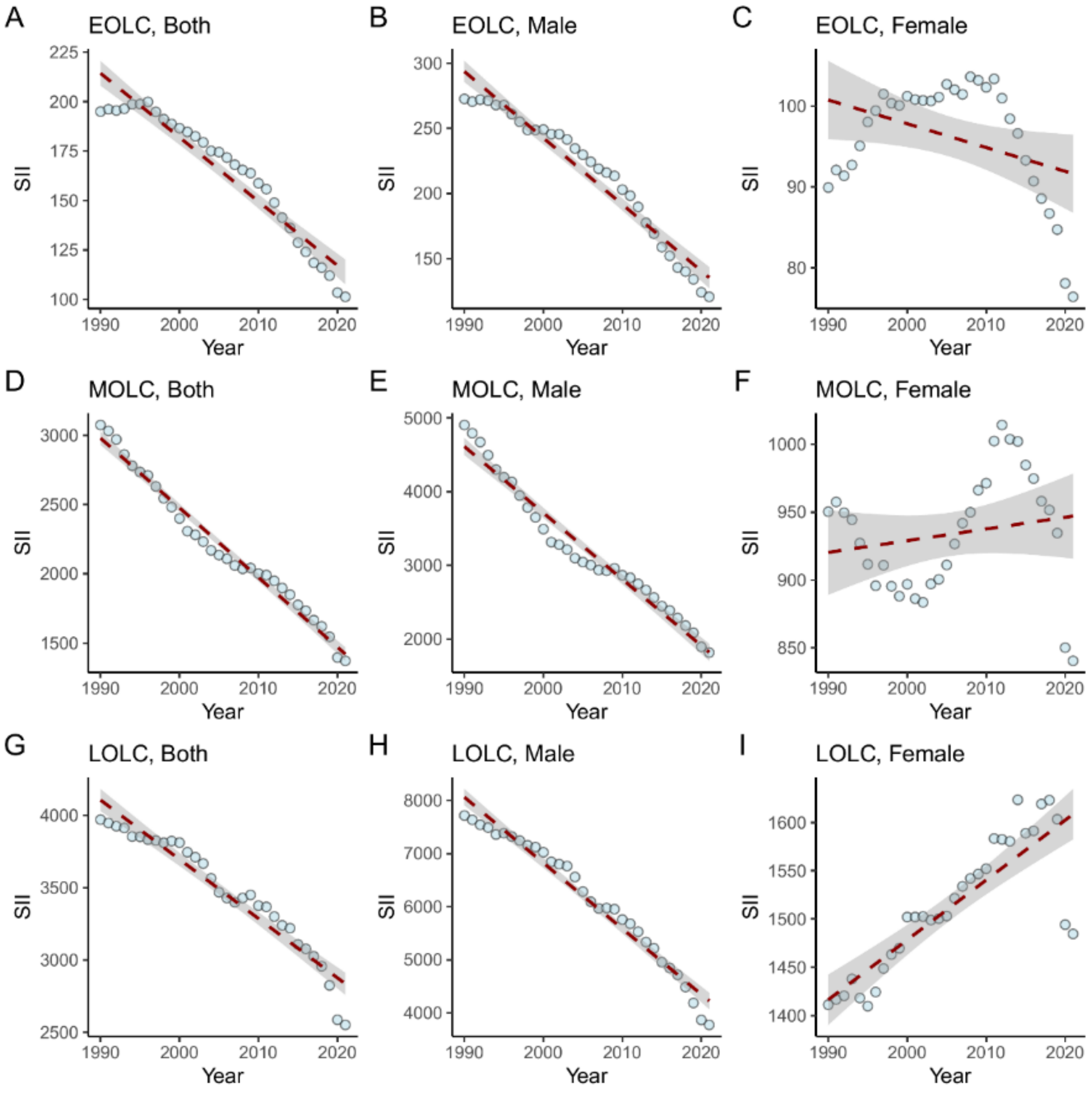

Supplementary Figure S19. Health inequality concentration curves for the DALYs of EOLC (A, B, C), MOLC (D, E, F) and LOLC (G, H, I) worldwide, in 1990 and 2021. The CI values are labeled in the bottom right corner of the figures. DALYs, disability-adjusted life-years; EOLC, early-onset lung cancer; MOLC, middle-onset lung cancer; LOLC, late-onset lung cancer; CI, concentration index.

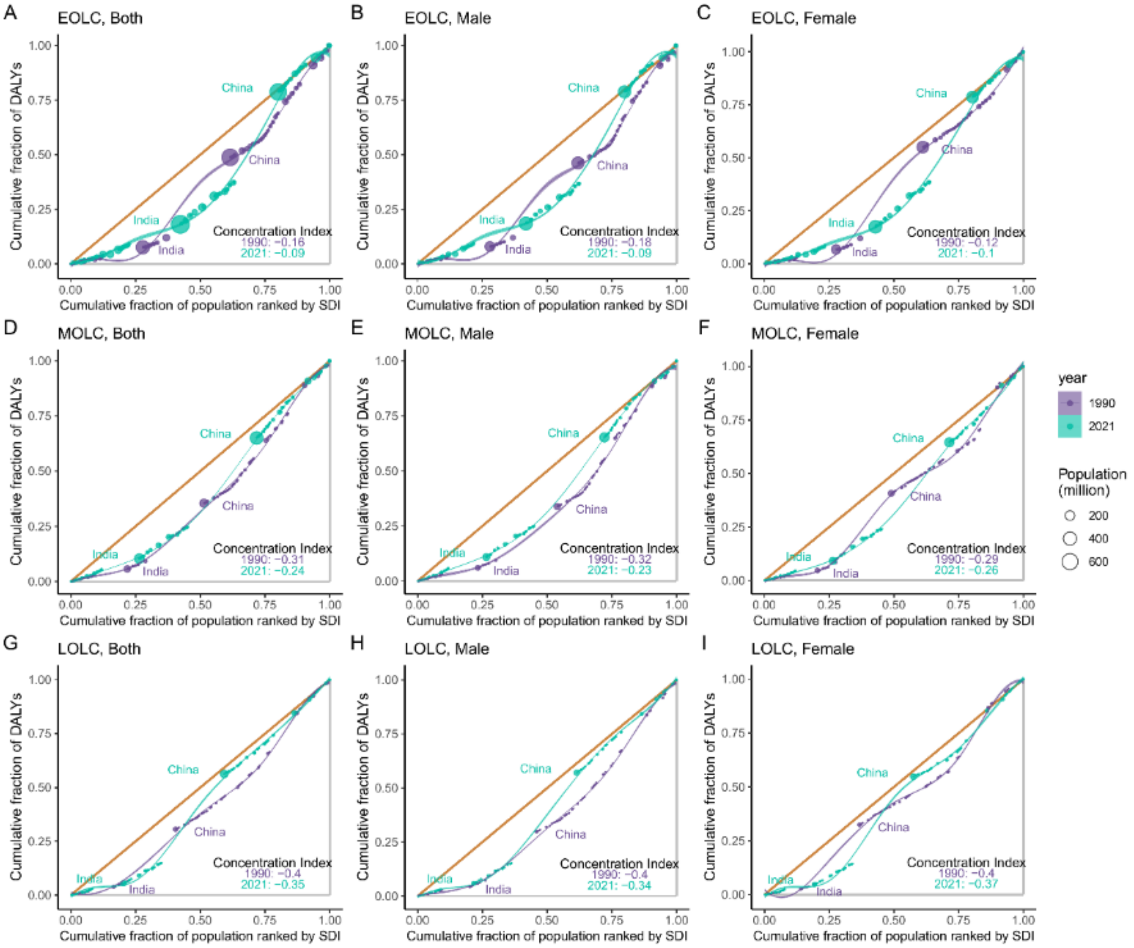

Supplementary Figure S20. Changes in incidence, mortality, and DALYs for EOLC (A, B, C), MOLC (D, E, F), and LOLC (G, H, I) attributed to aging, population growth, and epidemiological changes **in male** at the global, SDI quintile, and regional levels from 1990 to 2021. Black dots represent the cumulative contribution of all three factors to the observed changes. A positive value for any component reflects its contribution to an increase in lung cancer incidence, mortality, and DALYs, while a negative value indicates a reduction in these measures. DALYs, disability-adjusted life-years; EOLC, early-onset lung cancer; MOLC, middle-onset lung cancer; LOLC, late-onset lung cancer; SDI: Socio-demographic index.

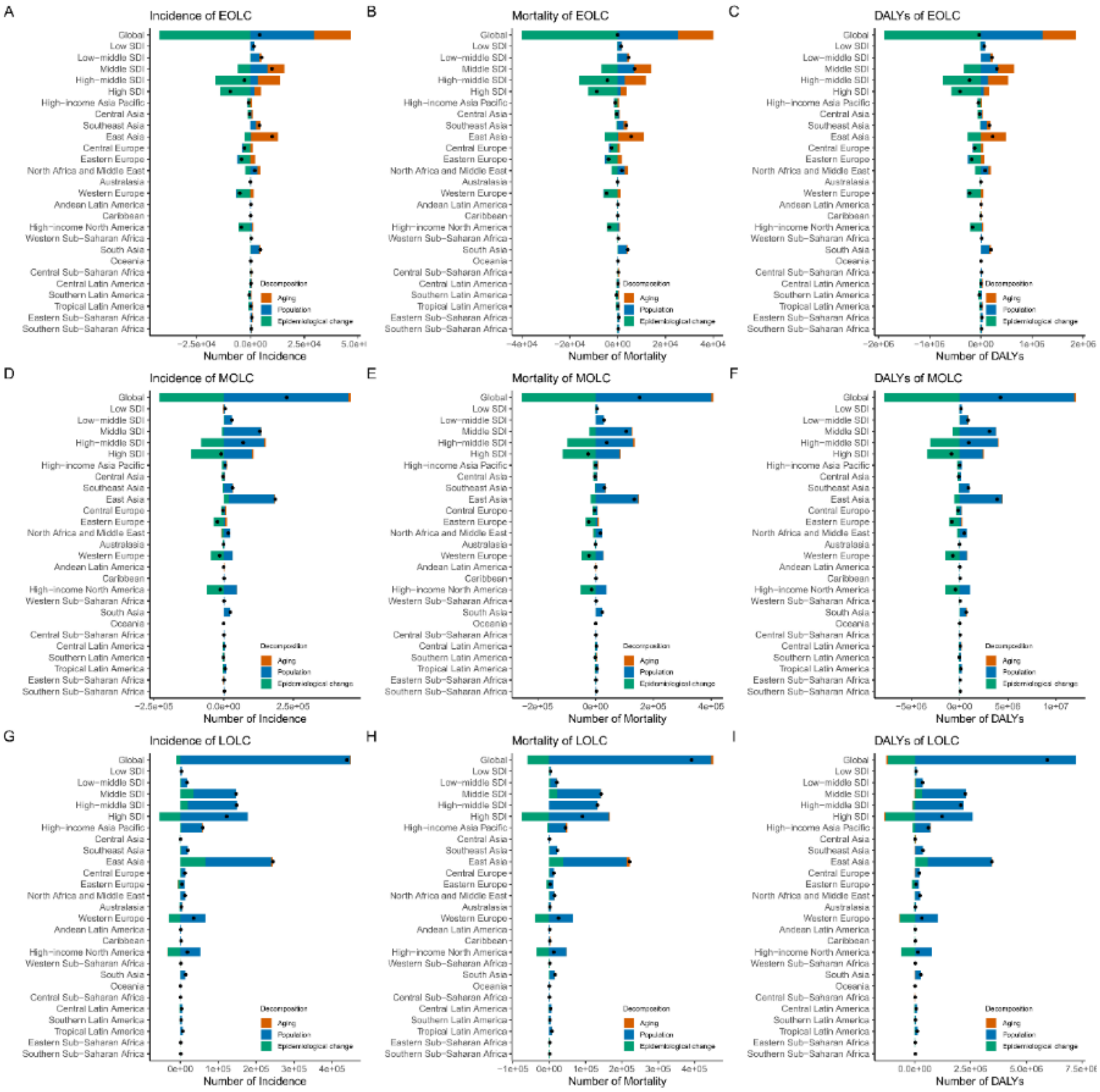

Supplementary Figure S21. Changes in incidence, mortality, and DALYs for EOLC (A, B, C), MOLC (D, E, F), and LOLC (G, H, I) attributed to aging, population growth, and epidemiological changes **in female** at the global, SDI quintile, and regional levels from 1990 to 2021. Black dots represent the cumulative contribution of all three factors to the observed changes. A positive value for any component reflects its contribution to an increase in lung cancer incidence, mortality, and DALYs, while a negative value indicates a reduction in these measures. DALYs, disability-adjusted life-years; EOLC, early-onset lung cancer; MOLC, middle-onset lung cancer; LOLC, late-onset lung cancer; SDI: Socio-demographic index.

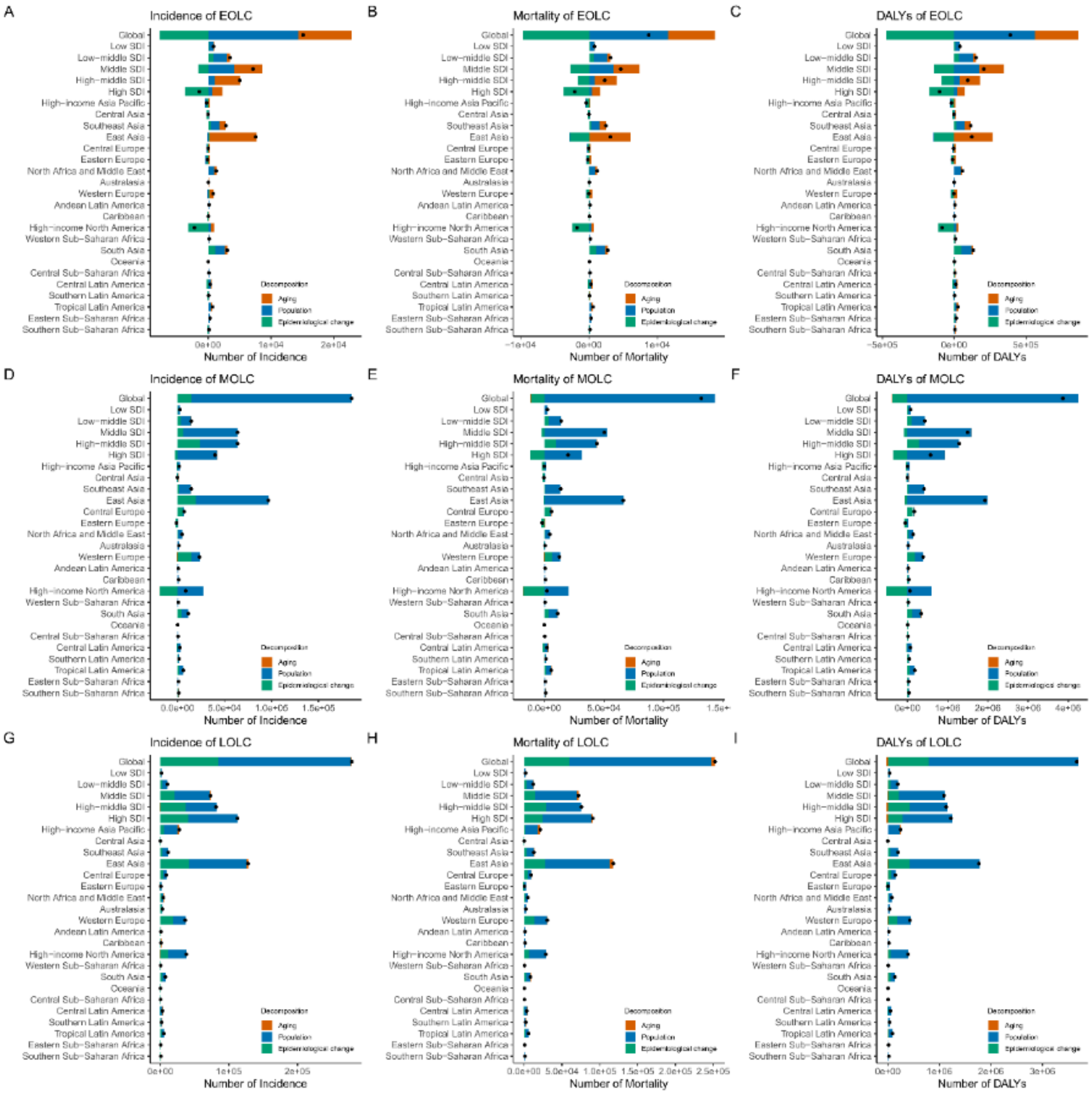

Supplementary Figure S22. Percentage contributions of major risk factors to DALYs of EOLC, MOLC and LOLC **in male** at the global, SDI quintile, and regional levels in 1990 and 2021. DALYs, disability-adjusted life-years; EOLC, early-onset lung cancer; MOLC, middle-onset lung cancer; LOLC, late-onset lung cancer; SDI: Socio-demographic index.

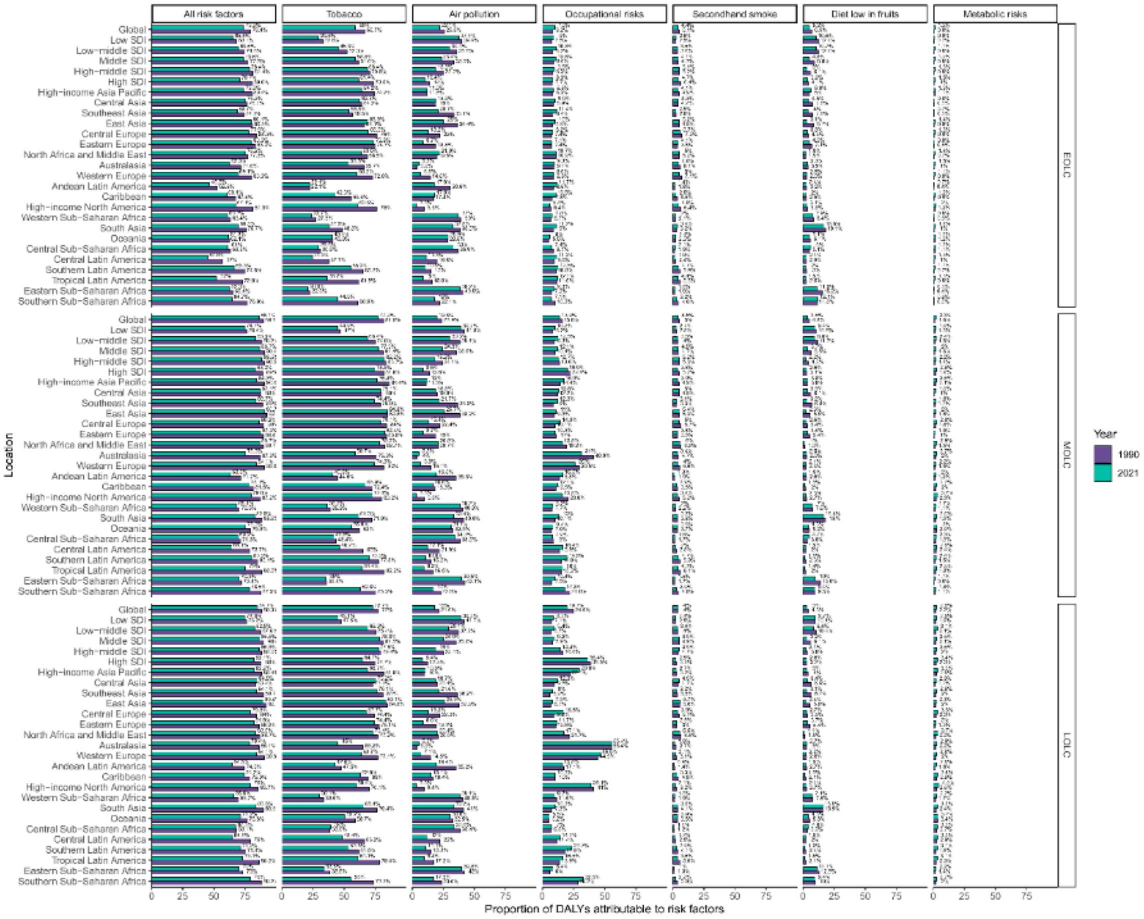

Supplementary Figure S23. Percentage contributions of major risk factors to DALYs of EOLC, MOLC and LOLC **in female** at the global, SDI quintile, and regional levels in 1990 and 2021. DALYs, disability-adjusted life-years; EOLC, early-onset lung cancer; MOLC, middle-onset lung cancer; LOLC, late-onset lung cancer; SDI: Socio-demographic index.

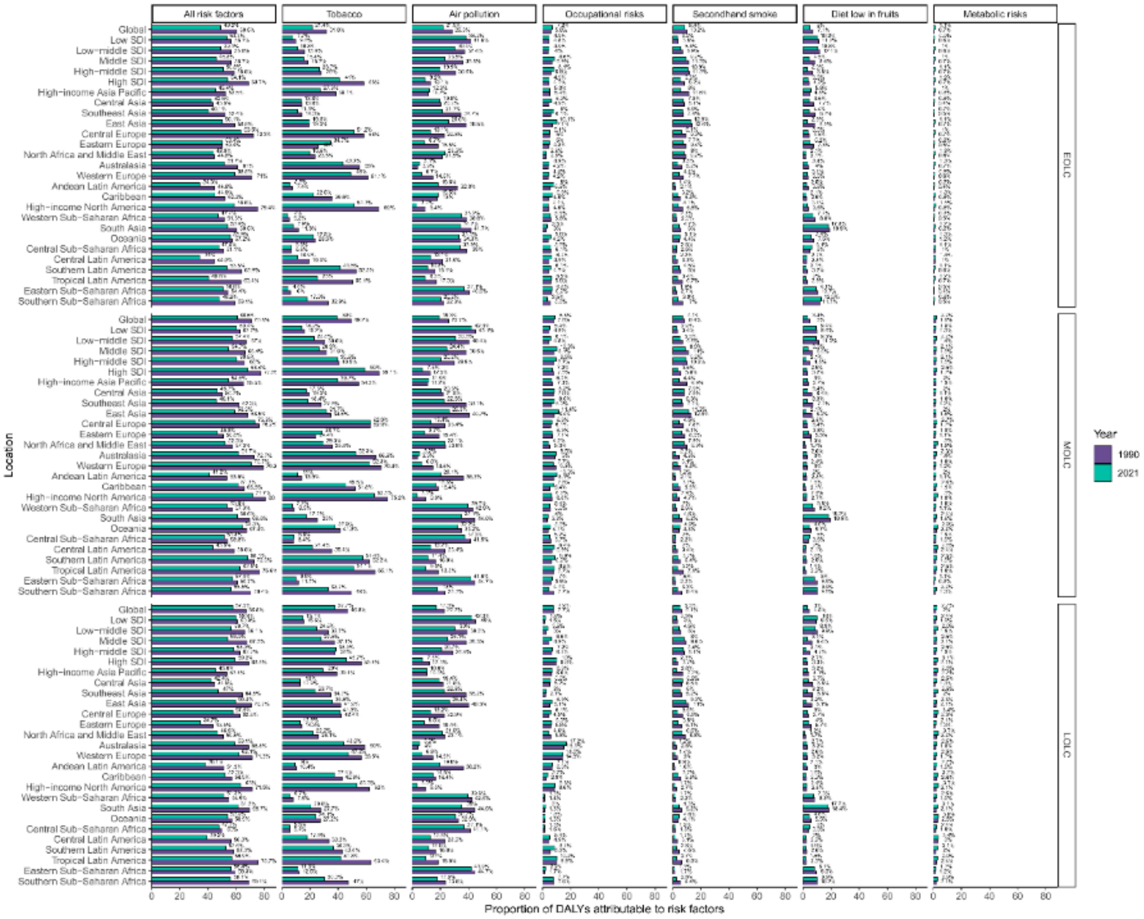

Supplementary Figure S24. BAPC model predicted trends of ASIR, ASMR and ASDR for EOLC (A, B, C), MOLC (D, E, F), and LOLC (G, H, I) **in male**: observed (1990–2021) and predicted rates (2022–2035). The blue region in the figures shows the upper and lower limits of the 95% uncertainty intervals (95% UI). BAPC, Bayesian age-period-cohort; ASIR, age-standardized incidence rate; ASMR, age-standardized mortality rate; ASDR, age-standardized disability-adjusted life year rate; EOLC, early-onset lung cancer; MOLC, middle-onset lung cancer; LOLC, late-onset lung cancer.

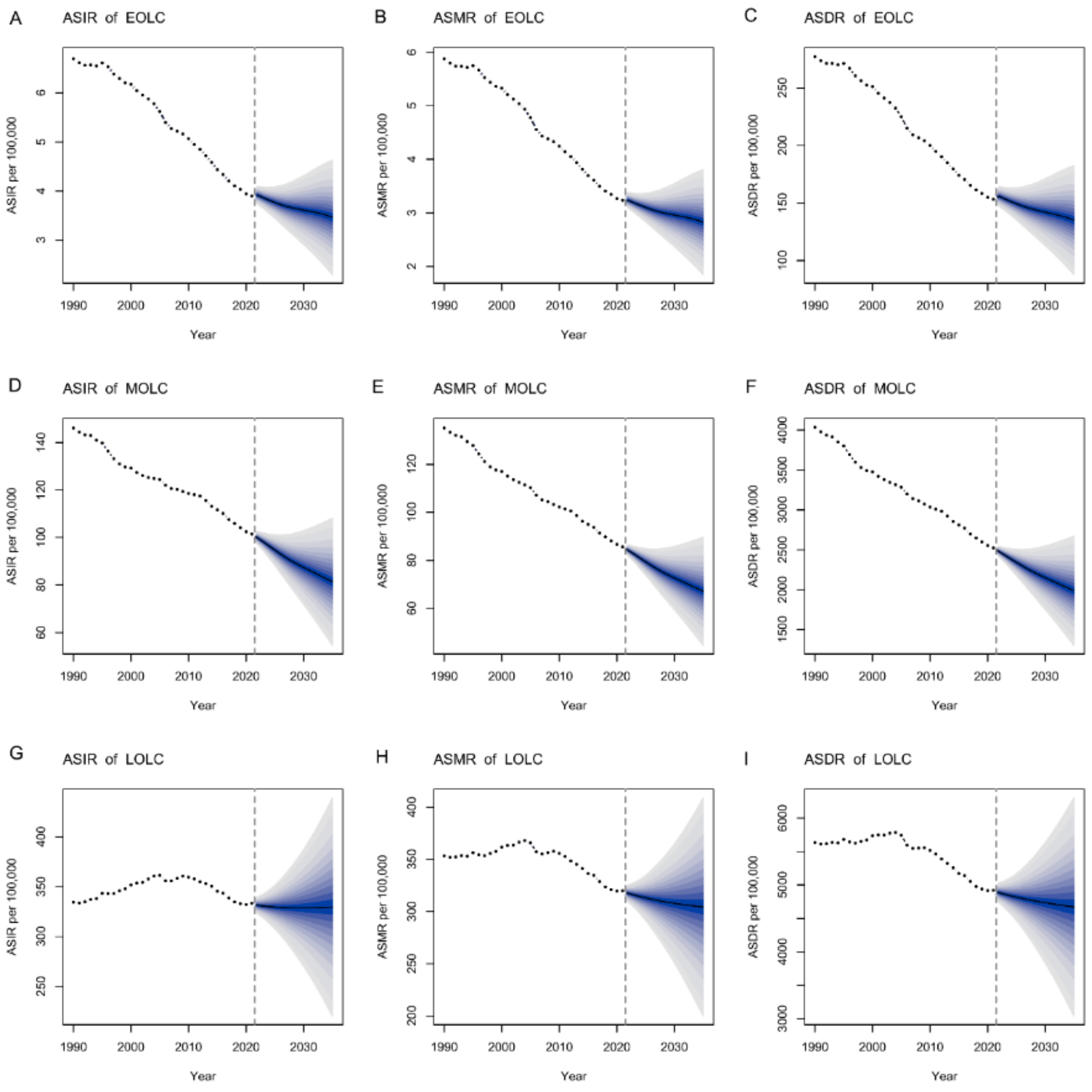

Supplementary Figure S25. BAPC model predicted trends of ASIR, ASMR and ASDR for EOLC (A, B, C), MOLC (D, E, F), and LOLC (G, H, I) **in female**: observed (1990–2021) and predicted rates (2022–2035). The blue region in the figures shows the upper and lower limits of the 95% uncertainty intervals (95% UI). BAPC, Bayesian age-period-cohort; ASIR, age-standardized incidence rate; ASMR, age-standardized mortality rate; ASDR, age-standardized disability-adjusted life year rate; EOLC, early-onset lung cancer; MOLC, middle-onset lung cancer; LOLC, late-onset lung cancer.

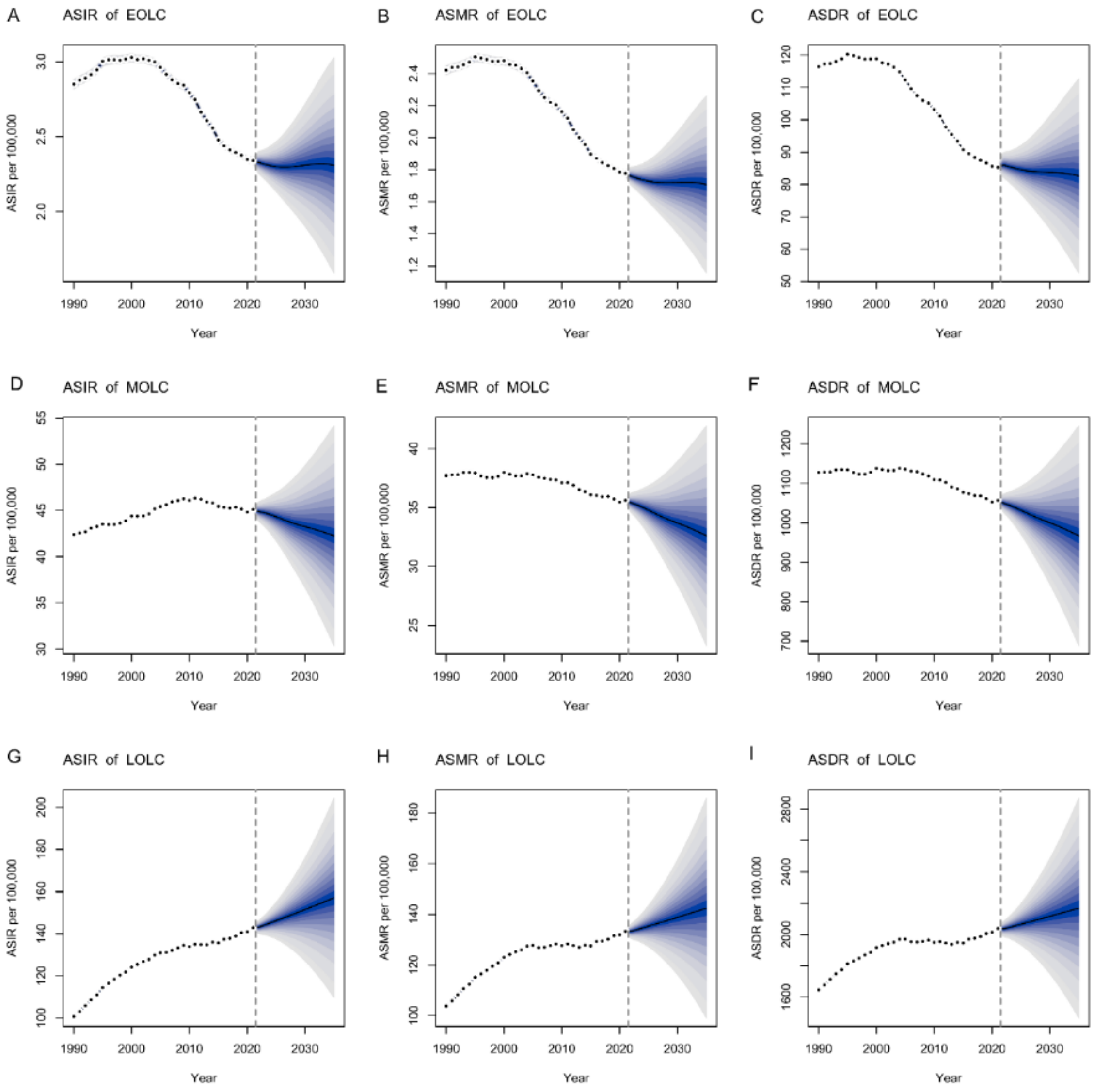

Supplementary Figure S26. ARIMA model predicted trends of ASIR, ASMR and ASDR for EOLC (A, B, C), MOLC (D, E, F), and LOLC (G, H, I): observed (1990–2021) and predicted rates (2022–2035). The blue region in the figures shows the upper and lower limits of the 95% uncertainty intervals (95% UI). ARIMA, autoregressive integrated moving average; ASIR, age-standardized incidence rate; ASMR, age-standardized mortality rate; ASDR, age-standardized disability-adjusted life year rate; EOLC, early-onset lung cancer; MOLC, middle-onset lung cancer; LOLC, late-onset lung cancer.

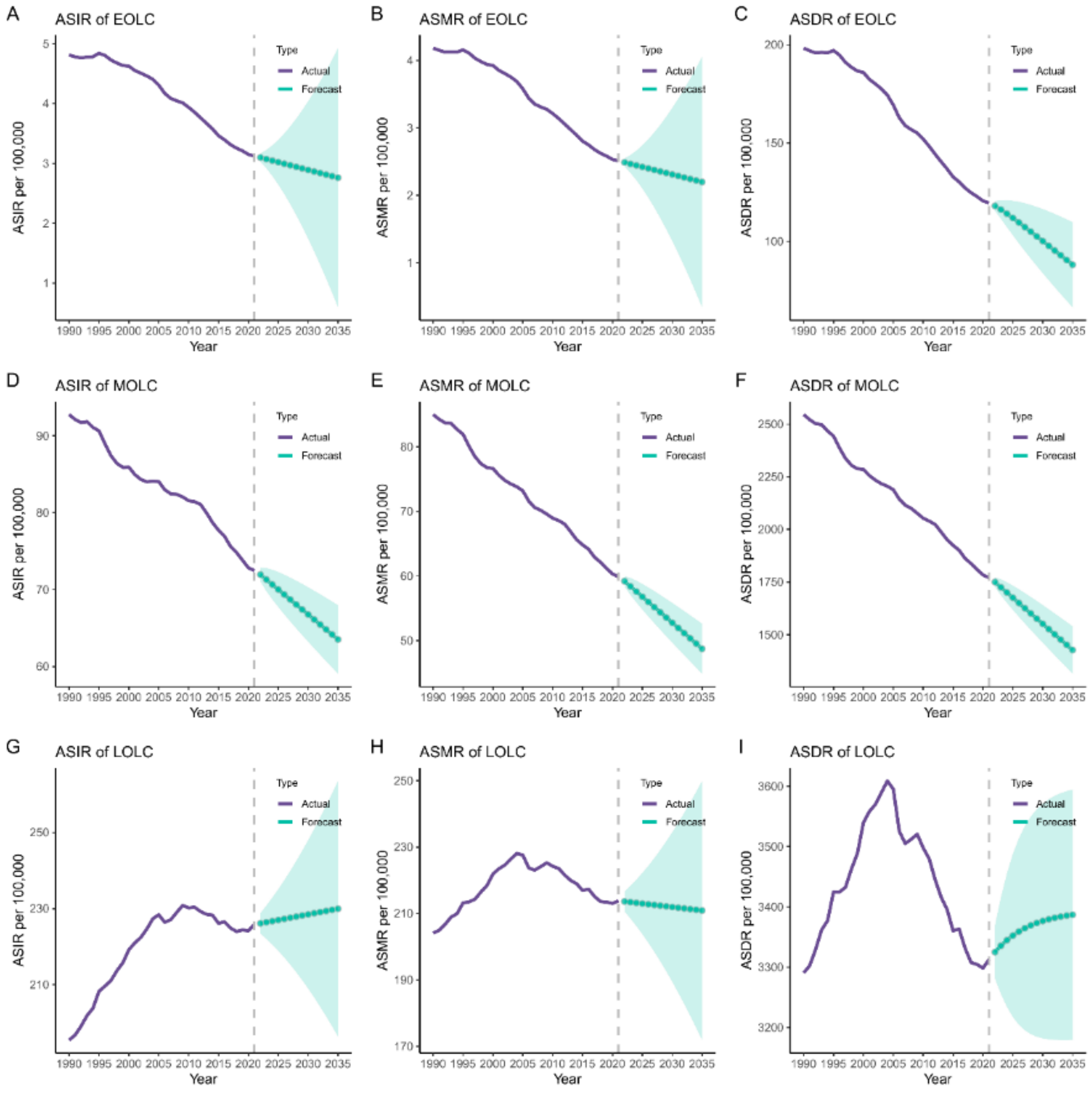

Supplementary Figure S27. ARIMA model predicted trends of ASIR, ASMR and ASDR for EOLC (A, B, C), MOLC (D, E, F), and LOLC (G, H, I) **in male**: observed (1990–2021) and predicted rates (2022–2035). The blue region in the figures shows the upper and lower limits of the 95% uncertainty intervals (95% UI). ARIMA, autoregressive integrated moving average; ASIR, age-standardized incidence rate; ASMR, age-standardized mortality rate; ASDR, age-standardized disability-adjusted life year rate; EOLC, early-onset lung cancer; MOLC, middle-onset lung cancer; LOLC, late-onset lung cancer.

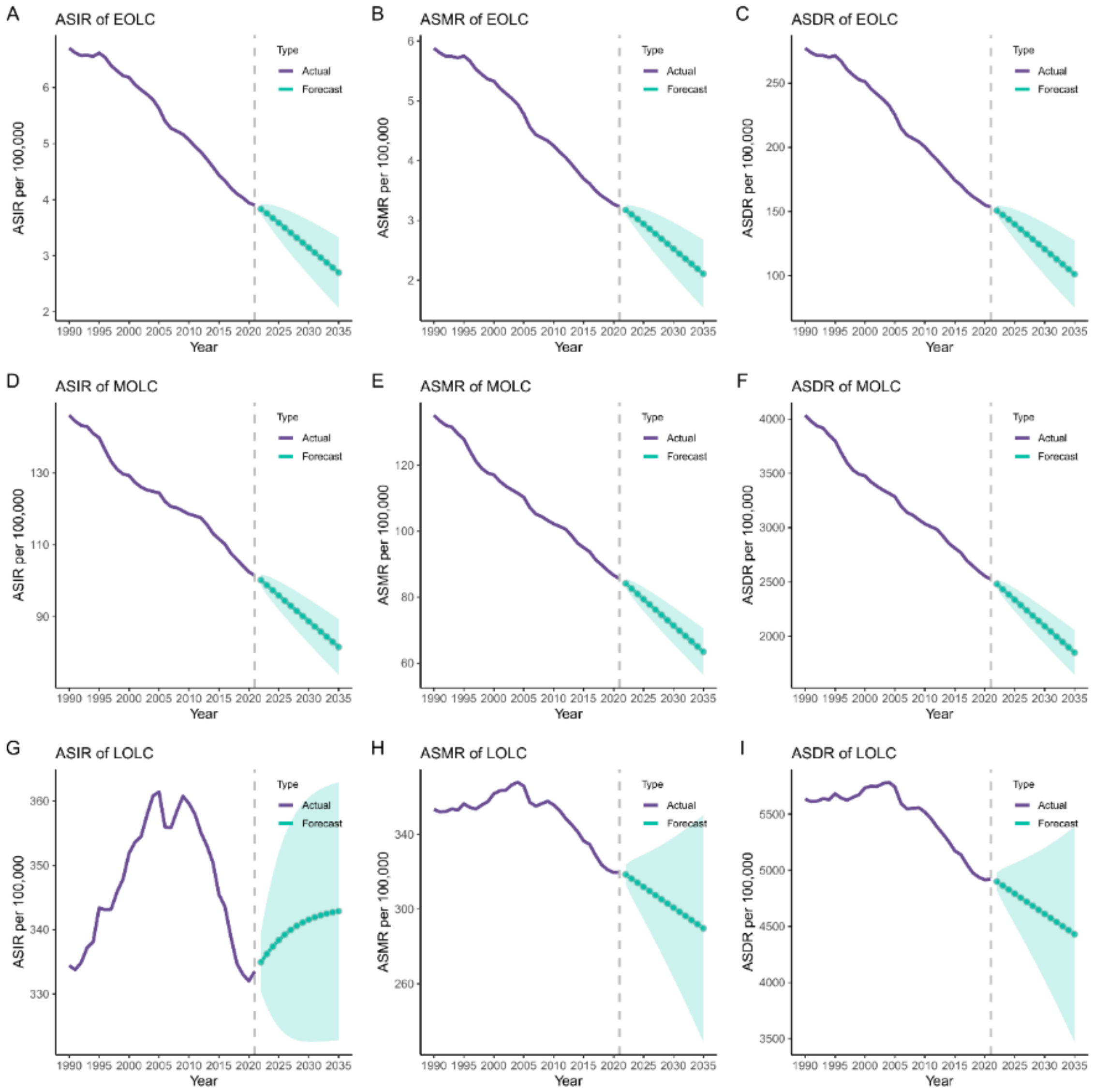

Supplementary Figure S28. ARIMA model predicted trends of ASIR, ASMR and ASDR for EOLC (A, B, C), MOLC (D, E, F), and LOLC (G, H, I) **in female**: observed (1990–2021) and predicted rates (2022–2035). The blue region in the figures shows the upper and lower limits of the 95% uncertainty intervals (95% UI). ARIMA, autoregressive integrated moving average; ASIR, age-standardized incidence rate; ASMR, age-standardized mortality rate; ASDR, age-standardized disability-adjusted life year rate; EOLC, early-onset lung cancer; MOLC, middle-onset lung cancer; LOLC, late-onset lung cancer.

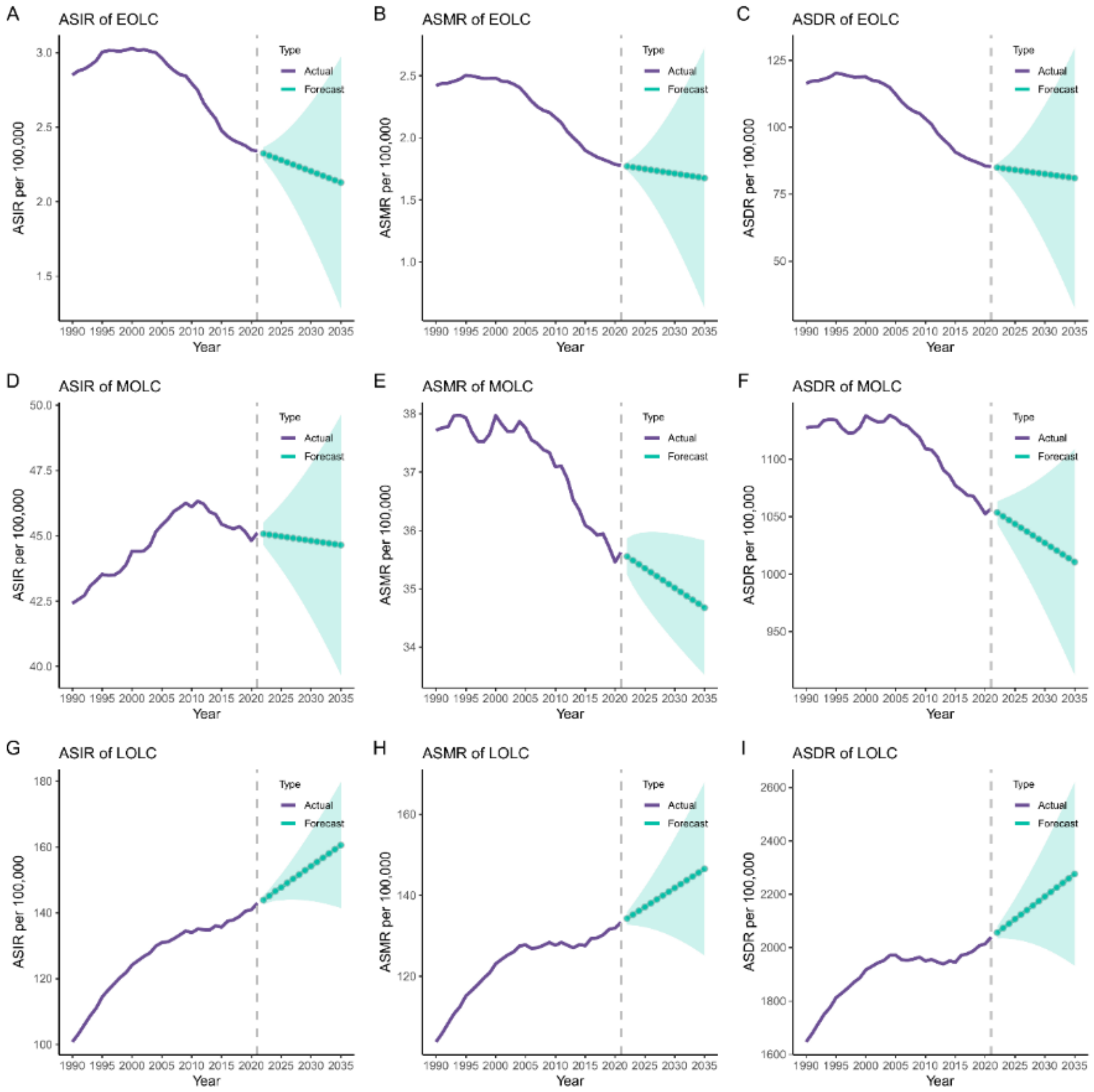

Supplement: Supplementary file 1 — Figure S1. [file CAM4-14-e70639-s002.pdf]
