# Supplementary material for: Global Trends of Early, Middle, and Late‐Onset Lung Cancer From 1990 to 2021: Results From the Global Burden of Disease Study 2021
Source: Cancer Med. 2025 Feb 7;14(3):e70639. doi: 10.1002/cam4.70639 (PMC11803626; doi:10.1002/cam4.70639)
Supplement: Supplementary file 2 — Table S1. [file CAM4-14-e70639-s001.docx]

***Supplementary Tables***

**Global Trends of Early, Middle and Late-onset Lung Cancer from 1990 to 2021: Results from the Global Burden of Disease Study 2021**

Contents

[**Supplementary Table S1.** The GBD regions and corresponding countries. 3](#_Toc181706474)

[**Supplementary Table S2.** The global populations from 1990 to 2021 from Global Burden of Disease database. 5](#_Toc181706475)

[**Supplementary Table S3.** The incidence, mortality and DALYs of EOLC, MOLC and LOLC in 1990. 22](#_Toc181706476)

[**Supplementary Table S4.** List of countries/territories with top 5 significantly increased EAPC of ASIR, ASMR and ASDR in EOLC, MOLC and LOLC, by gender, from 1990 to 2021. 31](#_Toc181706477)

[**Supplementary Table S5.** Incidence number and ASIR of EOLC, MOLC and LOLC in 1990 and 2021, and AAPC from 1990 to 2021 among 204 countries and territories. 40](#_Toc181706478)

[**Supplementary Table S6.** Mortality number and ASMR of EOLC, MOLC and LOLC in 1990 and 2021, and AAPC from 1990 to 2021 among 204 countries and territories. 88](#_Toc181706479)

[**Supplementary Table S7.** DALYs number and ASDR of EOLC, MOLC and LOLC in 1990 and 2021, and AAPC from 1990 to 2021 among 204 countries and territories. 136](#_Toc181706480)

[**Supplementary Table S8.** Frontier DALYs, and effective difference of EOLC, MOLC and LOLC in 2021, by country or territory. 189](#_Toc181706481)

[**Supplementary Table S9.** Frontier DALYs, and effective difference of EOLC, MOLC and LOLC **in male** in 2021, by country or territory. 210](#_Toc181706482)

[**Supplementary Table S10.** Frontier DALYs, and effective difference of EOLC, MOLC and LOLC **in female** in 2021, by country or territory. 230](#_Toc181706483)

[**Supplementary Table S11.** Summary measures for SII values in DALYs of EOLC, MOLC and LOLC from 1990 to 2021. 250](#_Toc181706484)

[**Supplementary Table S12.** Summary measures for concentration index values in DALYs of EOLC, MOLC and LOLC from 1990 to 2021. 259](#_Toc181706485)

[**Supplementary Table S13.** Changes in Incidence, mortality and DALYs number of EOLC, MOLC and LOLC according to population-level determinants from 1990 to 2021. 268](#_Toc181706486)

[**Supplementary Table S14.** Changes in Incidence, mortality and DALYs number of EOLC, MOLC and LOLC **in male** according to population-level determinants from 1990 to 2021. 284](#_Toc181706487)

[**Supplementary Table S15.** Changes in Incidence, mortality and DALYs number of EOLC, MOLC and LOLC **in female** according to population-level determinants from 1990 to 2021. 301](#_Toc181706488)

[**Supplementary Table S16.** BAPC model predicted trends of ASIR, ASMR and ASDR for EOLC, MOLC and LOLC from 2022 to 2035. 319](#_Toc181706489)

[**Supplementary Table S17.** ARIMA model predicted trends of ASIR, ASMR and ASDR for EOLC, MOLC and LOLC from 2022 to 2035. 323](#_Toc181706490)

# **Supplementary Table S1.** The GBD regions and corresponding countries.

| GBD regions | Countries |
| --- | --- |
| High-income Asia Pacific | Brunei Darussalam, Japan, Republic of Korea, Singapore |
| High-income North America | Canada, United States of America |
| Western Europe | Andorra, Austria, Belgium, Cyprus, Denmark, Finland, France, Germany, Greece, Guernsey, Iceland, Ireland, Isle of Man, Israel, Italy, Jersey, Liechtenstein, Luxembourg, Malta, Monaco, Netherlands, Norway, Portugal, San Marino, Spain, Sweden, Switzerland, United Kingdom, Vatican City |
| Australasia | Australia, New Zealand |
| Andean Latin America | Bolivia, Ecuador, Peru |
| Tropical Latin America | Brazil, Paraguay |
| Central Latin America | Colombia, Costa Rica, El Salvador, Guatemala, Honduras, Mexico, Nicaragua, Panama, Venezuela (Bolivarian Republic of) |
| Southern Latin America | Argentina, Chile, Uruguay |
| Caribbean | Antigua and Barbuda, Bahamas, Barbados, Belize, Cuba, Dominica, Dominican Republic, Grenada, Guyana, Haiti, Jamaica, Saint, Kitts and Nevis, Saint Lucia, Saint Vincent and the Grenadines, Suriname, Trinidad and Tobago |
| Central Europe | Albania, Bosnia and Herzegovina, Bulgaria, Croatia, Czechia, Hungary, Kosovo, Montenegro, North Macedonia, Poland, Romania, Serbia, Slovakia, Slovenia |
| Eastern Europe | Belarus, Estonia, Latvia, Lithuania, Republic of Moldova, Russian Federation, Ukraine |
| Central Asia | Armenia, Azerbaijan, Georgia, Kazakhstan, Kyrgyzstan, Mongolia, Tajikistan, Turkmenistan, Uzbekistan |
| North Africa and Middle East | Afghanistan, Algeria, Bahrain, Egypt, Iran (Islamic Republic of), Iraq, Jordan, Kuwait, Lebanon, Libya, Morocco, Oman, Palestine, Qatar, Saudi Arabia, Syrian Arab Republic, Tunisia, Turkiye, United Arab Emirates, Western Sahara, Yemen |
| South Asia | Bangladesh, Bhutan, India, Nepal, Pakistan |
| Southeast Asia | Cambodia, Indonesia, Lao People's Democratic Republic, Malaysia, Maldives, Mauritius, Myanmar, Philippines, Seychelles, Sri Lanka, Thailand, Timor-Leste, Viet Nam |
| East Asia | China, Democratic People's Republic of Korea, Taiwan (Province of China) |
| Oceania | Fiji, Kiribati, Marshall Islands, Micronesia (Federated States of), Nauru, Niue, Palau, Papua New Guinea, Samoa, Solomon, Islands, Tonga, Tuvalu, Vanuatu |
| Western Sub-Saharan Africa | Benin, Burkina Faso, Cabo Verde, Cameroon, Chad, Cote d'Ivoire, Gambia, Ghana, Guinea, Guinea-Bissau, Liberia, Mali, Mauritania, Niger, Nigeria, Sao Tome and Principe, Senegal, Sierra Leone, Togo |
| Eastern Sub-Saharan Africa | Burundi, Comoros, Djibouti, Eritrea, Ethiopia, Kenya, Madagascar, Malawi, Mozambique, Rwanda, Somalia, South Sudan, Sudan, Uganda, United Republic of Tanzania, Zambia |
| Central Sub-Saharan Africa | Angola, Central African Republic, Congo, Democratic Republic of the Congo, Equatorial Guinea, Gabon |
| Southern Sub-Saharan Africa | Botswana, Eswatini, Lesotho, Namibia, South Africa, Zimbabwe |

Abbreviations: GBD, global burden of disease.

# **Supplementary Table S2.** The global populations from 1990 to 2021 from Global Burden of Disease database.

| Age group | Year | Population (Number) | | |
| --- | --- | --- | --- | --- |
|  |  | Value | 95% UI lower | 95% UI upper |
| 15-19 years | 1990 | 519423451 | 508862515 | 530724737 |
| 20-24 years | 1990 | 492087905 | 481525127 | 503481056 |
| 25-29 years | 1990 | 442619653 | 433620464 | 452439505 |
| 30-34 years | 1990 | 385422487 | 377887066 | 393550872 |
| 35-39 years | 1990 | 352244471 | 344922797 | 360366501 |
| 40-44 years | 1990 | 286481635 | 280859543 | 292686275 |
| 45-49 years | 1990 | 232195492 | 227724763 | 237040519 |
| 50-54 years | 1990 | 212571467 | 208564893 | 216971774 |
| 55-59 years | 1990 | 185199959 | 181629801 | 189173190 |
| 60-64 years | 1990 | 160609237 | 157624140 | 163858800 |
| 65-69 years | 1990 | 123609457 | 121382571 | 126044770 |
| 70-74 years | 1990 | 84661155 | 83100754 | 86320731 |
| 75-79 years | 1990 | 61555514 | 60548489 | 62631090 |
| 80-84 years | 1990 | 35375927 | 34810482 | 35960020 |
| 85-89 years | 1990 | 15111073 | 14883743 | 15349648 |
| 90-94 years | 1990 | 4285188 | 4219089 | 4355023 |
| 95+ years | 1990 | 1018087 | 998973 | 1037516 |
| Total | 1990 | 3594472157 | 3523165210 | 3671992027 |
| 15-19 years | 1991 | 518350547 | 507189731 | 529910182 |
| 20-24 years | 1991 | 497020860 | 485584816 | 508801469 |
| 25-29 years | 1991 | 455741897 | 445400937 | 466507034 |
| 30-34 years | 1991 | 390561332 | 382497817 | 398952043 |
| 35-39 years | 1991 | 361091531 | 352793846 | 369733259 |
| 40-44 years | 1991 | 300529642 | 294135129 | 307488997 |
| 45-49 years | 1991 | 235388282 | 230501236 | 240625148 |
| 50-54 years | 1991 | 214880957 | 210531087 | 219629696 |
| 55-59 years | 1991 | 187841080 | 183948021 | 192068061 |
| 60-64 years | 1991 | 163914797 | 160693832 | 167542064 |
| 65-69 years | 1991 | 127429488 | 124929976 | 130118159 |
| 70-74 years | 1991 | 87729312 | 85983162 | 89539262 |
| 75-79 years | 1991 | 61771393 | 60654585 | 62940576 |
| 80-84 years | 1991 | 36491447 | 35880541 | 37140479 |
| 85-89 years | 1991 | 15805921 | 15554743 | 16073719 |
| 90-94 years | 1991 | 4576829 | 4500407 | 4656054 |
| 95+ years | 1991 | 1059748 | 1038936 | 1081745 |
| Total | 1991 | 3660185062 | 3581818803 | 3742807946 |
| 15-19 years | 1992 | 517295515 | 506008568 | 529311205 |
| 20-24 years | 1992 | 500647331 | 488969303 | 513229096 |
| 25-29 years | 1992 | 466919881 | 455683989 | 478916634 |
| 30-34 years | 1992 | 398915095 | 390235361 | 408034140 |
| 35-39 years | 1992 | 368394794 | 359598031 | 377750856 |
| 40-44 years | 1992 | 310707131 | 303560750 | 318463642 |
| 45-49 years | 1992 | 243591565 | 238219094 | 249511898 |
| 50-54 years | 1992 | 216306483 | 211736303 | 221412214 |
| 55-59 years | 1992 | 191103916 | 186904580 | 195773018 |
| 60-64 years | 1992 | 166298016 | 162810708 | 170264312 |
| 65-69 years | 1992 | 131286171 | 128612876 | 134289703 |
| 70-74 years | 1992 | 91404862 | 89503927 | 93433926 |
| 75-79 years | 1992 | 61751840 | 60541661 | 63043811 |
| 80-84 years | 1992 | 37507009 | 36815648 | 38211330 |
| 85-89 years | 1992 | 16564525 | 16275524 | 16865238 |
| 90-94 years | 1992 | 4843764 | 4752904 | 4933591 |
| 95+ years | 1992 | 1108169 | 1083917 | 1131703 |
| Total | 1992 | 3724646067 | 3641313143 | 3814576316 |
| 15-19 years | 1993 | 517308029 | 505373622 | 529613219 |
| 20-24 years | 1993 | 503144509 | 490689284 | 516244742 |
| 25-29 years | 1993 | 474777318 | 462285231 | 487916391 |
| 30-34 years | 1993 | 411338279 | 401597542 | 421472814 |
| 35-39 years | 1993 | 373309814 | 364114885 | 383132152 |
| 40-44 years | 1993 | 321865700 | 313915031 | 330432662 |
| 45-49 years | 1993 | 252553515 | 246595663 | 259005354 |
| 50-54 years | 1993 | 217241047 | 212354246 | 222573886 |
| 55-59 years | 1993 | 194246045 | 189769386 | 199172100 |
| 60-64 years | 1993 | 168255823 | 164410234 | 172466573 |
| 65-69 years | 1993 | 135141884 | 132175521 | 138437337 |
| 70-74 years | 1993 | 95390014 | 93315810 | 97571736 |
| 75-79 years | 1993 | 61662095 | 60314452 | 63051279 |
| 80-84 years | 1993 | 38570727 | 37805711 | 39344384 |
| 85-89 years | 1993 | 17209646 | 16885131 | 17541350 |
| 90-94 years | 1993 | 5154551 | 5051005 | 5256816 |
| 95+ years | 1993 | 1156853 | 1129038 | 1182924 |
| Total | 1993 | 3788325848 | 3697781790 | 3884415720 |
| 15-19 years | 1994 | 519239610 | 506764103 | 532451288 |
| 20-24 years | 1994 | 504292345 | 491026830 | 518401301 |
| 25-29 years | 1994 | 480314260 | 467027254 | 494816772 |
| 30-34 years | 1994 | 425652299 | 414633735 | 437634256 |
| 35-39 years | 1994 | 376473676 | 366777842 | 386916275 |
| 40-44 years | 1994 | 330818898 | 322078983 | 340555069 |
| 45-49 years | 1994 | 265230770 | 258489817 | 272738065 |
| 50-54 years | 1994 | 218149042 | 212792027 | 224038567 |
| 55-59 years | 1994 | 197003771 | 192097662 | 202463297 |
| 60-64 years | 1994 | 169914554 | 165703505 | 174579441 |
| 65-69 years | 1994 | 138844403 | 135525580 | 142554869 |
| 70-74 years | 1994 | 99207618 | 96973337 | 101692482 |
| 75-79 years | 1994 | 62205838 | 60754875 | 63751806 |
| 80-84 years | 1994 | 39445602 | 38618544 | 40285717 |
| 85-89 years | 1994 | 17839978 | 17493742 | 18197320 |
| 90-94 years | 1994 | 5473774 | 5363061 | 5585602 |
| 95+ years | 1994 | 1211025 | 1182826 | 1239013 |
| Total | 1994 | 3851317461 | 3753303723 | 3957901140 |
| 15-19 years | 1995 | 523594826 | 510425708 | 537543655 |
| 20-24 years | 1995 | 504445556 | 490577194 | 519370302 |
| 25-29 years | 1995 | 484649609 | 470140528 | 499919144 |
| 30-34 years | 1995 | 439746808 | 427182729 | 452990108 |
| 35-39 years | 1995 | 379502375 | 369512990 | 390260370 |
| 40-44 years | 1995 | 343378568 | 333482648 | 354166356 |
| 45-49 years | 1995 | 275592318 | 268225984 | 283727139 |
| 50-54 years | 1995 | 218767487 | 213017686 | 225013055 |
| 55-59 years | 1995 | 199545803 | 194343682 | 205356720 |
| 60-64 years | 1995 | 171790716 | 167180783 | 176842827 |
| 65-69 years | 1995 | 142008434 | 138463035 | 145982780 |
| 70-74 years | 1995 | 102693526 | 100177341 | 105482985 |
| 75-79 years | 1995 | 63806210 | 62230097 | 65512919 |
| 80-84 years | 1995 | 40080289 | 39186502 | 41028067 |
| 85-89 years | 1995 | 18423094 | 18044943 | 18825206 |
| 90-94 years | 1995 | 5787521 | 5662818 | 5915499 |
| 95+ years | 1995 | 1280261 | 1246680 | 1313014 |
| Total | 1995 | 3915093403 | 3809101349 | 4029250147 |
| 15-19 years | 1996 | 529823063 | 516617676 | 543678169 |
| 20-24 years | 1996 | 503870796 | 490437890 | 518269219 |
| 25-29 years | 1996 | 489097095 | 475199361 | 504119283 |
| 30-34 years | 1996 | 452012934 | 439166948 | 465905155 |
| 35-39 years | 1996 | 384584619 | 374644207 | 395276608 |
| 40-44 years | 1996 | 351109627 | 341272916 | 361957513 |
| 45-49 years | 1996 | 288704234 | 281210817 | 297213109 |
| 50-54 years | 1996 | 221628602 | 215857326 | 227945441 |
| 55-59 years | 1996 | 201345881 | 196209508 | 206989520 |
| 60-64 years | 1996 | 174300190 | 169764119 | 179355148 |
| 65-69 years | 1996 | 144863890 | 141284168 | 148918034 |
| 70-74 years | 1996 | 105970261 | 103479499 | 108787531 |
| 75-79 years | 1996 | 66328465 | 64746098 | 68008287 |
| 80-84 years | 1996 | 40266498 | 39377484 | 41204867 |
| 85-89 years | 1996 | 19043761 | 18660647 | 19449835 |
| 90-94 years | 1996 | 6081446 | 5954460 | 6212572 |
| 95+ years | 1996 | 1369146 | 1335871 | 1402078 |
| Total | 1996 | 3980400511 | 3875218995 | 4094692370 |
| 15-19 years | 1997 | 537487841 | 524652096 | 550830659 |
| 20-24 years | 1997 | 503203357 | 490339633 | 516213063 |
| 25-29 years | 1997 | 492738284 | 479154026 | 506770274 |
| 30-34 years | 1997 | 462274858 | 449249184 | 475632340 |
| 35-39 years | 1997 | 393026683 | 383099398 | 403289637 |
| 40-44 years | 1997 | 357515200 | 347812285 | 367586051 |
| 45-49 years | 1997 | 298073400 | 290352767 | 306393044 |
| 50-54 years | 1997 | 229578011 | 223828468 | 235797719 |
| 55-59 years | 1997 | 202410909 | 197516062 | 207738724 |
| 60-64 years | 1997 | 177507785 | 173142139 | 182291678 |
| 65-69 years | 1997 | 146974994 | 143448099 | 150801759 |
| 70-74 years | 1997 | 109343979 | 106855699 | 112011878 |
| 75-79 years | 1997 | 69352393 | 67784819 | 70994717 |
| 80-84 years | 1997 | 40346262 | 39475990 | 41248649 |
| 85-89 years | 1997 | 19652552 | 19267498 | 20046434 |
| 90-94 years | 1997 | 6407794 | 6281542 | 6534378 |
| 95+ years | 1997 | 1452953 | 1421339 | 1483665 |
| Total | 1997 | 4047347253 | 3943681043 | 4155664670 |
| 15-19 years | 1998 | 546654361 | 534565205 | 559397662 |
| 20-24 years | 1998 | 503629787 | 492279346 | 515504314 |
| 25-29 years | 1998 | 495721140 | 483603952 | 508583029 |
| 30-34 years | 1998 | 469431359 | 457293629 | 482295460 |
| 35-39 years | 1998 | 405580835 | 396050006 | 415605833 |
| 40-44 years | 1998 | 361849185 | 353094089 | 371146030 |
| 45-49 years | 1998 | 308498974 | 301063174 | 316590346 |
| 50-54 years | 1998 | 238307902 | 232837272 | 244260616 |
| 55-59 years | 1998 | 203196414 | 198703992 | 208186000 |
| 60-64 years | 1998 | 180680519 | 176615459 | 185192619 |
| 65-69 years | 1998 | 148794512 | 145529283 | 152445490 |
| 70-74 years | 1998 | 112769851 | 110405798 | 115371489 |
| 75-79 years | 1998 | 72638798 | 71146198 | 74191686 |
| 80-84 years | 1998 | 40453043 | 39613977 | 41317185 |
| 85-89 years | 1998 | 20319783 | 19944412 | 20710808 |
| 90-94 years | 1998 | 6705581 | 6582711 | 6830309 |
| 95+ years | 1998 | 1553609 | 1522128 | 1584875 |
| Total | 1998 | 4116785656 | 4020850631 | 4219213750 |
| 15-19 years | 1999 | 557122922 | 545570042 | 568799310 |
| 20-24 years | 1999 | 506173067 | 495767266 | 516794310 |
| 25-29 years | 1999 | 497771482 | 486771181 | 509518816 |
| 30-34 years | 1999 | 474679669 | 463487336 | 486448503 |
| 35-39 years | 1999 | 420076030 | 410827146 | 429781848 |
| 40-44 years | 1999 | 364779394 | 356846348 | 372995010 |
| 45-49 years | 1999 | 316890103 | 309801201 | 324499155 |
| 50-54 years | 1999 | 250781032 | 245443234 | 256642810 |
| 55-59 years | 1999 | 204165021 | 199987343 | 208758628 |
| 60-64 years | 1999 | 183577741 | 179805941 | 187765471 |
| 65-69 years | 1999 | 150503841 | 147430622 | 153919191 |
| 70-74 years | 1999 | 116133668 | 113851823 | 118645531 |
| 75-79 years | 1999 | 75825393 | 74400008 | 77342846 |
| 80-84 years | 1999 | 41041381 | 40254828 | 41867835 |
| 85-89 years | 1999 | 20914632 | 20559677 | 21293102 |
| 90-94 years | 1999 | 7004236 | 6888920 | 7125543 |
| 95+ years | 1999 | 1662535 | 1631502 | 1693057 |
| Total | 1999 | 4189102146 | 4099324417 | 4283890965 |
| 15-19 years | 2000 | 568797032 | 557170413 | 580476365 |
| 20-24 years | 2000 | 511025170 | 500766365 | 521207390 |
| 25-29 years | 2000 | 498746842 | 488164028 | 509814410 |
| 30-34 years | 2000 | 479100562 | 468249676 | 490367439 |
| 35-39 years | 2000 | 434357127 | 424938577 | 444097158 |
| 40-44 years | 2000 | 367832477 | 360325987 | 375675329 |
| 45-49 years | 2000 | 328965816 | 321835140 | 336495896 |
| 50-54 years | 2000 | 261120565 | 255856537 | 266846595 |
| 55-59 years | 2000 | 205047564 | 200946368 | 209413764 |
| 60-64 years | 2000 | 186331085 | 182675789 | 190280418 |
| 65-69 years | 2000 | 152556164 | 149567504 | 155823223 |
| 70-74 years | 2000 | 119117958 | 116896682 | 121574410 |
| 75-79 years | 2000 | 78780507 | 77370619 | 80233190 |
| 80-84 years | 2000 | 42441236 | 41672963 | 43244765 |
| 85-89 years | 2000 | 21393632 | 21042042 | 21751973 |
| 90-94 years | 2000 | 7289776 | 7176151 | 7406446 |
| 95+ years | 2000 | 1783051 | 1752444 | 1813106 |
| Total | 2000 | 4264686564 | 4176407284 | 4356521877 |
| 15-19 years | 2001 | 581845640 | 570270099 | 593833700 |
| 20-24 years | 2001 | 517477441 | 507430828 | 527746600 |
| 25-29 years | 2001 | 498690155 | 488338312 | 509408429 |
| 30-34 years | 2001 | 484048275 | 473201429 | 495596794 |
| 35-39 years | 2001 | 446650187 | 436717707 | 457148650 |
| 40-44 years | 2001 | 373029737 | 365569893 | 380893094 |
| 45-49 years | 2001 | 336668739 | 329282097 | 344534358 |
| 50-54 years | 2001 | 274031668 | 268317592 | 280307128 |
| 55-59 years | 2001 | 208212653 | 204012588 | 212812235 |
| 60-64 years | 2001 | 188427282 | 184817543 | 192545933 |
| 65-69 years | 2001 | 155246816 | 152172327 | 158699804 |
| 70-74 years | 2001 | 121875275 | 119604864 | 124435790 |
| 75-79 years | 2001 | 81571515 | 80100085 | 83140583 |
| 80-84 years | 2001 | 44470745 | 43665899 | 45327212 |
| 85-89 years | 2001 | 21642463 | 21263333 | 22021839 |
| 90-94 years | 2001 | 7587695 | 7466300 | 7713990 |
| 95+ years | 2001 | 1908775 | 1876311 | 1941490 |
| Total | 2001 | 4343385062 | 4254107208 | 4438107628 |
| 15-19 years | 2002 | 594977658 | 582120632 | 608302551 |
| 20-24 years | 2002 | 525370560 | 514628456 | 536545743 |
| 25-29 years | 2002 | 498458561 | 487439771 | 509992643 |
| 30-34 years | 2002 | 488475654 | 476557097 | 501183989 |
| 35-39 years | 2002 | 456967717 | 445713782 | 469219101 |
| 40-44 years | 2002 | 381555836 | 373055718 | 390351888 |
| 45-49 years | 2002 | 343198407 | 335203350 | 351999336 |
| 50-54 years | 2002 | 283378503 | 276839194 | 290456928 |
| 55-59 years | 2002 | 216437149 | 211602027 | 221695333 |
| 60-64 years | 2002 | 189847982 | 185805278 | 194298685 |
| 65-69 years | 2002 | 158572359 | 155151468 | 162345837 |
| 70-74 years | 2002 | 124003128 | 121364445 | 126808974 |
| 75-79 years | 2002 | 84433895 | 82697361 | 86291836 |
| 80-84 years | 2002 | 46774505 | 45836555 | 47786281 |
| 85-89 years | 2002 | 21811449 | 21403258 | 22239071 |
| 90-94 years | 2002 | 7869799 | 7732372 | 8018639 |
| 95+ years | 2002 | 2040280 | 2003814 | 2080273 |
| Total | 2002 | 4424173444 | 4325154577 | 4529617108 |
| 15-19 years | 2003 | 606682488 | 592862299 | 621317543 |
| 20-24 years | 2003 | 534767900 | 523287979 | 546747187 |
| 25-29 years | 2003 | 499214349 | 487748631 | 511097544 |
| 30-34 years | 2003 | 492330594 | 479833634 | 505315699 |
| 35-39 years | 2003 | 464324075 | 452325930 | 477122740 |
| 40-44 years | 2003 | 394112707 | 384758208 | 403821539 |
| 45-49 years | 2003 | 347827312 | 339303884 | 356968436 |
| 50-54 years | 2003 | 293821954 | 286350773 | 301617752 |
| 55-59 years | 2003 | 225455626 | 219989761 | 231155157 |
| 60-64 years | 2003 | 191124736 | 186712574 | 195815311 |
| 65-69 years | 2003 | 161862224 | 158067509 | 165855967 |
| 70-74 years | 2003 | 125909711 | 123033745 | 128958252 |
| 75-79 years | 2003 | 87349135 | 85432899 | 89330926 |
| 80-84 years | 2003 | 49186604 | 48126309 | 50258960 |
| 85-89 years | 2003 | 21983311 | 21540826 | 22436162 |
| 90-94 years | 2003 | 8168147 | 8017271 | 8326966 |
| 95+ years | 2003 | 2161713 | 2119378 | 2203891 |
| Total | 2003 | 4506282584 | 4399511610 | 4618350031 |
| 15-19 years | 2004 | 615883401 | 600665162 | 631832723 |
| 20-24 years | 2004 | 545567777 | 532818341 | 558839182 |
| 25-29 years | 2004 | 502013548 | 490028739 | 514557549 |
| 30-34 years | 2004 | 495268641 | 482072896 | 509180176 |
| 35-39 years | 2004 | 469954599 | 456970999 | 483691413 |
| 40-44 years | 2004 | 408650640 | 397958193 | 419568167 |
| 45-49 years | 2004 | 351170444 | 342250510 | 360431511 |
| 50-54 years | 2004 | 302366759 | 294381570 | 310794999 |
| 55-59 years | 2004 | 238113287 | 232190447 | 244514836 |
| 60-64 years | 2004 | 192695822 | 188073724 | 197707095 |
| 65-69 years | 2004 | 164893410 | 160861346 | 169185482 |
| 70-74 years | 2004 | 127788259 | 124743042 | 131034798 |
| 75-79 years | 2004 | 90234396 | 88156275 | 92348608 |
| 80-84 years | 2004 | 51522221 | 50404209 | 52703787 |
| 85-89 years | 2004 | 22433590 | 21959952 | 22923727 |
| 90-94 years | 2004 | 8453719 | 8294463 | 8622496 |
| 95+ years | 2004 | 2285511 | 2240203 | 2330408 |
| Total | 2004 | 4589296024 | 4474070069 | 4710266956 |
| 15-19 years | 2005 | 621771808 | 605500925 | 639352583 |
| 20-24 years | 2005 | 557734427 | 544057379 | 572618393 |
| 25-29 years | 2005 | 507111038 | 494746896 | 520374201 |
| 30-34 years | 2005 | 497076511 | 483198657 | 511842715 |
| 35-39 years | 2005 | 474836585 | 460742426 | 489874792 |
| 40-44 years | 2005 | 423047280 | 411132595 | 435840985 |
| 45-49 years | 2005 | 354616770 | 345296480 | 364634332 |
| 50-54 years | 2005 | 314537879 | 305277967 | 324312293 |
| 55-59 years | 2005 | 248652274 | 241915648 | 255967048 |
| 60-64 years | 2005 | 194262613 | 189092512 | 199891373 |
| 65-69 years | 2005 | 167807680 | 163500029 | 172446807 |
| 70-74 years | 2005 | 130017106 | 126626857 | 133668959 |
| 75-79 years | 2005 | 92843236 | 90590625 | 95254371 |
| 80-84 years | 2005 | 53736705 | 52489221 | 55050179 |
| 85-89 years | 2005 | 23397800 | 22875837 | 23951988 |
| 90-94 years | 2005 | 8709327 | 8532519 | 8899901 |
| 95+ years | 2005 | 2411778 | 2362142 | 2463022 |
| Total | 2005 | 4672570817 | 4547938715 | 4806443942 |
| 15-19 years | 2006 | 623563669 | 606798535 | 641679774 |
| 20-24 years | 2006 | 571463491 | 556685803 | 587761165 |
| 25-29 years | 2006 | 513740008 | 500869483 | 527896204 |
| 30-34 years | 2006 | 497827475 | 483985129 | 512956155 |
| 35-39 years | 2006 | 480258553 | 466067149 | 495497978 |
| 40-44 years | 2006 | 435485536 | 422685786 | 449113689 |
| 45-49 years | 2006 | 360112452 | 350636435 | 370367890 |
| 50-54 years | 2006 | 322549284 | 313260526 | 332613517 |
| 55-59 years | 2006 | 261576303 | 254488742 | 269480358 |
| 60-64 years | 2006 | 197945087 | 192543775 | 203869072 |
| 65-69 years | 2006 | 170237090 | 165833042 | 175133094 |
| 70-74 years | 2006 | 132855791 | 129406816 | 136680289 |
| 75-79 years | 2006 | 95382227 | 93071740 | 97908501 |
| 80-84 years | 2006 | 55886656 | 54599720 | 57248225 |
| 85-89 years | 2006 | 24751547 | 24215349 | 25334415 |
| 90-94 years | 2006 | 8897652 | 8717345 | 9087384 |
| 95+ years | 2006 | 2544617 | 2494070 | 2595837 |
| Total | 2006 | 4755077440 | 4626359446 | 4895223546 |
| 15-19 years | 2007 | 622269327 | 607130027 | 638832487 |
| 20-24 years | 2007 | 585423169 | 571105219 | 600763644 |
| 25-29 years | 2007 | 521732624 | 509531349 | 534893786 |
| 30-34 years | 2007 | 498283381 | 485753952 | 512161638 |
| 35-39 years | 2007 | 485131006 | 471989296 | 499345549 |
| 40-44 years | 2007 | 446052087 | 433820116 | 459151780 |
| 45-49 years | 2007 | 368877497 | 359780966 | 378672057 |
| 50-54 years | 2007 | 329390624 | 320667780 | 338930549 |
| 55-59 years | 2007 | 270959057 | 264093121 | 278701251 |
| 60-64 years | 2007 | 206519392 | 201248832 | 212382815 |
| 65-69 years | 2007 | 172127472 | 168009912 | 176756748 |
| 70-74 years | 2007 | 136266565 | 132985066 | 139915540 |
| 75-79 years | 2007 | 97547783 | 95322564 | 100011867 |
| 80-84 years | 2007 | 58153302 | 56923466 | 59465932 |
| 85-89 years | 2007 | 26292040 | 25756708 | 26867072 |
| 90-94 years | 2007 | 9071543 | 8895253 | 9256378 |
| 95+ years | 2007 | 2680519 | 2631311 | 2733945 |
| Total | 2007 | 4836777387 | 4715644940 | 4968843040 |
| 15-19 years | 2008 | 619025736 | 604957373 | 634164851 |
| 20-24 years | 2008 | 598293158 | 584974135 | 612859740 |
| 25-29 years | 2008 | 531413324 | 519974737 | 543752378 |
| 30-34 years | 2008 | 499723960 | 488621198 | 511939274 |
| 35-39 years | 2008 | 489448370 | 477634524 | 502200664 |
| 40-44 years | 2008 | 453756649 | 442405357 | 465840294 |
| 45-49 years | 2008 | 381654490 | 372940976 | 390967807 |
| 50-54 years | 2008 | 334351165 | 326469287 | 342932593 |
| 55-59 years | 2008 | 281391129 | 274683045 | 288673245 |
| 60-64 years | 2008 | 215704291 | 210753523 | 221219742 |
| 65-69 years | 2008 | 173957866 | 170110452 | 178248816 |
| 70-74 years | 2008 | 139624354 | 136533847 | 143035902 |
| 75-79 years | 2008 | 99590939 | 97476422 | 101884176 |
| 80-84 years | 2008 | 60463968 | 59276473 | 61728492 |
| 85-89 years | 2008 | 27883275 | 27362627 | 28421974 |
| 90-94 years | 2008 | 9265863 | 9091539 | 9441807 |
| 95+ years | 2008 | 2825095 | 2775173 | 2876790 |
| Total | 2008 | 4918373631 | 4806040687 | 5040188547 |
| 15-19 years | 2009 | 614712394 | 601739950 | 628146599 |
| 20-24 years | 2009 | 608637695 | 595661178 | 622086121 |
| 25-29 years | 2009 | 542516841 | 531375109 | 553700319 |
| 30-34 years | 2009 | 503098568 | 492813741 | 513779302 |
| 35-39 years | 2009 | 492801486 | 481968776 | 504544597 |
| 40-44 years | 2009 | 459734673 | 449205874 | 470899333 |
| 45-49 years | 2009 | 396380281 | 387890422 | 405545425 |
| 50-54 years | 2009 | 338025348 | 330802425 | 345785804 |
| 55-59 years | 2009 | 289990711 | 283522623 | 296930816 |
| 60-64 years | 2009 | 228261686 | 223352785 | 233565263 |
| 65-69 years | 2009 | 176048857 | 172442091 | 180008631 |
| 70-74 years | 2009 | 142728644 | 139924744 | 145791307 |
| 75-79 years | 2009 | 101661140 | 99689028 | 103809503 |
| 80-84 years | 2009 | 62740669 | 61607193 | 63936024 |
| 85-89 years | 2009 | 29443981 | 28930165 | 29978934 |
| 90-94 years | 2009 | 9604404 | 9437436 | 9781095 |
| 95+ years | 2009 | 2978278 | 2928900 | 3029631 |
| Total | 2009 | 4999365656 | 4893292441 | 5111318704 |
| 15-19 years | 2010 | 609950128 | 597742864 | 622497415 |
| 20-24 years | 2010 | 614620886 | 602213327 | 627317050 |
| 25-29 years | 2010 | 554462121 | 543651111 | 565442349 |
| 30-34 years | 2010 | 508260908 | 498471357 | 518499786 |
| 35-39 years | 2010 | 494751856 | 484749011 | 505376059 |
| 40-44 years | 2010 | 464822244 | 455017846 | 475320249 |
| 45-49 years | 2010 | 410903514 | 402339502 | 419924990 |
| 50-54 years | 2010 | 341763284 | 335092370 | 348691788 |
| 55-59 years | 2010 | 302127746 | 295901468 | 308953987 |
| 60-64 years | 2010 | 238616758 | 233849171 | 243811096 |
| 65-69 years | 2010 | 178040130 | 174608905 | 181790686 |
| 70-74 years | 2010 | 145726902 | 143011228 | 148725670 |
| 75-79 years | 2010 | 104021646 | 102117680 | 106140146 |
| 80-84 years | 2010 | 64830074 | 63736956 | 66010189 |
| 85-89 years | 2010 | 30934340 | 30454700 | 31434939 |
| 90-94 years | 2010 | 10204486 | 10044999 | 10374833 |
| 95+ years | 2010 | 3127375 | 3078672 | 3176769 |
| Total | 2010 | 5077164399 | 4976081168 | 5183488001 |
| 15-19 years | 2011 | 606360611 | 594589784 | 618393909 |
| 20-24 years | 2011 | 615726483 | 603115468 | 628892047 |
| 25-29 years | 2011 | 567548935 | 556420781 | 579130944 |
| 30-34 years | 2011 | 514464783 | 504450801 | 524811000 |
| 35-39 years | 2011 | 495348280 | 485070297 | 506462253 |
| 40-44 years | 2011 | 470305669 | 459925235 | 481606765 |
| 45-49 years | 2011 | 423380625 | 414020665 | 433742122 |
| 50-54 years | 2011 | 347447633 | 340493648 | 355102913 |
| 55-59 years | 2011 | 310125206 | 303184929 | 317734731 |
| 60-64 years | 2011 | 251284540 | 245956095 | 257211985 |
| 65-69 years | 2011 | 181854614 | 178060294 | 186002441 |
| 70-74 years | 2011 | 148300604 | 145376158 | 151581145 |
| 75-79 years | 2011 | 106781640 | 104647749 | 109151147 |
| 80-84 years | 2011 | 66890106 | 65715663 | 68245308 |
| 85-89 years | 2011 | 32316717 | 31764641 | 32905234 |
| 90-94 years | 2011 | 10936829 | 10755212 | 11128705 |
| 95+ years | 2011 | 3254695 | 3203231 | 3309717 |
| Total | 2011 | 5152327970 | 5046750653 | 5265412370 |
| 15-19 years | 2012 | 604266521 | 591411844 | 617402115 |
| 20-24 years | 2012 | 613674305 | 600374772 | 627668701 |
| 25-29 years | 2012 | 580775574 | 568198988 | 594004777 |
| 30-34 years | 2012 | 521925037 | 511213403 | 533322816 |
| 35-39 years | 2012 | 495552674 | 484804822 | 507392497 |
| 40-44 years | 2012 | 475184702 | 463813389 | 487051420 |
| 45-49 years | 2012 | 434023175 | 423321215 | 445391063 |
| 50-54 years | 2012 | 356296207 | 348513904 | 364683654 |
| 55-59 years | 2012 | 316927809 | 309260272 | 325166628 |
| 60-64 years | 2012 | 260451575 | 254468569 | 267068021 |
| 65-69 years | 2012 | 190244638 | 185952033 | 194938143 |
| 70-74 years | 2012 | 150343752 | 147134402 | 153910369 |
| 75-79 years | 2012 | 109942284 | 107625897 | 112541782 |
| 80-84 years | 2012 | 68689663 | 67316209 | 70150578 |
| 85-89 years | 2012 | 33792349 | 33156325 | 34479128 |
| 90-94 years | 2012 | 11721069 | 11502064 | 11952117 |
| 95+ years | 2012 | 3375641 | 3315010 | 3439849 |
| Total | 2012 | 5227186975 | 5111383116 | 5350563660 |
| 15-19 years | 2013 | 603076397 | 589798159 | 616798080 |
| 20-24 years | 2013 | 609699773 | 595605479 | 624259277 |
| 25-29 years | 2013 | 592435348 | 578582480 | 606703744 |
| 30-34 years | 2013 | 530850776 | 519103845 | 543127061 |
| 35-39 years | 2013 | 496534600 | 484987312 | 508729164 |
| 40-44 years | 2013 | 479400156 | 467308398 | 492483352 |
| 45-49 years | 2013 | 441784269 | 429871237 | 454538635 |
| 50-54 years | 2013 | 369048911 | 360356127 | 378695166 |
| 55-59 years | 2013 | 321855346 | 313603562 | 330706164 |
| 60-64 years | 2013 | 270572885 | 263632746 | 278226459 |
| 65-69 years | 2013 | 199111566 | 194282808 | 204548746 |
| 70-74 years | 2013 | 152368609 | 148851179 | 156378756 |
| 75-79 years | 2013 | 113064927 | 110490398 | 115982492 |
| 80-84 years | 2013 | 70444838 | 68944447 | 72113227 |
| 85-89 years | 2013 | 35296189 | 34573464 | 36049197 |
| 90-94 years | 2013 | 12492317 | 12246555 | 12751341 |
| 95+ years | 2013 | 3497853 | 3429528 | 3571209 |
| Total | 2013 | 5301534761 | 5175667726 | 5435662070 |
| 15-19 years | 2014 | 602502881 | 588109034 | 617349617 |
| 20-24 years | 2014 | 604844180 | 589957271 | 620129591 |
| 25-29 years | 2014 | 601270294 | 585972852 | 617147187 |
| 30-34 years | 2014 | 541087888 | 527976730 | 554517928 |
| 35-39 years | 2014 | 499330325 | 486958232 | 511976315 |
| 40-44 years | 2014 | 482564362 | 469552796 | 496063990 |
| 45-49 years | 2014 | 447768355 | 434981387 | 461224471 |
| 50-54 years | 2014 | 383650184 | 373578346 | 394355495 |
| 55-59 years | 2014 | 325504790 | 317094535 | 334527356 |
| 60-64 years | 2014 | 278902060 | 271158911 | 287089227 |
| 65-69 years | 2014 | 211131041 | 205459258 | 217115977 |
| 70-74 years | 2014 | 154666522 | 150713198 | 158846553 |
| 75-79 years | 2014 | 115953636 | 113078236 | 119020213 |
| 80-84 years | 2014 | 72216011 | 70521616 | 74012523 |
| 85-89 years | 2014 | 36738727 | 35953366 | 37552609 |
| 90-94 years | 2014 | 13207653 | 12936911 | 13485561 |
| 95+ years | 2014 | 3646107 | 3569978 | 3722876 |
| Total | 2014 | 5374985017 | 5237572657 | 5518137488 |
| 15-19 years | 2015 | 602739088 | 587575012 | 618474921 |
| 20-24 years | 2015 | 600149228 | 584819259 | 616537683 |
| 25-29 years | 2015 | 606445316 | 590068520 | 623384277 |
| 30-34 years | 2015 | 552649257 | 538325776 | 567362258 |
| 35-39 years | 2015 | 504246657 | 491321835 | 517839599 |
| 40-44 years | 2015 | 484461088 | 470871193 | 498698642 |
| 45-49 years | 2015 | 452937041 | 439260005 | 467111066 |
| 50-54 years | 2015 | 398041151 | 386318696 | 410319055 |
| 55-59 years | 2015 | 329208085 | 320372956 | 338655869 |
| 60-64 years | 2015 | 290535287 | 281839047 | 299753471 |
| 65-69 years | 2015 | 220972047 | 214662323 | 227610691 |
| 70-74 years | 2015 | 156861725 | 152517102 | 161474687 |
| 75-79 years | 2015 | 118780057 | 115644895 | 122093087 |
| 80-84 years | 2015 | 74231527 | 72298043 | 76202223 |
| 85-89 years | 2015 | 38073226 | 37221433 | 38949391 |
| 90-94 years | 2015 | 13891262 | 13594453 | 14194408 |
| 95+ years | 2015 | 3874883 | 3791287 | 3956932 |
| Total | 2015 | 5448096924 | 5300501835 | 5602618261 |
| 15-19 years | 2016 | 603580125 | 587665290 | 619642224 |
| 20-24 years | 2016 | 597052243 | 581576923 | 612922765 |
| 25-29 years | 2016 | 607160027 | 590908633 | 623663527 |
| 30-34 years | 2016 | 565703744 | 550873795 | 580896709 |
| 35-39 years | 2016 | 510427292 | 497390934 | 523928994 |
| 40-44 years | 2016 | 485119767 | 471974613 | 498856232 |
| 45-49 years | 2016 | 458610673 | 445263725 | 472455154 |
| 50-54 years | 2016 | 410405422 | 398582227 | 422937726 |
| 55-59 years | 2016 | 334815009 | 326027348 | 344097964 |
| 60-64 years | 2016 | 298107194 | 289582079 | 307257077 |
| 65-69 years | 2016 | 233016893 | 226672896 | 239964749 |
| 70-74 years | 2016 | 160629109 | 156318247 | 165284559 |
| 75-79 years | 2016 | 121320545 | 118311817 | 124673094 |
| 80-84 years | 2016 | 76577010 | 74686662 | 78586723 |
| 85-89 years | 2016 | 39448835 | 38600593 | 40340181 |
| 90-94 years | 2016 | 14568280 | 14261728 | 14879274 |
| 95+ years | 2016 | 4131509 | 4049311 | 4218301 |
| Total | 2016 | 5520673679 | 5372746820 | 5674605254 |
| 15-19 years | 2017 | 605193443 | 589158418 | 622315223 |
| 20-24 years | 2017 | 595480749 | 579944171 | 612465442 |
| 25-29 years | 2017 | 604731282 | 588608774 | 622078626 |
| 30-34 years | 2017 | 578817474 | 563504980 | 595328524 |
| 35-39 years | 2017 | 517782480 | 504531281 | 532225147 |
| 40-44 years | 2017 | 485372751 | 472424861 | 499251048 |
| 45-49 years | 2017 | 463630995 | 450528594 | 477603839 |
| 50-54 years | 2017 | 420960618 | 408831747 | 433816270 |
| 55-59 years | 2017 | 343469603 | 334455917 | 353064090 |
| 60-64 years | 2017 | 304485987 | 295977329 | 313684723 |
| 65-69 years | 2017 | 241727275 | 235212294 | 249040125 |
| 70-74 years | 2017 | 168505837 | 164043686 | 173430226 |
| 75-79 years | 2017 | 123416368 | 120268845 | 126807571 |
| 80-84 years | 2017 | 79205796 | 77303764 | 81276811 |
| 85-89 years | 2017 | 40673020 | 39790469 | 41622330 |
| 90-94 years | 2017 | 15285198 | 14959838 | 15623840 |
| 95+ years | 2017 | 4396913 | 4305343 | 4491350 |
| Total | 2017 | 5593135789 | 5443850311 | 5754125185 |
| 15-19 years | 2018 | 607935331 | 591436058 | 624709846 |
| 20-24 years | 2018 | 594837159 | 578846611 | 611449481 |
| 25-29 years | 2018 | 600498512 | 584379518 | 617046795 |
| 30-34 years | 2018 | 590341040 | 574233104 | 606800175 |
| 35-39 years | 2018 | 526565910 | 512631971 | 540580522 |
| 40-44 years | 2018 | 486382760 | 473766958 | 499510301 |
| 45-49 years | 2018 | 467961541 | 455463816 | 481292448 |
| 50-54 years | 2018 | 428680253 | 417121970 | 441181380 |
| 55-59 years | 2018 | 355923529 | 346971486 | 365479906 |
| 60-64 years | 2018 | 309019455 | 300913674 | 317891234 |
| 65-69 years | 2018 | 251242945 | 244671758 | 258449265 |
| 70-74 years | 2018 | 176655967 | 172346427 | 181518463 |
| 75-79 years | 2018 | 125472909 | 122487780 | 128771043 |
| 80-84 years | 2018 | 81794977 | 79978589 | 83832772 |
| 85-89 years | 2018 | 41922846 | 41049528 | 42859721 |
| 90-94 years | 2018 | 16043812 | 15718442 | 16385247 |
| 95+ years | 2018 | 4666218 | 4572675 | 4765207 |
| Total | 2018 | 5665945163 | 5516590363 | 5822523806 |
| 15-19 years | 2019 | 611964864 | 595122108 | 629967521 |
| 20-24 years | 2019 | 594924367 | 578481386 | 612429633 |
| 25-29 years | 2019 | 595610151 | 579466824 | 612928455 |
| 30-34 years | 2019 | 599062598 | 582696098 | 616412942 |
| 35-39 years | 2019 | 536692305 | 522202108 | 551699867 |
| 40-44 years | 2019 | 489246750 | 476405396 | 502927671 |
| 45-49 years | 2019 | 471230258 | 458627741 | 484642792 |
| 50-54 years | 2019 | 434648511 | 422916529 | 447139439 |
| 55-59 years | 2019 | 370192263 | 360657058 | 380300741 |
| 60-64 years | 2019 | 312308385 | 304285555 | 320826118 |
| 65-69 years | 2019 | 259038664 | 252183887 | 266359371 |
| 70-74 years | 2019 | 187569520 | 182855964 | 192710445 |
| 75-79 years | 2019 | 127669776 | 124647053 | 131021407 |
| 80-84 years | 2019 | 84197083 | 82309362 | 86308152 |
| 85-89 years | 2019 | 43277839 | 42376682 | 44241645 |
| 90-94 years | 2019 | 16820238 | 16485632 | 17172133 |
| 95+ years | 2019 | 4952025 | 4856590 | 5053133 |
| Total | 2019 | 5739405597 | 5586575973 | 5902141465 |
| 15-19 years | 2020 | 617182562 | 598950027 | 635770419 |
| 20-24 years | 2020 | 595839341 | 578330360 | 613970557 |
| 25-29 years | 2020 | 591051881 | 574613718 | 608315605 |
| 30-34 years | 2020 | 604034936 | 587160087 | 621671941 |
| 35-39 years | 2020 | 548078541 | 532996885 | 563747398 |
| 40-44 years | 2020 | 494192164 | 480850791 | 508129248 |
| 45-49 years | 2020 | 473085416 | 460451940 | 486364826 |
| 50-54 years | 2020 | 439653643 | 427815525 | 452165067 |
| 55-59 years | 2020 | 384108080 | 374275278 | 394477792 |
| 60-64 years | 2020 | 315441703 | 307515757 | 323885562 |
| 65-69 years | 2020 | 269557352 | 262438621 | 277151167 |
| 70-74 years | 2020 | 196046223 | 191217039 | 201356459 |
| 75-79 years | 2020 | 129360401 | 126321054 | 132710043 |
| 80-84 years | 2020 | 86243045 | 84320927 | 88368994 |
| 85-89 years | 2020 | 44592974 | 43687130 | 45589929 |
| 90-94 years | 2020 | 17441307 | 17101457 | 17797992 |
| 95+ years | 2020 | 5228020 | 5128987 | 5329571 |
| Total | 2020 | 5811137590 | 5653175584 | 5976802570 |
| 15-19 years | 2021 | 623979871 | 604617205 | 644251986 |
| 20-24 years | 2021 | 597158138 | 578749000 | 616901307 |
| 25-29 years | 2021 | 588343219 | 570487097 | 607422119 |
| 30-34 years | 2021 | 604480175 | 586195015 | 623603836 |
| 35-39 years | 2021 | 560866106 | 544251815 | 578297965 |
| 40-44 years | 2021 | 500250796 | 485928431 | 515432478 |
| 45-49 years | 2021 | 473504626 | 460213136 | 487919674 |
| 50-54 years | 2021 | 444922983 | 432300957 | 458714304 |
| 55-59 years | 2021 | 395728004 | 384764582 | 407669300 |
| 60-64 years | 2021 | 320047853 | 311524380 | 329387624 |
| 65-69 years | 2021 | 275842159 | 268116931 | 284217678 |
| 70-74 years | 2021 | 205839220 | 200406729 | 211972020 |
| 75-79 years | 2021 | 131884405 | 128518633 | 135613447 |
| 80-84 years | 2021 | 87583057 | 85481848 | 89948888 |
| 85-89 years | 2021 | 45721791 | 44709231 | 46852863 |
| 90-94 years | 2021 | 17889374 | 17518671 | 18289317 |
| 95+ years | 2021 | 5450309 | 5337366 | 5569993 |
| Total | 2021 | 5879492084 | 5709121028 | 6062064800 |

# **Supplementary Table S3.** The incidence, mortality and DALYs of EOLC, MOLC and LOLC in 1990.

| Group | Incidence | | Mortality | | DALYs | |
| --- | --- | --- | --- | --- | --- | --- |
|  | Cases (95%UI), 1990 | ASIR (95% UI), 1990 | Cases (95%UI), 1990 | ASMR (95% UI), 1990 | Cases (95%UI), 1990 | ASDR (95% UI), 1990 |
| EOLC |  |  |  |  |  |  |
| Global | 104060 (97860, 110755) | 4.81 (4.51, 5.13) | 90525 (84883, 96630) | 4.18 (3.92, 4.48) | 4361710 (4080803, 4659757) | 198.3 (185.28, 212.38) |
| Gender |  |  |  |  |  |  |
| Male | 73344 (68038, 79758) | 6.7 (6.19, 7.29) | 64399 (59509, 70112) | 5.88 (5.42, 6.42) | 3081683 (2843170, 3359056) | 277.11 (254.92, 303.39) |
| Female | 30716 (27911, 34169) | 2.85 (2.58, 3.18) | 26126 (23607, 29165) | 2.42 (2.17, 2.71) | 1280027 (1154305, 1430378) | 116.44 (104.43, 130.78) |
| SDI Levels |  |  |  |  |  |  |
| Low SDI | 1638 (1372, 2093) | 1.05 (0.87, 1.36) | 1526 (1274, 1944) | 0.97 (0.81, 1.27) | 73674 (61582, 93461) | 46.02 (38.02, 59.76) |
| Low-middle SDI | 6773 (6041, 7761) | 1.62 (1.43, 1.87) | 6291 (5622, 7222) | 1.5 (1.32, 1.74) | 308559 (276386, 354088) | 72.07 (63.48, 83.48) |
| Middle SDI | 29326 (26345, 32665) | 4.28 (3.82, 4.82) | 26773 (24042, 29819) | 3.91 (3.49, 4.39) | 1318980 (1186074, 1469163) | 188.02 (167.48, 211.48) |
| High-middle SDI | 36596 (33629, 39536) | 7.83 (7.16, 8.51) | 32473 (29804, 35189) | 6.96 (6.35, 7.58) | 1560766 (1428782, 1692400) | 328.85 (299.61, 359.2) |
| High SDI | 29591 (29027, 30259) | 6.57 (6.4, 6.76) | 23341 (22870, 23845) | 5.19 (5.06, 5.33) | 1094026 (1071588, 1117322) | 241.85 (235.65, 248.85) |
| GBD Regions |  |  |  |  |  |  |
| High-income Asia Pacific | 3897 (3716, 4099) | 3.99 (3.72, 4.31) | 2951 (2813, 3118) | 3.03 (2.83, 3.28) | 140849 (134053, 148535) | 145.04 (134.94, 157.45) |
| Central Asia | 1605 (1494, 1716) | 7.11 (6.53, 7.65) | 1464 (1361, 1567) | 6.49 (5.96, 7) | 71641 (66795, 76541) | 307 (282.12, 330.78) |
| Southeast Asia | 5786 (5148, 6445) | 3.35 (2.92, 3.81) | 5359 (4761, 5978) | 3.1 (2.69, 3.53) | 262808 (233453, 292377) | 148.09 (128.36, 168.81) |
| East Asia | 36845 (31656, 42614) | 6.88 (5.83, 8.01) | 33369 (28680, 38549) | 6.24 (5.29, 7.26) | 1644186 (1410867, 1898079) | 300.47 (254.72, 349.84) |
| Central Europe | 5702 (5455, 5959) | 9.4 (8.91, 9.92) | 5099 (4877, 5328) | 8.42 (7.99, 8.86) | 237756 (227292, 248305) | 390.14 (369.83, 411.15) |
| Eastern Europe | 8805 (8465, 9172) | 8.87 (8.42, 9.38) | 7626 (7348, 7927) | 7.7 (7.33, 8.11) | 357068 (344090, 370544) | 355.91 (338.65, 374.91) |
| North Africa and Middle East | 3856 (3112, 4710) | 6.77 (5.24, 8.62) | 3604 (2912, 4416) | 6.32 (4.91, 8.08) | 176508 (143452, 215181) | 301.22 (233.74, 384.71) |
| Australasia | 472 (430, 514) | 4.58 (4.09, 5.09) | 341 (313, 369) | 3.32 (2.98, 3.67) | 15889 (14532, 17194) | 153.72 (137.82, 170.29) |
| Western Europe | 13016 (12580, 13485) | 6.68 (6.36, 7.03) | 10363 (10025, 10712) | 5.32 (5.07, 5.58) | 483137 (466912, 499681) | 247.67 (235.79, 260.14) |
| Andean Latin America | 348 (299, 404) | 2.46 (2.01, 3) | 321 (277, 374) | 2.27 (1.86, 2.77) | 16315 (14119, 19101) | 111.65 (91.43, 136.12) |
| Caribbean | 573 (530, 621) | 4.05 (3.64, 4.51) | 495 (458, 533) | 3.5 (3.15, 3.9) | 23811 (22101, 25715) | 165.62 (148.83, 184.71) |
| High-income North America | 11454 (11162, 11721) | 8.12 (7.87, 8.37) | 8749 (8521, 8958) | 6.21 (6.02, 6.39) | 407776 (397828, 417533) | 287.27 (278.64, 295.84) |
| Western Sub-Saharan Africa | 274 (227, 326) | 0.45 (0.37, 0.55) | 254 (211, 303) | 0.42 (0.35, 0.51) | 12373 (10251, 14752) | 20.06 (16.41, 24.46) |
| South Asia | 5116 (4462, 6006) | 2.48 (2.13, 2.91) | 4749 (4139, 5588) | 2.3 (1.98, 2.71) | 231712 (201779, 272308) | 110.21 (94.48, 129.8) |
| Oceania | 64 (45, 97) | 2.73 (1.81, 4.3) | 60 (42, 90) | 2.54 (1.68, 3.96) | 2954 (2089, 4464) | 122.07 (80.94, 191.26) |
| Central Sub-Saharan Africa | 253 (173, 387) | 1.6 (1.06, 2.46) | 235 (162, 355) | 1.49 (0.99, 2.29) | 11250 (7723, 16951) | 69.55 (46.43, 106.53) |
| Central Latin America | 1382 (1335, 1428) | 2.33 (2.21, 2.45) | 1268 (1225, 1310) | 2.13 (2.02, 2.24) | 63858 (61728, 66046) | 103.67 (98.41, 109.01) |
| Southern Latin America | 1390 (1277, 1499) | 6.34 (5.55, 7.25) | 1254 (1154, 1351) | 5.72 (5.01, 6.55) | 59019 (54467, 63711) | 267.9 (234.61, 307.22) |
| Tropical Latin America | 1737 (1674, 1810) | 2.91 (2.74, 3.07) | 1590 (1532, 1657) | 2.66 (2.51, 2.82) | 77078 (74318, 80220) | 126.37 (119.2, 133.66) |
| Eastern Sub-Saharan Africa | 674 (557, 886) | 1.27 (1.02, 1.7) | 629 (521, 821) | 1.18 (0.95, 1.58) | 29968 (24837, 38928) | 55.14 (44.48, 73.54) |
| Southern Sub-Saharan Africa | 812 (690, 944) | 4.62 (3.85, 5.53) | 744 (633, 864) | 4.24 (3.54, 5.09) | 35752 (30436, 41364) | 200.03 (166.74, 239.95) |
| MOLC |  |  |  |  |  |  |
| Global | 629831 (599064, 660701) | 92.77 (88.1, 97.48) | 576757 (546547, 606960) | 85 (80.47, 89.61) | 17324507 (16412605, 18230086) | 2545.79 (2408.07, 2685.3) |
| Gender |  |  |  |  |  |  |
| Male | 482045 (453672, 512870) | 146.09 (137.33, 155.69) | 445317 (417537, 475829) | 135.12 (126.69, 144.59) | 13404298 (12559861, 14356359) | 4034.17 (3779.4, 4319.15) |
| Female | 147787 (138229, 158115) | 42.42 (39.51, 45.6) | 131440 (122391, 141408) | 37.72 (35.01, 40.71) | 3920209 (3646836, 4226605) | 1127.31 (1044.96, 1219.81) |
| SDI Levels |  |  |  |  |  |  |
| Low SDI | 7881 (6534, 10131) | 19.4 (16.06, 25.06) | 7947 (6607, 10200) | 19.61 (16.25, 25.32) | 240956 (200436, 309974) | 587.11 (486.51, 759.22) |
| Low-middle SDI | 29531 (26041, 34539) | 27.26 (23.91, 32.08) | 29669 (26146, 34742) | 27.47 (24.12, 32.38) | 902154 (795306, 1054975) | 823.93 (724.08, 970.96) |
| Middle SDI | 124049 (110685, 138383) | 67.07 (59.59, 74.96) | 122421 (108966, 136598) | 66.34 (58.97, 74.03) | 3719007 (3314369, 4156761) | 1994.48 (1770.14, 2227.94) |
| High-middle SDI | 222815 (209446, 236098) | 127.48 (119.43, 135.7) | 210276 (197090, 223130) | 120.34 (112.5, 128.32) | 6391006 (5991242, 6783888) | 3655.87 (3413.78, 3899.34) |
| High SDI | 244680 (239573, 249075) | 143.46 (139.91, 146.75) | 205603 (201528, 209200) | 120.36 (117.57, 122.9) | 6046054 (5930779, 6147847) | 3582.17 (3501.12, 3659.12) |
| GBD Regions |  |  |  |  |  |  |
| High-income Asia Pacific | 26856 (25908, 27787) | 79.23 (74.99, 83.64) | 20637 (19977, 21326) | 60.95 (58.18, 63.93) | 611299 (591391, 631973) | 1798.42 (1712.08, 1889.2) |
| Central Asia | 9229 (8758, 9727) | 108.14 (101.81, 114.86) | 9049 (8585, 9525) | 106.44 (100.19, 113.16) | 283014 (267964, 298752) | 3275.08 (3077.25, 3489.45) |
| Southeast Asia | 28360 (24844, 32151) | 62.33 (53.92, 71.18) | 28454 (24921, 32188) | 62.74 (54.23, 71.44) | 861463 (756786, 973503) | 1872.48 (1621.17, 2132.63) |
| East Asia | 158544 (133796, 184108) | 100.23 (84.07, 117.16) | 154406 (130344, 179478) | 97.73 (81.96, 114.24) | 4672624 (3933494, 5422726) | 2941.21 (2461.95, 3444.45) |
| Central Europe | 38866 (37509, 40156) | 147.78 (142.21, 153.35) | 37678 (36420, 38901) | 142.95 (137.68, 148.31) | 1142787 (1104273, 1180216) | 4383.7 (4222.76, 4549.93) |
| Eastern Europe | 74726 (72963, 76466) | 150.23 (145.31, 155.18) | 68982 (67444, 70415) | 138.77 (134.68, 142.74) | 2131692 (2082279, 2175356) | 4277.82 (4151.38, 4403.32) |
| North Africa and Middle East | 18018 (14832, 21452) | 118.16 (94.18, 146.09) | 18169 (15007, 21639) | 119.44 (95.37, 147.22) | 554095 (456124, 661058) | 3602.23 (2865.79, 4444.29) |
| Australasia | 4544 (4310, 4780) | 124.31 (115.57, 133.59) | 3753 (3561, 3936) | 102.37 (95.46, 109.38) | 108821 (103567, 114098) | 3018.77 (2813.92, 3233.15) |
| Western Europe | 113004 (110377, 115439) | 130.16 (126.1, 134.34) | 98871 (96583, 100983) | 113.47 (110.17, 116.71) | 2897254 (2830099, 2961197) | 3378.71 (3277.29, 3478.09) |
| Andean Latin America | 1248 (1064, 1433) | 37.09 (30.69, 44.06) | 1249 (1066, 1438) | 37.23 (30.88, 44.32) | 37466 (32085, 43035) | 1103.01 (913.13, 1315.31) |
| Caribbean | 2594 (2441, 2781) | 62.79 (57.91, 68.47) | 2434 (2286, 2615) | 58.94 (54.4, 64.45) | 72910 (68347, 78425) | 1764.16 (1627.24, 1928.45) |
| High-income North America | 103130 (100250, 105531) | 203.49 (197.18, 209.28) | 82523 (80424, 84428) | 162.45 (157.71, 166.77) | 2413878 (2353599, 2468685) | 4854.51 (4714.88, 4985.27) |
| Western Sub-Saharan Africa | 1618 (1343, 1926) | 10.66 (8.76, 12.78) | 1632 (1354, 1945) | 10.78 (8.88, 12.93) | 49085 (40758, 58463) | 320.66 (264.02, 384.75) |
| South Asia | 20865 (18239, 24191) | 40.29 (34.86, 47.26) | 20930 (18277, 24280) | 40.56 (35.15, 47.51) | 639883 (559616, 742745) | 1220.18 (1057.55, 1428.53) |
| Oceania | 269 (191, 408) | 49.21 (33.74, 75.52) | 271 (192, 408) | 49.67 (33.95, 75.32) | 8144 (5772, 12317) | 1472.21 (1002.68, 2245.66) |
| Central Sub-Saharan Africa | 1416 (1014, 2140) | 33.1 (22.67, 51.32) | 1425 (1029, 2153) | 33.45 (23.05, 51.78) | 43517 (31309, 65256) | 1005.63 (693.73, 1559.82) |
| Central Latin America | 5594 (5440, 5738) | 40.47 (38.92, 41.98) | 5555 (5403, 5701) | 40.3 (38.76, 41.82) | 166620 (161928, 170774) | 1193.67 (1147.64, 1239.31) |
| Southern Latin America | 7253 (6753, 7799) | 95.54 (87.46, 104.19) | 7107 (6609, 7628) | 93.53 (85.6, 101.72) | 214558 (199041, 230756) | 2837.35 (2591.57, 3094.49) |
| Tropical Latin America | 8056 (7779, 8317) | 51.26 (48.8, 53.8) | 7978 (7713, 8233) | 50.86 (48.44, 53.34) | 242390 (234342, 250340) | 1531.58 (1458.87, 1605.94) |
| Eastern Sub-Saharan Africa | 3174 (2639, 3989) | 23.97 (19.79, 30.48) | 3202 (2676, 4024) | 24.25 (20.07, 30.79) | 97092 (80760, 122724) | 725.94 (599.76, 927.85) |
| Southern Sub-Saharan Africa | 2467 (2128, 3061) | 53.57 (45.37, 66.11) | 2450 (2110, 3060) | 53.29 (45.24, 65.85) | 75915 (65335, 94763) | 1638.46 (1388.74, 2024.69) |
| LOLC |  |  |  |  |  |  |
| Global | 398172 (373736, 416385) | 195.39 (181.97, 205.24) | 412846 (387126, 433229) | 204.11 (189.79, 214.79) | 6773620 (6388937, 7100599) | 3290.52 (3077.82, 3458.64) |
| Gender |  |  |  |  |  |  |
| Male | 277524 (262478, 292364) | 334.42 (311.83, 354.03) | 289105 (272795, 305806) | 353.44 (329.01, 375.52) | 4798755 (4526217, 5083737) | 5635.25 (5266, 5993.46) |
| Female | 120648 (109120, 128534) | 100.77 (90.16, 108.01) | 123741 (112357, 132158) | 103.81 (92.79, 111.71) | 1974865 (1801441, 2109475) | 1647 (1488.82, 1768.78) |
| SDI Levels |  |  |  |  |  |  |
| Low SDI | 3402 (2806, 4239) | 35.27 (28.85, 44.72) | 3943 (3254, 4916) | 41.66 (34.03, 52.74) | 68979 (56981, 86063) | 690.12 (565.59, 873.14) |
| Low-middle SDI | 13228 (11480, 15195) | 49.83 (43.37, 58.15) | 15446 (13424, 17756) | 59.02 (51.4, 68.95) | 261572 (227637, 301433) | 960.46 (838.71, 1121.16) |
| Middle SDI | 64117 (57847, 70080) | 139.88 (124.73, 154.42) | 73531 (66321, 80269) | 163.33 (145.85, 180.1) | 1237496 (1115268, 1353287) | 2615.81 (2337.08, 2883.95) |
| High-middle SDI | 106230 (99229, 113037) | 203.57 (187.71, 218.44) | 116786 (108800, 124623) | 225.34 (207.25, 242.13) | 1940129 (1807385, 2073341) | 3697.87 (3413.56, 3976.03) |
| High SDI | 210765 (196990, 217352) | 304.13 (283.65, 314.96) | 202661 (189566, 208887) | 291.52 (272.38, 301.69) | 3257700 (3076744, 3348048) | 4757.08 (4483.57, 4902.26) |
| GBD Regions |  |  |  |  |  |  |
| High-income Asia Pacific | 30776 (28331, 32248) | 271.99 (248.74, 289.51) | 27661 (25613, 28813) | 245.95 (226.05, 258.41) | 433366 (406008, 449709) | 3810.33 (3547.21, 3986.31) |
| Central Asia | 2478 (2339, 2605) | 112.49 (105.35, 119.22) | 2844 (2688, 2992) | 128.46 (120.19, 136.39) | 47913 (45360, 50373) | 2207.33 (2075.28, 2334) |
| Southeast Asia | 13455 (11802, 15197) | 122.26 (104.73, 140.8) | 15758 (13823, 17792) | 144.81 (124.54, 166.97) | 263584 (231460, 297264) | 2346.2 (2022.23, 2699.33) |
| East Asia | 87504 (76677, 97999) | 229.26 (197.95, 260.94) | 98682 (86637, 110618) | 266.21 (229.96, 303.16) | 1673989 (1462379, 1877219) | 4180.79 (3608.49, 4754.91) |
| Central Europe | 14518 (13862, 15061) | 182.04 (172.71, 189.91) | 16423 (15715, 17032) | 205.82 (195.42, 214.63) | 268929 (258120, 278426) | 3409.63 (3254.07, 3545.45) |
| Eastern Europe | 23836 (22951, 24574) | 156.31 (149.41, 162.23) | 25181 (24226, 25932) | 164.92 (157.89, 170.65) | 425263 (410158, 436711) | 2819.5 (2710.9, 2912.03) |
| North Africa and Middle East | 8016 (6600, 9584) | 221.31 (176.76, 275.68) | 9430 (7766, 11278) | 263.76 (210.47, 328.51) | 157164 (129260, 187926) | 4229 (3395.31, 5246.92) |
| Australasia | 3999 (3713, 4230) | 271.85 (244.44, 297.95) | 3807 (3537, 4024) | 259.27 (233.8, 283.29) | 62284 (58294, 65627) | 4233.64 (3856.23, 4597.31) |
| Western Europe | 94013 (88548, 97079) | 251.5 (236.5, 261.1) | 97511 (91935, 100710) | 258.88 (243.72, 268.99) | 1547584 (1468643, 1591495) | 4225.76 (4010.21, 4372.57) |
| Andean Latin America | 997 (869, 1132) | 97.52 (81.72, 116.24) | 1184 (1031, 1340) | 115.99 (97.31, 138.32) | 18707 (16289, 21362) | 1830.05 (1538.61, 2177.1) |
| Caribbean | 2545 (2399, 2699) | 172.48 (156.71, 188.93) | 2809 (2656, 2972) | 191.81 (174.75, 209.9) | 43899 (41543, 46449) | 2954.69 (2708.23, 3213.37) |
| High-income North America | 90827 (83909, 94491) | 391.83 (361.19, 409.57) | 82203 (75969, 85541) | 353.6 (325.7, 369.41) | 1341989 (1252032, 1391004) | 5838.27 (5435.27, 6075.28) |
| Western Sub-Saharan Africa | 965 (807, 1136) | 23.66 (19.66, 28.17) | 1128 (946, 1329) | 28.07 (23.36, 33.37) | 19089 (16043, 22585) | 455.69 (379.88, 542.55) |
| South Asia | 7947 (6776, 9257) | 66.41 (55.58, 78.13) | 9245 (7887, 10759) | 78.45 (65.53, 92.25) | 158492 (135348, 184025) | 1288.31 (1081.34, 1510.5) |
| Oceania | 127 (95, 184) | 120.6 (88.51, 175.38) | 147 (110, 210) | 142.98 (105.27, 207.46) | 2568 (1913, 3697) | 2317.67 (1704, 3362.9) |
| Central Sub-Saharan Africa | 509 (364, 772) | 60.49 (40.61, 95.35) | 587 (419, 885) | 71.54 (47.99, 112.4) | 10495 (7482, 15887) | 1181.4 (798.77, 1853) |
| Central Latin America | 4310 (4092, 4443) | 107.14 (100.97, 112.01) | 5085 (4825, 5242) | 127.28 (119.87, 133.15) | 80174 (76565, 82573) | 1975.86 (1873.15, 2060.68) |
| Southern Latin America | 4320 (4012, 4642) | 162.81 (147.49, 178.54) | 4981 (4630, 5347) | 188.63 (170.76, 207.11) | 81573 (75849, 87581) | 3057.97 (2784.93, 3344.56) |
| Tropical Latin America | 4493 (4206, 4704) | 102.46 (94.48, 108.79) | 5222 (4889, 5468) | 120.55 (111.15, 128.16) | 86312 (81630, 90148) | 1929.55 (1796.65, 2042.21) |
| Eastern Sub-Saharan Africa | 1325 (1121, 1568) | 40.55 (33.78, 48.45) | 1530 (1291, 1805) | 47.71 (39.77, 56.85) | 27187 (22948, 32171) | 801.96 (669.66, 956.24) |
| Southern Sub-Saharan Africa | 1211 (1052, 1423) | 93.77 (79.03, 112.17) | 1428 (1241, 1693) | 111.5 (93.95, 133.92) | 23059 (20044, 27440) | 1754.05 (1483.09, 2105.96) |

Notes: ASIR, ASMR and ASDR are reported per 100,000 population.

Abbreviations: DALYs, disability-adjusted life-years; AAPC, average annual percentage change; SDI: socio-demographic index; GBD, global burden of disease; EOLC, early-onset lung cancer; MOLC, middle-onset lung cancer; LOLC, late-onset lung cancer; ASIR, age- standardized incidence rate; ASMR, age-standardized mortality rate; ASDR, age-standardized disability-adjusted life year rate; UI, uncertainty interval; CI, confidence interval.

# **Supplementary Table S4.** List of countries/territories with top 5 significantly increased EAPC of ASIR, ASMR and ASDR in EOLC, MOLC and LOLC, by gender, from 1990 to 2021.

| Group | Measure | Gender | Country/Territory | EAPC (95% CI), 1990-2021 | P value |
| --- | --- | --- | --- | --- | --- |
| EOLC | ASIR | Both | Lesotho | 4.06 (3.49, 4.64) | <0.001 |
| EOLC | ASIR | Both | Mozambique | 2.25 (2, 2.5) | <0.001 |
| EOLC | ASIR | Both | Zimbabwe | 2.07 (1.32, 2.83) | <0.001 |
| EOLC | ASIR | Both | Eswatini | 1.91 (1.16, 2.66) | <0.001 |
| EOLC | ASIR | Both | Kenya | 1.57 (1.25, 1.89) | <0.001 |
| EOLC | ASIR | Male | Lesotho | 3.72 (3.11, 4.32) | <0.001 |
| EOLC | ASIR | Male | Mozambique | 2.61 (2.31, 2.9) | <0.001 |
| EOLC | ASIR | Male | Eswatini | 1.85 (1.01, 2.69) | <0.001 |
| EOLC | ASIR | Male | Zambia | 1.5 (1.28, 1.73) | <0.001 |
| EOLC | ASIR | Male | Zimbabwe | 1.4 (0.68, 2.14) | 0.001 |
| EOLC | ASIR | Female | Lesotho | 4.21 (3.53, 4.89) | <0.001 |
| EOLC | ASIR | Female | Zimbabwe | 3.38 (2.37, 4.41) | <0.001 |
| EOLC | ASIR | Female | France | 2.92 (2.14, 3.71) | <0.001 |
| EOLC | ASIR | Female | Monaco | 2.84 (2.21, 3.47) | <0.001 |
| EOLC | ASIR | Female | Bulgaria | 2.69 (2.36, 3.03) | <0.001 |
| EOLC | ASIR | Female | Guam | 2.69 (2.36, 3.03) | <0.001 |
| EOLC | ASMR | Both | Lesotho | 4.06 (3.49, 4.64) | <0.001 |
| EOLC | ASMR | Both | Mozambique | 2.22 (1.98, 2.47) | <0.001 |
| EOLC | ASMR | Both | Zimbabwe | 2.08 (1.33, 2.84) | <0.001 |
| EOLC | ASMR | Both | Eswatini | 1.89 (1.14, 2.65) | <0.001 |
| EOLC | ASMR | Both | Kenya | 1.58 (1.25, 1.91) | <0.001 |
| EOLC | ASMR | Male | Lesotho | 3.71 (3.1, 4.32) | <0.001 |
| EOLC | ASMR | Male | Mozambique | 2.58 (2.29, 2.88) | <0.001 |
| EOLC | ASMR | Male | Eswatini | 1.84 (0.99, 2.68) | <0.001 |
| EOLC | ASMR | Male | Zambia | 1.49 (1.26, 1.72) | <0.001 |
| EOLC | ASMR | Male | Zimbabwe | 1.42 (0.7, 2.16) | 0.001 |
| EOLC | ASMR | Female | Lesotho | 4.2 (3.51, 4.88) | <0.001 |
| EOLC | ASMR | Female | Zimbabwe | 3.41 (2.38, 4.44) | <0.001 |
| EOLC | ASMR | Female | Bulgaria | 2.58 (2.24, 2.92) | <0.001 |
| EOLC | ASMR | Female | Guam | 2.56 (2.2, 2.91) | <0.001 |
| EOLC | ASMR | Female | Kenya | 2.43 (2.29, 2.56) | <0.001 |
| EOLC | ASDR | Both | Lesotho | 4.08 (3.5, 4.65) | <0.001 |
| EOLC | ASDR | Both | Mozambique | 2.24 (1.99, 2.49) | <0.001 |
| EOLC | ASDR | Both | Zimbabwe | 2.12 (1.37, 2.89) | <0.001 |
| EOLC | ASDR | Both | Eswatini | 1.91 (1.16, 2.67) | <0.001 |
| EOLC | ASDR | Both | Egypt | 1.56 (1.36, 1.76) | <0.001 |
| EOLC | ASDR | Both | Kenya | 1.56 (1.24, 1.88) | <0.001 |
| EOLC | ASDR | Male | Lesotho | 3.72 (3.12, 4.33) | <0.001 |
| EOLC | ASDR | Male | Mozambique | 2.61 (2.31, 2.91) | <0.001 |
| EOLC | ASDR | Male | Eswatini | 1.85 (1.01, 2.69) | <0.001 |
| EOLC | ASDR | Male | Zambia | 1.49 (1.26, 1.72) | <0.001 |
| EOLC | ASDR | Male | Zimbabwe | 1.46 (0.73, 2.2) | <0.001 |
| EOLC | ASDR | Female | Lesotho | 4.23 (3.54, 4.92) | <0.001 |
| EOLC | ASDR | Female | Zimbabwe | 3.43 (2.41, 4.46) | <0.001 |
| EOLC | ASDR | Female | Bulgaria | 2.55 (2.2, 2.91) | <0.001 |
| EOLC | ASDR | Female | Guam | 2.53 (2.21, 2.85) | <0.001 |
| EOLC | ASDR | Female | Equatorial Guinea | 2.39 (2.15, 2.62) | <0.001 |
| MOLC | ASIR | Both | Egypt | 3.54 (3.05, 4.04) | <0.001 |
| MOLC | ASIR | Both | Lesotho | 3.15 (2.7, 3.6) | <0.001 |
| MOLC | ASIR | Both | Honduras | 1.69 (1.54, 1.84) | <0.001 |
| MOLC | ASIR | Both | Kenya | 1.66 (1.42, 1.9) | <0.001 |
| MOLC | ASIR | Both | Chad | 1.6 (1.46, 1.74) | <0.001 |
| MOLC | ASIR | Male | Egypt | 3.33 (2.82, 3.84) | <0.001 |
| MOLC | ASIR | Male | Lesotho | 2.72 (2.3, 3.14) | <0.001 |
| MOLC | ASIR | Male | Mozambique | 1.7 (1.44, 1.96) | <0.001 |
| MOLC | ASIR | Male | Honduras | 1.42 (1.18, 1.66) | <0.001 |
| MOLC | ASIR | Male | Dominican Republic | 1.36 (1.21, 1.52) | <0.001 |
| MOLC | ASIR | Male | Kenya | 1.36 (1.01, 1.71) | <0.001 |
| MOLC | ASIR | Female | France | 5.17 (4.78, 5.57) | <0.001 |
| MOLC | ASIR | Female | Spain | 4.97 (4.62, 5.32) | <0.001 |
| MOLC | ASIR | Female | Egypt | 3.9 (3.47, 4.33) | <0.001 |
| MOLC | ASIR | Female | Lesotho | 3.47 (2.93, 4.02) | <0.001 |
| MOLC | ASIR | Female | Hungary | 3.12 (2.82, 3.41) | <0.001 |
| MOLC | ASMR | Both | Egypt | 3.53 (3.04, 4.03) | <0.001 |
| MOLC | ASMR | Both | Lesotho | 3.14 (2.69, 3.59) | <0.001 |
| MOLC | ASMR | Both | Honduras | 1.65 (1.5, 1.81) | <0.001 |
| MOLC | ASMR | Both | Kenya | 1.64 (1.4, 1.89) | <0.001 |
| MOLC | ASMR | Both | Chad | 1.6 (1.46, 1.74) | <0.001 |
| MOLC | ASMR | Male | Egypt | 3.32 (2.81, 3.83) | <0.001 |
| MOLC | ASMR | Male | Lesotho | 2.71 (2.29, 3.13) | <0.001 |
| MOLC | ASMR | Male | Mozambique | 1.68 (1.42, 1.94) | <0.001 |
| MOLC | ASMR | Male | Honduras | 1.39 (1.15, 1.63) | <0.001 |
| MOLC | ASMR | Male | Kenya | 1.35 (0.99, 1.7) | <0.001 |
| MOLC | ASMR | Female | Spain | 4.28 (3.94, 4.63) | <0.001 |
| MOLC | ASMR | Female | France | 3.96 (3.62, 4.31) | <0.001 |
| MOLC | ASMR | Female | Egypt | 3.88 (3.45, 4.32) | <0.001 |
| MOLC | ASMR | Female | Lesotho | 3.46 (2.91, 4) | <0.001 |
| MOLC | ASMR | Female | Bulgaria | 2.94 (2.65, 3.23) | <0.001 |
| MOLC | ASDR | Both | Egypt | 3.46 (2.96, 3.96) | <0.001 |
| MOLC | ASDR | Both | Lesotho | 3.2 (2.72, 3.69) | <0.001 |
| MOLC | ASDR | Both | Kenya | 1.64 (1.38, 1.89) | <0.001 |
| MOLC | ASDR | Both | Honduras | 1.6 (1.45, 1.75) | <0.001 |
| MOLC | ASDR | Both | Chad | 1.57 (1.43, 1.71) | <0.001 |
| MOLC | ASDR | Male | Egypt | 3.26 (2.75, 3.78) | <0.001 |
| MOLC | ASDR | Male | Lesotho | 2.78 (2.34, 3.22) | <0.001 |
| MOLC | ASDR | Male | Mozambique | 1.72 (1.45, 1.99) | <0.001 |
| MOLC | ASDR | Male | Kenya | 1.34 (0.98, 1.71) | <0.001 |
| MOLC | ASDR | Male | Honduras | 1.31 (1.08, 1.54) | <0.001 |
| MOLC | ASDR | Female | Spain | 4.41 (4.06, 4.77) | <0.001 |
| MOLC | ASDR | Female | France | 4.08 (3.7, 4.46) | <0.001 |
| MOLC | ASDR | Female | Egypt | 3.77 (3.34, 4.2) | <0.001 |
| MOLC | ASDR | Female | Lesotho | 3.48 (2.93, 4.04) | <0.001 |
| MOLC | ASDR | Female | Bulgaria | 3.01 (2.73, 3.29) | <0.001 |
| LOLC | ASIR | Both | Egypt | 5.27 (4.56, 5.99) | <0.001 |
| LOLC | ASIR | Both | United Arab Emirates | 2.8 (2.06, 3.55) | <0.001 |
| LOLC | ASIR | Both | Georgia | 2.55 (2, 3.11) | <0.001 |
| LOLC | ASIR | Both | Romania | 2.37 (2.18, 2.57) | <0.001 |
| LOLC | ASIR | Both | Honduras | 2.2 (2, 2.41) | <0.001 |
| LOLC | ASIR | Male | Egypt | 4.84 (4.14, 5.54) | <0.001 |
| LOLC | ASIR | Male | Georgia | 2.48 (1.83, 3.14) | <0.001 |
| LOLC | ASIR | Male | Romania | 2.41 (2.19, 2.63) | <0.001 |
| LOLC | ASIR | Male | Paraguay | 2.19 (1.89, 2.48) | <0.001 |
| LOLC | ASIR | Male | Honduras | 2.04 (1.73, 2.34) | <0.001 |
| LOLC | ASIR | Female | United Arab Emirates | 7.9 (6.89, 8.92) | <0.001 |
| LOLC | ASIR | Female | Egypt | 6.21 (5.49, 6.93) | <0.001 |
| LOLC | ASIR | Female | Netherlands | 4.17 (3.77, 4.56) | <0.001 |
| LOLC | ASIR | Female | Norway | 3.89 (3.62, 4.17) | <0.001 |
| LOLC | ASIR | Female | Equatorial Guinea | 3.65 (3.39, 3.92) | <0.001 |
| LOLC | ASMR | Both | Egypt | 5.28 (4.55, 6) | <0.001 |
| LOLC | ASMR | Both | United Arab Emirates | 2.75 (2, 3.5) | <0.001 |
| LOLC | ASMR | Both | Georgia | 2.56 (1.99, 3.13) | <0.001 |
| LOLC | ASMR | Both | Honduras | 2.16 (1.95, 2.36) | <0.001 |
| LOLC | ASMR | Both | Romania | 2.15 (1.96, 2.35) | <0.001 |
| LOLC | ASMR | Male | Egypt | 4.85 (4.15, 5.56) | <0.001 |
| LOLC | ASMR | Male | Georgia | 2.49 (1.82, 3.16) | <0.001 |
| LOLC | ASMR | Male | Romania | 2.17 (1.95, 2.39) | <0.001 |
| LOLC | ASMR | Male | Paraguay | 2.14 (1.85, 2.43) | <0.001 |
| LOLC | ASMR | Male | Honduras | 1.97 (1.67, 2.27) | <0.001 |
| LOLC | ASMR | Female | United Arab Emirates | 7.81 (6.81, 8.82) | <0.001 |
| LOLC | ASMR | Female | Egypt | 6.2 (5.48, 6.93) | <0.001 |
| LOLC | ASMR | Female | Equatorial Guinea | 3.61 (3.34, 3.88) | <0.001 |
| LOLC | ASMR | Female | Netherlands | 3.53 (3.18, 3.89) | <0.001 |
| LOLC | ASMR | Female | Norway | 3.16 (2.94, 3.38) | <0.001 |
| LOLC | ASDR | Both | Egypt | 5.13 (4.42, 5.83) | <0.001 |
| LOLC | ASDR | Both | United Arab Emirates | 2.76 (2.02, 3.5) | <0.001 |
| LOLC | ASDR | Both | Georgia | 2.51 (1.98, 3.04) | <0.001 |
| LOLC | ASDR | Both | Honduras | 2.18 (1.97, 2.38) | <0.001 |
| LOLC | ASDR | Both | Paraguay | 2.08 (1.8, 2.36) | <0.001 |
| LOLC | ASDR | Both | Romania | 2.08 (1.89, 2.28) | <0.001 |
| LOLC | ASDR | Male | Egypt | 4.7 (4.02, 5.39) | <0.001 |
| LOLC | ASDR | Male | Georgia | 2.44 (1.81, 3.08) | <0.001 |
| LOLC | ASDR | Male | Romania | 2.1 (1.88, 2.32) | <0.001 |
| LOLC | ASDR | Male | Paraguay | 2.06 (1.76, 2.37) | <0.001 |
| LOLC | ASDR | Male | Honduras | 2.03 (1.73, 2.34) | <0.001 |
| LOLC | ASDR | Female | United Arab Emirates | 7.85 (6.82, 8.88) | <0.001 |
| LOLC | ASDR | Female | Egypt | 6.02 (5.31, 6.72) | <0.001 |
| LOLC | ASDR | Female | Netherlands | 3.53 (3.16, 3.9) | <0.001 |
| LOLC | ASDR | Female | Equatorial Guinea | 3.48 (3.22, 3.75) | <0.001 |
| LOLC | ASDR | Female | Lesotho | 3.11 (2.72, 3.49) | <0.001 |

Abbreviations: EAPC, estimated annual percentage change; ASIR, age- standardized incidence rate; ASMR, age-standardized mortality rate; ASDR, age-standardized disability-adjusted life year rate; EOLC, early-onset lung cancer; MOLC, middle-onset lung cancer; LOLC, late-onset lung cancer.

# **Supplementary Table S5.** Incidence number and ASIR of EOLC, MOLC and LOLC in 1990 and 2021, and AAPC from 1990 to 2021 among 204 countries and territories.

| Country/Territory | Cases (95%UI), 1990 | ASIR (95% UI), 1990 | Cases (95%UI), 2021 | ASIR (95% UI), 2021 | AAPC (95% CI), 1990-2021 | P value |
| --- | --- | --- | --- | --- | --- | --- |
| EOLC |  |  |  |  |  |  |
| Afghanistan | 63 (31, 100) | 1.86 (0.87, 3.21) | 263 (156, 390) | 2.33 (1.32, 3.72) | 0.76 (0.65, 0.87) | <0.001 |
| Albania | 50 (40, 62) | 3.97 (2.97, 5.1) | 49 (34, 67) | 3.82 (2.62, 5.26) | -0.32 (-1.05, 0.42) | 0.398 |
| Algeria | 91 (68, 116) | 1.23 (0.84, 1.75) | 208 (148, 290) | 0.92 (0.59, 1.36) | -0.94 (-1.02, -0.85) | <0.001 |
| American Samoa | 1 (1, 1) | 4.72 (3.27, 6.68) | 1 (1, 2) | 4.73 (3.19, 6.74) | 0.03 (-0.21, 0.27) | 0.804 |
| Andorra | 2 (1, 3) | 6.96 (4.47, 10.5) | 2 (2, 4) | 4.03 (2.36, 6.22) | -1.92 (-2.24, -1.59) | <0.001 |
| Angola | 63 (41, 91) | 1.99 (1.21, 3.1) | 193 (132, 271) | 1.86 (1.17, 2.78) | -0.2 (-0.65, 0.25) | 0.375 |
| Antigua and Barbuda | 0 (0, 0) | 1.58 (1.35, 1.85) | 1 (0, 1) | 1.04 (0.88, 1.21) | -0.76 (-1.63, 0.12) | 0.089 |
| Argentina | 1080 (975, 1188) | 7.3 (6.17, 8.6) | 600 (523, 678) | 2.51 (2.06, 3.01) | -3.42 (-3.93, -2.9) | <0.001 |
| Armenia | 117 (109, 125) | 9.6 (8.94, 10.27) | 56 (49, 64) | 3.66 (3.19, 4.18) | -2.94 (-3.57, -2.31) | <0.001 |
| Australia | 398 (353, 441) | 4.6 (4.01, 5.22) | 417 (362, 487) | 3.09 (2.55, 3.73) | -1.28 (-1.8, -0.76) | <0.001 |
| Austria | 268 (242, 293) | 6.46 (5.6, 7.46) | 142 (123, 162) | 2.93 (2.42, 3.55) | -2.63 (-3.27, -2) | <0.001 |
| Azerbaijan | 151 (122, 185) | 6.45 (5.02, 8.05) | 173 (116, 246) | 3.13 (2.11, 4.4) | -2.32 (-3.1, -1.52) | <0.001 |
| Bahamas | 4 (3, 4) | 3.61 (3.06, 4.24) | 7 (5, 9) | 3.26 (2.41, 4.36) | -0.37 (-0.8, 0.06) | 0.09 |
| Bahrain | 7 (5, 8) | 3.31 (2.41, 4.4) | 22 (17, 29) | 2.04 (1.41, 2.86) | -1.52 (-1.85, -1.19) | <0.001 |
| Bangladesh | 443 (314, 640) | 1.33 (0.83, 2.08) | 791 (548, 1231) | 1 (0.65, 1.62) | -0.9 (-1.23, -0.57) | <0.001 |
| Barbados | 2 (2, 2) | 1.8 (1.55, 2.06) | 2 (2, 3) | 1.42 (1.05, 1.89) | -0.67 (-1.59, 0.26) | 0.156 |
| Belarus | 353 (304, 406) | 7.92 (6.66, 9.33) | 194 (145, 247) | 3.67 (2.68, 4.78) | -2.39 (-3.37, -1.4) | <0.001 |
| Belgium | 426 (386, 466) | 8.78 (7.53, 10.13) | 242 (213, 274) | 4.09 (3.41, 4.88) | -2.67 (-2.99, -2.35) | <0.001 |
| Belize | 1 (1, 1) | 1.62 (1.39, 1.86) | 4 (4, 5) | 2.21 (1.81, 2.7) | 1.12 (0.24, 2) | 0.012 |
| Benin | 10 (8, 13) | 0.77 (0.51, 1.09) | 32 (21, 46) | 0.72 (0.44, 1.14) | -0.24 (-0.39, -0.1) | 0.001 |
| Bermuda | 2 (2, 2) | 6.32 (5.15, 7.63) | 2 (1, 2) | 4.19 (3.11, 5.44) | -1.46 (-1.82, -1.09) | <0.001 |
| Bhutan | 2 (1, 3) | 0.97 (0.5, 1.52) | 4 (2, 6) | 0.96 (0.57, 1.56) | -0.07 (-0.14, 0) | 0.038 |
| Bolivia (Plurinational State of) | 55 (41, 71) | 2.42 (1.67, 3.36) | 100 (67, 143) | 1.8 (1.15, 2.67) | -0.92 (-1, -0.84) | <0.001 |
| Bosnia and Herzegovina | 138 (105, 174) | 6.74 (5.15, 8.56) | 85 (55, 119) | 4.55 (2.94, 6.35) | -1.37 (-2.06, -0.68) | <0.001 |
| Botswana | 11 (7, 16) | 3.01 (1.79, 4.69) | 29 (19, 40) | 2.41 (1.47, 3.58) | -0.71 (-1.16, -0.26) | 0.002 |
| Brazil | 1715 (1653, 1788) | 2.94 (2.77, 3.11) | 2483 (2357, 2606) | 2.05 (1.92, 2.18) | -1.18 (-1.5, -0.86) | <0.001 |
| Brunei Darussalam | 5 (3, 6) | 4.31 (2.96, 6.09) | 10 (8, 13) | 3.67 (2.65, 4.9) | -0.52 (-1.02, -0.01) | 0.045 |
| Bulgaria | 427 (373, 486) | 9.33 (7.85, 10.95) | 265 (211, 325) | 6.49 (5.1, 8.02) | -1.15 (-2.12, -0.18) | 0.021 |
| Burkina Faso | 18 (13, 27) | 0.66 (0.42, 1.07) | 51 (34, 76) | 0.7 (0.43, 1.15) | 0.21 (-0.02, 0.45) | 0.074 |
| Burundi | 20 (14, 30) | 1.34 (0.85, 2.13) | 39 (26, 60) | 0.95 (0.55, 1.55) | -1.1 (-1.38, -0.82) | <0.001 |
| Cabo Verde | 2 (2, 2) | 2.09 (1.46, 2.88) | 5 (4, 7) | 1.94 (1.27, 2.95) | -0.3 (-0.79, 0.2) | 0.244 |
| Cambodia | 116 (85, 160) | 3.67 (2.44, 5.37) | 264 (181, 379) | 3.5 (2.27, 5.24) | -0.15 (-0.28, -0.03) | 0.016 |
| Cameroon | 31 (24, 39) | 1 (0.7, 1.4) | 119 (75, 170) | 1.08 (0.62, 1.64) | 0.22 (0.1, 0.34) | <0.001 |
| Canada | 1084 (987, 1183) | 7.57 (6.68, 8.56) | 479 (411, 560) | 2.53 (2.09, 3.06) | -3.55 (-3.8, -3.29) | <0.001 |
| Central African Republic | 18 (10, 34) | 2.12 (1.17, 4.15) | 38 (19, 76) | 1.9 (0.93, 3.98) | -0.4 (-0.69, -0.11) | 0.007 |
| Chad | 10 (7, 14) | 0.57 (0.37, 0.89) | 38 (26, 56) | 0.78 (0.48, 1.23) | 1.19 (1.08, 1.29) | <0.001 |
| Chile | 184 (156, 216) | 3.19 (2.67, 3.79) | 179 (145, 220) | 1.82 (1.43, 2.26) | -1.71 (-2.58, -0.84) | <0.001 |
| China | 35890 (30753, 41673) | 6.95 (5.85, 8.11) | 52901 (41336, 65169) | 6.51 (5.1, 8.05) | -0.21 (-0.37, -0.05) | 0.009 |
| Colombia | 334 (304, 366) | 2.63 (2.26, 3.02) | 340 (274, 416) | 1.38 (1.06, 1.74) | -2.12 (-2.86, -1.38) | <0.001 |
| Comoros | 2 (1, 2) | 1.19 (0.71, 1.83) | 4 (3, 6) | 1.23 (0.74, 1.88) | -0.22 (-1.59, 1.18) | 0.758 |
| Congo | 20 (12, 27) | 2.89 (1.67, 4.33) | 54 (34, 81) | 2.2 (1.34, 3.43) | -0.86 (-1.49, -0.22) | 0.008 |
| Cook Islands | 0 (0, 0) | 4.7 (3.1, 6.91) | 0 (0, 1) | 4.49 (2.9, 6.7) | -0.16 (-0.26, -0.05) | 0.005 |
| Costa Rica | 16 (14, 18) | 1.44 (1.23, 1.69) | 30 (26, 36) | 1.24 (1.01, 1.52) | -0.78 (-1.67, 0.12) | 0.088 |
| Côte d'Ivoire | 18 (14, 24) | 0.51 (0.34, 0.73) | 54 (36, 81) | 0.52 (0.31, 0.85) | -2.16 (-3.09, -1.23) | <0.001 |
| Croatia | 230 (203, 258) | 9.16 (7.7, 10.69) | 111 (90, 135) | 4.85 (3.89, 5.91) | -1.7 (-2.11, -1.29) | <0.001 |
| Cuba | 329 (298, 359) | 6.37 (5.47, 7.36) | 234 (189, 283) | 3.8 (3.01, 4.69) | 0.5 (-0.03, 1.03) | 0.066 |
| Cyprus | 8 (6, 11) | 2.13 (1.45, 2.99) | 20 (15, 26) | 2.48 (1.7, 3.44) | -3.86 (-4.4, -3.31) | <0.001 |
| Czechia | 569 (496, 645) | 9.86 (8.41, 11.43) | 192 (155, 233) | 2.87 (2.3, 3.53) | 0.08 (-0.22, 0.38) | 0.605 |
| Democratic People's Republic of Korea | 544 (369, 802) | 5.51 (3.52, 8.37) | 828 (504, 1318) | 5.46 (3.11, 9.03) | -0.01 (-0.11, 0.09) | 0.897 |
| Democratic Republic of the Congo | 142 (89, 229) | 1.32 (0.78, 2.24) | 369 (216, 655) | 1.22 (0.65, 2.29) | -0.28 (-0.47, -0.09) | 0.005 |
| Denmark | 243 (223, 262) | 8.17 (7.18, 9.21) | 98 (85, 113) | 3.29 (2.74, 3.92) | -2.86 (-3.26, -2.46) | <0.001 |
| Djibouti | 2 (1, 3) | 1.29 (0.77, 2.03) | 10 (6, 15) | 1.58 (0.88, 2.65) | 0.62 (0.46, 0.78) | <0.001 |
| Dominica | 1 (0, 1) | 2.39 (1.79, 3.14) | 1 (1, 1) | 2.88 (1.89, 4.19) | 0.58 (0.34, 0.83) | <0.001 |
| Dominican Republic | 59 (48, 72) | 2.36 (1.8, 3.1) | 165 (114, 228) | 3.07 (2.04, 4.38) | 0.97 (0.17, 1.78) | 0.017 |
| Ecuador | 67 (60, 73) | 1.77 (1.5, 2.08) | 122 (92, 158) | 1.4 (1.02, 1.89) | -0.72 (-2.25, 0.84) | 0.365 |
| Egypt | 365 (303, 445) | 1.7 (1.27, 2.25) | 1182 (912, 1522) | 2.51 (1.79, 3.44) | 1.19 (0.83, 1.55) | <0.001 |
| El Salvador | 33 (28, 39) | 1.81 (1.44, 2.24) | 56 (42, 72) | 1.91 (1.37, 2.54) | 0.1 (-0.9, 1.11) | 0.847 |
| Equatorial Guinea | 3 (2, 4) | 2 (1.12, 3.53) | 11 (7, 18) | 2.25 (1.29, 3.7) | 0.46 (-0.37, 1.29) | 0.281 |
| Eritrea | 17 (12, 25) | 1.6 (1.03, 2.46) | 46 (29, 71) | 1.79 (1.08, 2.83) | 0.34 (0.14, 0.55) | 0.001 |
| Estonia | 64 (57, 72) | 8.28 (7.05, 9.75) | 18 (15, 22) | 2.57 (2.02, 3.15) | -4.04 (-4.66, -3.42) | <0.001 |
| Eswatini | 6 (4, 11) | 2.86 (1.57, 5.04) | 20 (10, 32) | 4.51 (2.2, 7.57) | 1.53 (1.18, 1.88) | <0.001 |
| Ethiopia | 333 (227, 488) | 2.29 (1.51, 3.5) | 425 (332, 531) | 1.16 (0.86, 1.49) | -2.17 (-2.29, -2.06) | <0.001 |
| Fiji | 6 (4, 8) | 1.81 (1.2, 2.66) | 7 (4, 10) | 1.47 (0.91, 2.25) | -0.68 (-0.84, -0.51) | <0.001 |
| Finland | 134 (122, 147) | 4.58 (3.91, 5.37) | 73 (64, 82) | 2.75 (2.28, 3.36) | -1.34 (-2.22, -0.45) | 0.003 |
| France | 2316 (2066, 2601) | 8.29 (7.17, 9.55) | 2421 (2134, 2765) | 7.19 (6.08, 8.45) | -0.45 (-0.63, -0.27) | <0.001 |
| Gabon | 8 (5, 11) | 2.62 (1.45, 4.21) | 18 (12, 27) | 2.44 (1.42, 3.86) | -0.13 (-0.38, 0.12) | 0.316 |
| Gambia | 1 (1, 2) | 0.41 (0.26, 0.62) | 3 (2, 5) | 0.42 (0.26, 0.68) | 0.14 (-1.11, 1.4) | 0.833 |
| Georgia | 177 (154, 204) | 7.51 (6.47, 8.69) | 81 (68, 95) | 4.39 (3.67, 5.17) | -1.68 (-3.1, -0.25) | 0.021 |
| Germany | 3112 (2795, 3453) | 7.47 (6.41, 8.61) | 1453 (1281, 1628) | 3.54 (2.99, 4.14) | -2.43 (-2.81, -2.04) | <0.001 |
| Ghana | 32 (23, 42) | 0.65 (0.43, 0.97) | 72 (51, 99) | 0.53 (0.34, 0.79) | -0.69 (-0.79, -0.59) | <0.001 |
| Greece | 389 (354, 426) | 7.53 (6.62, 8.51) | 259 (234, 286) | 4.35 (3.8, 4.98) | -1.74 (-2.01, -1.47) | <0.001 |
| Greenland | 5 (4, 6) | 17.29 (12.72, 22.94) | 2 (1, 3) | 8.28 (5.72, 11.41) | -2.26 (-2.45, -2.06) | <0.001 |
| Grenada | 1 (1, 1) | 2.45 (1.94, 3.02) | 1 (1, 1) | 1.92 (1.49, 2.45) | -0.71 (-1.74, 0.34) | 0.184 |
| Guam | 3 (3, 3) | 4.94 (3.86, 6.26) | 5 (4, 6) | 6.62 (5.17, 8.4) | 0.95 (0.26, 1.66) | 0.007 |
| Guatemala | 50 (47, 53) | 1.89 (1.67, 2.12) | 83 (70, 97) | 1.22 (1, 1.47) | -1.38 (-2.49, -0.27) | 0.015 |
| Guinea | 18 (14, 24) | 0.96 (0.65, 1.38) | 50 (33, 73) | 1.21 (0.73, 1.88) | 0.75 (0.57, 0.93) | <0.001 |
| Guinea-Bissau | 4 (2, 5) | 1.26 (0.76, 1.97) | 9 (6, 12) | 1.27 (0.78, 1.93) | 0.04 (-0.06, 0.14) | 0.461 |
| Guyana | 4 (3, 4) | 1.41 (1.11, 1.76) | 6 (4, 8) | 1.69 (1.17, 2.34) | 0.74 (0.23, 1.24) | 0.004 |
| Haiti | 55 (34, 79) | 2.58 (1.53, 3.88) | 106 (64, 164) | 1.87 (1.08, 3.13) | -0.91 (-1.03, -0.79) | <0.001 |
| Honduras | 39 (31, 49) | 2.7 (1.93, 3.76) | 113 (68, 175) | 2.68 (1.44, 4.41) | -0.02 (-0.23, 0.19) | 0.835 |
| Hungary | 786 (688, 891) | 13.93 (11.98, 16.08) | 293 (234, 358) | 4.79 (3.81, 5.87) | -3.44 (-4.09, -2.79) | <0.001 |
| Iceland | 6 (5, 7) | 5.31 (4.48, 6.23) | 11 (9, 13) | 5.93 (4.8, 7.32) | 0.29 (-0.63, 1.22) | 0.54 |
| India | 3917 (3407, 4551) | 1.17 (1, 1.36) | 9657 (8149, 11180) | 1.38 (1.15, 1.63) | 0.53 (0.24, 0.82) | <0.001 |
| Indonesia | 2111 (1654, 2529) | 2.94 (2.28, 3.58) | 5114 (3551, 7078) | 3.26 (2.22, 4.49) | 0.36 (0.29, 0.43) | <0.001 |
| Iran (Islamic Republic of) | 345 (275, 409) | 1.93 (1.54, 2.33) | 932 (838, 1050) | 1.83 (1.63, 2.09) | -0.24 (-0.38, -0.09) | 0.001 |
| Iraq | 132 (92, 187) | 2.44 (1.56, 3.66) | 397 (271, 569) | 2.06 (1.31, 3.09) | -0.57 (-0.76, -0.39) | <0.001 |
| Ireland | 80 (72, 88) | 4.95 (4.2, 5.77) | 106 (91, 122) | 3.7 (3.02, 4.5) | -1 (-2.07, 0.07) | 0.068 |
| Israel | 79 (69, 88) | 3.93 (3.35, 4.61) | 108 (93, 124) | 2.3 (1.93, 2.71) | -1.48 (-1.79, -1.17) | <0.001 |
| Italy | 1779 (1688, 1863) | 6.11 (5.73, 6.49) | 978 (911, 1048) | 2.83 (2.57, 3.12) | -2.45 (-2.64, -2.27) | <0.001 |
| Jamaica | 26 (22, 29) | 3.36 (2.69, 4.11) | 37 (26, 52) | 2.64 (1.76, 3.82) | -0.77 (-2.82, 1.31) | 0.466 |
| Japan | 2831 (2727, 2922) | 3.76 (3.58, 3.94) | 1683 (1574, 1800) | 2.38 (2.16, 2.63) | -1.55 (-1.97, -1.12) | <0.001 |
| Jordan | 25 (19, 32) | 2.39 (1.62, 3.33) | 102 (76, 139) | 1.7 (1.15, 2.51) | -1.01 (-1.37, -0.65) | <0.001 |
| Kazakhstan | 660 (589, 735) | 10.73 (9.43, 12.08) | 251 (218, 285) | 2.58 (2.23, 2.94) | -4.45 (-4.88, -4.02) | <0.001 |
| Kenya | 20 (13, 29) | 0.33 (0.22, 0.47) | 98 (75, 130) | 0.52 (0.4, 0.7) | 1.51 (1.36, 1.66) | <0.001 |
| Kiribati | 1 (1, 1) | 2.81 (1.89, 4.07) | 2 (1, 3) | 3.45 (2.11, 5.4) | 0.66 (0.56, 0.76) | <0.001 |
| Kuwait | 12 (10, 14) | 1.64 (1.31, 1.99) | 34 (27, 44) | 0.95 (0.71, 1.27) | -1.62 (-2.24, -0.99) | <0.001 |
| Kyrgyzstan | 96 (82, 110) | 7.14 (5.96, 8.46) | 63 (49, 78) | 2.04 (1.59, 2.56) | -4.23 (-4.48, -3.98) | <0.001 |
| Lao People's Democratic Republic | 54 (34, 86) | 4.02 (2.34, 6.65) | 109 (72, 159) | 3.23 (2.02, 5.05) | -0.68 (-0.78, -0.58) | <0.001 |
| Latvia | 105 (92, 122) | 7.73 (6.49, 9.15) | 32 (25, 39) | 3.19 (2.49, 3.97) | -2.96 (-4.19, -1.71) | <0.001 |
| Lebanon | 41 (29, 55) | 3.46 (2.34, 4.98) | 80 (59, 105) | 2.82 (1.95, 3.9) | -0.56 (-0.83, -0.29) | <0.001 |
| Lesotho | 8 (5, 13) | 1.66 (1.01, 2.79) | 34 (21, 52) | 4.85 (2.77, 8.1) | 3.58 (3.3, 3.85) | <0.001 |
| Liberia | 5 (4, 7) | 0.69 (0.45, 1.05) | 16 (9, 28) | 0.7 (0.36, 1.27) | 0.07 (-0.23, 0.36) | 0.654 |
| Libya | 45 (32, 62) | 3.54 (2.27, 5.42) | 167 (115, 243) | 3.75 (2.37, 5.78) | 0.18 (0.03, 0.32) | 0.016 |
| Lithuania | 135 (120, 150) | 7.51 (6.36, 8.81) | 51 (42, 62) | 3.6 (2.79, 4.46) | -2.48 (-3.49, -1.46) | <0.001 |
| Luxembourg | 15 (14, 17) | 7.37 (6.43, 8.4) | 12 (11, 14) | 3.23 (2.71, 3.82) | -2.69 (-3.07, -2.31) | <0.001 |
| Madagascar | 36 (28, 46) | 1.08 (0.75, 1.49) | 101 (71, 140) | 0.99 (0.61, 1.49) | -0.23 (-0.33, -0.12) | <0.001 |
| Malawi | 9 (7, 12) | 0.33 (0.22, 0.48) | 28 (20, 40) | 0.45 (0.28, 0.69) | 0.96 (0.71, 1.22) | <0.001 |
| Malaysia | 146 (117, 182) | 2.2 (1.62, 2.95) | 445 (350, 570) | 2.82 (2.07, 3.78) | 0.92 (0.11, 1.74) | 0.026 |
| Maldives | 1 (1, 2) | 1.98 (1.09, 3.01) | 3 (2, 4) | 0.97 (0.64, 1.39) | -2.42 (-2.6, -2.23) | <0.001 |
| Mali | 16 (13, 20) | 0.61 (0.43, 0.82) | 43 (30, 61) | 0.63 (0.41, 0.93) | 0.14 (-0.05, 0.34) | 0.144 |
| Malta | 6 (5, 7) | 2.96 (2.41, 3.6) | 8 (6, 9) | 3.14 (2.5, 3.94) | 0.13 (-0.54, 0.8) | 0.703 |
| Marshall Islands | 1 (0, 1) | 4.25 (2.65, 6.64) | 2 (1, 2) | 5.52 (3.03, 8.71) | 0.84 (0.73, 0.95) | <0.001 |
| Mauritania | 6 (4, 8) | 0.94 (0.6, 1.39) | 12 (8, 17) | 0.79 (0.49, 1.24) | -0.59 (-0.78, -0.41) | <0.001 |
| Mauritius | 11 (9, 12) | 2.24 (1.95, 2.57) | 12 (11, 14) | 1.78 (1.51, 2.05) | -0.94 (-2.57, 0.72) | 0.265 |
| Mexico | 620 (602, 639) | 2.02 (1.93, 2.09) | 807 (713, 913) | 1.18 (1.04, 1.34) | -1.75 (-2.43, -1.05) | <0.001 |
| Micronesia (Federated States of) | 2 (1, 3) | 5.29 (3.22, 8.46) | 3 (2, 4) | 5.9 (3.41, 9.34) | 0.37 (0.3, 0.44) | <0.001 |
| Monaco | 2 (1, 2) | 10.11 (7.26, 13.94) | 3 (2, 4) | 14.92 (9.6, 21.8) | 1.26 (1.14, 1.38) | <0.001 |
| Mongolia | 31 (24, 40) | 4.7 (3.26, 6.53) | 68 (52, 86) | 3.94 (2.75, 5.46) | -0.63 (-1.49, 0.24) | 0.157 |
| Montenegro | 27 (21, 33) | 9.77 (7.57, 12.26) | 28 (21, 36) | 8.17 (6.04, 10.72) | -0.61 (-1.17, -0.04) | 0.035 |
| Morocco | 144 (106, 194) | 1.68 (1.1, 2.49) | 243 (161, 373) | 1.28 (0.78, 2.03) | -0.87 (-0.94, -0.81) | <0.001 |
| Mozambique | 21 (15, 28) | 0.49 (0.32, 0.7) | 72 (47, 107) | 0.77 (0.46, 1.24) | 1.53 (1.38, 1.67) | <0.001 |
| Myanmar | 541 (362, 747) | 3.73 (2.35, 5.57) | 772 (537, 1112) | 2.76 (1.79, 4.08) | -0.96 (-1.05, -0.88) | <0.001 |
| Namibia | 4 (3, 5) | 0.94 (0.65, 1.3) | 11 (7, 16) | 1.08 (0.65, 1.64) | 0.54 (0.14, 0.95) | 0.009 |
| Nauru | 0 (0, 0) | 7.69 (3.7, 11.98) | 0 (0, 1) | 8.12 (3.81, 12.09) | 0.17 (0.1, 0.25) | <0.001 |
| Nepal | 73 (48, 107) | 1.08 (0.65, 1.71) | 140 (96, 206) | 1.03 (0.64, 1.61) | -0.13 (-0.33, 0.08) | 0.22 |
| Netherlands | 575 (532, 619) | 7.14 (6.28, 8.13) | 365 (326, 411) | 4.28 (3.66, 5.01) | -1.6 (-1.84, -1.37) | <0.001 |
| New Zealand | 75 (67, 82) | 4.49 (3.97, 5.06) | 82 (72, 92) | 3.18 (2.74, 3.65) | -1.19 (-1.65, -0.72) | <0.001 |
| Nicaragua | 12 (10, 14) | 1.03 (0.77, 1.32) | 25 (20, 32) | 0.81 (0.56, 1.12) | -1.05 (-1.47, -0.62) | <0.001 |
| Niger | 12 (8, 19) | 0.52 (0.33, 0.84) | 29 (18, 50) | 0.45 (0.26, 0.82) | -0.45 (-0.68, -0.21) | <0.001 |
| Nigeria | 50 (35, 68) | 0.17 (0.12, 0.24) | 134 (89, 183) | 0.18 (0.12, 0.25) | 0.05 (-0.06, 0.16) | 0.386 |
| Niue | 0 (0, 0) | 3.66 (2.31, 5.63) | 0 (0, 0) | 4.18 (2.55, 6.56) | 0.43 (0.12, 0.74) | 0.007 |
| North Macedonia | 63 (49, 77) | 6.61 (5.06, 8.32) | 61 (44, 82) | 4.61 (3.31, 6.15) | -0.98 (-1.5, -0.47) | <0.001 |
| Northern Mariana Islands | 2 (1, 3) | 7.58 (4.69, 11.46) | 1 (1, 2) | 5.25 (3.64, 7.51) | -1.2 (-1.38, -1.02) | <0.001 |
| Norway | 79 (75, 84) | 3.6 (3.33, 3.88) | 76 (69, 83) | 2.61 (2.3, 2.96) | -0.9 (-1.27, -0.54) | <0.001 |
| Oman | 5 (3, 8) | 0.75 (0.44, 1.18) | 12 (8, 17) | 0.42 (0.27, 0.65) | -2.03 (-2.44, -1.62) | <0.001 |
| Pakistan | 682 (536, 835) | 1.89 (1.42, 2.45) | 2195 (1638, 2926) | 2.29 (1.62, 3.15) | 0.61 (0.49, 0.74) | <0.001 |
| Palau | 1 (0, 1) | 7.7 (5.11, 11.35) | 1 (1, 1) | 8.09 (5.61, 11.4) | 0.14 (-0.19, 0.48) | 0.406 |
| Palestine | 17 (12, 24) | 3.08 (1.92, 4.72) | 49 (39, 63) | 2.42 (1.71, 3.34) | -0.83 (-1.12, -0.53) | <0.001 |
| Panama | 19 (18, 21) | 2.04 (1.78, 2.3) | 26 (20, 32) | 1.25 (0.96, 1.58) | -1.47 (-2.42, -0.51) | 0.003 |
| Papua New Guinea | 36 (22, 62) | 2.5 (1.35, 4.67) | 117 (73, 188) | 2.61 (1.42, 4.57) | 0.15 (0.06, 0.24) | 0.002 |
| Paraguay | 22 (18, 27) | 1.58 (1.17, 2.11) | 62 (45, 84) | 1.88 (1.27, 2.69) | 0.77 (0.42, 1.11) | <0.001 |
| Peru | 226 (185, 278) | 2.79 (2.08, 3.62) | 372 (259, 495) | 2.03 (1.37, 2.86) | -0.98 (-2.78, 0.85) | 0.29 |
| Philippines | 799 (710, 897) | 3.52 (3.08, 4.02) | 1569 (1282, 1844) | 2.97 (2.42, 3.55) | -0.59 (-0.71, -0.47) | <0.001 |
| Poland | 1666 (1608, 1726) | 9.57 (9.14, 10) | 706 (643, 771) | 3.11 (2.81, 3.41) | -3.78 (-4.12, -3.44) | <0.001 |
| Portugal | 212 (190, 238) | 4.38 (3.7, 5.19) | 184 (161, 210) | 2.89 (2.41, 3.43) | -1.29 (-2.02, -0.57) | 0.001 |
| Puerto Rico | 53 (47, 60) | 3.08 (2.59, 3.66) | 37 (29, 46) | 2.2 (1.64, 2.85) | -1.26 (-1.43, -1.09) | <0.001 |
| Qatar | 5 (4, 7) | 2.11 (1.41, 3.05) | 27 (20, 38) | 1.24 (0.8, 1.88) | -1.78 (-3.08, -0.46) | 0.008 |
| Republic of Korea | 1002 (830, 1188) | 4.8 (3.73, 6.07) | 876 (715, 1087) | 2.77 (2.12, 3.62) | -1.8 (-2.04, -1.56) | <0.001 |
| Republic of Moldova | 143 (128, 158) | 7.39 (6.39, 8.5) | 64 (54, 74) | 2.9 (2.38, 3.49) | -2.96 (-4.51, -1.39) | <0.001 |
| Romania | 907 (771, 1059) | 8.4 (7.02, 10.01) | 616 (494, 745) | 5.38 (4.28, 6.58) | -1.39 (-1.81, -0.97) | <0.001 |
| Russian Federation | 5382 (5242, 5514) | 8.36 (8.07, 8.64) | 3022 (2735, 3278) | 3.62 (3.28, 3.94) | -2.61 (-3.39, -1.83) | <0.001 |
| Rwanda | 30 (21, 41) | 1.61 (1.04, 2.37) | 57 (37, 87) | 1.16 (0.67, 1.87) | -1.08 (-1.39, -0.77) | <0.001 |
| Saint Kitts and Nevis | 0 (0, 0) | 2.33 (1.98, 2.71) | 0 (0, 1) | 1.26 (0.91, 1.67) | -1.93 (-2.22, -1.64) | <0.001 |
| Saint Lucia | 1 (1, 1) | 2.91 (2.52, 3.36) | 2 (2, 3) | 2.22 (1.72, 2.81) | -0.88 (-1.41, -0.36) | 0.001 |
| Saint Vincent and the Grenadines | 1 (1, 1) | 1.99 (1.68, 2.31) | 1 (1, 2) | 2.09 (1.7, 2.55) | 0.12 (-0.2, 0.44) | 0.47 |
| Samoa | 1 (0, 1) | 1.15 (0.75, 1.72) | 1 (1, 2) | 1.34 (0.82, 2.01) | 0.48 (0.39, 0.58) | <0.001 |
| San Marino | 1 (1, 1) | 5.43 (3.94, 7.18) | 1 (0, 1) | 3.2 (1.66, 5.19) | -1.88 (-2.04, -1.73) | <0.001 |
| Sao Tome and Principe | 1 (0, 1) | 1.62 (1.07, 2.31) | 2 (1, 3) | 1.96 (1.13, 3.29) | 0.54 (-0.06, 1.15) | 0.076 |
| Saudi Arabia | 55 (38, 74) | 0.97 (0.61, 1.46) | 266 (183, 372) | 0.99 (0.63, 1.48) | 0.07 (-0.08, 0.22) | 0.341 |
| Senegal | 20 (15, 28) | 0.94 (0.62, 1.4) | 52 (37, 74) | 0.94 (0.58, 1.47) | 0.05 (-0.24, 0.33) | 0.742 |
| Serbia | 453 (354, 585) | 9.42 (6.91, 12.65) | 326 (244, 421) | 6.43 (4.58, 8.66) | -1.13 (-1.54, -0.71) | <0.001 |
| Seychelles | 1 (1, 1) | 3.47 (2.52, 4.75) | 1 (1, 2) | 2.39 (1.68, 3.29) | -1.08 (-1.66, -0.49) | <0.001 |
| Sierra Leone | 10 (7, 14) | 0.75 (0.48, 1.18) | 24 (15, 36) | 0.78 (0.45, 1.25) | 0.13 (-0.07, 0.33) | 0.191 |
| Singapore | 60 (53, 67) | 3.65 (3.1, 4.28) | 77 (66, 91) | 2.04 (1.62, 2.55) | -1.74 (-2.66, -0.81) | <0.001 |
| Slovakia | 223 (175, 275) | 8.93 (6.49, 11.72) | 123 (91, 159) | 3.62 (2.48, 4.92) | -2.92 (-3.67, -2.16) | <0.001 |
| Slovenia | 73 (64, 83) | 7.17 (6.13, 8.29) | 34 (26, 44) | 2.91 (2.21, 3.76) | -2.72 (-3.56, -1.89) | <0.001 |
| Solomon Islands | 5 (3, 7) | 4.58 (2.27, 7.5) | 16 (11, 23) | 5.58 (3.65, 8.14) | 0.65 (0.53, 0.76) | <0.001 |
| Somalia | 25 (15, 46) | 1.03 (0.56, 2.02) | 66 (35, 140) | 0.98 (0.47, 2.15) | -0.17 (-0.26, -0.07) | <0.001 |
| South Africa | 737 (625, 867) | 5.56 (4.58, 6.76) | 984 (855, 1131) | 3.48 (2.93, 4.14) | -1.5 (-1.97, -1.02) | <0.001 |
| South Sudan | 18 (11, 27) | 1.11 (0.62, 1.8) | 42 (28, 63) | 1.23 (0.76, 1.92) | 0.4 (0, 0.8) | 0.052 |
| Spain | 1371 (1238, 1513) | 7.47 (6.38, 8.71) | 1037 (891, 1195) | 3.53 (2.92, 4.18) | -2.47 (-2.73, -2.22) | <0.001 |
| Sri Lanka | 134 (106, 171) | 1.77 (1.28, 2.48) | 179 (113, 255) | 1.55 (0.91, 2.34) | -0.36 (-1, 0.27) | 0.26 |
| Sudan | 104 (69, 161) | 1.59 (0.97, 2.66) | 278 (169, 424) | 1.63 (0.93, 2.59) | 0.08 (-0.04, 0.2) | 0.216 |
| Suriname | 4 (3, 5) | 2.57 (1.79, 3.47) | 8 (6, 11) | 2.74 (1.81, 3.96) | 0.32 (-1.18, 1.84) | 0.678 |
| Sweden | 159 (144, 174) | 3.2 (2.8, 3.66) | 80 (69, 93) | 1.56 (1.27, 1.9) | -2.42 (-2.93, -1.91) | <0.001 |
| Switzerland | 214 (191, 238) | 5.42 (4.58, 6.35) | 140 (121, 158) | 2.88 (2.35, 3.5) | -2.13 (-3.01, -1.24) | <0.001 |
| Syrian Arab Republic | 118 (87, 155) | 3.14 (2.16, 4.35) | 171 (116, 242) | 2.26 (1.47, 3.27) | -1.16 (-1.33, -1) | <0.001 |
| Taiwan (Province of China) | 411 (373, 453) | 4.57 (3.96, 5.24) | 686 (592, 785) | 4.7 (3.9, 5.58) | 0.04 (-0.31, 0.4) | 0.805 |
| Tajikistan | 65 (49, 83) | 4.51 (3.34, 6) | 78 (49, 121) | 1.8 (1.11, 2.84) | -2.95 (-3.14, -2.76) | <0.001 |
| Thailand | 1266 (1028, 1551) | 5.2 (3.83, 6.91) | 2149 (1572, 2897) | 5.42 (3.74, 7.7) | 0.14 (-0.27, 0.56) | 0.499 |
| Timor-Leste | 5 (3, 8) | 1.87 (1.13, 2.93) | 10 (7, 15) | 2.02 (1.28, 3.01) | 0.29 (-0.16, 0.74) | 0.206 |
| Togo | 9 (6, 12) | 0.85 (0.54, 1.27) | 33 (19, 50) | 0.99 (0.54, 1.65) | 0.52 (0.15, 0.89) | 0.006 |
| Tokelau | 0 (0, 0) | 3.08 (1.97, 4.72) | 0 (0, 0) | 3.83 (2.42, 5.84) | 0.77 (0.59, 0.94) | <0.001 |
| Tonga | 1 (1, 2) | 3.93 (2.68, 5.57) | 2 (1, 3) | 4.35 (2.66, 6.71) | 0.32 (0.11, 0.53) | 0.003 |
| Trinidad and Tobago | 11 (10, 12) | 2.21 (1.92, 2.54) | 16 (11, 21) | 2.03 (1.47, 2.71) | -0.12 (-1.17, 0.94) | 0.823 |
| Tunisia | 68 (52, 86) | 2.43 (1.67, 3.42) | 177 (116, 250) | 2.66 (1.62, 4.03) | 0.28 (0.12, 0.44) | 0.001 |
| Turkey | 2120 (1546, 2802) | 9.9 (6.7, 13.96) | 2191 (1653, 2783) | 4.64 (3.24, 6.31) | -2.53 (-2.89, -2.17) | <0.001 |
| Turkmenistan | 44 (40, 48) | 4.02 (3.57, 4.47) | 59 (44, 79) | 2.4 (1.8, 3.21) | -1.45 (-2.52, -0.38) | 0.008 |
| Tuvalu | 0 (0, 0) | 3.72 (2.27, 5.97) | 0 (0, 0) | 4.49 (2.75, 6.9) | 0.62 (0.56, 0.69) | <0.001 |
| Uganda | 37 (27, 50) | 0.84 (0.57, 1.22) | 141 (93, 198) | 1.11 (0.68, 1.67) | 0.9 (0.73, 1.06) | <0.001 |
| Ukraine | 2623 (2343, 2959) | 10.68 (9.08, 12.58) | 1086 (702, 1541) | 4.24 (2.74, 6.18) | -3.07 (-3.65, -2.49) | <0.001 |
| United Arab Emirates | 23 (15, 34) | 2.5 (1.52, 3.85) | 99 (68, 151) | 1.04 (0.67, 1.63) | -2.81 (-3.25, -2.36) | <0.001 |
| United Kingdom | 1531 (1501, 1562) | 5.22 (5.09, 5.35) | 976 (944, 1006) | 2.83 (2.71, 2.94) | -2.03 (-2.44, -1.61) | <0.001 |
| United Republic of Tanzania | 76 (56, 108) | 1.07 (0.73, 1.61) | 233 (159, 331) | 1.14 (0.73, 1.76) | 0.2 (0.03, 0.36) | 0.023 |
| United States of America | 10365 (10108, 10601) | 8.18 (7.93, 8.43) | 4494 (4342, 4657) | 2.76 (2.66, 2.88) | -3.46 (-3.69, -3.23) | <0.001 |
| United States Virgin Islands | 2 (2, 3) | 3.51 (2.53, 4.73) | 1 (1, 2) | 3.54 (2.31, 5.3) | 0.13 (-0.21, 0.47) | 0.449 |
| Uruguay | 126 (111, 142) | 8.92 (7.54, 10.4) | 88 (73, 105) | 5.02 (4.08, 6.08) | -2.01 (-2.29, -1.73) | <0.001 |
| Uzbekistan | 263 (237, 292) | 4.32 (3.71, 4.96) | 269 (222, 325) | 1.56 (1.24, 1.95) | -3.16 (-4.91, -1.37) | 0.001 |
| Vanuatu | 1 (1, 3) | 2.91 (1.47, 5.62) | 4 (3, 7) | 3.28 (1.84, 5.8) | 0.36 (0.24, 0.49) | <0.001 |
| Venezuela (Bolivarian Republic of) | 258 (239, 279) | 3.76 (3.31, 4.23) | 382 (278, 509) | 2.63 (1.88, 3.58) | -1.15 (-1.58, -0.73) | <0.001 |
| Viet Nam | 595 (427, 800) | 3.07 (2.03, 4.41) | 2133 (1533, 2946) | 3.87 (2.56, 5.73) | 0.76 (0.68, 0.85) | <0.001 |
| Yemen | 67 (37, 121) | 1.85 (0.98, 3.37) | 210 (118, 362) | 1.69 (0.9, 2.99) | -0.33 (-0.53, -0.13) | 0.001 |
| Zambia | 28 (22, 36) | 1.3 (0.92, 1.82) | 133 (83, 269) | 2.01 (1.15, 4.24) | 1.42 (1.26, 1.57) | <0.001 |
| Zimbabwe | 45 (35, 56) | 1.62 (1.15, 2.19) | 162 (108, 236) | 2.79 (1.78, 4.23) | 1.84 (1.3, 2.38) | <0.001 |
| MOLC |  |  |  |  |  |  |
| Afghanistan | 362 (194, 657) | 27.74 (14.1, 52.2) | 433 (275, 628) | 28.57 (17.3, 42.74) | 0.08 (0.03, 0.13) | 0.001 |
| Albania | 337 (278, 407) | 94.29 (76.7, 114.47) | 519 (364, 698) | 71.04 (50.05, 95.59) | -1.02 (-1.79, -0.25) | 0.01 |
| Algeria | 489 (389, 603) | 22.99 (16.63, 31) | 1189 (886, 1531) | 18.87 (12.96, 26.48) | -0.66 (-0.96, -0.35) | <0.001 |
| American Samoa | 3 (3, 4) | 80.06 (61.68, 103.13) | 6 (5, 8) | 72.27 (54.93, 94.38) | -0.29 (-0.35, -0.23) | <0.001 |
| Andorra | 14 (10, 20) | 147.8 (98.44, 215.94) | 21 (13, 29) | 85.23 (52.15, 124.35) | -1.87 (-2.31, -1.43) | <0.001 |
| Angola | 317 (215, 463) | 43.31 (27.39, 65.14) | 861 (625, 1151) | 38.53 (26.21, 53.86) | -0.38 (-0.69, -0.08) | 0.014 |
| Antigua and Barbuda | 2 (2, 3) | 33.79 (29.06, 39.09) | 5 (5, 6) | 27.73 (23.66, 32.39) | -0.6 (-1.8, 0.61) | 0.329 |
| Argentina | 5440 (4985, 5937) | 102.23 (91.28, 113.97) | 5754 (5090, 6479) | 68.28 (59.4, 77.79) | -1.19 (-1.34, -1.03) | <0.001 |
| Armenia | 761 (720, 802) | 143.08 (134.26, 151.95) | 683 (600, 776) | 89.18 (78.13, 101.48) | -1.66 (-2.55, -0.76) | <0.001 |
| Australia | 3780 (3563, 4000) | 123.74 (113.73, 134.24) | 5027 (4565, 5462) | 79.76 (70.18, 89.76) | -1.41 (-1.68, -1.14) | <0.001 |
| Austria | 1801 (1709, 1896) | 104.01 (94.38, 114.71) | 2315 (2143, 2525) | 94.33 (83.37, 107.09) | -0.31 (-0.71, 0.1) | 0.141 |
| Azerbaijan | 846 (690, 1016) | 87.81 (71.6, 106.02) | 1107 (751, 1535) | 51.71 (35.14, 71.88) | -1.74 (-2.14, -1.34) | <0.001 |
| Bahamas | 15 (13, 16) | 59.45 (50.95, 68.83) | 32 (25, 41) | 42.54 (32.72, 54.67) | -1.13 (-2.18, -0.07) | 0.036 |
| Bahrain | 30 (25, 36) | 94.35 (73.81, 118.54) | 80 (63, 106) | 40.44 (29.44, 56.62) | -2.67 (-3.1, -2.25) | <0.001 |
| Bangladesh | 2213 (1575, 3204) | 28.75 (19.22, 43.7) | 4185 (3082, 5654) | 17.99 (12.41, 25.66) | -1.34 (-1.73, -0.94) | <0.001 |
| Barbados | 11 (10, 12) | 29.11 (24.94, 33.3) | 22 (17, 29) | 26.63 (19.44, 34.89) | 0.04 (-0.1, 0.18) | 0.539 |
| Belarus | 3005 (2762, 3247) | 129.06 (115.92, 143.25) | 2512 (1934, 3132) | 92.8 (71.15, 116.58) | -0.99 (-1.61, -0.38) | 0.002 |
| Belgium | 4102 (3873, 4343) | 170.53 (154.8, 186.9) | 3538 (3254, 3833) | 113.06 (99.28, 127.64) | -1.47 (-1.69, -1.24) | <0.001 |
| Belize | 5 (4, 5) | 32.21 (27.9, 37.27) | 19 (16, 22) | 35.66 (29.3, 42.5) | 0.47 (-0.14, 1.09) | 0.133 |
| Benin | 61 (49, 75) | 19.27 (14.1, 26.17) | 159 (114, 215) | 18.15 (12.46, 26.22) | -0.17 (-0.44, 0.11) | 0.239 |
| Bermuda | 12 (11, 13) | 118.48 (103.01, 135.54) | 16 (13, 20) | 80.83 (63.31, 102.83) | -1.48 (-1.88, -1.09) | <0.001 |
| Bhutan | 7 (4, 11) | 16.5 (8.95, 26.15) | 16 (10, 22) | 15.88 (9.76, 23.81) | -0.07 (-0.2, 0.06) | 0.281 |
| Bolivia (Plurinational State of) | 243 (178, 318) | 44.9 (31.7, 60.29) | 535 (358, 750) | 34.09 (22.53, 49.24) | -0.88 (-0.94, -0.81) | <0.001 |
| Bosnia and Herzegovina | 1078 (951, 1215) | 131.89 (110.72, 156.25) | 1313 (1012, 1634) | 130.52 (98.18, 166.86) | 0.04 (-0.43, 0.52) | 0.856 |
| Botswana | 56 (40, 76) | 54.96 (36.04, 79.44) | 125 (88, 168) | 46.56 (30.28, 66.2) | -0.56 (-0.75, -0.38) | <0.001 |
| Brazil | 7946 (7668, 8201) | 51.75 (49.27, 54.32) | 18758 (17779, 19669) | 44.63 (41.7, 47.43) | -0.48 (-0.66, -0.3) | <0.001 |
| Brunei Darussalam | 17 (13, 22) | 106.31 (79.28, 143.06) | 42 (34, 51) | 62.78 (48.2, 80.2) | -1.74 (-2.32, -1.15) | <0.001 |
| Bulgaria | 2490 (2225, 2769) | 112.6 (100.56, 125.68) | 2721 (2274, 3209) | 139.73 (115.01, 166.04) | 0.76 (0.19, 1.34) | 0.009 |
| Burkina Faso | 125 (94, 178) | 16.34 (11.12, 24.89) | 267 (187, 401) | 17.02 (11.22, 26.01) | 0.14 (-0.04, 0.32) | 0.131 |
| Burundi | 100 (71, 138) | 26.13 (17.66, 38.14) | 160 (108, 243) | 17.87 (11.17, 28.24) | -1.21 (-1.39, -1.04) | <0.001 |
| Cabo Verde | 9 (7, 11) | 27.25 (20.26, 35.32) | 35 (26, 45) | 44.42 (30.54, 60.53) | 1.56 (1, 2.13) | <0.001 |
| Cambodia | 632 (451, 858) | 79.04 (53.26, 113.17) | 1675 (1154, 2269) | 73.03 (49.36, 102.59) | -0.24 (-0.35, -0.12) | <0.001 |
| Cameroon | 180 (146, 221) | 22.4 (16.68, 29.73) | 544 (369, 767) | 24.43 (15.56, 35.43) | 0.26 (0.14, 0.38) | <0.001 |
| Canada | 8991 (8475, 9483) | 179.76 (165.2, 194.38) | 10101 (9269, 10960) | 93.51 (82.41, 105.36) | -2.07 (-2.19, -1.96) | <0.001 |
| Central African Republic | 95 (61, 168) | 43.18 (25.74, 80.61) | 156 (94, 292) | 35.58 (20.2, 69.74) | -0.62 (-0.75, -0.49) | <0.001 |
| Chad | 64 (46, 92) | 14.1 (9.52, 21.37) | 214 (150, 317) | 21.29 (13.94, 32.19) | 1.37 (1.26, 1.47) | <0.001 |
| Chile | 940 (881, 996) | 56.97 (50.87, 63.33) | 1685 (1545, 1833) | 40.65 (35.51, 46.23) | -1.11 (-1.33, -0.89) | <0.001 |
| China | 153736 (128763, 179233) | 101 (84.2, 118.46) | 430878 (340964, 531986) | 114.13 (89.84, 141) | 0.39 (0.2, 0.58) | <0.001 |
| Colombia | 1379 (1293, 1466) | 46.57 (42.27, 51.37) | 2564 (2077, 3144) | 28.21 (22.44, 34.91) | -1.97 (-2.08, -1.86) | <0.001 |
| Comoros | 8 (6, 11) | 23.51 (15.66, 33.3) | 18 (12, 26) | 21.72 (13.54, 32.25) | -0.29 (-0.48, -0.1) | 0.002 |
| Congo | 98 (58, 125) | 50.17 (29.83, 69.73) | 212 (155, 283) | 42 (28.24, 59.35) | -0.53 (-0.78, -0.28) | <0.001 |
| Cook Islands | 2 (2, 3) | 93.78 (70.15, 125.04) | 3 (3, 4) | 77.12 (56.42, 102.11) | -0.59 (-0.79, -0.39) | <0.001 |
| Costa Rica | 92 (85, 98) | 33.31 (29.66, 37.18) | 199 (171, 229) | 21.7 (18.25, 25.69) | -1.38 (-1.84, -0.92) | <0.001 |
| Côte d'Ivoire | 98 (78, 122) | 13.29 (9.73, 18.35) | 249 (175, 367) | 12.43 (8.21, 18.86) | -0.14 (-0.25, -0.03) | 0.013 |
| Croatia | 1887 (1707, 2074) | 160.81 (144.27, 178.03) | 1835 (1579, 2086) | 143.93 (122.88, 165.5) | 0.58 (0.39, 0.78) | <0.001 |
| Cuba | 1454 (1364, 1552) | 92.88 (83.7, 102.7) | 3308 (2807, 3867) | 109.03 (89.48, 131.01) | -0.03 (-0.52, 0.45) | 0.894 |
| Cyprus | 86 (67, 107) | 66.84 (49.37, 89.18) | 205 (164, 245) | 65.55 (50.5, 84.01) | -2.13 (-2.54, -1.71) | <0.001 |
| Czechia | 4047 (3796, 4310) | 182.63 (167.48, 199.11) | 2857 (2482, 3230) | 97.06 (81.69, 113.41) | -0.22 (-0.41, -0.03) | 0.022 |
| Democratic People's Republic of Korea | 2467 (1717, 3417) | 81.12 (53.83, 116.46) | 4672 (3004, 6755) | 81.39 (50.99, 120.48) | 0.01 (-0.06, 0.08) | 0.731 |
| Democratic Republic of the Congo | 841 (537, 1408) | 28.11 (17.37, 47.69) | 1811 (1099, 3212) | 25.99 (15, 47.39) | -0.25 (-0.38, -0.12) | <0.001 |
| Denmark | 2099 (1957, 2242) | 191.83 (176.69, 208.07) | 1720 (1582, 1867) | 110.73 (98.2, 124.71) | -1.8 (-2.06, -1.55) | <0.001 |
| Djibouti | 7 (4, 9) | 26.37 (16.69, 38.91) | 39 (24, 58) | 32.11 (19.14, 50.38) | 0.64 (0.58, 0.69) | <0.001 |
| Dominica | 5 (4, 5) | 51.64 (41.08, 64.14) | 8 (6, 11) | 54.73 (39.73, 73.52) | 0.19 (0, 0.38) | 0.047 |
| Dominican Republic | 194 (163, 234) | 31.2 (23.7, 40.32) | 679 (465, 921) | 41.74 (26.7, 60.5) | 1.02 (0.06, 1.99) | 0.037 |
| Ecuador | 211 (194, 230) | 24.68 (22.19, 27.42) | 492 (372, 637) | 18.65 (13.86, 24.53) | -0.92 (-1.87, 0.04) | 0.062 |
| Egypt | 960 (788, 1159) | 18.57 (14.43, 23.61) | 5066 (3869, 6531) | 40 (29.19, 52.99) | 2.6 (1.98, 3.21) | <0.001 |
| El Salvador | 116 (99, 135) | 24.27 (19.78, 29.1) | 241 (184, 306) | 25.88 (19.16, 34.26) | 0.38 (-0.44, 1.21) | 0.364 |
| Equatorial Guinea | 15 (9, 24) | 41.37 (23.96, 72.3) | 39 (24, 57) | 43.43 (26.12, 66.74) | 0.21 (-0.18, 0.6) | 0.29 |
| Eritrea | 60 (45, 86) | 26.8 (18.94, 39.39) | 140 (103, 186) | 26.97 (18.45, 37.98) | 0 (-0.15, 0.15) | 0.991 |
| Estonia | 535 (502, 568) | 154.83 (140.64, 169.21) | 284 (239, 326) | 77.9 (64.58, 91.33) | -2.24 (-2.42, -2.06) | <0.001 |
| Eswatini | 29 (18, 48) | 58.42 (34.33, 97.64) | 69 (39, 106) | 68.83 (38.27, 107.24) | 0.55 (0.21, 0.9) | 0.002 |
| Ethiopia | 1413 (989, 2033) | 39.39 (27.32, 57.84) | 1435 (1116, 1815) | 19.94 (15.4, 25.4) | -2.18 (-2.28, -2.09) | <0.001 |
| Fiji | 20 (16, 26) | 31.33 (23.27, 41.92) | 40 (29, 54) | 26.94 (18.75, 37.66) | -0.5 (-0.74, -0.27) | <0.001 |
| Finland | 1158 (1084, 1235) | 107.94 (97.36, 118.74) | 1088 (959, 1219) | 68.23 (58.4, 79.76) | -1.57 (-1.82, -1.32) | <0.001 |
| France | 14671 (13509, 15750) | 118.89 (107.35, 130.76) | 24337 (21802, 26741) | 139.74 (121.35, 159.1) | 0.59 (0.44, 0.74) | <0.001 |
| Gabon | 50 (29, 71) | 49.9 (28.98, 74.3) | 100 (70, 134) | 49.74 (32.69, 69.95) | 0.01 (-0.22, 0.24) | 0.928 |
| Gambia | 6 (4, 8) | 9.81 (6.77, 13.87) | 16 (11, 22) | 10.03 (6.65, 14.56) | 0.09 (-0.83, 1.02) | 0.841 |
| Georgia | 1143 (1016, 1274) | 100.61 (89.19, 113.09) | 845 (720, 989) | 88.37 (75.25, 103.27) | -0.65 (-1.37, 0.08) | 0.079 |
| Germany | 24610 (22964, 26303) | 128.76 (116.37, 141.67) | 28726 (26343, 31313) | 112.74 (99.39, 127.03) | -0.43 (-0.67, -0.19) | 0.001 |
| Ghana | 160 (124, 207) | 14.43 (9.87, 19.95) | 486 (348, 637) | 16.34 (11.01, 23.06) | 0.42 (0.28, 0.56) | <0.001 |
| Greece | 3254 (3046, 3471) | 133.37 (119.58, 148.35) | 3850 (3583, 4142) | 134.89 (120.12, 150.55) | 0.04 (-0.27, 0.36) | 0.779 |
| Greenland | 21 (18, 24) | 341.34 (279.96, 406.54) | 29 (23, 35) | 197.46 (152.26, 247.89) | -1.61 (-1.84, -1.38) | <0.001 |
| Grenada | 4 (4, 5) | 43.48 (35.99, 51.78) | 7 (6, 8) | 34.75 (27.9, 42.93) | -0.77 (-1.33, -0.21) | 0.007 |
| Guam | 15 (13, 17) | 105.27 (84.39, 129.76) | 30 (25, 36) | 84 (65.4, 104.23) | -0.81 (-1.42, -0.19) | 0.01 |
| Guatemala | 138 (127, 149) | 22.35 (19.64, 25.4) | 259 (215, 312) | 14.35 (11.57, 17.54) | -1.82 (-2.24, -1.39) | <0.001 |
| Guinea | 109 (85, 139) | 19.35 (13.79, 26.43) | 233 (161, 328) | 24.22 (15.48, 35.96) | 0.74 (0.61, 0.87) | <0.001 |
| Guinea-Bissau | 20 (14, 28) | 28.72 (18.12, 43.87) | 35 (26, 47) | 26.75 (18.07, 38.38) | -0.22 (-0.3, -0.14) | <0.001 |
| Guyana | 16 (13, 18) | 24.45 (19.99, 29.27) | 29 (21, 39) | 24.18 (17.32, 32.35) | 0.16 (-0.5, 0.83) | 0.634 |
| Haiti | 270 (171, 409) | 45.65 (28.05, 73.19) | 444 (271, 718) | 33.79 (20.16, 55.42) | -0.94 (-0.98, -0.89) | <0.001 |
| Honduras | 129 (104, 159) | 37.46 (29.07, 48.21) | 612 (426, 869) | 57.07 (38.17, 82.89) | 1.41 (1.08, 1.75) | <0.001 |
| Hungary | 4256 (3869, 4653) | 176.01 (158.58, 193.78) | 4960 (4216, 5658) | 181.24 (153.75, 208) | 0.07 (-0.3, 0.45) | 0.698 |
| Iceland | 50 (46, 54) | 117.99 (104.48, 133.28) | 81 (71, 91) | 94.03 (78.86, 110.19) | -0.86 (-1.61, -0.1) | 0.027 |
| India | 14664 (12733, 16961) | 17.22 (14.78, 20.12) | 41849 (34128, 48687) | 20.26 (16.45, 23.68) | 0.51 (0.07, 0.95) | 0.024 |
| Indonesia | 9198 (6865, 10892) | 51.17 (38.05, 61.67) | 29680 (20008, 38713) | 63.95 (43.17, 84.21) | 0.71 (0.61, 0.81) | <0.001 |
| Iran (Islamic Republic of) | 1490 (1193, 1809) | 28.82 (23.03, 35.14) | 3505 (3197, 3848) | 25.64 (23.08, 28.49) | -0.44 (-0.55, -0.32) | <0.001 |
| Iraq | 614 (445, 832) | 48.49 (32.82, 69.64) | 2131 (1450, 2790) | 50.44 (33.16, 69.93) | 0.14 (-0.03, 0.31) | 0.107 |
| Ireland | 784 (728, 843) | 128.77 (115.28, 142.62) | 943 (839, 1050) | 81.79 (69.09, 96.43) | -1.3 (-1.65, -0.96) | <0.001 |
| Israel | 571 (534, 608) | 77.56 (70.2, 85.03) | 1043 (949, 1145) | 60.41 (52.81, 68.8) | -0.76 (-1.03, -0.48) | <0.001 |
| Italy | 19147 (18564, 19735) | 136.04 (129.89, 142.97) | 14002 (13160, 14791) | 79.34 (72.93, 86.66) | -1.78 (-1.96, -1.6) | <0.001 |
| Jamaica | 150 (139, 162) | 58.4 (51.53, 66.09) | 265 (198, 357) | 52.92 (38.27, 71.69) | -0.32 (-0.97, 0.33) | 0.323 |
| Japan | 21710 (20944, 22401) | 77.05 (73.13, 81.2) | 23273 (21633, 24337) | 65 (59.29, 69.16) | -0.58 (-0.8, -0.35) | <0.001 |
| Jordan | 108 (83, 138) | 43.35 (30.84, 59.21) | 415 (305, 551) | 29.73 (20.92, 41.81) | -1.15 (-1.41, -0.9) | <0.001 |
| Kazakhstan | 3886 (3589, 4197) | 168.48 (150.76, 186.6) | 1939 (1644, 2264) | 57.65 (48.81, 67.34) | -3.33 (-3.85, -2.8) | <0.001 |
| Kenya | 94 (63, 136) | 6.72 (4.44, 9.78) | 435 (343, 554) | 10.55 (8.2, 13.46) | 1.44 (1.27, 1.6) | <0.001 |
| Kiribati | 3 (2, 3) | 43.4 (32.37, 56.91) | 7 (5, 9) | 47.86 (32.82, 67.84) | 0.34 (0.28, 0.4) | <0.001 |
| Kuwait | 47 (41, 52) | 44.71 (38.14, 51.85) | 100 (79, 123) | 18.43 (14.14, 23.63) | -2.82 (-5.11, -0.46) | 0.019 |
| Kyrgyzstan | 557 (502, 615) | 102.75 (90.42, 116.07) | 354 (290, 424) | 38.28 (31.09, 46.25) | -3.27 (-3.85, -2.68) | <0.001 |
| Lao People's Democratic Republic | 300 (198, 463) | 80.97 (49.4, 130.61) | 529 (386, 742) | 63.54 (42.24, 92.17) | -0.77 (-0.85, -0.68) | <0.001 |
| Latvia | 886 (820, 953) | 146.87 (132.51, 162.42) | 491 (412, 562) | 90.64 (74.16, 108.16) | -1.6 (-2.43, -0.76) | <0.001 |
| Lebanon | 305 (217, 393) | 75.01 (51.01, 103.98) | 520 (404, 662) | 60.62 (44.23, 80.46) | -0.62 (-0.87, -0.37) | <0.001 |
| Lesotho | 53 (37, 80) | 37.44 (24.52, 59.87) | 151 (103, 216) | 78.9 (50.04, 117.56) | 2.5 (2.28, 2.71) | <0.001 |
| Liberia | 32 (25, 43) | 17.01 (11.9, 24.13) | 57 (36, 90) | 15.9 (9.25, 26.32) | -0.3 (-0.49, -0.1) | 0.003 |
| Libya | 220 (160, 294) | 67.88 (46.86, 95.3) | 647 (453, 907) | 70.1 (46.39, 101.7) | 0.03 (-0.27, 0.33) | 0.837 |
| Lithuania | 1045 (973, 1111) | 135.75 (122.43, 150.28) | 728 (613, 842) | 90.22 (74.28, 107.07) | -1.38 (-1.92, -0.84) | <0.001 |
| Luxembourg | 123 (114, 132) | 145.53 (129.98, 162.85) | 153 (136, 172) | 95.89 (82.79, 110.85) | -1.39 (-2, -0.77) | <0.001 |
| Madagascar | 179 (145, 218) | 19.8 (14.83, 26.38) | 363 (258, 491) | 16.86 (11.39, 23.53) | -0.51 (-0.66, -0.37) | <0.001 |
| Malawi | 48 (38, 62) | 7.01 (5.07, 9.65) | 106 (75, 146) | 8.29 (5.47, 12.15) | 0.56 (0.37, 0.75) | <0.001 |
| Malaysia | 788 (664, 977) | 51.27 (38.97, 67.38) | 2694 (2226, 3155) | 53.57 (40.61, 68.41) | 0.31 (0.02, 0.6) | 0.035 |
| Maldives | 7 (5, 11) | 42.68 (27.27, 63.95) | 11 (8, 14) | 18.09 (12.47, 24.85) | -2.98 (-3.36, -2.59) | <0.001 |
| Mali | 101 (82, 126) | 13.9 (10.24, 18.6) | 227 (163, 308) | 14.48 (9.47, 21.02) | 0.17 (0.02, 0.33) | 0.028 |
| Malta | 62 (58, 67) | 90.15 (79.48, 101.59) | 82 (73, 91) | 66.13 (55.97, 76.78) | -1.15 (-1.59, -0.71) | <0.001 |
| Marshall Islands | 2 (1, 3) | 74.92 (45.18, 117.65) | 5 (3, 8) | 80.33 (47.04, 121.6) | 0.25 (0.1, 0.4) | 0.001 |
| Mauritania | 37 (27, 48) | 22.75 (15.55, 31.36) | 77 (53, 106) | 20.52 (13.4, 29.81) | -0.37 (-0.5, -0.23) | <0.001 |
| Mauritius | 56 (52, 60) | 43.1 (37.83, 48.81) | 100 (91, 109) | 29.92 (25.88, 33.94) | -1.19 (-2.07, -0.31) | 0.008 |
| Mexico | 2654 (2585, 2728) | 37.6 (36.27, 38.93) | 4012 (3495, 4528) | 18.75 (16.29, 21.34) | -2.38 (-2.62, -2.14) | <0.001 |
| Micronesia (Federated States of) | 7 (5, 9) | 83.06 (56.14, 121.18) | 14 (9, 19) | 90 (57.7, 132.34) | 0.26 (0.18, 0.33) | <0.001 |
| Monaco | 16 (13, 20) | 183.52 (138.52, 234.95) | 28 (23, 34) | 226.34 (177.03, 289.51) | 0.67 (0.53, 0.8) | <0.001 |
| Mongolia | 192 (146, 249) | 109.95 (83.47, 142.69) | 308 (235, 400) | 70.69 (53.42, 91.33) | -1.48 (-1.8, -1.15) | <0.001 |
| Montenegro | 185 (157, 216) | 166.12 (135.54, 199.93) | 280 (221, 342) | 168.81 (131.58, 211.04) | 0.15 (-0.28, 0.58) | 0.505 |
| Morocco | 992 (793, 1252) | 41.58 (29.74, 56.5) | 2748 (1947, 3508) | 44.2 (28.8, 61.16) | 0.21 (0.1, 0.31) | <0.001 |
| Mozambique | 156 (127, 190) | 14.6 (10.89, 19.23) | 383 (276, 510) | 19.28 (13.12, 27.15) | 0.91 (0.78, 1.05) | <0.001 |
| Myanmar | 3121 (2121, 4625) | 74.18 (48.06, 112.79) | 4852 (3484, 6524) | 54.31 (37.17, 76.4) | -1 (-1.08, -0.93) | <0.001 |
| Namibia | 21 (17, 26) | 17.61 (12.77, 23.42) | 48 (34, 63) | 19.46 (13.35, 27.25) | 0.36 (0.13, 0.58) | 0.002 |
| Nauru | 1 (1, 1) | 122.31 (63.84, 183.99) | 1 (1, 2) | 120.32 (64.97, 173.28) | -0.06 (-0.13, 0.01) | 0.116 |
| Nepal | 327 (217, 477) | 19.02 (11.88, 29.19) | 693 (506, 942) | 17.08 (11.25, 24.67) | -0.34 (-0.49, -0.19) | <0.001 |
| Netherlands | 4864 (4586, 5129) | 164.63 (151.75, 178.28) | 5520 (5038, 5967) | 112.38 (100.47, 125.65) | -1.24 (-1.47, -1.01) | <0.001 |
| New Zealand | 764 (716, 813) | 127.21 (114.84, 140.24) | 1023 (937, 1110) | 82.04 (72.74, 92.29) | -1.42 (-1.67, -1.17) | <0.001 |
| Nicaragua | 41 (35, 48) | 16.3 (12.98, 20.15) | 118 (94, 149) | 14.43 (10.71, 19.05) | -0.42 (-0.73, -0.11) | 0.009 |
| Niger | 67 (47, 103) | 13.36 (8.63, 21.19) | 179 (118, 306) | 12.18 (7.41, 20.9) | -0.26 (-0.47, -0.04) | 0.02 |
| Nigeria | 316 (215, 430) | 4.16 (2.81, 5.73) | 666 (508, 870) | 4.09 (3.1, 5.31) | -0.06 (-0.16, 0.03) | 0.202 |
| Niue | 0 (0, 0) | 67.07 (47.42, 91.24) | 0 (0, 0) | 77.71 (52.3, 108.5) | 0.46 (0.36, 0.57) | <0.001 |
| North Macedonia | 376 (313, 446) | 109.5 (88.95, 133.08) | 667 (497, 852) | 115.33 (85.76, 149.02) | 0.11 (-0.16, 0.39) | 0.426 |
| Northern Mariana Islands | 4 (3, 5) | 132.44 (99.14, 175.52) | 11 (9, 12) | 102.42 (79.87, 128.61) | -0.84 (-0.93, -0.74) | <0.001 |
| Norway | 836 (804, 871) | 94.83 (89.35, 100.56) | 1099 (1028, 1174) | 80.12 (72.56, 88.06) | -0.55 (-0.78, -0.33) | <0.001 |
| Oman | 22 (14, 31) | 20.48 (12.98, 30.65) | 45 (32, 64) | 12.86 (8.77, 18.45) | -1.49 (-2.13, -0.84) | <0.001 |
| Pakistan | 3654 (2962, 4371) | 39.39 (30.31, 49.76) | 9347 (6857, 12623) | 43.39 (31.1, 59.71) | 0.31 (0.2, 0.42) | <0.001 |
| Palau | 2 (2, 3) | 130.67 (93.25, 180.04) | 5 (4, 7) | 114.98 (80.66, 154.94) | -0.43 (-0.6, -0.26) | <0.001 |
| Palestine | 83 (58, 111) | 55.9 (37.08, 80.87) | 237 (190, 286) | 50.5 (38.4, 65.32) | -0.29 (-0.62, 0.03) | 0.079 |
| Panama | 95 (87, 103) | 40.2 (35.13, 45.75) | 149 (113, 184) | 21.26 (15.88, 26.89) | -2.19 (-3.31, -1.05) | <0.001 |
| Papua New Guinea | 158 (95, 273) | 46.33 (25.98, 81.3) | 465 (300, 726) | 50.07 (31.22, 81.48) | 0.28 (0.22, 0.35) | <0.001 |
| Paraguay | 110 (87, 134) | 30.47 (23.7, 38.51) | 465 (338, 629) | 46.99 (32.99, 65.67) | 1.47 (1.24, 1.69) | <0.001 |
| Peru | 794 (645, 959) | 40.34 (31.01, 51.1) | 1529 (1093, 1979) | 28.39 (19.47, 39.35) | -0.84 (-2.02, 0.35) | 0.164 |
| Philippines | 2613 (2305, 2987) | 50.97 (44.7, 58.92) | 7566 (6136, 9129) | 50.49 (40.65, 61.33) | -0.03 (-0.18, 0.12) | 0.691 |
| Poland | 12653 (12360, 12972) | 169.81 (164.72, 174.94) | 14907 (13552, 16214) | 133.64 (121.36, 145.49) | -0.91 (-1.13, -0.69) | <0.001 |
| Portugal | 1435 (1319, 1565) | 63.49 (57.2, 70.36) | 2173 (1951, 2394) | 71.28 (63.39, 79.62) | 0.42 (-0.03, 0.87) | 0.065 |
| Puerto Rico | 281 (261, 306) | 50.25 (44.63, 56.36) | 282 (228, 339) | 30.79 (24.16, 38.32) | -1.72 (-2.23, -1.2) | <0.001 |
| Qatar | 13 (10, 17) | 66.35 (46.56, 91.36) | 59 (41, 83) | 29.71 (19.47, 43.58) | -2.61 (-3.45, -1.77) | <0.001 |
| Republic of Korea | 4741 (4088, 5479) | 87.77 (70.58, 106.13) | 10374 (8735, 12160) | 64.65 (50.36, 82.27) | -1.02 (-1.47, -0.57) | <0.001 |
| Republic of Moldova | 942 (861, 1025) | 115.95 (103.41, 128.45) | 757 (665, 854) | 75.85 (65.14, 87.95) | -1.3 (-2.15, -0.45) | 0.003 |
| Romania | 5235 (4833, 5646) | 102.83 (91.45, 114.57) | 6686 (5777, 7627) | 127.21 (106.86, 150.1) | 0.72 (0.26, 1.19) | 0.002 |
| Russian Federation | 47752 (46921, 48529) | 147.32 (144.14, 150.3) | 36122 (32364, 39506) | 89.51 (80.01, 98.05) | -1.34 (-2.15, -0.54) | 0.001 |
| Rwanda | 157 (113, 198) | 30.23 (20.57, 41.29) | 268 (176, 377) | 22.9 (14.34, 34.48) | -0.89 (-1.04, -0.74) | <0.001 |
| Saint Kitts and Nevis | 2 (2, 2) | 32.91 (28.67, 37.49) | 4 (3, 5) | 29.73 (23.2, 37.02) | -0.34 (-1.01, 0.32) | 0.309 |
| Saint Lucia | 6 (5, 6) | 41.2 (35.72, 47.18) | 13 (10, 16) | 31.74 (24.32, 40.18) | -0.81 (-1.39, -0.23) | 0.007 |
| Saint Vincent and the Grenadines | 3 (3, 4) | 30.67 (26.53, 35.22) | 7 (6, 8) | 27.16 (22.71, 32.36) | -0.41 (-1.04, 0.22) | 0.201 |
| Samoa | 4 (3, 6) | 27.79 (20.24, 37.87) | 7 (5, 9) | 27.27 (18.29, 38.18) | -0.06 (-0.16, 0.04) | 0.216 |
| San Marino | 6 (5, 7) | 120.11 (91.81, 151.83) | 6 (3, 9) | 63.07 (35.89, 97.74) | -2.28 (-2.56, -2) | <0.001 |
| Sao Tome and Principe | 4 (3, 5) | 37.02 (27.19, 49.37) | 9 (7, 12) | 46.64 (31.96, 66.32) | 0.74 (0.43, 1.06) | <0.001 |
| Saudi Arabia | 188 (133, 253) | 19.66 (13.25, 28.06) | 679 (518, 883) | 18.32 (13.08, 25.21) | -0.22 (-0.31, -0.12) | <0.001 |
| Senegal | 123 (96, 155) | 22.26 (15.92, 30.11) | 311 (224, 421) | 23.13 (15.74, 32.73) | 0.11 (-0.09, 0.32) | 0.277 |
| Serbia | 3337 (2656, 4150) | 148.57 (117.04, 186.71) | 4223 (3299, 5248) | 171.2 (132.88, 214.51) | 0.57 (-0.13, 1.28) | 0.113 |
| Seychelles | 5 (4, 7) | 60.69 (45.55, 78.4) | 8 (7, 10) | 38.09 (28.94, 49.49) | -1.39 (-2.1, -0.69) | <0.001 |
| Sierra Leone | 62 (44, 86) | 18.85 (12.64, 27.14) | 118 (79, 169) | 18.67 (11.95, 27.75) | 0.01 (-0.13, 0.14) | 0.898 |
| Singapore | 388 (359, 422) | 104 (93.43, 116.19) | 714 (634, 799) | 47.92 (40.75, 56.17) | -2.42 (-3.24, -1.61) | <0.001 |
| Slovakia | 1762 (1531, 2017) | 175.93 (143.02, 212.31) | 1609 (1250, 1972) | 106.84 (79.92, 136.36) | -1.66 (-2.13, -1.18) | <0.001 |
| Slovenia | 602 (561, 645) | 144.35 (130.58, 159.2) | 669 (561, 790) | 105.52 (87.14, 125.66) | -1.11 (-1.93, -0.28) | 0.009 |
| Solomon Islands | 16 (10, 25) | 61.96 (36.24, 96.57) | 39 (29, 55) | 63.51 (44.41, 90.9) | 0.08 (-0.13, 0.29) | 0.445 |
| Somalia | 88 (56, 150) | 20.33 (12.18, 36.58) | 193 (120, 363) | 17.5 (9.94, 34.05) | -0.47 (-0.53, -0.41) | <0.001 |
| South Africa | 2045 (1746, 2601) | 58.89 (49.26, 75.17) | 5394 (4828, 6075) | 65.75 (57.56, 75.33) | 0.22 (-0.61, 1.06) | 0.605 |
| South Sudan | 114 (73, 173) | 26.66 (15.98, 41.89) | 178 (121, 251) | 24.98 (16.28, 37.34) | -0.2 (-0.28, -0.11) | <0.001 |
| Spain | 9643 (9086, 10203) | 112.61 (101.87, 124.35) | 13486 (12359, 14710) | 107.38 (93.52, 123.4) | -0.23 (-0.49, 0.02) | 0.075 |
| Sri Lanka | 496 (419, 592) | 26.63 (20.31, 34.66) | 1222 (739, 1735) | 25.82 (15.33, 38.29) | 0.02 (-0.41, 0.45) | 0.933 |
| Sudan | 349 (234, 567) | 23.21 (14.39, 39.91) | 805 (529, 1198) | 24.1 (14.9, 36.99) | 0.12 (0.06, 0.17) | <0.001 |
| Suriname | 20 (17, 23) | 44.16 (35.31, 54.6) | 49 (36, 63) | 43.24 (30.88, 58.86) | 0 (-0.8, 0.8) | 0.995 |
| Sweden | 1298 (1217, 1389) | 68.49 (62.81, 74.75) | 1153 (1004, 1312) | 44.35 (37.89, 51.41) | -1.51 (-1.9, -1.12) | <0.001 |
| Switzerland | 1712 (1588, 1837) | 118.99 (105.69, 132.43) | 1962 (1796, 2145) | 81.86 (71.17, 93.03) | -1.34 (-1.66, -1.03) | <0.001 |
| Syrian Arab Republic | 352 (259, 465) | 37.28 (25.45, 51.81) | 900 (613, 1274) | 35.03 (22.55, 51.42) | -0.31 (-0.61, -0.02) | 0.037 |
| Taiwan (Province of China) | 2342 (2218, 2456) | 79.53 (72.16, 87.07) | 5380 (4854, 5817) | 75.43 (65.72, 85.94) | -0.21 (-0.42, 0.01) | 0.058 |
| Tajikistan | 310 (252, 375) | 62.3 (49.52, 76.58) | 305 (203, 444) | 26.07 (17.51, 37.63) | -2.89 (-3.31, -2.47) | <0.001 |
| Thailand | 6176 (4992, 7676) | 97.15 (73.98, 125.16) | 12019 (8948, 15643) | 63.82 (45.43, 87.69) | -1.4 (-1.67, -1.14) | <0.001 |
| Timor-Leste | 21 (14, 29) | 40.3 (26.03, 60.77) | 62 (46, 81) | 42.91 (29.32, 60.23) | 0.2 (0.02, 0.38) | 0.03 |
| Togo | 44 (34, 58) | 20.89 (14.44, 28.94) | 169 (109, 240) | 24.22 (14.53, 36.88) | 0.52 (0.22, 0.82) | 0.001 |
| Tokelau | 0 (0, 0) | 61.15 (42.32, 84.18) | 0 (0, 0) | 63.74 (44.03, 88.56) | 0.12 (-0.06, 0.3) | 0.208 |
| Tonga | 7 (6, 9) | 75.48 (54.39, 101.48) | 10 (8, 13) | 78.33 (54.46, 108.63) | 0.15 (-0.2, 0.49) | 0.401 |
| Trinidad and Tobago | 48 (44, 52) | 36.09 (31.6, 41.39) | 112 (83, 147) | 33.84 (24.41, 45.44) | -0.16 (-0.29, -0.03) | 0.016 |
| Tunisia | 520 (412, 639) | 58.16 (42.36, 76.41) | 1328 (853, 1905) | 56.4 (34.95, 84.95) | -0.09 (-0.22, 0.03) | 0.141 |
| Turkey | 10553 (8148, 13370) | 165.68 (118.37, 223.37) | 17581 (13494, 22149) | 108.36 (80.21, 142.66) | -1.39 (-1.57, -1.21) | <0.001 |
| Turkmenistan | 225 (203, 247) | 64.24 (56.02, 72.71) | 243 (180, 329) | 30.79 (22.76, 41.36) | -2.32 (-3.31, -1.31) | <0.001 |
| Tuvalu | 1 (1, 1) | 67.47 (45.72, 104.32) | 1 (1, 2) | 69.57 (48.69, 99.88) | 0.09 (0.05, 0.13) | <0.001 |
| Uganda | 208 (158, 269) | 18.89 (13.39, 25.89) | 514 (362, 704) | 19.94 (13.14, 29.06) | 0.17 (-0.04, 0.37) | 0.114 |
| Ukraine | 20561 (19141, 22104) | 164.66 (148.29, 182.61) | 9792 (6636, 13582) | 79.68 (53.15, 112.76) | -2.36 (-2.78, -1.94) | <0.001 |
| United Arab Emirates | 43 (29, 60) | 62.05 (39.71, 93.08) | 225 (161, 347) | 22.02 (14.79, 34.08) | -3.32 (-3.71, -2.92) | <0.001 |
| United Kingdom | 20568 (20208, 20893) | 160.75 (157.39, 164.18) | 14346 (13872, 14766) | 83.77 (80.52, 86.92) | -2.12 (-2.3, -1.93) | <0.001 |
| United Republic of Tanzania | 412 (320, 561) | 21.51 (15.04, 30.59) | 944 (662, 1285) | 21.36 (13.62, 31.87) | -0.04 (-0.19, 0.11) | 0.579 |
| United States of America | 94115 (91479, 96383) | 206.09 (199.26, 212.17) | 89286 (85270, 92412) | 99.6 (94.84, 103.48) | -2.32 (-2.53, -2.12) | <0.001 |
| United States Virgin Islands | 8 (7, 10) | 55.25 (41.16, 72.42) | 10 (7, 14) | 36.66 (25.67, 51.85) | -1.39 (-2.56, -0.2) | 0.022 |
| Uruguay | 872 (807, 936) | 143.44 (129.11, 159) | 870 (806, 936) | 113.79 (101.94, 126.5) | -0.82 (-0.97, -0.68) | <0.001 |
| Uzbekistan | 1310 (1187, 1432) | 64.85 (58.22, 71.73) | 1280 (1022, 1569) | 24.38 (19.48, 29.89) | -3.24 (-4, -2.48) | <0.001 |
| Vanuatu | 6 (4, 10) | 50.78 (29.32, 90.18) | 16 (11, 26) | 50.36 (31.42, 82.68) | -0.01 (-0.32, 0.29) | 0.92 |
| Venezuela (Bolivarian Republic of) | 951 (893, 1015) | 59.67 (52.96, 67.37) | 2762 (2057, 3622) | 52.63 (38.06, 70.65) | -0.53 (-0.98, -0.08) | 0.021 |
| Viet Nam | 4906 (3554, 6362) | 70.14 (49.41, 93.82) | 14253 (9952, 18856) | 76.24 (52.56, 103) | 0.28 (0.14, 0.42) | <0.001 |
| Yemen | 268 (156, 470) | 30.27 (17.06, 53.62) | 684 (424, 1087) | 28.12 (16.4, 46.31) | -0.23 (-0.38, -0.07) | 0.004 |
| Zambia | 126 (100, 155) | 25.19 (18.43, 33.51) | 404 (276, 731) | 33.43 (21.56, 60.57) | 0.92 (0.8, 1.04) | <0.001 |
| Zimbabwe | 263 (210, 326) | 36.68 (26.84, 48.18) | 510 (390, 684) | 40.59 (28.68, 56.75) | 0.36 (0.14, 0.56) | 0.001 |
| LOLC |  |  |  |  |  |  |
| Afghanistan | 167 (96, 324) | 53.3 (28.89, 103.72) | 262 (174, 422) | 64.87 (40.52, 106.31) | 0.62 (0.55, 0.7) | <0.001 |
| Albania | 186 (147, 234) | 184.57 (142.73, 236.21) | 501 (345, 692) | 183.47 (125.13, 254.63) | -0.06 (-0.43, 0.31) | 0.761 |
| Algeria | 334 (246, 441) | 62.02 (44.06, 85.61) | 798 (566, 1107) | 49.59 (34.46, 70.49) | -0.76 (-1.03, -0.49) | <0.001 |
| American Samoa | 2 (2, 2) | 217.1 (163.98, 278.13) | 4 (4, 6) | 201.73 (152.27, 263.76) | -0.15 (-0.28, -0.02) | 0.025 |
| Andorra | 11 (8, 15) | 332.07 (226.12, 474.06) | 20 (14, 27) | 217.85 (143, 312.2) | -1.46 (-1.81, -1.1) | <0.001 |
| Angola | 95 (65, 133) | 69.98 (44.01, 104.11) | 307 (230, 389) | 75.18 (52.47, 103.72) | 0.25 (0.09, 0.41) | 0.002 |
| Antigua and Barbuda | 3 (2, 3) | 73.24 (63.68, 83.7) | 4 (4, 5) | 76.88 (66.8, 89.08) | 0.02 (-0.93, 0.98) | 0.965 |
| Argentina | 3107 (2836, 3396) | 166.82 (148.31, 186.67) | 5129 (4589, 5660) | 144.19 (125.13, 163.14) | -0.41 (-0.57, -0.24) | <0.001 |
| Armenia | 165 (151, 179) | 141.89 (129.44, 154.87) | 451 (401, 504) | 189.32 (166.62, 213.28) | 0.91 (0.18, 1.65) | 0.014 |
| Australia | 3289 (3044, 3497) | 269.3 (239.64, 299.54) | 8331 (7100, 9404) | 267.43 (221.45, 315.46) | -0.03 (-0.35, 0.3) | 0.874 |
| Austria | 1512 (1402, 1607) | 194.42 (174.04, 214.45) | 2531 (2217, 2813) | 210.46 (181.18, 240.74) | 0.3 (-0.08, 0.68) | 0.12 |
| Azerbaijan | 191 (167, 217) | 87.77 (73.88, 104.27) | 318 (228, 424) | 82.54 (56.32, 115.56) | -0.17 (-0.55, 0.2) | 0.366 |
| Bahamas | 7 (7, 8) | 90.61 (78.65, 103.28) | 19 (15, 23) | 96.14 (77.07, 118.38) | 0.22 (-0.63, 1.08) | 0.609 |
| Bahrain | 18 (15, 21) | 313.94 (241.48, 401.16) | 45 (36, 57) | 196.6 (143.03, 262.57) | -1.56 (-2.12, -1) | <0.001 |
| Bangladesh | 868 (604, 1272) | 37.75 (24.84, 58.32) | 2486 (1829, 3362) | 34.17 (23.73, 50.3) | -0.17 (-0.68, 0.33) | 0.497 |
| Barbados | 16 (15, 17) | 75.5 (66.57, 85.11) | 24 (20, 29) | 75.63 (59.78, 93.37) | 0.13 (-0.8, 1.07) | 0.784 |
| Belarus | 931 (865, 1006) | 138.22 (122.38, 154.82) | 1173 (952, 1393) | 126.07 (99.79, 154.33) | -0.31 (-0.94, 0.34) | 0.348 |
| Belgium | 3433 (3105, 3754) | 357.74 (317.57, 401) | 4302 (3689, 4872) | 270.53 (228.07, 311.02) | -0.96 (-1.3, -0.62) | <0.001 |
| Belize | 4 (3, 4) | 72.27 (62.36, 82.65) | 11 (10, 13) | 79.48 (66.6, 93.57) | 0.26 (-0.48, 0.99) | 0.496 |
| Benin | 46 (37, 59) | 44.68 (31.63, 61.7) | 105 (81, 137) | 46.49 (32.26, 64.52) | 0.13 (0.01, 0.25) | 0.029 |
| Bermuda | 11 (10, 12) | 316.08 (269.71, 369.25) | 22 (18, 27) | 236.9 (184.37, 302.03) | -0.96 (-1.32, -0.6) | <0.001 |
| Bhutan | 2 (1, 4) | 25.33 (14.18, 40.03) | 12 (8, 17) | 37.22 (23.48, 55.42) | 1.25 (1.13, 1.37) | <0.001 |
| Bolivia (Plurinational State of) | 170 (123, 224) | 112.02 (76.19, 155.37) | 520 (364, 742) | 113.45 (74.83, 166.02) | 0.05 (-0.02, 0.12) | 0.178 |
| Bosnia and Herzegovina | 311 (263, 363) | 183.43 (151.97, 221.19) | 890 (667, 1166) | 230.41 (171.21, 303.96) | 0.79 (0.6, 0.99) | <0.001 |
| Botswana | 23 (17, 30) | 98.2 (67.38, 136.4) | 61 (44, 79) | 103.64 (71.13, 143.39) | 0.18 (-0.05, 0.42) | 0.121 |
| Brazil | 4417 (4132, 4628) | 103.49 (95.4, 109.91) | 15338 (13709, 16428) | 109.14 (96.89, 118.18) | 0.2 (0.01, 0.39) | 0.044 |
| Brunei Darussalam | 15 (11, 19) | 324.52 (232.31, 446.8) | 32 (25, 38) | 241.26 (178.31, 316.17) | -1.01 (-1.71, -0.31) | 0.005 |
| Bulgaria | 795 (713, 889) | 123.15 (108.23, 139.39) | 1799 (1501, 2119) | 174.81 (144.18, 208.06) | 1.2 (0.89, 1.51) | <0.001 |
| Burkina Faso | 72 (53, 107) | 35.89 (24.32, 55.96) | 183 (134, 269) | 43.24 (29.57, 65.92) | 0.64 (0.46, 0.83) | <0.001 |
| Burundi | 50 (38, 67) | 42.05 (29.96, 59.6) | 65 (46, 92) | 35.97 (23.37, 54.5) | -0.51 (-0.66, -0.36) | <0.001 |
| Cabo Verde | 13 (11, 15) | 84.6 (63.46, 109.22) | 31 (24, 38) | 149.32 (104.45, 199.51) | 1.88 (1.16, 2.62) | <0.001 |
| Cambodia | 281 (199, 376) | 146.14 (95.9, 212.89) | 963 (689, 1267) | 174.86 (116.35, 243.25) | 0.61 (0.52, 0.7) | <0.001 |
| Cameroon | 95 (77, 115) | 51.33 (37.3, 68.17) | 299 (223, 394) | 59.25 (40.72, 84.48) | 0.49 (0.36, 0.62) | <0.001 |
| Canada | 7258 (6738, 7733) | 359.42 (321.8, 397.95) | 16938 (14961, 18792) | 346.86 (292.81, 402.72) | -0.03 (-0.19, 0.13) | 0.695 |
| Central African Republic | 24 (16, 42) | 59.61 (37.07, 106.56) | 35 (23, 62) | 52.12 (32.16, 92.7) | -0.44 (-0.54, -0.35) | <0.001 |
| Chad | 50 (35, 73) | 32.56 (21.43, 49.59) | 124 (86, 183) | 51.27 (33.61, 77.88) | 1.48 (1.32, 1.63) | <0.001 |
| Chile | 649 (597, 701) | 119.19 (106.34, 132.21) | 1986 (1735, 2211) | 127.07 (108.38, 144.47) | 0.14 (-0.1, 0.38) | 0.265 |
| China | 85126 (74364, 95664) | 231.98 (199.58, 264.77) | 450925 (366551, 538018) | 382.36 (308.87, 459.79) | 1.61 (1.33, 1.9) | <0.001 |
| Colombia | 948 (877, 1011) | 112.98 (100.18, 126.58) | 2993 (2488, 3534) | 94.55 (75.27, 115.18) | -0.73 (-1.15, -0.31) | 0.001 |
| Comoros | 4 (2, 5) | 42.68 (28.68, 60.31) | 11 (7, 16) | 49.09 (30.21, 72.44) | 0.45 (0.31, 0.58) | <0.001 |
| Congo | 39 (26, 52) | 85.83 (55.81, 120.31) | 82 (60, 108) | 84.82 (58.44, 119.23) | -0.03 (-0.1, 0.03) | 0.321 |
| Cook Islands | 2 (1, 2) | 271.03 (199.35, 362.94) | 4 (3, 4) | 227.94 (165.69, 301.93) | -0.55 (-0.67, -0.44) | <0.001 |
| Costa Rica | 87 (80, 95) | 91.74 (80.32, 103.9) | 202 (173, 230) | 64.16 (52.17, 76.39) | -1.11 (-2.63, 0.43) | 0.158 |
| Côte d'Ivoire | 38 (31, 47) | 27.2 (20.16, 36.4) | 117 (87, 158) | 27.39 (18.96, 38.65) | 0.2 (-0.16, 0.57) | 0.281 |
| Croatia | 756 (668, 845) | 263.63 (232.04, 296.95) | 1718 (1502, 1936) | 280.52 (235.93, 330.72) | 0.06 (-0.43, 0.54) | 0.824 |
| Cuba | 1665 (1556, 1781) | 264.69 (233.79, 297.41) | 3408 (2945, 3926) | 272.69 (227.2, 321.97) | -0.03 (-0.29, 0.23) | 0.829 |
| Cyprus | 85 (66, 105) | 190.04 (136.18, 258.78) | 253 (202, 315) | 187.93 (139.34, 245.75) | 0.32 (-0.14, 0.79) | 0.173 |
| Czechia | 1915 (1778, 2068) | 240.41 (217.16, 266.46) | 3933 (3431, 4472) | 254.79 (215.38, 296.61) | 0.03 (-0.09, 0.14) | 0.648 |
| Democratic People's Republic of Korea | 990 (733, 1248) | 149.69 (103.56, 204.3) | 2507 (1623, 3624) | 142.32 (86.91, 214.25) | -0.17 (-0.23, -0.1) | <0.001 |
| Democratic Republic of the Congo | 320 (208, 543) | 54.65 (33.13, 97.27) | 609 (381, 1061) | 47.29 (27.86, 86.03) | -0.45 (-0.56, -0.34) | <0.001 |
| Denmark | 1860 (1709, 2001) | 337.08 (302.91, 371.43) | 3039 (2577, 3452) | 351.35 (292.62, 409.88) | 0.08 (-0.14, 0.3) | 0.494 |
| Djibouti | 2 (2, 3) | 48.41 (32.29, 68.71) | 15 (10, 21) | 67.87 (41.88, 100.11) | 1.11 (1.03, 1.19) | <0.001 |
| Dominica | 5 (4, 6) | 130.45 (101.02, 165.08) | 6 (5, 8) | 136.76 (101.73, 178.25) | 0.15 (-0.02, 0.32) | 0.085 |
| Dominican Republic | 137 (114, 161) | 76.73 (57.38, 100.27) | 530 (366, 719) | 94.75 (60.25, 138.08) | 0.82 (0.23, 1.42) | 0.006 |
| Ecuador | 182 (167, 196) | 67.42 (59.55, 75.6) | 646 (521, 792) | 69.07 (53.94, 87.14) | 0.19 (-0.98, 1.39) | 0.748 |
| Egypt | 287 (239, 342) | 29.39 (22.39, 37.73) | 2244 (1766, 2802) | 100.93 (75.6, 132.4) | 4.01 (3.54, 4.49) | <0.001 |
| El Salvador | 92 (80, 107) | 58.24 (46.8, 71.1) | 258 (207, 326) | 67.1 (50.4, 88.4) | 0.45 (-0.43, 1.33) | 0.318 |
| Equatorial Guinea | 5 (3, 8) | 61.53 (39.15, 101.76) | 18 (12, 26) | 95.71 (59.61, 143.2) | 1.49 (1.39, 1.6) | <0.001 |
| Eritrea | 10 (8, 14) | 33.64 (23.99, 47.86) | 44 (34, 56) | 47.4 (34.5, 63.14) | 1.13 (1.02, 1.24) | <0.001 |
| Estonia | 216 (203, 231) | 188.29 (169.68, 207.55) | 351 (295, 402) | 187.42 (156.21, 219.23) | -0.22 (-0.68, 0.25) | 0.359 |
| Eswatini | 12 (7, 19) | 94.5 (55.7, 155.15) | 26 (15, 38) | 110.8 (62.43, 168.29) | 0.54 (0.41, 0.68) | <0.001 |
| Ethiopia | 501 (381, 663) | 61.18 (45.58, 83.37) | 945 (754, 1154) | 49.46 (38.02, 62.5) | -0.66 (-0.75, -0.56) | <0.001 |
| Fiji | 10 (8, 12) | 74.31 (54.34, 98.7) | 23 (17, 29) | 71.87 (50.77, 96.59) | -0.16 (-0.5, 0.19) | 0.382 |
| Finland | 1036 (950, 1106) | 225.46 (200.81, 249.42) | 2151 (1861, 2419) | 230.03 (191.8, 267.16) | 0.09 (-0.13, 0.31) | 0.417 |
| France | 9738 (9022, 10432) | 193.6 (172.08, 214.66) | 22691 (19828, 25157) | 231.31 (195.9, 266.31) | 0.6 (0.43, 0.77) | <0.001 |
| Gabon | 27 (18, 39) | 92.97 (57.59, 142.7) | 41 (30, 56) | 101.41 (67.41, 142.78) | 0.31 (0.23, 0.39) | <0.001 |
| Gambia | 3 (2, 4) | 18.17 (12.8, 25.02) | 10 (7, 13) | 20.98 (13.73, 30.62) | 0.45 (0.05, 0.85) | 0.027 |
| Georgia | 352 (317, 385) | 107.15 (94.17, 120.5) | 525 (459, 588) | 148.37 (127.03, 171.15) | 0.9 (-0.08, 1.89) | 0.072 |
| Germany | 17523 (16153, 18730) | 214.88 (192.54, 238.1) | 31702 (27839, 35012) | 240.05 (205.01, 275.83) | 0.39 (0.16, 0.62) | 0.001 |
| Ghana | 100 (79, 125) | 39.91 (27.75, 55.57) | 335 (251, 424) | 49.03 (33.32, 68.08) | 0.68 (0.62, 0.75) | <0.001 |
| Greece | 3059 (2786, 3312) | 319.43 (280.84, 356.63) | 5119 (4523, 5597) | 302.79 (262.93, 340.16) | -0.16 (-0.31, -0.02) | 0.03 |
| Greenland | 9 (7, 10) | 679.82 (537.56, 832.07) | 14 (11, 19) | 476.16 (356.27, 630.71) | -1.01 (-1.43, -0.59) | <0.001 |
| Grenada | 4 (4, 4) | 80.17 (67.86, 92.89) | 5 (4, 6) | 84.57 (70.25, 100.39) | 0.52 (-0.73, 1.79) | 0.417 |
| Guam | 8 (7, 9) | 281.18 (227.49, 340.67) | 18 (14, 21) | 148.1 (113.23, 186.55) | -2.2 (-3.3, -1.08) | <0.001 |
| Guatemala | 97 (89, 104) | 69.17 (61.41, 77.14) | 264 (225, 304) | 44.99 (37.52, 53.35) | -1.55 (-2.25, -0.84) | <0.001 |
| Guinea | 74 (55, 96) | 44.24 (29.7, 62.59) | 146 (102, 199) | 55.86 (35.57, 83.54) | 0.79 (0.66, 0.92) | <0.001 |
| Guinea-Bissau | 9 (7, 13) | 54.19 (36.14, 79.16) | 15 (11, 19) | 57.03 (40.71, 79.31) | 0.17 (0.09, 0.25) | <0.001 |
| Guyana | 9 (8, 10) | 49.37 (41.67, 58.14) | 13 (10, 16) | 43.78 (33.4, 55.66) | -0.42 (-0.84, 0) | 0.047 |
| Haiti | 114 (79, 174) | 83.94 (54.14, 137.14) | 216 (139, 349) | 76.02 (45.62, 128.13) | -0.3 (-0.36, -0.24) | <0.001 |
| Honduras | 83 (64, 105) | 84.75 (61.3, 115.29) | 467 (308, 669) | 148.15 (93.35, 221.61) | 1.88 (1.58, 2.19) | <0.001 |
| Hungary | 2006 (1816, 2191) | 235.52 (209.77, 262.95) | 3553 (3025, 4122) | 267.68 (225.63, 310.56) | 0.51 (0.19, 0.82) | 0.002 |
| Iceland | 47 (43, 52) | 260.59 (227.59, 295.62) | 106 (89, 121) | 277.08 (225.38, 329.67) | 0.04 (-0.58, 0.68) | 0.889 |
| India | 4781 (4031, 5609) | 25.82 (21.53, 30.55) | 21675 (17849, 24931) | 36.11 (29.58, 41.82) | 1.19 (0.72, 1.67) | <0.001 |
| Indonesia | 3845 (2838, 4771) | 99.92 (72.89, 125.18) | 15291 (10151, 19446) | 159.7 (104.87, 205.89) | 1.54 (1.47, 1.6) | <0.001 |
| Iran (Islamic Republic of) | 695 (551, 853) | 74.04 (58.09, 92.85) | 2909 (2560, 3220) | 79.41 (68.38, 89.39) | 0.17 (0.01, 0.32) | 0.033 |
| Iraq | 372 (275, 492) | 93.89 (64.19, 131.63) | 1426 (1062, 1809) | 144.05 (99.3, 195.73) | 1.49 (1.14, 1.84) | <0.001 |
| Ireland | 830 (768, 886) | 303.16 (270.69, 335.48) | 1306 (1105, 1467) | 249.91 (207.53, 292.08) | -0.6 (-0.99, -0.2) | 0.003 |
| Israel | 523 (475, 567) | 171.37 (150.77, 192.3) | 1386 (1184, 1555) | 166.72 (138.97, 192.64) | -0.04 (-0.25, 0.16) | 0.668 |
| Italy | 13092 (12159, 13718) | 235.85 (218.81, 248.97) | 25722 (22156, 28069) | 242.47 (208.55, 269.12) | 0.1 (-0.03, 0.24) | 0.116 |
| Jamaica | 122 (110, 134) | 104.64 (89.75, 120.96) | 218 (168, 279) | 122.83 (91.02, 161.08) | 0.96 (0.48, 1.44) | <0.001 |
| Japan | 28103 (25810, 29493) | 281.85 (257.56, 300.25) | 96775 (81326, 105685) | 312.78 (263.71, 345.89) | 0.29 (0.03, 0.56) | 0.031 |
| Jordan | 37 (29, 48) | 75.83 (55.11, 104) | 229 (178, 294) | 75.14 (53.68, 102.4) | -0.03 (-0.49, 0.44) | 0.916 |
| Kazakhstan | 993 (919, 1071) | 166.57 (151.03, 181.96) | 726 (621, 839) | 86.91 (73.8, 100.74) | -2.05 (-2.77, -1.32) | <0.001 |
| Kenya | 48 (31, 69) | 12.67 (8.16, 18.29) | 206 (161, 260) | 21.51 (16.76, 27.33) | 1.7 (1.48, 1.91) | <0.001 |
| Kiribati | 1 (1, 2) | 91.49 (64.15, 122.65) | 3 (2, 4) | 107.18 (73.76, 152.09) | 0.51 (0.4, 0.63) | <0.001 |
| Kuwait | 20 (17, 22) | 98.61 (81.87, 117.75) | 71 (57, 88) | 71.64 (54.13, 92.35) | -1.03 (-4.2, 2.25) | 0.534 |
| Kyrgyzstan | 154 (137, 170) | 110.75 (97.06, 124.23) | 123 (101, 150) | 64.62 (52.37, 78.98) | -1.84 (-2.54, -1.13) | <0.001 |
| Lao People's Democratic Republic | 132 (88, 211) | 141.03 (89.18, 227.22) | 291 (209, 397) | 150.21 (101.46, 216.33) | 0.21 (0.14, 0.27) | <0.001 |
| Latvia | 339 (307, 374) | 166.6 (149.09, 185.69) | 460 (388, 531) | 169.32 (139.39, 199.58) | 0.22 (-1.07, 1.52) | 0.744 |
| Lebanon | 165 (123, 216) | 171.21 (121.72, 233) | 725 (553, 950) | 185.25 (134.46, 255.26) | 0.31 (-0.01, 0.63) | 0.055 |
| Lesotho | 30 (22, 44) | 66.4 (45.45, 100.92) | 58 (41, 78) | 112.19 (73.36, 161.28) | 1.74 (1.52, 1.95) | <0.001 |
| Liberia | 25 (19, 32) | 41.44 (29.46, 58.59) | 32 (21, 48) | 40.41 (24.17, 64.01) | -0.09 (-0.34, 0.15) | 0.45 |
| Libya | 146 (106, 192) | 168.79 (114.23, 239.29) | 388 (279, 523) | 176.81 (117.62, 253.25) | 0.17 (-0.17, 0.52) | 0.326 |
| Lithuania | 424 (392, 456) | 172.18 (155.46, 189.75) | 687 (590, 787) | 177.72 (147.29, 210.34) | -0.1 (-0.56, 0.36) | 0.658 |
| Luxembourg | 97 (90, 104) | 285.77 (255.24, 318.83) | 162 (141, 182) | 243.79 (207.83, 278.2) | -0.49 (-1.12, 0.14) | 0.129 |
| Madagascar | 79 (64, 96) | 35.77 (26.6, 47.75) | 122 (91, 156) | 33.43 (23.14, 45.7) | -0.2 (-0.32, -0.08) | 0.001 |
| Malawi | 22 (17, 27) | 13.71 (9.8, 18.6) | 50 (38, 66) | 15.29 (10.66, 21.65) | 0.4 (0.29, 0.52) | <0.001 |
| Malaysia | 480 (385, 611) | 110.78 (80.22, 152.34) | 1814 (1478, 2177) | 129.03 (95.42, 169.16) | 0.25 (-0.09, 0.59) | 0.144 |
| Maldives | 3 (2, 4) | 110.89 (73.23, 165.34) | 10 (8, 13) | 73.87 (53.61, 97.96) | -1.44 (-1.69, -1.18) | <0.001 |
| Mali | 48 (39, 60) | 29.06 (21.6, 38.18) | 125 (93, 163) | 33.48 (23.25, 46.65) | 0.48 (0.34, 0.61) | <0.001 |
| Malta | 50 (45, 55) | 196.02 (171.18, 223.02) | 111 (94, 127) | 155.46 (129.41, 181.52) | -0.86 (-1.15, -0.56) | <0.001 |
| Marshall Islands | 1 (1, 2) | 156.3 (105.33, 235.39) | 2 (2, 3) | 203.58 (131.77, 298.37) | 0.81 (0.65, 0.98) | <0.001 |
| Mauritania | 25 (19, 32) | 47.86 (34.31, 63.93) | 60 (43, 81) | 58.7 (39.99, 83.43) | 0.65 (0.52, 0.77) | <0.001 |
| Mauritius | 32 (29, 35) | 96.67 (85.83, 108.52) | 74 (66, 80) | 74.26 (64.54, 84.09) | -1.24 (-2.13, -0.34) | 0.007 |
| Mexico | 2349 (2244, 2420) | 115.36 (109.03, 120.64) | 4231 (3767, 4689) | 62.57 (55.26, 69.65) | -2.04 (-2.28, -1.8) | <0.001 |
| Micronesia (Federated States of) | 4 (3, 6) | 176.33 (124.07, 251.2) | 5 (4, 7) | 201.15 (134.61, 291.01) | 0.43 (0.38, 0.48) | <0.001 |
| Monaco | 18 (14, 23) | 369.27 (269.86, 485.83) | 41 (32, 50) | 571.27 (434.64, 731.28) | 1.41 (1.22, 1.61) | <0.001 |
| Mongolia | 98 (75, 127) | 184.56 (138.8, 242.06) | 128 (96, 170) | 149.66 (110.61, 197.8) | -0.72 (-1.17, -0.28) | 0.001 |
| Montenegro | 77 (61, 96) | 237.43 (185.04, 301.36) | 193 (147, 251) | 332.7 (247.32, 434.74) | 1.27 (0.91, 1.63) | <0.001 |
| Morocco | 586 (460, 740) | 81.45 (57.25, 113.62) | 1567 (1147, 1940) | 93.88 (62.73, 132.12) | 0.43 (0.32, 0.54) | <0.001 |
| Mozambique | 97 (79, 117) | 40.34 (29.51, 54.43) | 217 (156, 288) | 51.02 (34.18, 72.48) | 0.77 (0.61, 0.93) | <0.001 |
| Myanmar | 1344 (977, 1908) | 133.56 (90.17, 199.95) | 2913 (2273, 3741) | 130.21 (92.9, 178.5) | -0.07 (-0.13, -0.01) | 0.021 |
| Namibia | 8 (6, 10) | 29.39 (21.07, 40) | 21 (17, 27) | 34.59 (24.91, 47.19) | 0.55 (0.43, 0.68) | <0.001 |
| Nauru | 0 (0, 1) | 279.8 (152.09, 410.64) | 1 (0, 1) | 260.74 (142.18, 354.77) | -0.23 (-0.3, -0.16) | <0.001 |
| Nepal | 118 (75, 177) | 31.62 (19.21, 50.02) | 442 (315, 616) | 37.99 (25.78, 55.47) | 0.61 (0.5, 0.71) | <0.001 |
| Netherlands | 4337 (4028, 4619) | 339.6 (306.74, 373.85) | 7417 (6506, 8178) | 303.19 (257.81, 345.89) | -0.38 (-0.47, -0.29) | <0.001 |
| New Zealand | 710 (646, 771) | 284.33 (249.11, 319.02) | 1498 (1298, 1667) | 266.38 (222.05, 309.04) | -0.28 (-0.55, 0) | 0.047 |
| Nicaragua | 30 (26, 35) | 41.34 (32.13, 52.78) | 95 (76, 118) | 38.26 (27.93, 50.61) | -0.28 (-0.75, 0.19) | 0.239 |
| Niger | 31 (21, 47) | 29.25 (18.79, 46.65) | 113 (79, 188) | 33.5 (20.96, 57.88) | 0.45 (0.33, 0.57) | <0.001 |
| Nigeria | 184 (134, 235) | 8.65 (6.25, 11.12) | 421 (352, 512) | 11.23 (9.09, 13.83) | 0.83 (0.75, 0.92) | <0.001 |
| Niue | 0 (0, 0) | 165.63 (117.32, 226.63) | 0 (0, 0) | 221.4 (149.35, 304.92) | 0.95 (0.88, 1.02) | <0.001 |
| North Macedonia | 135 (114, 161) | 152.29 (122.85, 187.22) | 359 (282, 445) | 188.68 (144.58, 236.87) | 0.75 (0.41, 1.1) | <0.001 |
| Northern Mariana Islands | 2 (1, 2) | 358.74 (273.65, 466.37) | 6 (5, 7) | 301.53 (227.39, 386.08) | -0.54 (-0.75, -0.34) | <0.001 |
| Norway | 902 (840, 943) | 185.22 (170.92, 198.09) | 2085 (1813, 2273) | 302.32 (259.02, 339.18) | 1.71 (1.58, 1.85) | <0.001 |
| Oman | 11 (7, 16) | 37.32 (24.01, 54.5) | 24 (18, 32) | 34.54 (23.77, 49) | -0.22 (-1.05, 0.63) | 0.618 |
| Pakistan | 2177 (1696, 2614) | 76.17 (57.32, 97.71) | 4367 (3285, 5636) | 85.61 (61.77, 115.02) | 0.38 (0.3, 0.47) | <0.001 |
| Palau | 2 (1, 2) | 367.28 (272.47, 489.61) | 3 (3, 4) | 379.31 (278.82, 496.38) | 0.05 (-0.06, 0.17) | 0.36 |
| Palestine | 64 (48, 82) | 151.15 (101.7, 215.03) | 140 (113, 169) | 135.75 (100.6, 179.84) | -0.43 (-0.8, -0.06) | 0.023 |
| Panama | 76 (69, 84) | 94.72 (81.32, 108.6) | 184 (142, 221) | 69.96 (53.32, 86.85) | -0.92 (-1.5, -0.35) | 0.002 |
| Papua New Guinea | 70 (43, 118) | 113.49 (66.18, 197.62) | 240 (153, 388) | 136.36 (80.9, 225.7) | 0.63 (0.57, 0.68) | <0.001 |
| Paraguay | 76 (63, 92) | 65.45 (51.04, 82.99) | 361 (272, 479) | 119.81 (83.57, 166.85) | 2.2 (1.88, 2.52) | <0.001 |
| Peru | 645 (536, 778) | 107.31 (83.74, 135.15) | 1732 (1250, 2218) | 91.05 (63.82, 122.61) | -0.44 (-1.92, 1.07) | 0.57 |
| Philippines | 1466 (1314, 1666) | 118.28 (104.54, 135.9) | 3998 (3376, 4695) | 109.62 (91.76, 129.14) | -0.22 (-0.47, 0.04) | 0.093 |
| Poland | 4979 (4769, 5146) | 206.01 (196.38, 213.73) | 13317 (11954, 14468) | 287.61 (256.62, 313.83) | 1.09 (0.94, 1.25) | <0.001 |
| Portugal | 1078 (981, 1179) | 125 (109.46, 142.07) | 2346 (2022, 2645) | 132.47 (111.35, 153.28) | 0.2 (0.01, 0.38) | 0.042 |
| Puerto Rico | 299 (275, 320) | 134.45 (118.55, 151.12) | 476 (388, 560) | 90 (71.21, 110.8) | -1.49 (-1.84, -1.14) | <0.001 |
| Qatar | 5 (4, 7) | 213.88 (152.48, 292.72) | 21 (16, 28) | 119.61 (82.47, 176.04) | -1.82 (-2.97, -0.66) | 0.002 |
| Republic of Korea | 2344 (2047, 2667) | 181.88 (149.92, 216.88) | 19702 (15964, 23592) | 347.05 (266.29, 436.52) | 2.06 (1.85, 2.28) | <0.001 |
| Republic of Moldova | 283 (262, 303) | 132.97 (118.87, 146.9) | 315 (278, 356) | 93.38 (80.92, 107.61) | -1.25 (-2.24, -0.25) | 0.014 |
| Romania | 1137 (1036, 1242) | 80.75 (71.53, 90.36) | 4106 (3563, 4686) | 165.67 (140.64, 194.01) | 2.36 (2.06, 2.67) | <0.001 |
| Russian Federation | 14500 (13979, 14885) | 148.56 (142.87, 153.12) | 21201 (19414, 22973) | 149.93 (135.58, 162.91) | -0.05 (-0.78, 0.68) | 0.895 |
| Rwanda | 57 (43, 71) | 49.2 (34.48, 66.37) | 124 (84, 170) | 50.2 (32.58, 73.29) | 0.07 (-0.1, 0.24) | 0.421 |
| Saint Kitts and Nevis | 2 (2, 2) | 78.97 (69.17, 89.85) | 2 (2, 3) | 79.47 (65.44, 95.26) | 0.08 (-0.46, 0.62) | 0.782 |
| Saint Lucia | 5 (5, 6) | 101.84 (88.73, 115.76) | 11 (9, 13) | 82.78 (65.91, 101.36) | -0.79 (-1.33, -0.25) | 0.004 |
| Saint Vincent and the Grenadines | 3 (3, 3) | 72.13 (63.51, 81.67) | 5 (5, 6) | 65.98 (55.7, 77.81) | -0.32 (-0.81, 0.18) | 0.214 |
| Samoa | 2 (2, 3) | 57.72 (42.36, 76.87) | 4 (3, 5) | 56.37 (38.75, 76.93) | -0.09 (-0.18, 0.01) | 0.073 |
| San Marino | 7 (6, 8) | 301.64 (230.21, 388.85) | 8 (6, 11) | 162.6 (103.46, 234.06) | -2.31 (-2.57, -2.05) | <0.001 |
| Sao Tome and Principe | 3 (3, 4) | 94.94 (70.14, 125.69) | 6 (5, 8) | 139.88 (96.47, 197.63) | 1.26 (1.08, 1.45) | <0.001 |
| Saudi Arabia | 105 (80, 135) | 42.3 (29.82, 58.39) | 247 (197, 305) | 47.73 (34.22, 65.32) | 0.38 (0.23, 0.53) | <0.001 |
| Senegal | 75 (58, 95) | 48.8 (34.07, 68.03) | 219 (163, 282) | 61.64 (41.57, 87.2) | 0.8 (0.63, 0.98) | <0.001 |
| Serbia | 999 (803, 1240) | 214.1 (163.07, 276.46) | 2541 (2008, 3138) | 238.08 (180.01, 302.91) | 0.38 (0.03, 0.74) | 0.036 |
| Seychelles | 4 (4, 5) | 126.53 (96.9, 162.06) | 6 (5, 7) | 112.07 (85.94, 145.68) | -0.69 (-0.92, -0.47) | <0.001 |
| Sierra Leone | 48 (37, 63) | 43.63 (30.88, 61.16) | 77 (54, 106) | 44.86 (29.25, 64.6) | 0.12 (0.04, 0.2) | 0.004 |
| Singapore | 314 (289, 341) | 296.91 (264.52, 331.87) | 1068 (920, 1200) | 222.48 (183.5, 261.01) | -0.82 (-1.95, 0.32) | 0.158 |
| Slovakia | 751 (620, 880) | 226.16 (177.56, 279.71) | 1182 (928, 1464) | 196.47 (143.43, 260.81) | -0.41 (-0.83, 0.02) | 0.06 |
| Slovenia | 239 (219, 259) | 177.86 (158.78, 197.74) | 690 (584, 779) | 233.2 (192.32, 275.61) | 0.96 (0.7, 1.21) | <0.001 |
| Solomon Islands | 6 (4, 8) | 119.02 (81.52, 167.19) | 15 (12, 20) | 115.28 (84.91, 155.8) | -0.08 (-0.18, 0.03) | 0.154 |
| Somalia | 23 (15, 38) | 31.04 (18.95, 54.05) | 57 (36, 102) | 27.81 (16.48, 51.89) | -0.34 (-0.39, -0.29) | <0.001 |
| South Africa | 987 (843, 1199) | 97.51 (80.69, 119.56) | 2665 (2392, 2958) | 120.72 (105.23, 136.41) | 0.69 (0.13, 1.26) | 0.016 |
| South Sudan | 61 (39, 90) | 45.72 (28.39, 70.34) | 68 (48, 92) | 48.56 (32.45, 69.65) | 0.19 (0.11, 0.26) | <0.001 |
| Spain | 7244 (6693, 7723) | 209.52 (186.38, 233.67) | 14105 (12077, 15672) | 211.6 (175.21, 250.65) | 0.06 (-0.12, 0.25) | 0.503 |
| Sri Lanka | 294 (249, 355) | 60.39 (45.44, 79.83) | 768 (508, 1044) | 50.35 (32.62, 72.92) | -0.63 (-1.14, -0.12) | 0.015 |
| Sudan | 226 (156, 370) | 48.06 (30.21, 82.36) | 533 (367, 780) | 62.59 (40.14, 94.51) | 0.87 (0.79, 0.94) | <0.001 |
| Suriname | 11 (9, 13) | 91.41 (71.74, 114.68) | 28 (21, 38) | 85.38 (59.42, 119.14) | 0.03 (-0.62, 0.69) | 0.92 |
| Sweden | 1490 (1360, 1599) | 136.81 (121.42, 151.34) | 2791 (2357, 3177) | 173.13 (143.76, 202.69) | 0.77 (0.4, 1.14) | <0.001 |
| Switzerland | 1502 (1362, 1630) | 217.4 (188.41, 246.81) | 2892 (2435, 3203) | 231.66 (189.98, 271.58) | 0.23 (-0.06, 0.51) | 0.116 |
| Syrian Arab Republic | 176 (131, 222) | 79.54 (56.39, 107.07) | 478 (343, 624) | 84.83 (57.8, 117.09) | 0.19 (-0.19, 0.58) | 0.33 |
| Taiwan (Province of China) | 1388 (1299, 1467) | 185.2 (166.86, 204.2) | 6136 (5419, 6711) | 240.48 (203.28, 275.83) | 0.74 (0.46, 1.01) | <0.001 |
| Tajikistan | 99 (78, 122) | 78.9 (61.31, 99.36) | 95 (63, 138) | 47.18 (30.55, 69.13) | -1.72 (-2.31, -1.13) | <0.001 |
| Thailand | 3395 (2763, 4053) | 223.1 (168.07, 288.77) | 12143 (9411, 15073) | 193.54 (139.88, 258.98) | -0.45 (-0.79, -0.12) | 0.008 |
| Timor-Leste | 7 (5, 10) | 85.66 (54.71, 128.59) | 46 (35, 60) | 102.73 (72.1, 143.75) | 0.58 (0.48, 0.67) | <0.001 |
| Togo | 24 (18, 31) | 46.22 (31.65, 64.2) | 77 (51, 110) | 52.72 (34.1, 77.46) | 0.42 (0.3, 0.54) | <0.001 |
| Tokelau | 0 (0, 0) | 162.83 (118.05, 220.45) | 0 (0, 0) | 174.84 (123.94, 236.11) | 0.24 (0.04, 0.44) | 0.017 |
| Tonga | 6 (4, 7) | 213.38 (157.5, 282.68) | 10 (8, 12) | 223.46 (162.45, 296.87) | 0.17 (-0.14, 0.48) | 0.277 |
| Trinidad and Tobago | 37 (35, 40) | 80.21 (70.73, 90.74) | 76 (59, 94) | 67.81 (51.34, 86.47) | -0.57 (-0.72, -0.43) | <0.001 |
| Tunisia | 335 (268, 414) | 134.05 (95.95, 180.77) | 797 (547, 1097) | 113.96 (71.94, 166.48) | -0.61 (-0.84, -0.38) | <0.001 |
| Turkey | 4145 (3193, 5342) | 275.31 (198.11, 377.38) | 11243 (8835, 14085) | 221.13 (163.17, 292.43) | -0.77 (-1.15, -0.38) | <0.001 |
| Turkmenistan | 56 (51, 62) | 65.6 (57.34, 73.53) | 63 (49, 81) | 41.01 (31.52, 52.09) | -1.44 (-2.19, -0.68) | <0.001 |
| Tuvalu | 0 (0, 1) | 140.43 (99.49, 213.26) | 1 (1, 1) | 168.95 (121.71, 234.55) | 0.61 (0.56, 0.66) | <0.001 |
| Uganda | 112 (89, 138) | 37.35 (27.27, 49.63) | 275 (207, 358) | 45.32 (31.28, 63.25) | 0.64 (0.52, 0.77) | <0.001 |
| Ukraine | 7142 (6646, 7617) | 176.69 (158.65, 194.86) | 4659 (3555, 5985) | 97.37 (72.7, 128.47) | -1.98 (-2.68, -1.28) | <0.001 |
| United Arab Emirates | 15 (10, 22) | 122.36 (78.86, 178.8) | 71 (52, 103) | 143.52 (97.79, 212.08) | 0.4 (-3.38, 4.33) | 0.837 |
| United Kingdom | 24460 (23322, 25100) | 389.68 (369.47, 402.8) | 32601 (28898, 34398) | 347.68 (311.07, 369.91) | -0.4 (-0.64, -0.16) | 0.001 |
| United Republic of Tanzania | 201 (156, 263) | 39.8 (29.09, 55.84) | 496 (363, 655) | 44.66 (31.43, 61.97) | 0.39 (0.29, 0.48) | <0.001 |
| United States of America | 83558 (77057, 87104) | 394.92 (362.99, 413.23) | 130178 (114722, 138797) | 338.05 (295.79, 361.65) | -0.5 (-0.65, -0.35) | <0.001 |
| United States Virgin Islands | 5 (4, 6) | 126.12 (93.46, 166.49) | 12 (9, 17) | 98.31 (68.05, 138.78) | -0.86 (-1.36, -0.34) | 0.001 |
| Uruguay | 564 (521, 601) | 229 (202.84, 256.06) | 813 (737, 893) | 221.13 (192.47, 251.19) | -0.08 (-0.25, 0.09) | 0.375 |
| Uzbekistan | 371 (331, 410) | 68.88 (60.97, 77.44) | 358 (297, 431) | 37.72 (30.83, 45.8) | -1.75 (-2.48, -1.02) | <0.001 |
| Vanuatu | 3 (2, 5) | 126.18 (75.82, 217.38) | 9 (6, 14) | 129.63 (81.68, 211.97) | 0.1 (0, 0.19) | 0.051 |
| Venezuela (Bolivarian Republic of) | 549 (505, 590) | 115.72 (102.37, 128.71) | 2009 (1571, 2516) | 126.62 (97, 160.14) | 0.37 (-0.33, 1.07) | 0.302 |
| Viet Nam | 2150 (1732, 2651) | 104.18 (78.16, 138.31) | 5807 (4502, 6950) | 129.72 (93.73, 169.19) | 0.74 (0.57, 0.91) | <0.001 |
| Yemen | 101 (61, 177) | 50.15 (28.56, 90.13) | 402 (248, 656) | 67.95 (39.72, 112.53) | 1.02 (0.89, 1.15) | <0.001 |
| Zambia | 58 (45, 70) | 48.15 (35, 64.72) | 173 (131, 239) | 63.39 (43.63, 92.13) | 0.93 (0.81, 1.05) | <0.001 |
| Zimbabwe | 152 (122, 180) | 81.88 (61.16, 106.15) | 244 (193, 313) | 88.97 (65.44, 118.89) | 0.31 (0.18, 0.44) | <0.001 |

Notes: ASIR rates are reported per 100,000 population.

Abbreviations: ASIR, age-standardized incidence rate; EOLC, early-onset lung cancer; MOLC, middle-onset lung cancer; LOLC, late-onset lung cancer; AAPC, average annual percentage change; UI, uncertainty interval; CI, confidence interval.

# **Supplementary Table S6.** Mortality number and ASMR of EOLC, MOLC and LOLC in 1990 and 2021, and AAPC from 1990 to 2021 among 204 countries and territories.

| Country/Territory | Cases (95%UI), 1990 | ASMR (95% UI), 1990 | Cases (95%UI), 2021 | ASMR (95% UI), 2021 | AAPC (95% CI), 1990-2021 | P value |
| --- | --- | --- | --- | --- | --- | --- |
| EOLC |  |  |  |  |  |  |
| Afghanistan | 60 (29, 95) | 1.74 (0.83, 3.05) | 247 (147, 365) | 2.18 (1.24, 3.52) | 0.76 (0.64, 0.88) | <0.001 |
| Albania | 46 (36, 57) | 3.62 (2.71, 4.63) | 41 (29, 56) | 3.23 (2.24, 4.45) | -0.49 (-1.46, 0.49) | 0.327 |
| Algeria | 85 (63, 109) | 1.15 (0.77, 1.63) | 191 (135, 268) | 0.84 (0.55, 1.25) | -0.98 (-1.06, -0.9) | <0.001 |
| American Samoa | 1 (1, 1) | 4.36 (2.98, 6.2) | 1 (1, 1) | 4.28 (2.87, 6.06) | -0.03 (-0.27, 0.21) | 0.802 |
| Andorra | 2 (1, 2) | 5.46 (3.45, 8.31) | 2 (1, 3) | 2.67 (1.64, 4.13) | -2.49 (-2.95, -2.02) | <0.001 |
| Angola | 59 (39, 86) | 1.86 (1.15, 2.89) | 178 (121, 256) | 1.72 (1.08, 2.58) | -0.23 (-0.69, 0.22) | 0.315 |
| Antigua and Barbuda | 0 (0, 0) | 1.38 (1.18, 1.62) | 0 (0, 0) | 0.85 (0.72, 0.99) | -0.95 (-1.82, -0.08) | 0.033 |
| Argentina | 976 (878, 1076) | 6.6 (5.58, 7.76) | 514 (450, 580) | 2.15 (1.77, 2.58) | -3.58 (-4.1, -3.06) | <0.001 |
| Armenia | 106 (98, 113) | 8.71 (8.06, 9.33) | 49 (43, 56) | 3.2 (2.8, 3.65) | -3.05 (-3.69, -2.41) | <0.001 |
| Australia | 281 (251, 309) | 3.26 (2.84, 3.68) | 245 (215, 280) | 1.81 (1.54, 2.12) | -1.77 (-2.35, -1.18) | <0.001 |
| Austria | 211 (192, 230) | 5.06 (4.41, 5.82) | 86 (77, 97) | 1.77 (1.5, 2.09) | -3.52 (-4.14, -2.89) | <0.001 |
| Azerbaijan | 139 (113, 170) | 5.93 (4.68, 7.43) | 155 (103, 220) | 2.81 (1.87, 3.93) | -2.38 (-3.25, -1.52) | <0.001 |
| Bahamas | 3 (3, 4) | 3.19 (2.71, 3.74) | 6 (5, 8) | 2.75 (2.07, 3.71) | -0.42 (-1.15, 0.32) | 0.262 |
| Bahrain | 6 (5, 8) | 3.09 (2.26, 4.1) | 20 (15, 27) | 1.86 (1.29, 2.63) | -1.6 (-1.92, -1.27) | <0.001 |
| Bangladesh | 412 (293, 598) | 1.24 (0.78, 1.95) | 716 (490, 1120) | 0.91 (0.59, 1.48) | -0.99 (-1.32, -0.66) | <0.001 |
| Barbados | 2 (2, 2) | 1.56 (1.35, 1.78) | 2 (1, 2) | 1.15 (0.85, 1.53) | -0.9 (-1.82, 0.02) | 0.055 |
| Belarus | 312 (269, 356) | 7 (5.87, 8.16) | 151 (112, 191) | 2.86 (2.07, 3.69) | -2.78 (-3.75, -1.8) | <0.001 |
| Belgium | 344 (313, 376) | 7.11 (6.11, 8.13) | 164 (146, 183) | 2.77 (2.34, 3.26) | -3.25 (-3.57, -2.93) | <0.001 |
| Belize | 1 (1, 1) | 1.45 (1.26, 1.67) | 4 (3, 5) | 1.9 (1.54, 2.32) | 0.99 (0.11, 1.88) | 0.027 |
| Benin | 10 (7, 12) | 0.71 (0.48, 1.01) | 29 (20, 43) | 0.67 (0.41, 1.06) | -0.27 (-0.42, -0.13) | <0.001 |
| Bermuda | 2 (1, 2) | 5.25 (4.32, 6.33) | 1 (1, 1) | 2.64 (1.97, 3.36) | -2.36 (-2.7, -2.01) | <0.001 |
| Bhutan | 2 (1, 3) | 0.9 (0.47, 1.41) | 3 (2, 5) | 0.88 (0.51, 1.4) | -0.15 (-0.22, -0.08) | <0.001 |
| Bolivia (Plurinational State of) | 51 (38, 66) | 2.25 (1.55, 3.16) | 91 (61, 130) | 1.64 (1.03, 2.44) | -0.99 (-1.06, -0.91) | <0.001 |
| Bosnia and Herzegovina | 124 (95, 157) | 6.08 (4.67, 7.74) | 73 (47, 101) | 3.87 (2.5, 5.4) | -1.79 (-2.46, -1.12) | <0.001 |
| Botswana | 10 (7, 15) | 2.79 (1.68, 4.37) | 26 (17, 36) | 2.2 (1.37, 3.25) | -0.75 (-1.19, -0.31) | 0.001 |
| Brazil | 1570 (1513, 1638) | 2.69 (2.54, 2.85) | 2184 (2070, 2293) | 1.8 (1.69, 1.92) | -1.3 (-1.61, -1) | <0.001 |
| Brunei Darussalam | 4 (3, 5) | 3.83 (2.64, 5.39) | 8 (6, 10) | 3.03 (2.2, 4.03) | -0.76 (-1.26, -0.26) | 0.003 |
| Bulgaria | 382 (333, 435) | 8.35 (7.03, 9.79) | 228 (183, 277) | 5.57 (4.44, 6.81) | -1.28 (-2.28, -0.26) | 0.014 |
| Burkina Faso | 17 (12, 25) | 0.61 (0.39, 1) | 47 (32, 71) | 0.65 (0.4, 1.06) | 0.2 (-0.04, 0.44) | 0.108 |
| Burundi | 19 (13, 28) | 1.25 (0.79, 1.96) | 37 (24, 56) | 0.88 (0.52, 1.46) | -1.11 (-1.39, -0.84) | <0.001 |
| Cabo Verde | 2 (1, 2) | 1.91 (1.34, 2.63) | 5 (3, 7) | 1.73 (1.12, 2.64) | -0.38 (-0.87, 0.12) | 0.135 |
| Cambodia | 109 (79, 150) | 3.43 (2.27, 5.11) | 241 (166, 347) | 3.2 (2.09, 4.79) | -0.22 (-0.34, -0.09) | 0.001 |
| Cameroon | 29 (22, 36) | 0.93 (0.65, 1.3) | 110 (67, 157) | 0.99 (0.57, 1.53) | 0.19 (0.08, 0.31) | 0.001 |
| Canada | 833 (761, 899) | 5.83 (5.16, 6.48) | 309 (267, 356) | 1.63 (1.38, 1.94) | -4.11 (-4.41, -3.82) | <0.001 |
| Central African Republic | 17 (10, 32) | 1.98 (1.11, 3.87) | 35 (18, 71) | 1.77 (0.85, 3.7) | -0.4 (-0.69, -0.11) | 0.007 |
| Chad | 9 (6, 13) | 0.53 (0.34, 0.83) | 35 (24, 52) | 0.73 (0.45, 1.14) | 1.17 (1.06, 1.27) | <0.001 |
| Chile | 165 (140, 195) | 2.87 (2.41, 3.41) | 142 (115, 175) | 1.44 (1.15, 1.79) | -2.14 (-3.14, -1.14) | <0.001 |
| China | 32520 (27890, 37748) | 6.3 (5.31, 7.35) | 40816 (31721, 50083) | 5.03 (3.92, 6.23) | -0.74 (-0.9, -0.58) | <0.001 |
| Colombia | 307 (279, 336) | 2.4 (2.07, 2.77) | 289 (233, 355) | 1.17 (0.9, 1.49) | -2.35 (-3.09, -1.6) | <0.001 |
| Comoros | 2 (1, 2) | 1.11 (0.66, 1.67) | 4 (2, 5) | 1.14 (0.68, 1.73) | 0 (-1.05, 1.05) | 0.993 |
| Congo | 18 (11, 25) | 2.7 (1.56, 4.05) | 49 (31, 76) | 2.03 (1.23, 3.21) | -0.79 (-1.4, -0.18) | 0.011 |
| Cook Islands | 0 (0, 0) | 4.3 (2.91, 6.28) | 0 (0, 0) | 3.82 (2.51, 5.74) | -0.39 (-0.5, -0.28) | <0.001 |
| Costa Rica | 14 (13, 16) | 1.28 (1.09, 1.5) | 25 (21, 30) | 1.03 (0.84, 1.26) | -1 (-1.91, -0.08) | 0.033 |
| Côte d'Ivoire | 17 (12, 23) | 0.47 (0.31, 0.68) | 50 (33, 76) | 0.48 (0.29, 0.79) | -2.69 (-3.53, -1.84) | <0.001 |
| Croatia | 191 (170, 216) | 7.62 (6.45, 8.92) | 76 (63, 92) | 3.32 (2.7, 4.02) | -2.06 (-2.46, -1.66) | <0.001 |
| Cuba | 278 (252, 302) | 5.38 (4.64, 6.19) | 177 (145, 213) | 2.86 (2.3, 3.5) | -0.25 (-0.84, 0.35) | 0.42 |
| Cyprus | 7 (5, 9) | 1.83 (1.25, 2.55) | 14 (10, 18) | 1.7 (1.17, 2.36) | -4.4 (-4.89, -3.91) | <0.001 |
| Czechia | 499 (436, 564) | 8.65 (7.42, 9.99) | 143 (116, 174) | 2.14 (1.73, 2.62) | 0.05 (-0.24, 0.35) | 0.729 |
| Democratic People's Republic of Korea | 502 (339, 734) | 5.09 (3.22, 7.75) | 740 (449, 1205) | 4.88 (2.77, 8.25) | -0.11 (-0.21, -0.02) | 0.019 |
| Democratic Republic of the Congo | 132 (84, 214) | 1.23 (0.73, 2.08) | 342 (197, 608) | 1.13 (0.6, 2.13) | -0.29 (-0.48, -0.09) | 0.004 |
| Denmark | 187 (173, 201) | 6.31 (5.57, 7.1) | 65 (57, 74) | 2.19 (1.86, 2.57) | -3.38 (-3.94, -2.82) | <0.001 |
| Djibouti | 2 (1, 2) | 1.2 (0.72, 1.91) | 9 (5, 14) | 1.46 (0.82, 2.42) | 0.59 (0.43, 0.74) | <0.001 |
| Dominica | 1 (0, 1) | 2.14 (1.61, 2.82) | 1 (1, 1) | 2.51 (1.62, 3.64) | 0.51 (0.35, 0.68) | <0.001 |
| Dominican Republic | 53 (44, 65) | 2.14 (1.65, 2.81) | 144 (100, 202) | 2.68 (1.78, 3.82) | 0.84 (0, 1.69) | 0.051 |
| Ecuador | 61 (56, 67) | 1.63 (1.38, 1.92) | 107 (81, 139) | 1.24 (0.9, 1.68) | -0.86 (-2.38, 0.7) | 0.278 |
| Egypt | 340 (282, 413) | 1.59 (1.19, 2.1) | 1096 (834, 1420) | 2.33 (1.65, 3.19) | 1.18 (0.82, 1.54) | <0.001 |
| El Salvador | 30 (26, 36) | 1.67 (1.33, 2.06) | 49 (37, 64) | 1.67 (1.21, 2.23) | -0.08 (-1.07, 0.93) | 0.882 |
| Equatorial Guinea | 2 (1, 4) | 1.88 (1.06, 3.33) | 10 (6, 16) | 2.04 (1.18, 3.36) | 0.36 (-0.45, 1.18) | 0.386 |
| Eritrea | 16 (11, 23) | 1.49 (0.97, 2.25) | 43 (26, 65) | 1.66 (0.97, 2.61) | 0.33 (0.12, 0.54) | 0.002 |
| Estonia | 57 (51, 65) | 7.42 (6.32, 8.69) | 15 (12, 18) | 2.07 (1.6, 2.5) | -4 (-4.87, -3.12) | <0.001 |
| Eswatini | 6 (4, 10) | 2.65 (1.45, 4.63) | 18 (10, 29) | 4.16 (2.01, 7.08) | 1.51 (1.17, 1.86) | <0.001 |
| Ethiopia | 311 (211, 455) | 2.14 (1.41, 3.25) | 392 (307, 489) | 1.07 (0.8, 1.37) | -2.21 (-2.33, -2.1) | <0.001 |
| Fiji | 5 (4, 7) | 1.68 (1.11, 2.44) | 6 (4, 9) | 1.35 (0.85, 2.06) | -0.71 (-0.88, -0.54) | <0.001 |
| Finland | 99 (90, 107) | 3.38 (2.91, 3.92) | 45 (40, 50) | 1.71 (1.44, 2.02) | -1.94 (-2.63, -1.24) | <0.001 |
| France | 1861 (1656, 2087) | 6.68 (5.81, 7.68) | 1378 (1215, 1556) | 4.07 (3.51, 4.66) | -1.53 (-1.65, -1.41) | <0.001 |
| Gabon | 7 (4, 10) | 2.44 (1.35, 3.89) | 16 (11, 24) | 2.23 (1.31, 3.5) | -0.18 (-0.44, 0.07) | 0.157 |
| Gambia | 1 (1, 1) | 0.38 (0.24, 0.58) | 3 (2, 5) | 0.39 (0.24, 0.62) | 0.11 (-1.13, 1.36) | 0.864 |
| Georgia | 159 (139, 184) | 6.76 (5.83, 7.81) | 72 (61, 85) | 3.91 (3.29, 4.62) | -1.7 (-3.15, -0.23) | 0.023 |
| Germany | 2434 (2211, 2669) | 5.84 (5.08, 6.69) | 940 (839, 1054) | 2.29 (1.96, 2.65) | -3.04 (-3.42, -2.66) | <0.001 |
| Ghana | 29 (22, 39) | 0.6 (0.4, 0.88) | 66 (47, 91) | 0.48 (0.31, 0.72) | -0.73 (-0.83, -0.62) | <0.001 |
| Greece | 305 (279, 332) | 5.9 (5.2, 6.65) | 184 (168, 202) | 3.08 (2.74, 3.44) | -2.06 (-2.33, -1.8) | <0.001 |
| Greenland | 5 (3, 6) | 15.65 (11.47, 20.96) | 2 (1, 2) | 7.13 (4.85, 9.83) | -2.43 (-2.63, -2.23) | <0.001 |
| Grenada | 1 (0, 1) | 2.2 (1.75, 2.7) | 1 (1, 1) | 1.64 (1.28, 2.1) | -0.87 (-1.91, 0.18) | 0.105 |
| Guam | 3 (2, 3) | 4.48 (3.5, 5.65) | 5 (4, 5) | 5.81 (4.53, 7.4) | 0.89 (0.11, 1.68) | 0.025 |
| Guatemala | 46 (43, 49) | 1.75 (1.54, 1.96) | 75 (63, 88) | 1.1 (0.9, 1.33) | -1.46 (-2.56, -0.34) | 0.011 |
| Guinea | 17 (13, 23) | 0.89 (0.61, 1.3) | 46 (30, 68) | 1.12 (0.67, 1.74) | 0.74 (0.56, 0.92) | <0.001 |
| Guinea-Bissau | 3 (2, 5) | 1.17 (0.71, 1.85) | 8 (6, 12) | 1.18 (0.72, 1.82) | 0.02 (-0.08, 0.12) | 0.739 |
| Guyana | 3 (3, 4) | 1.3 (1.02, 1.63) | 5 (4, 7) | 1.52 (1.04, 2.11) | 0.68 (0.15, 1.2) | 0.012 |
| Haiti | 51 (32, 74) | 2.39 (1.42, 3.67) | 97 (58, 150) | 1.72 (0.98, 2.88) | -0.95 (-1.06, -0.83) | <0.001 |
| Honduras | 36 (28, 46) | 2.5 (1.8, 3.48) | 103 (61, 160) | 2.44 (1.33, 4.11) | -0.07 (-0.28, 0.14) | 0.497 |
| Hungary | 698 (612, 790) | 12.38 (10.68, 14.31) | 240 (194, 293) | 3.92 (3.15, 4.8) | -3.95 (-4.46, -3.44) | <0.001 |
| Iceland | 5 (4, 5) | 3.98 (3.42, 4.61) | 6 (5, 7) | 3.53 (2.95, 4.15) | -0.48 (-1.2, 0.25) | 0.196 |
| India | 3634 (3155, 4222) | 1.08 (0.93, 1.27) | 8736 (7356, 10136) | 1.25 (1.04, 1.48) | 0.45 (0.16, 0.74) | 0.002 |
| Indonesia | 1964 (1536, 2366) | 2.73 (2.11, 3.33) | 4662 (3220, 6477) | 2.97 (2.03, 4.09) | 0.3 (0.23, 0.36) | <0.001 |
| Iran (Islamic Republic of) | 323 (257, 383) | 1.8 (1.44, 2.17) | 855 (770, 961) | 1.69 (1.5, 1.93) | -0.28 (-0.43, -0.14) | <0.001 |
| Iraq | 124 (86, 172) | 2.27 (1.47, 3.4) | 367 (250, 520) | 1.9 (1.22, 2.86) | -0.61 (-0.79, -0.43) | <0.001 |
| Ireland | 66 (60, 72) | 4.05 (3.45, 4.71) | 67 (58, 76) | 2.33 (1.93, 2.76) | -1.9 (-2.96, -0.83) | 0.001 |
| Israel | 66 (59, 74) | 3.33 (2.84, 3.87) | 77 (67, 88) | 1.65 (1.4, 1.93) | -2.03 (-2.33, -1.73) | <0.001 |
| Italy | 1470 (1406, 1529) | 5.04 (4.78, 5.3) | 670 (631, 707) | 1.94 (1.81, 2.06) | -3.25 (-3.6, -2.91) | <0.001 |
| Jamaica | 22 (19, 25) | 2.93 (2.34, 3.57) | 31 (22, 43) | 2.19 (1.48, 3.14) | -0.91 (-2.92, 1.14) | 0.38 |
| Japan | 2026 (1969, 2089) | 2.69 (2.6, 2.79) | 918 (889, 947) | 1.29 (1.24, 1.34) | -2.4 (-2.84, -1.97) | <0.001 |
| Jordan | 24 (18, 30) | 2.22 (1.51, 3.06) | 94 (69, 129) | 1.56 (1.05, 2.33) | -1.06 (-1.43, -0.69) | <0.001 |
| Kazakhstan | 602 (535, 673) | 9.8 (8.61, 11.08) | 221 (193, 250) | 2.28 (1.98, 2.6) | -4.56 (-4.99, -4.12) | <0.001 |
| Kenya | 18 (12, 27) | 0.3 (0.2, 0.44) | 89 (69, 119) | 0.48 (0.36, 0.64) | 1.48 (1.32, 1.63) | <0.001 |
| Kiribati | 1 (1, 1) | 2.64 (1.77, 3.8) | 2 (1, 2) | 3.21 (1.94, 5.08) | 0.64 (0.54, 0.74) | <0.001 |
| Kuwait | 11 (10, 13) | 1.51 (1.21, 1.83) | 30 (24, 40) | 0.86 (0.64, 1.14) | -1.7 (-2.34, -1.06) | <0.001 |
| Kyrgyzstan | 88 (75, 100) | 6.56 (5.44, 7.69) | 56 (44, 69) | 1.83 (1.44, 2.28) | -4.3 (-4.56, -4.05) | <0.001 |
| Lao People's Democratic Republic | 51 (32, 81) | 3.76 (2.22, 6.24) | 100 (66, 148) | 2.97 (1.86, 4.67) | -0.72 (-0.82, -0.63) | <0.001 |
| Latvia | 91 (80, 105) | 6.68 (5.62, 7.9) | 25 (20, 31) | 2.51 (1.97, 3.11) | -3.16 (-4.58, -1.72) | <0.001 |
| Lebanon | 38 (27, 52) | 3.22 (2.18, 4.63) | 73 (54, 96) | 2.56 (1.77, 3.53) | -0.63 (-0.9, -0.37) | <0.001 |
| Lesotho | 8 (5, 12) | 1.54 (0.94, 2.55) | 31 (19, 48) | 4.5 (2.61, 7.47) | 3.57 (3.29, 3.86) | <0.001 |
| Liberia | 5 (3, 7) | 0.64 (0.42, 0.99) | 15 (8, 26) | 0.65 (0.33, 1.17) | 0.03 (-0.27, 0.32) | 0.867 |
| Libya | 42 (30, 58) | 3.29 (2.12, 4.98) | 154 (106, 227) | 3.47 (2.18, 5.44) | 0.16 (0.02, 0.3) | 0.029 |
| Lithuania | 115 (103, 128) | 6.42 (5.42, 7.46) | 40 (33, 49) | 2.82 (2.19, 3.49) | -2.84 (-4.26, -1.4) | <0.001 |
| Luxembourg | 13 (11, 14) | 6.13 (5.38, 6.91) | 8 (7, 9) | 2.16 (1.84, 2.49) | -3.38 (-4.08, -2.69) | <0.001 |
| Madagascar | 34 (26, 42) | 1 (0.7, 1.39) | 93 (65, 129) | 0.91 (0.57, 1.37) | -0.24 (-0.35, -0.14) | <0.001 |
| Malawi | 9 (6, 11) | 0.31 (0.21, 0.45) | 26 (18, 36) | 0.41 (0.26, 0.64) | 0.94 (0.7, 1.19) | <0.001 |
| Malaysia | 134 (108, 167) | 2.03 (1.51, 2.71) | 391 (306, 499) | 2.48 (1.81, 3.34) | 0.76 (-0.05, 1.58) | 0.066 |
| Maldives | 1 (1, 2) | 1.84 (1.03, 2.78) | 3 (2, 4) | 0.83 (0.55, 1.2) | -2.65 (-2.83, -2.48) | <0.001 |
| Mali | 15 (12, 19) | 0.56 (0.4, 0.77) | 40 (28, 56) | 0.58 (0.38, 0.86) | 0.12 (-0.07, 0.31) | 0.227 |
| Malta | 5 (4, 6) | 2.52 (2.05, 3.02) | 5 (5, 6) | 2.25 (1.81, 2.8) | -0.43 (-1.11, 0.25) | 0.213 |
| Marshall Islands | 1 (0, 1) | 3.97 (2.48, 6.26) | 1 (1, 2) | 5.09 (2.84, 8.04) | 0.8 (0.69, 0.91) | <0.001 |
| Mauritania | 5 (4, 7) | 0.87 (0.56, 1.3) | 11 (7, 16) | 0.72 (0.44, 1.15) | -0.66 (-0.85, -0.47) | <0.001 |
| Mauritius | 10 (9, 11) | 2.05 (1.79, 2.35) | 11 (10, 12) | 1.56 (1.32, 1.79) | -1.07 (-2.69, 0.57) | 0.199 |
| Mexico | 570 (553, 587) | 1.85 (1.77, 1.92) | 710 (628, 802) | 1.04 (0.92, 1.18) | -1.88 (-2.57, -1.18) | <0.001 |
| Micronesia (Federated States of) | 2 (1, 2) | 4.92 (3.01, 7.8) | 3 (2, 4) | 5.42 (3.14, 8.52) | 0.33 (0.26, 0.4) | <0.001 |
| Monaco | 1 (1, 2) | 7.75 (5.55, 10.55) | 2 (1, 3) | 9.86 (6.38, 14.32) | 0.76 (0.58, 0.94) | <0.001 |
| Mongolia | 29 (22, 38) | 4.37 (3.07, 6.13) | 62 (47, 79) | 3.58 (2.52, 4.95) | -0.69 (-1.53, 0.15) | 0.109 |
| Montenegro | 23 (18, 28) | 8.32 (6.47, 10.38) | 23 (17, 29) | 6.68 (4.96, 8.69) | -0.73 (-1.29, -0.17) | 0.011 |
| Morocco | 135 (100, 182) | 1.57 (1.03, 2.33) | 227 (150, 352) | 1.2 (0.73, 1.91) | -0.88 (-0.95, -0.82) | <0.001 |
| Mozambique | 19 (14, 26) | 0.45 (0.3, 0.65) | 67 (44, 100) | 0.72 (0.42, 1.15) | 1.53 (1.38, 1.67) | <0.001 |
| Myanmar | 505 (338, 695) | 3.48 (2.21, 5.18) | 707 (487, 1020) | 2.53 (1.63, 3.71) | -1.03 (-1.11, -0.94) | <0.001 |
| Namibia | 4 (3, 5) | 0.87 (0.6, 1.22) | 10 (6, 15) | 0.99 (0.6, 1.52) | 0.49 (0.09, 0.89) | 0.015 |
| Nauru | 0 (0, 0) | 7.15 (3.46, 11.11) | 0 (0, 0) | 7.45 (3.52, 11.11) | 0.14 (0.07, 0.2) | <0.001 |
| Nepal | 68 (44, 101) | 1 (0.61, 1.6) | 128 (87, 190) | 0.94 (0.58, 1.49) | -0.19 (-0.38, 0.01) | 0.064 |
| Netherlands | 421 (391, 453) | 5.25 (4.61, 5.96) | 232 (208, 258) | 2.71 (2.36, 3.11) | -2.04 (-2.14, -1.95) | <0.001 |
| New Zealand | 61 (55, 67) | 3.67 (3.25, 4.11) | 55 (49, 62) | 2.16 (1.88, 2.45) | -1.8 (-2.27, -1.34) | <0.001 |
| Nicaragua | 11 (9, 13) | 0.94 (0.71, 1.22) | 22 (17, 28) | 0.71 (0.49, 0.99) | -1.19 (-1.62, -0.76) | <0.001 |
| Niger | 11 (8, 17) | 0.49 (0.31, 0.79) | 27 (17, 47) | 0.42 (0.24, 0.77) | -0.46 (-0.7, -0.22) | <0.001 |
| Nigeria | 47 (32, 63) | 0.16 (0.11, 0.22) | 123 (83, 168) | 0.16 (0.11, 0.23) | 0.01 (-0.1, 0.12) | 0.863 |
| Niue | 0 (0, 0) | 3.38 (2.13, 5.16) | 0 (0, 0) | 3.76 (2.31, 5.91) | 0.35 (0.03, 0.66) | 0.031 |
| North Macedonia | 57 (44, 70) | 5.98 (4.61, 7.56) | 53 (38, 70) | 3.95 (2.87, 5.25) | -1.18 (-1.68, -0.67) | <0.001 |
| Northern Mariana Islands | 2 (1, 2) | 6.9 (4.2, 10.46) | 1 (1, 2) | 4.62 (3.19, 6.67) | -1.28 (-1.46, -1.09) | <0.001 |
| Norway | 60 (57, 63) | 2.71 (2.54, 2.89) | 47 (44, 50) | 1.62 (1.49, 1.75) | -1.55 (-1.86, -1.23) | <0.001 |
| Oman | 5 (3, 7) | 0.7 (0.42, 1.07) | 11 (7, 15) | 0.38 (0.25, 0.59) | -2.08 (-2.49, -1.68) | <0.001 |
| Pakistan | 634 (500, 778) | 1.76 (1.31, 2.27) | 2020 (1509, 2676) | 2.1 (1.5, 2.9) | 0.58 (0.46, 0.7) | <0.001 |
| Palau | 0 (0, 1) | 7.1 (4.75, 10.5) | 1 (1, 1) | 7.27 (5, 10.34) | 0.06 (-0.26, 0.39) | 0.701 |
| Palestine | 16 (11, 23) | 2.87 (1.77, 4.35) | 46 (37, 58) | 2.23 (1.59, 3.08) | -0.86 (-1.16, -0.57) | <0.001 |
| Panama | 17 (16, 19) | 1.85 (1.62, 2.09) | 22 (17, 28) | 1.07 (0.82, 1.35) | -1.66 (-2.62, -0.7) | 0.001 |
| Papua New Guinea | 34 (20, 58) | 2.33 (1.26, 4.27) | 109 (68, 178) | 2.42 (1.32, 4.21) | 0.14 (0.05, 0.23) | 0.003 |
| Paraguay | 20 (16, 25) | 1.45 (1.07, 1.93) | 55 (39, 75) | 1.67 (1.13, 2.4) | 0.66 (0.31, 1) | <0.001 |
| Peru | 209 (170, 257) | 2.57 (1.92, 3.35) | 319 (221, 428) | 1.74 (1.16, 2.44) | -1.19 (-2.85, 0.5) | 0.166 |
| Philippines | 739 (657, 828) | 3.25 (2.84, 3.72) | 1430 (1173, 1684) | 2.7 (2.21, 3.24) | -0.64 (-0.76, -0.52) | <0.001 |
| Poland | 1528 (1476, 1580) | 8.81 (8.43, 9.2) | 624 (569, 681) | 2.75 (2.49, 3.01) | -3.92 (-4.26, -3.59) | <0.001 |
| Portugal | 190 (169, 212) | 3.91 (3.31, 4.62) | 146 (129, 165) | 2.28 (1.92, 2.68) | -1.79 (-2.67, -0.89) | <0.001 |
| Puerto Rico | 44 (40, 50) | 2.58 (2.17, 3.05) | 26 (20, 32) | 1.54 (1.16, 1.97) | -1.87 (-2.04, -1.71) | <0.001 |
| Qatar | 5 (4, 6) | 1.96 (1.31, 2.81) | 24 (18, 35) | 1.11 (0.73, 1.69) | -1.88 (-3.17, -0.58) | 0.005 |
| Republic of Korea | 872 (725, 1031) | 4.18 (3.25, 5.31) | 497 (403, 611) | 1.57 (1.2, 2.02) | -3.15 (-3.4, -2.91) | <0.001 |
| Republic of Moldova | 128 (115, 142) | 6.65 (5.73, 7.65) | 53 (45, 62) | 2.42 (1.99, 2.91) | -3.36 (-4.11, -2.61) | <0.001 |
| Romania | 821 (697, 959) | 7.62 (6.37, 9.03) | 522 (419, 637) | 4.56 (3.63, 5.58) | -1.61 (-2.03, -1.2) | <0.001 |
| Russian Federation | 4784 (4654, 4903) | 7.46 (7.2, 7.7) | 2463 (2231, 2676) | 2.95 (2.67, 3.21) | -2.95 (-3.39, -2.51) | <0.001 |
| Rwanda | 28 (20, 38) | 1.5 (0.96, 2.17) | 53 (34, 80) | 1.07 (0.62, 1.73) | -1.12 (-1.43, -0.81) | <0.001 |
| Saint Kitts and Nevis | 0 (0, 0) | 2.11 (1.8, 2.46) | 0 (0, 0) | 1.07 (0.79, 1.43) | -2.14 (-2.42, -1.86) | <0.001 |
| Saint Lucia | 1 (1, 1) | 2.61 (2.27, 3.01) | 2 (2, 2) | 1.88 (1.46, 2.36) | -1.15 (-1.63, -0.66) | <0.001 |
| Saint Vincent and the Grenadines | 1 (1, 1) | 1.78 (1.52, 2.08) | 1 (1, 1) | 1.81 (1.48, 2.2) | 0.02 (-0.44, 0.48) | 0.937 |
| Samoa | 1 (0, 1) | 1.07 (0.69, 1.62) | 1 (1, 1) | 1.22 (0.73, 1.85) | 0.43 (0.34, 0.52) | <0.001 |
| San Marino | 0 (0, 1) | 4.09 (2.99, 5.41) | 0 (0, 1) | 2.11 (1.09, 3.41) | -2.31 (-2.49, -2.13) | <0.001 |
| Sao Tome and Principe | 0 (0, 1) | 1.49 (0.98, 2.13) | 2 (1, 3) | 1.78 (1.02, 3) | 0.55 (0.25, 0.84) | <0.001 |
| Saudi Arabia | 51 (35, 69) | 0.91 (0.57, 1.35) | 243 (168, 344) | 0.9 (0.58, 1.37) | 0.01 (-0.14, 0.16) | 0.894 |
| Senegal | 19 (14, 26) | 0.87 (0.57, 1.3) | 48 (34, 70) | 0.86 (0.54, 1.37) | 0.02 (-0.27, 0.31) | 0.894 |
| Serbia | 401 (313, 517) | 8.34 (6.13, 11.22) | 261 (197, 336) | 5.15 (3.72, 6.86) | -1.44 (-1.83, -1.05) | <0.001 |
| Seychelles | 1 (1, 1) | 3.2 (2.31, 4.35) | 1 (1, 2) | 2.12 (1.48, 2.91) | -1.19 (-1.75, -0.62) | <0.001 |
| Sierra Leone | 9 (6, 13) | 0.7 (0.45, 1.1) | 23 (14, 33) | 0.72 (0.41, 1.18) | 0.11 (-0.09, 0.32) | 0.285 |
| Singapore | 49 (44, 56) | 3.01 (2.57, 3.53) | 45 (39, 52) | 1.19 (0.97, 1.46) | -2.75 (-3.75, -1.74) | <0.001 |
| Slovakia | 188 (148, 230) | 7.53 (5.45, 9.87) | 94 (70, 121) | 2.78 (1.95, 3.73) | -3.23 (-3.96, -2.49) | <0.001 |
| Slovenia | 60 (53, 68) | 5.93 (5.09, 6.83) | 23 (18, 29) | 1.92 (1.47, 2.45) | -3.64 (-4.74, -2.54) | <0.001 |
| Solomon Islands | 4 (2, 7) | 4.27 (2.11, 7.06) | 15 (10, 21) | 5.15 (3.37, 7.5) | 0.63 (0.51, 0.74) | <0.001 |
| Somalia | 24 (14, 44) | 0.97 (0.53, 1.9) | 61 (32, 129) | 0.91 (0.44, 2.03) | -0.18 (-0.28, -0.09) | <0.001 |
| South Africa | 675 (572, 791) | 5.1 (4.21, 6.21) | 889 (773, 1020) | 3.15 (2.65, 3.76) | -1.55 (-2.02, -1.08) | <0.001 |
| South Sudan | 17 (10, 26) | 1.03 (0.58, 1.7) | 39 (26, 58) | 1.14 (0.7, 1.75) | 0.37 (-0.04, 0.78) | 0.076 |
| Spain | 1087 (990, 1200) | 5.92 (5.1, 6.89) | 684 (596, 783) | 2.32 (1.97, 2.72) | -3.08 (-3.37, -2.79) | <0.001 |
| Sri Lanka | 123 (98, 158) | 1.63 (1.18, 2.26) | 154 (97, 219) | 1.33 (0.8, 1.99) | -0.6 (-1.23, 0.03) | 0.06 |
| Sudan | 97 (66, 152) | 1.49 (0.92, 2.5) | 259 (157, 398) | 1.52 (0.86, 2.42) | 0.06 (0.02, 0.11) | 0.008 |
| Suriname | 3 (3, 4) | 2.33 (1.62, 3.15) | 7 (5, 9) | 2.42 (1.6, 3.46) | 0.23 (-1.25, 1.73) | 0.762 |
| Sweden | 131 (120, 143) | 2.64 (2.31, 3) | 56 (49, 64) | 1.09 (0.9, 1.31) | -2.99 (-3.45, -2.53) | <0.001 |
| Switzerland | 150 (135, 167) | 3.81 (3.24, 4.41) | 81 (71, 90) | 1.66 (1.39, 1.97) | -2.79 (-3.42, -2.15) | <0.001 |
| Syrian Arab Republic | 110 (81, 144) | 2.93 (2.02, 4.03) | 158 (106, 225) | 2.08 (1.35, 3.03) | -1.17 (-1.35, -0.99) | <0.001 |
| Taiwan (Province of China) | 346 (315, 380) | 3.84 (3.34, 4.41) | 513 (441, 581) | 3.51 (2.93, 4.13) | -0.36 (-0.64, -0.08) | 0.013 |
| Tajikistan | 60 (45, 76) | 4.16 (3.06, 5.54) | 71 (45, 112) | 1.65 (1.03, 2.6) | -2.98 (-3.17, -2.78) | <0.001 |
| Thailand | 1162 (949, 1435) | 4.77 (3.55, 6.38) | 1823 (1327, 2442) | 4.59 (3.14, 6.47) | -0.11 (-0.53, 0.31) | 0.604 |
| Timor-Leste | 5 (3, 7) | 1.74 (1.05, 2.73) | 10 (7, 14) | 1.86 (1.2, 2.79) | 0.24 (-0.21, 0.7) | 0.29 |
| Togo | 8 (6, 11) | 0.79 (0.5, 1.2) | 30 (17, 47) | 0.92 (0.48, 1.54) | 0.5 (0.12, 0.87) | 0.009 |
| Tokelau | 0 (0, 0) | 2.86 (1.8, 4.48) | 0 (0, 0) | 3.46 (2.21, 5.25) | 0.67 (0.5, 0.85) | <0.001 |
| Tonga | 1 (1, 2) | 3.63 (2.52, 5.14) | 2 (1, 3) | 3.94 (2.42, 6.02) | 0.27 (0.07, 0.47) | 0.009 |
| Trinidad and Tobago | 10 (9, 11) | 1.97 (1.71, 2.27) | 13 (9, 17) | 1.69 (1.23, 2.25) | -0.34 (-1.39, 0.72) | 0.526 |
| Tunisia | 63 (49, 81) | 2.27 (1.56, 3.16) | 162 (106, 231) | 2.45 (1.47, 3.72) | 0.23 (0.07, 0.4) | 0.004 |
| Turkey | 1982 (1445, 2647) | 9.25 (6.25, 13.15) | 2008 (1517, 2549) | 4.26 (3, 5.74) | -2.59 (-2.96, -2.22) | <0.001 |
| Turkmenistan | 41 (37, 44) | 3.7 (3.29, 4.11) | 54 (41, 73) | 2.18 (1.65, 2.93) | -1.5 (-2.57, -0.43) | 0.006 |
| Tuvalu | 0 (0, 0) | 3.47 (2.12, 5.6) | 0 (0, 0) | 4.12 (2.49, 6.38) | 0.57 (0.5, 0.63) | <0.001 |
| Uganda | 35 (25, 46) | 0.78 (0.52, 1.13) | 130 (86, 185) | 1.03 (0.62, 1.55) | 0.87 (0.71, 1.03) | <0.001 |
| Ukraine | 2139 (1900, 2403) | 8.71 (7.45, 10.17) | 836 (551, 1185) | 3.27 (2.13, 4.75) | -3.23 (-3.8, -2.65) | <0.001 |
| United Arab Emirates | 22 (14, 32) | 2.34 (1.42, 3.59) | 91 (62, 140) | 0.97 (0.63, 1.53) | -2.74 (-3.7, -1.78) | <0.001 |
| United Kingdom | 1240 (1218, 1266) | 4.23 (4.14, 4.33) | 675 (657, 695) | 1.95 (1.89, 2.02) | -2.52 (-2.92, -2.12) | <0.001 |
| United Republic of Tanzania | 70 (52, 100) | 0.99 (0.68, 1.49) | 214 (146, 304) | 1.05 (0.68, 1.61) | 0.16 (0, 0.34) | 0.057 |
| United States of America | 7911 (7710, 8107) | 6.25 (6.07, 6.43) | 3098 (2996, 3209) | 1.91 (1.84, 1.98) | -3.76 (-3.96, -3.56) | <0.001 |
| United States Virgin Islands | 2 (1, 2) | 3.07 (2.22, 4.09) | 1 (1, 2) | 2.87 (1.89, 4.4) | -0.1 (-0.45, 0.24) | 0.557 |
| Uruguay | 113 (98, 127) | 7.97 (6.73, 9.3) | 74 (61, 88) | 4.21 (3.44, 5.09) | -2.21 (-2.49, -1.93) | <0.001 |
| Uzbekistan | 241 (217, 266) | 3.95 (3.39, 4.54) | 243 (202, 292) | 1.41 (1.12, 1.75) | -3.19 (-4.98, -1.38) | 0.001 |
| Vanuatu | 1 (1, 3) | 2.72 (1.34, 5.27) | 4 (2, 7) | 3.04 (1.69, 5.36) | 0.34 (0.21, 0.46) | <0.001 |
| Venezuela (Bolivarian Republic of) | 236 (218, 254) | 3.44 (3.03, 3.87) | 337 (244, 453) | 2.33 (1.66, 3.17) | -1.25 (-1.67, -0.82) | <0.001 |
| Viet Nam | 549 (397, 734) | 2.84 (1.89, 4.04) | 1863 (1335, 2634) | 3.38 (2.24, 5.09) | 0.58 (0.5, 0.66) | <0.001 |
| Yemen | 63 (34, 113) | 1.73 (0.91, 3.17) | 196 (108, 336) | 1.58 (0.83, 2.79) | -0.34 (-0.54, -0.14) | 0.001 |
| Zambia | 26 (21, 33) | 1.21 (0.85, 1.7) | 123 (77, 251) | 1.86 (1.06, 3.96) | 1.4 (1.24, 1.56) | <0.001 |
| Zimbabwe | 41 (32, 52) | 1.5 (1.05, 2.03) | 150 (100, 219) | 2.58 (1.64, 3.92) | 1.84 (1.32, 2.36) | <0.001 |
| MOLC |  |  |  |  |  |  |
| Afghanistan | 367 (197, 669) | 28.18 (14.47, 52.84) | 432 (275, 622) | 28.89 (17.48, 42.73) | 0.07 (0.02, 0.12) | 0.007 |
| Albania | 333 (274, 402) | 93.37 (76, 113.62) | 482 (340, 651) | 65.88 (46.43, 89.25) | -1.23 (-1.78, -0.68) | <0.001 |
| Algeria | 495 (391, 611) | 23.27 (16.83, 31.43) | 1187 (889, 1530) | 18.88 (12.98, 26.1) | -0.69 (-0.99, -0.39) | <0.001 |
| American Samoa | 3 (3, 4) | 80.35 (61.98, 103.41) | 6 (5, 8) | 71.34 (54.26, 93.29) | -0.34 (-0.4, -0.28) | <0.001 |
| Andorra | 12 (8, 17) | 127.74 (85.65, 186.73) | 15 (10, 22) | 63.43 (40.08, 91.06) | -2.4 (-2.82, -1.97) | <0.001 |
| Angola | 319 (220, 464) | 43.75 (27.95, 65.54) | 859 (620, 1150) | 38.68 (26.08, 54.14) | -0.4 (-0.71, -0.1) | 0.01 |
| Antigua and Barbuda | 2 (2, 3) | 32.09 (27.75, 36.99) | 5 (4, 5) | 24.7 (21.25, 28.6) | -0.79 (-2.04, 0.47) | 0.219 |
| Argentina | 5340 (4897, 5831) | 100.22 (89.51, 111.49) | 5438 (4820, 6118) | 64.44 (56.41, 73.21) | -1.31 (-1.46, -1.15) | <0.001 |
| Armenia | 740 (700, 783) | 140.03 (131.23, 148.74) | 654 (575, 743) | 84.93 (74.76, 96.7) | -1.75 (-2.62, -0.87) | <0.001 |
| Australia | 3087 (2906, 3260) | 100.72 (92.98, 108.95) | 3348 (3105, 3602) | 53.01 (47.95, 58.26) | -2.08 (-2.27, -1.9) | <0.001 |
| Austria | 1592 (1516, 1665) | 91.39 (83.33, 99.41) | 1674 (1562, 1795) | 68.13 (61.2, 75.03) | -1.02 (-1.33, -0.7) | <0.001 |
| Azerbaijan | 832 (679, 1002) | 86.95 (70.93, 105.32) | 1075 (721, 1477) | 50.42 (33.83, 69.51) | -1.79 (-2.19, -1.39) | <0.001 |
| Bahamas | 14 (12, 15) | 57.08 (49.06, 65.99) | 29 (23, 37) | 39.09 (30.4, 49.98) | -1.26 (-2.29, -0.22) | 0.018 |
| Bahrain | 30 (26, 36) | 95.69 (74.9, 121) | 79 (62, 105) | 40.04 (29.28, 56.42) | -2.75 (-3.17, -2.33) | <0.001 |
| Bangladesh | 2223 (1597, 3190) | 28.99 (19.44, 43.67) | 4117 (3038, 5607) | 17.72 (12.21, 25.62) | -1.41 (-1.82, -1.01) | <0.001 |
| Barbados | 10 (9, 12) | 27.53 (23.71, 31.5) | 20 (15, 26) | 23.55 (17.14, 30.98) | -0.18 (-0.32, -0.04) | 0.015 |
| Belarus | 2878 (2642, 3105) | 123.59 (111.24, 136.68) | 2198 (1687, 2734) | 80.91 (61.83, 101.29) | -1.27 (-1.96, -0.58) | <0.001 |
| Belgium | 3675 (3492, 3866) | 152.11 (138.74, 165.18) | 2719 (2517, 2924) | 86.61 (77.08, 96.29) | -1.96 (-2.18, -1.74) | <0.001 |
| Belize | 5 (4, 5) | 31.21 (27.06, 36.06) | 17 (15, 20) | 33.4 (27.41, 39.7) | 0.34 (-0.27, 0.95) | 0.275 |
| Benin | 62 (49, 76) | 19.51 (14.34, 26.71) | 160 (116, 217) | 18.28 (12.59, 26.74) | -0.18 (-0.47, 0.1) | 0.208 |
| Bermuda | 11 (10, 12) | 107.22 (93.86, 121.95) | 11 (9, 14) | 55.86 (44.67, 69.89) | -2.43 (-2.79, -2.07) | <0.001 |
| Bhutan | 7 (4, 11) | 16.66 (8.96, 26.25) | 15 (10, 22) | 15.7 (9.68, 23.71) | -0.18 (-0.3, -0.06) | 0.003 |
| Bolivia (Plurinational State of) | 245 (180, 321) | 45.41 (31.97, 61.16) | 530 (357, 741) | 33.84 (22.33, 48.47) | -0.94 (-1.01, -0.87) | <0.001 |
| Bosnia and Herzegovina | 1046 (927, 1175) | 128.54 (108.25, 152.01) | 1223 (940, 1530) | 120.94 (91.12, 154.14) | -0.04 (-0.53, 0.45) | 0.879 |
| Botswana | 56 (40, 77) | 55.22 (36.26, 79.65) | 124 (88, 166) | 46.33 (30.46, 65.77) | -0.59 (-0.78, -0.4) | <0.001 |
| Brazil | 7869 (7604, 8122) | 51.34 (48.91, 53.89) | 17973 (17045, 18826) | 42.79 (40.04, 45.47) | -0.59 (-0.77, -0.41) | <0.001 |
| Brunei Darussalam | 16 (12, 22) | 103.18 (76.62, 138.63) | 38 (30, 46) | 56.61 (43.69, 72.09) | -1.97 (-2.58, -1.36) | <0.001 |
| Bulgaria | 2400 (2145, 2671) | 108.1 (96.41, 120.65) | 2490 (2071, 2913) | 127.31 (105.76, 149.43) | 0.62 (0.04, 1.22) | 0.037 |
| Burkina Faso | 126 (95, 180) | 16.52 (11.27, 25.29) | 269 (190, 401) | 17.16 (11.36, 26.14) | 0.13 (-0.05, 0.32) | 0.158 |
| Burundi | 101 (72, 139) | 26.43 (18.1, 38.23) | 161 (109, 245) | 18.06 (11.29, 28.79) | -1.22 (-1.39, -1.04) | <0.001 |
| Cabo Verde | 9 (8, 11) | 27.21 (20.36, 35.39) | 34 (25, 44) | 43.43 (29.81, 58.97) | 1.49 (0.87, 2.11) | <0.001 |
| Cambodia | 639 (455, 861) | 80.23 (54.31, 114.25) | 1667 (1145, 2249) | 72.87 (49.34, 102.05) | -0.29 (-0.41, -0.18) | <0.001 |
| Cameroon | 181 (147, 222) | 22.61 (16.92, 29.7) | 544 (372, 768) | 24.55 (15.32, 35.22) | 0.25 (0.13, 0.37) | <0.001 |
| Canada | 7503 (7117, 7872) | 149.61 (139.55, 159.56) | 7046 (6569, 7500) | 65.08 (59.23, 70.97) | -2.67 (-2.78, -2.55) | <0.001 |
| Central African Republic | 96 (61, 170) | 43.65 (26.12, 80.62) | 156 (94, 289) | 35.89 (20.23, 70.15) | -0.63 (-0.71, -0.55) | <0.001 |
| Chad | 65 (47, 93) | 14.29 (9.64, 21.64) | 216 (151, 315) | 21.55 (14, 32.11) | 1.36 (1.25, 1.47) | <0.001 |
| Chile | 920 (865, 974) | 55.78 (49.96, 61.86) | 1485 (1364, 1602) | 35.83 (31.51, 40.49) | -1.46 (-1.68, -1.24) | <0.001 |
| China | 149732 (125574, 174131) | 98.48 (82.05, 115.52) | 345517 (272081, 427526) | 91.48 (71.85, 113.61) | -0.25 (-0.46, -0.04) | 0.022 |
| Colombia | 1367 (1281, 1453) | 46.3 (41.98, 50.95) | 2391 (1934, 2934) | 26.34 (21, 32.41) | -2.17 (-2.28, -2.06) | <0.001 |
| Comoros | 8 (6, 12) | 23.77 (15.8, 33.92) | 18 (12, 26) | 21.81 (13.59, 32.19) | -0.31 (-0.49, -0.12) | 0.001 |
| Congo | 99 (60, 125) | 50.58 (30.02, 69.89) | 211 (151, 280) | 42.02 (28.21, 59.24) | -0.56 (-0.81, -0.31) | <0.001 |
| Cook Islands | 2 (2, 3) | 93.67 (70.39, 124.18) | 3 (2, 4) | 71.95 (53.4, 95.18) | -0.81 (-1.01, -0.61) | <0.001 |
| Costa Rica | 89 (82, 95) | 32.26 (28.75, 36.02) | 181 (156, 209) | 19.81 (16.62, 23.41) | -1.57 (-2.04, -1.1) | <0.001 |
| Côte d'Ivoire | 98 (78, 123) | 13.44 (9.86, 18.52) | 248 (173, 369) | 12.48 (8.25, 19.14) | -0.66 (-0.76, -0.56) | <0.001 |
| Croatia | 1701 (1536, 1875) | 144.74 (130.65, 159.43) | 1428 (1241, 1618) | 111.38 (96.2, 126.67) | 0.28 (0.1, 0.46) | 0.003 |
| Cuba | 1339 (1254, 1420) | 85.5 (77.26, 93.96) | 2748 (2338, 3195) | 90.91 (75.64, 106.88) | -0.67 (-1.21, -0.13) | 0.016 |
| Cyprus | 82 (64, 101) | 62.9 (46.71, 83.55) | 159 (128, 192) | 50.78 (39.08, 63.84) | -2.53 (-2.66, -2.41) | <0.001 |
| Czechia | 3838 (3604, 4082) | 172.33 (158.19, 187.33) | 2357 (2054, 2645) | 79.73 (68.21, 92.36) | -0.25 (-0.44, -0.06) | 0.012 |
| Democratic People's Republic of Korea | 2459 (1676, 3400) | 81.34 (53.58, 116.65) | 4526 (2929, 6590) | 79.2 (49.83, 117.48) | -0.09 (-0.16, -0.02) | 0.013 |
| Democratic Republic of the Congo | 847 (545, 1398) | 28.42 (17.72, 48.05) | 1815 (1100, 3234) | 26.17 (15.05, 48.2) | -0.26 (-0.39, -0.13) | <0.001 |
| Denmark | 1870 (1748, 1998) | 169.9 (157.45, 183.72) | 1279 (1184, 1382) | 82.07 (74.5, 90.27) | -2.3 (-2.62, -1.98) | <0.001 |
| Djibouti | 7 (5, 9) | 26.61 (16.88, 39.22) | 39 (24, 57) | 32.21 (19.23, 50.49) | 0.61 (0.56, 0.67) | <0.001 |
| Dominica | 4 (4, 5) | 50.44 (40.12, 62.86) | 8 (6, 10) | 51.83 (37.56, 69.55) | 0.09 (-0.09, 0.28) | 0.325 |
| Dominican Republic | 191 (160, 230) | 30.7 (23.42, 39.67) | 641 (433, 884) | 39.52 (25.43, 57.25) | 1.01 (0.07, 1.96) | 0.036 |
| Ecuador | 211 (195, 231) | 24.74 (22.28, 27.52) | 473 (358, 613) | 17.95 (13.39, 23.69) | -1.05 (-1.99, -0.1) | 0.031 |
| Egypt | 966 (795, 1164) | 18.74 (14.6, 23.79) | 5072 (3849, 6546) | 40.17 (29.45, 53.61) | 2.58 (1.97, 3.19) | <0.001 |
| El Salvador | 116 (100, 135) | 24.3 (19.79, 29.09) | 229 (175, 290) | 24.64 (18.34, 32.77) | 0.21 (-0.6, 1.04) | 0.608 |
| Equatorial Guinea | 15 (9, 25) | 41.82 (24.3, 74.15) | 38 (24, 56) | 42.94 (25.76, 65.16) | 0.14 (-0.25, 0.52) | 0.491 |
| Eritrea | 60 (45, 85) | 27.03 (19.09, 39.02) | 139 (103, 185) | 27.13 (18.42, 37.89) | -0.01 (-0.15, 0.14) | 0.916 |
| Estonia | 523 (491, 554) | 151.26 (137.94, 165.48) | 262 (222, 300) | 71.5 (59.43, 83.41) | -2.51 (-2.67, -2.34) | <0.001 |
| Eswatini | 29 (18, 48) | 58.67 (34.4, 97.8) | 69 (38, 103) | 68.57 (38.2, 106.15) | 0.5 (0.21, 0.8) | 0.001 |
| Ethiopia | 1425 (1012, 2040) | 39.86 (27.95, 58.31) | 1436 (1122, 1815) | 20.02 (15.53, 25.55) | -2.21 (-2.31, -2.11) | <0.001 |
| Fiji | 20 (16, 26) | 31.55 (23.44, 42.41) | 39 (28, 52) | 26.86 (18.73, 37.31) | -0.53 (-0.76, -0.3) | <0.001 |
| Finland | 965 (905, 1027) | 89.57 (81.57, 97.79) | 775 (693, 858) | 48.24 (42.21, 55.13) | -2.03 (-2.32, -1.74) | <0.001 |
| France | 13165 (12139, 14170) | 106.12 (96.72, 116.26) | 16392 (14803, 17963) | 93.45 (83.38, 103.5) | -0.37 (-0.55, -0.19) | <0.001 |
| Gabon | 50 (30, 71) | 50.26 (29.2, 74.8) | 99 (69, 132) | 49.46 (32.32, 69.18) | -0.03 (-0.28, 0.22) | 0.818 |
| Gambia | 6 (4, 8) | 9.9 (6.85, 14.14) | 16 (11, 22) | 10.04 (6.65, 14.49) | 0.07 (-0.86, 1.01) | 0.881 |
| Georgia | 1107 (986, 1235) | 97.68 (86.68, 109.91) | 821 (702, 961) | 85.55 (73.16, 99.81) | -0.67 (-1.4, 0.07) | 0.075 |
| Germany | 20959 (19645, 22268) | 109.51 (99.55, 119.24) | 20743 (19252, 22429) | 81.23 (73.38, 89.52) | -0.92 (-1.08, -0.77) | <0.001 |
| Ghana | 161 (124, 208) | 14.58 (9.94, 20.19) | 485 (345, 635) | 16.38 (10.97, 23.08) | 0.39 (0.25, 0.54) | <0.001 |
| Greece | 2823 (2656, 3016) | 115.58 (104.57, 127.33) | 3089 (2898, 3286) | 107.89 (98.06, 118.16) | -0.21 (-0.49, 0.07) | 0.143 |
| Greenland | 20 (17, 23) | 335.77 (276.54, 398.82) | 27 (22, 32) | 185.15 (141.89, 230.78) | -1.77 (-2, -1.54) | <0.001 |
| Grenada | 4 (4, 5) | 42.46 (35.16, 50.54) | 7 (6, 8) | 32.33 (26.1, 39.7) | -0.93 (-1.5, -0.36) | 0.001 |
| Guam | 15 (13, 17) | 104.3 (83.83, 128.61) | 29 (24, 34) | 80.28 (62.53, 100.02) | -0.92 (-1.36, -0.48) | <0.001 |
| Guatemala | 139 (128, 150) | 22.54 (19.81, 25.62) | 255 (211, 303) | 14.11 (11.37, 17.27) | -1.63 (-2.34, -0.92) | <0.001 |
| Guinea | 111 (86, 140) | 19.53 (13.73, 26.5) | 234 (159, 331) | 24.41 (15.64, 36.46) | 0.73 (0.6, 0.86) | <0.001 |
| Guinea-Bissau | 20 (14, 29) | 29.11 (18.28, 44.38) | 35 (25, 47) | 27.02 (18.12, 38.69) | -0.23 (-0.31, -0.15) | <0.001 |
| Guyana | 16 (13, 18) | 24.3 (19.86, 28.92) | 28 (21, 37) | 23.52 (16.96, 31.32) | 0.08 (-0.7, 0.87) | 0.842 |
| Haiti | 271 (171, 417) | 45.96 (28.33, 74.92) | 442 (266, 709) | 33.75 (19.74, 54.62) | -0.96 (-1, -0.92) | <0.001 |
| Honduras | 129 (105, 158) | 37.71 (29.26, 48.19) | 607 (422, 877) | 56.79 (37.93, 83.66) | 1.38 (1.04, 1.71) | <0.001 |
| Hungary | 4099 (3724, 4490) | 168.75 (152.46, 185.75) | 4464 (3838, 5091) | 161.96 (139.02, 185.11) | -0.1 (-0.42, 0.22) | 0.548 |
| Iceland | 41 (38, 44) | 95.89 (85.63, 106.69) | 54 (47, 60) | 62.45 (53.69, 71.72) | -1.39 (-1.71, -1.06) | <0.001 |
| India | 14677 (12736, 16994) | 17.3 (14.85, 20.25) | 41032 (33498, 47638) | 19.89 (16.13, 23.26) | 0.43 (-0.02, 0.88) | 0.059 |
| Indonesia | 9257 (6917, 10978) | 51.7 (38.43, 62.43) | 29421 (19854, 38397) | 63.6 (42.76, 83.61) | 0.66 (0.57, 0.76) | <0.001 |
| Iran (Islamic Republic of) | 1506 (1207, 1831) | 29.18 (23.23, 35.61) | 3494 (3187, 3832) | 25.62 (23.07, 28.43) | -0.48 (-0.6, -0.36) | <0.001 |
| Iraq | 620 (447, 848) | 49.11 (33.24, 70.96) | 2129 (1437, 2776) | 50.63 (33.39, 70.05) | 0.11 (-0.06, 0.29) | 0.197 |
| Ireland | 712 (663, 758) | 116.32 (104.78, 128.56) | 679 (614, 752) | 58.81 (51.02, 67.78) | -2.15 (-2.76, -1.55) | <0.001 |
| Israel | 530 (496, 565) | 71.75 (65.01, 78.57) | 840 (770, 910) | 48.45 (42.94, 54.27) | -1.23 (-1.5, -0.95) | <0.001 |
| Italy | 17167 (16709, 17628) | 121.53 (117.59, 125.28) | 10519 (10003, 10932) | 59.5 (56.31, 62.08) | -2.4 (-2.54, -2.25) | <0.001 |
| Jamaica | 142 (132, 154) | 55.19 (48.69, 62.48) | 238 (179, 317) | 47.66 (35, 64.65) | -0.5 (-1.15, 0.15) | 0.127 |
| Japan | 15850 (15370, 16198) | 56.32 (54.46, 57.96) | 14361 (13640, 14811) | 39.95 (37.98, 41.37) | -1.15 (-1.3, -1.01) | <0.001 |
| Jordan | 108 (83, 140) | 43.75 (31.13, 59.52) | 409 (303, 544) | 29.51 (20.68, 41.64) | -1.21 (-1.46, -0.95) | <0.001 |
| Kazakhstan | 3805 (3512, 4122) | 165.65 (148.73, 183.57) | 1863 (1588, 2178) | 55.43 (47.04, 64.72) | -3.4 (-3.89, -2.9) | <0.001 |
| Kenya | 95 (63, 137) | 6.77 (4.46, 9.86) | 435 (342, 554) | 10.56 (8.22, 13.54) | 1.41 (1.25, 1.58) | <0.001 |
| Kiribati | 3 (2, 3) | 44.02 (32.74, 57.82) | 7 (5, 9) | 48.31 (33.02, 68.5) | 0.32 (0.26, 0.38) | <0.001 |
| Kuwait | 46 (41, 52) | 44.75 (38.21, 51.93) | 96 (77, 118) | 17.97 (13.96, 23.11) | -3.12 (-5.24, -0.95) | 0.005 |
| Kyrgyzstan | 549 (495, 608) | 101.62 (89.29, 114.61) | 345 (282, 413) | 37.4 (30.31, 45.16) | -3.3 (-3.89, -2.71) | <0.001 |
| Lao People's Democratic Republic | 304 (201, 473) | 82.19 (50.72, 133.6) | 530 (384, 743) | 63.86 (42.45, 93.8) | -0.8 (-0.88, -0.72) | <0.001 |
| Latvia | 825 (765, 887) | 136.75 (124.16, 150.66) | 418 (352, 475) | 77.07 (63.35, 90.82) | -1.89 (-2.7, -1.08) | <0.001 |
| Lebanon | 307 (219, 395) | 75.75 (51.59, 104.84) | 511 (398, 651) | 59.62 (43.66, 79.2) | -0.71 (-0.96, -0.46) | <0.001 |
| Lesotho | 53 (38, 80) | 37.73 (24.67, 60.12) | 151 (103, 219) | 79.08 (50.26, 119.52) | 2.48 (2.26, 2.7) | <0.001 |
| Liberia | 33 (25, 44) | 17.23 (11.91, 24.69) | 57 (35, 91) | 15.97 (9.27, 26.49) | -0.32 (-0.51, -0.13) | 0.001 |
| Libya | 222 (162, 298) | 68.67 (47.26, 96.04) | 644 (449, 907) | 70.33 (46.64, 102.5) | 0 (-0.3, 0.3) | 0.983 |
| Lithuania | 967 (899, 1030) | 125.63 (113.42, 138.29) | 627 (533, 721) | 77.63 (63.83, 91.23) | -1.6 (-2.18, -1.01) | <0.001 |
| Luxembourg | 112 (103, 121) | 132.71 (118.88, 147.34) | 116 (103, 128) | 72.51 (63.42, 82.07) | -1.99 (-2.55, -1.43) | <0.001 |
| Madagascar | 181 (146, 220) | 20.02 (15.08, 26.56) | 363 (257, 481) | 16.96 (11.4, 23.48) | -0.53 (-0.67, -0.38) | <0.001 |
| Malawi | 49 (39, 63) | 7.1 (5.18, 9.87) | 106 (74, 146) | 8.33 (5.5, 12.15) | 0.54 (0.35, 0.72) | <0.001 |
| Malaysia | 789 (666, 976) | 51.57 (39.22, 67.37) | 2590 (2149, 3038) | 51.52 (39.18, 65.73) | 0.16 (-0.13, 0.45) | 0.284 |
| Maldives | 8 (5, 11) | 43.18 (27.7, 64.53) | 10 (7, 13) | 17.13 (11.87, 23.56) | -3.18 (-3.56, -2.8) | <0.001 |
| Mali | 102 (82, 128) | 14.05 (10.31, 18.91) | 228 (163, 312) | 14.56 (9.52, 21.11) | 0.16 (0, 0.31) | 0.05 |
| Malta | 59 (55, 64) | 84.91 (74.92, 95.2) | 67 (60, 75) | 53.8 (46.05, 62.05) | -1.61 (-2.06, -1.15) | <0.001 |
| Marshall Islands | 2 (1, 3) | 75.99 (46.12, 118.27) | 5 (3, 8) | 80.62 (47.35, 121.46) | 0.21 (0.06, 0.36) | 0.005 |
| Mauritania | 37 (27, 48) | 22.99 (15.65, 31.76) | 76 (53, 107) | 20.44 (13.31, 30.04) | -0.4 (-0.54, -0.25) | <0.001 |
| Mauritius | 56 (52, 60) | 43.07 (37.79, 48.75) | 97 (88, 105) | 28.77 (24.96, 32.53) | -1.31 (-2.19, -0.43) | 0.004 |
| Mexico | 2640 (2572, 2712) | 37.5 (36.19, 38.84) | 3845 (3354, 4341) | 18.01 (15.66, 20.47) | -2.5 (-2.74, -2.26) | <0.001 |
| Micronesia (Federated States of) | 7 (5, 10) | 84.14 (56.6, 122.9) | 14 (9, 19) | 90.06 (57.88, 132.26) | 0.21 (0.14, 0.29) | <0.001 |
| Monaco | 14 (11, 17) | 155.39 (118.25, 197.79) | 21 (17, 26) | 169.16 (134.1, 214.64) | 0.26 (0.09, 0.42) | 0.002 |
| Mongolia | 194 (150, 252) | 111.16 (85.6, 144.07) | 303 (236, 392) | 70.15 (54.18, 90.24) | -1.53 (-1.86, -1.21) | <0.001 |
| Montenegro | 169 (143, 197) | 152.67 (125.24, 183.01) | 251 (199, 307) | 150.64 (118.59, 188.88) | 0.04 (-0.41, 0.5) | 0.851 |
| Morocco | 1004 (804, 1261) | 42.15 (30.13, 57.25) | 2773 (1959, 3564) | 44.67 (29.43, 62.26) | 0.21 (0.09, 0.32) | <0.001 |
| Mozambique | 158 (129, 192) | 14.82 (11.06, 19.36) | 385 (279, 511) | 19.47 (13.26, 27.33) | 0.89 (0.76, 1.02) | <0.001 |
| Myanmar | 3160 (2133, 4698) | 75.23 (48.88, 114.66) | 4836 (3455, 6540) | 54.24 (37.02, 76.31) | -1.05 (-1.13, -0.98) | <0.001 |
| Namibia | 21 (17, 26) | 17.69 (12.88, 23.5) | 47 (34, 62) | 19.33 (13.21, 27.15) | 0.3 (0.09, 0.52) | 0.006 |
| Nauru | 1 (1, 1) | 123.41 (65.37, 186.94) | 1 (1, 2) | 120.01 (64.91, 171.84) | -0.1 (-0.17, -0.02) | 0.01 |
| Nepal | 329 (219, 475) | 19.18 (11.95, 29.22) | 689 (501, 947) | 16.98 (11.19, 24.85) | -0.38 (-0.53, -0.23) | <0.001 |
| Netherlands | 4156 (3932, 4382) | 140.03 (129.68, 150.52) | 4021 (3698, 4321) | 81.54 (74.29, 88.67) | -1.84 (-2.04, -1.63) | <0.001 |
| New Zealand | 666 (624, 711) | 110.75 (100.51, 121.09) | 738 (680, 798) | 59.09 (52.72, 65.55) | -2 (-2.33, -1.66) | <0.001 |
| Nicaragua | 41 (35, 48) | 16.29 (12.98, 20.21) | 113 (90, 142) | 13.92 (10.33, 18.4) | -0.54 (-0.85, -0.24) | 0.001 |
| Niger | 67 (48, 103) | 13.53 (8.8, 21.31) | 180 (119, 305) | 12.32 (7.56, 21.16) | -0.26 (-0.48, -0.05) | 0.017 |
| Nigeria | 319 (218, 436) | 4.21 (2.83, 5.79) | 663 (508, 864) | 4.1 (3.12, 5.31) | -0.09 (-0.18, 0.01) | 0.081 |
| Niue | 0 (0, 0) | 67.54 (47.59, 91.37) | 0 (0, 0) | 76.39 (51.5, 107.2) | 0.39 (0.25, 0.52) | <0.001 |
| North Macedonia | 367 (305, 435) | 107.05 (86.77, 130.39) | 624 (466, 786) | 107.55 (80.57, 136.97) | -0.05 (-0.33, 0.23) | 0.745 |
| Northern Mariana Islands | 4 (3, 5) | 131.42 (98.2, 176.16) | 10 (9, 12) | 98.73 (77.3, 123.94) | -0.9 (-0.99, -0.81) | <0.001 |
| Norway | 707 (682, 730) | 79.56 (76.02, 83.03) | 752 (710, 788) | 54.71 (51.14, 57.99) | -1.17 (-1.32, -1.01) | <0.001 |
| Oman | 22 (15, 31) | 20.74 (13.24, 30.9) | 45 (31, 64) | 12.76 (8.63, 18.43) | -1.55 (-2.18, -0.92) | <0.001 |
| Pakistan | 3693 (3000, 4421) | 39.88 (30.83, 50.47) | 9333 (6854, 12558) | 43.53 (31.09, 59.59) | 0.28 (0.17, 0.39) | <0.001 |
| Palau | 2 (2, 3) | 131.2 (94.04, 179.94) | 5 (4, 7) | 112.92 (79.21, 152.08) | -0.5 (-0.67, -0.33) | <0.001 |
| Palestine | 84 (59, 114) | 56.59 (37.58, 82.45) | 236 (189, 287) | 50.59 (38.35, 65.08) | -0.32 (-0.66, 0.01) | 0.054 |
| Panama | 93 (86, 102) | 39.66 (34.71, 45.08) | 139 (106, 172) | 19.88 (14.9, 25.23) | -2.36 (-3.49, -1.22) | <0.001 |
| Papua New Guinea | 160 (95, 273) | 46.86 (26.34, 80.98) | 466 (299, 738) | 50.51 (31.3, 82.63) | 0.28 (0.21, 0.34) | <0.001 |
| Paraguay | 109 (86, 134) | 30.26 (23.46, 38.67) | 449 (327, 608) | 45.39 (31.96, 63.22) | 1.38 (1.15, 1.61) | <0.001 |
| Peru | 793 (648, 953) | 40.41 (31.2, 51.08) | 1429 (1015, 1854) | 26.59 (18.12, 36.71) | -1.07 (-2.25, 0.13) | 0.08 |
| Philippines | 2614 (2306, 2977) | 51.19 (44.88, 59.09) | 7494 (6063, 9063) | 50.16 (40.34, 61.16) | -0.06 (-0.22, 0.08) | 0.391 |
| Poland | 12686 (12400, 13001) | 169.84 (164.81, 174.91) | 14637 (13317, 15888) | 130.48 (118.3, 141.91) | -1.01 (-1.25, -0.77) | <0.001 |
| Portugal | 1409 (1292, 1533) | 62.12 (56.04, 68.73) | 1936 (1743, 2130) | 63.27 (56.49, 70.18) | 0.09 (-0.62, 0.8) | 0.81 |
| Puerto Rico | 258 (239, 279) | 46.03 (41.15, 51.36) | 220 (180, 264) | 23.91 (19.07, 29.38) | -2.32 (-2.76, -1.87) | <0.001 |
| Qatar | 13 (10, 17) | 66.99 (46.93, 91.35) | 56 (40, 80) | 29.02 (19.08, 42.99) | -2.71 (-3.56, -1.86) | <0.001 |
| Republic of Korea | 4427 (3829, 5066) | 82.25 (66.65, 99.3) | 6580 (5560, 7746) | 41.01 (32.31, 50.97) | -2.29 (-2.57, -2.01) | <0.001 |
| Republic of Moldova | 918 (840, 997) | 112.91 (100.62, 124.9) | 702 (616, 796) | 70.13 (59.94, 81.84) | -1.47 (-2.35, -0.59) | 0.001 |
| Romania | 5099 (4710, 5502) | 100.07 (88.92, 111.35) | 6167 (5381, 7002) | 116.77 (99.02, 136.62) | 0.52 (0.06, 0.99) | 0.027 |
| Russian Federation | 45007 (44282, 45748) | 139.02 (136.08, 141.81) | 30876 (27663, 33774) | 76.33 (68.28, 83.72) | -1.66 (-2.52, -0.8) | <0.001 |
| Rwanda | 159 (114, 199) | 30.6 (20.93, 41.62) | 268 (176, 382) | 22.99 (14.47, 34.59) | -0.92 (-1.09, -0.74) | <0.001 |
| Saint Kitts and Nevis | 2 (2, 2) | 32.49 (28.43, 36.96) | 4 (3, 5) | 27.5 (21.68, 33.89) | -0.56 (-1.19, 0.09) | 0.091 |
| Saint Lucia | 6 (5, 6) | 40.15 (34.78, 45.84) | 12 (9, 15) | 29.17 (22.66, 36.7) | -1 (-1.57, -0.42) | 0.001 |
| Saint Vincent and the Grenadines | 3 (3, 4) | 29.98 (25.98, 34.39) | 6 (5, 7) | 25.47 (21.33, 30.25) | -0.52 (-1.1, 0.06) | 0.078 |
| Samoa | 4 (3, 6) | 28.04 (20.4, 38.65) | 7 (5, 9) | 27 (18.11, 37.76) | -0.12 (-0.22, -0.03) | 0.015 |
| San Marino | 5 (4, 6) | 100.31 (76.34, 125.9) | 4 (3, 7) | 46.95 (26.13, 72.83) | -2.67 (-2.97, -2.37) | <0.001 |
| Sao Tome and Principe | 4 (3, 5) | 37.4 (27.44, 49.78) | 9 (7, 12) | 46.38 (31.75, 65.58) | 0.69 (0.37, 1.01) | <0.001 |
| Saudi Arabia | 189 (135, 256) | 19.93 (13.59, 28.49) | 668 (512, 867) | 18.2 (13.06, 25.06) | -0.28 (-0.37, -0.19) | <0.001 |
| Senegal | 124 (96, 156) | 22.51 (16.04, 30.54) | 312 (223, 426) | 23.24 (15.72, 33.12) | 0.1 (-0.11, 0.3) | 0.346 |
| Serbia | 3225 (2559, 4024) | 143.39 (113.15, 180.72) | 3745 (2934, 4637) | 150.7 (117.15, 187.67) | 0.4 (-0.36, 1.18) | 0.304 |
| Seychelles | 5 (4, 7) | 60.84 (45.44, 78.72) | 8 (6, 10) | 36.96 (28.02, 48.2) | -1.49 (-2.08, -0.89) | <0.001 |
| Sierra Leone | 62 (45, 87) | 19.06 (12.8, 27.58) | 118 (79, 170) | 18.81 (12.05, 28.02) | 0 (-0.14, 0.13) | 0.964 |
| Singapore | 344 (318, 373) | 92.42 (83.57, 102.07) | 458 (412, 517) | 30.76 (26.9, 35.31) | -3.47 (-4.13, -2.8) | <0.001 |
| Slovakia | 1554 (1355, 1777) | 154.66 (125.66, 186.47) | 1307 (1015, 1600) | 86.46 (65.43, 108.84) | -1.94 (-2.42, -1.47) | <0.001 |
| Slovenia | 558 (520, 598) | 133.62 (121.02, 146.19) | 530 (446, 620) | 82.99 (69.38, 97.82) | -1.64 (-2.23, -1.04) | <0.001 |
| Solomon Islands | 16 (10, 25) | 62.72 (36.79, 97.37) | 39 (28, 55) | 63.62 (44.17, 91.49) | 0.06 (-0.16, 0.27) | 0.606 |
| Somalia | 88 (56, 150) | 20.57 (12.36, 36.93) | 194 (121, 361) | 17.69 (10.12, 34.08) | -0.48 (-0.54, -0.41) | <0.001 |
| South Africa | 2026 (1729, 2591) | 58.46 (48.85, 74.78) | 5274 (4712, 5922) | 64.37 (56.47, 73.74) | 0.18 (-0.65, 1.01) | 0.678 |
| South Sudan | 116 (73, 175) | 27.03 (15.97, 42.43) | 177 (122, 251) | 25.14 (16.46, 37.08) | -0.22 (-0.3, -0.14) | <0.001 |
| Spain | 8256 (7789, 8701) | 96.04 (87.36, 105.39) | 9858 (9113, 10565) | 78.42 (69.56, 87.87) | -0.72 (-0.97, -0.46) | <0.001 |
| Sri Lanka | 496 (420, 587) | 26.65 (20.41, 34.47) | 1150 (700, 1627) | 24.25 (14.32, 35.8) | -0.2 (-0.63, 0.23) | 0.353 |
| Sudan | 354 (238, 578) | 23.57 (14.62, 40.52) | 809 (533, 1200) | 24.34 (14.98, 37.2) | 0.1 (0.04, 0.16) | 0.001 |
| Suriname | 19 (17, 22) | 43.58 (34.93, 53.87) | 46 (35, 60) | 41.41 (29.66, 56.23) | -0.1 (-0.89, 0.7) | 0.809 |
| Sweden | 1196 (1114, 1278) | 62.72 (57.69, 68.2) | 944 (830, 1075) | 36.17 (31.27, 41.53) | -1.88 (-2.21, -1.54) | <0.001 |
| Switzerland | 1328 (1237, 1416) | 92.08 (82.94, 100.96) | 1261 (1163, 1354) | 52.57 (47.18, 58.3) | -1.82 (-2.09, -1.56) | <0.001 |
| Syrian Arab Republic | 354 (261, 464) | 37.6 (25.81, 51.65) | 894 (604, 1264) | 34.9 (22.31, 51.59) | -0.35 (-0.65, -0.05) | 0.021 |
| Taiwan (Province of China) | 2215 (2102, 2324) | 75.15 (68.34, 82.14) | 4499 (4076, 4841) | 62.79 (54.93, 70.69) | -0.64 (-0.93, -0.34) | <0.001 |
| Tajikistan | 307 (250, 373) | 62.02 (49.48, 76.14) | 302 (205, 434) | 25.93 (17.56, 37.16) | -2.89 (-3.31, -2.48) | <0.001 |
| Thailand | 6136 (4985, 7543) | 96.99 (74.25, 124.13) | 11196 (8336, 14447) | 59.48 (42.43, 81.19) | -1.62 (-1.88, -1.36) | <0.001 |
| Timor-Leste | 21 (14, 30) | 40.87 (26.54, 61.77) | 62 (46, 82) | 43 (29.5, 60.21) | 0.16 (-0.02, 0.34) | 0.082 |
| Togo | 45 (34, 59) | 21.11 (14.66, 29.4) | 169 (107, 239) | 24.34 (14.5, 36.99) | 0.5 (0.2, 0.81) | 0.001 |
| Tokelau | 0 (0, 0) | 61.84 (42.97, 84.85) | 0 (0, 0) | 62.88 (43.13, 86.57) | 0.04 (-0.16, 0.23) | 0.713 |
| Tonga | 7 (6, 9) | 75.98 (55.05, 103.1) | 10 (8, 13) | 77.38 (54.07, 105.84) | 0.09 (-0.26, 0.44) | 0.604 |
| Trinidad and Tobago | 47 (43, 50) | 34.92 (30.6, 39.98) | 102 (75, 133) | 30.66 (22.33, 40.71) | -0.41 (-0.54, -0.29) | <0.001 |
| Tunisia | 525 (412, 645) | 58.83 (42.87, 76.86) | 1321 (848, 1915) | 56.11 (34.47, 83.33) | -0.15 (-0.28, -0.03) | 0.018 |
| Turkey | 10631 (8221, 13402) | 167.4 (119.75, 224) | 17457 (13422, 21936) | 107.57 (79.95, 140.17) | -1.44 (-1.62, -1.26) | <0.001 |
| Turkmenistan | 222 (199, 244) | 63.65 (55.4, 71.86) | 238 (179, 324) | 30.2 (22.67, 40.8) | -2.34 (-3.35, -1.33) | <0.001 |
| Tuvalu | 1 (1, 1) | 68.39 (46.14, 106.07) | 1 (1, 2) | 69.44 (48.61, 99.92) | 0.04 (0, 0.09) | 0.076 |
| Uganda | 210 (159, 272) | 19.1 (13.47, 26.24) | 513 (358, 707) | 19.98 (13.11, 28.9) | 0.14 (-0.1, 0.37) | 0.25 |
| Ukraine | 17865 (16609, 19127) | 143.04 (129.87, 157.56) | 8085 (5458, 11337) | 65.6 (43.79, 94.24) | -2.55 (-3.27, -1.82) | <0.001 |
| United Arab Emirates | 43 (29, 60) | 62.89 (40.39, 93.43) | 222 (160, 349) | 22.08 (14.8, 34.75) | -3.35 (-3.74, -2.96) | <0.001 |
| United Kingdom | 17954 (17682, 18204) | 139.87 (137.34, 142.23) | 10529 (10218, 10805) | 61.44 (59.33, 63.17) | -2.64 (-2.82, -2.46) | <0.001 |
| United Republic of Tanzania | 415 (324, 568) | 21.72 (15.17, 30.98) | 942 (664, 1297) | 21.4 (13.78, 32.17) | -0.07 (-0.21, 0.08) | 0.384 |
| United States of America | 74997 (73110, 76740) | 163.85 (158.86, 168.28) | 63746 (61196, 65954) | 71 (67.75, 73.57) | -2.67 (-2.86, -2.48) | <0.001 |
| United States Virgin Islands | 8 (6, 9) | 52.51 (39.23, 67.95) | 9 (7, 12) | 32.61 (22.92, 46.6) | -1.6 (-2.43, -0.77) | <0.001 |
| Uruguay | 847 (784, 907) | 138.86 (125.29, 153.76) | 805 (745, 867) | 105.08 (94.42, 115.97) | -0.98 (-1.13, -0.82) | <0.001 |
| Uzbekistan | 1291 (1169, 1412) | 64.14 (57.63, 70.93) | 1257 (1006, 1523) | 23.99 (19.26, 29.11) | -3.25 (-4.01, -2.5) | <0.001 |
| Vanuatu | 6 (4, 10) | 51.45 (29.84, 90.7) | 16 (11, 26) | 50.69 (31.7, 83.29) | -0.04 (-0.33, 0.26) | 0.819 |
| Venezuela (Bolivarian Republic of) | 941 (883, 1003) | 59.23 (52.68, 66.82) | 2651 (1969, 3470) | 50.53 (36.24, 68.05) | -0.68 (-1.13, -0.23) | 0.003 |
| Viet Nam | 4930 (3581, 6351) | 70.48 (50.12, 93.41) | 13550 (9424, 17822) | 72.62 (50.21, 97.53) | 0.11 (0, 0.22) | 0.059 |
| Yemen | 271 (159, 473) | 30.69 (17.42, 54.69) | 691 (420, 1101) | 28.48 (16.67, 46.59) | -0.23 (-0.39, -0.07) | 0.004 |
| Zambia | 127 (101, 154) | 25.5 (18.69, 33.77) | 403 (277, 724) | 33.56 (21.61, 60.99) | 0.89 (0.77, 1.01) | <0.001 |
| Zimbabwe | 264 (210, 327) | 36.87 (27.13, 48.57) | 511 (392, 687) | 40.75 (28.84, 56.48) | 0.35 (0.17, 0.53) | <0.001 |
| LOLC |  |  |  |  |  |  |
| Afghanistan | 196 (113, 379) | 63.79 (34.31, 125.21) | 310 (207, 494) | 77.72 (48.07, 126.79) | 0.63 (0.55, 0.71) | <0.001 |
| Albania | 213 (167, 271) | 213.54 (164.11, 276.31) | 556 (384, 774) | 204.58 (138.67, 285.72) | -0.16 (-0.52, 0.19) | 0.356 |
| Algeria | 391 (288, 522) | 76.11 (54.08, 106.03) | 945 (674, 1318) | 60.2 (41.8, 86.36) | -0.82 (-1.07, -0.56) | <0.001 |
| American Samoa | 2 (2, 3) | 258.19 (194.9, 333.83) | 5 (4, 6) | 237.79 (178.78, 311.4) | -0.18 (-0.31, -0.05) | 0.006 |
| Andorra | 11 (8, 16) | 348.58 (237.48, 499.48) | 19 (14, 26) | 205.67 (137.97, 295.75) | -1.82 (-2.23, -1.41) | <0.001 |
| Angola | 109 (75, 153) | 82.39 (52.22, 122.92) | 355 (266, 454) | 88.8 (61.96, 122.89) | 0.26 (0.1, 0.43) | 0.002 |
| Antigua and Barbuda | 3 (3, 3) | 81.93 (71.33, 93.37) | 5 (4, 5) | 80.97 (70.67, 92.9) | -0.14 (-1.01, 0.74) | 0.756 |
| Argentina | 3588 (3284, 3910) | 193.68 (172.4, 217.1) | 5798 (5212, 6395) | 162.08 (140.75, 183.14) | -0.52 (-0.67, -0.36) | <0.001 |
| Armenia | 190 (173, 206) | 161.89 (147.02, 176.51) | 505 (450, 566) | 210.6 (186.38, 238.2) | 0.82 (0.07, 1.58) | 0.032 |
| Australia | 3108 (2885, 3299) | 254.98 (227.38, 282.94) | 6653 (5731, 7493) | 210.51 (176.02, 244.39) | -0.62 (-0.76, -0.48) | <0.001 |
| Austria | 1646 (1521, 1745) | 209.27 (187.25, 230.67) | 2360 (2081, 2613) | 191.02 (165.34, 214.82) | -0.23 (-0.64, 0.18) | 0.269 |
| Azerbaijan | 221 (194, 251) | 101.29 (85.28, 120.65) | 360 (259, 480) | 93.48 (63.36, 128.89) | -0.23 (-0.61, 0.16) | 0.246 |
| Bahamas | 8 (7, 9) | 102.14 (88.55, 116.44) | 20 (17, 25) | 105.54 (85.18, 129.58) | 0.21 (-0.67, 1.1) | 0.636 |
| Bahrain | 21 (17, 24) | 372.31 (286.13, 477.83) | 51 (41, 65) | 229.96 (167.47, 310.88) | -1.62 (-2.23, -1) | <0.001 |
| Bangladesh | 1020 (711, 1496) | 44.65 (29.49, 68.16) | 2899 (2127, 3891) | 40.33 (27.89, 59.55) | -0.19 (-0.72, 0.35) | 0.496 |
| Barbados | 18 (17, 19) | 84.46 (74.67, 94.87) | 26 (21, 31) | 80.53 (63.85, 98.94) | -0.08 (-0.76, 0.6) | 0.816 |
| Belarus | 1039 (969, 1124) | 152.87 (135.83, 171.27) | 1182 (953, 1409) | 126.37 (100.37, 154.65) | -0.53 (-1.46, 0.4) | 0.262 |
| Belgium | 3716 (3371, 4048) | 383.02 (339.89, 428.43) | 4256 (3638, 4816) | 260.49 (219.82, 299.47) | -1.31 (-1.71, -0.92) | <0.001 |
| Belize | 4 (4, 5) | 82.8 (71.6, 94.36) | 12 (11, 14) | 88 (74.24, 103.24) | 0.14 (-0.59, 0.88) | 0.7 |
| Benin | 55 (44, 70) | 52.94 (37.48, 72.86) | 123 (95, 162) | 55.19 (38.24, 77.22) | 0.11 (-0.07, 0.3) | 0.238 |
| Bermuda | 12 (11, 13) | 341.62 (290.75, 397.34) | 19 (16, 23) | 200.69 (160.3, 253.98) | -1.81 (-2.16, -1.46) | <0.001 |
| Bhutan | 3 (2, 4) | 29.82 (16.58, 47.26) | 14 (10, 20) | 43.71 (27.32, 64.77) | 1.24 (1.13, 1.36) | <0.001 |
| Bolivia (Plurinational State of) | 200 (146, 263) | 133.09 (90.23, 184.38) | 608 (425, 869) | 134.38 (87.93, 199.48) | 0.04 (-0.03, 0.11) | 0.287 |
| Bosnia and Herzegovina | 354 (302, 415) | 208.08 (172.95, 250.85) | 976 (736, 1295) | 251.95 (187.82, 336.27) | 0.69 (0.49, 0.88) | <0.001 |
| Botswana | 27 (20, 35) | 115.52 (80.04, 161.65) | 71 (52, 91) | 121.38 (84.38, 167.12) | 0.17 (-0.06, 0.41) | 0.153 |
| Brazil | 5133 (4800, 5378) | 121.77 (112.18, 129.51) | 17651 (15721, 18885) | 125.3 (111, 135.66) | 0.12 (-0.08, 0.32) | 0.231 |
| Brunei Darussalam | 17 (13, 22) | 370.23 (263.65, 510.52) | 34 (26, 41) | 263.85 (194.64, 346.03) | -1.15 (-1.86, -0.43) | 0.002 |
| Bulgaria | 887 (791, 994) | 139.92 (122.76, 158.84) | 1901 (1592, 2218) | 185.18 (153.87, 217.71) | 1 (0.68, 1.31) | <0.001 |
| Burkina Faso | 84 (61, 124) | 42.52 (28.64, 65.71) | 215 (157, 315) | 51.37 (34.84, 78.67) | 0.65 (0.47, 0.84) | <0.001 |
| Burundi | 58 (44, 78) | 49.39 (34.91, 70.4) | 76 (53, 108) | 42.77 (27.68, 65.32) | -0.47 (-0.62, -0.32) | <0.001 |
| Cabo Verde | 15 (13, 19) | 100.73 (75.82, 130.25) | 37 (28, 46) | 174.96 (121.71, 234.85) | 1.83 (1.11, 2.55) | <0.001 |
| Cambodia | 327 (233, 439) | 173.07 (113.4, 252.06) | 1114 (802, 1467) | 206.07 (137.4, 287.25) | 0.59 (0.51, 0.68) | <0.001 |
| Cameroon | 112 (91, 135) | 60.93 (44.2, 81.07) | 348 (261, 458) | 70.07 (47.96, 99.74) | 0.48 (0.34, 0.6) | <0.001 |
| Canada | 7035 (6539, 7461) | 348.15 (314.28, 380.27) | 14434 (12752, 15959) | 291.21 (249.49, 331) | -0.51 (-0.66, -0.36) | <0.001 |
| Central African Republic | 28 (18, 47) | 69.95 (43.7, 124.82) | 40 (26, 71) | 61.55 (37.87, 108.64) | -0.42 (-0.51, -0.33) | <0.001 |
| Chad | 59 (41, 86) | 38.54 (25.26, 58.74) | 145 (101, 213) | 60.79 (39.76, 91.96) | 1.48 (1.33, 1.63) | <0.001 |
| Chile | 746 (686, 806) | 137.72 (122.54, 153.1) | 2146 (1875, 2384) | 136.21 (116.9, 154.29) | -0.12 (-0.35, 0.12) | 0.344 |
| China | 95974 (83952, 107987) | 269.53 (231.92, 307.73) | 428031 (348046, 510340) | 367.48 (296.58, 442.89) | 1.03 (0.77, 1.3) | <0.001 |
| Colombia | 1107 (1021, 1181) | 132.61 (117.35, 149.02) | 3391 (2829, 4027) | 106.01 (84.44, 129.25) | -0.87 (-1.29, -0.45) | <0.001 |
| Comoros | 4 (3, 6) | 50.21 (33.95, 70.95) | 13 (8, 19) | 58.4 (36.12, 87.52) | 0.47 (0.34, 0.59) | <0.001 |
| Congo | 45 (30, 60) | 101.52 (66.11, 143.16) | 95 (70, 126) | 100.46 (69.1, 141.74) | -0.03 (-0.09, 0.04) | 0.395 |
| Cook Islands | 2 (2, 2) | 320.76 (236.92, 431.34) | 4 (3, 5) | 258.64 (186.66, 343.27) | -0.69 (-0.8, -0.58) | <0.001 |
| Costa Rica | 100 (91, 109) | 105.13 (91.85, 119.17) | 227 (194, 260) | 71.17 (57.92, 85.07) | -1.2 (-2.7, 0.32) | 0.121 |
| Côte d'Ivoire | 44 (36, 54) | 32.04 (23.51, 42.87) | 135 (101, 182) | 32.16 (22.15, 45.54) | -0.38 (-0.78, 0.01) | 0.059 |
| Croatia | 780 (689, 866) | 271.12 (239.6, 304.67) | 1505 (1315, 1696) | 244.97 (208.75, 284.4) | -0.26 (-0.64, 0.12) | 0.185 |
| Cuba | 1820 (1703, 1947) | 291.18 (258.52, 326.03) | 3407 (2947, 3883) | 269.18 (226.3, 314.68) | -0.56 (-0.83, -0.28) | <0.001 |
| Cyprus | 95 (74, 117) | 221.53 (158.55, 304.41) | 246 (196, 303) | 184.37 (136.83, 240.64) | -0.22 (-0.57, 0.14) | 0.227 |
| Czechia | 2104 (1956, 2272) | 262.48 (237.48, 289.95) | 3689 (3239, 4166) | 238.85 (202.69, 275.79) | 0.02 (-0.09, 0.13) | 0.751 |
| Democratic People's Republic of Korea | 1156 (858, 1467) | 177.09 (121.85, 243.22) | 2883 (1850, 4197) | 163.88 (99.92, 250.75) | -0.26 (-0.32, -0.19) | <0.001 |
| Democratic Republic of the Congo | 368 (237, 620) | 64.73 (39.12, 115.64) | 709 (440, 1237) | 55.92 (32.59, 101.88) | -0.46 (-0.57, -0.34) | <0.001 |
| Denmark | 1986 (1830, 2134) | 357.82 (322.29, 394.88) | 2941 (2506, 3348) | 337 (283.3, 388.69) | -0.26 (-0.5, -0.02) | 0.034 |
| Djibouti | 2 (2, 3) | 56.93 (38.08, 81.21) | 17 (11, 24) | 80.38 (49.9, 118.22) | 1.11 (1.02, 1.21) | <0.001 |
| Dominica | 6 (5, 7) | 150.08 (115.33, 190.41) | 7 (6, 9) | 153.87 (114.27, 201.02) | 0.08 (-0.08, 0.25) | 0.32 |
| Dominican Republic | 160 (135, 189) | 91.45 (68.54, 119.3) | 608 (417, 825) | 107.96 (68.77, 157.86) | 0.5 (-0.34, 1.34) | 0.243 |
| Ecuador | 216 (199, 234) | 80.63 (71.1, 90.53) | 762 (620, 929) | 81.72 (64.02, 102.82) | 0.17 (-1, 1.35) | 0.782 |
| Egypt | 336 (279, 400) | 35.21 (26.8, 45.56) | 2599 (2047, 3249) | 119.86 (89.62, 158.86) | 3.98 (3.51, 4.46) | <0.001 |
| El Salvador | 109 (95, 127) | 68.92 (55.08, 84.19) | 301 (241, 383) | 76.98 (57.84, 102.22) | 0.34 (-0.53, 1.21) | 0.447 |
| Equatorial Guinea | 6 (4, 9) | 72.47 (45.99, 119.8) | 21 (14, 30) | 112.33 (70.53, 167.07) | 1.46 (1.35, 1.56) | <0.001 |
| Eritrea | 12 (9, 16) | 39.39 (28.04, 55.1) | 51 (39, 65) | 56.19 (40.58, 74.72) | 1.17 (1.06, 1.28) | <0.001 |
| Estonia | 246 (231, 263) | 212.88 (191.22, 235.42) | 379 (319, 432) | 199.21 (165.36, 232.28) | -0.41 (-0.85, 0.04) | 0.078 |
| Eswatini | 13 (8, 21) | 111.08 (65.34, 181.59) | 30 (17, 43) | 129.56 (72.71, 196.71) | 0.53 (0.4, 0.67) | <0.001 |
| Ethiopia | 574 (440, 752) | 72.39 (54.06, 98.58) | 1122 (898, 1371) | 59.69 (45.88, 75.43) | -0.59 (-0.68, -0.49) | <0.001 |
| Fiji | 12 (10, 14) | 88.28 (64.73, 117.55) | 27 (20, 34) | 85.1 (59.87, 114.16) | -0.16 (-0.51, 0.2) | 0.389 |
| Finland | 1006 (925, 1073) | 218.72 (195.57, 240.94) | 1801 (1560, 1997) | 190.05 (161.69, 216.56) | -0.49 (-0.79, -0.19) | 0.001 |
| France | 10687 (9909, 11432) | 208.07 (185.95, 231.62) | 20714 (18120, 23077) | 202.19 (171.95, 229.62) | -0.07 (-0.24, 0.1) | 0.415 |
| Gabon | 31 (20, 46) | 109.58 (67.32, 168.76) | 48 (35, 64) | 118.96 (78.58, 166.6) | 0.29 (0.2, 0.38) | <0.001 |
| Gambia | 3 (3, 4) | 21.36 (14.88, 29.55) | 11 (8, 16) | 24.58 (15.97, 35.71) | 0.44 (0.07, 0.82) | 0.02 |
| Georgia | 399 (359, 437) | 121.3 (106.03, 136.52) | 593 (522, 665) | 166.3 (142.5, 192.24) | 0.86 (-0.13, 1.85) | 0.088 |
| Germany | 18150 (16723, 19397) | 219.61 (196.71, 243.38) | 29445 (25635, 32676) | 215.81 (186.52, 243.09) | -0.08 (-0.29, 0.13) | 0.452 |
| Ghana | 117 (93, 146) | 47.76 (33.09, 66.95) | 391 (295, 492) | 58.16 (39.43, 81.27) | 0.65 (0.58, 0.72) | <0.001 |
| Greece | 3236 (2949, 3492) | 338.73 (298.93, 376.15) | 5243 (4633, 5741) | 299.5 (260.79, 333.74) | -0.4 (-0.57, -0.23) | <0.001 |
| Greenland | 10 (8, 12) | 779.96 (616.8, 959.47) | 16 (12, 20) | 526.53 (390.83, 687.98) | -1.13 (-1.59, -0.67) | <0.001 |
| Grenada | 5 (4, 5) | 92.04 (78.01, 106.82) | 6 (5, 6) | 93.13 (77.13, 110.32) | 0.21 (-0.92, 1.36) | 0.72 |
| Guam | 9 (7, 10) | 331.85 (267.71, 401.89) | 20 (16, 24) | 166.63 (127, 209.3) | -2.22 (-3.14, -1.29) | <0.001 |
| Guatemala | 114 (106, 123) | 84.09 (74.46, 93.99) | 312 (266, 360) | 53.58 (44.78, 63.39) | -1.61 (-2.3, -0.91) | <0.001 |
| Guinea | 87 (66, 111) | 52.69 (36.23, 74.09) | 172 (118, 239) | 66.21 (41.76, 100.38) | 0.78 (0.65, 0.9) | <0.001 |
| Guinea-Bissau | 11 (8, 15) | 63.76 (43.33, 92.96) | 17 (13, 23) | 67.51 (48.01, 93.64) | 0.19 (0.11, 0.27) | <0.001 |
| Guyana | 10 (9, 11) | 57.4 (48.22, 67.38) | 15 (11, 18) | 50.43 (38.74, 63.82) | -0.44 (-0.87, -0.02) | 0.042 |
| Haiti | 132 (92, 202) | 99.07 (63.9, 161.89) | 250 (158, 400) | 89.57 (53.25, 150.73) | -0.31 (-0.38, -0.24) | <0.001 |
| Honduras | 98 (76, 124) | 100.88 (73.2, 137.54) | 543 (355, 781) | 174.57 (108.32, 264.17) | 1.85 (1.54, 2.16) | <0.001 |
| Hungary | 2280 (2059, 2492) | 267.1 (236.6, 299.05) | 3768 (3210, 4346) | 282.38 (239.64, 327.25) | 0.27 (-0.02, 0.55) | 0.065 |
| Iceland | 48 (43, 52) | 259.92 (227.65, 292.62) | 93 (78, 105) | 238.72 (195.94, 278.54) | -0.38 (-0.99, 0.24) | 0.227 |
| India | 5535 (4672, 6487) | 30.38 (25.34, 35.93) | 24881 (20489, 28569) | 41.96 (34.36, 48.57) | 1.09 (0.55, 1.64) | <0.001 |
| Indonesia | 4504 (3329, 5598) | 118.62 (86.98, 148.53) | 17680 (11732, 22525) | 188.55 (123.08, 244.28) | 1.52 (1.45, 1.58) | <0.001 |
| Iran (Islamic Republic of) | 816 (647, 1002) | 89.19 (70, 111.88) | 3490 (3068, 3859) | 95.15 (81.7, 107.37) | 0.15 (0, 0.31) | 0.056 |
| Iraq | 439 (324, 575) | 110.53 (75.25, 153.77) | 1655 (1227, 2093) | 169.32 (116.38, 229.09) | 1.49 (1.13, 1.84) | <0.001 |
| Ireland | 890 (828, 949) | 326.89 (292.63, 360.32) | 1223 (1037, 1374) | 231.93 (193.84, 267.49) | -1.06 (-1.39, -0.73) | <0.001 |
| Israel | 583 (530, 634) | 192.43 (168.33, 216.12) | 1427 (1212, 1594) | 169.59 (141.6, 195.3) | -0.36 (-0.58, -0.13) | 0.002 |
| Italy | 14068 (13107, 14621) | 251.66 (234.37, 263.68) | 25496 (21999, 27640) | 232.46 (202.68, 251.81) | -0.24 (-0.29, -0.19) | <0.001 |
| Jamaica | 137 (124, 150) | 117.22 (100.36, 135.44) | 237 (183, 301) | 130.79 (97.81, 171.3) | 0.76 (0.3, 1.22) | 0.002 |
| Japan | 24798 (22876, 25802) | 249.82 (229.52, 261.97) | 76839 (64118, 83321) | 237.15 (202.13, 255.65) | -0.2 (-0.26, -0.15) | <0.001 |
| Jordan | 44 (34, 56) | 89.96 (65.22, 122.02) | 263 (204, 344) | 87.72 (62.35, 121.19) | -0.08 (-0.55, 0.39) | 0.748 |
| Kazakhstan | 1134 (1051, 1224) | 189.75 (171.83, 207.45) | 807 (688, 934) | 97.12 (82.73, 112.52) | -2.07 (-2.8, -1.33) | <0.001 |
| Kenya | 56 (36, 81) | 14.93 (9.56, 21.58) | 237 (186, 300) | 25.22 (19.65, 32.08) | 1.68 (1.47, 1.89) | <0.001 |
| Kiribati | 2 (1, 2) | 108.74 (75.59, 146.25) | 3 (2, 4) | 127.85 (87.17, 182.49) | 0.53 (0.41, 0.64) | <0.001 |
| Kuwait | 23 (20, 26) | 114.26 (94.66, 136.88) | 82 (66, 102) | 81.97 (62.44, 106.19) | -1.07 (-4.21, 2.18) | 0.514 |
| Kyrgyzstan | 178 (157, 196) | 126.78 (111.15, 142.55) | 139 (114, 170) | 73.05 (59.3, 89.41) | -1.88 (-2.56, -1.2) | <0.001 |
| Lao People's Democratic Republic | 153 (102, 243) | 166.38 (105.08, 269.19) | 340 (243, 465) | 177.91 (120.38, 255.27) | 0.22 (0.15, 0.3) | <0.001 |
| Latvia | 369 (335, 406) | 180.11 (160.83, 200.66) | 464 (392, 537) | 167.72 (138.59, 196.89) | -0.18 (-0.65, 0.28) | 0.445 |
| Lebanon | 194 (146, 254) | 202.31 (143.43, 277.48) | 851 (653, 1118) | 214.83 (155.5, 297.25) | 0.25 (-0.07, 0.56) | 0.122 |
| Lesotho | 34 (25, 51) | 77.72 (53.11, 118.19) | 66 (47, 89) | 130.79 (85.88, 188.92) | 1.73 (1.52, 1.94) | <0.001 |
| Liberia | 29 (22, 37) | 49.38 (34.94, 70.06) | 38 (25, 57) | 48.03 (28.8, 76.45) | -0.11 (-0.26, 0.03) | 0.125 |
| Libya | 173 (127, 227) | 199.95 (133.17, 285.39) | 457 (333, 623) | 208.41 (139.76, 303.38) | 0.15 (-0.21, 0.51) | 0.407 |
| Lithuania | 450 (416, 482) | 180.99 (163.49, 198.73) | 662 (568, 761) | 169.16 (140.79, 198.35) | -0.32 (-1.21, 0.57) | 0.478 |
| Luxembourg | 107 (99, 114) | 311.59 (278.47, 345.29) | 158 (139, 176) | 232.68 (199.09, 264.34) | -0.93 (-1.53, -0.32) | 0.003 |
| Madagascar | 92 (75, 112) | 42.2 (31.37, 56.39) | 142 (105, 180) | 39.71 (27.33, 54.36) | -0.17 (-0.29, -0.05) | 0.005 |
| Malawi | 26 (20, 31) | 16.2 (11.52, 22.09) | 57 (43, 75) | 17.9 (12.4, 25.38) | 0.34 (0.19, 0.49) | <0.001 |
| Malaysia | 562 (451, 714) | 129.64 (93.49, 178.67) | 2070 (1686, 2491) | 148.83 (109.83, 195.44) | 0.2 (-0.14, 0.55) | 0.241 |
| Maldives | 4 (3, 5) | 133.26 (87.03, 200.05) | 12 (10, 15) | 86.01 (62.22, 113.82) | -1.55 (-1.82, -1.27) | <0.001 |
| Mali | 56 (46, 69) | 34.39 (25.62, 45.31) | 146 (108, 190) | 39.6 (27.53, 55.53) | 0.47 (0.32, 0.62) | <0.001 |
| Malta | 55 (49, 61) | 217.68 (189.98, 247.19) | 111 (94, 128) | 155.78 (130.08, 182.44) | -1.2 (-1.49, -0.9) | <0.001 |
| Marshall Islands | 1 (1, 2) | 185.51 (124.78, 278.91) | 3 (2, 4) | 242.24 (157.94, 356) | 0.8 (0.63, 0.97) | <0.001 |
| Mauritania | 30 (22, 37) | 56.73 (40.28, 75.31) | 70 (51, 96) | 69.48 (46.9, 99.85) | 0.65 (0.52, 0.77) | <0.001 |
| Mauritius | 37 (34, 40) | 114.38 (101.48, 128.51) | 84 (76, 92) | 85.52 (74.56, 97.09) | -1.32 (-2.21, -0.43) | 0.004 |
| Mexico | 2791 (2668, 2878) | 138.73 (131.31, 145.2) | 4900 (4403, 5426) | 72.54 (64.15, 80.64) | -2.16 (-2.4, -1.91) | <0.001 |
| Micronesia (Federated States of) | 5 (4, 7) | 209.22 (147.7, 296.5) | 6 (4, 8) | 237.37 (160.34, 341.34) | 0.41 (0.36, 0.46) | <0.001 |
| Monaco | 19 (15, 25) | 382.14 (280.94, 504.67) | 40 (31, 49) | 544.6 (414.05, 696.72) | 1.14 (0.95, 1.33) | <0.001 |
| Mongolia | 113 (87, 147) | 215.28 (161.82, 282.65) | 149 (112, 197) | 174.04 (129.48, 230.45) | -0.74 (-1.19, -0.29) | 0.001 |
| Montenegro | 84 (67, 104) | 258.34 (201.72, 324.71) | 206 (156, 265) | 357.87 (265.8, 463.65) | 1.23 (0.88, 1.59) | <0.001 |
| Morocco | 691 (544, 893) | 96.45 (67.35, 135.81) | 1841 (1353, 2274) | 110.71 (73.79, 156.65) | 0.42 (0.31, 0.52) | <0.001 |
| Mozambique | 114 (92, 137) | 48.34 (35.17, 65.32) | 253 (183, 337) | 60.91 (40.87, 86.56) | 0.76 (0.6, 0.92) | <0.001 |
| Myanmar | 1565 (1149, 2214) | 158.18 (107.14, 236.42) | 3408 (2665, 4364) | 153.6 (108.85, 210.7) | -0.08 (-0.14, -0.03) | 0.005 |
| Namibia | 9 (7, 11) | 34.59 (24.62, 46.85) | 24 (19, 31) | 40.28 (29.05, 54.94) | 0.52 (0.4, 0.65) | <0.001 |
| Nauru | 1 (0, 1) | 333.69 (183.91, 491.6) | 1 (0, 1) | 307.76 (169.43, 418.6) | -0.24 (-0.31, -0.18) | <0.001 |
| Nepal | 137 (88, 205) | 37.33 (22.62, 58.65) | 513 (363, 718) | 44.79 (30.18, 65.8) | 0.6 (0.51, 0.69) | <0.001 |
| Netherlands | 4722 (4401, 5013) | 366.71 (330.92, 402.78) | 7397 (6500, 8145) | 299.43 (254.89, 340.21) | -0.65 (-0.72, -0.57) | <0.001 |
| New Zealand | 699 (641, 757) | 280.19 (248.06, 312.56) | 1221 (1069, 1351) | 215.65 (183.45, 245.44) | -0.84 (-1.59, -0.08) | 0.031 |
| Nicaragua | 36 (30, 42) | 49.15 (37.97, 62.77) | 110 (87, 136) | 44.24 (31.9, 58.93) | -0.36 (-0.83, 0.11) | 0.129 |
| Niger | 36 (25, 54) | 34.6 (22.21, 54.83) | 132 (93, 220) | 40.1 (25.39, 68.87) | 0.49 (0.38, 0.61) | <0.001 |
| Nigeria | 216 (157, 275) | 10.26 (7.42, 13.17) | 495 (414, 600) | 13.32 (10.8, 16.4) | 0.84 (0.74, 0.94) | <0.001 |
| Niue | 0 (0, 0) | 195.76 (138.86, 265.43) | 0 (0, 0) | 258.28 (175.66, 357.69) | 0.91 (0.83, 0.98) | <0.001 |
| North Macedonia | 155 (131, 184) | 174.17 (139.99, 214.1) | 393 (309, 485) | 210.38 (162.99, 263.48) | 0.61 (0.31, 0.9) | <0.001 |
| Northern Mariana Islands | 2 (2, 2) | 424.42 (322.59, 556.52) | 7 (6, 8) | 349.1 (260.85, 452.34) | -0.64 (-0.84, -0.43) | <0.001 |
| Norway | 880 (815, 918) | 180.06 (167.29, 190.37) | 1705 (1486, 1829) | 244 (212.88, 264.66) | 0.97 (0.37, 1.57) | 0.002 |
| Oman | 13 (9, 18) | 43.51 (27.97, 63.86) | 27 (20, 36) | 40.07 (27.81, 57.2) | -0.23 (-1.13, 0.68) | 0.619 |
| Pakistan | 2549 (1977, 3081) | 90.29 (67.44, 116.65) | 5084 (3841, 6611) | 100.97 (73.24, 136.24) | 0.37 (0.28, 0.45) | <0.001 |
| Palau | 2 (2, 3) | 437.18 (324.95, 581.07) | 4 (3, 5) | 447.77 (328.77, 589) | 0.03 (-0.1, 0.15) | 0.691 |
| Palestine | 75 (57, 97) | 180.79 (122.3, 256.19) | 163 (132, 196) | 160.33 (118.99, 212.49) | -0.38 (-0.78, 0.02) | 0.063 |
| Panama | 89 (81, 98) | 110.61 (94.82, 127.06) | 211 (160, 254) | 79.22 (60.01, 98.22) | -1 (-1.58, -0.43) | 0.001 |
| Papua New Guinea | 80 (50, 135) | 134.54 (78.15, 232.32) | 279 (177, 455) | 161.67 (95.84, 270.39) | 0.63 (0.57, 0.69) | <0.001 |
| Paraguay | 89 (74, 108) | 77.08 (59.8, 97.97) | 415 (312, 557) | 137.65 (95.28, 191.37) | 2.12 (1.8, 2.44) | <0.001 |
| Peru | 768 (640, 918) | 127.44 (99.56, 161.19) | 1992 (1434, 2568) | 103.6 (72.02, 139.56) | -0.58 (-2.05, 0.92) | 0.448 |
| Philippines | 1731 (1553, 1966) | 143.24 (126.65, 164.82) | 4632 (3932, 5431) | 128.17 (107.43, 151.04) | -0.33 (-0.6, -0.07) | 0.013 |
| Poland | 5828 (5588, 6026) | 240.48 (229.62, 249.61) | 15459 (13890, 16811) | 332.1 (297.18, 362.33) | 1 (0.9, 1.09) | <0.001 |
| Portugal | 1255 (1138, 1370) | 146.53 (128.38, 166.42) | 2647 (2260, 2969) | 146.24 (122.34, 170.61) | 0 (-0.22, 0.21) | 0.967 |
| Puerto Rico | 326 (302, 349) | 147.79 (130.32, 165.93) | 457 (371, 539) | 84.28 (67.59, 102.76) | -2.05 (-2.63, -1.47) | <0.001 |
| Qatar | 6 (5, 8) | 251.68 (180.45, 342.4) | 23 (18, 33) | 135.94 (93.55, 201.43) | -1.94 (-3.12, -0.74) | 0.002 |
| Republic of Korea | 2523 (2224, 2878) | 199.57 (164.85, 238.32) | 15553 (12489, 18525) | 273.1 (211.86, 338.93) | 0.94 (0.57, 1.3) | <0.001 |
| Republic of Moldova | 319 (295, 342) | 151.09 (135.18, 167.18) | 333 (294, 374) | 98.24 (85.23, 113.65) | -1.49 (-2.5, -0.48) | 0.004 |
| Romania | 1291 (1174, 1410) | 91.82 (81.16, 102.87) | 4414 (3879, 4993) | 176.98 (151.67, 206.16) | 2.15 (1.85, 2.44) | <0.001 |
| Russian Federation | 15700 (15134, 16112) | 160.79 (154.56, 165.82) | 20844 (19036, 22558) | 146.87 (132.75, 159.65) | -0.26 (-0.9, 0.38) | 0.423 |
| Rwanda | 66 (49, 82) | 57.9 (40.64, 78.01) | 144 (98, 198) | 59.53 (38.52, 87.16) | 0.1 (-0.08, 0.27) | 0.281 |
| Saint Kitts and Nevis | 2 (2, 3) | 92.55 (81.08, 105.07) | 2 (2, 3) | 87.53 (72.6, 103.59) | -0.12 (-0.64, 0.39) | 0.636 |
| Saint Lucia | 6 (5, 6) | 119.58 (104.41, 135.58) | 13 (10, 15) | 91.76 (73.58, 111.53) | -0.97 (-1.49, -0.45) | <0.001 |
| Saint Vincent and the Grenadines | 4 (3, 4) | 83.71 (73.52, 94.86) | 6 (5, 7) | 74.72 (63.45, 87.49) | -0.39 (-0.89, 0.11) | 0.128 |
| Samoa | 3 (2, 3) | 67.67 (49.9, 90.4) | 5 (3, 6) | 65.76 (45.24, 90.46) | -0.1 (-0.19, -0.01) | 0.032 |
| San Marino | 7 (6, 9) | 309.46 (235.01, 399.2) | 8 (6, 11) | 153.54 (97.74, 223.63) | -2.54 (-2.77, -2.31) | <0.001 |
| Sao Tome and Principe | 4 (3, 4) | 113.61 (83.55, 150.02) | 8 (6, 10) | 165.9 (114.74, 237.43) | 1.26 (1.13, 1.4) | <0.001 |
| Saudi Arabia | 124 (95, 159) | 50.37 (35.64, 70.03) | 281 (226, 347) | 55.64 (39.64, 76.23) | 0.31 (0.16, 0.45) | <0.001 |
| Senegal | 88 (67, 112) | 57.81 (40.01, 81.05) | 257 (193, 330) | 73.1 (49.16, 103.15) | 0.81 (0.64, 0.98) | <0.001 |
| Serbia | 1145 (917, 1420) | 247.69 (188.49, 323.1) | 2719 (2153, 3374) | 253.46 (191.88, 322.14) | 0.12 (-0.22, 0.46) | 0.484 |
| Seychelles | 5 (4, 6) | 149.84 (114.94, 191.97) | 7 (6, 9) | 130.16 (99.75, 169.44) | -0.74 (-0.95, -0.52) | <0.001 |
| Sierra Leone | 56 (44, 74) | 51.75 (36.48, 73.11) | 91 (64, 125) | 53.26 (34.9, 77.4) | 0.12 (0.04, 0.21) | 0.004 |
| Singapore | 324 (298, 350) | 308.93 (276.18, 342.53) | 856 (747, 960) | 177.08 (149.26, 204.12) | -1.76 (-2.67, -0.84) | <0.001 |
| Slovakia | 768 (637, 903) | 229.39 (181.11, 284.76) | 1092 (855, 1354) | 181.58 (134.48, 237.57) | -0.72 (-1.11, -0.32) | <0.001 |
| Slovenia | 270 (246, 292) | 198.15 (176.33, 219.91) | 704 (601, 794) | 233 (193.68, 273.18) | 0.57 (0.33, 0.81) | <0.001 |
| Solomon Islands | 7 (5, 9) | 141.05 (95.61, 199.21) | 18 (14, 23) | 136.58 (100.77, 183.84) | -0.09 (-0.19, 0.02) | 0.108 |
| Somalia | 26 (17, 44) | 36.51 (22.04, 64.11) | 65 (41, 116) | 32.74 (19.5, 61.73) | -0.34 (-0.39, -0.28) | <0.001 |
| South Africa | 1169 (998, 1430) | 116.07 (95.99, 143) | 3092 (2780, 3426) | 141.43 (123.58, 159.85) | 0.64 (0.1, 1.19) | 0.021 |
| South Sudan | 70 (45, 104) | 53.65 (33.03, 82.43) | 79 (57, 107) | 57.62 (38.8, 82.21) | 0.22 (0.15, 0.29) | <0.001 |
| Spain | 7353 (6806, 7821) | 212.38 (189.22, 235.51) | 12912 (11115, 14364) | 187.78 (157.01, 219.01) | -0.38 (-0.62, -0.15) | 0.001 |
| Sri Lanka | 344 (293, 414) | 72.01 (54.36, 95.5) | 854 (576, 1157) | 56.75 (37.23, 81.98) | -0.81 (-1.31, -0.31) | 0.002 |
| Sudan | 265 (182, 438) | 57.59 (36.16, 98.14) | 631 (432, 929) | 74.86 (47.59, 113.71) | 0.85 (0.78, 0.92) | <0.001 |
| Suriname | 13 (11, 15) | 106.19 (83.18, 133.72) | 32 (24, 43) | 97.23 (68.31, 134.7) | -0.03 (-0.7, 0.66) | 0.941 |
| Sweden | 1681 (1529, 1811) | 153.24 (135.83, 169.78) | 3056 (2567, 3478) | 185.9 (154.61, 217.8) | 0.65 (0.27, 1.02) | 0.001 |
| Switzerland | 1378 (1249, 1488) | 197.5 (173.35, 221.91) | 2284 (1945, 2511) | 178.46 (149.44, 205.5) | -0.35 (-0.63, -0.07) | 0.014 |
| Syrian Arab Republic | 211 (158, 266) | 95.47 (68.01, 127.21) | 557 (403, 726) | 101.38 (69.87, 140.64) | 0.18 (-0.21, 0.56) | 0.372 |
| Taiwan (Province of China) | 1552 (1460, 1639) | 210.99 (189.37, 232.87) | 6582 (5787, 7190) | 254.24 (215.41, 292.12) | 0.49 (0.23, 0.76) | <0.001 |
| Tajikistan | 116 (92, 143) | 91.48 (70.86, 115.22) | 109 (72, 158) | 54.61 (35.41, 79.17) | -1.74 (-2.31, -1.17) | <0.001 |
| Thailand | 3995 (3284, 4773) | 264.81 (199.25, 344.56) | 13914 (10693, 17126) | 219.91 (158.31, 291.07) | -0.62 (-0.89, -0.34) | <0.001 |
| Timor-Leste | 9 (6, 12) | 102.15 (65.53, 153.81) | 54 (40, 70) | 122.33 (85.34, 175.21) | 0.57 (0.48, 0.66) | <0.001 |
| Togo | 28 (21, 37) | 54.76 (37.26, 77.29) | 89 (59, 127) | 62.31 (39.94, 91.84) | 0.41 (0.29, 0.53) | <0.001 |
| Tokelau | 0 (0, 0) | 193.91 (139.57, 262.72) | 0 (0, 0) | 205.75 (144.75, 278.73) | 0.2 (0.01, 0.39) | 0.036 |
| Tonga | 7 (5, 8) | 252.7 (187.72, 336.41) | 12 (9, 15) | 262.22 (189.65, 348.95) | 0.15 (-0.16, 0.46) | 0.349 |
| Trinidad and Tobago | 43 (40, 46) | 92.48 (81.93, 104.24) | 81 (64, 101) | 73.01 (55.95, 92.07) | -0.8 (-0.94, -0.66) | <0.001 |
| Tunisia | 390 (314, 483) | 158.14 (111.49, 215.2) | 922 (629, 1274) | 132.19 (83.2, 195.18) | -0.67 (-0.9, -0.44) | <0.001 |
| Turkey | 4881 (3776, 6243) | 324.18 (232.58, 443.77) | 13016 (10318, 16279) | 257.07 (189.58, 339.39) | -0.8 (-1.19, -0.42) | <0.001 |
| Turkmenistan | 65 (58, 71) | 75.8 (66.26, 85.03) | 72 (57, 93) | 46.69 (36.51, 59.99) | -1.5 (-2.25, -0.74) | <0.001 |
| Tuvalu | 0 (0, 1) | 166.53 (117.61, 251.77) | 1 (1, 1) | 200.03 (143.62, 279.4) | 0.61 (0.56, 0.66) | <0.001 |
| Uganda | 131 (105, 160) | 44.05 (32.09, 58.53) | 320 (242, 414) | 53.42 (36.73, 74.6) | 0.65 (0.52, 0.77) | <0.001 |
| Ukraine | 7057 (6620, 7515) | 174.24 (157.29, 190.88) | 4351 (3334, 5588) | 90.44 (67.41, 118.01) | -2.14 (-3.01, -1.26) | <0.001 |
| United Arab Emirates | 18 (12, 25) | 144.54 (93.03, 209.27) | 79 (58, 115) | 167.07 (113.79, 248.52) | 0.36 (-3.35, 4.21) | 0.852 |
| United Kingdom | 23851 (22714, 24457) | 379.49 (360.09, 390.84) | 27860 (24659, 29386) | 293.35 (262.76, 309.93) | -0.85 (-1.13, -0.56) | <0.001 |
| United Republic of Tanzania | 232 (179, 305) | 46.86 (34.17, 65.84) | 581 (428, 769) | 52.83 (37.61, 73.44) | 0.4 (0.3, 0.5) | <0.001 |
| United States of America | 75156 (69425, 78369) | 354.11 (325.63, 370.04) | 108636 (95723, 116057) | 279.58 (244.73, 298.95) | -0.77 (-0.94, -0.61) | <0.001 |
| United States Virgin Islands | 6 (4, 7) | 143.17 (106.08, 189.6) | 13 (10, 18) | 105.77 (73, 149.32) | -0.95 (-1.46, -0.44) | <0.001 |
| Uruguay | 647 (598, 691) | 262.65 (232.13, 294.45) | 910 (823, 1002) | 242.94 (212.42, 276.45) | -0.2 (-0.38, -0.03) | 0.021 |
| Uzbekistan | 429 (381, 474) | 78.88 (69.71, 88.96) | 409 (339, 488) | 43.05 (35.25, 51.71) | -1.76 (-2.49, -1.03) | <0.001 |
| Vanuatu | 3 (2, 6) | 150.43 (90.58, 258.51) | 10 (7, 16) | 154.12 (96.86, 253.53) | 0.09 (0, 0.18) | 0.065 |
| Venezuela (Bolivarian Republic of) | 641 (592, 689) | 135.79 (119.89, 151.25) | 2309 (1795, 2897) | 145.72 (110.4, 184.02) | 0.31 (-0.36, 1) | 0.365 |
| Viet Nam | 2499 (2035, 3066) | 121.48 (91.38, 160.74) | 6586 (5186, 7862) | 147.23 (106.74, 192.61) | 0.66 (0.49, 0.82) | <0.001 |
| Yemen | 117 (71, 206) | 59.53 (34.23, 107.17) | 474 (291, 767) | 81.31 (47.25, 134.85) | 1.05 (0.92, 1.17) | <0.001 |
| Zambia | 67 (53, 82) | 56.72 (41.4, 76.44) | 200 (151, 279) | 74.76 (51.43, 108.87) | 0.95 (0.81, 1.08) | <0.001 |
| Zimbabwe | 175 (140, 207) | 96.09 (71.41, 124.44) | 281 (223, 358) | 104.64 (76.6, 139.64) | 0.32 (0.19, 0.44) | <0.001 |

Notes: ASMR rates are reported per 100,000 population.

Abbreviations: ASMR, age-standardized mortality rate; EOLC, early-onset lung cancer; MOLC, middle-onset lung cancer; LOLC, late-onset lung cancer; AAPC, average annual percentage change; UI, uncertainty interval; CI, confidence interval.

# **Supplementary Table S7.** DALYs number and ASDR of EOLC, MOLC and LOLC in 1990 and 2021, and AAPC from 1990 to 2021 among 204 countries and territories.

| Country/Territory | Cases (95%UI), 1990 | ASDR (95% UI), 1990 | Cases (95%UI), 2021 | ASDR (95% UI), 2021 | AAPC (95% CI), 1990-2021 | P value |
| --- | --- | --- | --- | --- | --- | --- |
| EOLC |  |  |  |  |  |  |
| Afghanistan | 2872 (1410, 4519) | 83.68 (39.74, 146.42) | 12138 (7283, 17729) | 105.26 (59.81, 170.23) | 0.79 (0.66, 0.91) | <0.001 |
| Albania | 2209 (1736, 2756) | 171.19 (127.79, 219.9) | 1936 (1340, 2640) | 152.22 (105.1, 210.08) | -0.51 (-1.47, 0.46) | 0.302 |
| Algeria | 4118 (3096, 5276) | 54.32 (36.48, 77.26) | 9085 (6538, 12724) | 39.88 (26.04, 59.38) | -0.98 (-1.16, -0.8) | <0.001 |
| American Samoa | 39 (31, 51) | 210.46 (143.12, 300.61) | 52 (38, 68) | 207.64 (138.66, 294.41) | -0.03 (-0.27, 0.22) | 0.839 |
| Andorra | 76 (51, 111) | 253.05 (159.6, 385.24) | 75 (48, 114) | 124.14 (76.01, 191.75) | -2.47 (-2.9, -2.03) | <0.001 |
| Angola | 2796 (1864, 4059) | 86.37 (53.44, 134.55) | 8544 (5812, 12218) | 80.72 (50.58, 121.38) | -0.2 (-0.66, 0.25) | 0.383 |
| Antigua and Barbuda | 16 (14, 18) | 65.47 (55.94, 76.84) | 21 (18, 23) | 40.08 (34.07, 46.78) | -0.93 (-1.75, -0.1) | 0.028 |
| Argentina | 45836 (41350, 50409) | 308.78 (260.92, 363.71) | 24611 (21560, 27819) | 103.11 (84.74, 123.61) | -3.5 (-3.98, -3.02) | <0.001 |
| Armenia | 5195 (4803, 5559) | 411.44 (380.71, 441.13) | 2309 (2031, 2631) | 151.04 (132.09, 172.4) | -3.06 (-3.67, -2.45) | <0.001 |
| Australia | 13079 (11714, 14379) | 150.88 (131.4, 170.91) | 11377 (9952, 12994) | 84.38 (71.59, 98.91) | -1.8 (-2.39, -1.21) | <0.001 |
| Austria | 9718 (8863, 10586) | 234.52 (203.87, 270.41) | 3999 (3572, 4519) | 83.07 (70.1, 98.26) | -3.47 (-4.08, -2.86) | <0.001 |
| Azerbaijan | 6893 (5631, 8423) | 281.49 (222.27, 352.07) | 7469 (4987, 10558) | 134.65 (89.69, 187.64) | -2.36 (-3.23, -1.48) | <0.001 |
| Bahamas | 159 (139, 179) | 150.34 (127.73, 175.94) | 286 (222, 380) | 131.79 (99.11, 177.12) | -0.36 (-1.08, 0.36) | 0.323 |
| Bahrain | 318 (255, 379) | 147.31 (107.28, 195.87) | 990 (749, 1296) | 91.53 (63.43, 129.62) | -1.5 (-1.82, -1.19) | <0.001 |
| Bangladesh | 20062 (14371, 29124) | 58.47 (36.8, 92.38) | 34734 (23687, 54373) | 43.56 (28.04, 71.55) | -0.92 (-1.25, -0.6) | <0.001 |
| Barbados | 82 (74, 90) | 73.91 (63.95, 84.44) | 88 (65, 117) | 54.57 (40.49, 72.72) | -0.91 (-1.8, -0.01) | 0.047 |
| Belarus | 14461 (12574, 16454) | 321.63 (269.14, 376.4) | 6972 (5181, 8836) | 132.39 (96.16, 171.12) | -2.77 (-3.72, -1.8) | <0.001 |
| Belgium | 16088 (14604, 17635) | 330.43 (283.47, 378.84) | 7644 (6785, 8511) | 130.21 (109.55, 153.58) | -3.2 (-3.52, -2.88) | <0.001 |
| Belize | 38 (34, 42) | 68.77 (59.88, 79.27) | 182 (153, 217) | 90.32 (73.41, 109.81) | 0.84 (0.21, 1.48) | 0.009 |
| Benin | 465 (343, 597) | 33.79 (22.6, 48.05) | 1453 (975, 2132) | 32.11 (19.47, 51.46) | -0.21 (-0.35, -0.07) | 0.003 |
| Bermuda | 81 (69, 95) | 246.06 (202.47, 297.2) | 44 (34, 56) | 124.79 (92.93, 159.25) | -2.33 (-2.68, -1.99) | <0.001 |
| Bhutan | 85 (47, 125) | 42.97 (22.33, 67.3) | 163 (101, 252) | 42.1 (24.5, 67.77) | -0.11 (-0.18, -0.04) | 0.002 |
| Bolivia (Plurinational State of) | 2528 (1913, 3296) | 108.64 (74.92, 152.92) | 4497 (2981, 6380) | 79.81 (50.12, 118.6) | -0.97 (-1.04, -0.9) | <0.001 |
| Bosnia and Herzegovina | 5858 (4493, 7384) | 283.47 (216.93, 361.62) | 3345 (2166, 4633) | 180.15 (116.1, 251.44) | -1.56 (-2.18, -0.93) | <0.001 |
| Botswana | 490 (329, 701) | 129.59 (78.02, 203.48) | 1237 (813, 1708) | 102.77 (64.07, 152.17) | -0.73 (-1.17, -0.29) | 0.001 |
| Brazil | 76095 (73362, 79290) | 127.75 (120.37, 135.29) | 104838 (99551, 110111) | 86.62 (81.27, 92.29) | -1.26 (-1.57, -0.96) | <0.001 |
| Brunei Darussalam | 204 (150, 273) | 184.76 (127.11, 260.83) | 396 (300, 499) | 144.05 (104.22, 191.96) | -0.82 (-1.31, -0.33) | 0.001 |
| Bulgaria | 17815 (15544, 20325) | 390.11 (327.95, 457.51) | 10471 (8400, 12762) | 259.93 (206.63, 318.7) | -1.27 (-2.26, -0.26) | 0.014 |
| Burkina Faso | 815 (567, 1221) | 29.2 (18.41, 47.84) | 2328 (1563, 3470) | 31.24 (18.93, 51.16) | 0.24 (-0.01, 0.49) | 0.061 |
| Burundi | 913 (630, 1310) | 58.57 (37.13, 91.52) | 1774 (1165, 2717) | 41.56 (24.3, 68.91) | -1.08 (-1.39, -0.78) | <0.001 |
| Cabo Verde | 98 (78, 122) | 96.64 (67.34, 134.13) | 237 (172, 333) | 84.62 (54.44, 129.53) | -0.5 (-0.99, -0.02) | 0.042 |
| Cambodia | 5325 (3865, 7307) | 162.95 (107.61, 242.72) | 11789 (8061, 17129) | 153.42 (99.38, 230.8) | -0.19 (-0.34, -0.04) | 0.012 |
| Cameroon | 1426 (1096, 1784) | 44.31 (30.78, 61.94) | 5462 (3374, 7823) | 47.81 (27.49, 73.92) | 0.23 (0.12, 0.34) | <0.001 |
| Canada | 38895 (35608, 41849) | 270.2 (239.01, 300.71) | 14589 (12625, 16698) | 77.49 (65.37, 91.85) | -4.05 (-4.34, -3.75) | <0.001 |
| Central African Republic | 787 (467, 1468) | 92.16 (51.44, 179.36) | 1652 (840, 3317) | 82.59 (39.46, 171.83) | -0.4 (-0.69, -0.1) | 0.008 |
| Chad | 439 (310, 647) | 25.04 (16.22, 39.34) | 1751 (1190, 2553) | 34.81 (21.44, 54.82) | 1.19 (1.07, 1.3) | <0.001 |
| Chile | 7919 (6763, 9321) | 135.61 (113.68, 161.79) | 6822 (5553, 8361) | 69.26 (55.15, 85.95) | -2.07 (-2.92, -1.22) | <0.001 |
| China | 1603077 (1374283, 1855486) | 303.53 (255.85, 354.18) | 1940033 (1512282, 2377447) | 242.62 (189.33, 300.16) | -0.74 (-0.91, -0.57) | <0.001 |
| Colombia | 15495 (14126, 16944) | 117.36 (100.73, 135.58) | 14315 (11547, 17498) | 57.54 (44.25, 73.15) | -2.32 (-3.05, -1.58) | <0.001 |
| Comoros | 72 (45, 106) | 52.09 (30.98, 78.9) | 183 (120, 261) | 53.82 (32.26, 82.37) | -0.24 (-1.68, 1.22) | 0.745 |
| Congo | 880 (545, 1199) | 125.64 (72.83, 189.01) | 2337 (1495, 3561) | 95.42 (57.86, 150.98) | -0.86 (-1.51, -0.21) | 0.009 |
| Cook Islands | 16 (12, 22) | 206.94 (139.18, 303.86) | 16 (11, 21) | 184.01 (119.97, 278.27) | -0.38 (-0.49, -0.26) | <0.001 |
| Costa Rica | 696 (616, 776) | 61.05 (51.79, 71.7) | 1231 (1041, 1454) | 50.13 (40.91, 61.36) | -0.93 (-1.82, -0.02) | 0.044 |
| Côte d'Ivoire | 824 (609, 1092) | 22.31 (14.82, 31.88) | 2414 (1582, 3655) | 22.79 (13.67, 37.72) | -2.66 (-3.5, -1.81) | <0.001 |
| Croatia | 8840 (7839, 9953) | 351.95 (297.26, 412.21) | 3512 (2894, 4222) | 154.29 (125.45, 187.16) | -2.07 (-2.46, -1.68) | <0.001 |
| Cuba | 13307 (12078, 14458) | 255.52 (219.8, 294.49) | 8243 (6733, 9894) | 135.66 (108.93, 165.84) | -0.27 (-0.81, 0.27) | 0.32 |
| Cyprus | 327 (248, 420) | 84.85 (57.89, 118.75) | 633 (480, 826) | 78.51 (54.17, 109.39) | -4.27 (-4.74, -3.8) | <0.001 |
| Czechia | 22869 (19950, 25778) | 396.37 (339.2, 458.72) | 6669 (5430, 8125) | 102.04 (82.36, 125.82) | 0.07 (-0.22, 0.38) | 0.623 |
| Democratic People's Republic of Korea | 24038 (16260, 35376) | 243.1 (153.08, 370.76) | 35019 (21229, 56885) | 232.35 (131.61, 394.79) | -0.13 (-0.2, -0.05) | 0.001 |
| Democratic Republic of the Congo | 6341 (4035, 10158) | 57.67 (34.2, 97.4) | 16348 (9501, 28881) | 53.07 (28.04, 99.86) | -0.29 (-0.48, -0.1) | 0.003 |
| Denmark | 8645 (8005, 9271) | 292.4 (257.28, 328.96) | 3049 (2675, 3480) | 103.82 (87.98, 121.81) | -3.31 (-3.87, -2.74) | <0.001 |
| Djibouti | 76 (50, 111) | 56.04 (33.5, 89.18) | 417 (246, 645) | 68.34 (38.19, 113.72) | 0.6 (0.43, 0.76) | <0.001 |
| Dominica | 26 (21, 32) | 100.29 (75.4, 132.38) | 39 (27, 52) | 118.38 (76.78, 171.52) | 0.53 (0.37, 0.7) | <0.001 |
| Dominican Republic | 2629 (2155, 3211) | 102.69 (78.76, 134.75) | 6943 (4818, 9696) | 128.16 (84.81, 182.65) | 0.82 (0.03, 1.61) | 0.041 |
| Ecuador | 3137 (2850, 3449) | 80.69 (68.08, 95.15) | 5382 (4071, 6956) | 61.44 (44.73, 83.04) | -0.85 (-2.33, 0.65) | 0.266 |
| Egypt | 17123 (14173, 20746) | 78.13 (58.49, 103.38) | 54638 (41660, 70429) | 114.13 (80.7, 156.62) | 1.19 (0.83, 1.55) | <0.001 |
| El Salvador | 1532 (1304, 1797) | 81.26 (64.74, 100.26) | 2405 (1806, 3110) | 80.94 (58.59, 108.11) | -0.1 (-1.12, 0.92) | 0.841 |
| Equatorial Guinea | 115 (68, 200) | 87.1 (49.08, 154.24) | 500 (297, 784) | 96.35 (55.61, 158.5) | 0.37 (-0.41, 1.15) | 0.357 |
| Eritrea | 736 (515, 1052) | 69.24 (44.85, 104.42) | 2043 (1248, 3092) | 78.05 (45.62, 122.57) | 0.37 (0.15, 0.58) | 0.001 |
| Estonia | 2639 (2342, 2990) | 342.89 (291.56, 402.74) | 684 (535, 816) | 96.88 (74.88, 117.35) | -3.98 (-4.84, -3.11) | <0.001 |
| Eswatini | 280 (167, 474) | 122.39 (67.05, 213.51) | 863 (455, 1379) | 193.23 (93.65, 327.64) | 1.52 (1.22, 1.83) | <0.001 |
| Ethiopia | 14667 (10019, 21252) | 99.36 (65.63, 150.81) | 19021 (14907, 23663) | 50.46 (37.79, 64.98) | -2.15 (-2.27, -2.04) | <0.001 |
| Fiji | 255 (191, 341) | 80.52 (53.02, 117.01) | 293 (197, 417) | 65.04 (40.63, 99.16) | -0.7 (-0.86, -0.53) | <0.001 |
| Finland | 4632 (4247, 5047) | 159.07 (136.49, 184.92) | 2154 (1919, 2398) | 81.66 (68.6, 97.01) | -2.03 (-2.69, -1.36) | <0.001 |
| France | 87519 (77849, 98279) | 311.34 (270.51, 359.02) | 63685 (56363, 71694) | 189.87 (163.93, 218.1) | -1.53 (-1.65, -1.42) | <0.001 |
| Gabon | 333 (211, 490) | 113.74 (63.1, 181.61) | 774 (510, 1149) | 104.44 (61.26, 164.57) | -0.17 (-0.42, 0.07) | 0.162 |
| Gambia | 51 (37, 69) | 17.77 (11.4, 27.02) | 150 (100, 224) | 18.38 (11.22, 29.47) | 0.14 (-1.12, 1.41) | 0.828 |
| Georgia | 7702 (6719, 8869) | 322.43 (278.43, 372.81) | 3383 (2869, 3962) | 184.16 (154.85, 217.55) | -1.54 (-2.57, -0.49) | 0.004 |
| Germany | 112206 (101926, 122598) | 269.93 (233.86, 309.2) | 43415 (38864, 48614) | 106.24 (90.46, 123.19) | -3.04 (-3.42, -2.66) | <0.001 |
| Ghana | 1469 (1077, 1927) | 29.24 (19.35, 42.93) | 3255 (2335, 4476) | 23.2 (14.77, 35.12) | -0.74 (-0.84, -0.64) | <0.001 |
| Greece | 14349 (13181, 15603) | 278.33 (245.24, 313.55) | 8480 (7763, 9271) | 144.24 (127.93, 161.06) | -2.09 (-2.44, -1.75) | <0.001 |
| Greenland | 216 (163, 276) | 732.42 (533.76, 986.04) | 82 (57, 110) | 327.9 (221.3, 454.92) | -2.46 (-2.66, -2.27) | <0.001 |
| Grenada | 29 (25, 34) | 105.49 (83.56, 129.79) | 41 (33, 50) | 77.45 (60.23, 99.53) | -0.92 (-1.92, 0.1) | 0.077 |
| Guam | 139 (121, 158) | 219.54 (171.61, 276.34) | 215 (182, 251) | 279.02 (217.93, 355.67) | 0.77 (0.12, 1.42) | 0.02 |
| Guatemala | 2368 (2233, 2512) | 86.53 (76.46, 97.12) | 3744 (3158, 4380) | 53.93 (44.36, 64.91) | -1.48 (-2.58, -0.35) | 0.01 |
| Guinea | 819 (612, 1069) | 42.07 (28.4, 61.18) | 2252 (1463, 3285) | 53.05 (31.78, 82.64) | 0.76 (0.58, 0.93) | <0.001 |
| Guinea-Bissau | 166 (111, 243) | 55.62 (33.62, 87.35) | 405 (276, 579) | 56.56 (34.8, 87.63) | 0.06 (-0.02, 0.16) | 0.152 |
| Guyana | 167 (139, 198) | 60.9 (47.71, 76.53) | 259 (183, 345) | 71.92 (49.31, 99.96) | 0.7 (0.18, 1.23) | 0.009 |
| Haiti | 2455 (1538, 3521) | 112.66 (67.08, 172.23) | 4682 (2818, 7196) | 81.54 (46.59, 136.15) | -0.92 (-1.04, -0.8) | <0.001 |
| Honduras | 1823 (1431, 2294) | 121.48 (87.8, 169.27) | 5008 (2950, 7769) | 116.09 (62.76, 196.42) | -0.15 (-0.36, 0.06) | 0.158 |
| Hungary | 32539 (28474, 36907) | 577.99 (497.4, 669.46) | 11016 (8898, 13440) | 183.36 (146.89, 225.23) | -3.69 (-4.34, -3.04) | <0.001 |
| Iceland | 215 (191, 242) | 185.95 (159.2, 215.61) | 300 (259, 346) | 166.84 (139.53, 196.72) | -0.4 (-0.73, -0.07) | 0.019 |
| India | 178017 (154415, 206802) | 51.95 (44.49, 60.89) | 422904 (357515, 488301) | 59.78 (49.76, 70.78) | 0.44 (0.15, 0.72) | 0.003 |
| Indonesia | 96938 (75578, 116862) | 131.59 (101.23, 160.69) | 224544 (155676, 312164) | 143.4 (97.85, 197.42) | 0.28 (0.21, 0.36) | <0.001 |
| Iran (Islamic Republic of) | 16759 (13379, 19833) | 88.9 (70.9, 107.2) | 42493 (38271, 47741) | 84.12 (74.44, 96.26) | -0.26 (-0.41, -0.11) | 0.001 |
| Iraq | 5974 (4195, 8340) | 106.88 (68.75, 159.85) | 17501 (11965, 24966) | 89.85 (57.5, 135.89) | -0.63 (-0.81, -0.45) | <0.001 |
| Ireland | 3073 (2792, 3353) | 188.9 (160.8, 219.62) | 3124 (2715, 3539) | 110.4 (91.32, 130.96) | -1.83 (-2.86, -0.78) | 0.001 |
| Israel | 3130 (2769, 3512) | 154.41 (131.66, 180.05) | 3605 (3132, 4129) | 77.37 (65.43, 90.67) | -2.04 (-2.38, -1.7) | <0.001 |
| Italy | 68357 (65426, 71135) | 234.89 (222.61, 247.32) | 30923 (29149, 32619) | 91.56 (85.18, 97.32) | -3.22 (-3.57, -2.87) | <0.001 |
| Jamaica | 1058 (919, 1199) | 136.03 (108.26, 165.85) | 1455 (1043, 2025) | 103.5 (69.89, 147.62) | -0.82 (-2.49, 0.87) | 0.34 |
| Japan | 95544 (92968, 98443) | 128 (123.77, 132.64) | 42717 (41340, 44058) | 61.53 (59.16, 63.87) | -2.38 (-2.81, -1.95) | <0.001 |
| Jordan | 1137 (868, 1442) | 104.39 (70.71, 144.29) | 4485 (3341, 6166) | 74.23 (49.73, 110.69) | -1.02 (-1.4, -0.64) | <0.001 |
| Kazakhstan | 29072 (25955, 32356) | 460.42 (405.56, 518.69) | 10574 (9241, 11951) | 108.92 (94.92, 124.08) | -4.52 (-4.96, -4.08) | <0.001 |
| Kenya | 888 (594, 1294) | 14.21 (9.4, 20.65) | 4293 (3285, 5703) | 22.48 (17.05, 30.17) | 1.47 (1.32, 1.62) | <0.001 |
| Kiribati | 37 (28, 48) | 129.07 (85.99, 186.86) | 85 (59, 119) | 157.92 (95.22, 249.78) | 0.66 (0.56, 0.75) | <0.001 |
| Kuwait | 528 (455, 604) | 69.78 (55.89, 84.82) | 1472 (1170, 1904) | 41.62 (31.22, 55.54) | -1.61 (-2.26, -0.97) | <0.001 |
| Kyrgyzstan | 4328 (3718, 4972) | 310.12 (257.27, 364.22) | 2758 (2179, 3405) | 88.72 (69.47, 110.57) | -4.23 (-4.48, -3.99) | <0.001 |
| Lao People's Democratic Republic | 2457 (1551, 3882) | 178.26 (104.97, 296.01) | 4945 (3260, 7279) | 143.71 (89.56, 226.17) | -0.66 (-0.76, -0.56) | <0.001 |
| Latvia | 4189 (3662, 4812) | 308.35 (259.05, 365.55) | 1170 (928, 1415) | 117.71 (92.39, 145.92) | -3.2 (-4.4, -1.98) | <0.001 |
| Lebanon | 1814 (1300, 2460) | 151.67 (102.16, 217.89) | 3466 (2559, 4570) | 120.51 (83.04, 166.76) | -0.65 (-0.92, -0.38) | <0.001 |
| Lesotho | 358 (235, 568) | 71.13 (43.39, 117.93) | 1459 (925, 2251) | 207.7 (120.48, 344.7) | 3.57 (3.29, 3.85) | <0.001 |
| Liberia | 234 (168, 328) | 30.74 (19.79, 47.1) | 728 (415, 1253) | 31.22 (16.11, 56.6) | 0.07 (-0.24, 0.38) | 0.649 |
| Libya | 2034 (1446, 2781) | 154.84 (99.11, 234.37) | 7203 (4977, 10587) | 162.65 (102.29, 254.56) | 0.16 (0.06, 0.25) | 0.001 |
| Lithuania | 5331 (4768, 5904) | 297.5 (250.6, 346.81) | 1851 (1504, 2252) | 131.26 (101.52, 162.95) | -2.82 (-4.2, -1.41) | <0.001 |
| Luxembourg | 583 (534, 637) | 284.79 (249.47, 321.41) | 383 (335, 431) | 100.28 (85.6, 115.7) | -3.41 (-3.77, -3.05) | <0.001 |
| Madagascar | 1643 (1269, 2040) | 47.34 (32.67, 65.48) | 4517 (3162, 6249) | 43.4 (26.97, 65.31) | -0.22 (-0.32, -0.12) | <0.001 |
| Malawi | 417 (314, 551) | 14.57 (9.72, 21.09) | 1239 (886, 1739) | 19.41 (12.24, 29.9) | 0.98 (0.76, 1.2) | <0.001 |
| Malaysia | 6485 (5282, 8019) | 95.8 (70.98, 128.15) | 18548 (14622, 23420) | 116.39 (84.9, 156.87) | 0.73 (-0.07, 1.55) | 0.075 |
| Maldives | 57 (33, 78) | 88.12 (49.04, 134.12) | 139 (101, 177) | 40.63 (26.73, 58.65) | -2.55 (-2.72, -2.38) | <0.001 |
| Mali | 716 (562, 908) | 26.65 (18.8, 36.36) | 1939 (1377, 2734) | 27.57 (17.86, 41.17) | 0.12 (-0.05, 0.29) | 0.163 |
| Malta | 238 (207, 275) | 117.41 (95.6, 141.22) | 252 (212, 300) | 104.92 (84.43, 130.49) | -0.24 (-0.77, 0.29) | 0.366 |
| Marshall Islands | 27 (18, 39) | 193.07 (119.07, 306.36) | 69 (40, 105) | 246.26 (137.26, 390.11) | 0.79 (0.69, 0.89) | <0.001 |
| Mauritania | 266 (193, 359) | 41.41 (26.78, 62.07) | 531 (353, 796) | 34.47 (21.14, 55.32) | -0.6 (-0.81, -0.38) | <0.001 |
| Mauritius | 476 (428, 523) | 98.24 (85.72, 112.74) | 523 (464, 578) | 75.2 (64.14, 86.54) | -1.06 (-2.65, 0.56) | 0.198 |
| Mexico | 28872 (28024, 29721) | 90.28 (86.7, 93.83) | 34927 (31021, 39323) | 51.3 (45.36, 57.75) | -1.8 (-2.46, -1.14) | <0.001 |
| Micronesia (Federated States of) | 80 (53, 119) | 236.67 (143.86, 376.07) | 124 (78, 183) | 261.59 (151.21, 412.6) | 0.34 (0.28, 0.4) | <0.001 |
| Monaco | 64 (50, 84) | 364.19 (260.72, 497.02) | 84 (56, 118) | 467.04 (300.85, 681.52) | 0.78 (0.59, 0.97) | <0.001 |
| Mongolia | 1451 (1109, 1861) | 208.9 (145.38, 294.06) | 2957 (2254, 3756) | 171.12 (119.46, 237.49) | -0.71 (-1.58, 0.18) | 0.118 |
| Montenegro | 1080 (857, 1335) | 386.98 (300.64, 483.83) | 1054 (786, 1359) | 310.04 (230.02, 404.08) | -0.7 (-1.27, -0.13) | 0.016 |
| Morocco | 6357 (4725, 8531) | 72.86 (47.61, 108.32) | 10437 (6957, 16267) | 55.01 (33.63, 87.94) | -0.91 (-0.98, -0.84) | <0.001 |
| Mozambique | 912 (677, 1222) | 21.12 (13.9, 30.29) | 3182 (2097, 4678) | 33.52 (19.68, 53.75) | 1.54 (1.4, 1.69) | <0.001 |
| Myanmar | 24981 (16894, 34225) | 166.92 (105.88, 248.19) | 34467 (23711, 49449) | 122.81 (79.13, 180.97) | -0.98 (-1.07, -0.89) | <0.001 |
| Namibia | 176 (134, 224) | 40.8 (27.99, 57) | 473 (305, 691) | 46.24 (28.21, 71.36) | 0.5 (0.1, 0.89) | 0.013 |
| Nauru | 14 (7, 20) | 342.02 (165.8, 532.77) | 17 (8, 23) | 358.91 (170.05, 535.83) | 0.16 (0.09, 0.22) | <0.001 |
| Nepal | 3268 (2152, 4858) | 47.67 (28.73, 75.9) | 6243 (4244, 9301) | 45.3 (27.81, 71.96) | -0.15 (-0.35, 0.04) | 0.119 |
| Netherlands | 19651 (18263, 21108) | 243.6 (213.59, 277.2) | 10777 (9715, 11996) | 127.2 (110.88, 146.34) | -2.02 (-2.12, -1.92) | <0.001 |
| New Zealand | 2810 (2528, 3084) | 168.63 (149.02, 189.14) | 2584 (2273, 2896) | 100.82 (87.81, 114.84) | -1.76 (-2.21, -1.31) | <0.001 |
| Nicaragua | 554 (466, 649) | 45.85 (34.24, 59.57) | 1095 (854, 1384) | 34.56 (23.91, 48.08) | -1.19 (-1.61, -0.77) | <0.001 |
| Niger | 558 (379, 823) | 23.04 (14.44, 37.58) | 1354 (834, 2359) | 20.2 (11.63, 37.34) | -0.42 (-0.64, -0.2) | <0.001 |
| Nigeria | 2229 (1542, 3003) | 7.57 (5.16, 10.32) | 5952 (3999, 8167) | 7.71 (5.25, 10.72) | 0.04 (-0.09, 0.17) | 0.517 |
| Niue | 2 (1, 2) | 162.82 (101.87, 248.96) | 2 (1, 2) | 183.87 (113.56, 288) | 0.4 (0.11, 0.7) | 0.007 |
| North Macedonia | 2649 (2075, 3253) | 277.3 (213.77, 350.29) | 2417 (1765, 3189) | 182.55 (132.54, 242.11) | -1.19 (-1.7, -0.69) | <0.001 |
| Northern Mariana Islands | 80 (55, 112) | 327.98 (198.88, 499.06) | 62 (48, 78) | 223.03 (153.34, 322.38) | -1.27 (-1.45, -1.1) | <0.001 |
| Norway | 2782 (2649, 2918) | 126.24 (117.92, 134.44) | 2189 (2045, 2340) | 75.63 (69.81, 82.16) | -1.52 (-1.84, -1.2) | <0.001 |
| Oman | 233 (151, 336) | 32.62 (19.32, 50.02) | 504 (359, 742) | 17.97 (11.69, 27.85) | -2.05 (-2.45, -1.65) | <0.001 |
| Pakistan | 30281 (24090, 36946) | 82.89 (61.8, 107.19) | 97228 (72633, 128310) | 99.71 (70.91, 137.46) | 0.6 (0.48, 0.72) | <0.001 |
| Palau | 24 (17, 34) | 344.87 (229.87, 511.29) | 40 (30, 54) | 356.72 (244.23, 507.26) | 0.06 (-0.2, 0.31) | 0.662 |
| Palestine | 814 (551, 1156) | 138.79 (85.48, 210.49) | 2273 (1823, 2890) | 108.16 (76.51, 149.71) | -0.86 (-1.17, -0.54) | <0.001 |
| Panama | 859 (788, 931) | 88.8 (77.94, 100.56) | 1100 (860, 1354) | 52.64 (40.32, 66.22) | -1.59 (-2.47, -0.7) | <0.001 |
| Papua New Guinea | 1673 (1015, 2875) | 112.02 (60.22, 205.72) | 5389 (3371, 8723) | 117.78 (64.2, 204.43) | 0.17 (0.07, 0.26) | 0.001 |
| Paraguay | 983 (790, 1207) | 68.8 (50.54, 91.3) | 2648 (1901, 3618) | 79.35 (53.53, 113.99) | 0.66 (0.32, 1) | <0.001 |
| Peru | 10650 (8709, 13133) | 126.63 (94.7, 165.15) | 15802 (11024, 20998) | 85.7 (57.26, 120.25) | -1.19 (-2.84, 0.48) | 0.16 |
| Philippines | 36690 (32744, 41107) | 157 (137.06, 179.31) | 70450 (57879, 82808) | 131.55 (107.59, 157.14) | -0.61 (-0.73, -0.49) | <0.001 |
| Poland | 71453 (69050, 73842) | 405.86 (388.1, 423.9) | 28897 (26350, 31498) | 128.22 (116.17, 140.57) | -3.88 (-4.21, -3.56) | <0.001 |
| Portugal | 8974 (8022, 10017) | 184.81 (155.9, 218.82) | 6671 (5882, 7553) | 105.94 (88.95, 124.4) | -1.85 (-2.76, -0.93) | <0.001 |
| Puerto Rico | 2144 (1920, 2416) | 123.87 (103.9, 146.54) | 1219 (956, 1510) | 73.78 (55.61, 94.57) | -1.86 (-2.03, -1.7) | <0.001 |
| Qatar | 239 (181, 314) | 95.18 (63.56, 136.63) | 1194 (881, 1679) | 53.75 (35.24, 81.71) | -1.9 (-3.14, -0.65) | 0.003 |
| Republic of Korea | 42732 (35699, 50494) | 200.71 (155.79, 255.18) | 23390 (18964, 28792) | 75.06 (57.24, 97.13) | -3.16 (-3.39, -2.92) | <0.001 |
| Republic of Moldova | 6001 (5403, 6607) | 308.05 (265.95, 353.87) | 2457 (2089, 2841) | 112.24 (92.33, 134.38) | -3.35 (-4.18, -2.51) | <0.001 |
| Romania | 38525 (32839, 44899) | 355.56 (297.1, 421.92) | 23917 (19233, 29156) | 211.63 (168.69, 259.55) | -1.64 (-2.05, -1.23) | <0.001 |
| Russian Federation | 224383 (218357, 229873) | 343.87 (331.8, 355.18) | 114883 (104242, 124694) | 138.43 (125.39, 150.54) | -2.9 (-3.34, -2.45) | <0.001 |
| Rwanda | 1372 (949, 1829) | 70.46 (44.89, 101.96) | 2551 (1629, 3839) | 50.35 (29.21, 82) | -1.12 (-1.34, -0.89) | <0.001 |
| Saint Kitts and Nevis | 13 (12, 14) | 100.71 (85.6, 117.17) | 18 (13, 23) | 49.62 (36.5, 66.59) | -2.22 (-2.5, -1.94) | <0.001 |
| Saint Lucia | 56 (51, 61) | 123.63 (107.41, 142.24) | 92 (74, 114) | 89.06 (69.32, 111.74) | -1.15 (-1.65, -0.64) | <0.001 |
| Saint Vincent and the Grenadines | 29 (26, 32) | 84.36 (71.69, 98.09) | 51 (44, 61) | 85.18 (69.78, 103.55) | 0.02 (-0.54, 0.58) | 0.94 |
| Samoa | 25 (18, 36) | 49.84 (32.32, 75.6) | 47 (30, 69) | 56.9 (34.24, 87.04) | 0.44 (0.36, 0.51) | <0.001 |
| San Marino | 23 (18, 29) | 191.03 (139.19, 253.03) | 18 (10, 29) | 98.79 (51.25, 160.39) | -2.29 (-2.48, -2.11) | <0.001 |
| Sao Tome and Principe | 25 (18, 34) | 73.76 (47.93, 106.34) | 83 (51, 133) | 87.58 (49.66, 149.06) | 0.52 (0.13, 0.92) | 0.009 |
| Saudi Arabia | 2439 (1693, 3307) | 42.59 (26.49, 63.43) | 11554 (8024, 16476) | 42.71 (27.37, 65.18) | 0.04 (-0.11, 0.18) | 0.644 |
| Senegal | 925 (685, 1248) | 41.41 (26.88, 62.21) | 2382 (1707, 3380) | 41.5 (26.02, 66.03) | 0.07 (-0.22, 0.37) | 0.633 |
| Serbia | 18637 (14540, 24057) | 388.03 (284.13, 522.95) | 11923 (9013, 15356) | 236.26 (170.39, 315.7) | -1.48 (-1.86, -1.1) | <0.001 |
| Seychelles | 41 (34, 48) | 152.75 (110.19, 208.34) | 62 (50, 76) | 101.49 (70.46, 139.47) | -1.17 (-1.64, -0.69) | <0.001 |
| Sierra Leone | 442 (302, 636) | 33.14 (21.01, 52.08) | 1125 (701, 1660) | 34.89 (19.92, 57.05) | 0.18 (-0.03, 0.39) | 0.098 |
| Singapore | 2369 (2120, 2682) | 142.59 (121.14, 167.53) | 2124 (1832, 2458) | 56.99 (46.32, 69.96) | -2.7 (-3.65, -1.75) | <0.001 |
| Slovakia | 8675 (6849, 10671) | 345.62 (249.61, 454.63) | 4367 (3216, 5623) | 129.69 (90.6, 175.48) | -3.17 (-3.91, -2.43) | <0.001 |
| Slovenia | 2804 (2455, 3168) | 275.05 (235.44, 317.62) | 1056 (824, 1338) | 90.93 (69.97, 116.28) | -3.56 (-4.62, -2.49) | <0.001 |
| Solomon Islands | 223 (118, 346) | 207.03 (102.12, 343.14) | 750 (519, 1036) | 250.98 (163.78, 366.09) | 0.63 (0.52, 0.75) | <0.001 |
| Somalia | 1116 (675, 2058) | 44.91 (24.43, 88.13) | 2919 (1566, 6148) | 42.72 (20.59, 94.79) | -0.15 (-0.25, -0.05) | 0.004 |
| South Africa | 32464 (27510, 37904) | 240.92 (198.62, 292.86) | 41840 (36438, 47898) | 146.95 (123.63, 175.28) | -1.56 (-2.04, -1.07) | <0.001 |
| South Sudan | 803 (478, 1227) | 47.95 (27.01, 79.13) | 1853 (1234, 2742) | 53.32 (32.84, 82.36) | 0.4 (-0.02, 0.82) | 0.061 |
| Spain | 51750 (47324, 56892) | 281.5 (241.74, 328.01) | 31228 (27148, 35788) | 107.86 (91.21, 126.33) | -3.18 (-3.47, -2.89) | <0.001 |
| Sri Lanka | 6073 (4849, 7701) | 78.88 (56.91, 109.38) | 7305 (4666, 10328) | 63.55 (38.09, 94.88) | -0.64 (-1.31, 0.03) | 0.06 |
| Sudan | 4836 (3280, 7522) | 71.98 (44.39, 120.47) | 13067 (7936, 20007) | 74.33 (41.98, 118.91) | 0.1 (-0.02, 0.22) | 0.106 |
| Suriname | 168 (129, 204) | 111.35 (77.45, 150.76) | 336 (241, 449) | 115.11 (76.37, 164.73) | 0.22 (-1.22, 1.67) | 0.77 |
| Sweden | 6029 (5516, 6557) | 122.28 (106.71, 139.4) | 2647 (2299, 3034) | 51.75 (42.63, 62.28) | -2.89 (-3.32, -2.45) | <0.001 |
| Switzerland | 6989 (6288, 7728) | 177.51 (151.34, 205.86) | 3773 (3331, 4212) | 78.34 (65.37, 93.14) | -2.72 (-3.33, -2.11) | <0.001 |
| Syrian Arab Republic | 5659 (4190, 7402) | 143.25 (99.22, 196.64) | 7572 (5124, 10713) | 101.09 (65.62, 146.65) | -1.2 (-1.35, -1.05) | <0.001 |
| Taiwan (Province of China) | 17071 (15643, 18637) | 184.59 (160.12, 211.93) | 23936 (20611, 27094) | 165.79 (138.24, 195.52) | -0.43 (-0.71, -0.14) | 0.003 |
| Tajikistan | 2974 (2225, 3831) | 197.72 (144.91, 264.35) | 3545 (2229, 5647) | 80.68 (49.92, 128.04) | -2.9 (-3.09, -2.72) | <0.001 |
| Thailand | 56285 (45910, 69289) | 226.46 (167.7, 303.01) | 87288 (64098, 116587) | 225.19 (154.13, 316.29) | -0.01 (-0.45, 0.43) | 0.956 |
| Timor-Leste | 243 (154, 363) | 83.85 (50.39, 131.66) | 472 (330, 672) | 89.7 (57.56, 134.79) | 0.16 (-0.28, 0.59) | 0.482 |
| Togo | 407 (281, 568) | 37.87 (23.85, 57.15) | 1476 (824, 2262) | 43.88 (23.17, 73.69) | 0.51 (0.13, 0.89) | 0.008 |
| Tokelau | 1 (1, 1) | 138.14 (86.58, 216.62) | 1 (1, 2) | 170.57 (109.32, 258.59) | 0.77 (0.6, 0.93) | <0.001 |
| Tonga | 56 (40, 77) | 172.91 (119.49, 244.99) | 82 (53, 122) | 189.84 (116.59, 290.56) | 0.31 (0.11, 0.51) | 0.002 |
| Trinidad and Tobago | 462 (420, 508) | 93.18 (81.05, 107.1) | 616 (452, 809) | 80.78 (58.64, 107.52) | -0.48 (-1.46, 0.51) | 0.343 |
| Tunisia | 3000 (2314, 3837) | 105.88 (72.47, 148.13) | 7591 (4988, 10741) | 114.4 (68.97, 173.88) | 0.24 (0.09, 0.39) | 0.002 |
| Turkey | 96051 (69742, 127010) | 437.93 (294.95, 623.86) | 95057 (72001, 119879) | 202.45 (142.44, 273.48) | -2.57 (-2.94, -2.2) | <0.001 |
| Turkmenistan | 2023 (1846, 2199) | 176.39 (156.55, 195.99) | 2616 (1998, 3523) | 104.93 (79.54, 141.06) | -1.5 (-2.69, -0.29) | 0.015 |
| Tuvalu | 7 (4, 10) | 166.55 (101.33, 269.88) | 11 (7, 16) | 199.03 (119.94, 308.84) | 0.58 (0.52, 0.65) | <0.001 |
| Uganda | 1664 (1201, 2235) | 36.65 (24.46, 53) | 6382 (4218, 8970) | 48.79 (29.57, 73.74) | 0.92 (0.75, 1.09) | <0.001 |
| Ukraine | 100063 (89067, 111991) | 406.24 (346.36, 474.8) | 38985 (25662, 54807) | 153.36 (100.26, 222.36) | -3.22 (-3.79, -2.64) | <0.001 |
| United Arab Emirates | 1078 (705, 1580) | 112.05 (68.09, 172.2) | 4379 (3013, 6678) | 48.88 (31.66, 76.61) | -2.54 (-2.77, -2.3) | <0.001 |
| United Kingdom | 57322 (56310, 58498) | 195.49 (191.23, 200.21) | 31518 (30695, 32478) | 91.8 (88.83, 95.09) | -2.48 (-2.91, -2.06) | <0.001 |
| United Republic of Tanzania | 3401 (2530, 4828) | 46.79 (31.85, 70.37) | 10358 (7072, 14650) | 49.82 (31.95, 76.3) | 0.19 (0.03, 0.35) | 0.019 |
| United States of America | 368656 (359464, 377821) | 289.1 (280.86, 297.69) | 144612 (139856, 149881) | 89.23 (85.99, 92.78) | -3.72 (-3.9, -3.53) | <0.001 |
| United States Virgin Islands | 85 (66, 109) | 146.07 (105.37, 195.24) | 56 (39, 81) | 139.02 (91.26, 212.89) | -0.05 (-0.42, 0.31) | 0.78 |
| Uruguay | 5261 (4587, 5926) | 371.53 (312.86, 434.44) | 3507 (2903, 4212) | 201.29 (163.81, 244.45) | -2.12 (-2.39, -1.84) | <0.001 |
| Uzbekistan | 12003 (10807, 13331) | 189.18 (161.62, 217.79) | 12081 (10018, 14476) | 69.61 (54.96, 86.62) | -3.13 (-4.81, -1.42) | <0.001 |
| Vanuatu | 69 (37, 125) | 130.75 (64.38, 253.83) | 196 (117, 325) | 147.07 (81.67, 260.15) | 0.36 (0.22, 0.51) | <0.001 |
| Venezuela (Bolivarian Republic of) | 11659 (10854, 12559) | 164.69 (145.14, 184.98) | 16329 (11875, 21851) | 113.93 (81.25, 154.54) | -1.16 (-1.59, -0.74) | <0.001 |
| Viet Nam | 26377 (19193, 35119) | 132.2 (87.83, 188.96) | 86444 (62487, 122042) | 156.7 (103.79, 236.43) | 0.56 (0.48, 0.64) | <0.001 |
| Yemen | 3029 (1649, 5519) | 81.23 (42.54, 150.21) | 9563 (5227, 16290) | 75.11 (39, 132.99) | -0.31 (-0.52, -0.09) | 0.005 |
| Zambia | 1265 (1001, 1580) | 57.19 (40.04, 80.16) | 5975 (3691, 12066) | 88.05 (49.72, 186.77) | 1.4 (1.24, 1.56) | <0.001 |
| Zimbabwe | 1985 (1550, 2509) | 69.9 (49.2, 95.17) | 7198 (4832, 10510) | 121.85 (77.37, 185.31) | 1.87 (1.35, 2.4) | <0.001 |
| MOLC |  |  |  |  |  |  |
| Afghanistan | 11164 (5973, 20180) | 850.17 (435.16, 1586.92) | 14063 (8915, 20189) | 873.9 (526.64, 1292.74) | 0.08 (0, 0.17) | 0.057 |
| Albania | 9964 (8212, 12084) | 2750.74 (2236.29, 3348.8) | 14198 (10023, 19135) | 1964.77 (1385.15, 2666.57) | -1.2 (-1.73, -0.66) | <0.001 |
| Algeria | 14905 (11682, 18477) | 696.1 (501.63, 942.02) | 35736 (26819, 46075) | 562.06 (387.03, 777.5) | -0.71 (-1.01, -0.41) | <0.001 |
| American Samoa | 98 (80, 119) | 2382.06 (1830.79, 3081.83) | 191 (152, 243) | 2116.58 (1603.71, 2774.22) | -0.34 (-0.4, -0.28) | <0.001 |
| Andorra | 362 (250, 515) | 3819.99 (2557.73, 5590.28) | 466 (310, 650) | 1894.88 (1198.68, 2721.69) | -2.39 (-2.75, -2.03) | <0.001 |
| Angola | 9837 (6745, 14397) | 1318.84 (838.44, 1980.8) | 26514 (19141, 35753) | 1161.97 (780.72, 1628.15) | -0.39 (-0.7, -0.08) | 0.014 |
| Antigua and Barbuda | 69 (63, 75) | 948.24 (819.1, 1093.26) | 139 (123, 154) | 725.84 (623.82, 839.16) | -0.81 (-1.99, 0.38) | 0.182 |
| Argentina | 161593 (147760, 176793) | 3053.6 (2718.23, 3403.86) | 158978 (140392, 179522) | 1898.07 (1656.42, 2163.3) | -1.43 (-1.58, -1.28) | <0.001 |
| Armenia | 23083 (21824, 24402) | 4267.78 (4001.25, 4534.61) | 18955 (16669, 21593) | 2513.38 (2208.17, 2865.81) | -1.85 (-2.7, -0.98) | <0.001 |
| Australia | 89436 (84391, 94369) | 2967.69 (2735.4, 3213.57) | 97490 (90419, 105085) | 1566.1 (1415.11, 1722.19) | -2.16 (-2.27, -2.05) | <0.001 |
| Austria | 46651 (44371, 48761) | 2741.62 (2493.51, 2987.17) | 49006 (45817, 52534) | 2000.28 (1794.53, 2204.1) | -1.07 (-1.46, -0.68) | <0.001 |
| Azerbaijan | 26425 (21545, 31759) | 2682.04 (2182.37, 3256.88) | 32895 (22017, 45295) | 1519.33 (1016.48, 2096.24) | -1.88 (-2.29, -1.47) | <0.001 |
| Bahamas | 429 (381, 478) | 1728.26 (1484.8, 2002.96) | 902 (710, 1153) | 1183.57 (917.74, 1519.86) | -1.27 (-2.31, -0.22) | 0.018 |
| Bahrain | 914 (774, 1095) | 2795.85 (2186.47, 3539.94) | 2405 (1894, 3199) | 1173.92 (856.63, 1652.57) | -2.75 (-3.22, -2.28) | <0.001 |
| Bangladesh | 68007 (48929, 97265) | 872.56 (582.83, 1311.39) | 124704 (91681, 169333) | 533.94 (367.17, 772.69) | -1.43 (-1.81, -1.04) | <0.001 |
| Barbados | 300 (262, 338) | 817.68 (704.46, 934.5) | 586 (430, 761) | 701.3 (509.11, 924.78) | -0.17 (-0.31, -0.04) | 0.012 |
| Belarus | 88770 (81283, 95976) | 3815.61 (3427.62, 4224.75) | 65186 (49788, 81291) | 2440.02 (1857.38, 3061.33) | -1.36 (-2.04, -0.69) | <0.001 |
| Belgium | 106685 (100986, 112331) | 4500.37 (4093.72, 4898.66) | 78750 (72930, 84922) | 2540.34 (2254.63, 2837.65) | -1.99 (-2.21, -1.77) | <0.001 |
| Belize | 139 (126, 151) | 945.23 (818.32, 1092.96) | 534 (450, 625) | 1003.28 (823.03, 1195.53) | 0.35 (-0.27, 0.97) | 0.273 |
| Benin | 1846 (1476, 2285) | 577.58 (422.96, 794.67) | 4796 (3459, 6587) | 537.74 (367.9, 789.77) | -0.21 (-0.47, 0.05) | 0.115 |
| Bermuda | 329 (304, 355) | 3210.97 (2802.02, 3658.14) | 336 (278, 416) | 1677.51 (1338.19, 2097.7) | -2.4 (-2.79, -2) | <0.001 |
| Bhutan | 228 (129, 347) | 501.22 (269.01, 787.28) | 463 (307, 661) | 468.81 (288.3, 708.51) | -0.17 (-0.3, -0.04) | 0.012 |
| Bolivia (Plurinational State of) | 7330 (5417, 9656) | 1345.22 (944.62, 1818.84) | 15691 (10454, 21884) | 996.9 (655.38, 1431.42) | -0.96 (-1.02, -0.9) | <0.001 |
| Bosnia and Herzegovina | 32328 (28528, 36483) | 3902.82 (3277.47, 4623.7) | 35764 (27502, 44800) | 3613.68 (2717.35, 4615.29) | -0.16 (-0.62, 0.3) | 0.487 |
| Botswana | 1710 (1212, 2339) | 1668.54 (1092.43, 2420.81) | 3768 (2680, 5049) | 1393.49 (913.39, 1980.51) | -0.61 (-0.81, -0.42) | <0.001 |
| Brazil | 239121 (231137, 246793) | 1546.32 (1472.49, 1623.76) | 533844 (505857, 559394) | 1267.67 (1187.73, 1346.01) | -0.61 (-0.84, -0.39) | <0.001 |
| Brunei Darussalam | 483 (367, 634) | 2962.96 (2191.82, 3998.9) | 1146 (918, 1398) | 1680.12 (1291.38, 2148.36) | -1.86 (-2.39, -1.33) | <0.001 |
| Bulgaria | 72542 (64762, 80895) | 3338.79 (2973.79, 3736.61) | 73688 (61340, 86304) | 3857.55 (3199.42, 4537.02) | 0.55 (-0.03, 1.13) | 0.064 |
| Burkina Faso | 3780 (2842, 5376) | 491.44 (333.57, 752.03) | 8075 (5660, 12109) | 507 (334.46, 774.59) | 0.11 (-0.06, 0.28) | 0.205 |
| Burundi | 3060 (2170, 4261) | 792.86 (541.35, 1150.48) | 4888 (3288, 7454) | 537.96 (334.63, 861.76) | -1.24 (-1.4, -1.08) | <0.001 |
| Cabo Verde | 279 (227, 337) | 815.62 (608.79, 1064.78) | 1022 (744, 1308) | 1278.17 (876.28, 1742.93) | 1.43 (0.94, 1.92) | <0.001 |
| Cambodia | 19236 (13636, 26159) | 2380.88 (1607.44, 3397.28) | 50043 (34486, 67360) | 2159.92 (1457.71, 3033.78) | -0.31 (-0.43, -0.19) | <0.001 |
| Cameroon | 5490 (4458, 6772) | 674.3 (504.19, 889.33) | 16478 (11354, 23121) | 727.11 (453.26, 1049.96) | 0.22 (0.13, 0.32) | <0.001 |
| Canada | 219738 (208500, 230891) | 4453.13 (4148.61, 4756.93) | 202075 (188868, 215622) | 1897.01 (1722.74, 2075.19) | -2.72 (-2.83, -2.62) | <0.001 |
| Central African Republic | 2949 (1886, 5270) | 1320.1 (785.81, 2451.08) | 4924 (2940, 9265) | 1094.82 (611.24, 2156.73) | -0.6 (-0.68, -0.53) | <0.001 |
| Chad | 1920 (1402, 2743) | 422.07 (283.36, 639.23) | 6472 (4497, 9402) | 633.25 (410.48, 946.05) | 1.34 (1.21, 1.46) | <0.001 |
| Chile | 27469 (25756, 29119) | 1658.79 (1480.66, 1842.32) | 43356 (39810, 46723) | 1046.64 (918.57, 1185.32) | -1.5 (-1.71, -1.28) | <0.001 |
| China | 4531376 (3792654, 5268971) | 2964.53 (2464.26, 3481.95) | 10227882 (8035609, 12678646) | 2708.07 (2122.76, 3366.13) | -0.3 (-0.51, -0.09) | 0.006 |
| Colombia | 41123 (38524, 43743) | 1374.2 (1244.59, 1515.33) | 70106 (56693, 86178) | 769.25 (612.44, 949.19) | -2.21 (-2.32, -2.1) | <0.001 |
| Comoros | 254 (177, 350) | 707.29 (468.49, 1014.4) | 557 (359, 782) | 647.65 (403.24, 961.34) | -0.32 (-0.51, -0.12) | 0.001 |
| Congo | 3015 (1818, 3856) | 1532.04 (902.17, 2126.16) | 6537 (4693, 8758) | 1264.99 (842.24, 1790.32) | -0.57 (-0.83, -0.31) | <0.001 |
| Cook Islands | 60 (49, 75) | 2744.02 (2050.1, 3664.58) | 93 (73, 114) | 2124.73 (1562.07, 2829.78) | -0.81 (-0.95, -0.67) | <0.001 |
| Costa Rica | 2614 (2418, 2804) | 945.1 (840.43, 1057.16) | 5335 (4592, 6153) | 582.08 (487.79, 689.14) | -1.75 (-1.93, -1.56) | <0.001 |
| Côte d'Ivoire | 2988 (2360, 3759) | 398.49 (291.57, 550.14) | 7560 (5271, 11184) | 369.96 (243.49, 571.78) | -0.97 (-1.36, -0.58) | <0.001 |
| Croatia | 51932 (46797, 57393) | 4445.46 (4006.4, 4911.13) | 41257 (35861, 46710) | 3294.07 (2840.1, 3745.36) | 0.18 (-0.01, 0.36) | 0.067 |
| Cuba | 39935 (37409, 42379) | 2551.66 (2302.43, 2809.19) | 81295 (69098, 94696) | 2654.05 (2201.85, 3125.88) | -0.66 (-1.18, -0.15) | 0.012 |
| Cyprus | 2375 (1844, 2934) | 1853.26 (1368.43, 2468.1) | 4605 (3689, 5566) | 1484.37 (1141.07, 1871.36) | -2.74 (-2.88, -2.61) | <0.001 |
| Czechia | 113671 (106650, 121101) | 5240.07 (4804.71, 5699.98) | 66178 (57935, 74227) | 2290.5 (1956.42, 2656.53) | -0.25 (-0.44, -0.05) | 0.013 |
| Democratic People's Republic of Korea | 76075 (51224, 106564) | 2454.07 (1615.51, 3523.46) | 139134 (90230, 202471) | 2387 (1493.08, 3556.53) | -0.09 (-0.13, -0.05) | <0.001 |
| Democratic Republic of the Congo | 25739 (16489, 42711) | 851.6 (529.69, 1439.39) | 55848 (33587, 99386) | 785.92 (449.43, 1447.47) | -0.26 (-0.38, -0.13) | <0.001 |
| Denmark | 54292 (50640, 58025) | 5041.22 (4665.1, 5452.16) | 36532 (33733, 39499) | 2371.05 (2147.46, 2611.49) | -2.39 (-2.7, -2.08) | <0.001 |
| Djibouti | 206 (137, 290) | 793.52 (500.03, 1172.34) | 1188 (743, 1770) | 960.82 (571.33, 1514.82) | 0.62 (0.56, 0.67) | <0.001 |
| Dominica | 129 (110, 147) | 1485.72 (1181.53, 1852.86) | 234 (181, 298) | 1547.11 (1117.53, 2081.3) | 0.14 (-0.06, 0.35) | 0.162 |
| Dominican Republic | 5870 (4920, 7113) | 934.71 (714.46, 1207.86) | 19557 (13205, 26592) | 1195.27 (774.44, 1725.05) | 0.87 (-0.05, 1.8) | 0.063 |
| Ecuador | 6327 (5813, 6931) | 732.69 (658.04, 815.99) | 14131 (10665, 18382) | 533.67 (397.35, 706.3) | -1.05 (-1.97, -0.12) | 0.028 |
| Egypt | 30022 (24785, 36063) | 573.14 (445.52, 728.91) | 154333 (116932, 198909) | 1206.03 (880.94, 1617.09) | 2.52 (1.86, 3.18) | <0.001 |
| El Salvador | 3489 (2994, 4054) | 724.79 (587.45, 868.66) | 6900 (5274, 8750) | 739.72 (548.16, 987.38) | 0.24 (-0.59, 1.07) | 0.578 |
| Equatorial Guinea | 462 (284, 758) | 1266.88 (734.32, 2259.7) | 1175 (731, 1726) | 1285.37 (771.67, 1952.71) | 0.09 (-0.28, 0.47) | 0.628 |
| Eritrea | 1898 (1411, 2691) | 822.9 (578.29, 1193.16) | 4338 (3171, 5818) | 818.66 (552.5, 1154.73) | -0.04 (-0.18, 0.1) | 0.556 |
| Estonia | 15907 (14930, 16913) | 4609.25 (4194.91, 5045.91) | 7422 (6289, 8507) | 2071.59 (1718.55, 2418.97) | -2.67 (-2.84, -2.5) | <0.001 |
| Eswatini | 906 (563, 1479) | 1780.94 (1040.24, 2975.45) | 2143 (1189, 3239) | 2103.84 (1165.24, 3275) | 0.53 (0.23, 0.84) | 0.001 |
| Ethiopia | 43569 (30483, 63210) | 1201.07 (835.39, 1769.64) | 43448 (33912, 55187) | 595.78 (459.65, 763.01) | -2.25 (-2.35, -2.14) | <0.001 |
| Fiji | 607 (475, 805) | 943.22 (699.82, 1270.12) | 1188 (857, 1583) | 798.14 (555.61, 1112.91) | -0.55 (-0.78, -0.32) | <0.001 |
| Finland | 27848 (26071, 29634) | 2624.33 (2384.58, 2865.96) | 21852 (19534, 24196) | 1396.24 (1219.82, 1597.18) | -2.14 (-2.4, -1.88) | <0.001 |
| France | 389880 (360005, 418835) | 3212.88 (2919.99, 3521.63) | 486595 (440436, 533056) | 2829.15 (2521.4, 3142.63) | -0.38 (-0.52, -0.23) | <0.001 |
| Gabon | 1515 (909, 2131) | 1510.14 (877.65, 2248.89) | 2995 (2098, 3996) | 1476.47 (962.68, 2072.38) | -0.05 (-0.28, 0.19) | 0.703 |
| Gambia | 176 (132, 233) | 295.18 (203.08, 422.88) | 488 (339, 665) | 298.85 (196.57, 434.36) | 0.06 (-0.87, 1.01) | 0.892 |
| Georgia | 34269 (30489, 38310) | 2999.58 (2654.44, 3384.23) | 24203 (20671, 28338) | 2571.18 (2197.48, 3002.3) | -0.75 (-1.47, -0.02) | 0.044 |
| Germany | 628986 (589717, 669396) | 3302.36 (2992.36, 3603.94) | 605751 (562217, 655462) | 2393.23 (2158.16, 2646.25) | -1.01 (-1.15, -0.86) | <0.001 |
| Ghana | 4845 (3742, 6283) | 431.7 (293.98, 601.24) | 14470 (10255, 19003) | 480.71 (320.96, 679.42) | 0.37 (0.26, 0.49) | <0.001 |
| Greece | 83529 (78486, 89197) | 3438.28 (3103.55, 3793.25) | 89138 (83712, 94895) | 3149.16 (2855.99, 3455.56) | -0.3 (-0.57, -0.04) | 0.025 |
| Greenland | 627 (537, 717) | 10005.92 (8210.64, 11905.52) | 810 (653, 977) | 5503.17 (4224.33, 6867.54) | -1.77 (-1.98, -1.57) | <0.001 |
| Grenada | 126 (111, 141) | 1279.37 (1057.4, 1525.66) | 201 (166, 235) | 966.55 (776.5, 1191.07) | -0.91 (-2.02, 0.22) | 0.114 |
| Guam | 443 (385, 502) | 3039.63 (2434.41, 3758.34) | 873 (726, 1029) | 2417.94 (1878.79, 3022.02) | -0.81 (-1.44, -0.18) | 0.012 |
| Guatemala | 4182 (3854, 4519) | 670.52 (588.1, 762.62) | 7708 (6356, 9212) | 424.89 (341.92, 520.63) | -1.61 (-2.33, -0.89) | <0.001 |
| Guinea | 3316 (2551, 4262) | 588.77 (411.64, 801.29) | 7113 (4802, 10099) | 733.21 (465.74, 1099.61) | 0.72 (0.59, 0.84) | <0.001 |
| Guinea-Bissau | 608 (414, 864) | 867.83 (542.72, 1327.74) | 1072 (771, 1443) | 804.5 (536.37, 1157.8) | -0.23 (-0.31, -0.16) | <0.001 |
| Guyana | 475 (403, 543) | 735.18 (598.69, 877.05) | 872 (633, 1142) | 716.58 (515.45, 957.54) | 0.14 (-0.54, 0.82) | 0.689 |
| Haiti | 8217 (5169, 12726) | 1383.11 (852.05, 2248.04) | 13445 (8080, 21590) | 1010.73 (589.73, 1634.84) | -0.98 (-1.02, -0.94) | <0.001 |
| Honduras | 3891 (3139, 4808) | 1123.31 (869.86, 1439.23) | 18045 (12407, 26062) | 1672.64 (1114.44, 2471.49) | 1.33 (1, 1.67) | <0.001 |
| Hungary | 123548 (112240, 135599) | 5195.71 (4686.57, 5725.64) | 128728 (110757, 146852) | 4811.32 (4123.67, 5507.92) | -0.28 (-0.64, 0.08) | 0.123 |
| Iceland | 1181 (1105, 1268) | 2835.26 (2523.96, 3163.3) | 1560 (1374, 1755) | 1841.18 (1580.06, 2118.16) | -1.55 (-2.3, -0.79) | <0.001 |
| India | 451929 (393055, 523432) | 524.05 (450.82, 612.32) | 1244568 (1015373, 1444395) | 600.73 (486.37, 704.1) | 0.53 (0.33, 0.73) | <0.001 |
| Indonesia | 281022 (209230, 332287) | 1541.51 (1145.59, 1858.4) | 882307 (593263, 1151588) | 1878.99 (1262.54, 2473.47) | 0.63 (0.54, 0.73) | <0.001 |
| Iran (Islamic Republic of) | 45232 (36259, 54952) | 871.26 (693.48, 1062.54) | 105537 (96171, 115721) | 765.13 (688.1, 849.44) | -0.48 (-0.6, -0.36) | <0.001 |
| Iraq | 18762 (13508, 25800) | 1466.39 (989.82, 2127.65) | 63858 (43102, 83641) | 1483.72 (976.61, 2059.4) | 0.05 (-0.12, 0.22) | 0.574 |
| Ireland | 20452 (19026, 21756) | 3407.98 (3061.31, 3769.77) | 19839 (17935, 21922) | 1731.88 (1498.77, 1999.75) | -2.13 (-2.74, -1.51) | <0.001 |
| Israel | 15537 (14499, 16604) | 2144.77 (1939.56, 2355.13) | 24543 (22515, 26759) | 1438.59 (1272.33, 1614.44) | -1.25 (-1.53, -0.96) | <0.001 |
| Italy | 501039 (487565, 514323) | 3607.72 (3489.67, 3719.6) | 304894 (290175, 316858) | 1736.44 (1642.64, 1812.73) | -2.44 (-2.57, -2.31) | <0.001 |
| Jamaica | 4253 (3941, 4591) | 1668.09 (1465.31, 1891.43) | 7190 (5408, 9614) | 1431.96 (1047.33, 1946.33) | -0.55 (-1.19, 0.1) | 0.093 |
| Japan | 466243 (452672, 476743) | 1654.74 (1600.85, 1703.62) | 405642 (386360, 418209) | 1153.46 (1097.84, 1194.78) | -1.19 (-1.49, -0.88) | <0.001 |
| Jordan | 3358 (2557, 4333) | 1314.98 (933.46, 1799.42) | 12642 (9352, 16908) | 882.1 (615.95, 1249.49) | -1.22 (-1.47, -0.97) | <0.001 |
| Kazakhstan | 119599 (110261, 129570) | 5116.44 (4582.06, 5685.71) | 55893 (47573, 65297) | 1656.59 (1402.43, 1934.19) | -3.5 (-4, -3) | <0.001 |
| Kenya | 2833 (1879, 4117) | 200.69 (131.94, 292.35) | 13063 (10288, 16651) | 313.24 (243.85, 402.05) | 1.42 (1.25, 1.58) | <0.001 |
| Kiribati | 87 (70, 105) | 1313.3 (970.97, 1735.3) | 207 (150, 284) | 1445.06 (979.06, 2057.14) | 0.34 (0.29, 0.4) | <0.001 |
| Kuwait | 1415 (1261, 1589) | 1316.91 (1121.05, 1531.27) | 2971 (2382, 3664) | 526.33 (407.61, 679.9) | -2.92 (-5.18, -0.6) | 0.014 |
| Kyrgyzstan | 17119 (15400, 18942) | 3136.34 (2750.58, 3544.26) | 10405 (8490, 12460) | 1111.04 (898.48, 1343.48) | -3.43 (-4, -2.86) | <0.001 |
| Lao People's Democratic Republic | 9102 (6020, 14103) | 2444.64 (1499.4, 3969.15) | 15951 (11562, 22390) | 1885.22 (1253.19, 2778.39) | -0.82 (-0.9, -0.73) | <0.001 |
| Latvia | 25242 (23334, 27272) | 4194.36 (3802.84, 4629.94) | 12154 (10186, 13860) | 2276.54 (1866.03, 2689.93) | -2.04 (-2.85, -1.22) | <0.001 |
| Lebanon | 9200 (6558, 11799) | 2251.01 (1529.67, 3121.42) | 15237 (11804, 19363) | 1773.07 (1295.44, 2356.04) | -0.7 (-0.96, -0.44) | <0.001 |
| Lesotho | 1602 (1120, 2419) | 1128.96 (734.71, 1809.08) | 4636 (3147, 6726) | 2421.53 (1528.83, 3675.65) | 2.55 (2.34, 2.77) | <0.001 |
| Liberia | 966 (732, 1279) | 510.31 (351.33, 731.93) | 1728 (1071, 2765) | 471.03 (272.31, 784.14) | -0.34 (-0.52, -0.15) | <0.001 |
| Libya | 6715 (4878, 9019) | 2047.28 (1404.33, 2871.34) | 19978 (13894, 28125) | 2097.35 (1391.65, 3063.33) | 0.08 (-0.17, 0.32) | 0.538 |
| Lithuania | 29470 (27450, 31377) | 3837.18 (3458.32, 4238.51) | 18281 (15465, 21099) | 2285.01 (1870.31, 2695.48) | -1.73 (-2.32, -1.13) | <0.001 |
| Luxembourg | 3318 (3071, 3565) | 3953.62 (3536.62, 4395.7) | 3414 (3059, 3785) | 2137.26 (1868.07, 2418.14) | -2.05 (-2.61, -1.48) | <0.001 |
| Madagascar | 5445 (4385, 6597) | 596.82 (447.48, 794.22) | 11158 (7877, 14868) | 506.26 (339.24, 705.56) | -0.53 (-0.67, -0.38) | <0.001 |
| Malawi | 1463 (1160, 1883) | 211.09 (153.8, 294.86) | 3221 (2262, 4414) | 249.51 (163.49, 367.84) | 0.55 (0.36, 0.74) | <0.001 |
| Malaysia | 23808 (20009, 29464) | 1522.32 (1155.64, 1995.91) | 77471 (63910, 91628) | 1538.51 (1167.2, 1966.62) | 0.16 (-0.15, 0.48) | 0.313 |
| Maldives | 227 (156, 321) | 1271.88 (809.57, 1904.81) | 301 (215, 388) | 504.34 (347.69, 697.42) | -3.08 (-3.47, -2.68) | <0.001 |
| Mali | 3090 (2489, 3848) | 419.4 (308.04, 562.92) | 6882 (4907, 9454) | 432.54 (283.53, 627.64) | 0.14 (-0.01, 0.29) | 0.076 |
| Malta | 1720 (1588, 1859) | 2504.65 (2206.47, 2814.68) | 1947 (1725, 2169) | 1601.65 (1367.53, 1848.74) | -1.56 (-1.95, -1.17) | <0.001 |
| Marshall Islands | 60 (39, 90) | 2234.2 (1355.91, 3476.18) | 167 (102, 244) | 2402.36 (1404.14, 3628.31) | 0.26 (0.11, 0.4) | <0.001 |
| Mauritania | 1119 (816, 1446) | 683.34 (462.39, 948.95) | 2259 (1572, 3216) | 600.86 (390.86, 885.77) | -0.45 (-0.59, -0.32) | <0.001 |
| Mauritius | 1645 (1528, 1781) | 1270.64 (1113.22, 1441.07) | 2840 (2591, 3086) | 850.96 (736.85, 964.71) | -1.28 (-2.4, -0.14) | 0.028 |
| Mexico | 78751 (76699, 80920) | 1104.8 (1065.94, 1144.65) | 114694 (99883, 129692) | 532.52 (462.57, 605.43) | -2.47 (-2.71, -2.22) | <0.001 |
| Micronesia (Federated States of) | 201 (145, 283) | 2500.01 (1672.75, 3659.16) | 410 (275, 580) | 2684.08 (1714.92, 3942.86) | 0.22 (0.16, 0.29) | <0.001 |
| Monaco | 404 (319, 501) | 4683.17 (3578.48, 5959.6) | 612 (502, 762) | 5060.95 (4004.38, 6451.05) | 0.23 (0.06, 0.4) | 0.009 |
| Mongolia | 5727 (4430, 7454) | 3259.45 (2506.12, 4232.85) | 9293 (7203, 12004) | 2080.17 (1601.85, 2681.34) | -1.49 (-1.79, -1.19) | <0.001 |
| Montenegro | 5219 (4427, 6078) | 4649.05 (3815.73, 5567.01) | 7380 (5846, 9002) | 4508.9 (3558.66, 5650.09) | 0.14 (-0.24, 0.53) | 0.46 |
| Morocco | 30243 (24115, 38076) | 1262.34 (898.7, 1720.41) | 82487 (57760, 106920) | 1319.26 (864.43, 1840.94) | 0.15 (0.04, 0.26) | 0.008 |
| Mozambique | 4704 (3840, 5723) | 434.97 (323.83, 571.3) | 11576 (8290, 15456) | 575.18 (390.01, 810.54) | 0.91 (0.78, 1.05) | <0.001 |
| Myanmar | 94726 (64133, 140673) | 2238.99 (1446.67, 3425.92) | 144165 (102931, 195891) | 1602.52 (1090.05, 2258.84) | -1.08 (-1.15, -1) | <0.001 |
| Namibia | 637 (499, 781) | 534.47 (387.74, 712.27) | 1446 (1028, 1921) | 583.48 (396.25, 823.47) | 0.3 (0.07, 0.53) | 0.009 |
| Nauru | 31 (16, 44) | 3692.62 (1952.4, 5599.96) | 38 (21, 50) | 3597.25 (1931.81, 5159.21) | -0.09 (-0.16, -0.02) | 0.013 |
| Nepal | 10035 (6629, 14508) | 577.62 (359.45, 879.69) | 20716 (14988, 28492) | 509.19 (334.43, 746.52) | -0.4 (-0.55, -0.24) | <0.001 |
| Netherlands | 120909 (114350, 127229) | 4138.54 (3826.85, 4454.84) | 116560 (106973, 125327) | 2391.92 (2180.55, 2603.95) | -1.8 (-1.9, -1.71) | <0.001 |
| New Zealand | 19385 (18124, 20636) | 3277.59 (2968.79, 3591.87) | 21698 (20010, 23462) | 1757.44 (1564.33, 1955.37) | -2.06 (-2.27, -1.84) | <0.001 |
| Nicaragua | 1236 (1055, 1440) | 483.08 (383.62, 601.48) | 3401 (2693, 4272) | 412.77 (305.21, 547.51) | -0.54 (-0.85, -0.23) | 0.001 |
| Niger | 2025 (1426, 3109) | 401.6 (259.82, 633.82) | 5392 (3594, 9183) | 361.74 (220.77, 623.88) | -0.3 (-0.52, -0.07) | 0.009 |
| Nigeria | 9600 (6539, 13139) | 125.58 (84.28, 173.41) | 20255 (15464, 26434) | 121.39 (92.03, 157.82) | -0.13 (-0.23, -0.03) | 0.014 |
| Niue | 6 (5, 8) | 1981.71 (1392.29, 2697.4) | 9 (6, 11) | 2244.75 (1503.14, 3165.18) | 0.38 (0.25, 0.52) | <0.001 |
| North Macedonia | 11190 (9315, 13203) | 3246.47 (2622.99, 3958.6) | 18537 (13805, 23314) | 3232.09 (2415.3, 4118.88) | -0.07 (-0.34, 0.21) | 0.641 |
| Northern Mariana Islands | 118 (95, 150) | 3898.47 (2889.31, 5258.11) | 312 (265, 357) | 2913.59 (2276.15, 3669.6) | -0.92 (-1.01, -0.82) | <0.001 |
| Norway | 20172 (19437, 20830) | 2346.55 (2240.09, 2448.81) | 21535 (20333, 22591) | 1583.52 (1480.39, 1680.52) | -1.23 (-1.38, -1.08) | <0.001 |
| Oman | 679 (444, 951) | 612.98 (388.94, 915.73) | 1377 (964, 1959) | 375.6 (252.69, 545.56) | -1.58 (-2.21, -0.94) | <0.001 |
| Pakistan | 109685 (89040, 131216) | 1173.89 (907, 1484.95) | 283374 (208635, 383097) | 1292.97 (922.66, 1774.3) | 0.31 (0.2, 0.42) | <0.001 |
| Palau | 63 (48, 80) | 3873.67 (2748.84, 5352.25) | 156 (116, 200) | 3335.02 (2322.4, 4517.38) | -0.5 (-0.64, -0.36) | <0.001 |
| Palestine | 2496 (1753, 3427) | 1680.5 (1113.47, 2453.84) | 7205 (5780, 8743) | 1502.75 (1135.29, 1940.99) | -0.32 (-0.65, 0) | 0.053 |
| Panama | 2768 (2543, 3009) | 1167.06 (1019.79, 1328.31) | 4160 (3177, 5141) | 591.07 (443.39, 751.24) | -2.29 (-3.2, -1.37) | <0.001 |
| Papua New Guinea | 4800 (2867, 8271) | 1389.5 (777.26, 2418.14) | 14185 (9159, 22514) | 1489.88 (917.83, 2444.39) | 0.26 (0.2, 0.32) | <0.001 |
| Paraguay | 3268 (2581, 4033) | 902.79 (698.83, 1153.64) | 13350 (9752, 18032) | 1343.19 (942.63, 1874.56) | 1.35 (1.14, 1.57) | <0.001 |
| Peru | 23809 (19424, 28704) | 1197.53 (923.09, 1517.03) | 42882 (30421, 55821) | 791.21 (536.58, 1097.65) | -1.01 (-2.16, 0.14) | 0.086 |
| Philippines | 80189 (70731, 91366) | 1540.55 (1348.33, 1781.22) | 225318 (182021, 272614) | 1488.26 (1194.46, 1816.67) | -0.16 (-0.27, -0.05) | 0.005 |
| Poland | 383762 (375386, 393229) | 5196.79 (5042.76, 5355.01) | 412686 (375679, 447937) | 3786.15 (3435.21, 4118.83) | -1.17 (-1.37, -0.96) | <0.001 |
| Portugal | 41569 (38136, 45107) | 1861.55 (1675.02, 2064.12) | 57012 (51249, 62788) | 1894.36 (1686.86, 2104.63) | 0.07 (-0.64, 0.79) | 0.843 |
| Puerto Rico | 7632 (7081, 8300) | 1371.29 (1223.11, 1532.92) | 6434 (5261, 7747) | 713.61 (567.56, 879.27) | -2.3 (-2.74, -1.86) | <0.001 |
| Qatar | 420 (323, 531) | 1978.07 (1381.69, 2702.91) | 1790 (1252, 2583) | 846.84 (555.03, 1259.25) | -2.78 (-3.55, -2.01) | <0.001 |
| Republic of Korea | 134380 (115991, 153056) | 2447.11 (1977.41, 2952) | 191614 (162071, 224788) | 1196.54 (943.21, 1489.68) | -2.34 (-2.56, -2.11) | <0.001 |
| Republic of Moldova | 28188 (25679, 30696) | 3479.78 (3094.92, 3854.6) | 20715 (18070, 23552) | 2109.46 (1800.07, 2466.49) | -1.57 (-2.4, -0.74) | <0.001 |
| Romania | 158885 (146858, 171470) | 3133.52 (2777.25, 3494.49) | 181950 (158517, 206622) | 3528.61 (2985.07, 4136.19) | 0.39 (-0.09, 0.88) | 0.111 |
| Russian Federation | 1393898 (1370559, 1416944) | 4284.07 (4191.17, 4371.96) | 901534 (806194, 986767) | 2278.44 (2034.89, 2499.5) | -1.8 (-2.66, -0.94) | <0.001 |
| Rwanda | 4788 (3415, 6009) | 914.88 (622.54, 1249.48) | 8030 (5287, 11492) | 681.56 (428.14, 1032.35) | -0.94 (-1.09, -0.79) | <0.001 |
| Saint Kitts and Nevis | 50 (45, 55) | 977.71 (852.36, 1114.76) | 118 (95, 144) | 817.83 (642.97, 1009.12) | -0.6 (-1.23, 0.04) | 0.065 |
| Saint Lucia | 164 (151, 176) | 1208.05 (1046.75, 1380.48) | 362 (287, 450) | 878.38 (680.66, 1107.95) | -1.01 (-1.76, -0.25) | 0.009 |
| Saint Vincent and the Grenadines | 98 (87, 109) | 889.52 (769.88, 1023.03) | 193 (167, 224) | 778.36 (651.04, 925.96) | -0.42 (-1.2, 0.36) | 0.291 |
| Samoa | 128 (99, 166) | 828.01 (598.7, 1146.34) | 208 (149, 277) | 804.16 (535.98, 1131.19) | -0.1 (-0.2, 0.01) | 0.065 |
| San Marino | 150 (122, 180) | 2969.72 (2259.65, 3726.61) | 133 (75, 202) | 1402.66 (781.87, 2180.35) | -2.64 (-2.97, -2.31) | <0.001 |
| Sao Tome and Principe | 124 (96, 155) | 1100.62 (804.77, 1465.23) | 275 (198, 369) | 1365.76 (930.09, 1941.21) | 0.7 (0.39, 1) | <0.001 |
| Saudi Arabia | 5784 (4100, 7826) | 593.12 (402.53, 852.74) | 20867 (15857, 27125) | 541.42 (386.88, 749.49) | -0.28 (-0.37, -0.19) | <0.001 |
| Senegal | 3701 (2854, 4676) | 667.26 (473.7, 906.32) | 9322 (6679, 12602) | 685.15 (462.23, 979.66) | 0.07 (-0.13, 0.28) | 0.479 |
| Serbia | 98078 (78123, 122382) | 4393.49 (3473.13, 5532.95) | 109178 (85587, 135111) | 4530.36 (3522.3, 5637.21) | 0.32 (-0.35, 0.99) | 0.349 |
| Seychelles | 160 (129, 194) | 1812.59 (1349.97, 2351.29) | 240 (194, 295) | 1101.06 (833.53, 1441.14) | -1.51 (-2.1, -0.91) | <0.001 |
| Sierra Leone | 1859 (1333, 2596) | 566.13 (380.11, 819.78) | 3561 (2389, 5109) | 556.18 (355.43, 831.71) | -0.02 (-0.16, 0.12) | 0.769 |
| Singapore | 10193 (9416, 11047) | 2704.93 (2439.42, 2996.62) | 13326 (12001, 14967) | 897.62 (783.39, 1033.21) | -3.48 (-4.23, -2.73) | <0.001 |
| Slovakia | 46437 (40434, 53104) | 4716.21 (3818.03, 5711.6) | 37996 (29446, 46421) | 2574.49 (1941.27, 3252.96) | -1.98 (-2.42, -1.52) | <0.001 |
| Slovenia | 16955 (15799, 18233) | 4077.71 (3686.75, 4467.58) | 15086 (12657, 17646) | 2406.81 (2009.42, 2839.55) | -1.8 (-2.67, -0.93) | <0.001 |
| Solomon Islands | 497 (299, 756) | 1883.46 (1091.03, 2938.55) | 1218 (882, 1729) | 1930.26 (1336.99, 2782.31) | 0.09 (-0.13, 0.3) | 0.428 |
| Somalia | 2766 (1754, 4747) | 620.17 (369.65, 1119.98) | 5899 (3641, 11015) | 532.43 (302.76, 1033.82) | -0.48 (-0.55, -0.41) | <0.001 |
| South Africa | 63168 (53799, 80779) | 1807.84 (1509.19, 2310.35) | 161723 (144531, 181728) | 1962.85 (1718.19, 2253.1) | 0.12 (-0.73, 0.98) | 0.784 |
| South Sudan | 3418 (2161, 5152) | 798.88 (470.06, 1258.42) | 5489 (3744, 7862) | 750.93 (489.77, 1113.64) | -0.18 (-0.27, -0.1) | <0.001 |
| Spain | 243119 (229234, 256181) | 2881.48 (2616.05, 3160.97) | 292425 (269681, 315110) | 2336.48 (2068.66, 2624.32) | -0.73 (-0.98, -0.47) | <0.001 |
| Sri Lanka | 15014 (12652, 17863) | 798.06 (609.58, 1037.53) | 34182 (20936, 48377) | 726.28 (428.33, 1074.2) | -0.19 (-0.62, 0.23) | 0.374 |
| Sudan | 10690 (7178, 17509) | 705.84 (435.95, 1212.26) | 24808 (16246, 37098) | 727.08 (446.57, 1114.93) | 0.09 (0.05, 0.14) | <0.001 |
| Suriname | 593 (508, 673) | 1305.69 (1043.6, 1616.72) | 1422 (1071, 1844) | 1250.76 (894.58, 1699.69) | -0.08 (-0.9, 0.74) | 0.84 |
| Sweden | 34473 (32282, 36716) | 1859.81 (1710, 2024.06) | 26674 (23453, 30273) | 1035.24 (894.65, 1190.07) | -1.98 (-2.32, -1.65) | <0.001 |
| Switzerland | 39075 (36450, 41682) | 2741.78 (2467.16, 3011.47) | 37008 (34146, 39805) | 1548.71 (1386.79, 1723.56) | -1.9 (-2.16, -1.64) | <0.001 |
| Syrian Arab Republic | 10849 (8045, 14231) | 1137.06 (781.49, 1564.94) | 27340 (18465, 38329) | 1049.74 (669.69, 1556.22) | -0.36 (-0.66, -0.07) | 0.016 |
| Taiwan (Province of China) | 65174 (61726, 68353) | 2217.27 (2016.55, 2427.4) | 132597 (120180, 142272) | 1880.31 (1643.62, 2120.62) | -0.58 (-0.88, -0.28) | <0.001 |
| Tajikistan | 9562 (7794, 11592) | 1899.58 (1514.35, 2333.97) | 9289 (6270, 13431) | 778.37 (524.94, 1121.55) | -2.97 (-3.38, -2.56) | <0.001 |
| Thailand | 187677 (151937, 231709) | 2902.3 (2216.36, 3729.58) | 334521 (248939, 430838) | 1775.16 (1265.73, 2425.82) | -1.63 (-1.88, -1.38) | <0.001 |
| Timor-Leste | 640 (429, 913) | 1214.53 (786.82, 1839.32) | 1828 (1342, 2408) | 1273.2 (870.81, 1789.76) | 0.14 (-0.01, 0.28) | 0.059 |
| Togo | 1351 (1027, 1777) | 627.14 (433.96, 878.37) | 5155 (3269, 7278) | 722.63 (427.49, 1103.59) | 0.5 (0.21, 0.78) | 0.001 |
| Tokelau | 4 (3, 6) | 1818.93 (1258.35, 2506.33) | 4 (3, 6) | 1843.1 (1258.25, 2547.96) | 0.04 (-0.11, 0.18) | 0.63 |
| Tonga | 222 (181, 275) | 2236.72 (1615.87, 3043.11) | 303 (229, 380) | 2281.96 (1588.5, 3134.03) | 0.1 (-0.22, 0.41) | 0.543 |
| Trinidad and Tobago | 1402 (1290, 1511) | 1056.27 (923.94, 1211.11) | 3063 (2264, 4013) | 934.5 (678.86, 1242.37) | -0.39 (-0.53, -0.26) | <0.001 |
| Tunisia | 15666 (12288, 19339) | 1743.86 (1269.84, 2284.72) | 39332 (25236, 56265) | 1671.35 (1024.18, 2483.79) | -0.15 (-0.26, -0.04) | 0.007 |
| Turkey | 325663 (251046, 410997) | 5061.42 (3604.91, 6789.88) | 520193 (400465, 652384) | 3208.39 (2376.51, 4192.41) | -1.49 (-1.69, -1.3) | <0.001 |
| Turkmenistan | 6988 (6312, 7686) | 1973.29 (1715.61, 2229.7) | 7357 (5515, 10030) | 923.46 (691.46, 1252.47) | -2.38 (-3.36, -1.38) | <0.001 |
| Tuvalu | 26 (19, 38) | 2024.23 (1355.12, 3140.99) | 39 (30, 54) | 2063.62 (1431.94, 2988.9) | 0.07 (0.03, 0.1) | <0.001 |
| Uganda | 6319 (4800, 8127) | 567.36 (399.12, 779.29) | 15619 (10954, 21432) | 594.14 (389.4, 859.38) | 0.14 (-0.07, 0.34) | 0.194 |
| Ukraine | 550216 (510632, 589655) | 4417.92 (4006.4, 4879.68) | 241582 (163418, 337404) | 2000.38 (1329.31, 2888.48) | -2.6 (-3.36, -1.84) | <0.001 |
| United Arab Emirates | 1363 (917, 1898) | 1858.97 (1191.18, 2766.99) | 7103 (5085, 11204) | 655.11 (438.4, 1031.59) | -3.37 (-3.74, -3) | <0.001 |
| United Kingdom | 511147 (503697, 518376) | 4065.47 (3993.75, 4135.36) | 304756 (296187, 312752) | 1789.69 (1729.68, 1841.33) | -2.64 (-2.81, -2.47) | <0.001 |
| United Republic of Tanzania | 12457 (9698, 16989) | 646.15 (452.1, 924.13) | 28656 (20251, 39500) | 636.84 (409.85, 957.89) | -0.07 (-0.2, 0.06) | 0.304 |
| United States of America | 2193457 (2137779, 2243622) | 4898.5 (4753.36, 5032.68) | 1846822 (1774370, 1911656) | 2092.84 (1998.78, 2169.4) | -2.71 (-2.93, -2.5) | <0.001 |
| United States Virgin Islands | 233 (189, 288) | 1573.3 (1173.14, 2042.89) | 267 (197, 368) | 983.09 (689.74, 1405.94) | -1.61 (-2.58, -0.63) | 0.001 |
| Uruguay | 25486 (23565, 27369) | 4233.85 (3812.35, 4695.24) | 23532 (21764, 25394) | 3107.07 (2784.52, 3435.17) | -1.08 (-1.24, -0.92) | <0.001 |
| Uzbekistan | 40241 (36313, 44115) | 1970.34 (1768.66, 2181.12) | 38085 (30389, 46319) | 718.78 (575.36, 874.29) | -3.33 (-4.07, -2.6) | <0.001 |
| Vanuatu | 173 (108, 286) | 1520.39 (873.94, 2691.56) | 490 (323, 774) | 1506.16 (934.26, 2485.75) | -0.02 (-0.25, 0.2) | 0.84 |
| Venezuela (Bolivarian Republic of) | 28566 (26831, 30464) | 1773.93 (1572.82, 2001.82) | 79531 (58686, 104259) | 1514.25 (1083.67, 2042.6) | -0.68 (-1.12, -0.23) | 0.003 |
| Viet Nam | 146772 (106812, 190436) | 2100.88 (1489.06, 2794.53) | 412201 (287033, 542922) | 2191.54 (1508.47, 2956.35) | 0.15 (0.09, 0.21) | <0.001 |
| Yemen | 8251 (4838, 14408) | 926.13 (523.14, 1650.6) | 20968 (12727, 33114) | 851.93 (497.33, 1395.59) | -0.26 (-0.41, -0.1) | 0.001 |
| Zambia | 3843 (3054, 4703) | 757.05 (553.75, 1006.48) | 12456 (8632, 22728) | 1007.4 (645.36, 1850.78) | 0.93 (0.8, 1.05) | <0.001 |
| Zimbabwe | 7892 (6210, 9861) | 1094.97 (803.83, 1448.26) | 15534 (11804, 21081) | 1227.71 (865, 1711.98) | 0.39 (0.18, 0.6) | <0.001 |
| LOLC |  |  |  |  |  |  |
| Afghanistan | 3347 (1921, 6462) | 1030.75 (557.44, 2011.01) | 5114 (3365, 8106) | 1233.79 (764.61, 1997.09) | 0.58 (0.51, 0.65) | <0.001 |
| Albania | 3573 (2819, 4495) | 3490.97 (2715.8, 4476.88) | 8714 (6041, 12048) | 3182.91 (2185.25, 4415.38) | -0.33 (-0.82, 0.17) | 0.198 |
| Algeria | 6538 (4822, 8644) | 1116.95 (801.37, 1543.51) | 14717 (10474, 20431) | 882.98 (618.36, 1258.66) | -0.82 (-1.14, -0.49) | <0.001 |
| American Samoa | 37 (30, 45) | 4104.02 (3122.77, 5260.5) | 84 (67, 104) | 3727.74 (2822.88, 4852.26) | -0.22 (-0.35, -0.09) | 0.001 |
| Andorra | 185 (131, 256) | 5616.26 (3857.26, 8015) | 295 (209, 399) | 3246.63 (2194.78, 4641.76) | -1.88 (-2.3, -1.45) | <0.001 |
| Angola | 1949 (1313, 2726) | 1383.42 (880.88, 2055.7) | 6137 (4587, 7835) | 1450.76 (1022.61, 1991.59) | 0.16 (0, 0.32) | 0.054 |
| Antigua and Barbuda | 47 (43, 51) | 1311.15 (1143.17, 1492.77) | 73 (66, 81) | 1284.92 (1121.43, 1475.26) | -0.39 (-1.58, 0.82) | 0.528 |
| Argentina | 58893 (53915, 64138) | 3140.88 (2813.57, 3503.86) | 91755 (82778, 100867) | 2606.68 (2286.93, 2922.4) | -0.53 (-0.7, -0.36) | <0.001 |
| Armenia | 3112 (2848, 3375) | 2725.01 (2490.31, 2959.45) | 8248 (7348, 9257) | 3508.63 (3112.85, 3965.44) | 0.82 (0.14, 1.51) | 0.019 |
| Australia | 50868 (47509, 54029) | 4162.73 (3750.33, 4583.04) | 99453 (86721, 110541) | 3241.22 (2748.74, 3716.75) | -0.83 (-0.96, -0.69) | <0.001 |
| Austria | 25143 (23327, 26641) | 3327.96 (2998.34, 3649.24) | 36525 (32484, 40115) | 3112.94 (2723.88, 3468.34) | -0.18 (-0.48, 0.12) | 0.246 |
| Azerbaijan | 3668 (3198, 4209) | 1720.46 (1457.32, 2039.95) | 6152 (4336, 8200) | 1593.26 (1087.06, 2195.93) | -0.24 (-0.62, 0.14) | 0.22 |
| Bahamas | 139 (123, 154) | 1689.53 (1470.64, 1922.34) | 324 (264, 393) | 1665.1 (1343.31, 2055.24) | -0.02 (-0.96, 0.92) | 0.961 |
| Bahrain | 362 (301, 420) | 5963.83 (4632.05, 7600.58) | 861 (686, 1109) | 3512.65 (2578.87, 4735.26) | -1.75 (-2.19, -1.3) | <0.001 |
| Bangladesh | 16974 (11857, 24893) | 730.98 (486.54, 1112.83) | 46710 (34062, 63427) | 630.55 (438, 934.59) | -0.32 (-0.77, 0.13) | 0.158 |
| Barbados | 283 (261, 307) | 1344.05 (1191.68, 1512.3) | 396 (317, 484) | 1237.11 (976.56, 1526.92) | -0.12 (-1.02, 0.78) | 0.79 |
| Belarus | 17161 (15974, 18488) | 2612.43 (2328.5, 2922.02) | 19868 (15862, 23835) | 2148.97 (1705.97, 2629.57) | -0.6 (-1.94, 0.75) | 0.38 |
| Belgium | 58476 (53686, 62816) | 6254.4 (5603.45, 6942.24) | 64134 (55903, 71627) | 4147.73 (3556.09, 4716.69) | -1.42 (-1.83, -1) | <0.001 |
| Belize | 69 (63, 76) | 1321.18 (1148.56, 1502.87) | 194 (170, 222) | 1394.71 (1179.68, 1635.06) | 0.12 (-0.86, 1.1) | 0.817 |
| Benin | 906 (720, 1141) | 860.96 (611.83, 1180.26) | 2014 (1541, 2658) | 881.38 (611.66, 1229.71) | 0.09 (-0.08, 0.26) | 0.316 |
| Bermuda | 187 (172, 204) | 5344.02 (4562.35, 6194.93) | 275 (235, 335) | 3060.77 (2459.8, 3857.05) | -1.9 (-2.27, -1.53) | <0.001 |
| Bhutan | 48 (27, 73) | 497.84 (277.71, 788.46) | 225 (152, 321) | 695.26 (433.79, 1031.2) | 1.08 (0.94, 1.21) | <0.001 |
| Bolivia (Plurinational State of) | 3324 (2388, 4413) | 2145.29 (1457.19, 2962.09) | 9838 (6731, 14304) | 2097.03 (1367.65, 3133.2) | -0.07 (-0.14, 0) | 0.061 |
| Bosnia and Herzegovina | 5859 (4977, 6836) | 3523.38 (2941.53, 4226.11) | 15913 (12021, 21045) | 4151.53 (3104.77, 5523.13) | 0.55 (0.36, 0.75) | <0.001 |
| Botswana | 478 (349, 620) | 1913.13 (1331.86, 2662.26) | 1202 (873, 1543) | 1980.02 (1378.68, 2721.17) | 0.11 (-0.11, 0.33) | 0.332 |
| Brazil | 84889 (80266, 88677) | 1948.6 (1812.73, 2063.92) | 276625 (250309, 294512) | 1973.92 (1775.31, 2126.9) | 0.06 (-0.12, 0.24) | 0.526 |
| Brunei Darussalam | 264 (202, 343) | 5853.09 (4189.62, 8028.65) | 538 (419, 662) | 3968.64 (2932.53, 5189.73) | -1.34 (-2.04, -0.64) | <0.001 |
| Bulgaria | 14982 (13388, 16797) | 2273.17 (2010.44, 2569.45) | 32097 (26947, 37392) | 3119.69 (2603.8, 3654.18) | 1.05 (0.74, 1.36) | <0.001 |
| Burkina Faso | 1442 (1051, 2136) | 692.6 (468.51, 1067.81) | 3582 (2583, 5294) | 823.25 (556.63, 1265.46) | 0.58 (0.39, 0.76) | <0.001 |
| Burundi | 1014 (767, 1366) | 835.02 (593.21, 1181.44) | 1297 (905, 1849) | 694.69 (450.3, 1052.66) | -0.6 (-0.72, -0.48) | <0.001 |
| Cabo Verde | 234 (191, 282) | 1565.22 (1186.45, 2009.35) | 545 (405, 673) | 2693.87 (1883.37, 3598.21) | 1.8 (0.96, 2.64) | <0.001 |
| Cambodia | 5679 (4005, 7666) | 2859.38 (1876.5, 4134.42) | 18860 (13445, 24979) | 3314.3 (2221.36, 4615.46) | 0.5 (0.41, 0.59) | <0.001 |
| Cameroon | 1879 (1516, 2290) | 984.64 (718.04, 1299.92) | 5840 (4253, 7766) | 1129.95 (771.63, 1615.66) | 0.46 (0.36, 0.57) | <0.001 |
| Canada | 115079 (107509, 122168) | 5718.74 (5202.2, 6207.52) | 214010 (191904, 233954) | 4450.79 (3878.15, 5005.58) | -0.75 (-0.91, -0.59) | <0.001 |
| Central African Republic | 504 (331, 875) | 1190.16 (747.11, 2118.24) | 723 (464, 1276) | 1030.88 (637.89, 1820) | -0.48 (-0.59, -0.37) | <0.001 |
| Chad | 998 (689, 1466) | 632.26 (417.46, 962.03) | 2445 (1693, 3621) | 987.68 (650, 1493.7) | 1.45 (1.3, 1.6) | <0.001 |
| Chile | 12181 (11288, 13152) | 2225.93 (1994.55, 2462.66) | 32945 (29195, 36456) | 2133.17 (1854.49, 2398.21) | -0.21 (-0.44, 0.02) | 0.073 |
| China | 1627922 (1417580, 1828777) | 4223.46 (3631.56, 4818.46) | 6752287 (5475263, 8086029) | 5636.69 (4541.3, 6809.93) | 0.97 (0.72, 1.22) | <0.001 |
| Colombia | 17981 (16665, 19190) | 2126.65 (1899.21, 2379.28) | 51956 (43379, 61753) | 1665.05 (1330.06, 2025.92) | -0.96 (-1.36, -0.57) | <0.001 |
| Comoros | 73 (50, 103) | 838.92 (567.67, 1182.84) | 213 (138, 306) | 926.37 (578.91, 1385.14) | 0.3 (0.18, 0.42) | <0.001 |
| Congo | 800 (531, 1081) | 1675.35 (1084.31, 2357.24) | 1615 (1166, 2169) | 1614.65 (1105.89, 2274.79) | -0.11 (-0.18, -0.04) | 0.003 |
| Cook Islands | 31 (25, 39) | 5088.39 (3782.87, 6822.86) | 63 (49, 77) | 4018.97 (2919.96, 5290.14) | -0.77 (-0.87, -0.67) | <0.001 |
| Costa Rica | 1590 (1456, 1733) | 1681.23 (1483.95, 1891.59) | 3422 (2932, 3901) | 1101.52 (904.84, 1305.58) | -1.34 (-2.88, 0.21) | 0.09 |
| Côte d'Ivoire | 763 (623, 959) | 531.57 (393.07, 708.56) | 2306 (1696, 3170) | 528.71 (363.48, 747.71) | -0.32 (-0.69, 0.04) | 0.085 |
| Croatia | 12399 (11077, 13690) | 4405.84 (3926.01, 4915.88) | 23998 (21091, 26947) | 4015.59 (3447.58, 4620.01) | -0.21 (-0.62, 0.2) | 0.311 |
| Cuba | 28126 (26370, 30081) | 4469.15 (3997.86, 4968.62) | 51884 (45040, 59168) | 4226.91 (3574.36, 4925.28) | -0.32 (-0.6, -0.03) | 0.03 |
| Cyprus | 1525 (1191, 1886) | 3195.99 (2312.71, 4341.25) | 3874 (3100, 4762) | 2880.29 (2147.9, 3732.16) | -0.28 (-0.56, 0) | 0.051 |
| Czechia | 34150 (31829, 36778) | 4401.19 (4002.42, 4842.93) | 60655 (53334, 68215) | 3931.38 (3363.35, 4518.2) | 0 (-0.13, 0.13) | 0.983 |
| Democratic People's Republic of Korea | 19401 (14365, 24609) | 2867.1 (1970.35, 3913.07) | 46860 (30272, 67712) | 2674.24 (1654.97, 4048.62) | -0.23 (-0.29, -0.18) | <0.001 |
| Democratic Republic of the Congo | 6609 (4276, 11163) | 1064.84 (649.88, 1892.4) | 12188 (7515, 21396) | 918.84 (541.06, 1664.08) | -0.46 (-0.55, -0.37) | <0.001 |
| Denmark | 32600 (30178, 34988) | 5994.84 (5425.47, 6585.57) | 44500 (38537, 50181) | 5185.7 (4427.17, 5911.12) | -0.54 (-0.76, -0.33) | <0.001 |
| Djibouti | 44 (31, 60) | 949.65 (635.49, 1354.83) | 299 (195, 418) | 1293.42 (811.6, 1899.66) | 1.01 (0.91, 1.1) | <0.001 |
| Dominica | 94 (78, 112) | 2396.91 (1857.99, 3018.91) | 111 (89, 137) | 2453.69 (1836.92, 3179.39) | 0.08 (-0.09, 0.25) | 0.35 |
| Dominican Republic | 2499 (2062, 2944) | 1374.53 (1042.44, 1767.63) | 9193 (6296, 12504) | 1661.71 (1069.6, 2402.94) | 0.58 (-0.19, 1.37) | 0.141 |
| Ecuador | 3385 (3099, 3664) | 1252.74 (1113.3, 1397.11) | 11218 (9055, 13761) | 1200.53 (936.28, 1513.49) | -0.03 (-1.2, 1.15) | 0.956 |
| Egypt | 5743 (4741, 6811) | 561.99 (431.16, 721.12) | 44308 (34605, 55747) | 1893.92 (1420.79, 2500.82) | 3.94 (3.41, 4.48) | <0.001 |
| El Salvador | 1723 (1488, 2001) | 1098.76 (891.79, 1330.26) | 4508 (3610, 5730) | 1201.47 (913.59, 1586.42) | 0.27 (-0.64, 1.19) | 0.556 |
| Equatorial Guinea | 100 (67, 158) | 1216.82 (777.41, 2007.94) | 352 (234, 497) | 1794.78 (1119.17, 2660.4) | 1.31 (1.2, 1.42) | <0.001 |
| Eritrea | 218 (163, 295) | 673.46 (482.91, 937.27) | 882 (679, 1124) | 916.58 (666.85, 1212.34) | 1.01 (0.92, 1.11) | <0.001 |
| Estonia | 4099 (3841, 4368) | 3642.06 (3293.51, 4002.67) | 5921 (5004, 6682) | 3260.25 (2726.77, 3766.7) | -0.36 (-0.85, 0.14) | 0.157 |
| Eswatini | 232 (143, 371) | 1842.78 (1088.03, 2998.36) | 525 (302, 757) | 2150.28 (1205.6, 3237.73) | 0.53 (0.38, 0.67) | <0.001 |
| Ethiopia | 10526 (8085, 13958) | 1207.78 (905.79, 1647.09) | 17902 (14347, 21813) | 915.07 (707.56, 1151.85) | -0.88 (-0.96, -0.79) | <0.001 |
| Fiji | 200 (160, 246) | 1418.15 (1047.15, 1873.2) | 448 (328, 573) | 1347.69 (945.86, 1803.94) | -0.19 (-0.54, 0.17) | 0.302 |
| Finland | 16432 (15240, 17474) | 3621.83 (3265.52, 3967.65) | 27845 (24607, 30727) | 3035.17 (2622.37, 3423.82) | -0.61 (-0.89, -0.34) | <0.001 |
| France | 165174 (153926, 176604) | 3427.91 (3084.75, 3790.01) | 309639 (275637, 340617) | 3226.79 (2785.22, 3638.28) | -0.2 (-0.45, 0.05) | 0.123 |
| Gabon | 533 (342, 771) | 1807.29 (1113.68, 2763.73) | 804 (576, 1085) | 1931.68 (1276.07, 2700.03) | 0.24 (0.16, 0.33) | <0.001 |
| Gambia | 59 (45, 74) | 355.61 (248.8, 489.88) | 187 (131, 259) | 405.28 (264.15, 588.54) | 0.41 (-0.04, 0.86) | 0.074 |
| Georgia | 6679 (6004, 7356) | 2054.07 (1798.24, 2312.43) | 9930 (8705, 11249) | 2846.9 (2443.82, 3287.96) | 0.89 (-0.07, 1.85) | 0.069 |
| Germany | 282183 (261094, 300702) | 3557.26 (3214.47, 3916.98) | 443982 (392802, 488691) | 3482.75 (3047.96, 3889.55) | -0.07 (-0.28, 0.14) | 0.525 |
| Ghana | 1965 (1539, 2477) | 754.04 (524.08, 1053.57) | 6493 (4914, 8306) | 920.45 (629.56, 1279.07) | 0.65 (0.58, 0.72) | <0.001 |
| Greece | 50773 (46234, 55022) | 5322.93 (4723.84, 5898.25) | 78141 (69731, 85310) | 4832.04 (4237.64, 5357.67) | -0.31 (-0.46, -0.16) | <0.001 |
| Greenland | 170 (141, 204) | 12953.35 (10260.06, 15872.19) | 265 (203, 343) | 8505.16 (6339.58, 11085.31) | -1.22 (-1.66, -0.78) | <0.001 |
| Grenada | 71 (64, 79) | 1498.67 (1278.89, 1734.84) | 93 (80, 105) | 1497.93 (1246.35, 1766.73) | 0.17 (-1.12, 1.48) | 0.799 |
| Guam | 149 (127, 172) | 5228.61 (4252.45, 6307) | 332 (268, 399) | 2807.59 (2161.64, 3511.36) | -2.09 (-2.92, -1.25) | <0.001 |
| Guatemala | 1846 (1709, 1991) | 1268.8 (1124.61, 1415.2) | 4797 (4065, 5553) | 815.55 (679.16, 967.69) | -1.63 (-2.37, -0.89) | <0.001 |
| Guinea | 1440 (1088, 1859) | 844.81 (582.84, 1185.42) | 2854 (1969, 4070) | 1074 (677.06, 1633.77) | 0.81 (0.7, 0.92) | <0.001 |
| Guinea-Bissau | 193 (137, 269) | 1071.22 (728.64, 1562.42) | 299 (224, 399) | 1105.25 (788.33, 1535.65) | 0.11 (0.04, 0.18) | 0.003 |
| Guyana | 170 (151, 190) | 948.45 (799.82, 1111.13) | 241 (189, 300) | 816.51 (627.82, 1034.66) | -0.46 (-0.8, -0.11) | 0.01 |
| Haiti | 2290 (1577, 3527) | 1623.84 (1047.35, 2655.7) | 4221 (2660, 6741) | 1436 (853.4, 2402.16) | -0.38 (-0.44, -0.32) | <0.001 |
| Honduras | 1592 (1231, 2029) | 1609.2 (1178.53, 2180.96) | 8957 (5894, 12969) | 2777.03 (1745.62, 4189.93) | 1.83 (1.53, 2.12) | <0.001 |
| Hungary | 36687 (33373, 39879) | 4374.77 (3911.59, 4866.84) | 62194 (53251, 71562) | 4735.76 (4047.9, 5459.98) | 0.33 (0.05, 0.62) | 0.023 |
| Iceland | 752 (685, 822) | 4204.1 (3709.82, 4706.05) | 1378 (1173, 1554) | 3673.12 (3059.43, 4246.92) | -0.57 (-1.24, 0.1) | 0.095 |
| India | 96137 (81097, 112765) | 504.1 (421.06, 596.82) | 417942 (344488, 479711) | 682.33 (558.82, 789.16) | 1.05 (0.61, 1.48) | <0.001 |
| Indonesia | 76124 (56321, 94022) | 1927.94 (1417.07, 2399.46) | 298222 (197892, 381060) | 2997.33 (1963.28, 3883.8) | 1.44 (1.39, 1.49) | <0.001 |
| Iran (Islamic Republic of) | 13922 (11054, 17053) | 1396.23 (1098.46, 1744.26) | 52690 (46741, 58243) | 1444.44 (1254.5, 1620.51) | 0.04 (-0.1, 0.19) | 0.544 |
| Iraq | 7203 (5311, 9426) | 1826.44 (1252.67, 2512.04) | 27428 (19983, 35055) | 2698.55 (1868.49, 3630.64) | 1.36 (1.02, 1.7) | <0.001 |
| Ireland | 14642 (13690, 15582) | 5329.16 (4804.25, 5838.19) | 18605 (16046, 20687) | 3593.78 (3052.1, 4109.87) | -1.24 (-1.61, -0.87) | <0.001 |
| Israel | 9171 (8355, 9966) | 2998.34 (2653.63, 3346.17) | 21228 (18444, 23600) | 2581.32 (2192.18, 2952.18) | -0.47 (-0.68, -0.26) | <0.001 |
| Italy | 225780 (212071, 234208) | 4142.92 (3892.57, 4325.37) | 371390 (326948, 399109) | 3615.2 (3199.79, 3888.36) | -0.43 (-0.54, -0.32) | <0.001 |
| Jamaica | 2169 (1968, 2369) | 1882.1 (1619.71, 2165.04) | 3676 (2834, 4658) | 2108.84 (1574.03, 2766.59) | 0.79 (0.3, 1.28) | 0.002 |
| Japan | 384659 (359028, 398762) | 3852.38 (3584.06, 4020.8) | 1040968 (892489, 1116780) | 3507.14 (3056.14, 3749.71) | -0.36 (-0.41, -0.31) | <0.001 |
| Jordan | 729 (568, 938) | 1453.05 (1063.53, 1966.55) | 4367 (3321, 5778) | 1393.28 (997.19, 1920.98) | -0.09 (-0.34, 0.16) | 0.495 |
| Kazakhstan | 19338 (17886, 20915) | 3279.49 (2986.31, 3574.99) | 13979 (11923, 16206) | 1650.48 (1409.01, 1913.32) | -2.18 (-2.76, -1.59) | <0.001 |
| Kenya | 947 (607, 1377) | 246.71 (158.09, 357.26) | 4122 (3233, 5226) | 416.78 (326.18, 529.43) | 1.68 (1.47, 1.9) | <0.001 |
| Kiribati | 27 (21, 34) | 1780.32 (1250.55, 2369.07) | 53 (39, 72) | 2043.05 (1406.75, 2904.63) | 0.44 (0.33, 0.55) | <0.001 |
| Kuwait | 382 (337, 431) | 1886.36 (1576.98, 2245.48) | 1255 (1012, 1556) | 1284.95 (986.72, 1655.01) | -1.25 (-4.57, 2.18) | 0.471 |
| Kyrgyzstan | 3015 (2682, 3313) | 2199.54 (1938.15, 2462.87) | 2401 (1953, 2923) | 1247.24 (1011.51, 1523.45) | -1.95 (-2.71, -1.19) | <0.001 |
| Lao People's Democratic Republic | 2735 (1809, 4327) | 2796.49 (1769.69, 4511.31) | 5654 (4019, 7886) | 2858.6 (1946.78, 4099.37) | 0.08 (0.02, 0.14) | 0.007 |
| Latvia | 6034 (5481, 6655) | 3030.44 (2716.38, 3359.8) | 7415 (6236, 8600) | 2805.99 (2327.33, 3281.22) | -0.11 (-1.39, 1.19) | 0.872 |
| Lebanon | 3169 (2361, 4202) | 3271.26 (2332.43, 4477.15) | 12715 (9604, 16666) | 3336.45 (2437.86, 4587.4) | 0.16 (-0.17, 0.5) | 0.344 |
| Lesotho | 594 (434, 888) | 1309.13 (897.74, 1990.14) | 1197 (843, 1624) | 2232.51 (1467.77, 3213.18) | 1.78 (1.55, 2.01) | <0.001 |
| Liberia | 495 (380, 641) | 793.82 (566.07, 1119.3) | 616 (397, 926) | 761.7 (454.34, 1207.4) | -0.14 (-0.38, 0.1) | 0.253 |
| Libya | 2800 (2031, 3677) | 3246.14 (2191.25, 4587.14) | 7320 (5335, 10040) | 3339.69 (2249.76, 4821.36) | 0.12 (-0.19, 0.42) | 0.452 |
| Lithuania | 7251 (6729, 7755) | 3039.78 (2766.79, 3314.99) | 10572 (9114, 12161) | 2843.09 (2379.76, 3316.25) | -0.28 (-0.88, 0.33) | 0.376 |
| Luxembourg | 1705 (1589, 1825) | 5080.97 (4556.17, 5623.91) | 2379 (2111, 2645) | 3663.12 (3148.35, 4149.9) | -1.09 (-1.69, -0.5) | <0.001 |
| Madagascar | 1583 (1286, 1960) | 700.57 (524.6, 932.57) | 2453 (1788, 3118) | 641.77 (442.65, 873.82) | -0.29 (-0.41, -0.16) | <0.001 |
| Malawi | 451 (353, 557) | 268.75 (192.5, 363.47) | 1005 (753, 1341) | 300.29 (209.04, 422.52) | 0.4 (0.29, 0.51) | <0.001 |
| Malaysia | 9360 (7505, 11849) | 2161.16 (1581.57, 2949.35) | 33665 (27571, 40404) | 2348.28 (1757.78, 3049.11) | 0.06 (-0.27, 0.38) | 0.738 |
| Maldives | 64 (46, 89) | 2091.55 (1379.35, 3121.83) | 175 (137, 216) | 1266.39 (920.55, 1671.16) | -1.77 (-2.01, -1.53) | <0.001 |
| Mali | 977 (782, 1217) | 563.31 (422.8, 739.22) | 2479 (1850, 3258) | 641.5 (450.15, 893.14) | 0.44 (0.3, 0.57) | <0.001 |
| Malta | 895 (809, 992) | 3541.1 (3118.56, 3988.63) | 1748 (1506, 2001) | 2465.82 (2086.5, 2862.4) | -1.16 (-1.58, -0.74) | <0.001 |
| Marshall Islands | 24 (17, 35) | 3021.64 (2035.53, 4523.11) | 47 (31, 66) | 3870 (2519.23, 5644.05) | 0.76 (0.59, 0.93) | <0.001 |
| Mauritania | 507 (380, 643) | 922.06 (657.12, 1217.47) | 1122 (815, 1541) | 1089.64 (739.35, 1557.44) | 0.52 (0.4, 0.64) | <0.001 |
| Mauritius | 616 (564, 673) | 1820.15 (1618.76, 2042.11) | 1353 (1220, 1482) | 1348.84 (1183, 1527.55) | -1.36 (-2.26, -0.47) | 0.003 |
| Mexico | 43020 (41344, 44348) | 2084.66 (1983.89, 2175.93) | 75348 (67567, 83605) | 1113.41 (984.01, 1241.28) | -2.11 (-2.35, -1.86) | <0.001 |
| Micronesia (Federated States of) | 84 (63, 113) | 3417.03 (2412.58, 4824.18) | 101 (71, 140) | 3837.4 (2588.82, 5522.41) | 0.38 (0.34, 0.43) | <0.001 |
| Monaco | 294 (227, 382) | 6113.16 (4540.66, 8033.16) | 595 (470, 734) | 8477.51 (6552.44, 10747.14) | 1.06 (0.84, 1.27) | <0.001 |
| Mongolia | 1936 (1495, 2498) | 3634.08 (2755.07, 4737.97) | 2435 (1848, 3208) | 2827.72 (2119.08, 3719.18) | -0.85 (-1.3, -0.4) | <0.001 |
| Montenegro | 1328 (1047, 1642) | 4186.43 (3279.31, 5248.81) | 3328 (2517, 4289) | 5648.18 (4219.7, 7298.06) | 1.07 (0.75, 1.39) | <0.001 |
| Morocco | 11452 (9069, 14637) | 1582.92 (1115.29, 2217.22) | 30284 (22285, 37604) | 1808.04 (1213.47, 2537.51) | 0.41 (0.29, 0.52) | <0.001 |
| Mozambique | 1921 (1534, 2347) | 766.27 (558.12, 1035.18) | 4297 (3126, 5717) | 968.04 (654.02, 1371.61) | 0.78 (0.64, 0.93) | <0.001 |
| Myanmar | 27161 (19812, 38211) | 2610.85 (1780.24, 3878.35) | 55860 (43497, 72578) | 2459.27 (1751.27, 3346.11) | -0.18 (-0.23, -0.13) | <0.001 |
| Namibia | 165 (129, 204) | 573.26 (411.61, 773.56) | 420 (326, 526) | 665.94 (483.75, 905.03) | 0.5 (0.37, 0.64) | <0.001 |
| Nauru | 9 (5, 13) | 5356.64 (2930.63, 7832.89) | 12 (6, 15) | 4958.74 (2705.18, 6722.03) | -0.25 (-0.32, -0.17) | <0.001 |
| Nepal | 2379 (1511, 3577) | 617.78 (376.59, 963.95) | 8591 (5978, 12111) | 717.53 (482.82, 1057.17) | 0.49 (0.4, 0.59) | <0.001 |
| Netherlands | 76935 (72186, 81619) | 6081.64 (5536.68, 6622.57) | 114209 (101222, 125273) | 4694.4 (4047.31, 5289.66) | -0.81 (-0.95, -0.67) | <0.001 |
| New Zealand | 11416 (10520, 12326) | 4580.89 (4080.65, 5089.49) | 18695 (16521, 20595) | 3365.92 (2895.24, 3812.37) | -0.98 (-1.07, -0.89) | <0.001 |
| Nicaragua | 567 (484, 661) | 768.11 (599.62, 972.82) | 1735 (1377, 2136) | 697.8 (510.83, 918.91) | -0.37 (-0.84, 0.11) | 0.129 |
| Niger | 625 (428, 950) | 569.34 (367.01, 905.41) | 2215 (1563, 3746) | 630.15 (400.55, 1085.33) | 0.36 (0.24, 0.47) | <0.001 |
| Nigeria | 3643 (2616, 4668) | 167.13 (119.61, 215.79) | 8072 (6707, 9871) | 211.69 (170.78, 261.23) | 0.77 (0.67, 0.87) | <0.001 |
| Niue | 4 (3, 6) | 3159.93 (2242.3, 4285.43) | 5 (3, 7) | 4091.67 (2779.83, 5678.92) | 0.84 (0.73, 0.95) | <0.001 |
| North Macedonia | 2555 (2164, 3038) | 2875.72 (2312, 3530.58) | 6616 (5196, 8229) | 3355.31 (2588.69, 4220.45) | 0.54 (0.25, 0.84) | <0.001 |
| Northern Mariana Islands | 33 (28, 40) | 6686 (5135.55, 8706.95) | 120 (98, 140) | 5529.56 (4154.62, 7105.76) | -0.57 (-0.92, -0.22) | 0.001 |
| Norway | 14322 (13369, 14916) | 2976.28 (2790.23, 3130.46) | 26131 (23150, 27919) | 3833.37 (3395.76, 4132.39) | 0.91 (0.78, 1.04) | <0.001 |
| Oman | 227 (151, 319) | 740.84 (477.22, 1084.58) | 451 (337, 606) | 639.41 (449.49, 905.38) | -0.45 (-1.19, 0.29) | 0.234 |
| Pakistan | 42953 (33230, 52076) | 1471.47 (1107.67, 1882.03) | 85733 (64619, 112828) | 1641.65 (1190.47, 2221.21) | 0.36 (0.26, 0.45) | <0.001 |
| Palau | 34 (27, 43) | 6896.13 (5188.59, 9104.58) | 63 (50, 80) | 6890.86 (5078.67, 9032.64) | -0.04 (-0.16, 0.07) | 0.482 |
| Palestine | 1215 (921, 1580) | 2843.91 (1930.28, 4022.27) | 2670 (2148, 3196) | 2527.31 (1888.66, 3328.88) | -0.45 (-0.82, -0.08) | 0.017 |
| Panama | 1425 (1289, 1576) | 1762.95 (1522.06, 2025.57) | 3172 (2438, 3821) | 1226.05 (931.31, 1519.82) | -1.11 (-1.7, -0.53) | <0.001 |
| Papua New Guinea | 1428 (895, 2399) | 2196.72 (1291.01, 3757.16) | 4781 (3003, 7779) | 2613.41 (1556.39, 4348.34) | 0.6 (0.54, 0.65) | <0.001 |
| Paraguay | 1423 (1187, 1716) | 1225.08 (959.42, 1544.42) | 6616 (4945, 8877) | 2203.06 (1537.19, 3059.49) | 2.12 (1.81, 2.44) | <0.001 |
| Peru | 11998 (9897, 14349) | 2008.86 (1571.72, 2534.65) | 29864 (21414, 38295) | 1595.14 (1112.53, 2143.81) | -0.62 (-2.16, 0.95) | 0.439 |
| Philippines | 28063 (25216, 31796) | 2180.28 (1933.94, 2490.45) | 77603 (65439, 91252) | 2090.87 (1749.62, 2469.42) | -0.04 (-0.22, 0.14) | 0.674 |
| Poland | 95498 (91997, 98577) | 4012.13 (3849.96, 4155.35) | 248865 (225193, 270951) | 5421.38 (4873.86, 5908.3) | 0.9 (0.8, 1.01) | <0.001 |
| Portugal | 20212 (18470, 22036) | 2326.29 (2046.88, 2639.62) | 39625 (34197, 44190) | 2319.12 (1967.3, 2690.21) | -0.03 (-0.24, 0.18) | 0.8 |
| Puerto Rico | 5090 (4720, 5435) | 2279.18 (2026.21, 2543.23) | 6712 (5546, 7923) | 1301.1 (1046.59, 1583.67) | -2.06 (-2.41, -1.72) | <0.001 |
| Qatar | 104 (82, 130) | 4111.81 (2961.74, 5566.4) | 390 (292, 550) | 2156.21 (1480.69, 3207.97) | -2.03 (-3.14, -0.91) | <0.001 |
| Republic of Korea | 43110 (38081, 48828) | 3251.49 (2717.46, 3841.27) | 224867 (185778, 264434) | 4000.51 (3156.27, 4913.31) | 0.62 (0.29, 0.94) | <0.001 |
| Republic of Moldova | 5410 (5027, 5816) | 2531.14 (2277.21, 2790.69) | 5804 (5113, 6532) | 1731.49 (1507.34, 1997.3) | -1.37 (-2.58, -0.15) | 0.028 |
| Romania | 21835 (19878, 23903) | 1564.96 (1392.86, 1742.52) | 73268 (64671, 82767) | 2991.2 (2578.06, 3464.87) | 2.13 (1.84, 2.43) | <0.001 |
| Russian Federation | 266660 (257570, 273548) | 2757.28 (2661.67, 2838.8) | 346356 (317099, 375320) | 2451.53 (2221.25, 2667.4) | -0.37 (-1, 0.27) | 0.256 |
| Rwanda | 1182 (874, 1489) | 975.35 (685.47, 1307.47) | 2449 (1659, 3407) | 958.56 (622.91, 1397.5) | -0.06 (-0.23, 0.1) | 0.445 |
| Saint Kitts and Nevis | 40 (37, 44) | 1459.36 (1281.02, 1656.17) | 40 (33, 46) | 1401.54 (1158.4, 1667.32) | -0.08 (-0.76, 0.61) | 0.825 |
| Saint Lucia | 94 (86, 103) | 1811.47 (1582.26, 2054.7) | 190 (157, 227) | 1406.82 (1126.34, 1713.03) | -0.94 (-1.49, -0.39) | 0.001 |
| Saint Vincent and the Grenadines | 58 (53, 64) | 1315.75 (1156.87, 1489.13) | 90 (81, 103) | 1139.44 (969.04, 1334.88) | -0.48 (-1, 0.04) | 0.07 |
| Samoa | 45 (36, 54) | 1132.66 (840.63, 1503.64) | 75 (56, 96) | 1072.68 (743.56, 1467.19) | -0.18 (-0.25, -0.11) | <0.001 |
| San Marino | 114 (94, 134) | 4904.96 (3760.11, 6270.38) | 121 (81, 168) | 2431.75 (1545.82, 3545.11) | -2.52 (-2.72, -2.32) | <0.001 |
| Sao Tome and Principe | 59 (47, 72) | 1764.4 (1302.14, 2320.27) | 119 (91, 156) | 2587.01 (1807.16, 3668.49) | 1.26 (1.07, 1.45) | <0.001 |
| Saudi Arabia | 2053 (1542, 2650) | 808.56 (572.81, 1120.34) | 4813 (3811, 5976) | 891.51 (640.38, 1207.87) | 0.31 (0.17, 0.46) | <0.001 |
| Senegal | 1485 (1142, 1895) | 941.98 (656.41, 1310.39) | 4217 (3156, 5481) | 1168.1 (788.45, 1638.25) | 0.73 (0.55, 0.92) | <0.001 |
| Serbia | 18854 (15197, 23292) | 3957.49 (3033.73, 5124.02) | 45050 (35375, 55819) | 4258.41 (3231.94, 5385.82) | 0.24 (-0.12, 0.6) | 0.189 |
| Seychelles | 82 (71, 95) | 2394.75 (1849.02, 3048.14) | 113 (95, 134) | 2047.61 (1577.55, 2652.74) | -0.87 (-1.11, -0.63) | <0.001 |
| Sierra Leone | 947 (731, 1254) | 843.87 (596.44, 1193.68) | 1504 (1051, 2051) | 854.55 (558.59, 1238.79) | 0.08 (0, 0.15) | 0.047 |
| Singapore | 5332 (4938, 5770) | 4967.39 (4470.23, 5485.2) | 12636 (11152, 14141) | 2629.43 (2245.39, 3022.36) | -2 (-2.94, -1.05) | <0.001 |
| Slovakia | 12564 (10563, 14686) | 3867.53 (3082.08, 4780.2) | 18134 (14169, 22263) | 3005.59 (2245.27, 3900.04) | -0.77 (-1.17, -0.37) | <0.001 |
| Slovenia | 4344 (3964, 4704) | 3327.65 (2984.36, 3678.23) | 10833 (9262, 12194) | 3754.38 (3156.83, 4363.21) | 0.44 (0.21, 0.67) | <0.001 |
| Solomon Islands | 119 (85, 157) | 2324.64 (1575.92, 3269.13) | 305 (235, 392) | 2205.52 (1631.67, 2970.33) | -0.14 (-0.25, -0.03) | 0.011 |
| Somalia | 459 (302, 771) | 616.46 (374.54, 1075.76) | 1189 (756, 2142) | 552.39 (332.54, 1032.02) | -0.34 (-0.39, -0.29) | <0.001 |
| South Africa | 18545 (15824, 22812) | 1814.63 (1504.28, 2243.1) | 50077 (44935, 55477) | 2232.52 (1957.41, 2513.3) | 0.67 (0.04, 1.31) | 0.036 |
| South Sudan | 1244 (792, 1859) | 908.52 (561.21, 1390.24) | 1331 (949, 1798) | 930.79 (633.01, 1322.56) | 0.07 (0.02, 0.12) | 0.005 |
| Spain | 118816 (110453, 125907) | 3458.04 (3104.05, 3809.44) | 193670 (169243, 213899) | 3015.51 (2556.85, 3479) | -0.45 (-0.7, -0.21) | <0.001 |
| Sri Lanka | 5701 (4801, 6956) | 1137.93 (865.18, 1504.14) | 14226 (9366, 19608) | 914.16 (593.79, 1330.81) | -0.76 (-1.31, -0.21) | 0.007 |
| Sudan | 4540 (3070, 7472) | 922.7 (585.09, 1561.8) | 10232 (7020, 14970) | 1180.46 (757.81, 1769.72) | 0.81 (0.74, 0.88) | <0.001 |
| Suriname | 205 (175, 241) | 1703.74 (1346.02, 2135.37) | 510 (376, 676) | 1541.18 (1086.48, 2126.82) | -0.08 (-0.69, 0.53) | 0.792 |
| Sweden | 26520 (24303, 28441) | 2466.22 (2211.45, 2714.43) | 45172 (38419, 51200) | 2852.4 (2400.33, 3321.51) | 0.47 (0.14, 0.8) | 0.006 |
| Switzerland | 21947 (19966, 23631) | 3256.74 (2882.6, 3629.59) | 34249 (29665, 37592) | 2830.97 (2401.65, 3233.74) | -0.42 (-0.71, -0.12) | 0.006 |
| Syrian Arab Republic | 3348 (2485, 4237) | 1495.29 (1060.18, 2001.17) | 8953 (6357, 11788) | 1529.19 (1044.33, 2141.21) | -0.04 (-0.32, 0.24) | 0.772 |
| Taiwan (Province of China) | 26667 (25005, 28229) | 3461.64 (3131.21, 3799.25) | 95035 (84091, 103843) | 3776.57 (3238.34, 4305.38) | 0.14 (-0.12, 0.4) | 0.279 |
| Tajikistan | 1901 (1506, 2342) | 1542.18 (1202.96, 1929.36) | 1862 (1250, 2681) | 919.01 (607.67, 1323.3) | -1.73 (-2.17, -1.28) | <0.001 |
| Thailand | 64763 (52781, 77734) | 4192.23 (3164.24, 5434.82) | 209260 (160882, 259032) | 3375.22 (2441.46, 4454.77) | -0.71 (-0.99, -0.43) | <0.001 |
| Timor-Leste | 143 (99, 206) | 1645.71 (1068.03, 2459.64) | 903 (676, 1196) | 1939.83 (1358.67, 2761.92) | 0.52 (0.44, 0.61) | <0.001 |
| Togo | 473 (348, 633) | 891.26 (607.65, 1251.1) | 1535 (1003, 2209) | 1011.83 (647.88, 1494.33) | 0.41 (0.29, 0.52) | <0.001 |
| Tokelau | 2 (2, 3) | 3096.39 (2244.69, 4158.05) | 3 (2, 4) | 3247.05 (2310.46, 4362.95) | 0.16 (-0.01, 0.32) | 0.061 |
| Tonga | 109 (88, 136) | 4077.87 (3051.72, 5399.98) | 181 (140, 227) | 4159.65 (3027.8, 5516.45) | 0.09 (-0.23, 0.41) | 0.578 |
| Trinidad and Tobago | 688 (637, 739) | 1448.56 (1285, 1632.74) | 1321 (1026, 1647) | 1172.91 (899.31, 1482.66) | -0.79 (-0.93, -0.65) | <0.001 |
| Tunisia | 6622 (5320, 8216) | 2584.06 (1836.24, 3496) | 15274 (10273, 21186) | 2169.9 (1360.71, 3193.02) | -0.61 (-1, -0.22) | 0.002 |
| Turkey | 80973 (62711, 105057) | 5394.15 (3891.39, 7353.95) | 211064 (166762, 267414) | 4113.56 (3047.95, 5409.86) | -0.95 (-1.33, -0.57) | <0.001 |
| Turkmenistan | 1106 (994, 1216) | 1287.63 (1123.36, 1447.85) | 1240 (971, 1599) | 804.89 (628.79, 1035.44) | -1.42 (-2.18, -0.66) | <0.001 |
| Tuvalu | 8 (6, 12) | 2738.41 (1940.18, 4128.27) | 17 (13, 22) | 3185.23 (2302.78, 4403.31) | 0.5 (0.45, 0.54) | <0.001 |
| Uganda | 2260 (1798, 2770) | 728.75 (534.15, 964.41) | 5358 (4026, 6971) | 866.33 (598.76, 1203.63) | 0.57 (0.46, 0.69) | <0.001 |
| Ukraine | 118649 (111088, 126151) | 2970.4 (2695.86, 3241.39) | 73495 (55516, 94743) | 1549.22 (1143.76, 2029.84) | -2.14 (-2.92, -1.36) | <0.001 |
| United Arab Emirates | 298 (198, 417) | 2362.69 (1533.13, 3407.69) | 1473 (1072, 2136) | 2741.65 (1882.64, 4054.49) | 0.37 (-3.5, 4.4) | 0.853 |
| United Kingdom | 381718 (365757, 390706) | 6155.41 (5889.8, 6322.54) | 407968 (366637, 428257) | 4453.5 (4044.81, 4686.03) | -1.05 (-1.22, -0.88) | <0.001 |
| United Republic of Tanzania | 4088 (3147, 5400) | 780.29 (573.08, 1093.68) | 9608 (7096, 12799) | 851.34 (607.68, 1184.48) | 0.29 (0.2, 0.38) | <0.001 |
| United States of America | 1226709 (1143261, 1273121) | 5849.61 (5442.19, 6091.14) | 1653959 (1477184, 1752716) | 4323.01 (3851.55, 4592.36) | -0.98 (-1.15, -0.82) | <0.001 |
| United States Virgin Islands | 91 (73, 112) | 2245.49 (1679.91, 2939.4) | 207 (152, 282) | 1617.22 (1127.2, 2273.43) | -1.02 (-1.48, -0.56) | <0.001 |
| Uruguay | 10496 (9713, 11185) | 4280.55 (3819.51, 4762.73) | 14161 (12929, 15440) | 3972.94 (3508.69, 4484.54) | -0.24 (-0.42, -0.06) | 0.009 |
| Uzbekistan | 7158 (6392, 7916) | 1361.02 (1213.34, 1527.25) | 6957 (5777, 8269) | 736.61 (606.64, 880.7) | -1.81 (-2.55, -1.07) | <0.001 |
| Vanuatu | 59 (38, 98) | 2424.84 (1470.94, 4150.82) | 171 (115, 273) | 2467.97 (1566.8, 4049.28) | 0.06 (-0.04, 0.17) | 0.241 |
| Venezuela (Bolivarian Republic of) | 10431 (9663, 11221) | 2192.52 (1949.8, 2437.09) | 36747 (28471, 46248) | 2299.51 (1744.98, 2909.41) | 0.28 (-0.59, 1.15) | 0.536 |
| Viet Nam | 42711 (34451, 52712) | 2058.41 (1550.43, 2712.66) | 107163 (83384, 128289) | 2394.61 (1744.55, 3106.44) | 0.52 (0.36, 0.67) | <0.001 |
| Yemen | 2050 (1245, 3608) | 982.93 (570.99, 1756.83) | 7891 (4795, 12789) | 1293.32 (758.08, 2134.21) | 0.93 (0.8, 1.06) | <0.001 |
| Zambia | 1159 (911, 1419) | 946.55 (695.79, 1269.86) | 3427 (2572, 4817) | 1221 (843.37, 1788.02) | 0.85 (0.74, 0.96) | <0.001 |
| Zimbabwe | 3045 (2447, 3631) | 1586.46 (1184.55, 2043.66) | 4936 (3875, 6343) | 1726.84 (1274.49, 2299.21) | 0.3 (0.19, 0.42) | <0.001 |

Notes: ASDR rates are reported per 100,000 population.

Abbreviations: DALYs, disability-adjusted life-years; ASDR, age-standardized disability-adjusted life year rate; EOLC, early-onset lung cancer; MOLC, middle-onset lung cancer; LOLC, late-onset lung cancer; AAPC, average annual percentage change; UI, uncertainty interval; CI, confidence interval.

# **Supplementary Table S8.** Frontier DALYs, and effective difference of EOLC, MOLC and LOLC in 2021, by country or territory.

| Country/Territory | SDI | ASDR | Frontier DALYs | Effective difference | Effective difference rank (ASDR rank) |
| --- | --- | --- | --- | --- | --- |
| EOLC |  |  |  |  |  |
| Afghanistan | 0.34 | 105.26 | 7.42 | 97.85 | 120 (122) |
| Albania | 0.71 | 152.22 | 7.03 | 145.19 | 164 (164) |
| Algeria | 0.66 | 39.88 | 6.96 | 32.93 | 20 (18) |
| American Samoa | 0.72 | 207.64 | 6.95 | 200.68 | 187 (187) |
| Andorra | 0.87 | 124.14 | 6.97 | 117.17 | 142 (142) |
| Angola | 0.45 | 80.72 | 6.96 | 73.76 | 80 (80) |
| Antigua and Barbuda | 0.75 | 40.08 | 7.01 | 33.07 | 21 (19) |
| Argentina | 0.72 | 103.11 | 7.01 | 96.1 | 116 (116) |
| Armenia | 0.7 | 151.04 | 7.02 | 144.02 | 163 (163) |
| Australia | 0.84 | 84.38 | 6.99 | 77.39 | 88 (88) |
| Austria | 0.85 | 83.07 | 6.96 | 76.11 | 86 (86) |
| Azerbaijan | 0.69 | 134.65 | 6.95 | 127.71 | 153 (153) |
| Bahamas | 0.81 | 131.79 | 7.06 | 124.73 | 151 (151) |
| Bahrain | 0.75 | 91.53 | 6.96 | 84.57 | 103 (102) |
| Bangladesh | 0.49 | 43.56 | 6.95 | 36.61 | 28 (28) |
| Barbados | 0.75 | 54.57 | 6.96 | 47.61 | 49 (49) |
| Belarus | 0.78 | 132.39 | 7.04 | 125.35 | 152 (152) |
| Belgium | 0.85 | 130.21 | 6.95 | 123.26 | 148 (148) |
| Belize | 0.61 | 90.32 | 7.01 | 83.31 | 100 (100) |
| Benin | 0.37 | 32.11 | 7.19 | 24.91 | 14 (12) |
| Bermuda | 0.82 | 124.79 | 6.98 | 117.81 | 143 (143) |
| Bhutan | 0.47 | 42.1 | 7.05 | 35.05 | 25 (24) |
| Bolivia (Plurinational State of) | 0.6 | 79.81 | 6.95 | 72.86 | 78 (78) |
| Bosnia and Herzegovina | 0.72 | 180.15 | 6.99 | 173.16 | 175 (175) |
| Botswana | 0.64 | 102.77 | 7.04 | 95.73 | 115 (115) |
| Brazil | 0.65 | 86.62 | 7.04 | 79.57 | 92 (92) |
| Brunei Darussalam | 0.81 | 144.05 | 7.02 | 137.03 | 159 (159) |
| Bulgaria | 0.77 | 259.93 | 6.96 | 252.97 | 197 (197) |
| Burkina Faso | 0.29 | 31.24 | 15.19 | 16.05 | 10 (11) |
| Burundi | 0.29 | 41.56 | 15 | 26.56 | 16 (22) |
| Cabo Verde | 0.53 | 84.62 | 7.05 | 77.57 | 89 (89) |
| Cambodia | 0.47 | 153.42 | 6.96 | 146.46 | 166 (166) |
| Cameroon | 0.48 | 47.81 | 6.98 | 40.83 | 33 (32) |
| Canada | 0.87 | 77.49 | 7.02 | 70.47 | 73 (73) |
| Central African Republic | 0.31 | 82.59 | 7.58 | 75.01 | 85 (85) |
| Chad | 0.24 | 34.81 | 15.08 | 19.73 | 12 (16) |
| Chile | 0.77 | 69.26 | 6.95 | 62.31 | 61 (61) |
| China | 0.72 | 242.62 | 6.95 | 235.67 | 194 (194) |
| Colombia | 0.66 | 57.54 | 6.96 | 50.58 | 54 (54) |
| Comoros | 0.48 | 53.82 | 7.04 | 46.78 | 47 (47) |
| Congo | 0.58 | 95.42 | 6.96 | 88.46 | 105 (105) |
| Cook Islands | 0.78 | 184.01 | 7 | 177.01 | 179 (179) |
| Costa Rica | 0.7 | 50.13 | 6.99 | 43.14 | 38 (37) |
| Croatia | 0.8 | 154.29 | 6.96 | 147.33 | 167 (167) |
| Cuba | 0.67 | 135.66 | 7.02 | 128.64 | 154 (154) |
| Cyprus | 0.84 | 78.51 | 7.03 | 71.48 | 76 (76) |
| Czechia | 0.83 | 102.04 | 6.97 | 95.07 | 114 (114) |
| Côte d'Ivoire | #N/A | 22.79 | 7.01 | 15.78 | 9 (7) |
| Democratic People's Republic of Korea | 0.57 | 232.35 | 6.97 | 225.39 | 192 (192) |
| Democratic Republic of the Congo | 0.38 | 53.07 | 7.12 | 45.96 | 45 (44) |
| Denmark | 0.9 | 103.82 | 6.98 | 96.84 | 118 (118) |
| Djibouti | 0.49 | 68.34 | 7.04 | 61.3 | 60 (60) |
| Dominica | 0.75 | 118.38 | 6.99 | 111.4 | 138 (138) |
| Dominican Republic | 0.62 | 128.16 | 6.96 | 121.2 | 146 (145) |
| Ecuador | 0.66 | 61.44 | 7.04 | 54.39 | 56 (56) |
| Egypt | 0.61 | 114.13 | 7.05 | 107.08 | 131 (131) |
| El Salvador | 0.56 | 80.94 | 7.06 | 73.89 | 82 (82) |
| Equatorial Guinea | 0.66 | 96.35 | 6.96 | 89.39 | 106 (106) |
| Eritrea | 0.4 | 78.05 | 7.02 | 71.02 | 74 (74) |
| Estonia | 0.84 | 96.88 | 7 | 89.87 | 107 (107) |
| Eswatini | 0.59 | 193.23 | 6.99 | 186.23 | 183 (183) |
| Ethiopia | 0.36 | 50.46 | 7.26 | 43.2 | 39 (39) |
| Fiji | 0.68 | 65.04 | 7.09 | 57.95 | 59 (59) |
| Finland | 0.86 | 81.66 | 6.97 | 74.69 | 84 (84) |
| France | 0.84 | 189.87 | 6.96 | 182.92 | 182 (182) |
| Gabon | 0.63 | 104.44 | 6.95 | 97.49 | 119 (119) |
| Gambia | 0.41 | 18.38 | 7.02 | 11.36 | 5 (3) |
| Georgia | 0.73 | 184.16 | 6.97 | 177.19 | 180 (180) |
| Germany | 0.9 | 106.24 | 6.96 | 99.29 | 124 (124) |
| Ghana | 0.56 | 23.2 | 7.05 | 16.15 | 11 (8) |
| Greece | 0.79 | 144.24 | 6.95 | 137.29 | 160 (160) |
| Greenland | 0.83 | 327.9 | 6.95 | 320.96 | 201 (201) |
| Grenada | 0.67 | 77.45 | 7.04 | 70.42 | 72 (72) |
| Guam | 0.8 | 279.02 | 7 | 272.02 | 199 (199) |
| Guatemala | 0.54 | 53.93 | 7.04 | 46.89 | 48 (48) |
| Guinea | 0.34 | 53.05 | 7.35 | 45.7 | 44 (43) |
| Guinea-Bissau | 0.35 | 56.56 | 7.29 | 49.27 | 51 (51) |
| Guyana | 0.65 | 71.92 | 6.96 | 64.97 | 63 (63) |
| Haiti | 0.45 | 81.54 | 6.95 | 74.58 | 83 (83) |
| Honduras | 0.51 | 116.09 | 7.02 | 109.07 | 134 (134) |
| Hungary | 0.79 | 183.36 | 7.03 | 176.33 | 177 (177) |
| Iceland | 0.88 | 166.84 | 6.95 | 159.89 | 172 (172) |
| India | 0.58 | 59.78 | 6.97 | 52.81 | 55 (55) |
| Indonesia | 0.66 | 143.4 | 6.97 | 136.43 | 157 (157) |
| Iran (Islamic Republic of) | 0.7 | 84.12 | 6.95 | 77.17 | 87 (87) |
| Iraq | 0.66 | 89.85 | 6.95 | 82.9 | 99 (99) |
| Ireland | 0.87 | 110.4 | 7.06 | 103.35 | 128 (128) |
| Israel | 0.81 | 77.37 | 6.99 | 70.39 | 71 (71) |
| Italy | 0.81 | 91.56 | 7 | 84.55 | 102 (103) |
| Jamaica | 0.68 | 103.5 | 6.98 | 96.53 | 117 (117) |
| Japan | 0.87 | 61.53 | 7.04 | 54.49 | 57 (57) |
| Jordan | 0.73 | 74.23 | 6.96 | 67.27 | 65 (65) |
| Kazakhstan | 0.73 | 108.92 | 6.96 | 101.96 | 127 (127) |
| Kenya | 0.52 | 22.48 | 6.95 | 15.53 | 8 (6) |
| Kiribati | 0.53 | 157.92 | 6.95 | 150.96 | 169 (169) |
| Kuwait | 0.85 | 41.62 | 6.96 | 34.66 | 24 (23) |
| Kyrgyzstan | 0.6 | 88.72 | 6.97 | 81.75 | 95 (95) |
| Lao People's Democratic Republic | 0.49 | 143.71 | 7.02 | 136.68 | 158 (158) |
| Latvia | 0.83 | 117.71 | 6.95 | 110.76 | 136 (136) |
| Lebanon | 0.74 | 120.51 | 6.95 | 113.57 | 139 (139) |
| Lesotho | 0.51 | 207.7 | 7.01 | 200.69 | 188 (188) |
| Liberia | 0.35 | 31.22 | 7.27 | 23.95 | 13 (10) |
| Libya | 0.73 | 162.65 | 6.96 | 155.7 | 170 (170) |
| Lithuania | 0.86 | 131.26 | 7.04 | 124.22 | 149 (149) |
| Luxembourg | 0.88 | 100.28 | 6.99 | 93.28 | 110 (110) |
| Madagascar | 0.4 | 43.4 | 6.94 | 36.45 | 27 (27) |
| Malawi | 0.38 | 19.41 | 7.15 | 12.26 | 6 (4) |
| Malaysia | 0.74 | 116.39 | 7.04 | 109.35 | 135 (135) |
| Maldives | 0.65 | 40.63 | 6.96 | 33.67 | 22 (20) |
| Mali | 0.27 | 27.57 | 14.66 | 12.92 | 7 (9) |
| Malta | 0.8 | 104.92 | 6.95 | 97.97 | 122 (120) |
| Marshall Islands | 0.57 | 246.26 | 6.95 | 239.3 | 195 (195) |
| Mauritania | 0.5 | 34.47 | 6.95 | 27.52 | 17 (14) |
| Mauritius | 0.72 | 75.2 | 6.98 | 68.22 | 69 (69) |
| Mexico | 0.66 | 51.3 | 6.96 | 44.35 | 41 (40) |
| Micronesia (Federated States of) | 0.59 | 261.59 | 7.03 | 254.55 | 198 (198) |
| Monaco | 0.91 | 467.04 | 7.02 | 460.02 | 204 (204) |
| Mongolia | 0.62 | 171.12 | 7 | 164.13 | 174 (174) |
| Montenegro | 0.8 | 310.04 | 6.97 | 303.07 | 200 (200) |
| Morocco | 0.56 | 55.01 | 7 | 48 | 50 (50) |
| Mozambique | 0.33 | 33.52 | 7.53 | 25.99 | 15 (13) |
| Myanmar | 0.53 | 122.81 | 7 | 115.8 | 141 (141) |
| Namibia | 0.62 | 46.24 | 7.05 | 39.19 | 32 (31) |
| Nauru | 0.63 | 358.91 | 6.97 | 351.95 | 203 (203) |
| Nepal | 0.43 | 45.3 | 7.01 | 38.29 | 31 (30) |
| Netherlands | 0.89 | 127.2 | 6.97 | 120.23 | 144 (144) |
| New Zealand | 0.85 | 100.82 | 7.05 | 93.78 | 111 (111) |
| Nicaragua | 0.52 | 34.56 | 7 | 27.55 | 18 (15) |
| Niger | 0.17 | 20.2 | 18.46 | 1.74 | 3 (5) |
| Nigeria | 0.5 | 7.71 | 6.96 | 0.75 | 2 (1) |
| Niue | 0.73 | 183.87 | 6.96 | 176.91 | 178 (178) |
| North Macedonia | 0.75 | 182.55 | 7.01 | 175.54 | 176 (176) |
| Northern Mariana Islands | 0.77 | 223.03 | 7.03 | 216 | 190 (190) |
| Norway | 0.92 | 75.63 | 7.01 | 68.62 | 70 (70) |
| Oman | 0.77 | 17.97 | 6.99 | 10.98 | 4 (2) |
| Pakistan | 0.5 | 99.71 | 6.96 | 92.75 | 109 (109) |
| Palau | 0.75 | 356.72 | 6.97 | 349.75 | 202 (202) |
| Palestine | 0.63 | 108.16 | 6.98 | 101.18 | 126 (126) |
| Panama | 0.71 | 52.64 | 7.03 | 45.61 | 43 (42) |
| Papua New Guinea | 0.42 | 117.78 | 7.02 | 110.77 | 137 (137) |
| Paraguay | 0.64 | 79.35 | 6.96 | 72.4 | 77 (77) |
| Peru | 0.66 | 85.7 | 7.01 | 78.69 | 91 (91) |
| Philippines | 0.65 | 131.55 | 7.02 | 124.52 | 150 (150) |
| Poland | 0.81 | 128.22 | 7.03 | 121.19 | 145 (146) |
| Portugal | 0.74 | 105.94 | 6.96 | 98.97 | 123 (123) |
| Puerto Rico | 0.83 | 73.78 | 7.02 | 66.75 | 64 (64) |
| Qatar | 0.85 | 53.75 | 7.06 | 46.7 | 46 (46) |
| Republic of Korea | 0.89 | 75.06 | 6.95 | 68.1 | 67 (67) |
| Republic of Moldova | 0.73 | 112.24 | 6.95 | 105.29 | 129 (129) |
| Romania | 0.77 | 211.63 | 7 | 204.63 | 189 (189) |
| Russian Federation | 0.81 | 138.43 | 6.99 | 131.45 | 155 (155) |
| Rwanda | 0.44 | 50.35 | 7.03 | 43.32 | 40 (38) |
| Saint Kitts and Nevis | 0.75 | 49.62 | 7.01 | 42.62 | 36 (35) |
| Saint Lucia | 0.67 | 89.06 | 7.05 | 82.01 | 96 (96) |
| Saint Vincent and the Grenadines | 0.64 | 85.18 | 6.97 | 78.21 | 90 (90) |
| Samoa | 0.59 | 56.9 | 7 | 49.89 | 52 (52) |
| San Marino | 0.89 | 98.79 | 7 | 91.79 | 108 (108) |
| Sao Tome and Principe | 0.51 | 87.58 | 7 | 80.59 | 93 (93) |
| Saudi Arabia | 0.82 | 42.71 | 7.05 | 35.65 | 26 (25) |
| Senegal | 0.41 | 41.5 | 6.95 | 34.55 | 23 (21) |
| Serbia | 0.79 | 236.26 | 7.01 | 229.26 | 193 (193) |
| Seychelles | 0.73 | 101.49 | 7.03 | 94.45 | 113 (113) |
| Sierra Leone | 0.36 | 34.89 | 7.26 | 27.63 | 19 (17) |
| Singapore | 0.86 | 56.99 | 6.98 | 50.01 | 53 (53) |
| Slovakia | 0.81 | 129.69 | 6.95 | 122.73 | 147 (147) |
| Slovenia | 0.84 | 90.93 | 7.03 | 83.9 | 101 (101) |
| Solomon Islands | 0.43 | 250.98 | 6.97 | 244.01 | 196 (196) |
| Somalia | 0.08 | 42.72 | 42.47 | 0.25 | 1 (26) |
| South Africa | 0.68 | 146.95 | 7 | 139.95 | 161 (161) |
| South Sudan | 0.28 | 53.32 | 15.25 | 38.08 | 30 (45) |
| Spain | 0.77 | 107.86 | 6.97 | 100.89 | 125 (125) |
| Sri Lanka | 0.7 | 63.55 | 7 | 56.55 | 58 (58) |
| Sudan | 0.54 | 74.33 | 7 | 67.32 | 66 (66) |
| Suriname | 0.63 | 115.11 | 6.96 | 108.15 | 133 (133) |
| Sweden | 0.89 | 51.75 | 6.98 | 44.77 | 42 (41) |
| Switzerland | 0.93 | 78.34 | 6.94 | 71.39 | 75 (75) |
| Syrian Arab Republic | 0.62 | 101.09 | 6.98 | 94.11 | 112 (112) |
| Taiwan (Province of China) | 0.87 | 165.79 | 7.04 | 158.75 | 171 (171) |
| Tajikistan | 0.54 | 80.68 | 6.97 | 73.71 | 79 (79) |
| Thailand | 0.68 | 225.19 | 7.01 | 218.18 | 191 (191) |
| Timor-Leste | 0.44 | 89.7 | 7 | 82.7 | 98 (98) |
| Togo | 0.41 | 43.88 | 6.99 | 36.89 | 29 (29) |
| Tokelau | 0.69 | 170.57 | 6.95 | 163.62 | 173 (173) |
| Tonga | 0.63 | 189.84 | 6.95 | 182.89 | 181 (181) |
| Trinidad and Tobago | 0.77 | 80.78 | 7 | 73.78 | 81 (81) |
| Tunisia | 0.68 | 114.4 | 6.95 | 107.45 | 132 (132) |
| Turkey | #N/A | 202.45 | 6.95 | 195.5 | 186 (186) |
| Turkmenistan | 0.68 | 104.93 | 7.04 | 97.89 | 121 (121) |
| Tuvalu | 0.58 | 199.03 | 6.95 | 192.08 | 184 (184) |
| Uganda | 0.42 | 48.79 | 7.02 | 41.76 | 34 (33) |
| Ukraine | 0.76 | 153.36 | 6.95 | 146.41 | 165 (165) |
| United Arab Emirates | 0.85 | 48.88 | 7.04 | 41.84 | 35 (34) |
| United Kingdom | 0.86 | 91.8 | 6.97 | 84.84 | 104 (104) |
| United Republic of Tanzania | 0.45 | 49.82 | 7 | 42.82 | 37 (36) |
| United States Virgin Islands | 0.82 | 139.02 | 7.03 | 131.99 | 156 (156) |
| United States of America | 0.86 | 89.23 | 6.97 | 82.26 | 97 (97) |
| Uruguay | 0.72 | 201.29 | 6.97 | 194.32 | 185 (185) |
| Uzbekistan | 0.66 | 69.61 | 6.95 | 62.66 | 62 (62) |
| Vanuatu | 0.47 | 147.07 | 6.96 | 140.11 | 162 (162) |
| Venezuela (Bolivarian Republic of) | 0.6 | 113.93 | 6.95 | 106.98 | 130 (130) |
| Viet Nam | 0.63 | 156.7 | 6.99 | 149.71 | 168 (168) |
| Yemen | 0.45 | 75.11 | 6.98 | 68.14 | 68 (68) |
| Zambia | 0.51 | 88.05 | 7.01 | 81.04 | 94 (94) |
| Zimbabwe | 0.47 | 121.85 | 6.95 | 114.89 | 140 (140) |
| MOLC |  |  |  |  |  |
| Afghanistan | 0.34 | 873.9 | 124.28 | 749.62 | 68 (68) |
| Albania | 0.71 | 1964.77 | 120.64 | 1844.13 | 149 (149) |
| Algeria | 0.66 | 562.06 | 121.43 | 440.63 | 28 (27) |
| American Samoa | 0.72 | 2116.58 | 120.74 | 1995.84 | 159 (159) |
| Andorra | 0.87 | 1894.88 | 120.67 | 1774.21 | 144 (144) |
| Angola | 0.45 | 1161.97 | 121.62 | 1040.36 | 88 (88) |
| Antigua and Barbuda | 0.75 | 725.84 | 120.64 | 605.2 | 48 (47) |
| Argentina | 0.72 | 1898.07 | 120.75 | 1777.32 | 146 (146) |
| Armenia | 0.7 | 2513.38 | 121.44 | 2391.93 | 180 (180) |
| Australia | 0.84 | 1566.1 | 120.94 | 1445.16 | 123 (123) |
| Austria | 0.85 | 2000.28 | 120.93 | 1879.36 | 150 (150) |
| Azerbaijan | 0.69 | 1519.33 | 120.95 | 1398.38 | 119 (119) |
| Bahamas | 0.81 | 1183.57 | 120.74 | 1062.83 | 90 (90) |
| Bahrain | 0.75 | 1173.92 | 121.02 | 1052.9 | 89 (89) |
| Bangladesh | 0.49 | 533.94 | 120.78 | 413.16 | 23 (22) |
| Barbados | 0.75 | 701.3 | 120.75 | 580.54 | 43 (42) |
| Belarus | 0.78 | 2440.02 | 120.84 | 2319.18 | 179 (179) |
| Belgium | 0.85 | 2540.34 | 120.83 | 2419.51 | 181 (181) |
| Belize | 0.61 | 1003.28 | 120.77 | 882.51 | 78 (78) |
| Benin | 0.37 | 537.74 | 122.75 | 414.99 | 24 (23) |
| Bermuda | 0.82 | 1677.51 | 120.79 | 1556.73 | 130 (130) |
| Bhutan | 0.47 | 468.81 | 120.66 | 348.14 | 15 (11) |
| Bolivia (Plurinational State of) | 0.6 | 996.9 | 120.69 | 876.21 | 77 (77) |
| Bosnia and Herzegovina | 0.72 | 3613.68 | 120.8 | 3492.89 | 197 (197) |
| Botswana | 0.64 | 1393.49 | 120.82 | 1272.66 | 105 (105) |
| Brazil | 0.65 | 1267.67 | 120.65 | 1147.02 | 97 (97) |
| Brunei Darussalam | 0.81 | 1680.12 | 120.83 | 1559.29 | 131 (131) |
| Bulgaria | 0.77 | 3857.55 | 120.65 | 3736.9 | 199 (199) |
| Burkina Faso | 0.29 | 507 | 211.59 | 295.41 | 11 (16) |
| Burundi | 0.29 | 537.96 | 214.17 | 323.8 | 13 (24) |
| Cabo Verde | 0.53 | 1278.17 | 120.73 | 1157.44 | 99 (99) |
| Cambodia | 0.47 | 2159.92 | 120.66 | 2039.27 | 162 (162) |
| Cameroon | 0.48 | 727.11 | 120.98 | 606.13 | 50 (50) |
| Canada | 0.87 | 1897.01 | 121.3 | 1775.71 | 145 (145) |
| Central African Republic | 0.31 | 1094.82 | 126.03 | 968.79 | 84 (84) |
| Chad | 0.24 | 633.25 | 213.13 | 420.12 | 25 (36) |
| Chile | 0.77 | 1046.64 | 121.76 | 924.88 | 82 (82) |
| China | 0.72 | 2708.07 | 120.65 | 2587.42 | 186 (186) |
| Colombia | 0.66 | 769.25 | 120.74 | 648.52 | 55 (55) |
| Comoros | 0.48 | 647.65 | 120.64 | 527 | 38 (38) |
| Congo | 0.58 | 1264.99 | 120.64 | 1144.35 | 96 (96) |
| Cook Islands | 0.78 | 2124.73 | 120.89 | 2003.84 | 160 (160) |
| Costa Rica | 0.7 | 582.08 | 121.63 | 460.46 | 30 (29) |
| Croatia | 0.8 | 3294.07 | 121.26 | 3172.81 | 193 (193) |
| Cuba | 0.67 | 2654.05 | 120.73 | 2533.33 | 184 (184) |
| Cyprus | 0.84 | 1484.37 | 121.04 | 1363.33 | 113 (113) |
| Czechia | 0.83 | 2290.5 | 121.02 | 2169.48 | 169 (169) |
| Côte d'Ivoire | #N/A | 369.96 | 120.83 | 249.13 | 8 (6) |
| Democratic People's Republic of Korea | 0.57 | 2387 | 121.02 | 2265.98 | 172 (172) |
| Democratic Republic of the Congo | 0.38 | 785.92 | 122.97 | 662.95 | 58 (58) |
| Denmark | 0.9 | 2371.05 | 120.76 | 2250.29 | 171 (171) |
| Djibouti | 0.49 | 960.82 | 122.03 | 838.79 | 74 (74) |
| Dominica | 0.75 | 1547.11 | 121.14 | 1425.97 | 121 (121) |
| Dominican Republic | 0.62 | 1195.27 | 120.8 | 1074.48 | 91 (91) |
| Ecuador | 0.66 | 533.67 | 120.67 | 413 | 22 (21) |
| Egypt | 0.61 | 1206.03 | 120.77 | 1085.27 | 93 (93) |
| El Salvador | 0.56 | 739.72 | 120.89 | 618.83 | 53 (52) |
| Equatorial Guinea | 0.66 | 1285.37 | 120.8 | 1164.58 | 100 (100) |
| Eritrea | 0.4 | 818.66 | 121.26 | 697.4 | 64 (64) |
| Estonia | 0.84 | 2071.59 | 120.83 | 1950.76 | 153 (153) |
| Eswatini | 0.59 | 2103.84 | 120.64 | 1983.2 | 157 (157) |
| Ethiopia | 0.36 | 595.78 | 122.94 | 472.84 | 33 (33) |
| Fiji | 0.68 | 798.14 | 120.7 | 677.43 | 60 (60) |
| Finland | 0.86 | 1396.24 | 121.54 | 1274.7 | 106 (106) |
| France | 0.84 | 2829.15 | 120.72 | 2708.43 | 187 (187) |
| Gabon | 0.63 | 1476.47 | 120.87 | 1355.61 | 111 (111) |
| Gambia | 0.41 | 298.85 | 120.98 | 177.87 | 5 (3) |
| Georgia | 0.73 | 2571.18 | 120.95 | 2450.23 | 182 (182) |
| Germany | 0.9 | 2393.23 | 121.02 | 2272.21 | 174 (174) |
| Ghana | 0.56 | 480.71 | 120.92 | 359.79 | 16 (13) |
| Greece | 0.79 | 3149.16 | 120.72 | 3028.44 | 190 (190) |
| Greenland | 0.83 | 5503.17 | 121.02 | 5382.15 | 204 (204) |
| Grenada | 0.67 | 966.55 | 121.35 | 845.2 | 75 (75) |
| Guam | 0.8 | 2417.94 | 120.64 | 2297.3 | 177 (177) |
| Guatemala | 0.54 | 424.89 | 120.64 | 304.25 | 12 (9) |
| Guinea | 0.34 | 733.21 | 124.25 | 608.96 | 52 (51) |
| Guinea-Bissau | 0.35 | 804.5 | 123.58 | 680.92 | 61 (62) |
| Guyana | 0.65 | 716.58 | 120.64 | 595.94 | 45 (44) |
| Haiti | 0.45 | 1010.73 | 120.67 | 890.06 | 80 (80) |
| Honduras | 0.51 | 1672.64 | 120.8 | 1551.84 | 129 (129) |
| Hungary | 0.79 | 4811.32 | 121.05 | 4690.27 | 202 (202) |
| Iceland | 0.88 | 1841.18 | 121.23 | 1719.95 | 138 (138) |
| India | 0.58 | 600.73 | 120.64 | 480.09 | 36 (34) |
| Indonesia | 0.66 | 1878.99 | 120.83 | 1758.16 | 140 (140) |
| Iran (Islamic Republic of) | 0.7 | 765.13 | 120.64 | 644.49 | 54 (54) |
| Iraq | 0.66 | 1483.72 | 120.64 | 1363.07 | 112 (112) |
| Ireland | 0.87 | 1731.88 | 121.22 | 1610.67 | 132 (132) |
| Israel | 0.81 | 1438.59 | 121.05 | 1317.54 | 109 (109) |
| Italy | 0.81 | 1736.44 | 120.73 | 1615.71 | 133 (133) |
| Jamaica | 0.68 | 1431.96 | 121.2 | 1310.75 | 108 (108) |
| Japan | 0.87 | 1153.46 | 120.83 | 1032.63 | 87 (87) |
| Jordan | 0.73 | 882.1 | 120.83 | 761.27 | 70 (70) |
| Kazakhstan | 0.73 | 1656.59 | 120.71 | 1535.88 | 127 (127) |
| Kenya | 0.52 | 313.24 | 120.95 | 192.29 | 6 (4) |
| Kiribati | 0.53 | 1445.06 | 120.9 | 1324.17 | 110 (110) |
| Kuwait | 0.85 | 526.33 | 120.7 | 405.63 | 20 (18) |
| Kyrgyzstan | 0.6 | 1111.04 | 120.89 | 990.15 | 86 (86) |
| Lao People's Democratic Republic | 0.49 | 1885.22 | 120.86 | 1764.36 | 142 (142) |
| Latvia | 0.83 | 2276.54 | 120.86 | 2155.68 | 165 (165) |
| Lebanon | 0.74 | 1773.07 | 121.15 | 1651.92 | 135 (135) |
| Lesotho | 0.51 | 2421.53 | 121.2 | 2300.33 | 178 (178) |
| Liberia | 0.35 | 471.03 | 123.55 | 347.48 | 14 (12) |
| Libya | 0.73 | 2097.35 | 120.65 | 1976.71 | 156 (156) |
| Lithuania | 0.86 | 2285.01 | 120.66 | 2164.36 | 168 (168) |
| Luxembourg | 0.88 | 2137.26 | 120.64 | 2016.62 | 161 (161) |
| Madagascar | 0.4 | 506.26 | 120.64 | 385.62 | 18 (15) |
| Malawi | 0.38 | 249.51 | 122.05 | 127.46 | 4 (2) |
| Malaysia | 0.74 | 1538.51 | 120.64 | 1417.86 | 120 (120) |
| Maldives | 0.65 | 504.34 | 120.83 | 383.51 | 17 (14) |
| Mali | 0.27 | 432.54 | 214.94 | 217.6 | 7 (10) |
| Malta | 0.8 | 1601.65 | 120.95 | 1480.71 | 125 (125) |
| Marshall Islands | 0.57 | 2402.36 | 120.74 | 2281.62 | 175 (175) |
| Mauritania | 0.5 | 600.86 | 121.1 | 479.76 | 35 (35) |
| Mauritius | 0.72 | 850.96 | 120.89 | 730.07 | 66 (66) |
| Mexico | 0.66 | 532.52 | 120.73 | 411.79 | 21 (20) |
| Micronesia (Federated States of) | 0.59 | 2684.08 | 121.12 | 2562.96 | 185 (185) |
| Monaco | 0.91 | 5060.95 | 120.91 | 4940.04 | 203 (203) |
| Mongolia | 0.62 | 2080.17 | 121.26 | 1958.92 | 154 (154) |
| Montenegro | 0.8 | 4508.9 | 120.8 | 4388.1 | 200 (200) |
| Morocco | 0.56 | 1319.26 | 120.85 | 1198.41 | 102 (102) |
| Mozambique | 0.33 | 575.18 | 125.62 | 449.56 | 29 (28) |
| Myanmar | 0.53 | 1602.52 | 121.2 | 1481.31 | 126 (126) |
| Namibia | 0.62 | 583.48 | 121.03 | 462.44 | 31 (30) |
| Nauru | 0.63 | 3597.25 | 120.8 | 3476.46 | 196 (196) |
| Nepal | 0.43 | 509.19 | 121.2 | 388 | 19 (17) |
| Netherlands | 0.89 | 2391.92 | 121.29 | 2270.64 | 173 (173) |
| New Zealand | 0.85 | 1757.44 | 120.77 | 1636.68 | 134 (134) |
| Nicaragua | 0.52 | 412.77 | 120.82 | 291.95 | 10 (8) |
| Niger | 0.17 | 361.74 | 322.35 | 39.39 | 3 (5) |
| Nigeria | 0.5 | 121.39 | 120.8 | 0.6 | 2 (1) |
| Niue | 0.73 | 2244.75 | 121.21 | 2123.54 | 164 (164) |
| North Macedonia | 0.75 | 3232.09 | 121.07 | 3111.02 | 192 (192) |
| Northern Mariana Islands | 0.77 | 2913.59 | 120.74 | 2792.85 | 188 (188) |
| Norway | 0.92 | 1583.52 | 120.91 | 1462.62 | 124 (124) |
| Oman | 0.77 | 375.6 | 121.2 | 254.39 | 9 (7) |
| Pakistan | 0.5 | 1292.97 | 121.11 | 1171.86 | 101 (101) |
| Palau | 0.75 | 3335.02 | 120.83 | 3214.19 | 194 (194) |
| Palestine | 0.63 | 1502.75 | 121.05 | 1381.69 | 116 (116) |
| Panama | 0.71 | 591.07 | 120.64 | 470.43 | 32 (31) |
| Papua New Guinea | 0.42 | 1489.88 | 120.86 | 1369.02 | 115 (115) |
| Paraguay | 0.64 | 1343.19 | 120.67 | 1222.52 | 103 (103) |
| Peru | 0.66 | 791.21 | 121.05 | 670.15 | 59 (59) |
| Philippines | 0.65 | 1488.26 | 120.64 | 1367.62 | 114 (114) |
| Poland | 0.81 | 3786.15 | 120.64 | 3665.5 | 198 (198) |
| Portugal | 0.74 | 1894.36 | 121.14 | 1773.22 | 143 (143) |
| Puerto Rico | 0.83 | 713.61 | 120.71 | 592.9 | 44 (43) |
| Qatar | 0.85 | 846.84 | 120.64 | 726.2 | 65 (65) |
| Republic of Korea | 0.89 | 1196.54 | 120.73 | 1075.82 | 92 (92) |
| Republic of Moldova | 0.73 | 2109.46 | 120.7 | 1988.77 | 158 (158) |
| Romania | 0.77 | 3528.61 | 120.68 | 3407.93 | 195 (195) |
| Russian Federation | 0.81 | 2278.44 | 120.83 | 2157.61 | 166 (166) |
| Rwanda | 0.44 | 681.56 | 121.55 | 560.01 | 41 (40) |
| Saint Kitts and Nevis | 0.75 | 817.83 | 120.96 | 696.87 | 63 (63) |
| Saint Lucia | 0.67 | 878.38 | 120.76 | 757.62 | 69 (69) |
| Saint Vincent and the Grenadines | 0.64 | 778.36 | 120.65 | 657.71 | 57 (56) |
| Samoa | 0.59 | 804.16 | 120.97 | 683.18 | 62 (61) |
| San Marino | 0.89 | 1402.66 | 121.02 | 1281.64 | 107 (107) |
| Sao Tome and Principe | 0.51 | 1365.76 | 120.66 | 1245.1 | 104 (104) |
| Saudi Arabia | 0.82 | 541.42 | 121.02 | 420.4 | 26 (25) |
| Senegal | 0.41 | 685.15 | 122.02 | 563.13 | 42 (41) |
| Serbia | 0.79 | 4530.36 | 120.64 | 4409.72 | 201 (201) |
| Seychelles | 0.73 | 1101.06 | 121.1 | 979.96 | 85 (85) |
| Sierra Leone | 0.36 | 556.18 | 123.14 | 433.03 | 27 (26) |
| Singapore | 0.86 | 897.62 | 120.7 | 776.91 | 71 (71) |
| Slovakia | 0.81 | 2574.49 | 120.77 | 2453.72 | 183 (183) |
| Slovenia | 0.84 | 2406.81 | 121.14 | 2285.67 | 176 (176) |
| Solomon Islands | 0.43 | 1930.26 | 121.31 | 1808.95 | 147 (147) |
| Somalia | 0.08 | 532.43 | 532.43 | 0 | 1 (19) |
| South Africa | 0.68 | 1962.85 | 120.95 | 1841.9 | 148 (148) |
| South Sudan | 0.28 | 750.93 | 211.52 | 539.41 | 40 (53) |
| Spain | 0.77 | 2336.48 | 120.9 | 2215.58 | 170 (170) |
| Sri Lanka | 0.7 | 726.28 | 120.83 | 605.45 | 49 (48) |
| Sudan | 0.54 | 727.08 | 120.74 | 606.34 | 51 (49) |
| Suriname | 0.63 | 1250.76 | 121.2 | 1129.56 | 95 (95) |
| Sweden | 0.89 | 1035.24 | 120.64 | 914.6 | 81 (81) |
| Switzerland | 0.93 | 1548.71 | 121.33 | 1427.38 | 122 (122) |
| Syrian Arab Republic | 0.62 | 1049.74 | 120.88 | 928.86 | 83 (83) |
| Taiwan (Province of China) | 0.87 | 1880.31 | 121.43 | 1758.88 | 141 (141) |
| Tajikistan | 0.54 | 778.37 | 121.27 | 657.1 | 56 (57) |
| Thailand | 0.68 | 1775.16 | 121.28 | 1653.89 | 136 (136) |
| Timor-Leste | 0.44 | 1273.2 | 121.09 | 1152.11 | 98 (98) |
| Togo | 0.41 | 722.63 | 121.37 | 601.26 | 47 (46) |
| Tokelau | 0.69 | 1843.1 | 120.97 | 1722.13 | 139 (139) |
| Tonga | 0.63 | 2281.96 | 120.92 | 2161.04 | 167 (167) |
| Trinidad and Tobago | 0.77 | 934.5 | 120.64 | 813.86 | 73 (73) |
| Tunisia | 0.68 | 1671.35 | 120.86 | 1550.49 | 128 (128) |
| Turkey | #N/A | 3208.39 | 120.85 | 3087.54 | 191 (191) |
| Turkmenistan | 0.68 | 923.46 | 120.91 | 802.56 | 72 (72) |
| Tuvalu | 0.58 | 2063.62 | 120.89 | 1942.73 | 152 (152) |
| Uganda | 0.42 | 594.14 | 120.64 | 473.49 | 34 (32) |
| Ukraine | 0.76 | 2000.38 | 120.64 | 1879.74 | 151 (151) |
| United Arab Emirates | 0.85 | 655.11 | 121.1 | 534.01 | 39 (39) |
| United Kingdom | 0.86 | 1789.69 | 120.64 | 1669.05 | 137 (137) |
| United Republic of Tanzania | 0.45 | 636.84 | 120.66 | 516.18 | 37 (37) |
| United States Virgin Islands | 0.82 | 983.09 | 120.65 | 862.44 | 76 (76) |
| United States of America | 0.86 | 2092.84 | 120.9 | 1971.94 | 155 (155) |
| Uruguay | 0.72 | 3107.07 | 121.14 | 2985.92 | 189 (189) |
| Uzbekistan | 0.66 | 718.78 | 120.76 | 598.01 | 46 (45) |
| Vanuatu | 0.47 | 1506.16 | 120.64 | 1385.52 | 117 (117) |
| Venezuela (Bolivarian Republic of) | 0.6 | 1514.25 | 120.85 | 1393.4 | 118 (118) |
| Viet Nam | 0.63 | 2191.54 | 120.89 | 2070.65 | 163 (163) |
| Yemen | 0.45 | 851.93 | 120.65 | 731.28 | 67 (67) |
| Zambia | 0.51 | 1007.4 | 120.74 | 886.65 | 79 (79) |
| Zimbabwe | 0.47 | 1227.71 | 120.98 | 1106.73 | 94 (94) |
| LOLC |  |  |  |  |  |
| Afghanistan | 0.34 | 1233.79 | 167.17 | 1066.63 | 58 (58) |
| Albania | 0.71 | 3182.91 | 167.11 | 3015.79 | 150 (150) |
| Algeria | 0.66 | 882.98 | 167.13 | 715.85 | 30 (28) |
| American Samoa | 0.72 | 3727.74 | 167.12 | 3560.61 | 171 (171) |
| Andorra | 0.87 | 3246.63 | 167.4 | 3079.23 | 154 (154) |
| Angola | 0.45 | 1450.76 | 167.53 | 1283.23 | 75 (75) |
| Antigua and Barbuda | 0.75 | 1284.92 | 167.24 | 1117.69 | 62 (62) |
| Argentina | 0.72 | 2606.68 | 167.4 | 2439.28 | 125 (125) |
| Armenia | 0.7 | 3508.63 | 167.21 | 3341.43 | 165 (165) |
| Australia | 0.84 | 3241.22 | 167.41 | 3073.81 | 153 (153) |
| Austria | 0.85 | 3112.94 | 167.12 | 2945.82 | 148 (148) |
| Azerbaijan | 0.69 | 1593.26 | 167.13 | 1426.14 | 80 (80) |
| Bahamas | 0.81 | 1665.1 | 167.17 | 1497.94 | 88 (88) |
| Bahrain | 0.75 | 3512.65 | 167.12 | 3345.53 | 166 (166) |
| Bangladesh | 0.49 | 630.55 | 167.53 | 463.02 | 10 (8) |
| Barbados | 0.75 | 1237.11 | 167.21 | 1069.89 | 59 (59) |
| Belarus | 0.78 | 2148.97 | 167.49 | 1981.48 | 104 (104) |
| Belgium | 0.85 | 4147.73 | 167.11 | 3980.61 | 185 (185) |
| Belize | 0.61 | 1394.71 | 167.54 | 1227.17 | 70 (70) |
| Benin | 0.37 | 881.38 | 167.15 | 714.23 | 29 (27) |
| Bermuda | 0.82 | 3060.77 | 167.39 | 2893.38 | 147 (147) |
| Bhutan | 0.47 | 695.26 | 167.61 | 527.65 | 15 (15) |
| Bolivia (Plurinational State of) | 0.6 | 2097.03 | 167.39 | 1929.64 | 101 (101) |
| Bosnia and Herzegovina | 0.72 | 4151.53 | 167.26 | 3984.27 | 186 (186) |
| Botswana | 0.64 | 1980.02 | 167.16 | 1812.85 | 97 (97) |
| Brazil | 0.65 | 1973.92 | 167.27 | 1806.65 | 96 (96) |
| Brunei Darussalam | 0.81 | 3968.64 | 167.46 | 3801.17 | 178 (178) |
| Bulgaria | 0.77 | 3119.69 | 167.18 | 2952.52 | 149 (149) |
| Burkina Faso | 0.29 | 823.25 | 269.58 | 553.67 | 18 (23) |
| Burundi | 0.29 | 694.69 | 269.46 | 425.23 | 9 (14) |
| Cabo Verde | 0.53 | 2693.87 | 167.49 | 2526.38 | 129 (129) |
| Cambodia | 0.47 | 3314.3 | 167.2 | 3147.11 | 157 (157) |
| Cameroon | 0.48 | 1129.95 | 167.12 | 962.83 | 49 (49) |
| Canada | 0.87 | 4450.79 | 167.12 | 4283.67 | 191 (191) |
| Central African Republic | 0.31 | 1030.88 | 188.58 | 842.3 | 41 (42) |
| Chad | 0.24 | 987.68 | 275.28 | 712.39 | 28 (40) |
| Chile | 0.77 | 2133.17 | 167.14 | 1966.03 | 103 (103) |
| China | 0.72 | 5636.69 | 167.28 | 5469.41 | 200 (200) |
| Colombia | 0.66 | 1665.05 | 167.2 | 1497.85 | 87 (87) |
| Comoros | 0.48 | 926.37 | 167.18 | 759.19 | 38 (36) |
| Congo | 0.58 | 1614.65 | 167.13 | 1447.52 | 82 (82) |
| Cook Islands | 0.78 | 4018.97 | 167.12 | 3851.85 | 182 (182) |
| Costa Rica | 0.7 | 1101.52 | 167.13 | 934.39 | 46 (46) |
| Croatia | 0.8 | 4015.59 | 167.2 | 3848.39 | 181 (181) |
| Cuba | 0.67 | 4226.91 | 167.17 | 4059.74 | 188 (188) |
| Cyprus | 0.84 | 2880.29 | 167.12 | 2713.16 | 141 (141) |
| Czechia | 0.83 | 3931.38 | 167.49 | 3763.89 | 177 (177) |
| Côte d'Ivoire | #N/A | 528.71 | 167.14 | 361.57 | 7 (5) |
| Democratic People's Republic of Korea | 0.57 | 2674.24 | 167.42 | 2506.83 | 128 (128) |
| Democratic Republic of the Congo | 0.38 | 918.84 | 167.28 | 751.57 | 35 (33) |
| Denmark | 0.9 | 5185.7 | 167.18 | 5018.52 | 197 (197) |
| Djibouti | 0.49 | 1293.42 | 167.13 | 1126.29 | 65 (65) |
| Dominica | 0.75 | 2453.69 | 167.12 | 2286.57 | 118 (118) |
| Dominican Republic | 0.62 | 1661.71 | 167.13 | 1494.58 | 86 (86) |
| Ecuador | 0.66 | 1200.53 | 167.16 | 1033.37 | 54 (54) |
| Egypt | 0.61 | 1893.92 | 167.12 | 1726.8 | 93 (93) |
| El Salvador | 0.56 | 1201.47 | 167.26 | 1034.21 | 55 (55) |
| Equatorial Guinea | 0.66 | 1794.78 | 167.26 | 1627.53 | 91 (91) |
| Eritrea | 0.4 | 916.58 | 167.12 | 749.45 | 34 (32) |
| Estonia | 0.84 | 3260.25 | 167.46 | 3092.8 | 156 (156) |
| Eswatini | 0.59 | 2150.28 | 167.12 | 1983.16 | 105 (105) |
| Ethiopia | 0.36 | 915.07 | 167.49 | 747.58 | 33 (31) |
| Fiji | 0.68 | 1347.69 | 167.4 | 1180.3 | 67 (67) |
| Finland | 0.86 | 3035.17 | 167.4 | 2867.77 | 146 (146) |
| France | 0.84 | 3226.79 | 167.13 | 3059.66 | 152 (152) |
| Gabon | 0.63 | 1931.68 | 167.15 | 1764.54 | 94 (94) |
| Gambia | 0.41 | 405.28 | 167.18 | 238.11 | 5 (3) |
| Georgia | 0.73 | 2846.9 | 167.12 | 2679.78 | 138 (138) |
| Germany | 0.9 | 3482.75 | 167.13 | 3315.63 | 163 (163) |
| Ghana | 0.56 | 920.45 | 167.12 | 753.32 | 37 (35) |
| Greece | 0.79 | 4832.04 | 167.12 | 4664.93 | 195 (195) |
| Greenland | 0.83 | 8505.16 | 167.39 | 8337.77 | 204 (204) |
| Grenada | 0.67 | 1497.93 | 167.27 | 1330.66 | 76 (76) |
| Guam | 0.8 | 2807.59 | 167.16 | 2640.43 | 134 (134) |
| Guatemala | 0.54 | 815.55 | 167.11 | 648.44 | 22 (21) |
| Guinea | 0.34 | 1074 | 167.25 | 906.76 | 44 (44) |
| Guinea-Bissau | 0.35 | 1105.25 | 167.12 | 938.13 | 47 (47) |
| Guyana | 0.65 | 816.51 | 167.12 | 649.39 | 23 (22) |
| Haiti | 0.45 | 1436 | 167.39 | 1268.61 | 73 (73) |
| Honduras | 0.51 | 2777.03 | 167.13 | 2609.9 | 132 (132) |
| Hungary | 0.79 | 4735.76 | 167.4 | 4568.36 | 194 (194) |
| Iceland | 0.88 | 3673.12 | 167.21 | 3505.91 | 170 (170) |
| India | 0.58 | 682.33 | 167.14 | 515.19 | 14 (13) |
| Indonesia | 0.66 | 2997.33 | 167.3 | 2830.03 | 143 (143) |
| Iran (Islamic Republic of) | 0.7 | 1444.44 | 167.58 | 1276.86 | 74 (74) |
| Iraq | 0.66 | 2698.55 | 167.15 | 2531.4 | 130 (130) |
| Ireland | 0.87 | 3593.78 | 167.12 | 3426.67 | 167 (167) |
| Israel | 0.81 | 2581.32 | 167.13 | 2414.19 | 123 (123) |
| Italy | 0.81 | 3615.2 | 167.2 | 3448 | 168 (168) |
| Jamaica | 0.68 | 2108.84 | 167.58 | 1941.26 | 102 (102) |
| Japan | 0.87 | 3507.14 | 167.11 | 3340.02 | 164 (164) |
| Jordan | 0.73 | 1393.28 | 167.11 | 1226.17 | 69 (69) |
| Kazakhstan | 0.73 | 1650.48 | 167.43 | 1483.04 | 85 (85) |
| Kenya | 0.52 | 416.78 | 167.14 | 249.64 | 6 (4) |
| Kiribati | 0.53 | 2043.05 | 167.3 | 1875.75 | 98 (98) |
| Kuwait | 0.85 | 1284.95 | 167.11 | 1117.83 | 63 (63) |
| Kyrgyzstan | 0.6 | 1247.24 | 167.13 | 1080.12 | 60 (60) |
| Lao People's Democratic Republic | 0.49 | 2858.6 | 167.12 | 2691.49 | 140 (140) |
| Latvia | 0.83 | 2805.99 | 167.15 | 2638.84 | 133 (133) |
| Lebanon | 0.74 | 3336.45 | 167.12 | 3169.32 | 158 (158) |
| Lesotho | 0.51 | 2232.51 | 167.49 | 2065.02 | 110 (110) |
| Liberia | 0.35 | 761.7 | 167.13 | 594.56 | 20 (19) |
| Libya | 0.73 | 3339.69 | 167.14 | 3172.54 | 159 (159) |
| Lithuania | 0.86 | 2843.09 | 167.15 | 2675.94 | 137 (137) |
| Luxembourg | 0.88 | 3663.12 | 167.33 | 3495.79 | 169 (169) |
| Madagascar | 0.4 | 641.77 | 167.46 | 474.31 | 12 (11) |
| Malawi | 0.38 | 300.29 | 167.17 | 133.12 | 4 (2) |
| Malaysia | 0.74 | 2348.28 | 167.12 | 2181.16 | 114 (114) |
| Maldives | 0.65 | 1266.39 | 167.27 | 1099.12 | 61 (61) |
| Mali | 0.27 | 641.5 | 269.69 | 371.82 | 8 (10) |
| Malta | 0.8 | 2465.82 | 167.12 | 2298.69 | 120 (120) |
| Marshall Islands | 0.57 | 3870 | 167.5 | 3702.5 | 176 (176) |
| Mauritania | 0.5 | 1089.64 | 167.45 | 922.19 | 45 (45) |
| Mauritius | 0.72 | 1348.84 | 167.16 | 1181.68 | 68 (68) |
| Mexico | 0.66 | 1113.41 | 167.13 | 946.29 | 48 (48) |
| Micronesia (Federated States of) | 0.59 | 3837.4 | 167.17 | 3670.23 | 175 (175) |
| Monaco | 0.91 | 8477.51 | 167.12 | 8310.39 | 203 (203) |
| Mongolia | 0.62 | 2827.72 | 167.12 | 2660.61 | 135 (135) |
| Montenegro | 0.8 | 5648.18 | 167.15 | 5481.03 | 201 (201) |
| Morocco | 0.56 | 1808.04 | 167.18 | 1640.85 | 92 (92) |
| Mozambique | 0.33 | 968.04 | 167.48 | 800.57 | 40 (39) |
| Myanmar | 0.53 | 2459.27 | 167.45 | 2291.82 | 119 (119) |
| Namibia | 0.62 | 665.94 | 167.11 | 498.83 | 13 (12) |
| Nauru | 0.63 | 4958.74 | 167.49 | 4791.26 | 196 (196) |
| Nepal | 0.43 | 717.53 | 167.4 | 550.13 | 17 (17) |
| Netherlands | 0.89 | 4694.4 | 167.26 | 4527.14 | 193 (193) |
| New Zealand | 0.85 | 3365.92 | 167.15 | 3198.77 | 161 (161) |
| Nicaragua | 0.52 | 697.8 | 167.44 | 530.35 | 16 (16) |
| Niger | 0.17 | 630.15 | 500.3 | 129.85 | 3 (7) |
| Nigeria | 0.5 | 211.69 | 167.43 | 44.26 | 2 (1) |
| Niue | 0.73 | 4091.67 | 167.15 | 3924.51 | 183 (183) |
| North Macedonia | 0.75 | 3355.31 | 167.37 | 3187.95 | 160 (160) |
| Northern Mariana Islands | 0.77 | 5529.56 | 167.26 | 5362.3 | 199 (199) |
| Norway | 0.92 | 3833.37 | 167.43 | 3665.94 | 174 (174) |
| Oman | 0.77 | 639.41 | 167.14 | 472.27 | 11 (9) |
| Pakistan | 0.5 | 1641.65 | 167.26 | 1474.39 | 84 (84) |
| Palau | 0.75 | 6890.86 | 167.41 | 6723.45 | 202 (202) |
| Palestine | 0.63 | 2527.31 | 167.12 | 2360.19 | 122 (122) |
| Panama | 0.71 | 1226.05 | 167.21 | 1058.85 | 57 (57) |
| Papua New Guinea | 0.42 | 2613.41 | 167.13 | 2446.28 | 126 (126) |
| Paraguay | 0.64 | 2203.06 | 167.39 | 2035.66 | 108 (108) |
| Peru | 0.66 | 1595.14 | 167.3 | 1427.84 | 81 (81) |
| Philippines | 0.65 | 2090.87 | 167.21 | 1923.66 | 100 (100) |
| Poland | 0.81 | 5421.38 | 167.37 | 5254.01 | 198 (198) |
| Portugal | 0.74 | 2319.12 | 167.11 | 2152 | 113 (113) |
| Puerto Rico | 0.83 | 1301.1 | 167.14 | 1133.97 | 66 (66) |
| Qatar | 0.85 | 2156.21 | 167.15 | 1989.05 | 106 (106) |
| Republic of Korea | 0.89 | 4000.51 | 167.47 | 3833.04 | 180 (180) |
| Republic of Moldova | 0.73 | 1731.49 | 167.33 | 1564.16 | 90 (90) |
| Romania | 0.77 | 2991.2 | 167.55 | 2823.65 | 142 (142) |
| Russian Federation | 0.81 | 2451.53 | 167.12 | 2284.41 | 117 (117) |
| Rwanda | 0.44 | 958.56 | 167.5 | 791.05 | 39 (38) |
| Saint Kitts and Nevis | 0.75 | 1401.54 | 167.42 | 1234.12 | 71 (71) |
| Saint Lucia | 0.67 | 1406.82 | 167.39 | 1239.43 | 72 (72) |
| Saint Vincent and the Grenadines | 0.64 | 1139.44 | 167.33 | 972.11 | 50 (50) |
| Samoa | 0.59 | 1072.68 | 167.44 | 905.25 | 43 (43) |
| San Marino | 0.89 | 2431.75 | 167.16 | 2264.59 | 116 (116) |
| Sao Tome and Principe | 0.51 | 2587.01 | 167.5 | 2419.51 | 124 (124) |
| Saudi Arabia | 0.82 | 891.51 | 167.6 | 723.91 | 31 (29) |
| Senegal | 0.41 | 1168.1 | 167.4 | 1000.7 | 51 (51) |
| Serbia | 0.79 | 4258.41 | 167.4 | 4091.01 | 189 (189) |
| Seychelles | 0.73 | 2047.61 | 167.13 | 1880.48 | 99 (99) |
| Sierra Leone | 0.36 | 854.55 | 167.23 | 687.33 | 26 (25) |
| Singapore | 0.86 | 2629.43 | 167.14 | 2462.29 | 127 (127) |
| Slovakia | 0.81 | 3005.59 | 167.12 | 2838.46 | 144 (144) |
| Slovenia | 0.84 | 3754.38 | 167.32 | 3587.06 | 172 (172) |
| Solomon Islands | 0.43 | 2205.52 | 167.12 | 2038.39 | 109 (109) |
| Somalia | 0.08 | 552.39 | 552.39 | 0 | 1 (6) |
| South Africa | 0.68 | 2232.52 | 167.12 | 2065.41 | 111 (111) |
| South Sudan | 0.28 | 930.79 | 269.59 | 661.2 | 24 (37) |
| Spain | 0.77 | 3015.51 | 167.26 | 2848.25 | 145 (145) |
| Sri Lanka | 0.7 | 914.16 | 167.39 | 746.77 | 32 (30) |
| Sudan | 0.54 | 1180.46 | 167.21 | 1013.25 | 53 (53) |
| Suriname | 0.63 | 1541.18 | 167.22 | 1373.96 | 78 (78) |
| Sweden | 0.89 | 2852.4 | 167.12 | 2685.28 | 139 (139) |
| Switzerland | 0.93 | 2830.97 | 167.18 | 2663.8 | 136 (136) |
| Syrian Arab Republic | 0.62 | 1529.19 | 167.12 | 1362.06 | 77 (77) |
| Taiwan (Province of China) | 0.87 | 3776.57 | 167.12 | 3609.44 | 173 (173) |
| Tajikistan | 0.54 | 919.01 | 167.11 | 751.9 | 36 (34) |
| Thailand | 0.68 | 3375.22 | 167.3 | 3207.92 | 162 (162) |
| Timor-Leste | 0.44 | 1939.83 | 167.25 | 1772.59 | 95 (95) |
| Togo | 0.41 | 1011.83 | 167.12 | 844.72 | 42 (41) |
| Tokelau | 0.69 | 3247.05 | 167.12 | 3079.93 | 155 (155) |
| Tonga | 0.63 | 4159.65 | 167.41 | 3992.25 | 187 (187) |
| Trinidad and Tobago | 0.77 | 1172.91 | 167.49 | 1005.42 | 52 (52) |
| Tunisia | 0.68 | 2169.9 | 167.14 | 2002.76 | 107 (107) |
| Turkey | #N/A | 4113.56 | 167.43 | 3946.13 | 184 (184) |
| Turkmenistan | 0.68 | 804.89 | 167.12 | 637.77 | 21 (20) |
| Tuvalu | 0.58 | 3185.23 | 167.25 | 3017.98 | 151 (151) |
| Uganda | 0.42 | 866.33 | 167.2 | 699.13 | 27 (26) |
| Ukraine | 0.76 | 1549.22 | 167.17 | 1382.05 | 79 (79) |
| United Arab Emirates | 0.85 | 2741.65 | 167.39 | 2574.26 | 131 (131) |
| United Kingdom | 0.86 | 4453.5 | 167.2 | 4286.3 | 192 (192) |
| United Republic of Tanzania | 0.45 | 851.34 | 167.43 | 683.91 | 25 (24) |
| United States Virgin Islands | 0.82 | 1617.22 | 167.37 | 1449.86 | 83 (83) |
| United States of America | 0.86 | 4323.01 | 167.12 | 4155.9 | 190 (190) |
| Uruguay | 0.72 | 3972.94 | 167.23 | 3805.71 | 179 (179) |
| Uzbekistan | 0.66 | 736.61 | 167.33 | 569.28 | 19 (18) |
| Vanuatu | 0.47 | 2467.97 | 167.12 | 2300.85 | 121 (121) |
| Venezuela (Bolivarian Republic of) | 0.6 | 2299.51 | 167.22 | 2132.29 | 112 (112) |
| Viet Nam | 0.63 | 2394.61 | 167.17 | 2227.43 | 115 (115) |
| Yemen | 0.45 | 1293.32 | 167.39 | 1125.93 | 64 (64) |
| Zambia | 0.51 | 1221 | 167.14 | 1053.86 | 56 (56) |
| Zimbabwe | 0.47 | 1726.84 | 167.26 | 1559.58 | 89 (89) |

Notes: ASDR tares are reported per 100,000 population.

Abbreviations: DALYs: disability-adjusted life years; EOLC, early-onset lung cancer; MOLC, middle-onset lung cancer; LOLC, late-onset lung cancer; SDI: socio-demographic index; ASDR, age-standardized disability-adjusted life year rate; UI: uncertainty interval.

# **Supplementary Table S9.** Frontier DALYs, and effective difference of EOLC, MOLC and LOLC **in male** in 2021, by country or territory.

| Country/Territory | SDI | ASDR | Frontier DALYs | Effective difference | Effective difference rank (ASDR rank) |
| --- | --- | --- | --- | --- | --- |
| EOLC |  |  |  |  |  |
| Afghanistan | 0.34 | 112.19 | 10.1 | 102.09 | 98 (100) |
| Albania | 0.71 | 211.37 | 9.65 | 201.72 | 162 (162) |
| Algeria | 0.66 | 57.79 | 9.77 | 48.02 | 34 (32) |
| American Samoa | 0.72 | 286.72 | 9.69 | 277.04 | 182 (182) |
| Andorra | 0.87 | 203.97 | 9.66 | 194.31 | 159 (159) |
| Angola | 0.45 | 124.61 | 9.68 | 114.93 | 114 (113) |
| Antigua and Barbuda | 0.75 | 46.7 | 9.67 | 37.03 | 20 (17) |
| Argentina | 0.72 | 122.02 | 9.68 | 112.34 | 111 (111) |
| Armenia | 0.7 | 252.66 | 9.71 | 242.95 | 174 (174) |
| Australia | 0.84 | 93.61 | 9.65 | 83.97 | 75 (75) |
| Austria | 0.85 | 79.66 | 9.73 | 69.93 | 57 (56) |
| Azerbaijan | 0.69 | 212.79 | 9.65 | 203.14 | 164 (164) |
| Bahamas | 0.81 | 184.72 | 9.68 | 175.04 | 153 (153) |
| Bahrain | 0.75 | 97.2 | 9.67 | 87.53 | 82 (82) |
| Bangladesh | 0.49 | 56.39 | 9.67 | 46.72 | 32 (30) |
| Barbados | 0.75 | 70.22 | 9.75 | 60.48 | 49 (48) |
| Belarus | 0.78 | 223.02 | 9.7 | 213.32 | 169 (169) |
| Belgium | 0.85 | 151.88 | 9.67 | 142.21 | 134 (134) |
| Belize | 0.61 | 124.48 | 9.73 | 114.75 | 113 (112) |
| Benin | 0.37 | 45.66 | 9.92 | 35.73 | 18 (14) |
| Bermuda | 0.82 | 167.36 | 9.65 | 157.71 | 148 (148) |
| Bhutan | 0.47 | 47.49 | 9.68 | 37.81 | 22 (19) |
| Bolivia (Plurinational State of) | 0.6 | 76.95 | 9.71 | 67.25 | 55 (54) |
| Bosnia and Herzegovina | 0.72 | 246.24 | 9.76 | 236.48 | 173 (173) |
| Botswana | 0.64 | 150.39 | 9.78 | 140.61 | 133 (133) |
| Brazil | 0.65 | 89.66 | 9.68 | 79.98 | 71 (71) |
| Brunei Darussalam | 0.81 | 140.31 | 9.66 | 130.65 | 125 (125) |
| Bulgaria | 0.77 | 340.95 | 9.78 | 331.17 | 196 (196) |
| Burkina Faso | 0.29 | 45.97 | 24.04 | 21.93 | 10 (15) |
| Burundi | 0.29 | 59.26 | 23.8 | 35.46 | 17 (34) |
| Cabo Verde | 0.53 | 109.3 | 9.65 | 99.65 | 97 (97) |
| Cambodia | 0.47 | 208.2 | 9.75 | 198.46 | 161 (161) |
| Cameroon | 0.48 | 66.18 | 9.65 | 56.53 | 44 (43) |
| Canada | 0.87 | 77.21 | 9.68 | 67.53 | 56 (55) |
| Central African Republic | 0.31 | 134.23 | 19.64 | 114.59 | 112 (120) |
| Chad | 0.24 | 52.39 | 24.33 | 28.07 | 13 (23) |
| Chile | 0.77 | 80.86 | 9.69 | 71.17 | 59 (58) |
| China | 0.72 | 310.13 | 9.69 | 300.44 | 189 (189) |
| Colombia | 0.66 | 57.37 | 9.75 | 47.63 | 33 (31) |
| Comoros | 0.48 | 68.33 | 9.68 | 58.65 | 46 (45) |
| Congo | 0.58 | 121.84 | 9.69 | 112.15 | 110 (110) |
| Cook Islands | 0.78 | 321.11 | 9.66 | 311.45 | 192 (192) |
| Costa Rica | 0.7 | 59.28 | 9.68 | 49.6 | 36 (35) |
| Croatia | 0.8 | 195.37 | 9.65 | 185.72 | 156 (156) |
| Cuba | 0.67 | 162.25 | 9.69 | 152.56 | 145 (145) |
| Cyprus | 0.84 | 118.32 | 9.69 | 108.63 | 108 (108) |
| Czechia | 0.83 | 114.07 | 9.72 | 104.36 | 104 (104) |
| Côte d'Ivoire | #N/A | 29.48 | 9.65 | 19.83 | 8 (5) |
| Democratic People's Republic of Korea | 0.57 | 296.77 | 9.67 | 287.1 | 185 (185) |
| Democratic Republic of the Congo | 0.38 | 76.53 | 9.85 | 66.69 | 54 (53) |
| Denmark | 0.9 | 86.47 | 9.66 | 76.8 | 66 (66) |
| Djibouti | 0.49 | 96.19 | 9.65 | 86.53 | 81 (81) |
| Dominica | 0.75 | 159.82 | 9.65 | 150.17 | 141 (141) |
| Dominican Republic | 0.62 | 155.11 | 9.66 | 145.45 | 137 (137) |
| Ecuador | 0.66 | 66.85 | 9.65 | 57.21 | 45 (44) |
| Egypt | 0.61 | 147.08 | 9.75 | 137.32 | 129 (129) |
| El Salvador | 0.56 | 94.05 | 9.71 | 84.34 | 76 (76) |
| Equatorial Guinea | 0.66 | 131.37 | 9.71 | 121.67 | 118 (117) |
| Eritrea | 0.4 | 113.48 | 9.67 | 103.8 | 103 (103) |
| Estonia | 0.84 | 141.88 | 9.73 | 132.16 | 127 (127) |
| Eswatini | 0.59 | 304.74 | 9.71 | 295.03 | 186 (186) |
| Ethiopia | 0.36 | 80.04 | 9.99 | 70.05 | 58 (57) |
| Fiji | 0.68 | 82.79 | 9.7 | 73.09 | 61 (60) |
| Finland | 0.86 | 90.43 | 9.65 | 80.78 | 72 (72) |
| France | 0.84 | 252.78 | 9.68 | 243.1 | 175 (175) |
| Gabon | 0.63 | 152.47 | 9.66 | 142.81 | 135 (135) |
| Gambia | 0.41 | 28.13 | 9.73 | 18.39 | 7 (4) |
| Georgia | 0.73 | 313.13 | 9.65 | 303.49 | 190 (190) |
| Germany | 0.9 | 120.31 | 9.66 | 110.65 | 109 (109) |
| Ghana | 0.56 | 31.11 | 9.77 | 21.34 | 9 (7) |
| Greece | 0.79 | 193.8 | 9.71 | 184.09 | 155 (155) |
| Greenland | 0.83 | 329.28 | 9.65 | 319.63 | 194 (194) |
| Grenada | 0.67 | 99.24 | 9.66 | 89.57 | 87 (87) |
| Guam | 0.8 | 393.25 | 9.72 | 383.52 | 200 (200) |
| Guatemala | 0.54 | 55.17 | 9.67 | 45.5 | 28 (26) |
| Guinea | 0.34 | 91.49 | 10.1 | 81.39 | 73 (73) |
| Guinea-Bissau | 0.35 | 83.24 | 9.99 | 73.25 | 62 (61) |
| Guyana | 0.65 | 89.57 | 9.65 | 79.93 | 70 (70) |
| Haiti | 0.45 | 106.33 | 9.69 | 96.64 | 94 (94) |
| Honduras | 0.51 | 75.61 | 9.68 | 65.93 | 51 (50) |
| Hungary | 0.79 | 213.74 | 9.77 | 203.97 | 165 (165) |
| Iceland | 0.88 | 115.05 | 9.68 | 105.37 | 107 (107) |
| India | 0.58 | 75.84 | 9.77 | 66.06 | 52 (51) |
| Indonesia | 0.66 | 164.28 | 9.66 | 154.62 | 147 (147) |
| Iran (Islamic Republic of) | 0.7 | 103.69 | 9.65 | 94.04 | 92 (92) |
| Iraq | 0.66 | 106.93 | 9.71 | 97.22 | 95 (95) |
| Ireland | 0.87 | 98.8 | 9.65 | 89.15 | 86 (86) |
| Israel | 0.81 | 98.38 | 9.77 | 88.6 | 85 (85) |
| Italy | 0.81 | 109.25 | 9.66 | 99.59 | 96 (96) |
| Jamaica | 0.68 | 134.81 | 9.71 | 125.1 | 122 (122) |
| Japan | 0.87 | 76.35 | 9.71 | 66.65 | 53 (52) |
| Jordan | 0.73 | 102.89 | 9.73 | 93.16 | 91 (91) |
| Kazakhstan | 0.73 | 161.19 | 9.65 | 151.54 | 142 (142) |
| Kenya | 0.52 | 25 | 9.68 | 15.32 | 6 (3) |
| Kiribati | 0.53 | 280.18 | 9.66 | 270.52 | 179 (179) |
| Kuwait | 0.85 | 53.55 | 9.69 | 43.86 | 26 (24) |
| Kyrgyzstan | 0.6 | 125.69 | 9.66 | 116.03 | 115 (114) |
| Lao People's Democratic Republic | 0.49 | 180.18 | 9.68 | 170.49 | 152 (152) |
| Latvia | 0.83 | 180.09 | 9.65 | 170.44 | 151 (151) |
| Lebanon | 0.74 | 154.7 | 9.66 | 145.04 | 136 (136) |
| Lesotho | 0.51 | 359.05 | 9.71 | 349.34 | 197 (197) |
| Liberia | 0.35 | 40.52 | 9.99 | 30.53 | 15 (12) |
| Libya | 0.73 | 265.17 | 9.67 | 255.5 | 178 (178) |
| Lithuania | 0.86 | 206.68 | 9.66 | 197.02 | 160 (160) |
| Luxembourg | 0.88 | 111.88 | 9.75 | 102.13 | 99 (98) |
| Madagascar | 0.4 | 54.86 | 9.76 | 45.09 | 27 (25) |
| Malawi | 0.38 | 32.23 | 9.87 | 22.35 | 11 (9) |
| Malaysia | 0.74 | 148.33 | 9.68 | 138.65 | 132 (132) |
| Maldives | 0.65 | 46.35 | 9.67 | 36.69 | 19 (16) |
| Mali | 0.27 | 31.94 | 23.86 | 8.08 | 4 (8) |
| Malta | 0.8 | 141.55 | 9.69 | 131.86 | 126 (126) |
| Marshall Islands | 0.57 | 365.73 | 9.66 | 356.07 | 198 (198) |
| Mauritania | 0.5 | 42.3 | 9.75 | 32.56 | 16 (13) |
| Mauritius | 0.72 | 94.33 | 9.76 | 84.57 | 77 (79) |
| Mexico | 0.66 | 60.07 | 9.68 | 50.39 | 37 (36) |
| Micronesia (Federated States of) | 0.59 | 386.82 | 9.65 | 377.18 | 199 (199) |
| Monaco | 0.91 | 558.52 | 9.65 | 548.87 | 204 (204) |
| Mongolia | 0.62 | 254.8 | 9.76 | 245.04 | 176 (176) |
| Montenegro | 0.8 | 441.97 | 9.65 | 432.32 | 201 (201) |
| Morocco | 0.56 | 93.39 | 9.66 | 83.73 | 74 (74) |
| Mozambique | 0.33 | 51.82 | 10.26 | 41.56 | 25 (22) |
| Myanmar | 0.53 | 140.15 | 9.65 | 130.5 | 124 (124) |
| Namibia | 0.62 | 64.33 | 9.67 | 54.66 | 40 (39) |
| Nauru | 0.63 | 518.44 | 9.68 | 508.76 | 203 (203) |
| Nepal | 0.43 | 56.18 | 9.66 | 46.52 | 31 (29) |
| Netherlands | 0.89 | 131.19 | 9.66 | 121.53 | 117 (116) |
| New Zealand | 0.85 | 83.73 | 9.67 | 74.06 | 65 (64) |
| Nicaragua | 0.52 | 36.77 | 9.66 | 27.11 | 12 (10) |
| Niger | 0.17 | 29.69 | 25.81 | 3.88 | 3 (6) |
| Nigeria | 0.5 | 10.34 | 9.68 | 0.65 | 2 (1) |
| Niue | 0.73 | 243.53 | 9.66 | 233.87 | 172 (172) |
| North Macedonia | 0.75 | 259.68 | 9.66 | 250.02 | 177 (177) |
| Northern Mariana Islands | 0.77 | 327.41 | 9.74 | 317.67 | 193 (193) |
| Norway | 0.92 | 65.9 | 9.73 | 56.17 | 43 (42) |
| Oman | 0.77 | 21.54 | 9.7 | 11.84 | 5 (2) |
| Pakistan | 0.5 | 157.65 | 9.74 | 147.91 | 139 (139) |
| Palau | 0.75 | 443.17 | 9.67 | 433.5 | 202 (202) |
| Palestine | 0.63 | 157.7 | 9.67 | 148.02 | 140 (140) |
| Panama | 0.71 | 55.86 | 9.73 | 46.13 | 30 (28) |
| Papua New Guinea | 0.42 | 163.82 | 9.75 | 154.08 | 146 (146) |
| Paraguay | 0.64 | 100.81 | 9.68 | 91.13 | 89 (89) |
| Peru | 0.66 | 87.91 | 9.67 | 78.25 | 67 (67) |
| Philippines | 0.65 | 161.77 | 9.74 | 152.03 | 144 (144) |
| Poland | 0.81 | 161.32 | 9.71 | 151.62 | 143 (143) |
| Portugal | 0.74 | 144.42 | 9.77 | 134.65 | 128 (128) |
| Puerto Rico | 0.83 | 97.61 | 9.65 | 87.96 | 84 (84) |
| Qatar | 0.85 | 61.37 | 9.68 | 51.69 | 38 (37) |
| Republic of Korea | 0.89 | 83.53 | 9.71 | 73.83 | 63 (63) |
| Republic of Moldova | 0.73 | 172.16 | 9.67 | 162.48 | 149 (149) |
| Romania | 0.77 | 282.02 | 9.67 | 272.36 | 181 (181) |
| Russian Federation | 0.81 | 217.07 | 9.65 | 207.42 | 166 (166) |
| Rwanda | 0.44 | 72.51 | 9.65 | 62.86 | 50 (49) |
| Saint Kitts and Nevis | 0.75 | 64.75 | 9.68 | 55.07 | 41 (40) |
| Saint Lucia | 0.67 | 114.46 | 9.67 | 104.79 | 105 (105) |
| Saint Vincent and the Grenadines | 0.64 | 94.32 | 9.71 | 84.61 | 78 (78) |
| Samoa | 0.59 | 81.65 | 9.68 | 71.98 | 60 (59) |
| San Marino | 0.89 | 130.85 | 9.66 | 121.2 | 116 (115) |
| Sao Tome and Principe | 0.51 | 112.36 | 9.72 | 102.64 | 101 (101) |
| Saudi Arabia | 0.82 | 47.67 | 9.71 | 37.96 | 23 (20) |
| Senegal | 0.41 | 58.18 | 9.7 | 48.48 | 35 (33) |
| Serbia | 0.79 | 314.86 | 9.65 | 305.22 | 191 (191) |
| Seychelles | 0.73 | 134.17 | 9.67 | 124.5 | 120 (119) |
| Sierra Leone | 0.36 | 47.09 | 9.99 | 37.1 | 21 (18) |
| Singapore | 0.86 | 61.56 | 9.68 | 51.88 | 39 (38) |
| Slovakia | 0.81 | 178.85 | 9.75 | 169.1 | 150 (150) |
| Slovenia | 0.84 | 101.86 | 9.69 | 92.18 | 90 (90) |
| Solomon Islands | 0.43 | 334.23 | 9.7 | 324.53 | 195 (195) |
| Somalia | 0.08 | 69.51 | 69.46 | 0.04 | 1 (47) |
| South Africa | 0.68 | 221.61 | 9.67 | 211.94 | 168 (168) |
| South Sudan | 0.28 | 83.43 | 24.13 | 59.3 | 48 (62) |
| Spain | 0.77 | 134.45 | 9.76 | 124.69 | 121 (121) |
| Sri Lanka | 0.7 | 89.06 | 9.72 | 79.34 | 69 (69) |
| Sudan | 0.54 | 94.32 | 9.65 | 84.66 | 79 (77) |
| Suriname | 0.63 | 147.2 | 9.74 | 137.46 | 130 (130) |
| Sweden | 0.89 | 39.77 | 9.67 | 30.1 | 14 (11) |
| Switzerland | 0.93 | 83.79 | 9.75 | 74.03 | 64 (65) |
| Syrian Arab Republic | 0.62 | 132.05 | 9.7 | 122.35 | 119 (118) |
| Taiwan (Province of China) | 0.87 | 186.66 | 9.67 | 176.99 | 154 (154) |
| Tajikistan | 0.54 | 100.43 | 9.75 | 90.68 | 88 (88) |
| Thailand | 0.68 | 290.59 | 9.73 | 280.86 | 184 (184) |
| Timor-Leste | 0.44 | 112.09 | 9.74 | 102.35 | 100 (99) |
| Togo | 0.41 | 65.3 | 9.67 | 55.64 | 42 (41) |
| Tokelau | 0.69 | 211.64 | 9.65 | 201.99 | 163 (163) |
| Tonga | 0.63 | 309.27 | 9.65 | 299.62 | 188 (188) |
| Trinidad and Tobago | 0.77 | 112.77 | 9.65 | 103.12 | 102 (102) |
| Tunisia | 0.68 | 203.14 | 9.72 | 193.42 | 158 (158) |
| Turkey | #N/A | 307.28 | 9.76 | 297.52 | 187 (187) |
| Turkmenistan | 0.68 | 147.23 | 9.69 | 137.54 | 131 (131) |
| Tuvalu | 0.58 | 281.25 | 9.68 | 271.57 | 180 (180) |
| Uganda | 0.42 | 55.29 | 9.69 | 45.59 | 29 (27) |
| Ukraine | 0.76 | 242.59 | 9.67 | 232.92 | 171 (171) |
| United Arab Emirates | 0.85 | 49.65 | 9.66 | 39.98 | 24 (21) |
| United Kingdom | 0.86 | 97.56 | 9.7 | 87.86 | 83 (83) |
| United Republic of Tanzania | 0.45 | 68.9 | 9.69 | 59.21 | 47 (46) |
| United States Virgin Islands | 0.82 | 201.03 | 9.66 | 191.37 | 157 (157) |
| United States of America | 0.86 | 95.97 | 9.68 | 86.29 | 80 (80) |
| Uruguay | 0.72 | 289.65 | 9.66 | 279.98 | 183 (183) |
| Uzbekistan | 0.66 | 88.06 | 9.69 | 78.37 | 68 (68) |
| Vanuatu | 0.47 | 235.39 | 9.69 | 225.7 | 170 (170) |
| Venezuela (Bolivarian Republic of) | 0.6 | 137.11 | 9.74 | 127.37 | 123 (123) |
| Viet Nam | 0.63 | 219.3 | 9.7 | 209.61 | 167 (167) |
| Yemen | 0.45 | 105.37 | 9.66 | 95.71 | 93 (93) |
| Zambia | 0.51 | 114.77 | 9.68 | 105.09 | 106 (106) |
| Zimbabwe | 0.47 | 157.57 | 9.73 | 147.83 | 138 (138) |
| MOLC |  |  |  |  |  |
| Afghanistan | 0.34 | 1171.44 | 174.6 | 996.84 | 62 (63) |
| Albania | 0.71 | 3150.3 | 168.12 | 2982.18 | 153 (154) |
| Algeria | 0.66 | 922.02 | 168.05 | 753.96 | 40 (38) |
| American Samoa | 0.72 | 2774 | 168.12 | 2605.88 | 145 (146) |
| Andorra | 0.87 | 3194.97 | 168.25 | 3026.72 | 159 (160) |
| Angola | 0.45 | 1936.35 | 170.7 | 1765.65 | 106 (107) |
| Antigua and Barbuda | 0.75 | 992.8 | 168.06 | 824.75 | 48 (48) |
| Argentina | 0.72 | 2703.15 | 168.39 | 2534.76 | 143 (144) |
| Armenia | 0.7 | 4791.64 | 168.16 | 4623.48 | 186 (187) |
| Australia | 0.84 | 1906.44 | 168.14 | 1738.3 | 103 (104) |
| Austria | 0.85 | 2479.1 | 168.09 | 2311.01 | 136 (137) |
| Azerbaijan | 0.69 | 2680.47 | 168.02 | 2512.44 | 142 (143) |
| Bahamas | 0.81 | 1778.25 | 168.06 | 1610.19 | 96 (96) |
| Bahrain | 0.75 | 1434.16 | 168.25 | 1265.91 | 79 (80) |
| Bangladesh | 0.49 | 773.7 | 170.02 | 603.68 | 26 (23) |
| Barbados | 0.75 | 983.58 | 168.02 | 815.56 | 47 (47) |
| Belarus | 0.78 | 5088.29 | 168.16 | 4920.13 | 190 (191) |
| Belgium | 0.85 | 3377.31 | 168.66 | 3208.66 | 161 (162) |
| Belize | 0.61 | 1339.88 | 168.05 | 1171.83 | 74 (74) |
| Benin | 0.37 | 825.43 | 172.91 | 652.52 | 30 (29) |
| Bermuda | 0.82 | 2531.25 | 168.02 | 2363.23 | 139 (140) |
| Bhutan | 0.47 | 607.61 | 171.66 | 435.94 | 13 (12) |
| Bolivia (Plurinational State of) | 0.6 | 1178.69 | 168.04 | 1010.65 | 63 (64) |
| Bosnia and Herzegovina | 0.72 | 5958.31 | 168.68 | 5789.64 | 197 (198) |
| Botswana | 0.64 | 2211.76 | 168.11 | 2043.65 | 121 (122) |
| Brazil | 0.65 | 1534.84 | 168.06 | 1366.79 | 82 (83) |
| Brunei Darussalam | 0.81 | 1912.65 | 168.1 | 1744.55 | 104 (105) |
| Bulgaria | 0.77 | 6305.83 | 168.23 | 6137.6 | 199 (200) |
| Burkina Faso | 0.29 | 811.61 | 371.56 | 440.04 | 14 (28) |
| Burundi | 0.29 | 789.07 | 372.61 | 416.46 | 11 (25) |
| Cabo Verde | 0.53 | 2030.39 | 168.02 | 1862.37 | 111 (112) |
| Cambodia | 0.47 | 3706.01 | 171.37 | 3534.64 | 171 (172) |
| Cameroon | 0.48 | 1102.85 | 169.95 | 932.91 | 54 (55) |
| Canada | 0.87 | 2057.51 | 168.23 | 1889.28 | 113 (114) |
| Central African Republic | 0.31 | 1896.94 | 182.26 | 1714.69 | 101 (102) |
| Chad | 0.24 | 923.72 | 372.45 | 551.27 | 23 (39) |
| Chile | 0.77 | 1341.83 | 168.02 | 1173.8 | 75 (75) |
| China | 0.72 | 3759.29 | 168.08 | 3591.22 | 172 (173) |
| Colombia | 0.66 | 973.93 | 168.06 | 805.87 | 46 (46) |
| Comoros | 0.48 | 904.59 | 171.55 | 733.05 | 38 (37) |
| Congo | 0.58 | 1777.45 | 168.5 | 1608.95 | 94 (94) |
| Cook Islands | 0.78 | 3425.15 | 168.46 | 3256.69 | 163 (164) |
| Costa Rica | 0.7 | 802.78 | 168.02 | 634.76 | 29 (26) |
| Croatia | 0.8 | 5064.12 | 168.41 | 4895.71 | 188 (189) |
| Cuba | 0.67 | 3415.16 | 168.3 | 3246.86 | 162 (163) |
| Cyprus | 0.84 | 2336.67 | 168.05 | 2168.62 | 125 (126) |
| Czechia | 0.83 | 3181.89 | 168.09 | 3013.8 | 158 (159) |
| Côte d'Ivoire | #N/A | 511.85 | 169.31 | 342.54 | 9 (7) |
| Democratic People's Republic of Korea | 0.57 | 3655.93 | 168.09 | 3487.84 | 170 (171) |
| Democratic Republic of the Congo | 0.38 | 1217.29 | 172.43 | 1044.86 | 66 (67) |
| Denmark | 0.9 | 2447.62 | 168.13 | 2279.49 | 133 (134) |
| Djibouti | 0.49 | 1342.6 | 171.26 | 1171.34 | 73 (76) |
| Dominica | 0.75 | 2205.66 | 168.02 | 2037.63 | 119 (120) |
| Dominican Republic | 0.62 | 1607.71 | 168.03 | 1439.67 | 86 (87) |
| Ecuador | 0.66 | 603.73 | 168.14 | 435.59 | 12 (11) |
| Egypt | 0.61 | 1768.03 | 168.02 | 1600.01 | 91 (92) |
| El Salvador | 0.56 | 927.04 | 168.06 | 758.98 | 41 (40) |
| Equatorial Guinea | 0.66 | 1992.35 | 168.18 | 1824.18 | 108 (109) |
| Eritrea | 0.4 | 1315.15 | 170.65 | 1144.5 | 71 (72) |
| Estonia | 0.84 | 3832.85 | 168.24 | 3664.61 | 173 (174) |
| Eswatini | 0.59 | 3601.39 | 168.07 | 3433.32 | 168 (169) |
| Ethiopia | 0.36 | 944.5 | 173.01 | 771.49 | 43 (42) |
| Fiji | 0.68 | 1037.54 | 168.03 | 869.51 | 52 (51) |
| Finland | 0.86 | 1842.65 | 168.13 | 1674.52 | 98 (99) |
| France | 0.84 | 4119.34 | 168.27 | 3951.07 | 179 (180) |
| Gabon | 0.63 | 2246.99 | 168.35 | 2078.64 | 124 (125) |
| Gambia | 0.41 | 462.23 | 170.43 | 291.8 | 7 (5) |
| Georgia | 0.73 | 5158.07 | 168.08 | 4989.99 | 191 (192) |
| Germany | 0.9 | 3051.75 | 168.02 | 2883.72 | 151 (152) |
| Ghana | 0.56 | 849.89 | 168.04 | 681.85 | 32 (31) |
| Greece | 0.79 | 5066.47 | 168.04 | 4898.42 | 189 (190) |
| Greenland | 0.83 | 6252.09 | 168.02 | 6084.06 | 198 (199) |
| Grenada | 0.67 | 1302.35 | 168.03 | 1134.32 | 70 (71) |
| Guam | 0.8 | 2998.26 | 168.03 | 2830.23 | 150 (151) |
| Guatemala | 0.54 | 448.38 | 168.31 | 280.07 | 6 (4) |
| Guinea | 0.34 | 1151.84 | 174.36 | 977.49 | 58 (59) |
| Guinea-Bissau | 0.35 | 1280.88 | 173.14 | 1107.74 | 68 (69) |
| Guyana | 0.65 | 900.78 | 168.05 | 732.73 | 37 (36) |
| Haiti | 0.45 | 1444.07 | 170.42 | 1273.65 | 80 (81) |
| Honduras | 0.51 | 1769.32 | 168.38 | 1600.95 | 92 (93) |
| Hungary | 0.79 | 6429.42 | 168.07 | 6261.34 | 200 (201) |
| Iceland | 0.88 | 1551.43 | 168.07 | 1383.36 | 83 (84) |
| India | 0.58 | 866.12 | 168.07 | 698.06 | 33 (32) |
| Indonesia | 0.66 | 2659.46 | 168.04 | 2491.42 | 141 (142) |
| Iran (Islamic Republic of) | 0.7 | 1102.45 | 168.2 | 934.25 | 55 (54) |
| Iraq | 0.66 | 2213.02 | 168.31 | 2044.71 | 122 (123) |
| Ireland | 0.87 | 1904.42 | 168.02 | 1736.39 | 102 (103) |
| Israel | 0.81 | 2126 | 168.05 | 1957.95 | 117 (118) |
| Italy | 0.81 | 2421.88 | 168.02 | 2253.86 | 131 (132) |
| Jamaica | 0.68 | 2203.8 | 168.44 | 2035.36 | 118 (119) |
| Japan | 0.87 | 1778.2 | 168.06 | 1610.14 | 95 (95) |
| Jordan | 0.73 | 1336.69 | 168.1 | 1168.59 | 72 (73) |
| Kazakhstan | 0.73 | 3175.86 | 168.18 | 3007.68 | 157 (158) |
| Kenya | 0.52 | 405.39 | 168.04 | 237.35 | 4 (2) |
| Kiribati | 0.53 | 2511.53 | 168.14 | 2343.39 | 138 (139) |
| Kuwait | 0.85 | 734.46 | 168.03 | 566.43 | 24 (21) |
| Kyrgyzstan | 0.6 | 1931.89 | 168.47 | 1763.42 | 105 (106) |
| Lao People's Democratic Republic | 0.49 | 2800.15 | 170.33 | 2629.82 | 147 (148) |
| Latvia | 0.83 | 4283.45 | 168.14 | 4115.32 | 182 (183) |
| Lebanon | 0.74 | 2757.29 | 168.28 | 2589.01 | 144 (145) |
| Lesotho | 0.51 | 4721.7 | 168.04 | 4553.66 | 185 (186) |
| Liberia | 0.35 | 650.86 | 173.07 | 477.78 | 15 (13) |
| Libya | 0.73 | 3647.44 | 168.03 | 3479.41 | 169 (170) |
| Lithuania | 0.86 | 4399.92 | 168.09 | 4231.83 | 183 (184) |
| Luxembourg | 0.88 | 2920.6 | 169 | 2751.59 | 148 (149) |
| Madagascar | 0.4 | 691.54 | 170.96 | 520.58 | 18 (17) |
| Malawi | 0.38 | 446.26 | 172.58 | 273.67 | 5 (3) |
| Malaysia | 0.74 | 2222.64 | 168.36 | 2054.28 | 123 (124) |
| Maldives | 0.65 | 675.06 | 168.23 | 506.83 | 16 (14) |
| Mali | 0.27 | 539.73 | 374.79 | 164.93 | 3 (9) |
| Malta | 0.8 | 2357.47 | 168.02 | 2189.45 | 127 (127) |
| Marshall Islands | 0.57 | 3535.45 | 168.03 | 3367.43 | 165 (166) |
| Mauritania | 0.5 | 782.06 | 169.33 | 612.73 | 27 (24) |
| Mauritius | 0.72 | 1297.04 | 168.16 | 1128.88 | 69 (70) |
| Mexico | 0.66 | 683.35 | 168.08 | 515.27 | 17 (15) |
| Micronesia (Federated States of) | 0.59 | 3917.08 | 168.14 | 3748.93 | 175 (176) |
| Monaco | 0.91 | 6589 | 168.02 | 6420.98 | 201 (202) |
| Mongolia | 0.62 | 3925.16 | 168.02 | 3757.14 | 176 (177) |
| Montenegro | 0.8 | 7069.53 | 168.04 | 6901.49 | 203 (204) |
| Morocco | 0.56 | 2409.21 | 168.38 | 2240.82 | 130 (131) |
| Mozambique | 0.33 | 894.71 | 179.72 | 714.99 | 36 (35) |
| Myanmar | 0.53 | 2394.71 | 168.08 | 2226.63 | 129 (130) |
| Namibia | 0.62 | 878.63 | 168.18 | 710.44 | 35 (34) |
| Nauru | 0.63 | 5338.14 | 168.14 | 5170 | 193 (194) |
| Nepal | 0.43 | 714.96 | 170.05 | 544.91 | 21 (19) |
| Netherlands | 0.89 | 2479.04 | 168.25 | 2310.79 | 135 (136) |
| New Zealand | 0.85 | 1742.97 | 168.03 | 1574.94 | 90 (91) |
| Nicaragua | 0.52 | 507.95 | 168.06 | 339.9 | 8 (6) |
| Niger | 0.17 | 548.47 | 461.59 | 86.88 | 2 (10) |
| Nigeria | 0.5 | 168.02 | 168.02 | 0 | 1 (1) |
| Niue | 0.73 | 3156.81 | 168.03 | 2988.78 | 154 (155) |
| North Macedonia | 0.75 | 5249.26 | 168.09 | 5081.17 | 192 (193) |
| Northern Mariana Islands | 0.77 | 4162.79 | 168.58 | 3994.22 | 180 (181) |
| Norway | 0.92 | 1669.94 | 168.11 | 1501.83 | 88 (89) |
| Oman | 0.77 | 513.95 | 168.02 | 345.93 | 10 (8) |
| Pakistan | 0.5 | 2053.75 | 168.05 | 1885.7 | 112 (113) |
| Palau | 0.75 | 3848.61 | 168.05 | 3680.56 | 174 (175) |
| Palestine | 0.63 | 2443.02 | 168.05 | 2274.97 | 132 (133) |
| Panama | 0.71 | 747.82 | 168.06 | 579.76 | 25 (22) |
| Papua New Guinea | 0.42 | 1941.46 | 171.74 | 1769.72 | 107 (108) |
| Paraguay | 0.64 | 2108.03 | 168.06 | 1939.97 | 115 (116) |
| Peru | 0.66 | 843.68 | 168.04 | 675.64 | 31 (30) |
| Philippines | 0.65 | 2209.75 | 168.04 | 2041.71 | 120 (121) |
| Poland | 0.81 | 5479.87 | 168.03 | 5311.84 | 194 (195) |
| Portugal | 0.74 | 3175.01 | 168.1 | 3006.91 | 156 (157) |
| Puerto Rico | 0.83 | 965.18 | 168.02 | 797.16 | 44 (44) |
| Qatar | 0.85 | 1019.42 | 168.22 | 851.2 | 50 (50) |
| Republic of Korea | 0.89 | 1868.27 | 168.09 | 1700.18 | 100 (101) |
| Republic of Moldova | 0.73 | 4117.72 | 168.04 | 3949.68 | 178 (179) |
| Romania | 0.77 | 5866.12 | 168.12 | 5697.99 | 196 (197) |
| Russian Federation | 0.81 | 4476.36 | 168.13 | 4308.23 | 184 (185) |
| Rwanda | 0.44 | 1060.32 | 170.8 | 889.53 | 53 (53) |
| Saint Kitts and Nevis | 0.75 | 1153.12 | 168.04 | 985.08 | 59 (60) |
| Saint Lucia | 0.67 | 1192.12 | 168.02 | 1024.1 | 64 (65) |
| Saint Vincent and the Grenadines | 0.64 | 969.07 | 168.03 | 801.05 | 45 (45) |
| Samoa | 0.59 | 1265.68 | 168.04 | 1097.65 | 67 (68) |
| San Marino | 0.89 | 2085.51 | 168.04 | 1917.46 | 114 (115) |
| Sao Tome and Principe | 0.51 | 2117.11 | 168.02 | 1949.09 | 116 (117) |
| Saudi Arabia | 0.82 | 690.48 | 168.35 | 522.12 | 19 (16) |
| Senegal | 0.41 | 1038.08 | 170.53 | 867.55 | 51 (52) |
| Serbia | 0.79 | 6895.23 | 168.13 | 6727.1 | 202 (203) |
| Seychelles | 0.73 | 1712.14 | 168.02 | 1544.12 | 89 (90) |
| Sierra Leone | 0.36 | 803.42 | 172.96 | 630.47 | 28 (27) |
| Singapore | 0.86 | 1156.39 | 168.48 | 987.91 | 61 (62) |
| Slovakia | 0.81 | 4270.41 | 168.07 | 4102.34 | 181 (182) |
| Slovenia | 0.84 | 3320.43 | 168.02 | 3152.41 | 160 (161) |
| Solomon Islands | 0.43 | 2788.15 | 171.34 | 2616.81 | 146 (147) |
| Somalia | 0.08 | 963.34 | 963.34 | 0 | 1 (43) |
| South Africa | 0.68 | 3168.47 | 168.17 | 3000.3 | 155 (156) |
| South Sudan | 0.28 | 1126.61 | 379.91 | 746.7 | 39 (56) |
| Spain | 0.77 | 3597.48 | 168.06 | 3429.42 | 167 (168) |
| Sri Lanka | 0.7 | 1153.94 | 168.07 | 985.87 | 60 (61) |
| Sudan | 0.54 | 1011.5 | 168.1 | 843.4 | 49 (49) |
| Suriname | 0.63 | 1783.7 | 168.09 | 1615.61 | 97 (98) |
| Sweden | 0.89 | 932.25 | 168.02 | 764.23 | 42 (41) |
| Switzerland | 0.93 | 1866.29 | 168.03 | 1698.26 | 99 (100) |
| Syrian Arab Republic | 0.62 | 1599.2 | 168.11 | 1431.09 | 85 (86) |
| Taiwan (Province of China) | 0.87 | 2490.72 | 168.25 | 2322.47 | 137 (138) |
| Tajikistan | 0.54 | 1143.54 | 168.13 | 975.42 | 57 (58) |
| Thailand | 0.68 | 2561.02 | 168.17 | 2392.85 | 140 (141) |
| Timor-Leste | 0.44 | 1779.64 | 171.84 | 1607.8 | 93 (97) |
| Togo | 0.41 | 1210.77 | 170.88 | 1039.89 | 65 (66) |
| Tokelau | 0.69 | 2361.24 | 168.25 | 2192.99 | 128 (129) |
| Tonga | 0.63 | 3556.67 | 168.03 | 3388.64 | 166 (167) |
| Trinidad and Tobago | 0.77 | 1451.36 | 168.11 | 1283.26 | 81 (82) |
| Tunisia | 0.68 | 3065.75 | 168.2 | 2897.55 | 152 (153) |
| Turkey | #N/A | 5661.35 | 168.05 | 5493.3 | 195 (196) |
| Turkmenistan | 0.68 | 1580.41 | 168.55 | 1411.86 | 84 (85) |
| Tuvalu | 0.58 | 2982.12 | 168.05 | 2814.06 | 149 (150) |
| Uganda | 0.42 | 709.03 | 171.2 | 537.83 | 20 (18) |
| Ukraine | 0.76 | 3991.62 | 169.14 | 3822.47 | 177 (178) |
| United Arab Emirates | 0.85 | 718.42 | 168.03 | 550.39 | 22 (20) |
| United Kingdom | 0.86 | 1999.61 | 168.72 | 1830.9 | 109 (110) |
| United Republic of Tanzania | 0.45 | 875.15 | 169.97 | 705.18 | 34 (33) |
| United States Virgin Islands | 0.82 | 1347.23 | 168.27 | 1178.96 | 76 (77) |
| United States of America | 0.86 | 2459 | 168.13 | 2290.87 | 134 (135) |
| Uruguay | 0.72 | 5028.9 | 168.33 | 4860.56 | 187 (188) |
| Uzbekistan | 0.66 | 1130.76 | 168.18 | 962.58 | 56 (57) |
| Vanuatu | 0.47 | 2360.1 | 171.19 | 2188.91 | 126 (128) |
| Venezuela (Bolivarian Republic of) | 0.6 | 2001.5 | 168.03 | 1833.47 | 110 (111) |
| Viet Nam | 0.63 | 3487.46 | 168.02 | 3319.44 | 164 (165) |
| Yemen | 0.45 | 1352.75 | 171.99 | 1180.76 | 77 (78) |
| Zambia | 0.51 | 1411.7 | 168.07 | 1243.63 | 78 (79) |
| Zimbabwe | 0.47 | 1669.35 | 170.19 | 1499.16 | 87 (88) |
| LOLC |  |  |  |  |  |
| Afghanistan | 0.34 | 1702.52 | 273.45 | 1429.07 | 47 (47) |
| Albania | 0.71 | 5068.52 | 273.11 | 4795.42 | 150 (150) |
| Algeria | 0.66 | 1375.25 | 273.29 | 1101.96 | 30 (28) |
| American Samoa | 0.72 | 5085.31 | 273.1 | 4812.21 | 151 (151) |
| Andorra | 0.87 | 5923.97 | 273.55 | 5650.42 | 170 (170) |
| Angola | 0.45 | 2595.99 | 273.54 | 2322.46 | 87 (87) |
| Antigua and Barbuda | 0.75 | 1855.38 | 273.55 | 1581.83 | 57 (57) |
| Argentina | 0.72 | 4174.61 | 273.09 | 3901.52 | 128 (128) |
| Armenia | 0.7 | 7193.97 | 273.34 | 6920.63 | 191 (191) |
| Australia | 0.84 | 4206.26 | 273.05 | 3933.21 | 129 (129) |
| Austria | 0.85 | 4254.62 | 273.09 | 3981.53 | 130 (130) |
| Azerbaijan | 0.69 | 2839.61 | 273.1 | 2566.51 | 91 (91) |
| Bahamas | 0.81 | 2612.07 | 273.85 | 2338.22 | 88 (88) |
| Bahrain | 0.75 | 5236.13 | 273.49 | 4962.64 | 154 (154) |
| Bangladesh | 0.49 | 885.89 | 273.42 | 612.47 | 10 (7) |
| Barbados | 0.75 | 1744.46 | 273.06 | 1471.41 | 49 (49) |
| Belarus | 0.78 | 5418.29 | 273.33 | 5144.96 | 162 (162) |
| Belgium | 0.85 | 6727.68 | 273.16 | 6454.52 | 185 (185) |
| Belize | 0.61 | 1983.58 | 273.31 | 1710.27 | 67 (66) |
| Benin | 0.37 | 1483.68 | 273.04 | 1210.64 | 36 (36) |
| Bermuda | 0.82 | 4943.3 | 273.2 | 4670.09 | 146 (146) |
| Bhutan | 0.47 | 940.3 | 273.07 | 667.22 | 13 (10) |
| Bolivia (Plurinational State of) | 0.6 | 2715.48 | 273.05 | 2442.43 | 89 (89) |
| Bosnia and Herzegovina | 0.72 | 7312.49 | 273.23 | 7039.25 | 194 (194) |
| Botswana | 0.64 | 3274.16 | 273.21 | 3000.95 | 102 (102) |
| Brazil | 0.65 | 2745.62 | 273.43 | 2472.19 | 90 (90) |
| Brunei Darussalam | 0.81 | 4905.28 | 273.04 | 4632.24 | 145 (145) |
| Bulgaria | 0.77 | 5844.06 | 273.05 | 5571.01 | 169 (169) |
| Burkina Faso | 0.29 | 1355.77 | 496.92 | 858.85 | 22 (27) |
| Burundi | 0.29 | 1056.45 | 496.81 | 559.64 | 9 (17) |
| Cabo Verde | 0.53 | 4801.55 | 273.43 | 4528.12 | 143 (143) |
| Cambodia | 0.47 | 6417.9 | 273.31 | 6144.59 | 182 (182) |
| Cameroon | 0.48 | 1801.02 | 273.54 | 1527.48 | 53 (53) |
| Canada | 0.87 | 5203.06 | 273.2 | 4929.87 | 152 (152) |
| Central African Republic | 0.31 | 2022.59 | 392.03 | 1630.56 | 63 (68) |
| Chad | 0.24 | 1475.84 | 518.01 | 957.83 | 27 (35) |
| Chile | 0.77 | 2915.77 | 273.07 | 2642.7 | 95 (95) |
| China | 0.72 | 8275.2 | 273.09 | 8002.11 | 198 (198) |
| Colombia | 0.66 | 2211.28 | 273.04 | 1938.24 | 76 (76) |
| Comoros | 0.48 | 1389.96 | 273.36 | 1116.6 | 31 (29) |
| Congo | 0.58 | 2512.11 | 273.16 | 2238.95 | 84 (84) |
| Cook Islands | 0.78 | 6727.93 | 273.05 | 6454.88 | 186 (186) |
| Costa Rica | 0.7 | 1520.62 | 273.18 | 1247.44 | 40 (39) |
| Croatia | 0.8 | 7036.05 | 273.05 | 6762.99 | 189 (189) |
| Cuba | 0.67 | 6036.82 | 273.49 | 5763.33 | 175 (175) |
| Cyprus | 0.84 | 4875.95 | 273.86 | 4602.09 | 144 (144) |
| Czechia | 0.83 | 6170.11 | 273.75 | 5896.36 | 178 (178) |
| Côte d'Ivoire | #N/A | 763.47 | 273.17 | 490.3 | 8 (5) |
| Democratic People's Republic of Korea | 0.57 | 4529.89 | 273.78 | 4256.11 | 136 (136) |
| Democratic Republic of the Congo | 0.38 | 1617.66 | 273.09 | 1344.57 | 44 (44) |
| Denmark | 0.9 | 5926.3 | 273.1 | 5653.19 | 171 (171) |
| Djibouti | 0.49 | 1945.28 | 273.29 | 1671.99 | 65 (64) |
| Dominica | 0.75 | 3896.4 | 273.09 | 3623.32 | 120 (120) |
| Dominican Republic | 0.62 | 2265.72 | 273.04 | 1992.68 | 78 (78) |
| Ecuador | 0.66 | 1498.78 | 273.8 | 1224.98 | 37 (37) |
| Egypt | 0.61 | 2548.07 | 273.29 | 2274.77 | 85 (85) |
| El Salvador | 0.56 | 1408.88 | 273.11 | 1135.78 | 32 (31) |
| Equatorial Guinea | 0.66 | 2904.76 | 273.06 | 2631.7 | 93 (93) |
| Eritrea | 0.4 | 1591.58 | 273.19 | 1318.39 | 43 (43) |
| Estonia | 0.84 | 6743.77 | 273.83 | 6469.94 | 187 (187) |
| Eswatini | 0.59 | 3686.07 | 273.19 | 3412.87 | 113 (113) |
| Ethiopia | 0.36 | 1449.01 | 273.06 | 1175.95 | 34 (33) |
| Fiji | 0.68 | 2094.51 | 273.17 | 1821.34 | 72 (72) |
| Finland | 0.86 | 4548.14 | 273.22 | 4274.92 | 138 (138) |
| France | 0.84 | 5383.27 | 273.19 | 5110.08 | 159 (159) |
| Gabon | 0.63 | 3210.63 | 273.06 | 2937.58 | 101 (101) |
| Gambia | 0.41 | 693.94 | 273.09 | 420.85 | 7 (4) |
| Georgia | 0.73 | 6165.94 | 273.09 | 5892.86 | 177 (177) |
| Germany | 0.9 | 5012.53 | 273.9 | 4738.63 | 148 (148) |
| Ghana | 0.56 | 1863.56 | 273.06 | 1590.5 | 58 (58) |
| Greece | 0.79 | 8536.82 | 273.67 | 8263.15 | 200 (200) |
| Greenland | 0.83 | 10580.81 | 273.21 | 10307.61 | 203 (203) |
| Grenada | 0.67 | 2472.68 | 273.06 | 2199.62 | 82 (82) |
| Guam | 0.8 | 4385.46 | 273.38 | 4112.08 | 133 (133) |
| Guatemala | 0.54 | 957.57 | 273.8 | 683.77 | 14 (11) |
| Guinea | 0.34 | 1753.04 | 273.07 | 1479.96 | 50 (50) |
| Guinea-Bissau | 0.35 | 1897.28 | 273.39 | 1623.89 | 62 (62) |
| Guyana | 0.65 | 1108.04 | 273.25 | 834.79 | 20 (20) |
| Haiti | 0.45 | 2115.45 | 273.16 | 1842.29 | 74 (74) |
| Honduras | 0.51 | 3192.79 | 273.09 | 2919.7 | 100 (100) |
| Hungary | 0.79 | 7257.34 | 273.48 | 6983.85 | 192 (192) |
| Iceland | 0.88 | 3189.03 | 273.04 | 2915.98 | 99 (99) |
| India | 0.58 | 991.43 | 273.3 | 718.13 | 15 (12) |
| Indonesia | 0.66 | 4544.92 | 273.12 | 4271.8 | 137 (137) |
| Iran (Islamic Republic of) | 0.7 | 1964.42 | 273.89 | 1690.52 | 66 (65) |
| Iraq | 0.66 | 4441.02 | 273.45 | 4167.57 | 134 (134) |
| Ireland | 0.87 | 4341.47 | 273.29 | 4068.18 | 132 (132) |
| Israel | 0.81 | 3771.29 | 273.3 | 3497.99 | 117 (117) |
| Italy | 0.81 | 5968.89 | 273.16 | 5695.72 | 174 (174) |
| Jamaica | 0.68 | 3284.88 | 273.25 | 3011.62 | 103 (103) |
| Japan | 0.87 | 5933 | 273.28 | 5659.73 | 173 (173) |
| Jordan | 0.73 | 2096.45 | 273.29 | 1823.16 | 73 (73) |
| Kazakhstan | 0.73 | 3555.96 | 273.25 | 3282.71 | 111 (111) |
| Kenya | 0.52 | 555.27 | 273.32 | 281.95 | 3 (2) |
| Kiribati | 0.53 | 4166.15 | 273.29 | 3892.86 | 127 (127) |
| Kuwait | 0.85 | 1878.21 | 273.72 | 1604.49 | 61 (61) |
| Kyrgyzstan | 0.6 | 2331.72 | 273.05 | 2058.67 | 80 (80) |
| Lao People's Democratic Republic | 0.49 | 4592.34 | 273.38 | 4318.97 | 139 (140) |
| Latvia | 0.83 | 6248.66 | 273.23 | 5975.42 | 180 (180) |
| Lebanon | 0.74 | 5230.72 | 273.17 | 4957.55 | 153 (153) |
| Lesotho | 0.51 | 4628.8 | 273.06 | 4355.74 | 141 (141) |
| Liberia | 0.35 | 1129.88 | 273.04 | 856.84 | 21 (21) |
| Libya | 0.73 | 6038.49 | 273.26 | 5765.23 | 176 (176) |
| Lithuania | 0.86 | 6463.33 | 273.78 | 6189.55 | 183 (183) |
| Luxembourg | 0.88 | 5425.41 | 273.42 | 5151.99 | 163 (163) |
| Madagascar | 0.4 | 925.23 | 273.21 | 652.02 | 12 (9) |
| Malawi | 0.38 | 596.06 | 273.5 | 322.56 | 5 (3) |
| Malaysia | 0.74 | 3452.74 | 273.32 | 3179.42 | 107 (107) |
| Maldives | 0.65 | 1787.61 | 273.07 | 1514.54 | 51 (51) |
| Mali | 0.27 | 878.68 | 502.33 | 376.35 | 6 (6) |
| Malta | 0.8 | 4107.75 | 273.17 | 3834.57 | 124 (124) |
| Marshall Islands | 0.57 | 5630.08 | 273.18 | 5356.89 | 165 (165) |
| Mauritania | 0.5 | 1516.14 | 273.92 | 1242.22 | 38 (38) |
| Mauritius | 0.72 | 2211.71 | 273.16 | 1938.55 | 77 (77) |
| Mexico | 0.66 | 1529.54 | 273.94 | 1255.6 | 42 (42) |
| Micronesia (Federated States of) | 0.59 | 6174.48 | 273.42 | 5901.06 | 179 (179) |
| Monaco | 0.91 | 11770.71 | 273.21 | 11497.5 | 204 (204) |
| Mongolia | 0.62 | 5396.27 | 273.37 | 5122.9 | 161 (161) |
| Montenegro | 0.8 | 10080.02 | 273.15 | 9806.86 | 202 (202) |
| Morocco | 0.56 | 3469.64 | 273.08 | 3196.56 | 108 (108) |
| Mozambique | 0.33 | 1528.34 | 282.35 | 1245.99 | 39 (41) |
| Myanmar | 0.53 | 3927.17 | 273.07 | 3654.09 | 121 (121) |
| Namibia | 0.62 | 1013.18 | 273.17 | 740.01 | 17 (14) |
| Nauru | 0.63 | 7874.96 | 273.74 | 7601.22 | 197 (197) |
| Nepal | 0.43 | 1023.86 | 273.06 | 750.8 | 18 (16) |
| Netherlands | 0.89 | 6410.84 | 273.06 | 6137.78 | 181 (181) |
| New Zealand | 0.85 | 3718.39 | 273.52 | 3444.87 | 114 (114) |
| Nicaragua | 0.52 | 923.81 | 273.07 | 650.74 | 11 (8) |
| Niger | 0.17 | 1016.86 | 720.86 | 296.01 | 4 (15) |
| Nigeria | 0.5 | 296.49 | 273.27 | 23.22 | 1 (1) |
| Niue | 0.73 | 6599.68 | 273.51 | 6326.17 | 184 (184) |
| North Macedonia | 0.75 | 5778.73 | 273.25 | 5505.48 | 167 (167) |
| Northern Mariana Islands | 0.77 | 8329.05 | 273.51 | 8055.54 | 199 (199) |
| Norway | 0.92 | 4333.76 | 273.04 | 4060.72 | 131 (131) |
| Oman | 0.77 | 1072.45 | 273.1 | 799.35 | 19 (19) |
| Pakistan | 0.5 | 2555.09 | 273.16 | 2281.93 | 86 (86) |
| Palau | 0.75 | 5296.93 | 273.15 | 5023.78 | 157 (157) |
| Palestine | 0.63 | 4476.48 | 273.53 | 4202.95 | 135 (135) |
| Panama | 0.71 | 1713.75 | 273.15 | 1440.6 | 48 (48) |
| Papua New Guinea | 0.42 | 3651.37 | 273.89 | 3377.48 | 112 (112) |
| Paraguay | 0.64 | 3725.25 | 273.78 | 3451.47 | 115 (115) |
| Peru | 0.66 | 1853.94 | 273.38 | 1580.56 | 56 (56) |
| Philippines | 0.65 | 3349.78 | 273.66 | 3076.13 | 105 (105) |
| Poland | 0.81 | 9024.97 | 273.14 | 8751.83 | 201 (201) |
| Portugal | 0.74 | 4110.12 | 273.16 | 3836.96 | 125 (125) |
| Puerto Rico | 0.83 | 1812.98 | 273.25 | 1539.73 | 54 (54) |
| Qatar | 0.85 | 2990.99 | 273.13 | 2717.86 | 96 (96) |
| Republic of Korea | 0.89 | 7130.45 | 274.1 | 6856.35 | 190 (190) |
| Republic of Moldova | 0.73 | 3336.55 | 273.05 | 3063.49 | 104 (104) |
| Romania | 0.77 | 5395.35 | 273.53 | 5121.82 | 160 (160) |
| Russian Federation | 0.81 | 5533.35 | 273.12 | 5260.24 | 164 (164) |
| Rwanda | 0.44 | 1629.53 | 273.29 | 1356.24 | 45 (45) |
| Saint Kitts and Nevis | 0.75 | 2068.17 | 273.09 | 1795.08 | 70 (70) |
| Saint Lucia | 0.67 | 2092.23 | 273.16 | 1819.07 | 71 (71) |
| Saint Vincent and the Grenadines | 0.64 | 1521.09 | 273.3 | 1247.79 | 41 (40) |
| Samoa | 0.59 | 1851.95 | 273.31 | 1578.65 | 55 (55) |
| San Marino | 0.89 | 3812.1 | 273.22 | 3538.88 | 118 (118) |
| Sao Tome and Principe | 0.51 | 4592.26 | 273.28 | 4318.98 | 140 (139) |
| Saudi Arabia | 0.82 | 1177.56 | 273.12 | 904.44 | 23 (22) |
| Senegal | 0.41 | 1866.28 | 273.06 | 1593.23 | 59 (59) |
| Serbia | 0.79 | 6966.89 | 273.11 | 6693.78 | 188 (188) |
| Seychelles | 0.73 | 3473.83 | 273.74 | 3200.1 | 109 (109) |
| Sierra Leone | 0.36 | 1322.86 | 273.26 | 1049.6 | 29 (26) |
| Singapore | 0.86 | 3761.11 | 273.07 | 3488.04 | 116 (116) |
| Slovakia | 0.81 | 5832.09 | 273.88 | 5558.21 | 168 (168) |
| Slovenia | 0.84 | 5932.4 | 273.21 | 5659.19 | 172 (172) |
| Solomon Islands | 0.43 | 3173.46 | 273.37 | 2900.09 | 98 (98) |
| Somalia | 0.08 | 1070.93 | 996.43 | 74.49 | 2 (18) |
| South Africa | 0.68 | 3535.39 | 273.42 | 3261.97 | 110 (110) |
| South Sudan | 0.28 | 1406.67 | 495.7 | 910.97 | 25 (30) |
| Spain | 0.77 | 5696.85 | 273.78 | 5423.07 | 166 (166) |
| Sri Lanka | 0.7 | 1452.59 | 273.1 | 1179.49 | 35 (34) |
| Sudan | 0.54 | 1657.22 | 273.42 | 1383.8 | 46 (46) |
| Suriname | 0.63 | 2278.65 | 273.36 | 2005.29 | 79 (79) |
| Sweden | 0.89 | 2905.34 | 273.17 | 2632.17 | 94 (94) |
| Switzerland | 0.93 | 3875.27 | 273.05 | 3602.22 | 119 (119) |
| Syrian Arab Republic | 0.62 | 2129.97 | 273.97 | 1856 | 75 (75) |
| Taiwan (Province of China) | 0.87 | 5378.11 | 273.36 | 5104.75 | 158 (158) |
| Tajikistan | 0.54 | 1299.72 | 273.06 | 1026.66 | 28 (25) |
| Thailand | 0.68 | 4956.43 | 273.25 | 4683.18 | 147 (147) |
| Timor-Leste | 0.44 | 2901.73 | 273.28 | 2628.45 | 92 (92) |
| Togo | 0.41 | 1931.5 | 273.04 | 1658.45 | 64 (63) |
| Tokelau | 0.69 | 4656.61 | 273.26 | 4383.34 | 142 (142) |
| Tonga | 0.63 | 7259.74 | 273.2 | 6986.54 | 193 (193) |
| Trinidad and Tobago | 0.77 | 1873.04 | 273.32 | 1599.72 | 60 (60) |
| Tunisia | 0.68 | 4078.18 | 273.05 | 3805.14 | 123 (123) |
| Turkey | #N/A | 7677.47 | 273.2 | 7404.27 | 196 (196) |
| Turkmenistan | 0.68 | 1440.34 | 273.33 | 1167.02 | 33 (32) |
| Tuvalu | 0.58 | 5049.68 | 273.69 | 4775.99 | 149 (149) |
| Uganda | 0.42 | 1008.29 | 273.26 | 735.03 | 16 (13) |
| Ukraine | 0.76 | 3428.41 | 273.99 | 3154.41 | 106 (106) |
| United Arab Emirates | 0.85 | 1991.63 | 273.21 | 1718.42 | 68 (67) |
| United Kingdom | 0.86 | 5253.78 | 273.15 | 4980.63 | 156 (156) |
| United Republic of Tanzania | 0.45 | 1215.05 | 273.05 | 942 | 26 (24) |
| United States Virgin Islands | 0.82 | 2493.49 | 273.88 | 2219.61 | 83 (83) |
| United States of America | 0.86 | 5242.95 | 273.04 | 4969.91 | 155 (155) |
| Uruguay | 0.72 | 7377.84 | 273.14 | 7104.7 | 195 (195) |
| Uzbekistan | 0.66 | 1180.03 | 273.16 | 906.87 | 24 (23) |
| Vanuatu | 0.47 | 3985.33 | 273.17 | 3712.16 | 122 (122) |
| Venezuela (Bolivarian Republic of) | 0.6 | 3153.03 | 273.1 | 2879.93 | 97 (97) |
| Viet Nam | 0.63 | 4152.55 | 273.13 | 3879.42 | 126 (126) |
| Yemen | 0.45 | 2059.52 | 273.16 | 1786.37 | 69 (69) |
| Zambia | 0.51 | 1789.13 | 273.29 | 1515.84 | 52 (52) |
| Zimbabwe | 0.47 | 2357.2 | 273.34 | 2083.86 | 81 (81) |

Notes: ASDR tares are reported per 100,000 population.

Abbreviations: DALYs: disability-adjusted life years; EOLC, early-onset lung cancer; MOLC, middle-onset lung cancer; LOLC, late-onset lung cancer; SDI: socio-demographic index; ASDR, age-standardized disability-adjusted life year rate; UI: uncertainty interval.

# **Supplementary Table S10.** Frontier DALYs, and effective difference of EOLC, MOLC and LOLC **in female** in 2021, by country or territory.

| Country/Territory | SDI | ASDR | Frontier DALYs | Effective difference | Effective difference rank (ASDR rank) |
| --- | --- | --- | --- | --- | --- |
| EOLC |  |  |  |  |  |
| Afghanistan | 0.34 | 97.84 | 3.81 | 94.03 | 161 (161) |
| Albania | 0.71 | 98.7 | 3.88 | 94.82 | 162 (162) |
| Algeria | 0.66 | 21.89 | 3.8 | 18.09 | 20 (19) |
| American Samoa | 0.72 | 124.65 | 3.87 | 120.78 | 183 (183) |
| Andorra | 0.87 | 39.81 | 3.81 | 36.01 | 50 (50) |
| Angola | 0.45 | 41.53 | 3.8 | 37.73 | 52 (52) |
| Antigua and Barbuda | 0.75 | 34.08 | 3.87 | 30.21 | 42 (42) |
| Argentina | 0.72 | 84.97 | 3.84 | 81.13 | 146 (146) |
| Armenia | 0.7 | 58.24 | 3.86 | 54.38 | 91 (91) |
| Australia | 0.84 | 75.43 | 3.85 | 71.58 | 127 (127) |
| Austria | 0.85 | 86.52 | 3.87 | 82.65 | 149 (149) |
| Azerbaijan | 0.69 | 58.07 | 3.85 | 54.22 | 89 (89) |
| Bahamas | 0.81 | 83.09 | 3.8 | 79.29 | 142 (142) |
| Bahrain | 0.75 | 77.93 | 3.81 | 74.11 | 130 (130) |
| Bangladesh | 0.49 | 32.06 | 3.8 | 28.26 | 36 (36) |
| Barbados | 0.75 | 40.08 | 3.8 | 36.28 | 51 (51) |
| Belarus | 0.78 | 47.57 | 3.86 | 43.71 | 65 (65) |
| Belgium | 0.85 | 108.26 | 3.86 | 104.39 | 170 (170) |
| Belize | 0.61 | 57.91 | 3.82 | 54.09 | 88 (88) |
| Benin | 0.37 | 19.71 | 3.8 | 15.91 | 13 (13) |
| Bermuda | 0.82 | 81.37 | 3.85 | 77.52 | 138 (138) |
| Bhutan | 0.47 | 36.05 | 3.89 | 32.15 | 44 (44) |
| Bolivia (Plurinational State of) | 0.6 | 82.66 | 3.81 | 78.85 | 140 (140) |
| Bosnia and Herzegovina | 0.72 | 113 | 3.81 | 109.19 | 173 (173) |
| Botswana | 0.64 | 54.39 | 3.86 | 50.52 | 76 (76) |
| Brazil | 0.65 | 83.75 | 3.86 | 79.89 | 144 (144) |
| Brunei Darussalam | 0.81 | 148.91 | 3.85 | 145.06 | 189 (189) |
| Bulgaria | 0.77 | 174.59 | 3.84 | 170.75 | 198 (198) |
| Burkina Faso | 0.29 | 17.38 | 5.7 | 11.68 | 8 (10) |
| Burundi | 0.29 | 21.69 | 5.7 | 15.99 | 15 (18) |
| Cabo Verde | 0.53 | 56.76 | 3.86 | 52.9 | 83 (83) |
| Cambodia | 0.47 | 102.53 | 3.81 | 98.72 | 166 (166) |
| Cameroon | 0.48 | 29.43 | 3.81 | 25.62 | 30 (30) |
| Canada | 0.87 | 77.77 | 3.81 | 73.96 | 129 (129) |
| Central African Republic | 0.31 | 31.28 | 3.8 | 27.48 | 35 (35) |
| Chad | 0.24 | 18.41 | 5.7 | 12.71 | 10 (12) |
| Chile | 0.77 | 58.07 | 3.85 | 54.22 | 90 (90) |
| China | 0.72 | 171.77 | 3.8 | 167.97 | 197 (197) |
| Colombia | 0.66 | 57.58 | 3.86 | 53.73 | 87 (87) |
| Comoros | 0.48 | 39.11 | 3.86 | 35.25 | 48 (48) |
| Congo | 0.58 | 67.93 | 3.87 | 64.06 | 113 (113) |
| Cook Islands | 0.78 | 64.41 | 3.83 | 60.58 | 107 (107) |
| Costa Rica | 0.7 | 41.84 | 3.8 | 38.04 | 54 (54) |
| Croatia | 0.8 | 112.05 | 3.8 | 108.24 | 172 (172) |
| Cuba | 0.67 | 109.08 | 3.8 | 105.28 | 171 (171) |
| Cyprus | 0.84 | 44.17 | 3.83 | 40.34 | 59 (59) |
| Czechia | 0.83 | 89.32 | 3.82 | 85.5 | 152 (152) |
| Côte d'Ivoire | #N/A | 14.78 | 3.82 | 10.96 | 7 (6) |
| Democratic People's Republic of Korea | 0.57 | 162.42 | 3.85 | 158.57 | 194 (194) |
| Democratic Republic of the Congo | 0.38 | 28.73 | 3.81 | 24.92 | 27 (27) |
| Denmark | 0.9 | 121.59 | 3.8 | 117.79 | 179 (179) |
| Djibouti | 0.49 | 37 | 3.8 | 33.2 | 46 (46) |
| Dominica | 0.75 | 74.58 | 3.87 | 70.72 | 126 (126) |
| Dominican Republic | 0.62 | 100.45 | 3.8 | 96.65 | 165 (165) |
| Ecuador | 0.66 | 56.09 | 3.88 | 52.21 | 81 (81) |
| Egypt | 0.61 | 78.46 | 3.86 | 74.61 | 132 (132) |
| El Salvador | 0.56 | 70.96 | 3.8 | 67.15 | 118 (118) |
| Equatorial Guinea | 0.66 | 65.06 | 3.8 | 61.26 | 108 (108) |
| Eritrea | 0.4 | 41.64 | 3.8 | 37.84 | 53 (53) |
| Estonia | 0.84 | 50.52 | 3.83 | 46.7 | 69 (69) |
| Eswatini | 0.59 | 82.84 | 3.8 | 79.03 | 141 (141) |
| Ethiopia | 0.36 | 21.02 | 3.8 | 17.22 | 18 (17) |
| Fiji | 0.68 | 46.26 | 3.8 | 42.46 | 61 (61) |
| Finland | 0.86 | 72.44 | 3.81 | 68.62 | 120 (120) |
| France | 0.84 | 128.48 | 3.82 | 124.67 | 184 (184) |
| Gabon | 0.63 | 59.02 | 3.81 | 55.21 | 96 (95) |
| Gambia | 0.41 | 9.01 | 3.86 | 5.15 | 5 (3) |
| Georgia | 0.73 | 58.43 | 3.8 | 54.63 | 93 (93) |
| Germany | 0.9 | 91.79 | 3.81 | 87.98 | 155 (155) |
| Ghana | 0.56 | 16.15 | 3.87 | 12.28 | 9 (7) |
| Greece | 0.79 | 95.69 | 3.8 | 91.89 | 159 (159) |
| Greenland | 0.83 | 325.58 | 3.88 | 321.7 | 203 (203) |
| Grenada | 0.67 | 54.62 | 3.87 | 50.75 | 77 (77) |
| Guam | 0.8 | 160.89 | 3.81 | 157.08 | 193 (193) |
| Guatemala | 0.54 | 52.81 | 3.88 | 48.92 | 73 (73) |
| Guinea | 0.34 | 20.96 | 3.88 | 17.08 | 17 (16) |
| Guinea-Bissau | 0.35 | 33.92 | 3.8 | 30.12 | 41 (41) |
| Guyana | 0.65 | 55.22 | 3.81 | 51.41 | 78 (78) |
| Haiti | 0.45 | 59.3 | 3.84 | 55.46 | 97 (97) |
| Honduras | 0.51 | 151.32 | 3.85 | 147.46 | 190 (190) |
| Hungary | 0.79 | 152.77 | 3.85 | 148.92 | 191 (191) |
| Iceland | 0.88 | 221.79 | 3.81 | 217.99 | 201 (201) |
| India | 0.58 | 42.89 | 3.84 | 39.05 | 56 (56) |
| Indonesia | 0.66 | 122.22 | 3.8 | 118.42 | 180 (180) |
| Iran (Islamic Republic of) | 0.7 | 63.78 | 3.81 | 59.97 | 104 (104) |
| Iraq | 0.66 | 69.97 | 3.8 | 66.17 | 115 (115) |
| Ireland | 0.87 | 121.51 | 3.86 | 117.65 | 178 (178) |
| Israel | 0.81 | 56.47 | 3.87 | 52.6 | 82 (82) |
| Italy | 0.81 | 74.02 | 3.8 | 70.22 | 124 (124) |
| Jamaica | 0.68 | 73.56 | 3.86 | 69.7 | 123 (123) |
| Japan | 0.87 | 46.29 | 3.82 | 42.47 | 62 (62) |
| Jordan | 0.73 | 38.32 | 3.8 | 34.52 | 47 (47) |
| Kazakhstan | 0.73 | 60.12 | 3.8 | 56.32 | 99 (99) |
| Kenya | 0.52 | 19.79 | 3.86 | 15.93 | 14 (14) |
| Kiribati | 0.53 | 47 | 3.88 | 43.13 | 63 (63) |
| Kuwait | 0.85 | 27.67 | 3.84 | 23.83 | 26 (26) |
| Kyrgyzstan | 0.6 | 53.51 | 3.87 | 49.63 | 75 (75) |
| Lao People's Democratic Republic | 0.49 | 106.92 | 3.83 | 103.09 | 168 (168) |
| Latvia | 0.83 | 57.19 | 3.8 | 53.39 | 84 (84) |
| Lebanon | 0.74 | 89.93 | 3.86 | 86.07 | 153 (153) |
| Lesotho | 0.51 | 63.92 | 3.88 | 60.05 | 105 (105) |
| Liberia | 0.35 | 20.86 | 3.81 | 17.05 | 16 (15) |
| Libya | 0.73 | 50.04 | 3.84 | 46.2 | 68 (68) |
| Lithuania | 0.86 | 59.96 | 3.88 | 56.08 | 98 (98) |
| Luxembourg | 0.88 | 88.18 | 3.8 | 84.38 | 150 (150) |
| Madagascar | 0.4 | 32.59 | 3.86 | 28.73 | 38 (38) |
| Malawi | 0.38 | 6.69 | 3.88 | 2.82 | 4 (2) |
| Malaysia | 0.74 | 81.21 | 3.88 | 77.33 | 137 (137) |
| Maldives | 0.65 | 29.88 | 3.8 | 26.07 | 32 (32) |
| Mali | 0.27 | 23.33 | 5.7 | 17.63 | 19 (21) |
| Malta | 0.8 | 66.3 | 3.83 | 62.47 | 111 (111) |
| Marshall Islands | 0.57 | 121.22 | 3.88 | 117.34 | 177 (177) |
| Mauritania | 0.5 | 27.42 | 3.82 | 23.6 | 25 (25) |
| Mauritius | 0.72 | 55.71 | 3.81 | 51.9 | 80 (80) |
| Mexico | 0.66 | 43.1 | 3.81 | 39.29 | 58 (58) |
| Micronesia (Federated States of) | 0.59 | 129.67 | 3.85 | 125.82 | 186 (186) |
| Monaco | 0.91 | 383.56 | 3.87 | 379.69 | 204 (204) |
| Mongolia | 0.62 | 90.68 | 3.81 | 86.87 | 154 (154) |
| Montenegro | 0.8 | 182.63 | 3.88 | 178.75 | 199 (199) |
| Morocco | 0.56 | 16.93 | 3.87 | 13.06 | 11 (9) |
| Mozambique | 0.33 | 17.43 | 3.86 | 13.57 | 12 (11) |
| Myanmar | 0.53 | 107.14 | 3.81 | 103.33 | 169 (169) |
| Namibia | 0.62 | 29.41 | 3.86 | 25.55 | 29 (29) |
| Nauru | 0.63 | 200.51 | 3.81 | 196.7 | 200 (200) |
| Nepal | 0.43 | 36.47 | 3.8 | 32.67 | 45 (45) |
| Netherlands | 0.89 | 123.32 | 3.85 | 119.47 | 182 (182) |
| New Zealand | 0.85 | 117.93 | 3.81 | 114.11 | 175 (175) |
| Nicaragua | 0.52 | 32.55 | 3.85 | 28.7 | 37 (37) |
| Niger | 0.17 | 11.9 | 10.54 | 1.36 | 2 (5) |
| Nigeria | 0.5 | 5.36 | 3.8 | 1.55 | 3 (1) |
| Niue | 0.73 | 123.27 | 3.86 | 119.41 | 181 (181) |
| North Macedonia | 0.75 | 100.26 | 3.85 | 96.41 | 164 (164) |
| Northern Mariana Islands | 0.77 | 113.44 | 3.87 | 109.57 | 174 (174) |
| Norway | 0.92 | 85.98 | 3.87 | 82.12 | 147 (147) |
| Oman | 0.77 | 10.6 | 3.8 | 6.8 | 6 (4) |
| Pakistan | 0.5 | 42.62 | 3.87 | 38.75 | 55 (55) |
| Palau | 0.75 | 232.83 | 3.85 | 228.98 | 202 (202) |
| Palestine | 0.63 | 57.31 | 3.82 | 53.49 | 85 (85) |
| Panama | 0.71 | 49.4 | 3.83 | 45.57 | 67 (67) |
| Papua New Guinea | 0.42 | 70.63 | 3.8 | 66.83 | 117 (117) |
| Paraguay | 0.64 | 57.54 | 3.82 | 53.72 | 86 (86) |
| Peru | 0.66 | 83.54 | 3.89 | 79.65 | 143 (143) |
| Philippines | 0.65 | 99.8 | 3.85 | 95.95 | 163 (163) |
| Poland | 0.81 | 94.69 | 3.8 | 90.88 | 158 (158) |
| Portugal | 0.74 | 70.39 | 3.83 | 66.56 | 116 (116) |
| Puerto Rico | 0.83 | 51.86 | 3.85 | 48.01 | 70 (71) |
| Qatar | 0.85 | 30.82 | 3.84 | 26.99 | 33 (33) |
| Republic of Korea | 0.89 | 66.14 | 3.85 | 62.29 | 110 (110) |
| Republic of Moldova | 0.73 | 53.1 | 3.8 | 49.3 | 74 (74) |
| Romania | 0.77 | 137.82 | 3.83 | 133.99 | 187 (187) |
| Russian Federation | 0.81 | 65.41 | 3.85 | 61.56 | 109 (109) |
| Rwanda | 0.44 | 30.89 | 3.8 | 27.09 | 34 (34) |
| Saint Kitts and Nevis | 0.75 | 33.46 | 3.83 | 29.62 | 40 (40) |
| Saint Lucia | 0.67 | 63.65 | 3.82 | 59.83 | 103 (103) |
| Saint Vincent and the Grenadines | 0.64 | 75.61 | 3.8 | 71.81 | 128 (128) |
| Samoa | 0.59 | 29.51 | 3.87 | 25.65 | 31 (31) |
| San Marino | 0.89 | 71.55 | 3.8 | 67.75 | 119 (119) |
| Sao Tome and Principe | 0.51 | 61.37 | 3.84 | 57.53 | 102 (102) |
| Saudi Arabia | 0.82 | 34.51 | 3.89 | 30.62 | 43 (43) |
| Senegal | 0.41 | 26.3 | 3.8 | 22.5 | 24 (24) |
| Serbia | 0.79 | 155.55 | 3.8 | 151.75 | 192 (192) |
| Seychelles | 0.73 | 61.15 | 3.88 | 57.27 | 101 (101) |
| Sierra Leone | 0.36 | 22.29 | 3.83 | 18.46 | 21 (20) |
| Singapore | 0.86 | 51.82 | 3.8 | 48.02 | 71 (70) |
| Slovakia | 0.81 | 78.45 | 3.87 | 74.58 | 131 (131) |
| Slovenia | 0.84 | 78.96 | 3.8 | 75.16 | 134 (134) |
| Solomon Islands | 0.43 | 167.36 | 3.83 | 163.53 | 196 (196) |
| Somalia | 0.08 | 16.51 | 16.51 | 0 | 1 (8) |
| South Africa | 0.68 | 74.26 | 3.81 | 70.45 | 125 (125) |
| South Sudan | 0.28 | 25.86 | 5.7 | 20.16 | 22 (23) |
| Spain | 0.77 | 80.82 | 3.87 | 76.95 | 136 (136) |
| Sri Lanka | 0.7 | 39.67 | 3.85 | 35.81 | 49 (49) |
| Sudan | 0.54 | 55.61 | 3.81 | 51.8 | 79 (79) |
| Suriname | 0.63 | 84.2 | 3.81 | 80.39 | 145 (145) |
| Sweden | 0.89 | 64.26 | 3.85 | 60.4 | 106 (106) |
| Switzerland | 0.93 | 72.73 | 3.81 | 68.92 | 122 (122) |
| Syrian Arab Republic | 0.62 | 72.61 | 3.89 | 68.72 | 121 (121) |
| Taiwan (Province of China) | 0.87 | 145.73 | 3.84 | 141.89 | 188 (188) |
| Tajikistan | 0.54 | 61.11 | 3.84 | 57.27 | 100 (100) |
| Thailand | 0.68 | 163.1 | 3.88 | 159.23 | 195 (195) |
| Timor-Leste | 0.44 | 66.73 | 3.85 | 62.88 | 112 (112) |
| Togo | 0.41 | 24.88 | 3.8 | 21.08 | 23 (22) |
| Tokelau | 0.69 | 128.54 | 3.87 | 124.67 | 185 (185) |
| Tonga | 0.63 | 80.54 | 3.8 | 76.73 | 135 (135) |
| Trinidad and Tobago | 0.77 | 47.5 | 3.85 | 43.65 | 64 (64) |
| Tunisia | 0.68 | 29.35 | 3.82 | 25.53 | 28 (28) |
| Turkey | #N/A | 96.41 | 3.81 | 92.6 | 160 (160) |
| Turkmenistan | 0.68 | 59.04 | 3.85 | 55.18 | 95 (96) |
| Tuvalu | 0.58 | 103.99 | 3.81 | 100.17 | 167 (167) |
| Uganda | 0.42 | 43.01 | 3.86 | 39.14 | 57 (57) |
| Ukraine | 0.76 | 69.94 | 3.81 | 66.13 | 114 (114) |
| United Arab Emirates | 0.85 | 49.14 | 3.86 | 45.28 | 66 (66) |
| United Kingdom | 0.86 | 86.22 | 3.8 | 82.42 | 148 (148) |
| United Republic of Tanzania | 0.45 | 32.62 | 3.81 | 28.82 | 39 (39) |
| United States Virgin Islands | 0.82 | 78.9 | 3.81 | 75.09 | 133 (133) |
| United States of America | 0.86 | 82.63 | 3.81 | 78.82 | 139 (139) |
| Uruguay | 0.72 | 118.04 | 3.85 | 114.2 | 176 (176) |
| Uzbekistan | 0.66 | 51.87 | 3.81 | 48.06 | 72 (72) |
| Vanuatu | 0.47 | 58.37 | 3.8 | 54.57 | 92 (92) |
| Venezuela (Bolivarian Republic of) | 0.6 | 92.21 | 3.81 | 88.4 | 156 (156) |
| Viet Nam | 0.63 | 93.18 | 3.87 | 89.31 | 157 (157) |
| Yemen | 0.45 | 44.56 | 3.81 | 40.75 | 60 (60) |
| Zambia | 0.51 | 58.96 | 3.81 | 55.15 | 94 (94) |
| Zimbabwe | 0.47 | 88.86 | 3.81 | 85.05 | 151 (151) |
| MOLC |  |  |  |  |  |
| Afghanistan | 0.34 | 665.93 | 51.41 | 614.53 | 110 (110) |
| Albania | 0.71 | 841.35 | 51.34 | 790.01 | 133 (133) |
| Algeria | 0.66 | 191.02 | 51.43 | 139.59 | 8 (7) |
| American Samoa | 0.72 | 1434.99 | 51.25 | 1383.74 | 170 (170) |
| Andorra | 0.87 | 439.52 | 51.47 | 388.05 | 60 (60) |
| Angola | 0.45 | 506.12 | 51.44 | 454.68 | 77 (77) |
| Antigua and Barbuda | 0.75 | 475.86 | 51.24 | 424.61 | 71 (71) |
| Argentina | 0.72 | 1181.41 | 51.48 | 1129.93 | 157 (157) |
| Armenia | 0.7 | 676.15 | 51.27 | 624.88 | 111 (111) |
| Australia | 0.84 | 1245.08 | 51.34 | 1193.74 | 161 (161) |
| Austria | 0.85 | 1548.42 | 51.37 | 1497.05 | 178 (178) |
| Azerbaijan | 0.69 | 517.19 | 51.29 | 465.9 | 79 (78) |
| Bahamas | 0.81 | 665.7 | 51.28 | 614.42 | 109 (109) |
| Bahrain | 0.75 | 771.72 | 51.3 | 720.42 | 123 (123) |
| Bangladesh | 0.49 | 285.57 | 51.39 | 234.17 | 19 (19) |
| Barbados | 0.75 | 455.29 | 51.29 | 404 | 65 (65) |
| Belarus | 0.78 | 427.27 | 51.48 | 375.79 | 55 (55) |
| Belgium | 0.85 | 1725.48 | 51.31 | 1674.17 | 187 (187) |
| Belize | 0.61 | 656.48 | 51.34 | 605.14 | 105 (105) |
| Benin | 0.37 | 268.97 | 51.3 | 217.67 | 17 (16) |
| Bermuda | 0.82 | 892.75 | 51.27 | 841.47 | 136 (136) |
| Bhutan | 0.47 | 322.89 | 51.3 | 271.59 | 31 (30) |
| Bolivia (Plurinational State of) | 0.6 | 830.53 | 51.32 | 779.22 | 131 (131) |
| Bosnia and Herzegovina | 0.72 | 1500.39 | 51.44 | 1448.96 | 176 (176) |
| Botswana | 0.64 | 720.35 | 51.52 | 668.83 | 113 (113) |
| Brazil | 0.65 | 1038.17 | 51.27 | 986.9 | 147 (147) |
| Brunei Darussalam | 0.81 | 1452.02 | 51.5 | 1400.52 | 172 (172) |
| Bulgaria | 0.77 | 1641.82 | 51.32 | 1590.5 | 184 (184) |
| Burkina Faso | 0.29 | 224.73 | 66 | 158.73 | 11 (11) |
| Burundi | 0.29 | 250.25 | 66.12 | 184.12 | 14 (15) |
| Cabo Verde | 0.53 | 650.78 | 51.42 | 599.36 | 103 (103) |
| Cambodia | 0.47 | 1007.82 | 51.54 | 956.28 | 144 (144) |
| Cameroon | 0.48 | 360.44 | 51.44 | 309 | 39 (39) |
| Canada | 0.87 | 1745.49 | 51.3 | 1694.2 | 188 (188) |
| Central African Republic | 0.31 | 376.09 | 53.08 | 323.01 | 42 (42) |
| Chad | 0.24 | 276.14 | 65.97 | 210.18 | 16 (18) |
| Chile | 0.77 | 785.13 | 51.27 | 733.86 | 124 (124) |
| China | 0.72 | 1662.16 | 51.27 | 1610.89 | 185 (185) |
| Colombia | 0.66 | 596.59 | 51.31 | 545.28 | 91 (91) |
| Comoros | 0.48 | 419.94 | 51.42 | 368.52 | 52 (52) |
| Congo | 0.58 | 752.23 | 51.48 | 700.75 | 117 (117) |
| Cook Islands | 0.78 | 817.6 | 51.26 | 766.34 | 128 (128) |
| Costa Rica | 0.7 | 390.52 | 51.4 | 339.12 | 47 (47) |
| Croatia | 0.8 | 1669.32 | 51.32 | 1617.99 | 186 (186) |
| Cuba | 0.67 | 1954.18 | 51.45 | 1902.73 | 193 (193) |
| Cyprus | 0.84 | 661.83 | 51.47 | 610.36 | 106 (106) |
| Czechia | 0.83 | 1453.63 | 51.28 | 1402.35 | 173 (173) |
| Côte d'Ivoire | #N/A | 206.08 | 51.25 | 154.83 | 10 (9) |
| Democratic People's Republic of Korea | 0.57 | 1286.14 | 51.26 | 1234.89 | 163 (163) |
| Democratic Republic of the Congo | 0.38 | 391.53 | 51.49 | 340.04 | 48 (48) |
| Denmark | 0.9 | 2298.33 | 51.49 | 2246.84 | 198 (198) |
| Djibouti | 0.49 | 461.64 | 51.4 | 410.23 | 68 (68) |
| Dominica | 0.75 | 809.61 | 51.31 | 758.3 | 127 (127) |
| Dominican Republic | 0.62 | 796.8 | 51.52 | 745.28 | 125 (125) |
| Ecuador | 0.66 | 468.28 | 51.29 | 416.98 | 69 (69) |
| Egypt | 0.61 | 576.42 | 51.43 | 524.99 | 90 (90) |
| El Salvador | 0.56 | 605.75 | 51.45 | 554.3 | 93 (93) |
| Equatorial Guinea | 0.66 | 751.21 | 51.45 | 699.75 | 116 (116) |
| Eritrea | 0.4 | 442.47 | 51.4 | 391.08 | 63 (63) |
| Estonia | 0.84 | 648.06 | 51.33 | 596.74 | 102 (102) |
| Eswatini | 0.59 | 991.4 | 51.42 | 939.97 | 142 (142) |
| Ethiopia | 0.36 | 238.2 | 51.53 | 186.67 | 15 (14) |
| Fiji | 0.68 | 567.99 | 51.4 | 516.59 | 88 (88) |
| Finland | 0.86 | 970.38 | 51.38 | 918.99 | 139 (139) |
| France | 0.84 | 1638.75 | 51.38 | 1587.37 | 183 (183) |
| Gabon | 0.63 | 693.51 | 51.35 | 642.16 | 112 (112) |
| Gambia | 0.41 | 131.98 | 51.31 | 80.68 | 5 (3) |
| Georgia | 0.73 | 498.37 | 51.43 | 446.95 | 75 (75) |
| Germany | 0.9 | 1758.09 | 51.31 | 1706.78 | 190 (190) |
| Ghana | 0.56 | 183.34 | 51.41 | 131.92 | 6 (5) |
| Greece | 0.79 | 1385.4 | 51.41 | 1333.99 | 168 (168) |
| Greenland | 0.83 | 4570.3 | 51.3 | 4519 | 204 (204) |
| Grenada | 0.67 | 605.93 | 51.33 | 554.59 | 94 (94) |
| Guam | 0.8 | 1831.66 | 51.28 | 1780.38 | 192 (192) |
| Guatemala | 0.54 | 404.35 | 51.26 | 353.09 | 50 (50) |
| Guinea | 0.34 | 291.06 | 51.39 | 239.67 | 21 (21) |
| Guinea-Bissau | 0.35 | 379.44 | 51.41 | 328.03 | 44 (44) |
| Guyana | 0.65 | 546.91 | 51.3 | 495.62 | 84 (84) |
| Haiti | 0.45 | 621.52 | 51.35 | 570.17 | 97 (97) |
| Honduras | 0.51 | 1587.68 | 51.43 | 1536.25 | 181 (181) |
| Hungary | 0.79 | 3442.11 | 51.29 | 3390.82 | 202 (202) |
| Iceland | 0.88 | 2131.2 | 51.51 | 2079.69 | 195 (195) |
| India | 0.58 | 342.77 | 51.36 | 291.41 | 34 (34) |
| Indonesia | 0.66 | 1110.73 | 51.25 | 1059.47 | 154 (154) |
| Iran (Islamic Republic of) | 0.7 | 430.31 | 51.54 | 378.76 | 56 (56) |
| Iraq | 0.66 | 756.54 | 51.48 | 705.07 | 119 (119) |
| Ireland | 0.87 | 1563.57 | 51.27 | 1512.3 | 179 (179) |
| Israel | 0.81 | 803.69 | 51.33 | 752.36 | 126 (126) |
| Italy | 0.81 | 1101.96 | 51.31 | 1050.65 | 153 (153) |
| Jamaica | 0.68 | 663.74 | 51.27 | 612.47 | 107 (107) |
| Japan | 0.87 | 546.18 | 51.27 | 494.91 | 82 (82) |
| Jordan | 0.73 | 364.53 | 51.43 | 313.1 | 40 (40) |
| Kazakhstan | 0.73 | 455.37 | 51.24 | 404.13 | 66 (66) |
| Kenya | 0.52 | 227.47 | 51.49 | 175.98 | 12 (12) |
| Kiribati | 0.53 | 565.83 | 51.37 | 514.46 | 86 (86) |
| Kuwait | 0.85 | 228.22 | 51.32 | 176.9 | 13 (13) |
| Kyrgyzstan | 0.6 | 439.91 | 51.26 | 388.66 | 62 (62) |
| Lao People's Democratic Republic | 0.49 | 996.25 | 51.3 | 944.95 | 143 (143) |
| Latvia | 0.83 | 731.75 | 51.26 | 680.49 | 114 (114) |
| Lebanon | 0.74 | 971.94 | 51.5 | 920.44 | 140 (140) |
| Lesotho | 0.51 | 824.21 | 51.31 | 772.89 | 130 (130) |
| Liberia | 0.35 | 270.77 | 51.32 | 219.45 | 18 (17) |
| Libya | 0.73 | 527.65 | 51.46 | 476.19 | 80 (80) |
| Lithuania | 0.86 | 653.43 | 51.32 | 602.11 | 104 (104) |
| Luxembourg | 0.88 | 1327.98 | 51.26 | 1276.72 | 166 (166) |
| Madagascar | 0.4 | 327.71 | 51.24 | 276.46 | 32 (32) |
| Malawi | 0.38 | 78.82 | 51.32 | 27.5 | 3 (1) |
| Malaysia | 0.74 | 860.28 | 51.38 | 808.9 | 135 (135) |
| Maldives | 0.65 | 298.09 | 51.54 | 246.54 | 23 (23) |
| Mali | 0.27 | 317.28 | 66.35 | 250.93 | 25 (26) |
| Malta | 0.8 | 850.46 | 51.32 | 799.14 | 134 (134) |
| Marshall Islands | 0.57 | 1277.45 | 51.53 | 1225.92 | 162 (162) |
| Mauritania | 0.5 | 420.65 | 51.54 | 369.12 | 53 (53) |
| Mauritius | 0.72 | 437.34 | 51.46 | 385.89 | 58 (58) |
| Mexico | 0.66 | 399.65 | 51.26 | 348.39 | 49 (49) |
| Micronesia (Federated States of) | 0.59 | 1477.99 | 51.37 | 1426.62 | 174 (174) |
| Monaco | 0.91 | 3533.46 | 51.41 | 3482.05 | 203 (203) |
| Mongolia | 0.62 | 648.03 | 51.35 | 596.68 | 101 (101) |
| Montenegro | 0.8 | 2116.54 | 51.49 | 2065.04 | 194 (194) |
| Morocco | 0.56 | 204.98 | 51.28 | 153.7 | 9 (8) |
| Mozambique | 0.33 | 297.26 | 51.35 | 245.91 | 22 (22) |
| Myanmar | 0.53 | 972.38 | 51.27 | 921.11 | 141 (141) |
| Namibia | 0.62 | 354.09 | 51.41 | 302.68 | 36 (36) |
| Nauru | 0.63 | 2161.5 | 51.27 | 2110.23 | 196 (196) |
| Nepal | 0.43 | 322.25 | 51.45 | 270.8 | 30 (29) |
| Netherlands | 0.89 | 2306.58 | 51.29 | 2255.29 | 199 (199) |
| New Zealand | 0.85 | 1771.13 | 51.27 | 1719.86 | 191 (191) |
| Nicaragua | 0.52 | 332.83 | 51.4 | 281.43 | 33 (33) |
| Niger | 0.17 | 180.92 | 157.83 | 23.09 | 2 (4) |
| Nigeria | 0.5 | 84.76 | 51.38 | 33.38 | 4 (2) |
| Niue | 0.73 | 1320.22 | 51.31 | 1268.91 | 165 (165) |
| North Macedonia | 0.75 | 1200.06 | 51.25 | 1148.81 | 158 (158) |
| Northern Mariana Islands | 0.77 | 1567.64 | 51.51 | 1516.13 | 180 (180) |
| Norway | 0.92 | 1497.11 | 51.31 | 1445.8 | 175 (175) |
| Oman | 0.77 | 187.19 | 51.27 | 135.92 | 7 (6) |
| Pakistan | 0.5 | 439.59 | 51.42 | 388.17 | 61 (61) |
| Palau | 0.75 | 2781.66 | 51.36 | 2730.3 | 201 (201) |
| Palestine | 0.63 | 531.94 | 51.29 | 480.65 | 81 (81) |
| Panama | 0.71 | 437.51 | 51.26 | 386.26 | 59 (59) |
| Papua New Guinea | 0.42 | 956.42 | 51.25 | 905.17 | 138 (138) |
| Paraguay | 0.64 | 598.92 | 51.46 | 547.46 | 92 (92) |
| Peru | 0.66 | 741.69 | 51.42 | 690.27 | 115 (115) |
| Philippines | 0.65 | 820.75 | 51.51 | 769.23 | 129 (129) |
| Poland | 0.81 | 2278.95 | 51.46 | 2227.49 | 197 (197) |
| Portugal | 0.74 | 769.83 | 51.48 | 718.34 | 122 (122) |
| Puerto Rico | 0.83 | 499.53 | 51.31 | 448.21 | 76 (76) |
| Qatar | 0.85 | 458.13 | 51.31 | 406.82 | 67 (67) |
| Republic of Korea | 0.89 | 546.84 | 51.55 | 495.29 | 83 (83) |
| Republic of Moldova | 0.73 | 558.25 | 51.53 | 506.72 | 85 (85) |
| Romania | 0.77 | 1427.35 | 51.26 | 1376.09 | 169 (169) |
| Russian Federation | 0.81 | 641.33 | 51.5 | 589.83 | 99 (99) |
| Rwanda | 0.44 | 385.5 | 51.55 | 333.96 | 45 (45) |
| Saint Kitts and Nevis | 0.75 | 492.22 | 51.4 | 440.81 | 73 (73) |
| Saint Lucia | 0.67 | 571.78 | 51.41 | 520.37 | 89 (89) |
| Saint Vincent and the Grenadines | 0.64 | 567.36 | 51.28 | 516.08 | 87 (87) |
| Samoa | 0.59 | 320.87 | 51.28 | 269.59 | 29 (28) |
| San Marino | 0.89 | 768.72 | 51.28 | 717.45 | 121 (121) |
| Sao Tome and Principe | 0.51 | 632.26 | 51.35 | 580.91 | 98 (98) |
| Saudi Arabia | 0.82 | 317.95 | 51.27 | 266.68 | 28 (27) |
| Senegal | 0.41 | 351.72 | 51.49 | 300.22 | 35 (35) |
| Serbia | 0.79 | 2364.62 | 51.26 | 2313.35 | 200 (200) |
| Seychelles | 0.73 | 469.29 | 51.46 | 417.84 | 70 (70) |
| Sierra Leone | 0.36 | 287.1 | 51.31 | 235.78 | 20 (20) |
| Singapore | 0.86 | 619.99 | 51.27 | 568.72 | 96 (96) |
| Slovakia | 0.81 | 1049.41 | 51.39 | 998.02 | 148 (148) |
| Slovenia | 0.84 | 1503.65 | 51.32 | 1452.33 | 177 (177) |
| Solomon Islands | 0.43 | 1037.87 | 51.26 | 986.61 | 146 (146) |
| Somalia | 0.08 | 208.98 | 208.98 | 0 | 1 (10) |
| South Africa | 0.68 | 1012.51 | 51.24 | 961.27 | 145 (145) |
| South Sudan | 0.28 | 324.98 | 65.97 | 259.01 | 26 (31) |
| Spain | 0.77 | 1151.19 | 51.31 | 1099.88 | 156 (156) |
| Sri Lanka | 0.7 | 356.59 | 51.28 | 305.32 | 37 (37) |
| Sudan | 0.54 | 390.17 | 51.41 | 338.76 | 46 (46) |
| Suriname | 0.63 | 755.53 | 51.28 | 704.26 | 118 (118) |
| Sweden | 0.89 | 1139.23 | 51.4 | 1087.83 | 155 (155) |
| Switzerland | 0.93 | 1234.68 | 51.26 | 1183.42 | 160 (160) |
| Syrian Arab Republic | 0.62 | 477.27 | 51.55 | 425.72 | 72 (72) |
| Taiwan (Province of China) | 0.87 | 1311.35 | 51.3 | 1260.06 | 164 (164) |
| Tajikistan | 0.54 | 434.32 | 51.28 | 383.04 | 57 (57) |
| Thailand | 0.68 | 1079.71 | 51.47 | 1028.25 | 152 (152) |
| Timor-Leste | 0.44 | 759 | 51.24 | 707.76 | 120 (120) |
| Togo | 0.41 | 316.8 | 51.47 | 265.34 | 27 (25) |
| Tokelau | 0.69 | 1330.05 | 51.4 | 1278.65 | 167 (167) |
| Tonga | 0.63 | 1055.83 | 51.43 | 1004.4 | 149 (149) |
| Trinidad and Tobago | 0.77 | 426.86 | 51.29 | 375.58 | 54 (54) |
| Tunisia | 0.68 | 302.09 | 51.43 | 250.66 | 24 (24) |
| Turkey | #N/A | 838.9 | 51.4 | 787.5 | 132 (132) |
| Turkmenistan | 0.68 | 371.19 | 51.39 | 319.79 | 41 (41) |
| Tuvalu | 0.58 | 1211.16 | 51.35 | 1159.81 | 159 (159) |
| Uganda | 0.42 | 496.19 | 51.45 | 444.73 | 74 (74) |
| Ukraine | 0.76 | 517.36 | 51.51 | 465.85 | 78 (79) |
| United Arab Emirates | 0.85 | 454.5 | 51.29 | 403.21 | 64 (64) |
| United Kingdom | 0.86 | 1587.79 | 51.41 | 1536.38 | 182 (182) |
| United Republic of Tanzania | 0.45 | 404.51 | 51.26 | 353.24 | 51 (51) |
| United States Virgin Islands | 0.82 | 647.5 | 51.28 | 596.22 | 100 (100) |
| United States of America | 0.86 | 1755.12 | 51.45 | 1703.67 | 189 (189) |
| Uruguay | 0.72 | 1442.42 | 51.26 | 1391.16 | 171 (171) |
| Uzbekistan | 0.66 | 356.9 | 51.29 | 305.61 | 38 (38) |
| Vanuatu | 0.47 | 665.58 | 51.4 | 614.17 | 108 (108) |
| Venezuela (Bolivarian Republic of) | 0.6 | 1073.86 | 51.42 | 1022.43 | 150 (150) |
| Viet Nam | 0.63 | 1079.5 | 51.36 | 1028.14 | 151 (151) |
| Yemen | 0.45 | 376.57 | 51.5 | 325.07 | 43 (43) |
| Zambia | 0.51 | 611.6 | 51.45 | 560.15 | 95 (95) |
| Zimbabwe | 0.47 | 906.35 | 51.47 | 854.89 | 137 (137) |
| LOLC |  |  |  |  |  |
| Afghanistan | 0.34 | 821.47 | 74.21 | 747.26 | 77 (77) |
| Albania | 0.71 | 1511.9 | 74.24 | 1437.65 | 142 (142) |
| Algeria | 0.66 | 336.06 | 74.17 | 261.89 | 12 (12) |
| American Samoa | 0.72 | 2434.14 | 74.57 | 2359.57 | 180 (180) |
| Andorra | 0.87 | 621.57 | 74.22 | 547.36 | 55 (55) |
| Angola | 0.45 | 650.93 | 74.3 | 576.64 | 58 (58) |
| Antigua and Barbuda | 0.75 | 797.79 | 74.62 | 723.16 | 75 (75) |
| Argentina | 0.72 | 1497.08 | 74.61 | 1422.48 | 140 (140) |
| Armenia | 0.7 | 1109.89 | 74.31 | 1035.58 | 108 (108) |
| Australia | 0.84 | 2413.3 | 74.63 | 2338.66 | 178 (178) |
| Austria | 0.85 | 2242.68 | 74.26 | 2168.42 | 174 (174) |
| Azerbaijan | 0.69 | 737.42 | 74.25 | 663.17 | 66 (66) |
| Bahamas | 0.81 | 965.71 | 74.61 | 891.1 | 93 (93) |
| Bahrain | 0.75 | 1957.13 | 74.29 | 1882.84 | 164 (164) |
| Bangladesh | 0.49 | 342.34 | 74.25 | 268.1 | 13 (13) |
| Barbados | 0.75 | 840.66 | 74.63 | 766.02 | 78 (78) |
| Belarus | 0.78 | 588.42 | 74.18 | 514.24 | 51 (51) |
| Belgium | 0.85 | 2125.17 | 74.62 | 2050.55 | 169 (169) |
| Belize | 0.61 | 767.01 | 74.61 | 692.4 | 70 (70) |
| Benin | 0.37 | 420.21 | 74.19 | 346.02 | 27 (27) |
| Bermuda | 0.82 | 1674.58 | 74.34 | 1600.24 | 151 (151) |
| Bhutan | 0.47 | 451.41 | 74.34 | 377.07 | 32 (32) |
| Bolivia (Plurinational State of) | 0.6 | 1584.64 | 74.63 | 1510.01 | 147 (147) |
| Bosnia and Herzegovina | 0.72 | 1919.63 | 74.22 | 1845.41 | 163 (163) |
| Botswana | 0.64 | 1167.78 | 74.18 | 1093.6 | 116 (116) |
| Brazil | 0.65 | 1404.53 | 74.2 | 1330.33 | 131 (131) |
| Brunei Darussalam | 0.81 | 3357.99 | 74.29 | 3283.7 | 193 (193) |
| Bulgaria | 0.77 | 1378.47 | 74.37 | 1304.1 | 127 (127) |
| Burkina Faso | 0.29 | 376.07 | 79.73 | 296.33 | 16 (16) |
| Burundi | 0.29 | 335.81 | 79.8 | 256.01 | 11 (11) |
| Cabo Verde | 0.53 | 1467.78 | 74.63 | 1393.15 | 138 (138) |
| Cambodia | 0.47 | 1499.61 | 74.61 | 1425 | 141 (141) |
| Cameroon | 0.48 | 559.42 | 74.47 | 484.95 | 44 (44) |
| Canada | 0.87 | 3845.77 | 74.57 | 3771.2 | 198 (198) |
| Central African Republic | 0.31 | 446.74 | 74.27 | 372.47 | 31 (31) |
| Chad | 0.24 | 387.86 | 79.78 | 308.08 | 19 (19) |
| Chile | 0.77 | 1544.66 | 74.62 | 1470.04 | 144 (144) |
| China | 0.72 | 3507 | 74.28 | 3432.72 | 195 (195) |
| Colombia | 0.66 | 1237.23 | 74.27 | 1162.96 | 120 (120) |
| Comoros | 0.48 | 566.31 | 74.32 | 491.99 | 47 (47) |
| Congo | 0.58 | 899.64 | 74.8 | 824.84 | 83 (83) |
| Cook Islands | 0.78 | 1581.99 | 74.32 | 1507.67 | 146 (146) |
| Costa Rica | 0.7 | 757.44 | 74.28 | 683.16 | 67 (67) |
| Croatia | 0.8 | 1968.5 | 74.7 | 1893.79 | 166 (166) |
[truncated: 140,776 more chars]
